# Deciphering the Transcriptional Landscape of Human Pluripotent Stem Cell-Derived GnRH Neurons: The Role of Wnt Signaling in Patterning the Neural Fate

**DOI:** 10.1093/stmcls/sxac069

**Published:** 2022-09-25

**Authors:** Yafei Wang, Shrinidhi Madhusudan, Ludovica Cotellessa, Jouni Kvist, Nazli Eskici, Venkatram Yellapragada, Kristiina Pulli, Carina Lund, Kirsi Vaaralahti, Timo Tuuri, Paolo Giacobini, Taneli Raivio

**Affiliations:** Stem Cells and Metabolism Research Program, Research Programs Unit, and Department of Physiology, Faculty of Medicine, University of Helsinki, Helsinki, Finland; Stem Cells and Metabolism Research Program, Research Programs Unit, and Department of Physiology, Faculty of Medicine, University of Helsinki, Helsinki, Finland; Univ. Lille, Inserm, CHU Lille, Laboratory of Development and Plasticity of the Postnatal Brain, Lille Neuroscience & Cognition, UMR-S1172, Lille, France; Stem Cells and Metabolism Research Program, Research Programs Unit, and Department of Physiology, Faculty of Medicine, University of Helsinki, Helsinki, Finland; Stem Cells and Metabolism Research Program, Research Programs Unit, and Department of Physiology, Faculty of Medicine, University of Helsinki, Helsinki, Finland; Stem Cells and Metabolism Research Program, Research Programs Unit, and Department of Physiology, Faculty of Medicine, University of Helsinki, Helsinki, Finland; Stem Cells and Metabolism Research Program, Research Programs Unit, and Department of Physiology, Faculty of Medicine, University of Helsinki, Helsinki, Finland; Folkhälsan Research Center, Helsinki, Finland; Stem Cells and Metabolism Research Program, Research Programs Unit, and Department of Physiology, Faculty of Medicine, University of Helsinki, Helsinki, Finland; New Children’s Hospital, Pediatric Research Center, Helsinki University Hospital, Helsinki, Finland; Department of Obstetrics and Gynecology, Helsinki University Hospital, Helsinki, Finland; Univ. Lille, Inserm, CHU Lille, Laboratory of Development and Plasticity of the Postnatal Brain, Lille Neuroscience & Cognition, UMR-S1172, Lille, France; Stem Cells and Metabolism Research Program, Research Programs Unit, and Department of Physiology, Faculty of Medicine, University of Helsinki, Helsinki, Finland; New Children’s Hospital, Pediatric Research Center, Helsinki University Hospital, Helsinki, Finland

## Abstract

Hypothalamic gonadotropin-releasing hormone (GnRH) neurons lay the foundation for human development and reproduction; however, the critical cell populations and the entangled mechanisms underlying the development of human GnRH neurons remain poorly understood. Here, by using our established human pluripotent stem cell-derived GnRH neuron model, we decoded the cellular heterogeneity and differentiation trajectories at the single-cell level. We found that a glutamatergic neuron population, which generated together with GnRH neurons, showed similar transcriptomic properties with olfactory sensory neuron and provided the migratory path for GnRH neurons. Through trajectory analysis, we identified a specific gene module activated along the GnRH neuron differentiation lineage, and we examined one of the transcription factors, *DLX5*, expression in human fetal GnRH neurons. Furthermore, we found that Wnt inhibition could increase *DLX5* expression and improve the GnRH neuron differentiation efficiency through promoting neurogenesis and switching the differentiation fates of neural progenitors into glutamatergic neurons/GnRH neurons. Our research comprehensively reveals the dynamic cell population transition and gene regulatory network during GnRH neuron differentiation.

## Graphical Abstract

**Graphical Abstract:**
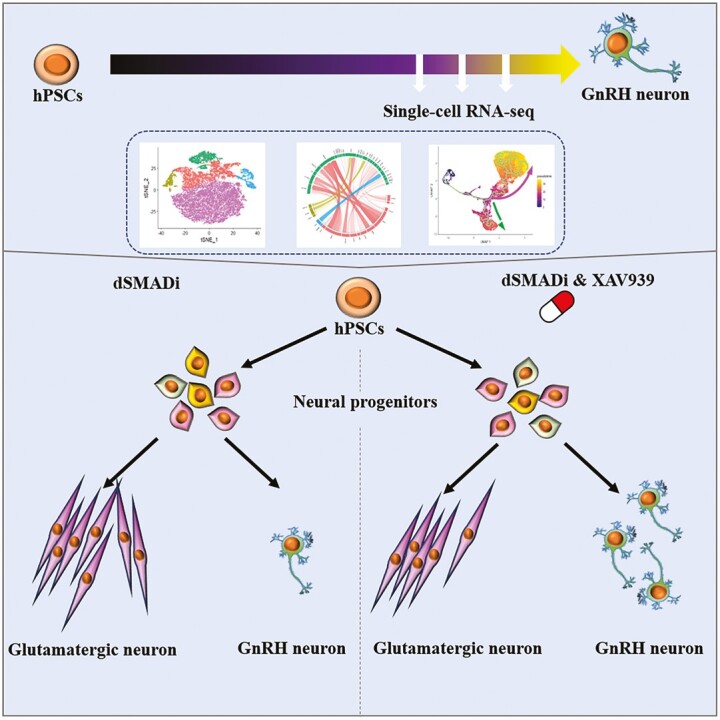

**Significance Statement**

This study first identified and characterized diverse cell types generated along the GnRH neuron differentiation protocol. Additionally, by analyzing the differentiation trajectories, we improved the efficiency of GnRH neuron differentiation with Wnt signaling inhibition.

## Introduction

Gonadotropin-releasing hormone (GnRH) neurons originate in the olfactory placode region and subsequently migrate from the nose to the brain along the terminal, vomeronasal, and olfactory nerves.^1-4^ GnRH neurons residing in the hypothalamus secrete GnRH decapeptide in the hypophyseal portal system, and thereby control the synthesis and release of pituitary gonadotropins, ie, luteinizing hormone (LH) and follicle-stimulating hormone (FSH), which then stimulate gonadal steroidogenesis and gametogenesis.^5^ Disruption of GnRH neuron development, GnRH signaling or GnRH decapeptide secretion can lead to congenital hypogonadotropic hypogonadism (CHH) featuring complete or partial pubertal failure and infertility. Until now, mutations in more than 60 genes have been implicated in CHH,^6^ and several genes, such as *Dlx* family members, *Otx2*, *Pou2f1*, and *Msx1*, have been identified as *Gnrh* promoter modulators in murine GnRH neuron models.^7,8^ Despite the progress in this area, information about how gene-environment interactions significantly influence GnRH neuron development in humans is still fragmentary.

Human pluripotent stem cells (hPSCs), which can self-renew indefinitely in culture while maintaining the ability to become any cell type in the human body,^9,10^ provide a possibility to model the development and function of human GnRH neurons in vitro. Accordingly, we have previously developed a protocol to differentiate hPSCs into GnRH decapeptide-secreting neurons with dual-SMAD inhibition (dSMADi), followed by FGF8 treatment and Notch inhibition.^11^ By now, we have used this model to investigate the role of *MKRN3* in GnRH neuron differentiation^12^ and to describe transcriptome profiles of the putative GnRH neuron progenitors and early postmitotic GnRH neurons with bulk RNA sequencing.^13^ The efficiency of our protocol is, however, approximately 15%,^11^ which suggests that it generates multiple cell types. Single-cell RNA sequencing (scRNA-seq) can reveal the heterogeneity of cells, allow the identification of rare cell populations, uncover putative regulatory relationships between genes, and track the developmental trajectories of distinct cell lineages during their differentiation.^14^

Here, we aimed to identify the developmental trajectories that lead to GnRH neuron fate in vitro using scRNA-seq. We defined emerging cell types along the differentiation process based on their global gene-expression profiles to create a precise cell-by-cell description of in vitro GnRH neuron differentiation and reconstructed the developmental trajectory of GnRH neuron specification. Based on the development trajectory, we identified 443 putative factors involved in the patterning of GnRH neuron differentiation and examined the expression of DLX5, one of the most noticeable *DLX* family genes, in human GnRH neurons both in vitro and in vivo. Furthermore, we observed that Wnt signaling is downregulated along the GnRH neuron specification. Given that Wnts could play a role in murine frontonasal development,^15,16^ and Wnt signaling regulates neural progenitors (NPs) heterogeneity and specifies their regional identity,^17^ we investigated the Wnt effect on the GnRH neuron differentiation by applying XAV939, a Wnt inhibitor, during dSMADi phase. Remarkably, we found that Wnt inhibition affects the cell fate determination already during the first 10 days, and significantly improved the yield of hPSC-derived GnRH neurons.

Together, the identified transcription factors and the role of Wnt signaling as a determinant of GnRH neuron fate patterning have potential implications for mechanisms of puberty, and drug development of contraception and hormone-dependent cancer.

## Materials and Methods

### Stem Cell Maintaining and Differentiation

Human embryonic stem cell line (WA09) and 2 hiPSC lines HEL11.4 and HEL24.3 were used in this study.

Human pluripotent stem cells were grown and expanded in monolayer on plastic cell culture dishes (Corning) coated with Matrigel (CORNING, Cat. 356231) in mTeSR1 (STEMCELL, Cat. 85850) cell culture medium, and dissociated for replating using 0.5 mM Ultrapure EDTA (15575-038, Invitrogen) in PBS (Corning) every 5 days. Before the experiment started, the cells were grown until approximately 95% confluence, covering most of the surface area of the dish. GnRH neuron differentiation was performed as described before.^11^ In brief, N2B27 medium (Neurobasal medium supplemented with N2 and B27 supplement, in 1:1 ratio with DMEM/F12, and containing 1× Glutamax (ThermoFisher Scientific), 100 U/mL penicillin, and 100 µg/mL streptomycin (Sigma)) was supplemented with 2 µM dorsomorphin (Selleckchem, Cat. S7306) and 10 µM SB431542 (Sigma, Cat. S4317), and replaced daily during the first 10 days. On day10, cells were replated in 1:2 ratio using 200 U/mL Collagenase IV (ThermoFisher Scientific) onto Matrigel-coated dishes in N2B27 supplemented with 10 µM Y-27632 (Selleckchem, Cat. S1049). From day 11 to day 20, N2B27 was supplemented with 100 ng/mL of FGF8 (PeproTech, Cat. AF-100-25). Cell vials were frozen for storage on day 17 by dissociation with EDTA and resuspension in a freezing medium containing N2B27, 10% DMSO HybriMax (D2650, Sigma-Aldrich) and 10 µM Y-27632 in 2 mL cryovial tubes (ThermoFisher Scientific) stored at −150 °C. Cells were thawed in N2B27 containing 100 ng/mL FGF8 and 10 µM Y-27632, and grown until they were confluent (approximately 3 days). On day 20, cells were detached using 0.5 mM EDTA and replated in N2B27 at 1:8 ratio. The next day following the split, N2B27 was supplemented with 20 µM DAPT (Selleckchem, Cat. S2215) and the medium was replaced every 2 days. For Wnt inhibition, based on the original differentiation protocol, we added 10 µM XAV939 (Selleckchem, Cat. S1180) from day 0 to day 10.

### Single-cell Isolation

Samples from days 22, 24, and 26: Medium was removed and cells were washed one time with DMEM/F12 and one time with sterile PBS, and incubated in 2 mg/mL Papain solution (STEMCELL, Cat. 07465) for 7 min at 37 °C. An equal volume of 1% BSA in PBS without Ca^2+^ and Mg^2+^ was added to the cell suspension, and cells were suspended by pipetting up and down approximately 15 times using a 1-mL pipette tip. The suspension was moved to a 2 mL Eppendorf tube and centrifuged at 300 × *g* for 3 min. The supernatant was removed and cells were resuspended in 1% BSA + 2 µM EDTA in PBS without Ca^2+^ and Mg^2+^, where volume was adjusted based on a viable cell count of approximately 1000 cells/µL using LUNA-FL Dual Fluorescence Cell Counter. The cell suspension was filtered 2-4 times through a 35-µm cell strainer tube cap (Falcon, Cat. 352235), and processed for scRNA-seq library construction and sequencing.

Samples from day 0, day 5, and day 10: medium was removed and cells were washed one time with sterile PBS and incubated in Accutase (ThermoFisher, Cat. A1110501) for 4 min at 37 °C. An equal volume of 1% BSA in PBS without Ca^2+^ and Mg^2+^ was added to the cell suspension, and cells were suspended by pipetting up and down approximately 15 times using a 1-mL pipette tip. The suspension was moved to a 2-mL Eppendorf tube and centrifuged at 300 × *g* for 3 min. The supernatant was removed and cells were resuspended in 1% BSA + 2 µM EDTA in PBS without Ca^2+^ and Mg^2+^. Cell numbers were counted using LUNA-FLTM Dual Fluorescence Cell Counter for further labeling.

### Cell Multiplexing Oligo Labeling for Single-cell RNA Sequencing

Five samples (D0, D5_dSMADi, D5_XAV939, D10_dSMADi, and D10_XAV939) were labeled with cell multiplexing oligo according to the manufacturer’s instructions (10× Genomics, 3ʹ CellPlex Kit Set A, Cat. 1000261). Briefly, 2 × 10^6^ cells from each sample were suspended in 100 µL Cell multiplexing Oligo (301–305), and incubated for 5 minutes at room temperature. The samples were washed by Wash & Resuspension Buffer 3 times. The cells were counted and then mixed as calculated for scRNA-seq library construction.

### scRNA-seq Library Construction and Sequencing

Single-cell RNA-sequencing was performed using the chromium single-cell 3ʹ RNA-sequencing system (10× Genomics, Pleasanton, CA) with the Reagent Kit v3 according to the manufacturer’s instructions. Briefly, the cells were loaded into chromium single cell B chip (10× Genomics, Pleasanton, CA) and gel beads in emulsion (GEM) generation were performed aiming at 10 000 cell capture per sample. Subsequent cDNA purification, amplification, and library construction were performed as instructed. Sample libraries were sequenced on an Illumina NovaSeq 6000 system using following read lengths: 28 bp (Read 1), 8 bp (i7 Index), 0 bp (i5 Index), and 89 bp (Read 2) aiming at 50 000 reads per cell sequencing depth.

The Cell Ranger v3.1 mkfastq and count pipelines (10× Genomics, Pleasanton, CA) were used to demultiplex and convert chromium single-cell 3ʹ RNA-sequencing barcodes and read data to FASTQ files and to generate aligned reads and gene-cell matrices. Reads were aligned to the human genome GRCh38.

### Processing the scRNA-seq Data

All main processing steps were performed with Seurat v.3.1. Quality control was first performed independently on each library to find appropriate filtering thresholds (see Supplementary Table S6 for detailed parameters of each library). Expression matrices for each sample were loaded into R as Seurat objects, retaining only cells that fit the standards. Genes significantly enriched in each cluster were identified using the default algorithm in Seurat.^18,19^ Functional annotation of the resulting marker genes list related to GO terms were performed using g:Profiler (https://biit.cs.ut.ee/gprofiler/gost). Intercellular communication is modeled by the NicheNet package.^20^ Trajectory analysis of the GnRH neuron development was performed using Monocle (v3).^21,22^ A comparison of the similarity between the Glut cluster identified from our scRNA-seq data and different cell types identified in the reference transcriptomic datasets (olfactory cleft and turbinate tissue scRNA-seq datasets) was performed using SingleR.^23^

### Immunofluorescence Assays

The cells grown on the Matrigel coated coverslips were fixed in 4% paraformaldehyde for 15 minutes at room temperature. Triton X-100 (0.25%) was used for permeabilization, followed by blocking reagent (ThermoFisher Scientific, Cat. B10710). Samples were incubated with primary antibodies (Supplementary Table S4) overnight at 4 °C, and then with secondary antibodies for 2 h at room temperature. DAPI was used to counterstain nuclei. After the samples were mounted, they were imaged by using a widefield microscope (Nikon Eclipse Ti-E, Japan) or a confocal microscope (Leica TCS SP8, Japan).

### Human Fetuses

Fetal tissues were made available in accordance with French bylaws (Good Practice Concerning the Conservation, Transformation, and Transportation of Human Tissue to Be Used Therapeutically, published on December 29, 1998). The studies on human fetal tissue were approved by the French agency for biomedical research (Agence de la Biome´ decine, Saint-Denis la Plaine, France, protocol n°: PFS16-002). Non-pathological human fetuses were obtained at GW8.5 and GW13 from pregnancies terminated voluntarily after written informed consent of the parents (Gynaecology Department, Jeanne de Flandre Hospital, Lille, France).

### Multiplex Fluorescence *In Situ* Hybridization (FISH) Combined With Immunofluorescence

Two human fetuses (males; 8.5 and 13 gestational weeks post-amenorrhea; GW) were obtained from voluntarily terminated pregnancy with the parent’s written informed consent (Gynecology Hospital Jean de Flandre, Lille, France). The fetuses were snap-frozen in liquid nitrogen and stored at −80 °C until use. Human tissues were cryosectioned using a CM3050 Leica Cryostat at 16 μm.

FISH was performed on frozen sections of the nasal region of a GW 8.5 and a GW 13 fetus with the RNAscope Multiplex Fluorescent Kit v2 according to the manufacturer’s protocol (Advanced Cell Diagnostics). Specific probe was used to detect *DLX5* (Hs-DLX5-C3 REF 569471-C3) mRNA. Hybridization with a probe against the *Bacillus subtilis* dihydrodipicolinate reductase (*dapB*) gene (320871) was used as a negative control. Immunofluorescence against GnRH was performed, after RNAscope staining, as previously reported.^24^ Briefly, the sections were rinsed with 0.1 M PBS and incubated at 4 °C overnight with the previously validated^3^ guinea-pig anti-GnRH (a generous gift from Dr. Erik Hrabovszky, Laboratory of Endocrine Neurobiology, Institute of Experimental Medicine of the Hungarian Academy of Sciences, Budapest, Hungary) diluted at 1:1000 in 0.1 M PBS containing 0.3% Triton X-100 and 10% normal donkey serum. The sections were then washed in PBS and incubated for 1 h with AlexaFluor 488-conjugated secondary antibody (Molecular Probes, Invitrogen, San Diego, CA) diluted 1:400 in PBS and counterstained with DAPI nuclear staining (1:10 000; Molecular Probes, Invitrogen, San Diego, CA). Sections were mounted using Mowiol (Calbiochem, USA) and analyzed using an LSM 710 confocal microscope (Zeiss).

### Flow cytometry

The cells were dissociated into single cells and suspended in FACS buffer (10% (v/v) FBS and 1 mM EDTA in 1× HBSS). Flow cytometry was performed using a Sony SH800z sorter at the Biomedicum Flow Cytometry Unit, University of Helsinki. Data analyses were performed with FlowJo software (v10; FlowJo LLC, Ashland, OR, USA).

### Real-time PCR

Total RNA was extracted using NucleoSpin RNA Plus (MACHEREY-NAGEL, 740984). mRNA was reverse transcribed using an iScript cDNA Synthesis Kit (Bio-Rad, 170-8891). Real-time quantitative PCR was performed with cDNA using HOT FIREPol EvaGreen qPCR Mix Plus (Solis BioDyne) and LightCycler 480 (Roche) for 45 cycles of 95 °C for 15 s, 60 °C for 20 s and 72 °C for 20 s. mRNA expression was normalized to cyclophilin G (*PPIG*) and all the primers used are listed in Supplementary Table S5.

### Cell Migration Assay

Cells on day 20 were spited and seeded into the Incucyte Imagelock 96-well plates. A total of 20 µM DAPT was given on day 21 and media change was done every other day. The plate was imaged using an IncuCyte S3 Live Cell Analysis System (Sartorius Corporation) from day 22 to day 26 at 1 h intervals.

### Quantification of Secreted GnRH

Cell culture medium was collected concurrently with medium refreshments at indicated time points. Medium was centrifuged shortly (1200 × *g* for 10 minutes at 4 °C) and stored in −80 °C. Secreted GnRH decapeptide was quantified by competitive Fluorescent Enzyme Immunoassay (EIA, Phoenix Pharmaceuticals, Inc., FEK-040-02) according to manufacturer’s instructions. GnRH concentrations were determined from the standard curve fitted with 4-point parameter logistic nonlinear regression model.

### Statistical Tests

The results are shown as means ± SD of data from at least 3 separate experiments. Statistical analyses were performed using GraphPad Prism 8 (Graphpad Software) and FlowJo 10.7 software (BD Biosciences). For the comparison between 2 groups, we used *t*-test analysis. For the comparisons among 3 groups (D0, D25-dSMADi, and D25-XAV939), we used one-way ANOVA analysis (Tukey’s multiple comparisons test). For the comparisons among 5 groups (D0, D5-dSMADi, D5-XAV939, D10-dSMADi, and D10-XAV939) and ELISA results, we used 2-way ANOVA analysis (Sidak’s multiple comparisons test). *P* values of .05 or less were considered statistically significant, as indicated by asterisks in the figures.

## Results

### Diverse Cell Populations Are Generated by the GnRH Neuron Differentiation Protocol

**Figure 1.**
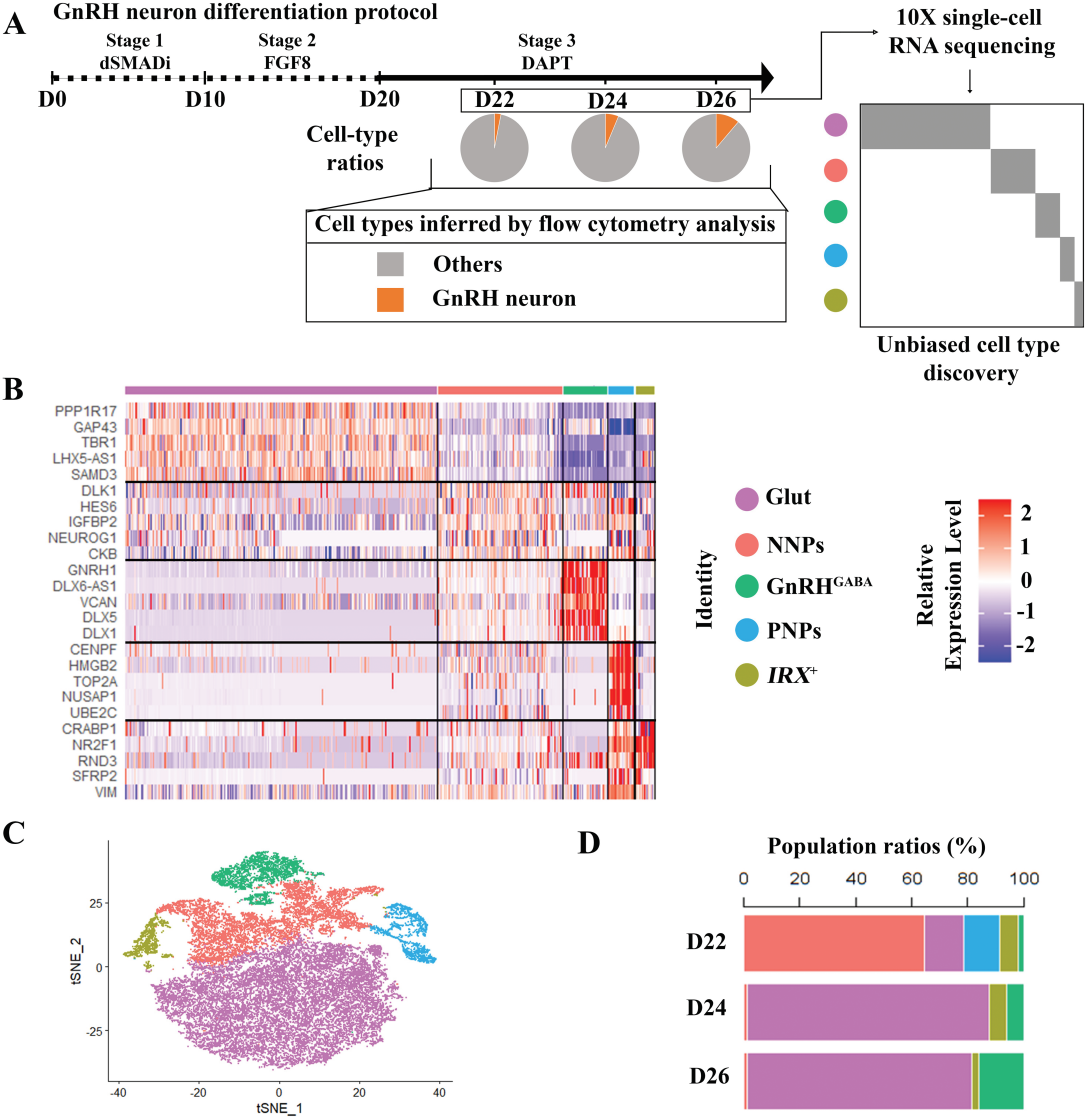
scRNA-seq reveals 5 cell clusters along GnRH neuron differentiation. (**A**) Schematic of GnRH neuron differentiation protocol and GnRH neuron population ratios identified by flow cytometry on day 22 (D22) (3.2 ± 0.12%), day 24 (D24) (6.3 ± 0.05%), and day 26 (D26) (10.9 ± 0.44%). (**B**) Heatmap of genes significantly enriched within each individual cluster. Single cells are shown in columns; genes are shown in rows. Top 5 differential genes of each cell cluster are shown. (C) t-SNE projections of cells sampled from all 3 days (D22, D24, and D26). Cells are colored according to their assigned cluster. (D) Components of different sample days (D22, D24, and D26) in each cluster during GnRH neuron differentiation. D = days of differentiation.

In the first phase of the GnRH neuron differentiation,^11^ dSMADi is used to produce NPs. This is followed by administration of FGF8 to pattern anterior neural progenitors and, finally, Notch inhibitor DAPT is used to induce neural maturation (Fig. 1A). We first investigated the temporal differentiation efficiency of GnRH neurons with flow cytometry analysis by using the *GNRH1*-*tdTomato* hPSC reporter line described before.^13^ The results showed that the cell population with tdTomato signal increased steadily from day 22 (D22, 3.2% ± 0.12%) to day 26 (D26, 10.9% ± 0.45%) (Fig. 1A and Supplementary Fig. S1).

Next, we performed scRNA-seq of the cells collected on D22, D24, and D26 of differentiation with 10× Genomics (Fig. 1A), and recovered a total of 31 899 cells and 23 113 genes from all the samples (Supplementary Fig. S2). Following data processing, high-quality transcriptomic data were obtained from 29 686 cells, including 9523 cells from D22, 9107 cells from D24, and 11 056 cells from D26 (Supplementary Fig. S2). By performing Seurat analysis with transcriptomes of cells from D22, D24, and D26 separately, and also with integrated datasets (29 686 cells), we identified 5 distinct cell clusters during the differentiation process. These clusters were subsequently annotated based on the expression of known marker genes and an automatic annotation toolkit, scCATCH.^25^ The heatmap shows the expression of the top 5 marker genes across 5 clusters: (i) proliferating neural progenitors (PNPs) that express *HMGB2*, *MKI67*, and *TOP2A* that are markers for cell proliferation; (ii) nonproliferating neural progenitors (NNPs) that express *DLK1*, *NEUROG1*, *NEUROD1/4*, and *SOX2* that most resembles neural progenitors; (iii) GnRH neurons (GnRH^GABA^) that express *GNRH1*, *GAD2*, and *DLX* family genes, such as *DLX1*, *DLX2*, and *DLX5*; (iv) glutamatergic neurons (Glut) that express *PPP1R17*, *TBR1*, and *LHX* family genes, such as *LHX1*, *LHX2*, *LHX5*, and *LHX9*; and (v) *IRX*^+^ which highly expresses *CRABP1*, *NR2F1,* and *IRX* family genes (Fig. 1B, Supplementary Table S1). We used *t*-distributed stochastic neighbor embedding (tSNE) to visualize the cell clusters (Fig. 1C, Supplementary Fig. S3). All 5 cell clusters were present on D22. However, the PNPs (D22: 13.0%) waned away on D24, and the proportion of NNPs also shrunk dramatically after D22. Conversely, the proportion of both Glut and GnRH^GABA^ populations increased markedly along the differentiation (Fig. 1D). Thus, we identified 5 emergent cell types at the last stage of GnRH neuron differentiation.

### Neural Progenitors Perform Different Functions and Have Different Differentiation Capacities

**Figure 2.**
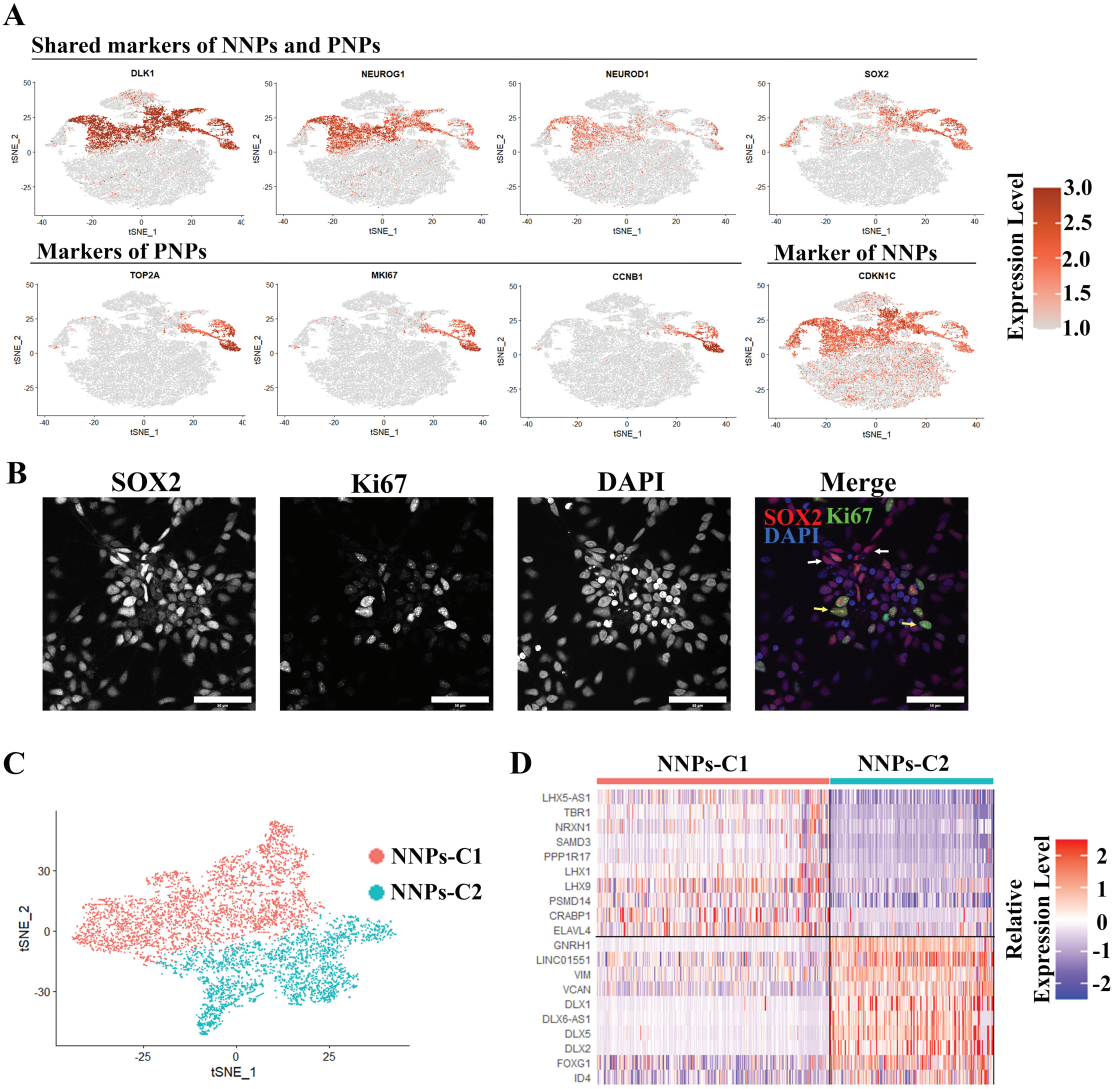
Characterization of neural progenitors. (**A**) Expression of NNPs markers and PNPs markers projected onto the t-SNE map. (**B**) Immunofluorescence staining for 2 major neural progenitors (NNPs (SOX2^+^/Ki67^−^), and PNPs (SOX2^+^/Ki67^+^)). White arrows indicate NNPs, and yellow arrows indicate PNPs. Scale bar = 50 µm. (**C**) t-SNE projections of NNPs, which are further divided into 2 sub-clusters (NNPs-C1 and NNPs-C2). (**D**) Heatmap of differentially expressed genes between NNPs-C1 and NNPs-C2.

Based on the Seurat analysis, 2 types of NPs were identified, including PNPs and NNPs. Both expressed *SOX2*, *DLK1*, and basic helix-loop-helix family genes, including *NEUROG1* and *NEUROD1* (Fig. 2A) that have been reported to be expressed in neural progenitors in vivo.^26-29^ PNPs highly expressed cell-cycle-associated genes (eg, *MKI67, TOP2A, CCNB1,* and *CCNB2*), while the NNPs highly expressed *STMN2*, and *CDKN1C,* a cell-cycle-inhibitor gene (Fig. 2A and Supplementary Fig. S13). We applied Gene Ontology (GO) enrichment analysis based on the profiles of differentially expressed genes (DEGs) between these 2 types of NPs and identified that neuron differentiation and neurogenesis were enriched in NNPs, and cell division and mitotic cell cycle in PNPs (Supplementary Fig. S4A and S4B). Consistent with the gene expression profiles, immunostaining also verified the 2 types of NPs (SOX2^+^/Ki67^+^ and SOX2^+^/Ki67^−^) on D22 (Fig. 2B). Furthermore, we performed an unsupervised sub-clustering analysis of the cells in NNPs using Seurat, and we identified 2 sub-clusters from NNPs, named NNPs-C1 and NNPs-C2 (Fig. 2C). NNPs-C1 highly expresses *PPP1R17, TBR1, LHX1*, and *LHX9*, while the NNPs-C2 is marked by higher expression of *GNRH1*, *DLX1*, *DLX2*, and *DLX5* (Fig. 2D and Supplementary Fig. S5). These genes are the markers for the Glut, and GnRH^GABA^ cell clusters, respectively (Fig. 1B, Supplementary Table S1), which suggests that the NNPs have already committed to different lineages after exiting the cell cycle. Together, these data suggest that PNPs, which maintain the capacity of cell proliferation, are probably responsible for maintaining NPs pool, while NNPs have exit the pool of proliferative cells and exhibit differentiation capabilities for different neuronal lineages.

### Glut Provides the Migratory Path for GnRH^GABA^ and Displays Similar Transcriptional Properties with Olfactory Neurons

**Figure 3.**
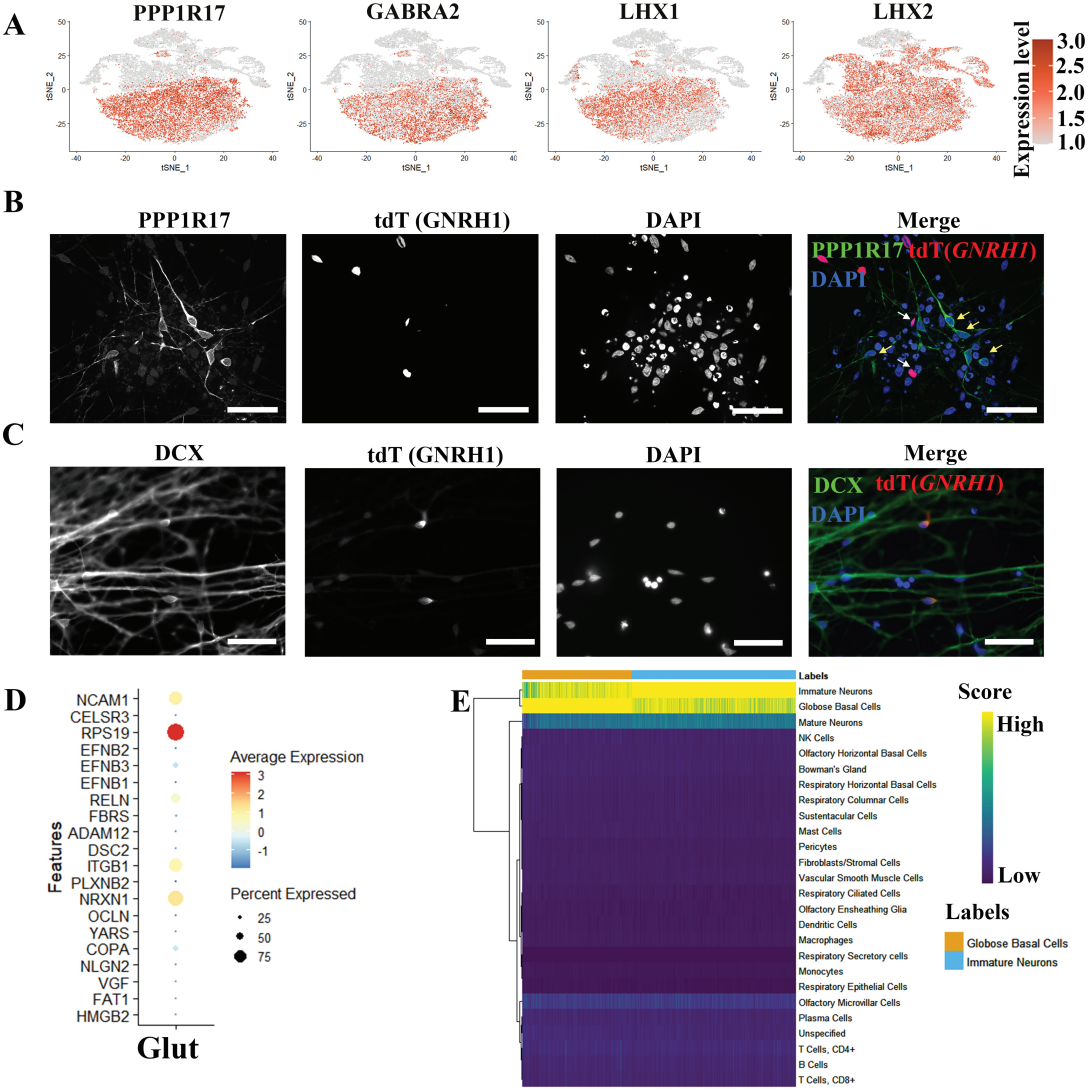
Characterization of glutamatergic neurons. (**A**) Expression of Glut markers projected onto the t-SNE map. (**B**) Immunofluorescence staining for Glut marker (PPP1R17) showing non co-localization with GnRH neurons. White arrows indicate GnRH neurons, and yellow arrows indicate Glut. Scale bar = 50 μm. (**C**) Immunofluorescence staining for migratory neuron marker (DCX) showing differentiated neurons are migratory. Scale bar = 50 µm. (**D**) Dot plot showing the expression level of prioritized ligands in Glut. (**E**) Comparing Glut cluster with published scRNA-seq dataset from olfactory cleft and turbinate tissues.

The cluster of Glut strongly expresses glutamatergic neuron markers, such as *SLC17A6* (also named as *VGLUT2*), *PPP1R17*, *GABRA2*, *LHX1*, and *LHX2* (Fig. 3A, Supplementary Table S1). GO enrichment analysis showed that neurogenesis, neuron differentiation, nervous system development, and cell projection morphogenesis were enriched in Glut cluster (Supplementary Fig. S4C). Through immunostaining of the differentiated GnRH reporter cell line (*GNRH1*-*tdTomato* hPSC, D25/D26), we verified that Glut (PPP1R17^+^), which are bipolar and show long axons, were non-GnRH neurons (tdTomato^-^) (Fig. 3B). Toward the end of the differentiation (D27), the neurons gathered together, and the PPP1R17^+^ cells (Glut) with extensively long axons formed the bundle of axons, with GnRH neurons set beside (Supplementary Fig. S6). To investigate the mobility of differentiated neurons, we took a video from D22 to D26 and noticed that the cultured cells organize in condensed nodes that are interconnected with bundles of axons. Notably, GnRH neurons (tdTomato^+^) were quite motile because they moved along the interconnecting tracts to integrate into the nodes (Supplementary Video S1). Next, we modeled the communications between Glut cluster and GnRH^GABA^ cluster with Nichenet.^20^ The Glut cluster highly expressed genes such as *NCAM1*, *RELN*, *ITGB1,* and *NRXN1* (Fig. 3D), and these genes regulate neuronal processes such as neuronal migration, and axon pathfinding,^30-34^ which suggests that the Glut may assist in the migration of GnRH^GABA^. Consistently, both Glut and GnRH^GABA^ highly express *DCX*, which is a marker for immature neurons and migratory neurons (Fig. 3C).^35,36^ Given that axons of the olfactory neurons provide the early GnRH neuron migration pathway in vivo,^37^ we compared the Glut cluster with the published single-cell datasets about olfactory cleft and turbinate tissue,^38^ and found that immature neurons, globose basal cells, and mature neurons were the top 3 cell types according to the estimated similarity score. This indicates that Glut is highly similar with the immature olfactory neurons in regard to transcriptomic properties (Fig. 3E). By checking the specific markers, Glut cluster indeed highly expressed *LHX2*, *EMX2*, *GNG8*, and *TUBB*, markers of immature OSNs (Supplementary Fig. S7).^38^ Thus, we identified Glut population generated by GnRH neuron differentiation protocol, with possible correspondence to olfactory neurons.

### Identifying Differentiation Trajectory and Regulatory Genes During GnRH Neuron Differentiation Using Pseudotime Analysis

**Figure 4.**
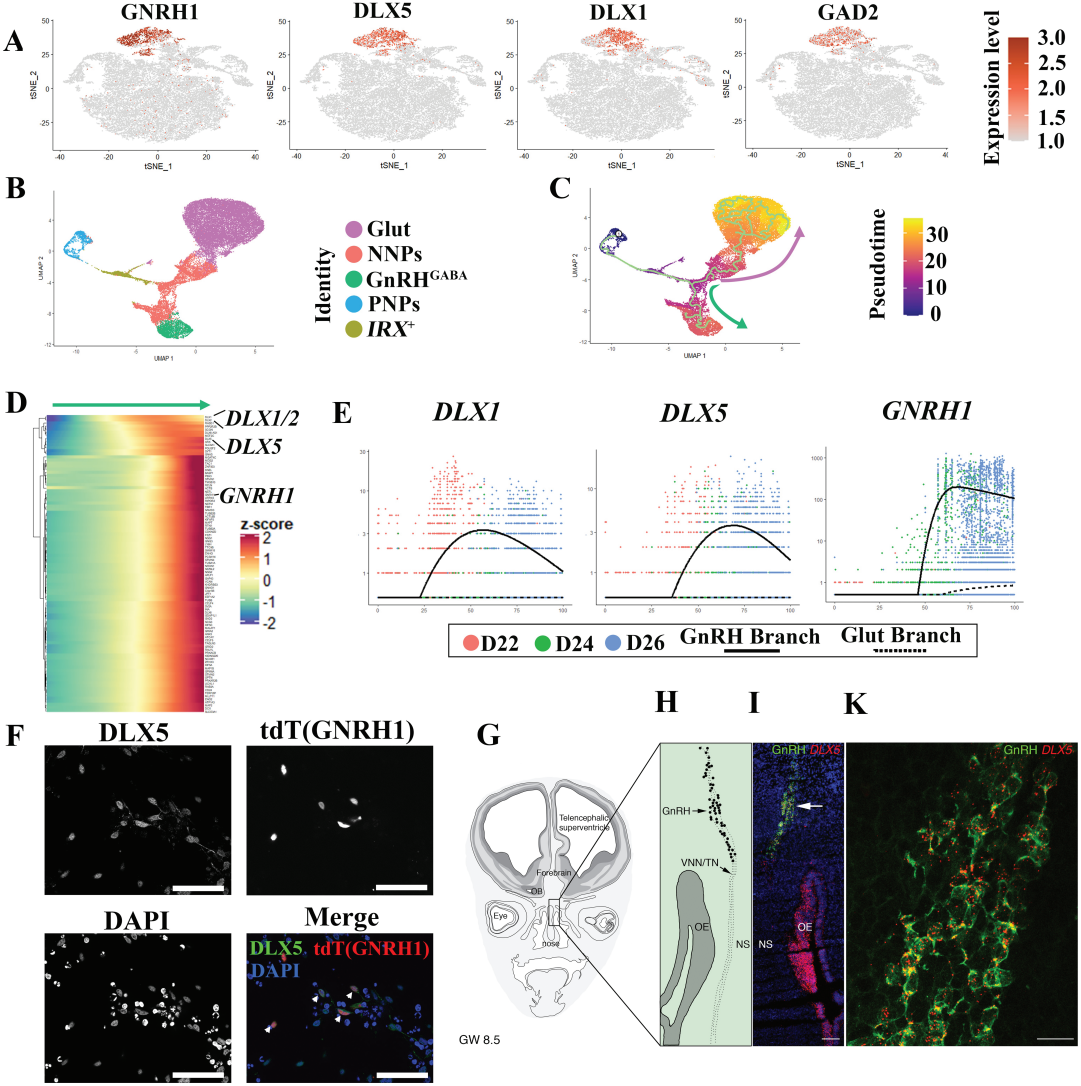
Reconstruction of the GnRH neuron differentiation trajectories. (**A**) Expression of GnRH^GABA^ markers projected onto the t-SNE map. (**B**) UMAP projection of all cells with Monocle analysis. Cells are colored according to their assigned cluster. (**C**) UMAP shading of branch assignment and pseudotime value of each cell. Green arrow shows the GnRH^GABA^ branch, and pink arrow shows the Glut branch. (**D**) Heatmap of differentially expressed genes (upregulated in GnRH^GABA^ branch) identified by Monocle (rows), with cells (columns) ordered according to the pseudotime development. (**E**) Expression of selected marker genes along pseudotime ordering. Dots show expression in single cells. Lines show regression on pseudotime for each branch (solid line, GnRH^GABA^; dash line, Glut). (**F**) Immunofluorescence staining for GnRH^GABA^ marker (DLX5) showing co-localization with GnRH neurons. Scale bar = 50 µm. Arrows indicate the DLX5^+^ GnRH neurons. (**G**) Schematic representation of a coronal section of a GW 8.5 fetal head. Squared box is magnified in (**H**) and it highlights the nasal region where simultaneous immunofluorescence for GnRH (green) and fluorescent in situ hybridization for DLX5 (red) was performed. (**I**) High-power confocal photomicrograph showing GnRH/DLX5 co-expressing migratory neurons (arrowheads). Scale bars: (I) 120 µm, (K) 20 µm. Abbreviations: OB, olfactory bulb; OE, olfactory epithelium, VNN/TN, vomeronasal nerve/terminal nerve; NS, nasal septum.

GnRH neurons were identified GABAergic and termed GnRH^GABA^. This cluster highly express GABAergic neuron markers, such as *SLC32A1* (also named as *VGAT*), *DLX1/2/5*, and *GAD2* (Fig. 4A, Supplementary Table S1) according to our analysis. We next sought to reveal the putative regulators pertinent to GnRH neuron development. We reconstructed the GnRH neuron differentiation trajectories by performing Monocle 3.^22^ After importing the integrated dataset from Seurat into Monocle 3, the cell distribution was displayed with Uniform Manifold Approximation and Projection (UMAP) (Fig. 4B). We found genes that vary in some interesting way across the clusters (Supplementary Fig. S8C and Table S2), and module 18 represents the co-regulated genes highly specific to GnRH^GABA^ cluster. After building the trajectory, we noticed that there is a branch point during the differentiation, one path led by Glut lineage (upwards), and the other by GnRH^GABA^ lineage (downwards) (Fig. 4C). Through analysis of each differentiation lineage, we identified the genes that vary across pseudotime (Fig. 4D, Supplementary Fig. S8A and S8B). Interestingly, in GnRH^GABA^ lineage, *DLX1/2* and *DLX5* are activated before *GNRH1* (Fig. 4D and 4E), and they are not activated in the Glut lineage (Fig. 4E). We observed that the differentiated GnRH neurons (tdTomato^+^) express stronger DLX5 than other cell types in the culture (Fig. 4F). To confirm *DLX5* expression in human GnRH neurons in vivo, we performed RNAscope staining for *DLX5* in human fetuses (8.5 gestational weeks) together with the expression of GnRH. Consistently, all GnRH-immunoreactive neurons expressed *DLX5* throughout the migratory pathway (Fig. 4G-4K). In addition, the olfactory epithelium, which is postulated to be the origin of GnRH neurons,^39^ also highly expresses *DLX5* (Fig. 4I). Together, these results model the trajectories for GnRH differentiation and indicate that the *DLX* family genes, including *DLX5*, are involved in GnRH neuron development.

### Wnt Inhibition Improves GnRH Neuron Differentiation Efficiency

**Figure 5.**
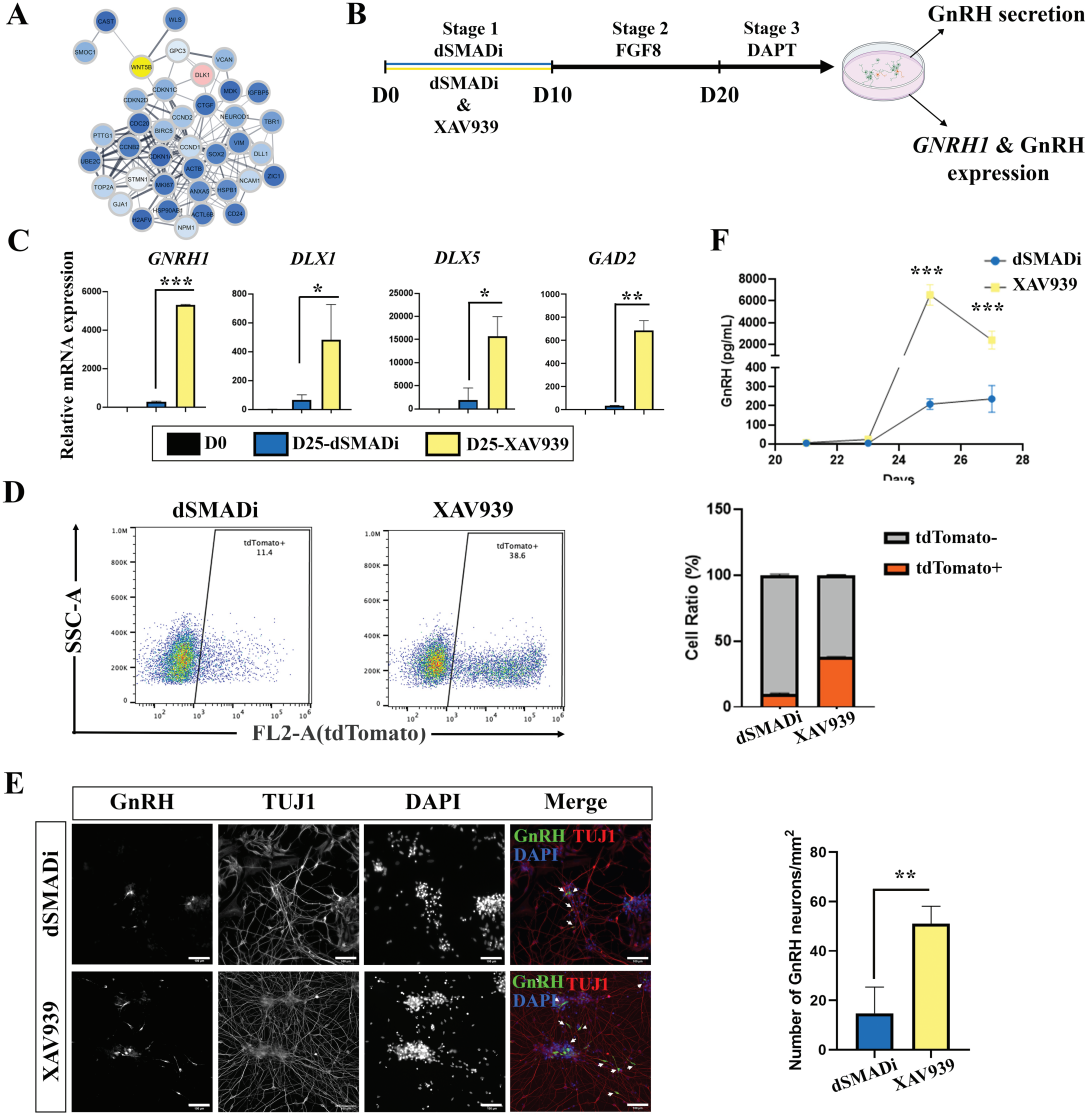
Wnt inhibition improves the efficiency of GnRH neuron differentiation. (**A**) Protein–protein interaction analysis of downregulated genes in GnRH^GABA^ branch. (**B**) Schematic of the differentiation protocols w/o XAV939 treatment. (**C**) RT-qPCR results show that *DLX1*, *DLX5*, and *GAD2* are upregulated with Wnt inhibition along with *GNRH1*. The mRNA expression levels were compared with D0 samples. (**D**) Quantification of the ratio of generated GnRH neurons from flow cytometry analysis results. (**E**) Immunofluorescence staining shows that there are more GnRH neurons (green) generated with Wnt inhibition (XAV939). Arrows indicate GnRH neurons. Scale bar = 100 µm. (**F**) Secretion of GnRH decapeptides were measured with ELISA. D = days of differentiation. Data are represented as mean ± SD. Asterisks indicate the following: *P < .05; **P < .01; ***P < .001.

From the pseudotime analysis, we also noticed that there is a cluster of genes downregulated along the GnRH lineage (Supplementary Fig. S8A). We performed an enrichment analysis and found that those genes were associated with sensory organ development, regulation of neurogenesis, and regulation of Wnt signaling pathway (Supplementary Fig. S9). Protein–protein interaction networks^40^ indicated that *WNT5B* has potential interactions with neuron progenitor marker genes and cell proliferation genes (Fig. 5A). These raised the intriguing possibility that Wnt inhibition might affect the yield of GnRH neurons. Therefore, we administrated XAV939, a Wnt inhibitor, during dSMADi stage (D0 to D10), and examined its effect at the end of differentiation (Fig. 5B). Interestingly, *GNRH1* expression was upregulated significantly by XAV939 treatment. Concomitantly, the expressions of *DLX1*, *DLX5*, and *GAD2* were increased in XAV939 treatment (Fig. 5C). These indicate that Wnt inhibition might enhance the function of GnRH neurons or improve the differentiation efficiency without affecting GnRH neuron properties. To investigate whether Wnt inhibition improves differentiation efficiency of GnRH neurons, we performed these 2 protocols on our GnRH reporter cell line, and used flow cytometry analysis to quantify the GnRH neurons. The results showed that the population of GnRH neurons (tdTomato^+^) increased over 3-fold in XAV939-treated cells (9.7 ± 0.7%) compared with dSMADi samples (37.8 ± 0.2%) (Fig. 5D). Consistently, the immunostaining of the differentiated cells also showed that there are more GnRH-positive neurons in XAV939-treated samples than dSMADi samples (Fig. 5E). Human GnRH is translated as a 96-amino-acid prohormone, which is processed by intracellular proteases to form the secreted decapeptide. We measured the secreted GnRH decapeptide of both conditions (XAV939 and dSMADi samples) in culture medium from D21 to D27. In line with our previous report,^11^ the secreted GnRH decapeptide was detectable since D21, but significantly increased from D25 onward. Moreover, XAV939-treated cells secreted more GnRH decapeptide than control samples (Fig. 5F), which also indicates that Wnt inhibition produced more functional GnRH neurons. To test whether the effect of Wnt inhibition is cell line-dependent, we repeated the differentiation in 2 additional hiPSC lines established from healthy donors (HEL11.4^41^ and HEL24.3^42^) and found that Wnt inhibition increased the *GNRH1* expression in both hiPSC lines (Supplementary Fig. S10). In conclusion, Wnt inhibition during the dSMADi stage increases the yield and the secretion of GnRH neurons.

### Wnt Inhibition Promotes Anterior Ventral GABAergic Neuron Specification

**Figure 6.**
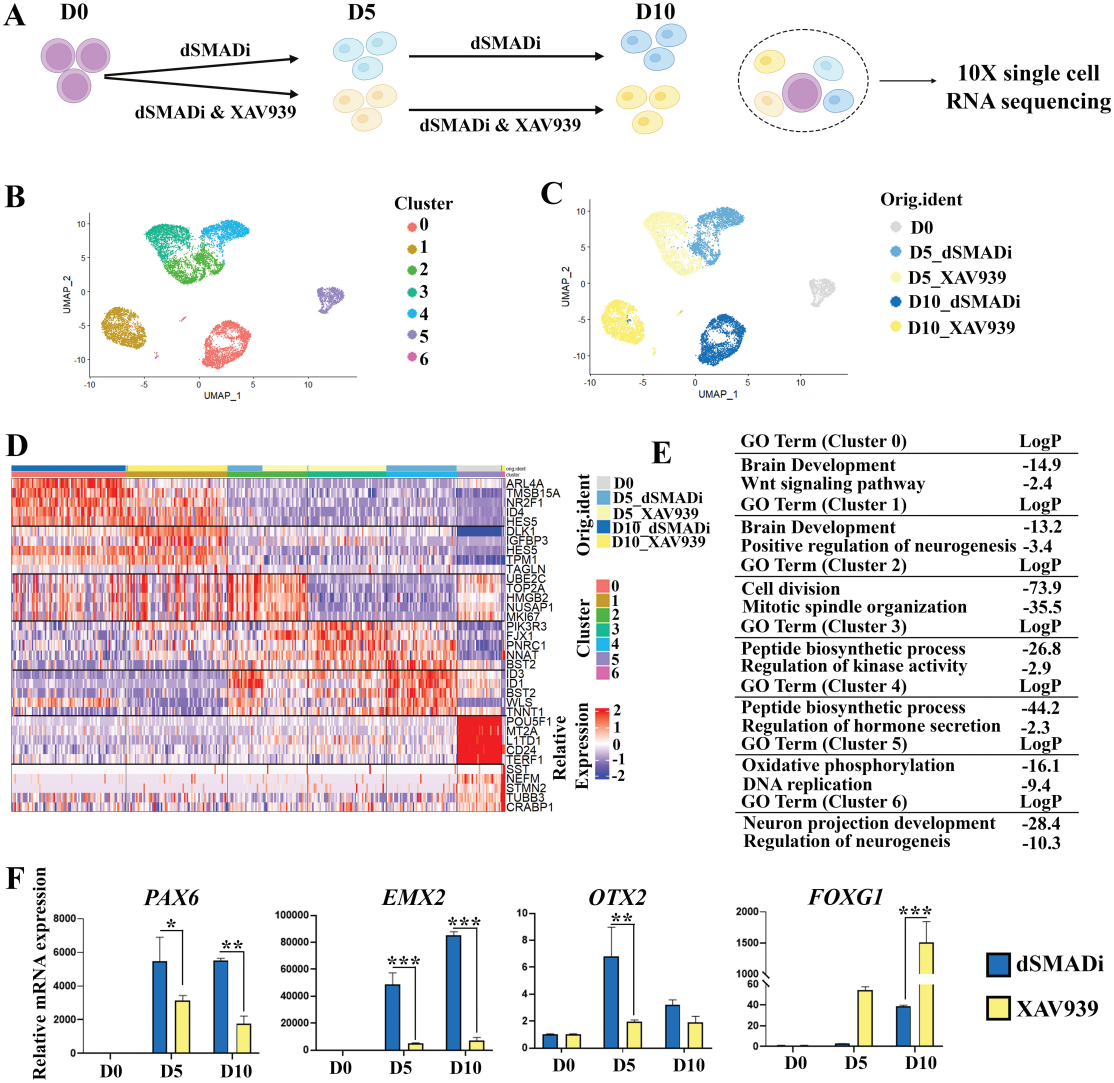
Wnt inhibition promotes anterior ventral neuron specification and neurogenesis progress. (**A**) Schematic of the sample collection for scRNA-seq. (**B**) UMAP projections of cells from all 5 samples. Cells are colored according to their assigned cluster. (**C**) UMAP projections of cells from all 5 samples. Cells are colored according to sample names. (**D**) Heatmap of genes significantly enriched within each individual cluster. Single cells are shown in columns; genes are shown in rows. Top 5 differential genes of each cell cluster are shown. Bars for the cell clusters and sample names are both provided. (**E**) Representative enriched GO terms corresponding to the DEGs of each cluster. (**F**) RT-qPCR results shows that dorsal telencephalic progenitor markers *PAX6*, *OTX2*, and *EMX2* are downregulated, and ventral telencephalic progenitor marker *FOXG1* is upregulated with Wnt inhibition. The mRNA expression levels were compared with D0 samples. D = days of differentiation. Asterisks indicate the following: *P < .05; **P < .01; ***P < .001.

To investigate the mechanism that underlying Wnt inhibition improves differentiation efficiency of GnRH neurons, we performed scRNA-seq with the samples w/o XAV939 treatment (Fig. 6A), including undifferentiated sample (D0, 1008 cells analyzed), differentiating samples treated with only dSMADi (D5_dSMADi, 2480 cells analyzed, and D10_dSMADi, 2778 cells analyzed), and differentiating samples treated with both dSMADi and XAV939 (D5_XAV939, 2919 cells analyzed, and D10_XAV939, 2638 cells analyzed) (Fig. 6A). With Seurat analysis, we identified 7 cell clusters (Fig. 6B). Surprisingly, the samples are not very heterogeneous, and Wnt inhibition could already exhibit patterning function as early as D5 (Fig. 6B and 6C). From the heatmap showing the top 5 markers of each cell cluster (Fig. 6D, Supplementary Table S3), we noticed that cluster 0 is mainly from D10_dSMADi, cluster 1 mainly from D10_XAV939, and cluster 6 from both D10_XAV939 and D10_dSMADi. Cluster 3 is mainly from D5_XAV939, cluster 4 from D5_dSMADi, and cluster 2 from both D5_XAV939 and D5_dSMADi. Cluster 5 constituted mostly undifferentiated stem cells. By performing enrichment analysis, brain development is enriched in both cluster 0 and cluster 1, cell division is enriched in cluster 2, peptide biosynthetic process is enriched in both cluster 3 and cluster 4, oxidative phosphorylation process and DNA replication are enriched in cluster 5, and neuron projection development is enriched in cluster 6 (Fig. 6E). Based on the DEGs, XAV939 samples show higher *FOXG1*(anterior neural marker), *DLK1*, and *PIK3R3* expression, while the dSMADi samples express not only higher Wnt signaling-related genes, including *WLS*, *SFRP2*, and *WNT2B* but also *OTX2* (forebrain/midbrain neural marker), *PAX6* (dorsal telencephalic progenitor marker), *LHX5*, and *EMX2* (dorsal telencephalic marker) (Supplementary Fig. S11). Meanwhile, the expression of ventral neural markers, like *HES1*, *ASCL1*, *GAD1/2*, and *DLX1/2*, are slightly increased in XAV939 samples (Supplementary Fig. S11C). Through RT-qPCR, we validated that Wnt inhibition could increase the *FOXG1* expression, and inhibit *PAX6*, *OTX2*, and *EMX2* expressions (Fig. 6F). In addition to the patterning effect on stem cells, D10_XAV939 contributes a bigger cell portion to neural progenitors (Cluster 6, which is marked by higher expression of *STMN2*, *NEUROG1*, and *NEUROD1*) than D10_dSMADi (Fig. 6B-6D). In addition, Wnt inhibition samples had higher expression level of marker genes of NNPs, including *DLK1* and *FOXG1* than dSMADi samples on D20 (Supplementary Fig. S12). Thus, the administration of Wnt inhibition could promote anterior ventral GABAergic neuron fate specification and neuron differentiation progress in our cell model.

## Discussion

GnRH neurons originate from the medial part of the nasal placode, vomeronasal organ (VNO), which in human fetuses contains actively proliferating (MKI67^+^/SOX2^+^) and nonproliferating progenitors (MKI67^-^/SOX2^+^).^3^ We identified 2 NP clusters in our *in vitro* model, consistent with the previous findings *in vivo*.^3^ Both NP clusters (NNPs and PNPs) expressed *SOX2*, *NEUROG1*, and *NEUROD1* which are marker genes of neuronal progenitors in rodents.^26-29^ This suggests that the NPs generated *in vitro* show similar gene expression patterns to the NPs *in vivo*. Our data also suggest that NNPs are probably post-mitotic neural progenitors, which derived from PNPs. Based on their transcriptional pattern, different cell fates have been assigned to NNPs as a consequence of the fate-priming effect that typically occurs at the cell cycle exit or shortly thereafter.^43^ In this stage, selector and terminal differentiation genes activate differentiation cassettes that are sets of genes guiding the neuron to exhibit its fate-specific function.^43^ For instance, our data show that one sub-cluster of NNPs (NNP-C2) highly expresses *GNRH1*, and the expression of *DLXs* could possibly mark GnRH^GABA^ precursors. Along the decision-making pathway, the cells will mainly differentiate into excitatory Glut neurons and to a lesser degree into inhibitory GnRH^GABA^ neurons. Parallel to our finding, mouse GnRH neurons are also characterized as GABAergic neurons,^44-46^ which suggests conservation across mammalian species. From the scRNA-seq, we also observed a small cell cluster, which specifically expresses *IRX* family genes. This cluster can be an intermediate state between PNPs and NNPs based on trajectory analysis. In line with this, *Irx* has been reported to contribute to neural patterning.^47^ NPs are supposed to have the capacity to differentiate into both neuronal and glial cell types; however, we did not observe any oligodendrocytes or astrocytes. It is probably because we aimed to generate neurons in the end, and have not exposed the NPs with gliogenic fate stimulators such as neurotrophic factors (CNTF) or proteins (BMP, EGF, and FGF2)^48,49^ during differentiation.

An important finding of this study was the identification of the Glut cluster produced by this protocol. Besides characterizing the cell properties, we observed that the Glut cluster highly expresses microtubule protein Doublecortin (*DCX*), neural adhesion molecules (eg, *NCAM1*), and molecules helping neural migration (eg, *RELN*). Meanwhile, a previous study^50^ showed that GABA could slow GnRH neuron migration by depolarization via changes in intracellular chloride concentrations. Thus, GnRH neuron migration might occur through synergistic autocrine and paracrine signaling. Since Glut neurons are generated together with GnRH^GABA^, we hypothesized that they originated from the same or adjacent regions in humans. GnRH neurons are generated at the olfactory placode,^4^ a region that also gives rise to glutamatergic olfactory sensory neurons.^51,52^ According to the published data for human olfactory neurogenesis,^38^ olfactory sensory neurons are derived from *NEUROG1* positive progenitors, and highly express *GNG8*, *TUBB*, *EMX2*, and *LHX2* in immature states, which is consistent with the properties of the Glut cluster in our study. Several studies also suggested that *Lhx2* is associated with olfactory receptor (OR) expression and is essential for OSNs identity^53,54^. However, we could not detect OR gene expression in our data (data not shown), which may be because of their immature state, as shown in human olfactory neurogenesis data,^38^ or insufficient sequencing depth. Further studies will be required to test whether Glut mimics the properties of olfactory sensory neurons in vivo.

From the integrated dataset, we identified 443 GnRH branch-associated genes (Supplementatry Fig. S8C, Table S2), including transcription factors, such as *ISL1*, *DLX1/2*, *DLX5*, *FOXG1*, *SIX3*, and *ARX*. Interestingly, they all associate with the olfactory epithelium or are involved in GnRH neuron development,^8,13,55-62^ which supports the reliability of our scRNA-seq analysis data. For example, ISL1 was found to be expressed in human fetal GnRH neurons and its expression was shown to persist during their migration to the hypothalamus.^13^ Isl1 expression was also observed in GnRH-3 neurons of terminal nerves in fish^56^ and in GnRH neurons in mice,^54^ but its ultimate function remains obscure as conditional ablation of *Isl1* alone does not significantly compromise GnRH neuron migration or GnRH expression. This suggests that there are other compensatory mechanisms for the role of Isl1 in GnRH neuron development or another population of olfactory derived GnRH neurons, which are Isl1 negative.^63^ In this study, consistently with previous investigations in mice,^58,59^ we confirmed *DLX5* expression in human GnRH neurons as well as in olfactory placode region. However, the role of Dlx5 in GnRH neuron development is not completely understood. Dlx5 seems to contribute to the activation of GnRH transcription directly in mouse GnRH neurons^59^ but also be required in building the connection of olfactory receptor neurons to the forebrain as in *Dlx5*^−/−^ mice. GnRH neurons were present but failed to migrate into forebrain region in *Dlx5*^−/−^ mice.^58^ Furthermore, Garaffo et al found that *Dlx5* could promote the expression of *miR-9* and *miR-200* class, and depletion of *miR-9* and *mi-200* class in zebrafish results in both delayed differentiation of olfactory receptor neurons and impaired development of GnRH neurons.^60^ However, they could not find direct evidence of the function of Dlx5 in the genesis of GnRH neurons. Thus, further investigation on the role of DLX5 in human GnRH neuron development is needed.

We observed that modification of our original protocol with early Wnt inhibition significantly improved GnRH neuron differentiation efficiency. The increase of *GNRH1* expression level (approximately 23-fold) and GnRH secretion at D25 (approximately 30-fold) were considerably higher than the increase of GnRH neuron yield (approximately 3-fold) in Wnt inhibition condition compared to plain dSMADi condition. Thus, we hypothesize that the GnRH neurons generated with Wnt inhibition are able to express higher levels of *GNRH1* than the ones generated with dSMADi. By investigating the underlying mechanisms, we found that Wnt inhibition during dSMADi stage promotes neurogenesis, which is in agreement with previous studies showing that Wnt signaling enhances self-renewal of neural progenitors^64^ and that inhibition of canonical Wnt signaling is required for neuronal differentiation.^65^ Additionally, Wnt-inhibited samples express higher *FOXG1* and *SIX3*, but lower *PAX6*, *OTX2*, and *EMX2*. These are all neural progenitor markers for different regional nervous system identity. In line with our findings, previous studies show that Wnt signaling specifies neural progenitor regional identity^17^ and that Wnt inhibition can induce a more ventralized gene expression profile including the augmentation of *DLX2, DLX5,* and *FOXG1* expression.^66,67^ It has been reported that DLX transcription factors are master regulators of the developing vertebrate brain, driving GABAergic neuron differentiation.^68^ We identified only one GABAergic neuron cluster in our scRNA-seq data, ie, GnRH^GABA^ neurons. However, it is not known whether there are other types of telencephalic GABAergic neurons promoted by XAV939. For this, we checked the expression of cortical interneuron markers at the end of differentiation,^69,70^ including *LHX8*, *HMGB1*, *BASP1*, *SST*, and *PVALB*, in addition to the spiny neuron markers,^71,72^ such as *PPP1R1B*, *FOXP1*, *BCL11B*, *CHRM4*, *PLXND1* (Supplementary Fig. S14). *LHX8* was increased in XAV939 condition, and when considering its potential role in GnRH neuron differentiation,^13^ the upregulation could also be caused by the higher yield of GnRH neurons. Since most of the cortical interneuron and spiny neuron markers were expressed at low levels and had no significant difference between dSMADi and XAV939 samples, it appears that there are no significant number of cortical neurons or spiny neurons generated following XAV939 condition. A further systematic study with higher resolution would be beneficial to investigate all the generated cell types with the developed protocol.

In previous studies, Wnt signaling activity has been detected in frontonasal region in transgenic mice^16^ and sFRP1 (a modulator of Wnt signaling) was detected in the nasal placode.^15^This suggests that Wnt signaling plays a role in olfactory development. Indeed, Wnt signaling has been demonstrated to be essential for promoting olfactory sensory neuronal fate choice.^73^ Thus, we suggest that during the embryonic olfactory neurogenesis, Wnt signaling could also play a role in patterning olfactory/GnRH neural fates. However, a recent study showed that *Lgr4* deficiency results in a reduced number of GnRH neurons and subsequently delayed puberty in mice, presumably through impaired Wnt/β-catenin signaling.^74^ Another recent study shows that in human hypothalamus development, oligodendrocytes could interact with hypothalamic neurons (GnRH neurons were not included) to mediate neuronal maturation by Wnt, Hippo, and integrin signaling.^75^ These studies did not reveal the direct role of Wnt signaling in GnRH neuron development and more studies are warranted to its timely actions on GnRH neuron specification. However, we observed that Wnt inhibition during dSMADi promotes the differentiation of NPs and GABAergic neural fate specification, thus leading to a higher number of GnRH neurons. This finding provides a considerable advantage for further in vitro studies, and is potentially clinically important given that endocrine disruptors such as bisphenol A are reported to inhibit Wnt/β-catenin pathway in neurons^76^ and are implicated in central precocious puberty of girls.^77^

In conclusion, our work identifies a differentiation trajectory of human GnRH neurons in vitro and shows how another major neuron type (Glut) is generated together with GnRH neurons. We found that *DLX* family genes are specifically involved in the ontogeny of GnRH neurons, and demonstrate a robust optimization to the efficiency of this protocol, based on inhibition of Wnt signaling, which not only promotes NP differentiation but also specifies anterior ventral neuron fate. These findings delineate the molecular basis of human GnRH neuron development in vitro.

## Supplementary Material

sxac069_suppl_Supplementary_Figure_S1

sxac069_suppl_Supplementary_Figure_S2

sxac069_suppl_Supplementary_Figure_S3

sxac069_suppl_Supplementary_Figure_S4

sxac069_suppl_Supplementary_Figure_S5

sxac069_suppl_Supplementary_Figure_S6

sxac069_suppl_Supplementary_Figure_S7

sxac069_suppl_Supplementary_Figure_S8

sxac069_suppl_Supplementary_Figure_S9

sxac069_suppl_Supplementary_Figure_S10

sxac069_suppl_Supplementary_Figure_S11

sxac069_suppl_Supplementary_Figure_S12

sxac069_suppl_Supplementary_Figure_S13

sxac069_suppl_Supplementary_Figure_S14

sxac069_suppl_Supplementary_Table_S1

sxac069_suppl_Supplementary_Table_S2

sxac069_suppl_Supplementary_Table_S3

sxac069_suppl_Supplementary_Table_S4

sxac069_suppl_Supplementary_Table_S5

sxac069_suppl_Supplementary_Table_S6

sxac069_suppl_Supplementary_Video_S1

## Data Availability

All data needed to evaluate the conclusions in the paper are present in the paper and the Supplementary Materials. The sequencing data have been deposited in the Gene Expression Omnibus under accession number GSE212901.
