# Supplementary figures and images for: Deciphering the Transcriptional Landscape of Human Pluripotent Stem Cell-Derived GnRH Neurons: The Role of Wnt Signaling in Patterning the Neural Fate
Source: Stem Cells. 2022 Sep 25;40(12):1107–21. doi: 10.1093/stmcls/sxac069 (PMC9806769; doi:10.1093/stmcls/sxac069)

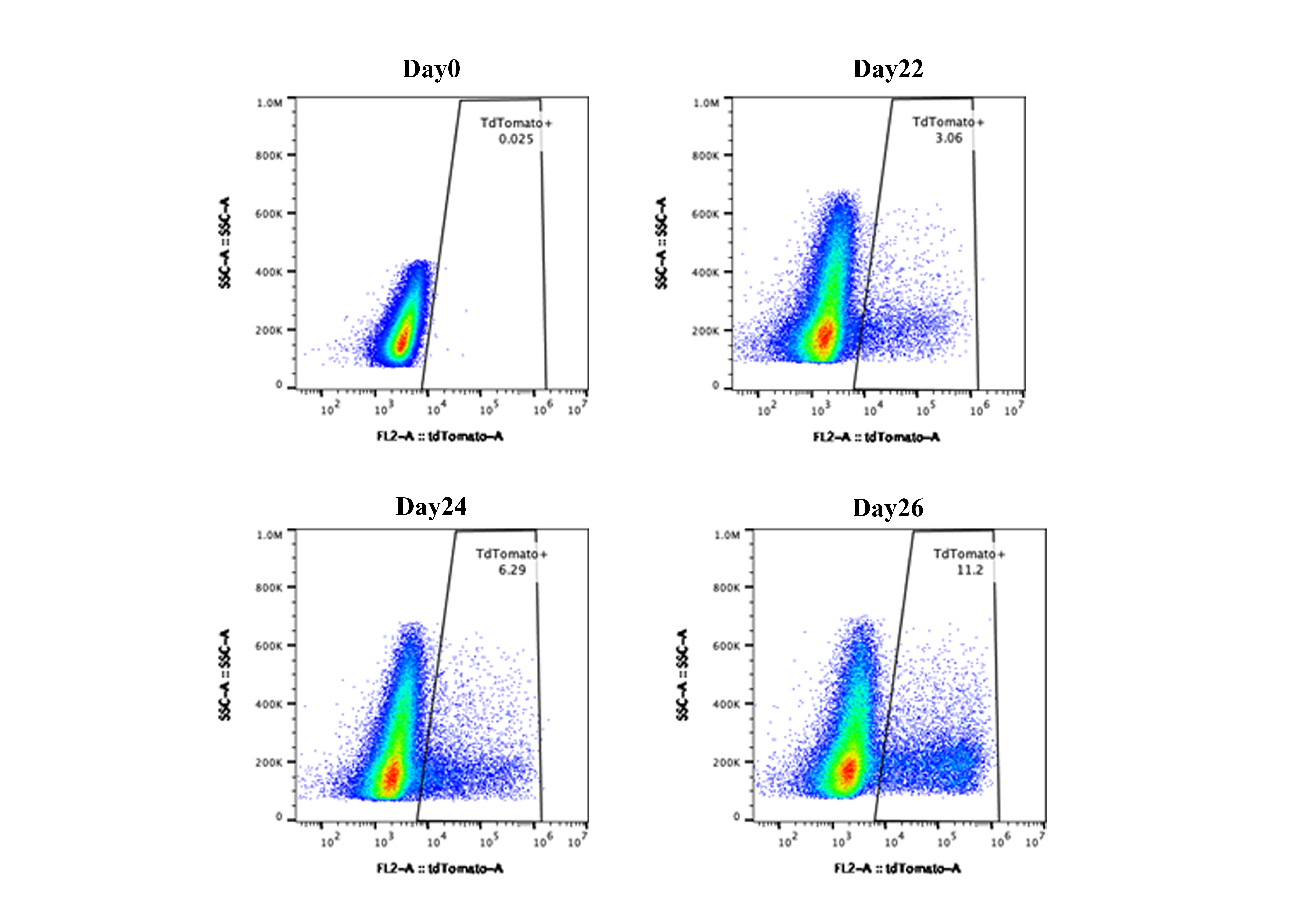

Supplement: sxac069_suppl_Supplementary_Figure_S1 [file sxac069_suppl_supplementary_figure_s1.jpeg]

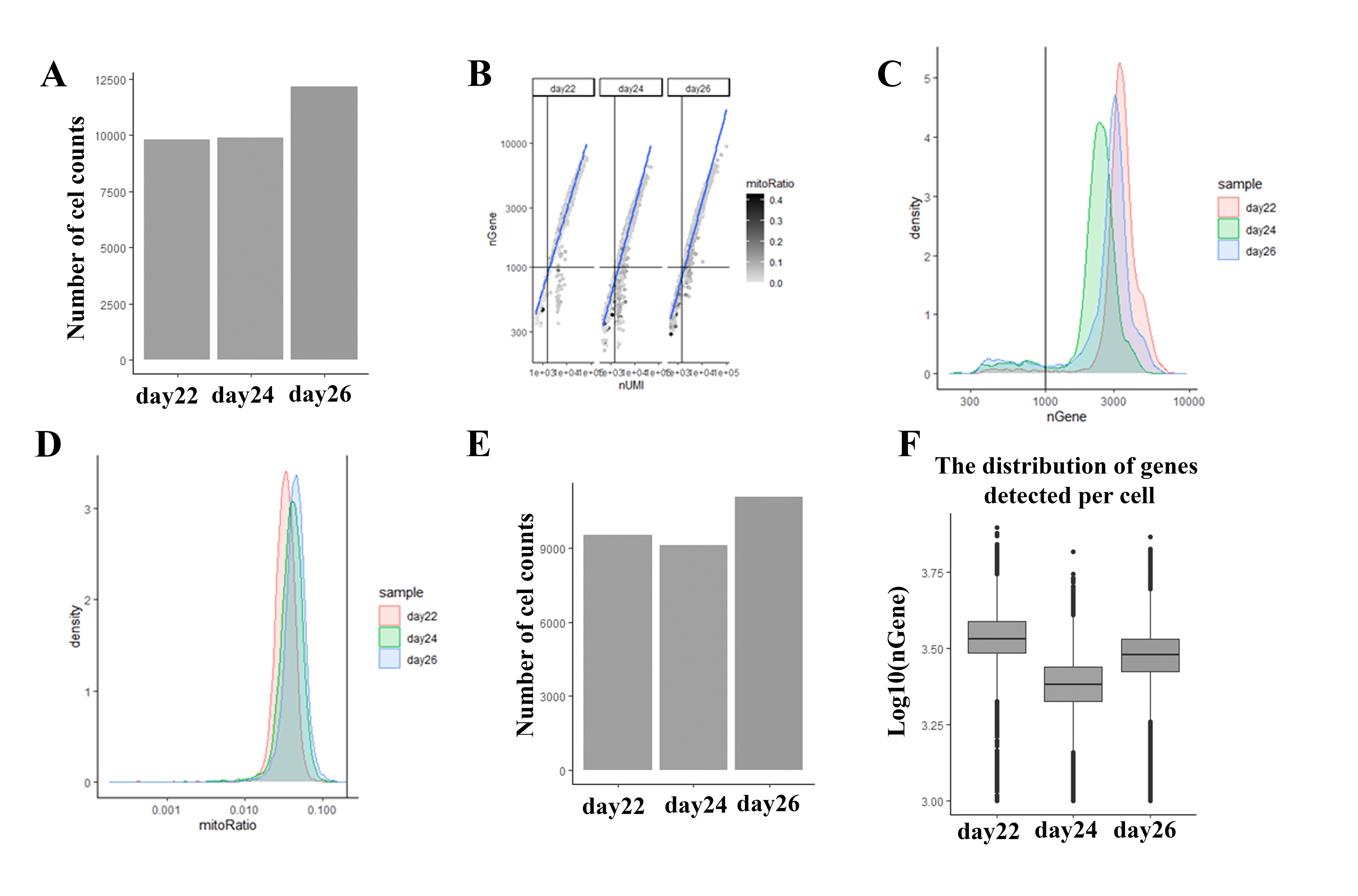

Supplement: sxac069_suppl_Supplementary_Figure_S2 [file sxac069_suppl_supplementary_figure_s2.jpeg]

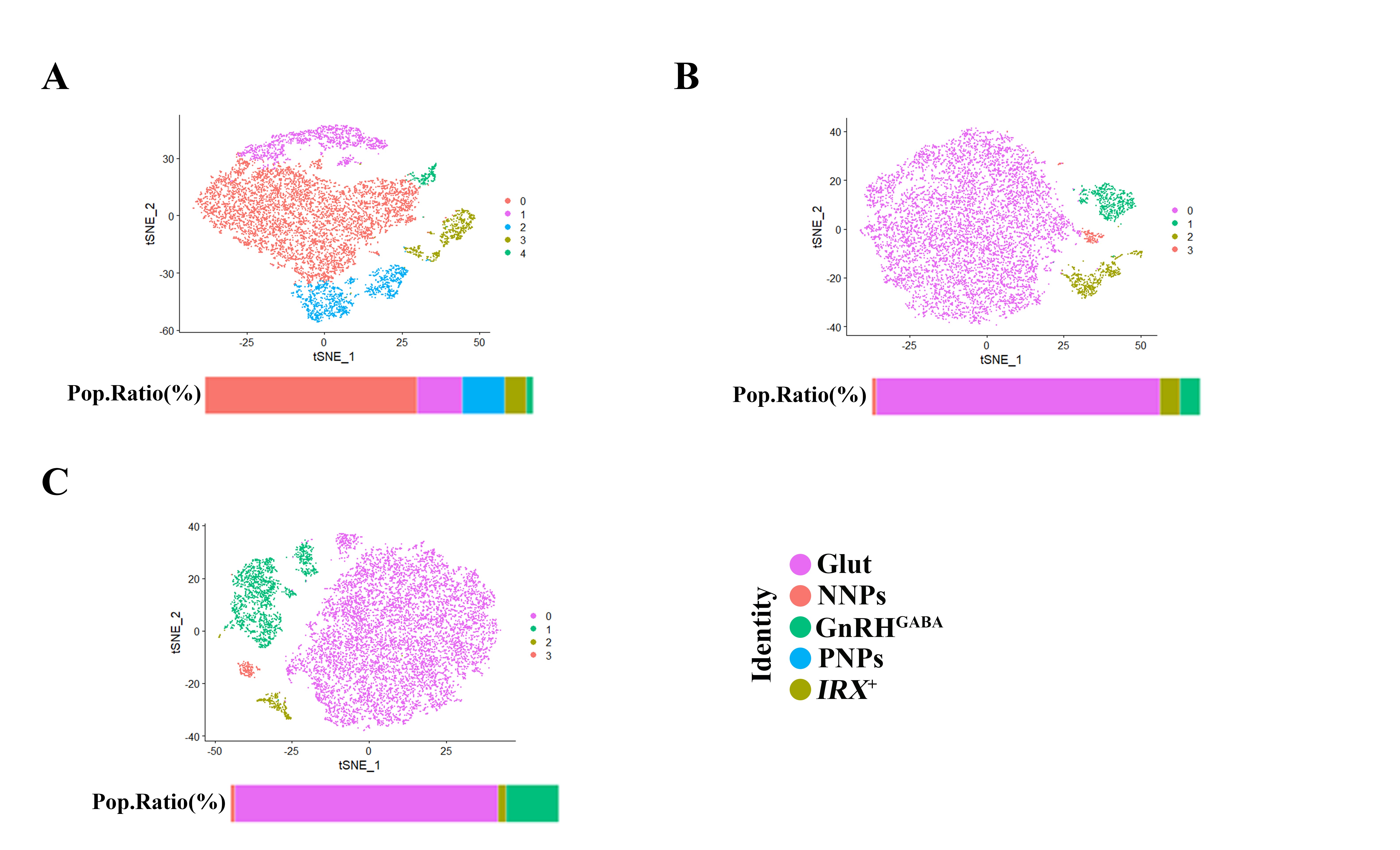

Supplement: sxac069_suppl_Supplementary_Figure_S3 [file sxac069_suppl_supplementary_figure_s3.jpeg]

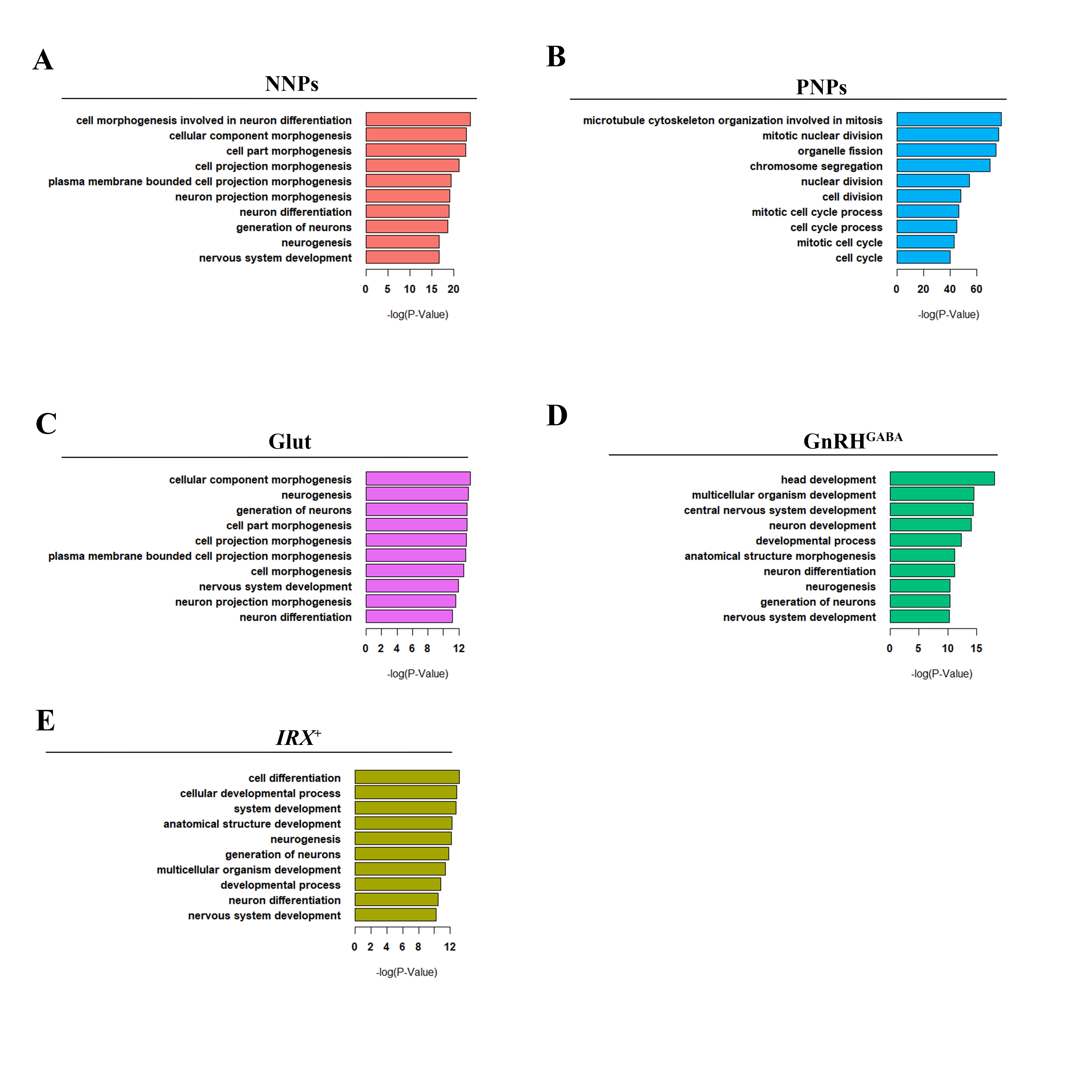

Supplement: sxac069_suppl_Supplementary_Figure_S4 [file sxac069_suppl_supplementary_figure_s4.jpeg]

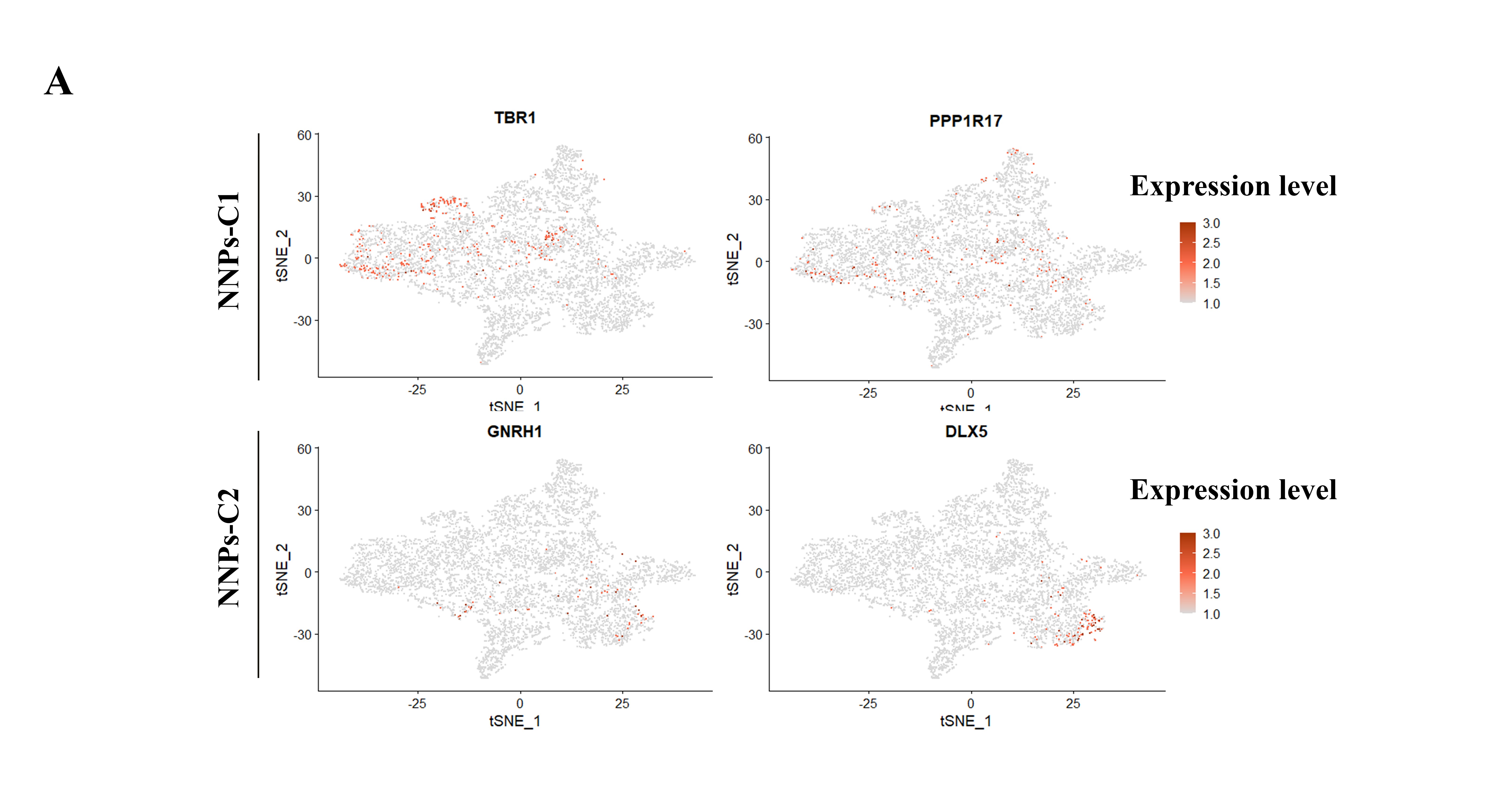

Supplement: sxac069_suppl_Supplementary_Figure_S5 [file sxac069_suppl_supplementary_figure_s5.jpeg]

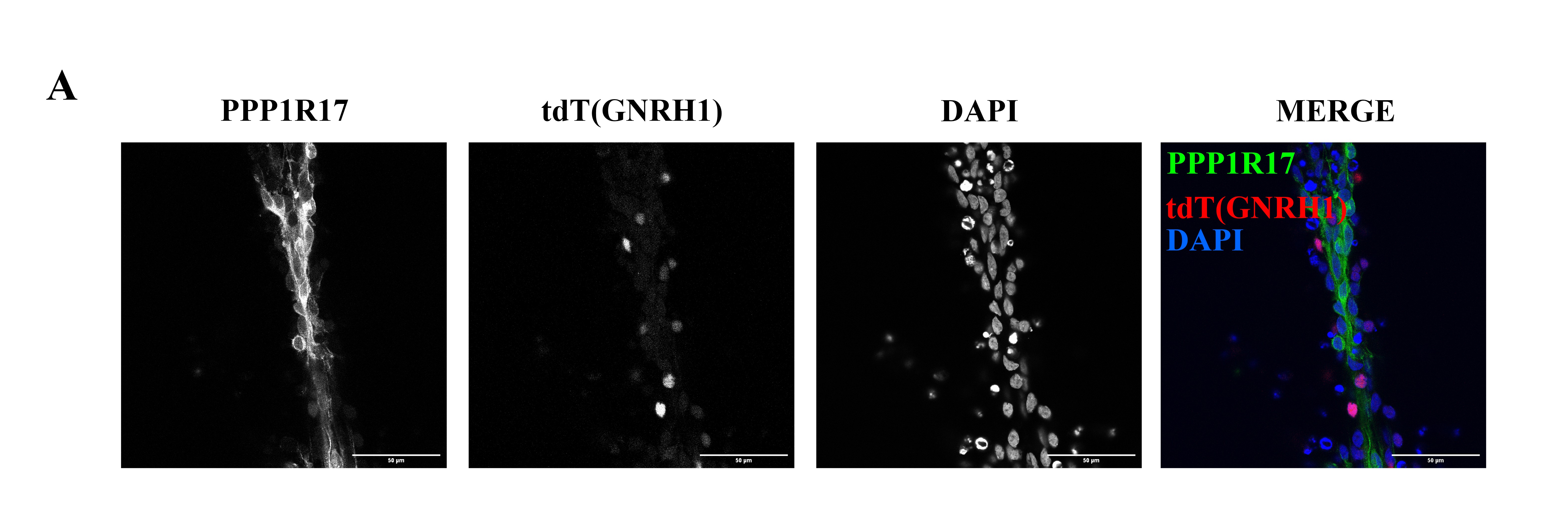

Supplement: sxac069_suppl_Supplementary_Figure_S6 [file sxac069_suppl_supplementary_figure_s6.jpeg]

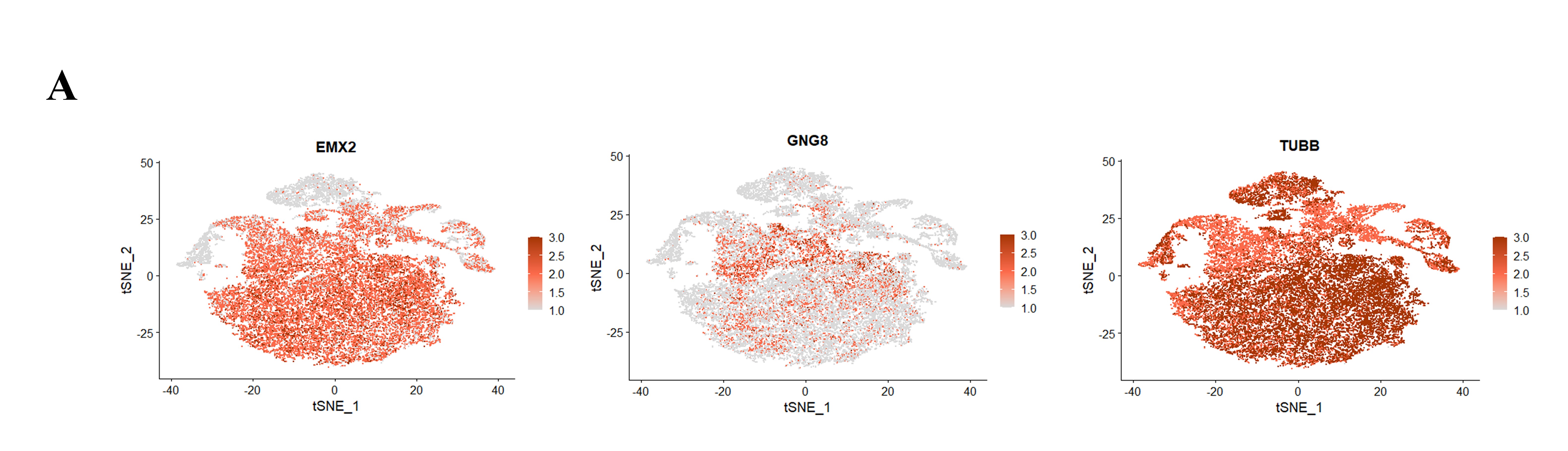

Supplement: sxac069_suppl_Supplementary_Figure_S7 [file sxac069_suppl_supplementary_figure_s7.jpeg]

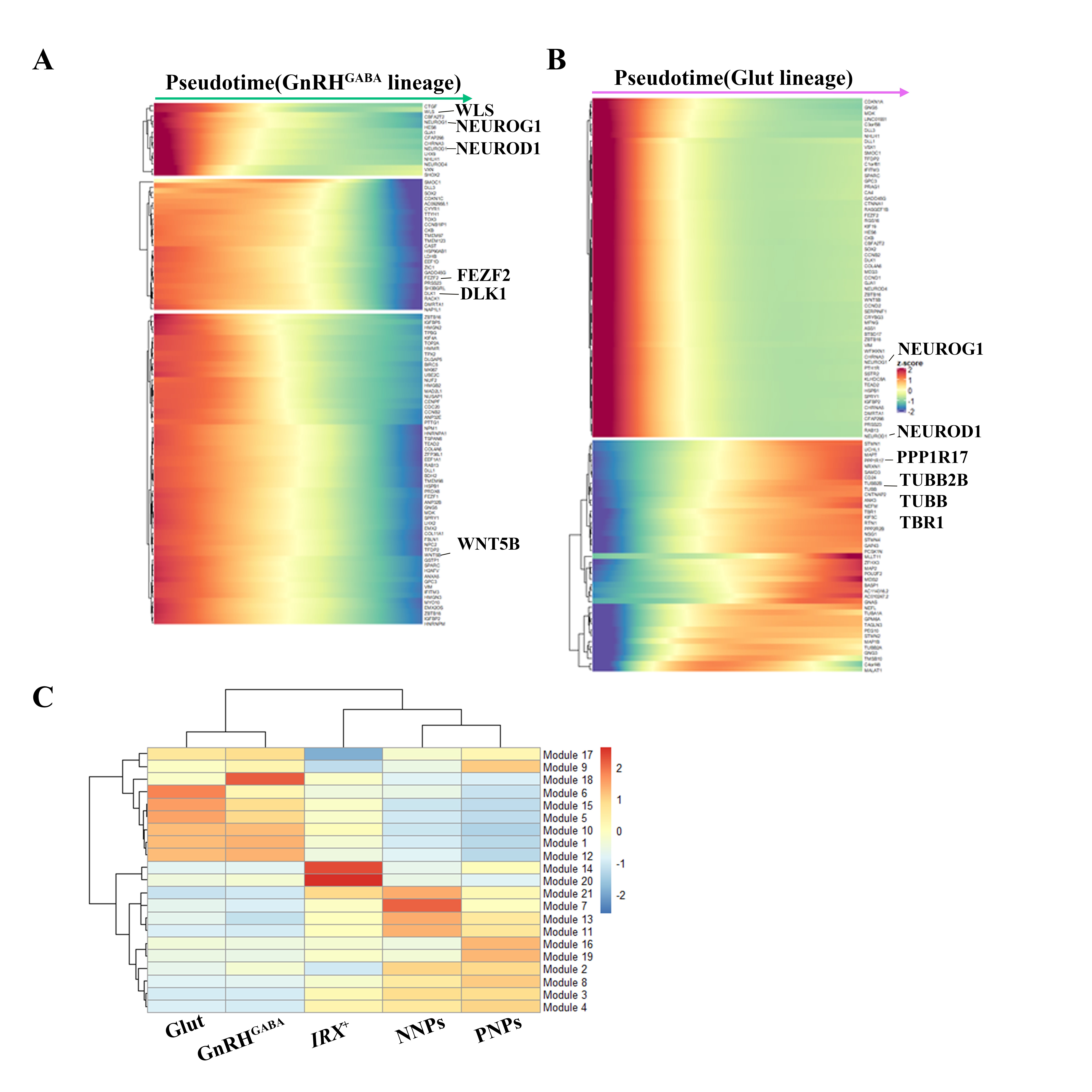

Supplement: sxac069_suppl_Supplementary_Figure_S8 [file sxac069_suppl_supplementary_figure_s8.jpeg]

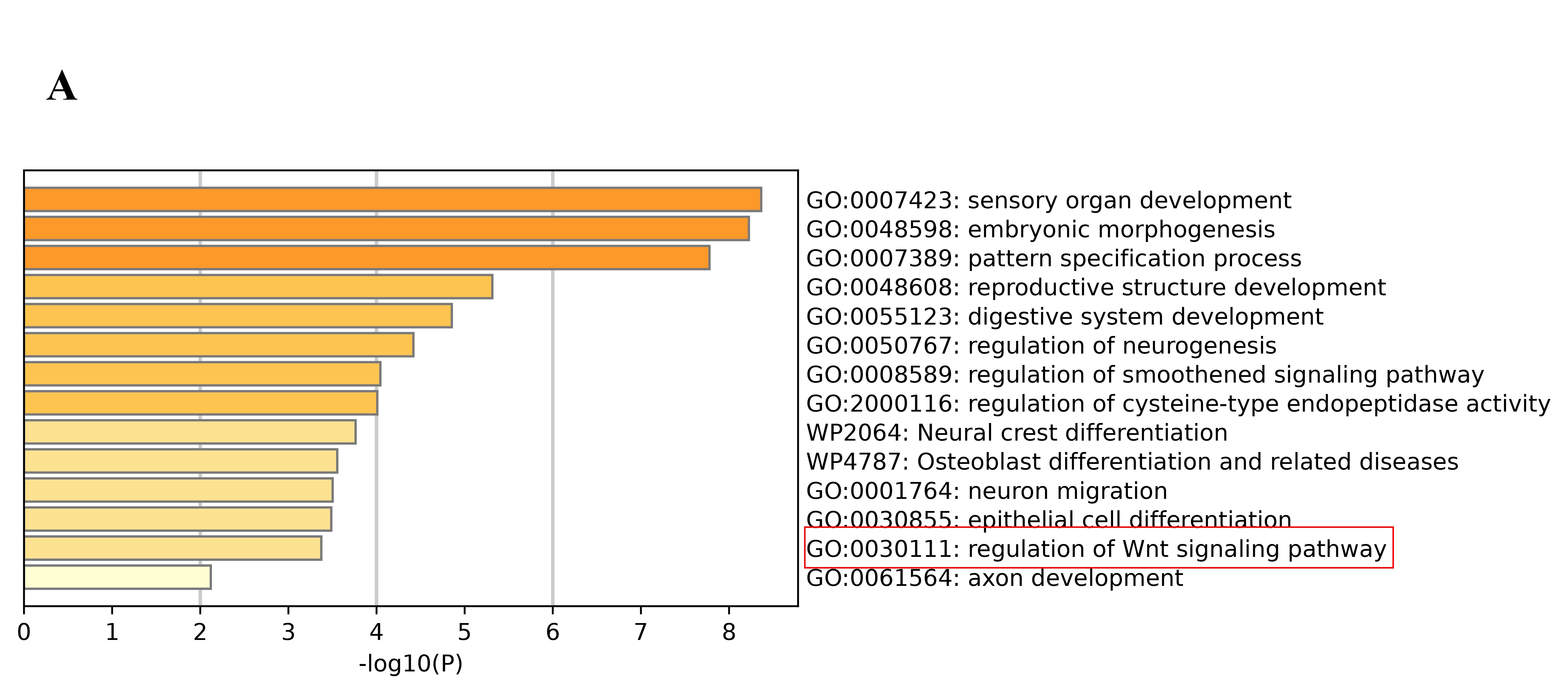

Supplement: sxac069_suppl_Supplementary_Figure_S9 [file sxac069_suppl_supplementary_figure_s9.jpeg]

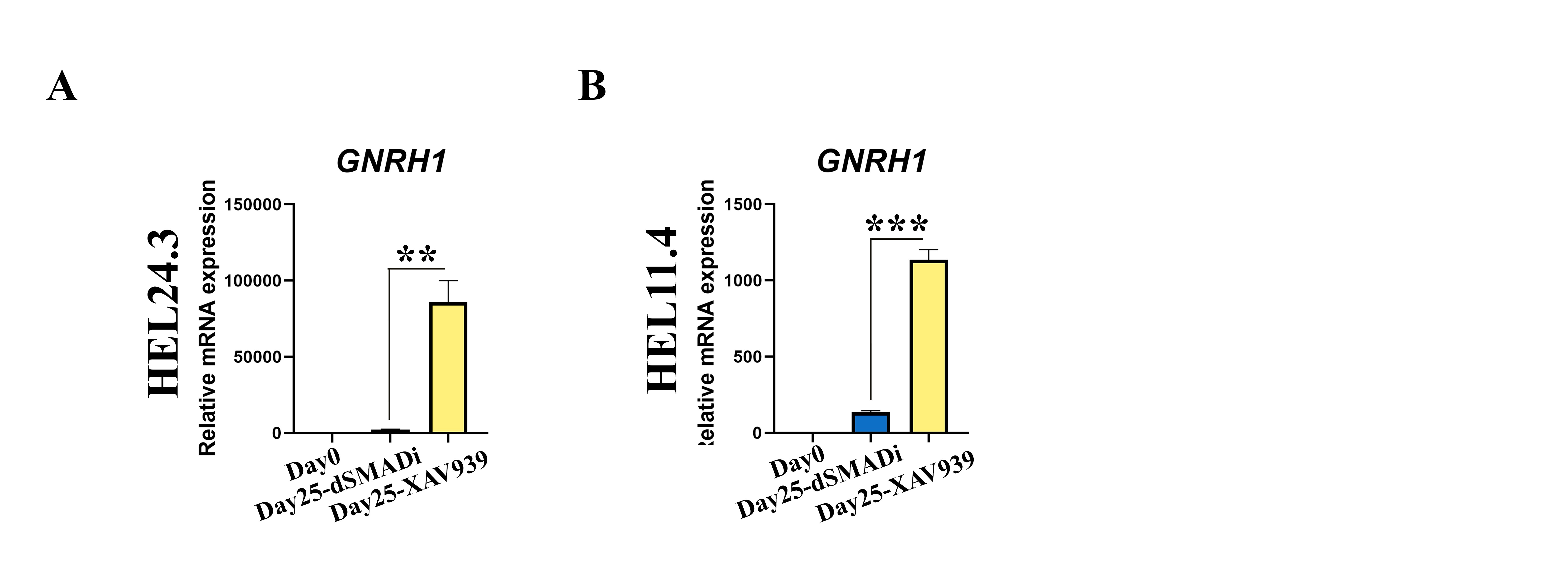

Supplement: sxac069_suppl_Supplementary_Figure_S10 [file sxac069_suppl_supplementary_figure_s10.jpeg]

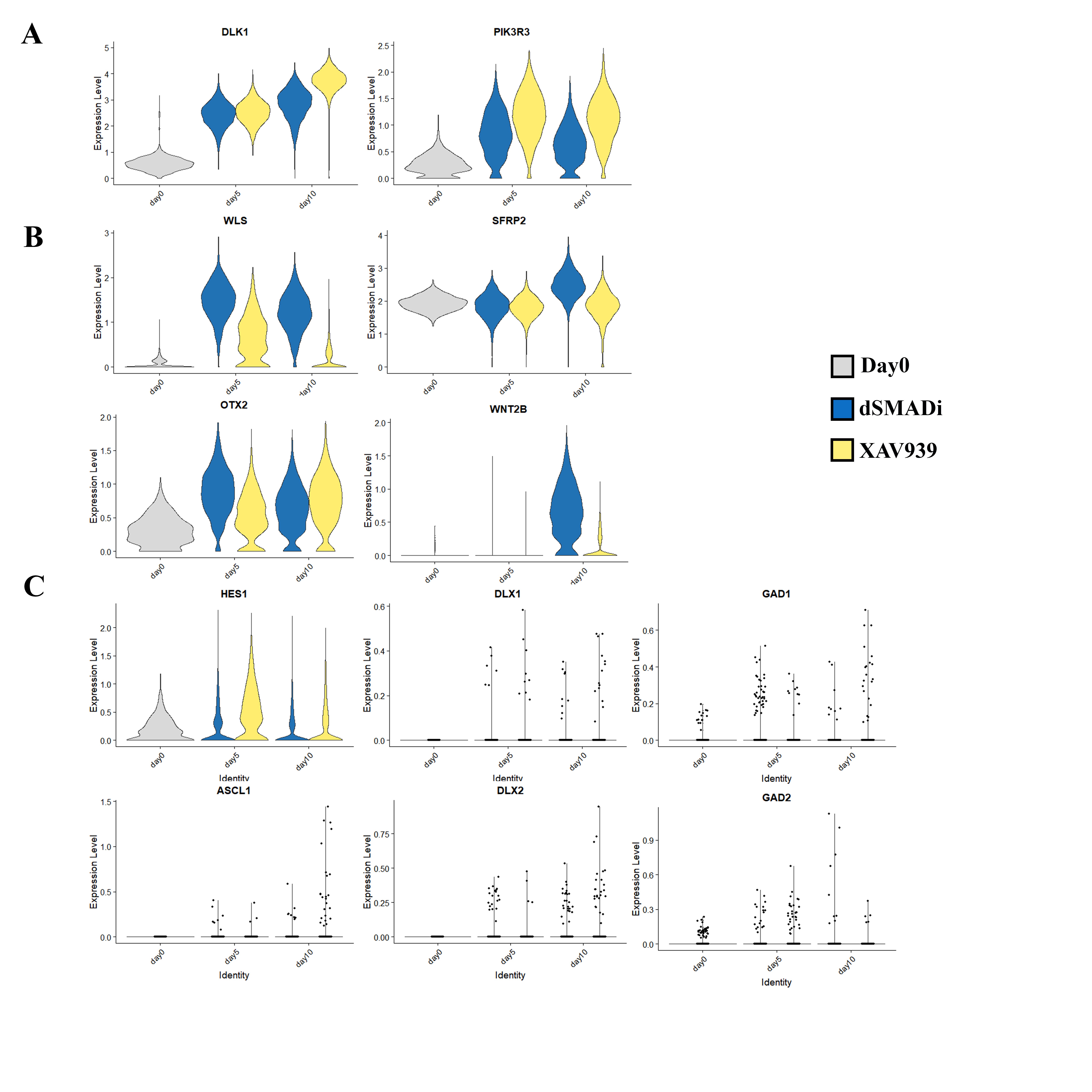

Supplement: sxac069_suppl_Supplementary_Figure_S11 [file sxac069_suppl_supplementary_figure_s11.jpeg]

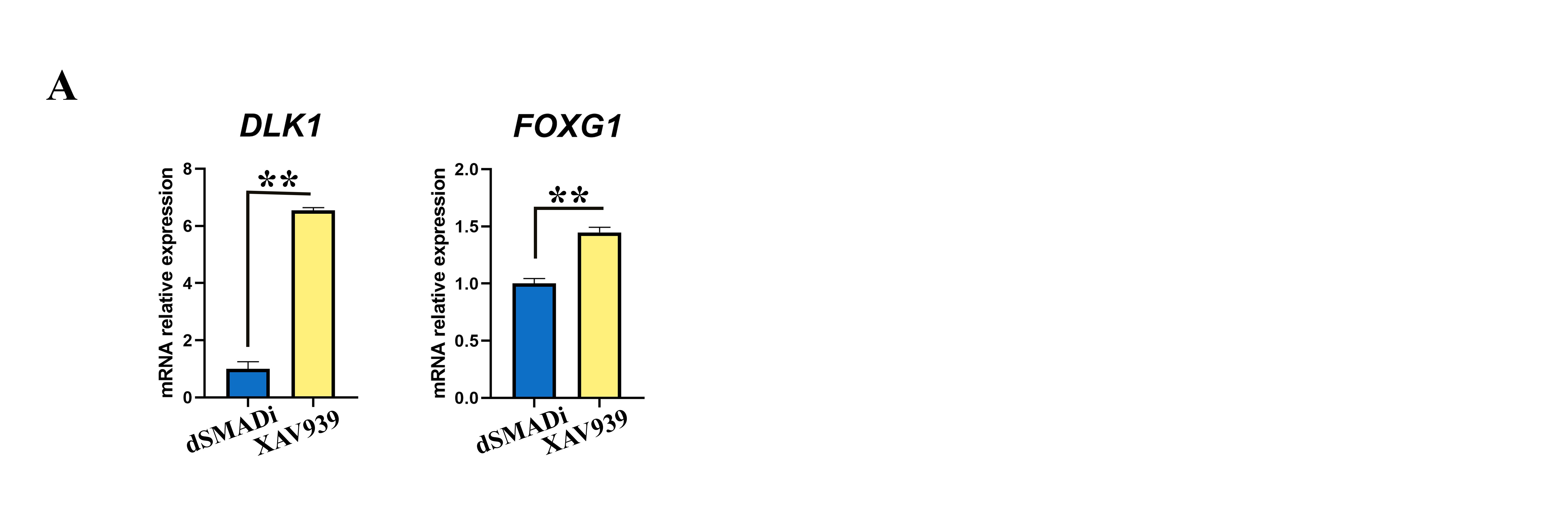

Supplement: sxac069_suppl_Supplementary_Figure_S12 [file sxac069_suppl_supplementary_figure_s12.jpeg]

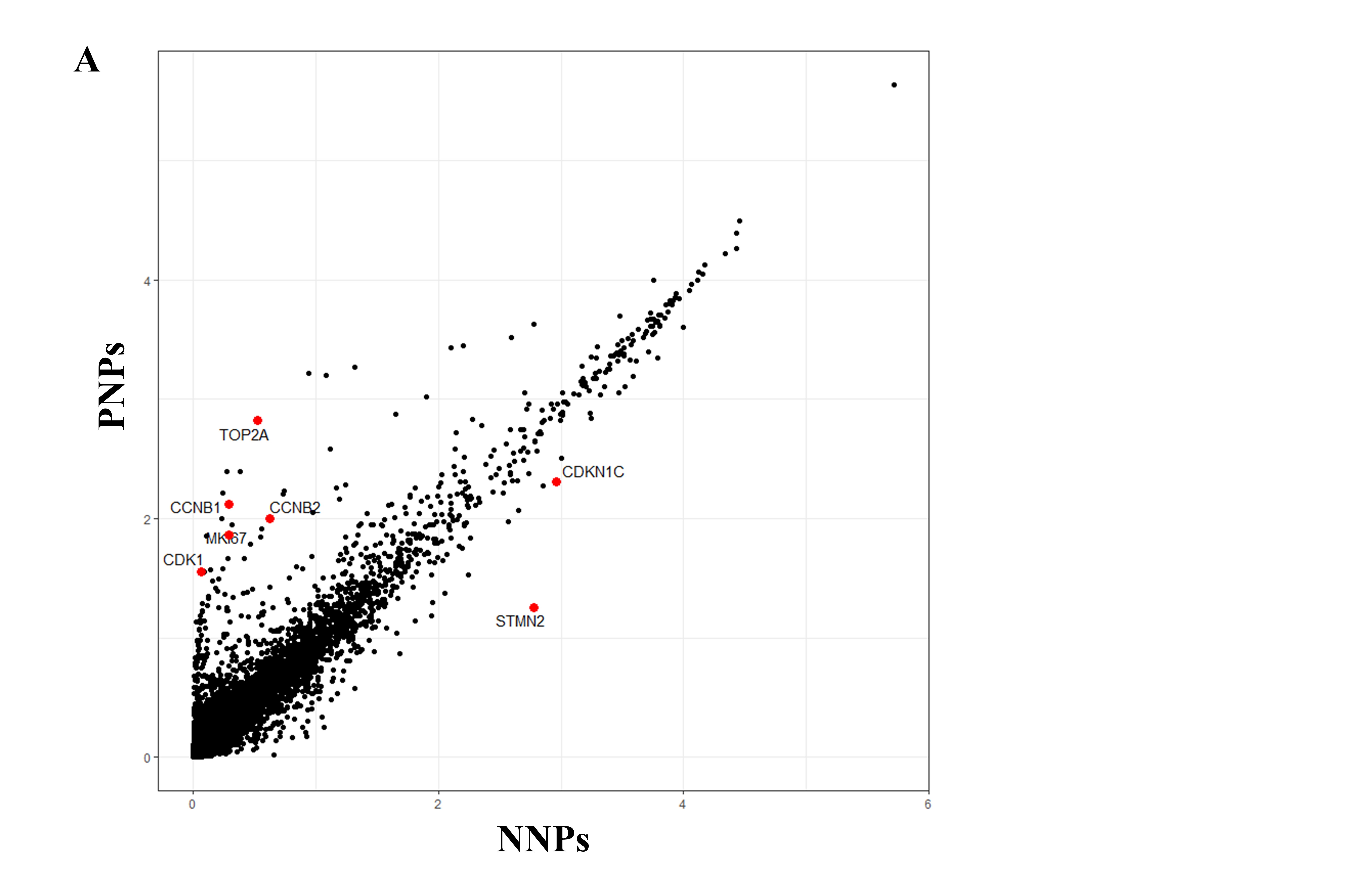

Supplement: sxac069_suppl_Supplementary_Figure_S13 [file sxac069_suppl_supplementary_figure_s13.jpeg]

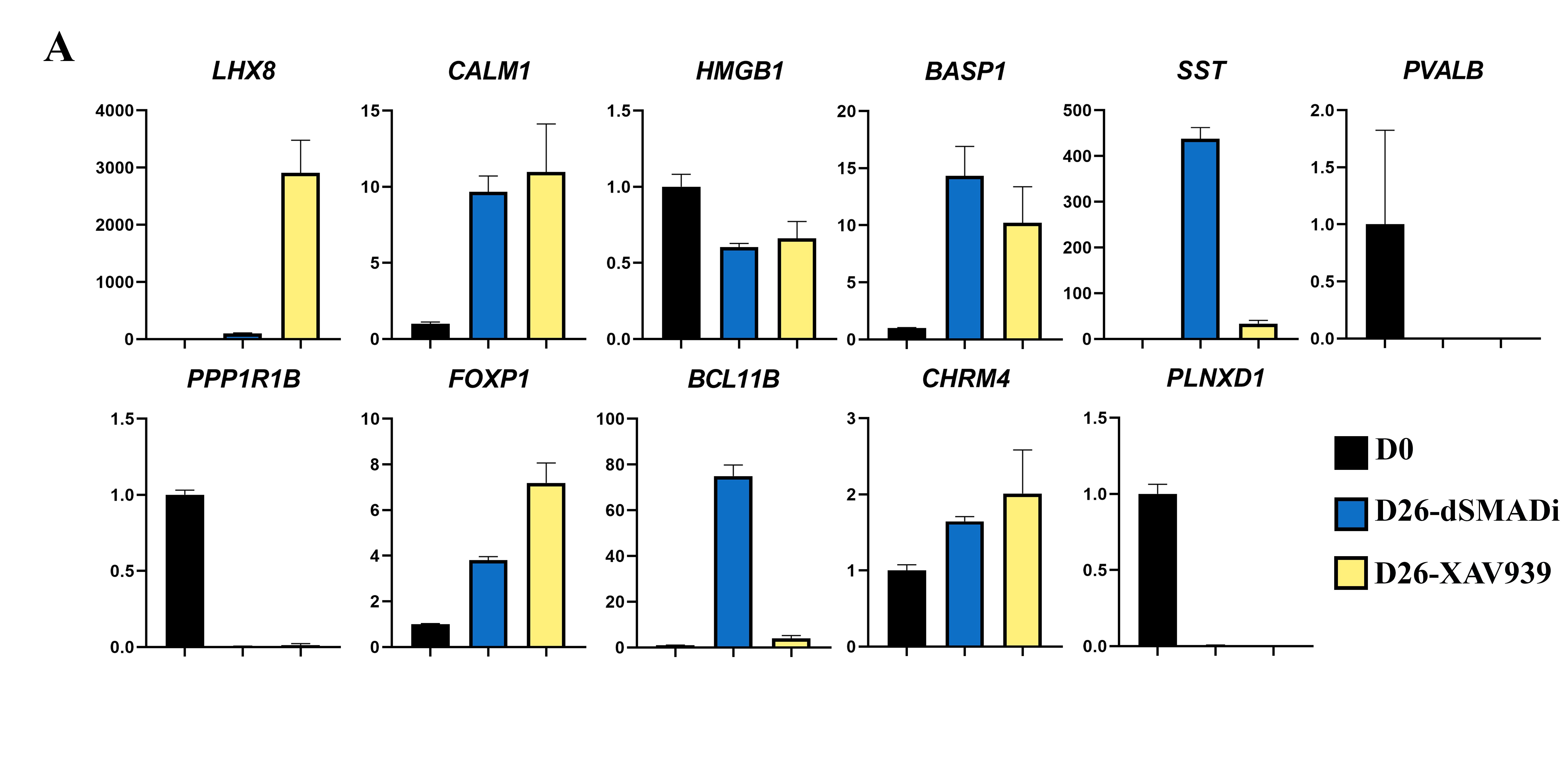

Supplement: sxac069_suppl_Supplementary_Figure_S14 [file sxac069_suppl_supplementary_figure_s14.jpeg]
