# Supplementary material for: Deciphering the Transcriptional Landscape of Human Pluripotent Stem Cell-Derived GnRH Neurons: The Role of Wnt Signaling in Patterning the Neural Fate
Source: Stem Cells. 2022 Sep 25;40(12):1107–21. doi: 10.1093/stmcls/sxac069 (PMC9806769; doi:10.1093/stmcls/sxac069)
Supplement: sxac069_suppl_Supplementary_Table_S1 [file sxac069_suppl_supplementary_table_s1.docx]

| gene | p_val | avg_logFC | pct.1 | pct.2 | p_val_adj | cluster |
| --- | --- | --- | --- | --- | --- | --- |
| PPP1R17 | 0 | 1.45647002288029 | 0.51875 | 0.092 | 0 | Glut |
| GAP43 | 0 | 1.23655652068562 | 0.686805556 | 0.3375 | 0 | Glut |
| TBR1 | 0 | 1.20872079448014 | 0.5875 | 0.098 | 0 | Glut |
| LHX5-AS1 | 0 | 1.17592046129863 | 0.667361111 | 0.380555556 | 0 | Glut |
| SAMD3 | 0 | 1.14092069235801 | 0.526388889 | 0.068 | 0 | Glut |
| RTN1 | 0 | 1.11939714905732 | 0.682638889 | 0.334722222 | 0 | Glut |
| STMN2 | 0 | 1.11170024931328 | 0.69375 | 0.534027778 | 0 | Glut |
| CNTNAP2 | 0 | 1.02905070282837 | 0.620138889 | 0.428472222 | 0 | Glut |
| POU2F2 | 0 | 1.02777397521164 | 0.6125 | 0.285416667 | 0 | Glut |
| TUBB2A | 0 | 1.01316605119149 | 0.06875 | 0.475694444 | 0 | Glut |
| GPM6A | 0 | 0.995368443115036 | 0.663194444 | 0.397916667 | 0 | Glut |
| PCSK1N | 0 | 0.979411743701137 | 0.674305556 | 0.317361111 | 0 | Glut |
| NRXN1 | 0 | 0.943289736227751 | 0.60625 | 00:34 | 0 | Glut |
| GNG3 | 0 | 0.934919395559039 | 0.672916667 | 0.369444444 | 0 | Glut |
| MGAT4C | 0 | 0.925824750330907 | 0.385416667 | 0.052 | 0 | Glut |
| GABRA2 | 0 | 0.917982471308124 | 0.338194444 | 0.041 | 0 | Glut |
| PSMD14 | 0 | 0.91611945151959 | 0.657638889 | 0.550694444 | 0 | Glut |
| KIF5C | 0 | 0.913854872336013 | 0.692361111 | 0.64375 | 0 | Glut |
| ZFHX4 | 0 | 0.847249726549369 | 0.640972222 | 0.502083333 | 0 | Glut |
| FABP7 | 0 | 0.846688800214386 | 0.571527778 | 0.411111111 | 0 | Glut |
| TUBB2B | 0 | 0.839294939196372 | 1 | 0.693055556 | 0 | Glut |
| SCG5 | 0 | 0.835571667371836 | 0.447916667 | 0.197222222 | 0 | Glut |
| LHX1 | 0 | 0.8256904311343 | 0.434027778 | 00:14 | 0 | Glut |
| STMN4 | 0 | 0.818716125203433 | 0.616666667 | 0.290277778 | 0 | Glut |
| PIK3R1 | 0 | 0.785547698990982 | 0.509722222 | 00:37 | 0 | Glut |
| ANK3 | 0 | 0.782695600850731 | 0.58125 | 0.275 | 0 | Glut |
| RSPO3 | 0 | 0.742795003473258 | 0.309027778 | 0.159027778 | 0 | Glut |
| NEFM | 0 | 0.732079732213779 | 0.654166667 | 0.344444444 | 0 | Glut |
| RELN | 0 | 0.725475868059684 | 0.409027778 | 0.099305556 | 0 | Glut |
| SCG2 | 0 | 0.718417879650218 | 0.481944444 | 0.207638889 | 0 | Glut |
| INA | 0 | 0.71503747161025 | 0.663888889 | 0.50625 | 0 | Glut |
| UBE2E3 | 0 | 0.711399083249447 | 0.689583333 | 0.679166667 | 0 | Glut |
| NEO1 | 0 | 0.700570935199495 | 0.468055556 | 0.265972222 | 0 | Glut |
| MAPT | 0 | 0.697857093796121 | 0.54375 | 0.185416667 | 0 | Glut |
| TTC9B | 0 | 0.696162428126991 | 0.54375 | 0.184027778 | 0 | Glut |
| EMX2 | 0 | 0.69495974725509 | 0.6625 | 00:07 | 0 | Glut |
| UCHL1 | 0 | 0.692450542082906 | 0.688888889 | 0.656944444 | 0 | Glut |
| FSTL5 | 0 | 0.69183189617742 | 0.257638889 | 0.048 | 0 | Glut |
| TUBA1A | 0 | 0.684110590154126 | 1 | 1 | 0 | Glut |
| BASP1 | 0 | 0.674776137226348 | 1 | 0.690972222 | 0 | Glut |
| NOVA1 | 0 | 0.657926720795023 | 0.66875 | 0.586805556 | 0 | Glut |
| C4orf48 | 0 | 0.655699272369053 | 0.692361111 | 0.686805556 | 0 | Glut |
| TMX4 | 0 | 0.65408760383238 | 0.546527778 | 0.338888889 | 0 | Glut |
| ANKS1B | 0 | 0.653973974633581 | 0.425 | 0.122916667 | 0 | Glut |
| MAP2 | 0 | 0.644662155680623 | 0.685416667 | 0.063888889 | 0 | Glut |
| NCAM1 | 0 | 0.639857946139836 | 0.553472222 | 0.304166667 | 0 | Glut |
| FXYD6 | 0 | 0.634290425773391 | 0.620138889 | 0.4625 | 0 | Glut |
| RFTN1 | 0 | 0.631818718918868 | 0.435416667 | 0.140972222 | 0 | Glut |
| CD24 | 0 | 0.630802017943642 | 0.686805556 | 0.645138889 | 0 | Glut |
| MLLT11 | 0 | 0.630639767426611 | 0.693055556 | 0.68125 | 0 | Glut |
| AR | 0 | 0.629487564454069 | 0.338888889 | 0.089583333 | 0 | Glut |
| CELF4 | 0 | 0.621194872869944 | 0.508333333 | 0.233333333 | 0 | Glut |
| PCSK2 | 0 | 0.612062491889119 | 0.449305556 | 0.190972222 | 0 | Glut |
| BEX2 | 0 | 0.61202517615087 | 0.634722222 | 0.538194444 | 0 | Glut |
| APLP1 | 0 | 0.605778214226236 | 0.634027778 | 0.480555556 | 0 | Glut |
| CXADR | 0 | 0.602557506967063 | 0.066666667 | 0.575 | 0 | Glut |
| RAB9B | 0 | 0.598219536890672 | 0.428472222 | 0.171527778 | 0 | Glut |
| PEG10 | 0 | 0.597683347273577 | 0.689583333 | 0.640972222 | 0 | Glut |
| PAK3 | 0 | 0.596931261631734 | 0.652083333 | 0.059027778 | 0 | Glut |
| PBX1 | 0 | 0.587225109525492 | 0.641666667 | 0.540972222 | 0 | Glut |
| KIF5A | 0 | 0.586368863891862 | 0.552083333 | 0.320833333 | 0 | Glut |
| NSG1 | 0 | 0.585169129340915 | 0.574305556 | 0.264583333 | 0 | Glut |
| PPP2R2B | 0 | 0.58371659181365 | 0.561805556 | 0.175694444 | 0 | Glut |
| ZIC2 | 0 | 0.583477604865072 | 0.605555556 | 0.047916667 | 0 | Glut |
| KIDINS220 | 0 | 0.583033879073917 | 0.632638889 | 0.552777778 | 0 | Glut |
| SPTBN1 | 0 | 0.581002785233312 | 0.486805556 | 0.290277778 | 0 | Glut |
| KIF21A | 0 | 0.576514744076244 | 0.61875 | 0.481944444 | 0 | Glut |
| BAALC | 0 | 0.571054975834625 | 0.408333333 | 0.219444444 | 0 | Glut |
| BEX1 | 0 | 0.57072836313652 | 0.661805556 | 0.598611111 | 0 | Glut |
| MYL6 | 0 | 0.567970406114094 | 0.691666667 | 0.691666667 | 0 | Glut |
| CNIH2 | 0 | 0.567721638976648 | 0.630555556 | 0.496527778 | 0 | Glut |
| ATP1B1 | 0 | 0.563013788829406 | 0.551388889 | 0.351388889 | 0 | Glut |
| AP1S2 | 0 | 0.561466492840885 | 0.648611111 | 0.570833333 | 0 | Glut |
| DAAM1 | 0 | 0.561396350497552 | 0.66875 | 0.611805556 | 0 | Glut |
| ZFHX4-AS1 | 0 | 0.560054224341417 | 00:52 | 00:15 | 0 | Glut |
| TERF2IP | 0 | 0.558449518756151 | 0.685416667 | 0.653472222 | 0 | Glut |
| CXXC5 | 0 | 0.557759896552469 | 0.599305556 | 0.470833333 | 0 | Glut |
| SEZ6L2 | 0 | 0.554518272249108 | 0.581944444 | 0.365972222 | 0 | Glut |
| KHDRBS3 | 0 | 0.553482392962669 | 0.522222222 | 0.288194444 | 0 | Glut |
| TMSB10 | 0 | 0.552327735508868 | 1 | 1 | 0 | Glut |
| SCN9A | 0 | 0.550598793539713 | 00:49 | 0.095 | 0 | Glut |
| KIFAP3 | 0 | 0.550480664055979 | 0.615972222 | 0.045833333 | 0 | Glut |
| PRKAR2B | 0 | 0.549842450749738 | 00:08 | 00:48 | 0 | Glut |
| UBE2E2 | 0 | 0.547535959486762 | 0.383333333 | 0.165277778 | 0 | Glut |
| ELAVL4 | 0 | 0.546315428037613 | 0.538888889 | 0.311111111 | 0 | Glut |
| TCEAL3 | 0 | 0.544608637781966 | 0.558333333 | 0.392361111 | 0 | Glut |
| NDUFB8 | 0 | 0.544347239706325 | 0.065277778 | 0.642361111 | 0 | Glut |
| NLRP1 | 0 | 0.541473360713348 | 0.663194444 | 0.59375 | 0 | Glut |
| SCG3 | 0 | 0.533843148259331 | 0.6375 | 0.438194444 | 0 | Glut |
| SLIT1 | 0 | 0.532410255364364 | 0.347916667 | 0.086111111 | 0 | Glut |
| TAGLN3 | 0 | 0.531476407853636 | 0.665277778 | 0.509722222 | 0 | Glut |
| LRRN3 | 0 | 0.529134481477545 | 0.405555556 | 0.129861111 | 0 | Glut |
| ENO2 | 0 | 0.528989060924883 | 0.474305556 | 0.220833333 | 0 | Glut |
| DCX | 0 | 0.525445676118417 | 0.683333333 | 0.629861111 | 0 | Glut |
| RUFY3 | 0 | 0.521762100889416 | 0.605555556 | 0.486805556 | 0 | Glut |
| SOBP | 0 | 0.521023888863318 | 0.424305556 | 0.200694444 | 0 | Glut |
| JUN | 0 | 0.517961644542959 | 0.3875 | 0.224305556 | 0 | Glut |
| MAP1B | 0 | 0.516647003784595 | 1 | 0.690972222 | 0 | Glut |
| CCDC28B | 0 | 0.513681124906436 | 0.499305556 | 0.279166667 | 0 | Glut |

| PAM | 0 | 0.513181146748453 | 0.410416667 | 0.195833333 | 0 | Glut |
| --- | --- | --- | --- | --- | --- | --- |
| MRPS6 | 0 | 0.509681898557299 | 0.59375 | 0.515277778 | 0 | Glut |
| TCEAL7 | 0 | 0.509224891371998 | 0.440277778 | 0.355555556 | 0 | Glut |
| ASAH1 | 0 | 0.502971639254169 | 0.439583333 | 0.247222222 | 0 | Glut |
| DACH2 | 0 | 0.502528336850643 | 00:46 | 0.109722222 | 0 | Glut |
| NSG2 | 0 | 0.502168423572546 | 0.400694444 | 0.140972222 | 0 | Glut |
| PHACTR3 | 0 | 0.500392369090521 | 0.338194444 | 0.104861111 | 0 | Glut |
| DSTN | 0 | 0.499737569882859 | 0.667361111 | 0.615277778 | 0 | Glut |
| DCLK1 | 0 | 0.499003262714503 | 0.406944444 | 0.211111111 | 0 | Glut |
| YWHAH | 0 | 0.498585827153484 | 0.657638889 | 0.598611111 | 0 | Glut |
| GDAP1L1 | 0 | 0.49818221609108 | 0.525 | 00:42 | 0 | Glut |
| TMEM14A | 0 | 0.495595940804881 | 0.413888889 | 00:27 | 0 | Glut |
| DCC | 0 | 0.494917066645407 | 0.56875 | 0.408333333 | 0 | Glut |
| NDN | 0 | 0.492029231818884 | 0.619444444 | 0.543055556 | 0 | Glut |
| RERE | 0 | 0.491579149558529 | 0.063194444 | 0.58125 | 0 | Glut |
| CADM1 | 0 | 0.491267564663705 | 0.49375 | 0.313194444 | 0 | Glut |
| PKIA | 0 | 0.489793833017076 | 0.633333333 | 0.544444444 | 0 | Glut |
| ENHO | 0 | 0.48573655758803 | 0.43125 | 0.211805556 | 0 | Glut |
| DPYSL2 | 0 | 0.485394478642211 | 0.664583333 | 0.636805556 | 0 | Glut |
| CDH18 | 0 | 0.485314589103371 | 0.253472222 | 0.035 | 0 | Glut |
| SLC22A17 | 0 | 0.484146036384865 | 0.404166667 | 0.195833333 | 0 | Glut |
| JUND | 0 | 0.48189838696647 | 0.688194444 | 0.684722222 | 0 | Glut |
| FAM229B | 0 | 0.481157308276428 | 0.564583333 | 0.465972222 | 0 | Glut |
| TUBB | 0 | 0.479991651732294 | 0.69375 | 1 | 0 | Glut |
| ISLR2 | 0 | 0.478081493112449 | 0.429166667 | 00:03 | 0 | Glut |
| CCDC112 | 0 | 0.477670127207098 | 0.605555556 | 0.508333333 | 0 | Glut |
| SARAF | 0 | 0.477518618068365 | 0.679861111 | 0.664583333 | 0 | Glut |
| RUNX1T1 | 0 | 0.475391586187038 | 0.438194444 | 0.26875 | 0 | Glut |
| SYT5 | 0 | 0.474763223258753 | 0.374305556 | 0.135416667 | 0 | Glut |
| VAT1 | 0 | 0.471284893133832 | 0.5125 | 0.355555556 | 0 | Glut |
| KLHL4 | 0 | 0.471208106987225 | 0.260416667 | 0.043 | 0 | Glut |
| CELF5 | 0 | 0.470363629852939 | 0.527083333 | 0.338194444 | 0 | Glut |
| SV2A | 0 | 0.46820551100282 | 0.409027778 | 0.184722222 | 0 | Glut |
| AC004540.2 | 0 | 0.466141066584493 | 0.560416667 | 0.460416667 | 0 | Glut |
| TUSC3 | 0 | 0.464989001374149 | 0.560416667 | 0.46875 | 0 | Glut |
| CALM1 | 0 | 0.464354731890073 | 0.688888889 | 0.672222222 | 0 | Glut |
| CDH12 | 0 | 0.462359812877459 | 0.229861111 | 0.041 | 0 | Glut |
| CHGB | 0 | 0.457111193050035 | 0.415277778 | 0.216666667 | 0 | Glut |
| SH3BP5 | 0 | 0.456971714564505 | 0.354861111 | 0.125694444 | 0 | Glut |
| CHL1 | 0 | 0.456593680197799 | 0.271527778 | 0.075 | 0 | Glut |
| OPTN | 0 | 0.456321902010629 | 0.422222222 | 0.179861111 | 0 | Glut |
| NRN1 | 0 | 0.4562341772236 | 0.325694444 | 0.135416667 | 0 | Glut |
| FJX1 | 0 | 0.455036810719825 | 0.420138889 | 00:38 | 0 | Glut |
| PTPRE | 0 | 0.453463363753547 | 0.238888889 | 0.035 | 0 | Glut |
| CEP170 | 0 | 0.451354469901355 | 0.638194444 | 0.601388889 | 0 | Glut |
| SLC17A6 | 0 | 0.450197831739867 | 0.265972222 | 0.054 | 0 | Glut |
| BRINP1 | 0 | 0.447515477943927 | 0.240277778 | 0.053 | 0 | Glut |
| CCNI | 0 | 0.447497514872324 | 0.69375 | 0.69375 | 0 | Glut |
| EIF4A2 | 0 | 0.445149039159543 | 0.605555556 | 0.574305556 | 0 | Glut |
| ACAT2 | 0 | 0.444661816243136 | 0.600694444 | 0.525694444 | 0 | Glut |
| PTPRO | 0 | 0.444436347490227 | 0.3 | 0.095138889 | 0 | Glut |
| ARL6IP5 | 0 | 0.443626249987153 | 0.596527778 | 0.509027778 | 0 | Glut |
| MALAT1 | 0 | 0.442809476581958 | 0.692361111 | 0.691666667 | 0 | Glut |
| MEAF6 | 0 | 0.441086269454608 | 0.640972222 | 0.579861111 | 0 | Glut |
| RIMS2 | 0 | 0.437523700591342 | 00:48 | 0.1375 | 0 | Glut |
| TSPYL4 | 0 | 0.437302915309655 | 0.606944444 | 0.55 | 0 | Glut |
| TTC3 | 0 | 0.434075084719742 | 0.690277778 | 0.686111111 | 0 | Glut |
| ACTL6B | 0 | 0.43328308325698 | 0.377777778 | 0.134722222 | 0 | Glut |
| ARPC5 | 0 | 0.431692938542312 | 0.650694444 | 0.619444444 | 0 | Glut |
| LHX5 | 0 | 0.430743742191464 | 0.356944444 | 0.171527778 | 0 | Glut |
| AC010931.2 | 0 | 0.429409138054285 | 0.507638889 | 0.365972222 | 0 | Glut |
| EVL | 0 | 0.428219332053353 | 0.626388889 | 0.575694444 | 0 | Glut |
| HMGCR | 0 | 0.427512430894092 | 0.546527778 | 0.045138889 | 0 | Glut |
| RBP1 | 0 | 0.426871717609116 | 0.448611111 | 0.348611111 | 0 | Glut |
| CDC42 | 0 | 0.424026452983844 | 0.678472222 | 0.665972222 | 0 | Glut |
| MTSS1 | 0 | 0.423593056890202 | 0.41875 | 0.231944444 | 0 | Glut |
| GNAI1 | 0 | 0.423389682018522 | 0.383333333 | 0.223611111 | 0 | Glut |
| TMEM163 | 0 | 0.423126275126204 | 00:45 | 0.116666667 | 0 | Glut |
| MAGI2 | 0 | 0.421367164125428 | 0.306944444 | 0.133333333 | 0 | Glut |
| SVBP | 0 | 0.419722802099607 | 0.661805556 | 0.639583333 | 0 | Glut |
| XPR1 | 0 | 0.419132940112911 | 0.409027778 | 00:04 | 0 | Glut |
| SORBS2 | 0 | 0.417199600542427 | 0.346527778 | 0.148611111 | 0 | Glut |
| STMN1 | 0 | 0.41635487364197 | 1 | 1 | 0 | Glut |
| NAP1L3 | 0 | 0.41601491661101 | 0.427777778 | 0.26875 | 0 | Glut |
| VAMP2 | 0 | 0.414063094074905 | 0.595833333 | 0.052777778 | 0 | Glut |
| SPTAN1 | 0 | 0.413582709029975 | 0.489583333 | 0.35625 | 0 | Glut |
| ZC2HC1A | 0 | 0.41251027937808 | 0.525694444 | 0.397222222 | 0 | Glut |
| BMP3 | 0 | 0.412453538179529 | 0.190972222 | 0.023 | 0 | Glut |
| SCN3A | 0 | 0.411373139295006 | 0.303472222 | 0.095138889 | 0 | Glut |
| MT-ND4 | 0 | 0.410992344273566 | 0.69375 | 0.690972222 | 0 | Glut |
| PCMT1 | 0 | 0.408911317765151 | 0.598611111 | 0.5375 | 0 | Glut |
| CDKN2D | 0 | 0.408245471056225 | 0.489583333 | 0.281944444 | 0 | Glut |
| FGF13 | 0 | 0.407097568575051 | 0.455555556 | 0.316666667 | 0 | Glut |
| DLG2 | 0 | 0.406152488879432 | 0.240277778 | 0.086 | 0 | Glut |
| RCAN2 | 0 | 0.405684506306591 | 0.311111111 | 0.134027778 | 0 | Glut |
| ROBO1 | 0 | 0.404797611738393 | 0.344444444 | 0.215972222 | 0 | Glut |
| ADCY2 | 0 | 0.403640950190026 | 0.234722222 | 0.049 | 0 | Glut |
| ATP6V0E2 | 0 | 0.403190128119277 | 0.479861111 | 0.315277778 | 0 | Glut |
| LHX2 | 0 | 0.402452434126877 | 0.551388889 | 0.456944444 | 0 | Glut |
| MT-ND2 | 0 | 0.398768773247324 | 0.693055556 | 0.688888889 | 0 | Glut |
| SMCO4 | 0 | 0.398369596134832 | 0.267361111 | 0.100694444 | 0 | Glut |
| SMIM18 | 0 | 0.398054756399834 | 0.349305556 | 0.132638889 | 0 | Glut |
| ZBTB20 | 0 | 0.397766119691087 | 0.408333333 | 0.290972222 | 0 | Glut |
| ATP1A3 | 0 | 0.397069936552553 | 0.398611111 | 0.188194444 | 0 | Glut |
| FEZ1 | 0 | 0.396744155971672 | 0.572916667 | 0.400694444 | 0 | Glut |
| SYT1 | 0 | 0.396623844154617 | 0.664583333 | 0.558333333 | 0 | Glut |
| FAM57B | 0 | 0.396367474414535 | 0.523611111 | 0.375694444 | 0 | Glut |
| KIF3A | 0 | 0.395343308646872 | 0.545833333 | 0.447222222 | 0 | Glut |
| FNBP1L | 0 | 0.394508056428565 | 0.656944444 | 0.063888889 | 0 | Glut |
| HMGCS1 | 0 | 0.39437529504988 | 0.057638889 | 0.050694444 | 0 | Glut |

| MSMO1 | 0 | 0.391712008310327 | 0.522916667 | 0.438194444 | 0 | Glut |
| --- | --- | --- | --- | --- | --- | --- |
| RORB | 0 | 0.390410198662953 | 0.3625 | 0.254861111 | 0 | Glut |
| FAM171B | 0 | 0.389272169265405 | 0.315277778 | 00:17 | 0 | Glut |
| AKAP6 | 0 | 0.387262974110231 | 0.304166667 | 0.093055556 | 0 | Glut |
| FAM13C | 0 | 0.384683477076193 | 0.342361111 | 0.160416667 | 0 | Glut |
| ONECUT2 | 0 | 0.383859834738593 | 0.353472222 | 0.225 | 0 | Glut |
| TMOD2 | 0 | 0.381530892853728 | 00:52 | 0.182638889 | 0 | Glut |
| NDRG4 | 0 | 0.381230834097902 | 0.343055556 | 0.15 | 0 | Glut |
| PJA2 | 0 | 0.380132728614703 | 0.570138889 | 0.507638889 | 0 | Glut |
| TULP4 | 0 | 0.379247673715792 | 0.483333333 | 0.393055556 | 0 | Glut |
| CAMK2N2 | 0 | 0.377885418182443 | 0.318055556 | 0.134027778 | 0 | Glut |
| ETV1 | 0 | 0.377091564112104 | 0.250694444 | 0.109722222 | 0 | Glut |
| CLASP2 | 0 | 0.37635154354024 | 0.383333333 | 0.233333333 | 0 | Glut |
| TNIK | 0 | 0.376344182071646 | 0.282638889 | 0.089583333 | 0 | Glut |
| TSHZ2 | 0 | 0.375857867007081 | 0.28125 | 0.115277778 | 0 | Glut |
| JPT1 | 0 | 0.375485029559447 | 0.691666667 | 0.690277778 | 0 | Glut |
| MAPRE3 | 0 | 0.375049948718083 | 0.395138889 | 0.2375 | 0 | Glut |
| AASDHPPT | 0 | 0.374471036133062 | 0.601388889 | 0.570833333 | 0 | Glut |
| NLGN4X | 0 | 0.37251729864317 | 0.279166667 | 0.14375 | 0 | Glut |
| SLC8A1 | 0 | 0.371470316606235 | 0.321527778 | 0.117361111 | 0 | Glut |
| YPEL3 | 0 | 0.371076995621559 | 0.388194444 | 00:36 | 0 | Glut |
| BEX4 | 0 | 0.370865832388134 | 0.650694444 | 0.63125 | 0 | Glut |
| RAB3A | 0 | 0.370803189022696 | 0.359722222 | 0.145138889 | 0 | Glut |
| CRMP1 | 0 | 0.369910847343527 | 0.657638889 | 0.626388889 | 0 | Glut |
| C16orf45 | 0 | 0.369753186698107 | 0.421527778 | 0.290277778 | 0 | Glut |
| CASTOR3 | 0 | 0.368363391677024 | 0.397222222 | 0.279166667 | 0 | Glut |
| ATCAY | 0 | 0.367525177935093 | 0.420833333 | 0.246527778 | 0 | Glut |
| LRRC4C | 0 | 0.366389823613416 | 0.277083333 | 0.103472222 | 0 | Glut |
| CNR1 | 0 | 0.366214732341486 | 0.271527778 | 0.102777778 | 0 | Glut |
| TP53BP1 | 0 | 0.364835980770393 | 0.559027778 | 0.515277778 | 0 | Glut |
| ACTB | 0 | 0.364167054569072 | 1 | 1 | 0 | Glut |
| SYP | 0 | 0.363967531666249 | 0.363888889 | 0.246527778 | 0 | Glut |
| TUT4 | 0 | 0.363844925213759 | 0.063194444 | 0.613194444 | 0 | Glut |
| ASXL3 | 0 | 0.363712707728181 | 0.3625 | 0.210416667 | 0 | Glut |
| WSB2 | 0 | 0.363315301005233 | 0.415972222 | 0.286111111 | 0 | Glut |
| YWHAG | 0 | 0.362684570678066 | 0.05625 | 0.514583333 | 0 | Glut |
| MPHOSPH8 | 0 | 0.361287102216731 | 0.527083333 | 0.452083333 | 0 | Glut |
| CMIP | 0 | 0.360672667839384 | 0.445138889 | 0.346527778 | 0 | Glut |
| CCDC184 | 0 | 0.36055213335769 | 0.324305556 | 0.140277778 | 0 | Glut |
| SLC5A3 | 0 | 0.358078325622727 | 0.302777778 | 0.16875 | 0 | Glut |
| TNRC6B | 0 | 0.358023036613101 | 0.579166667 | 0.538888889 | 0 | Glut |
| NSD3 | 0 | 0.357703200061394 | 0.63125 | 0.613194444 | 0 | Glut |
| THRA | 0 | 0.356428688384231 | 0.377777778 | 0.220138889 | 0 | Glut |
| LBH | 0 | 0.356281505591059 | 0.508333333 | 0.395138889 | 0 | Glut |
| COX20 | 0 | 0.355628403281642 | 0.059027778 | 0.560416667 | 0 | Glut |
| HDGFL3 | 0 | 0.355392572972943 | 0.616666667 | 0.584722222 | 0 | Glut |
| KIF1A | 0 | 0.354488146796373 | 0.592361111 | 0.515277778 | 0 | Glut |
| SRGAP2B | 0 | 0.354213796962579 | 0.28125 | 0.13125 | 0 | Glut |
| TRIM36 | 0 | 0.352804041066603 | 0.429166667 | 0.336805556 | 0 | Glut |
| PCDH9 | 0 | 0.35250216359004 | 0.241666667 | 0.100694444 | 0 | Glut |
| LHX1-DT | 0 | 0.351213350285951 | 0.229861111 | 0.077 | 0 | Glut |
| CHN2 | 0 | 0.350630392565974 | 0.239583333 | 0.086 | 0 | Glut |
| ATOX1 | 0 | 0.349485236974436 | 0.60625 | 0.575694444 | 0 | Glut |
| CCSER1 | 0 | 0.348653222141568 | 0.261805556 | 0.1 | 0 | Glut |
| RAB11FIP2 | 0 | 0.348275900170816 | 0.409027778 | 0.320138889 | 0 | Glut |
| CLCN3 | 0 | 0.347593646142951 | 0.398611111 | 0.293055556 | 0 | Glut |
| CELF2 | 0 | 0.346874847083409 | 0.403472222 | 00:43 | 0 | Glut |
| KIF1B | 0 | 0.345917132073002 | 0.536111111 | 0.457638889 | 0 | Glut |
| PRKAR2A | 0 | 0.345796605699522 | 0.40625 | 0.286111111 | 0 | Glut |
| CPE | 0 | 0.34573964821321 | 0.639583333 | 0.585416667 | 0 | Glut |
| APLP2 | 0 | 0.345303606493726 | 0.542361111 | 0.530555556 | 0 | Glut |
| JPH4 | 0 | 0.344413834054915 | 0.289583333 | 0.108333333 | 0 | Glut |
| CLTB | 0 | 0.343900683627092 | 0.527083333 | 0.433333333 | 0 | Glut |
| ATP6V0B | 0 | 0.343525343042064 | 0.661805556 | 0.649305556 | 0 | Glut |
| Sep-07 | 0 | 0.343413085700301 | 0.685416667 | 0.68125 | 0 | Glut |
| ZFHX3 | 0 | 0.343095366328329 | 0.586805556 | 0.397222222 | 0 | Glut |
| ATL1 | 0 | 0.342632197218439 | 0.049305556 | 0.408333333 | 0 | Glut |
| GABARAPL2 | 0 | 0.342631207011607 | 0.682638889 | 0.679861111 | 0 | Glut |
| DST | 0 | 0.341709682572486 | 0.516666667 | 0.454861111 | 0 | Glut |
| NFASC | 0 | 0.340714100053335 | 0.286805556 | 0.116666667 | 0 | Glut |
| ANKRD12 | 0 | 0.339500874653373 | 0.565277778 | 0.05 | 0 | Glut |
| CAMK2N1 | 0 | 0.335246166748701 | 0.615277778 | 0.558333333 | 0 | Glut |
| CTXN1 | 0 | 0.332394141456383 | 0.650694444 | 0.636805556 | 0 | Glut |
| SCD | 0 | 0.330709083888521 | 0.343055556 | 0.231944444 | 0 | Glut |
| FAM241B | 0 | 0.330220017202316 | 0.436111111 | 00:46 | 0 | Glut |
| PLPPR3 | 0 | 0.329048369840582 | 0.588194444 | 0.547222222 | 0 | Glut |
| NEFL | 0 | 0.327405191032719 | 0.636111111 | 0.340972222 | 0 | Glut |
| SHTN1 | 0 | 0.326100670957075 | 0.294444444 | 0.174305556 | 0 | Glut |
| GDAP1 | 0 | 0.326058512930381 | 0.471527778 | 00:58 | 0 | Glut |
| STX12 | 0 | 0.325926777567652 | 00:55 | 0.261805556 | 0 | Glut |
| PAFAH1B1 | 0 | 0.3256998346327 | 0.597916667 | 0.577777778 | 0 | Glut |
| KLC1 | 0 | 0.324753902081173 | 0.586111111 | 0.545833333 | 0 | Glut |
| RAB6B | 0 | 0.321373781135423 | 0.323611111 | 0.165277778 | 0 | Glut |
| PEA15 | 0 | 0.32114796662076 | 0.320833333 | 0.206944444 | 0 | Glut |
| SEC62 | 0 | 0.321057144017563 | 0.663194444 | 0.658333333 | 0 | Glut |
| TMEM196 | 0 | 0.320664660233072 | 0.265972222 | 0.115277778 | 0 | Glut |
| TACC2 | 0 | 0.320476431965137 | 0.295138889 | 00:23 | 0 | Glut |
| GABRB3 | 0 | 0.319257177408106 | 0.392361111 | 0.269444444 | 0 | Glut |
| DUSP8 | 0 | 0.318687027474786 | 0.272222222 | 0.108333333 | 0 | Glut |
| TM2D3 | 0 | 0.317301279606763 | 0.339583333 | 0.204861111 | 0 | Glut |
| ZCCHC12 | 0 | 0.317053181488777 | 0.261805556 | 0.098611111 | 0 | Glut |
| PAFAH1B3 | 0 | 0.315801761578915 | 0.672222222 | 0.654166667 | 0 | Glut |
| TCEAL4 | 0 | 0.315155249719017 | 0.600694444 | 0.585416667 | 0 | Glut |
| PLD3 | 0 | 0.314659203984107 | 0.578472222 | 0.522916667 | 0 | Glut |
| CELF3 | 0 | 0.314657148172726 | 0.456944444 | 0.351388889 | 0 | Glut |
| EIF1B | 0 | 0.313992970156825 | 0.684027778 | 0.684722222 | 0 | Glut |
| RASGRP1 | 0 | 0.313911956005555 | 0.314583333 | 0.189583333 | 0 | Glut |
| AP2B1 | 0 | 0.311710253820799 | 0.05 | 0.446527778 | 0 | Glut |
| G3BP2 | 0 | 0.311528434896647 | 0.624305556 | 0.609027778 | 0 | Glut |
| NACAD | 0 | 0.311233095092189 | 0.325 | 0.183333333 | 0 | Glut |

| TUBGCP4 | 0 | 0.310953056972516 | 0.447222222 | 0.38125 | 0 | Glut |
| --- | --- | --- | --- | --- | --- | --- |
| MEIS2 | 0 | 0.310950421501578 | 0.411111111 | 0.295138889 | 0 | Glut |
| EPB41L3 | 0 | 0.310444171247777 | 0.188888889 | 0.058 | 0 | Glut |
| SELENOW | 0 | 0.310203018405138 | 0.665972222 | 0.655555556 | 0 | Glut |
| NAP1L5 | 0 | 0.309979675724454 | 0.271527778 | 0.120138889 | 0 | Glut |
| PPP3CA | 0 | 0.309339614776713 | 0.338194444 | 0.202777778 | 0 | Glut |
| GDI1 | 0 | 0.309131853042868 | 0.517361111 | 0.439583333 | 0 | Glut |
| PHYHIPL | 0 | 0.308969048815919 | 0.429166667 | 0.293055556 | 0 | Glut |
| ATP6V0A1 | 0 | 0.308555685518873 | 0.365972222 | 0.257638889 | 0 | Glut |
| CACNA2D1 | 0 | 0.308006108873858 | 0.2875 | 00:23 | 0 | Glut |
| NEDD4L | 0 | 0.307315886772617 | 00:05 | 00:32 | 0 | Glut |
| SMDT1 | 0 | 0.306391112097645 | 0.060416667 | 0.058333333 | 0 | Glut |
| SH3GL3 | 0 | 0.305860048243161 | 0.226388889 | 0.098 | 0 | Glut |
| GSTA4 | 0 | 0.304984985436632 | 0.55625 | 0.525 | 0 | Glut |
| SCN2A | 0 | 0.304644529213582 | 0.219444444 | 0.074305556 | 0 | Glut |
| ZNF428 | 0 | 0.304271284055723 | 0.674305556 | 0.672222222 | 0 | Glut |
| LCOR | 0 | 0.304021645799173 | 0.489583333 | 0.431944444 | 0 | Glut |
| PGM2L1 | 0 | 0.303524868557606 | 0.452083333 | 0.372916667 | 0 | Glut |
| DNM1 | 0 | 0.303103263070541 | 0.265277778 | 00:17 | 0 | Glut |
| BLOC1S2 | 0 | 0.302611294055662 | 0.504166667 | 0.440277778 | 0 | Glut |
| TSPAN13 | 0 | 0.302465769595545 | 0.521527778 | 0.463888889 | 0 | Glut |
| ATP6V1G2 | 0 | 0.301849542517491 | 0.346527778 | 0.219444444 | 0 | Glut |
| DMD | 0 | 0.300944410746968 | 0.2 | 0.086 | 0 | Glut |
| CD200 | 0 | 0.300687757007396 | 0.45625 | 0.395138889 | 0 | Glut |
| MGST3 | 0 | 0.300195433391269 | 0.638194444 | 0.063194444 | 0 | Glut |
| DNER | 0 | 0.2999175583078 | 0.440972222 | 0.321527778 | 0 | Glut |
| TCTEX1D2 | 0 | 0.29946828814443 | 0.543055556 | 0.501388889 | 0 | Glut |
| KMT2E | 0 | 0.29877498286273 | 0.621527778 | 0.611805556 | 0 | Glut |
| SKIL | 0 | 0.298250553614913 | 0.468055556 | 0.386111111 | 0 | Glut |
| SOX11 | 0 | 0.297870325799183 | 0.690972222 | 0.692361111 | 0 | Glut |
| JAKMIP2 | 0 | 0.295505504804087 | 0.4875 | 0.426388889 | 0 | Glut |
| MPP3 | 0 | 0.294575038868997 | 0.197222222 | 0.067 | 0 | Glut |
| LDOC1 | 0 | 0.294398056924076 | 0.316666667 | 0.197222222 | 0 | Glut |
| RAB33A | 0 | 0.294116507257513 | 0.290277778 | 0.149305556 | 0 | Glut |
| PPP2R2C | 0 | 0.292641922474189 | 0.177777778 | 0.031 | 0 | Glut |
| DYNLT1 | 0 | 0.290687443983121 | 0.066666667 | 0.665972222 | 0 | Glut |
| TSPAN7 | 0 | 0.290022495686503 | 0.209722222 | 0.092 | 0 | Glut |
| NPDC1 | 0 | 0.28970762834465 | 0.454166667 | 0.379861111 | 0 | Glut |
| GNAS | 0 | 0.289262411425822 | 0.69375 | 1 | 0 | Glut |
| MT-ATP6 | 0 | 0.289016535909988 | 0.69375 | 0.690972222 | 0 | Glut |
| CACNG8 | 0 | 0.28649224797171 | 00:35 | 0.109027778 | 0 | Glut |
| TMEM160 | 0 | 0.28400413303874 | 0.526388889 | 0.047916667 | 0 | Glut |
| DCTN3 | 0 | 0.283867287962157 | 0.654166667 | 0.647222222 | 0 | Glut |
| SRGAP2C | 0 | 0.282355118823429 | 0.225 | 00:15 | 0 | Glut |
| C1QTNF4 | 0 | 0.281891967138803 | 00:42 | 0.151388889 | 0 | Glut |
| RABGAP1L | 0 | 0.281577711796925 | 0.2125 | 0.075 | 0 | Glut |
| SNAP25 | 0 | 0.281030274330473 | 0.25625 | 0.102083333 | 0 | Glut |
| NREP | 0 | 0.28057857023754 | 0.675 | 0.676388889 | 0 | Glut |
| SAMD5 | 0 | 0.277275042591465 | 0.176388889 | 00:05 | 0 | Glut |
| REEP1 | 0 | 0.275991270088068 | 0.213888889 | 0.074305556 | 0 | Glut |
| REEP2 | 0 | 0.275488365427111 | 0.308333333 | 0.186111111 | 0 | Glut |
| DPYSL3 | 0 | 0.275176896328766 | 0.630555556 | 0.609722222 | 0 | Glut |
| TUBB4A | 0 | 0.27497108786878 | 0.224305556 | 0.080555556 | 0 | Glut |
| ARHGDIG | 0 | 0.274432043326038 | 0.184027778 | 00:08 | 0 | Glut |
| CDK5R1 | 0 | 0.274039925913281 | 0.313194444 | 0.185416667 | 0 | Glut |
| SCN3B | 0 | 0.273675245911466 | 0.207638889 | 0.095 | 0 | Glut |
| DTD1 | 0 | 0.273478978206935 | 0.566666667 | 0.55 | 0 | Glut |
| RGMB | 0 | 0.269192096799261 | 0.266666667 | 0.124305556 | 0 | Glut |
| GPR153 | 0 | 0.268342776462755 | 0.207638889 | 0.085416667 | 0 | Glut |
| XKR4 | 0 | 0.268184491353786 | 0.221527778 | 0.081944444 | 0 | Glut |
| DNAJB6 | 0 | 0.267704965983677 | 0.646527778 | 0.647916667 | 0 | Glut |
| SNRPN | 0 | 0.267602834668681 | 0.642361111 | 0.640972222 | 0 | Glut |
| ATP8A2 | 0 | 0.267424325679243 | 00:31 | 00:13 | 0 | Glut |
| P4HTM | 0 | 0.267406068355349 | 0.253472222 | 00:19 | 0 | Glut |
| IRF2BPL | 0 | 0.266949663241884 | 0.259722222 | 00:18 | 0 | Glut |
| EPHA3 | 0 | 0.266936145243022 | 0.179861111 | 0.063 | 0 | Glut |
| Mar-06 | 0 | 0.263098495226915 | 0.591666667 | 0.572222222 | 0 | Glut |
| FABP5 | 0 | 0.262571118077476 | 0.550694444 | 0.474305556 | 0 | Glut |
| TBCB | 0 | 0.262304215899846 | 0.628472222 | 0.625694444 | 0 | Glut |
| RAB2A | 0 | 0.260332615108892 | 0.639583333 | 0.636805556 | 0 | Glut |
| TMEM59L | 0 | 0.259098618270611 | 0.234027778 | 0.096527778 | 0 | Glut |
| ATP1B2 | 0 | 0.257171868693047 | 0.215972222 | 0.095138889 | 0 | Glut |
| L1CAM | 0 | 0.255905116648227 | 00:38 | 0.119444444 | 0 | Glut |
| VWA5A | 0 | 0.255320328456122 | 0.178472222 | 0.078 | 0 | Glut |
| MYT1L | 0 | 0.253172305508382 | 0.2 | 0.096 | 0 | Glut |
| TMEM200A | 0 | 0.252672433034366 | 0.189583333 | 0.096 | 0 | Glut |
| CBX3 | 0 | 0.251157981121495 | 0.647222222 | 0.640277778 | 0 | Glut |
| SLC4A8 | 8.620020581432e-308 | 0.310708149758475 | 0.369444444 | 0.275 | 1.78977487332273e-303 | Glut |
| Sep-03 | 2.64765724880229e-307 | 0.283933578581971 | 0.36875 | 0.259722222 | 5.49733074568819e-303 | Glut |
| CCDC82 | 6.01058797970984e-307 | 0.286588493642391 | 0.422916667 | 0.345138889 | 1.24797838222715e-302 | Glut |
| PCSK1 | 1.27636994309928e-304 | 0.296952446302469 | 0.325694444 | 0.2 | 2.65012691285705e-300 | Glut |
| AC008522.1 | 1.85020758014855e-303 | 0.261193648907302 | 0.199305556 | 0.077083333 | 3.84158599866244e-299 | Glut |
| WRB | 2.34844120352827e-303 | 0.270643272753409 | 0.504166667 | 0.473611111 | 4.87606847088574e-299 | Glut |
| ST8SIA1 | 2.42862554426507e-303 | 0.258663637284281 | 0.209027778 | 00:13 | 5.04255521755756e-299 | Glut |
| APC | 1.07514830083082e-302 | 0.28376166417857 | 0.04375 | 00:51 | 2.23233041701503e-298 | Glut |
| PNMA8C | 2.92219607337728e-301 | 0.264149768922241 | 0.233333333 | 0.113888889 | 6.06735570715325e-297 | Glut |
| MYCBP2 | 2.62743990553189e-300 | 0.315100082832709 | 0.415277778 | 0.335416667 | 5.45535347585586e-296 | Glut |
| SBK1 | 6.23073426692808e-300 | 0.278044330813602 | 0.448611111 | 0.379861111 | 1.29368735584228e-295 | Glut |
| PAK5 | 1.23880290373071e-297 | 0.277240783705308 | 0.295138889 | 0.177083333 | 2.57212646901606e-293 | Glut |
| SMAP1 | 1.68595271832375e-296 | 0.274449195024272 | 0.501388889 | 0.460416667 | 3.5005436290556e-292 | Glut |
| SMARCC2 | 5.20306145005586e-296 | 0.293019476203769 | 0.495138889 | 0.463194444 | 1.0803116488751e-291 | Glut |
| RBFOX2 | 6.3440050581499e-296 | 0.27901998962071 | 0.530555556 | 0.510416667 | 1.31720577022366e-291 | Glut |
| ZNRF1 | 7.52922007170033e-296 | 0.272914293506705 | 0.482638889 | 0.443055556 | 1.56329196348714e-291 | Glut |
| LINC00632 | 4.03800614653987e-292 | 0.295727564747991 | 0.303472222 | 0.186111111 | 8.38411216206073e-288 | Glut |
| SYT14 | 1.59733272938939e-288 | 0.297066184190279 | 0.324305556 | 0.218055556 | 3.31654194603118e-284 | Glut |
| BTRC | 1.0461447470589e-286 | 0.296939451103284 | 0.307638889 | 0.206944444 | 2.17211033831839e-282 | Glut |
| ATP6V1A | 5.2262977835044e-283 | 0.280578632663023 | 0.310416667 | 0.202083333 | 1.08513620878902e-278 | Glut |
| THUMPD3-AS1 | 1.36743853883221e-282 | 0.270648725915172 | 0.521527778 | 0.49375 | 2.83921263817732e-278 | Glut |
| ABAT | 5.69061542642916e-281 | 0.289893623590124 | 0.427083333 | 0.35 | 1.18154248098949e-276 | Glut |
| PHACTR1 | 2.85146132807117e-279 | 0.2668387194276 | 0.273611111 | 0.159027778 | 5.92048915547418e-275 | Glut |

| NUDT3 | 4.39165612355914e-279 | 0.287784837428076 | 0.34375 | 0.242361111 | 9.11839560934585e-275 | Glut |
| --- | --- | --- | --- | --- | --- | --- |
| RIMS1 | 4.13889565790939e-278 | 0.25013540947626 | 0.204861111 | 0.09375 | 8.59358905451726e-274 | Glut |
| SLF1 | 2.27441891737949e-276 | 0.295502762336163 | 0.285416667 | 0.178472222 | 4.72237599815503e-272 | Glut |
| DICER1 | 9.2273778086199e-276 | 0.292481151443699 | 0.490277778 | 0.459722222 | 1.91588045440375e-271 | Glut |
| GNB2 | 4.84297725163063e-274 | 0.26068193718468 | 0.504166667 | 0.477083333 | 1.00554736675607e-269 | Glut |
| SSBP4 | 4.77699379294461e-271 | 0.268320266997882 | 0.463194444 | 0.413194444 | 9.91847221229089e-267 | Glut |
| OLFM1 | 7.0490493003576e-270 | 0.250948563932884 | 0.288888889 | 0.175694444 | 1.46359410623325e-265 | Glut |
| OCIAD2 | 1.69879297238135e-268 | 0.432715971925991 | 0.359027778 | 0.272222222 | 3.52720384855539e-264 | Glut |
| VKORC1 | 4.44096646559858e-266 | 0.283883019329737 | 0.443055556 | 0.391666667 | 9.22077867252234e-262 | Glut |
| TAOK3 | 3.9294493056802e-265 | 0.268712785552654 | 0.304861111 | 0.196527778 | 8.15871559338379e-261 | Glut |
| RSBN1L | 5.33343951102494e-264 | 0.259908485542459 | 0.49375 | 0.463194444 | 1.10738204567411e-259 | Glut |
| PCLO | 6.47251825109882e-264 | 0.275238512219371 | 0.288888889 | 0.175694444 | 1.34388896447565e-259 | Glut |
| SMIM14 | 1.5700465708587e-261 | 0.284536000294111 | 0.427777778 | 0.369444444 | 3.25988769507392e-257 | Glut |
| MINDY2 | 2.49345104766186e-260 | 0.271049311761437 | 0.361805556 | 0.271527778 | 5.17715241026031e-256 | Glut |
| LINC01003 | 1.77733977529748e-259 | 0.259110263526061 | 0.296527778 | 0.193055556 | 3.69029057545015e-255 | Glut |
| ARHGEF9 | 1.19193439058299e-258 | 0.264358918089086 | 0.477777778 | 0.434027778 | 2.47481337516747e-254 | Glut |
| SEC14L1 | 8.47182943853743e-256 | 0.267972900284267 | 0.418055556 | 0.360416667 | 1.75900594632353e-251 | Glut |
| FADS1 | 2.4252031829878e-253 | 0.262488105411239 | 0.5125 | 0.484027778 | 5.03544936883758e-249 | Glut |
| SQLE | 2.39778902679849e-252 | 0.250943056437441 | 0.613194444 | 0.602083333 | 4.97852935634171e-248 | Glut |
| MAPRE2 | 5.42778044305862e-252 | 0.253863243209821 | 0.275694444 | 0.16875 | 1.12697005339226e-247 | Glut |
| PIP4P2 | 1.27635691398421e-248 | 0.260299987898957 | 0.3 | 0.200694444 | 2.65009986050541e-244 | Glut |
| ARID4A | 2.32345832642292e-244 | 0.268048279388709 | 0.497222222 | 0.463888889 | 4.82419652315191e-240 | Glut |
| R3HDM2 | 3.23860546405864e-244 | 0.269956633530712 | 0.445138889 | 0.397916667 | 6.72431652502496e-240 | Glut |
| CRIP2 | 1.40011883367891e-242 | 0.262304173417621 | 0.427777778 | 0.365972222 | 2.90706673436752e-238 | Glut |
| IDS | 4.90216380221831e-242 | 0.264581704746925 | 0.377777778 | 0.304166667 | 1.01783627025459e-237 | Glut |
| FAM161A | 9.81978805030767e-242 | 0.274948820039398 | 0.322916667 | 0.232638889 | 2.03888259288538e-237 | Glut |
| CHD9 | 1.56198001366227e-237 | 0.252429247377242 | 0.5375 | 0.530555556 | 3.24313910236697e-233 | Glut |
| DCTN1 | 1.26920622390152e-235 | 0.259712594745717 | 0.44375 | 0.405555556 | 2.63525288268672e-231 | Glut |
| MT-ND4L | 2.40621675328605e-235 | 0.254414829347251 | 0.549305556 | 0.543055556 | 4.99602784484783e-231 | Glut |
| TXNDC16 | 8.79986058577463e-234 | 0.266385039837181 | 0.289583333 | 0.191666667 | 1.82711505342439e-229 | Glut |
| TNRC6C | 1.75328665355364e-233 | 0.267832394394712 | 0.363888889 | 0.278472222 | 3.64034907877342e-229 | Glut |
| DNMT3A | 1.25352733031576e-231 | 0.286454463172552 | 00:49 | 0.263194444 | 2.60269879593461e-227 | Glut |
| LNPK | 2.43025440569746e-230 | 0.267674477010248 | 0.329166667 | 00:36 | 5.04593722254964e-226 | Glut |
| RUFY2 | 1.65882783528136e-226 | 0.255543293529692 | 00:45 | 0.221527778 | 3.44422423439469e-222 | Glut |
| ABHD14A | 1.92966196433369e-225 | 0.261580770597396 | 0.405555556 | 00:05 | 4.00655713654604e-221 | Glut |
| NRP1 | 3.20710621133008e-225 | 0.275976946590648 | 0.379861111 | 0.304166667 | 6.65891462658465e-221 | Glut |
| COMMD9 | 1.10376381797834e-224 | 0.253603080445707 | 0.363888889 | 0.295138889 | 2.29174481526842e-220 | Glut |
| CISD1 | 8.32616650244539e-224 | 0.258425495097534 | 0.411111111 | 0.355555556 | 1.72876195090274e-219 | Glut |
| TMEM178A | 3.29233596221448e-222 | 0.260304289865576 | 0.246527778 | 0.155555556 | 6.83587715834592e-218 | Glut |
| MTUS1 | 6.59446766524257e-220 | 0.272320298224518 | 0.282638889 | 0.193055556 | 1.36920932133431e-215 | Glut |
| ONECUT1 | 3.35875956284517e-213 | 0.254232434624119 | 0.259722222 | 0.1625 | 6.97379248033542e-209 | Glut |
| FTX | 4.37942202334794e-208 | 0.271912303280447 | 0.536111111 | 0.531944444 | 9.09299394707734e-204 | Glut |
| RNF2 | 2.51377297234223e-203 | 0.270385143232785 | 0.345833333 | 00:41 | 5.21934682247417e-199 | Glut |
| LHX9 | 6.24804199904757e-197 | 0.266703848965653 | 0.404166667 | 0.306944444 | 1.29728096026225e-192 | Glut |
| TCEAL2 | 5.28497731792842e-181 | 0.264282994066397 | 0.188888889 | 0.100694444 | 1.09731984052148e-176 | Glut |
| CASK | 4.51981868803235e-161 | 0.257913147027407 | 0.336111111 | 0.286111111 | 9.38449954196157e-157 | Glut |
| TARS | 1.17349970872947e-153 | 0.250990038646878 | 0.359722222 | 0.320833333 | 2.436537445235e-149 | Glut |
| DZIP3 | 9.02193915002484e-149 | 0.250502843694366 | 0.317361111 | 0.26875 | 1.87322522571966e-144 | Glut |
| GNG8 | 2.2944807947013e-106 | 0.345841239581655 | 0.350694444 | 0.302777778 | 4.76403047403831e-102 | Glut |
| TRH | 1.04226986117675e-86 | 0.278461820007192 | 0.228472222 | 00:25 | 2.16406491276128e-82 | Glut |
| CTNNB1 | 6.85734311493951e-70 | 0.272035856104016 | 0.542361111 | 0.056944444 | 1.42379015095489e-65 | Glut |
| DLK1 | 0 | 2.2094780098632 | 0.640972222 | 0.245138889 | 0 | NNPs |
| HES6 | 0 | 1.94102084240231 | 0.63125 | 0.253472222 | 0 | NNPs |
| IGFBP2 | 0 | 1.6596415891715 | 0.679861111 | 0.500694444 | 0 | NNPs |
| NEUROG1 | 0 | 1.52836986867901 | 0.585416667 | 0.106944444 | 0 | NNPs |
| CKB | 0 | 1.37878980406229 | 0.690972222 | 0.663888889 | 0 | NNPs |
| FEZF2 | 0 | 1.32209463633033 | 0.582638889 | 0.089583333 | 0 | NNPs |
| CDKN1C | 0 | 1.26927125622129 | 0.677777778 | 0.558333333 | 0 | NNPs |
| SPRY1 | 0 | 1.26511446311022 | 0.634722222 | 0.204861111 | 0 | NNPs |
| VIM | 0 | 1.25890546055824 | 0.68125 | 0.533333333 | 0 | NNPs |
| GADD45G | 0 | 1.15907965922419 | 0.567361111 | 0.143055556 | 0 | NNPs |
| CFAP298 | 0 | 1.11679686249272 | 0.615277778 | 0.395138889 | 0 | NNPs |
| DLL1 | 0 | 1.0714071667555 | 0.488194444 | 0.095833333 | 0 | NNPs |
| DLL3 | 0 | 1.06630151870701 | 0.608333333 | 0.275694444 | 0 | NNPs |
| TFDP2 | 0 | 1.05711170886159 | 0.642361111 | 0.338194444 | 0 | NNPs |
| LINC01551 | 0 | 1.03625918040922 | 0.663194444 | 0.379166667 | 0 | NNPs |
| PRSS23 | 0 | 1.02281649124284 | 0.606944444 | 0.154166667 | 0 | NNPs |
| MDK | 0 | 0.99483469053979 | 0.68125 | 0.547222222 | 0 | NNPs |
| RPS2 | 0 | 0.992284729541784 | 1 | 0.692361111 | 0 | NNPs |
| GNG5 | 0 | 0.975572870838293 | 0.668055556 | 0.331944444 | 0 | NNPs |
| BTBD17 | 0 | 0.904919623995492 | 0.504861111 | 0.133333333 | 0 | NNPs |
| EEF1D | 0 | 0.869365302554295 | 0.672916667 | 0.502777778 | 0 | NNPs |
| DMRTA1 | 0 | 0.860336620377791 | 0.513194444 | 00:12 | 0 | NNPs |
| NPM1 | 0 | 0.855513952163284 | 0.69375 | 0.679861111 | 0 | NNPs |
| MYO10 | 0 | 0.849455744721501 | 0.606944444 | 0.275 | 0 | NNPs |
| C1orf61 | 0 | 0.847488677502685 | 0.327083333 | 0.068 | 0 | NNPs |
| SSTR2 | 0 | 0.822139311901529 | 0.296527778 | 0.054 | 0 | NNPs |
| CTNNA1 | 0 | 0.770874748052353 | 0.059027778 | 0.260416667 | 0 | NNPs |
| GLUL | 0 | 0.766204503148657 | 0.624305556 | 00:53 | 0 | NNPs |
| RPS27L | 0 | 0.757645448302305 | 0.661805556 | 0.461805556 | 0 | NNPs |
| NEUROD4 | 0 | 0.748455727278124 | 0.338888889 | 0.039 | 0 | NNPs |
| RPL10A | 0 | 0.739777826263166 | 0.69375 | 0.068055556 | 0 | NNPs |
| SYNE2 | 0 | 0.726329861572397 | 0.636111111 | 0.459722222 | 0 | NNPs |
| NHLH1 | 0 | 0.72591673788389 | 0.3875 | 0.096 | 0 | NNPs |
| KCNQ1OT1 | 0 | 0.724150502050483 | 0.661111111 | 0.533333333 | 0 | NNPs |
| HMGN2 | 0 | 0.724094436009784 | 0.685416667 | 0.620833333 | 0 | NNPs |
| FBLN1 | 0 | 0.716080250376452 | 0.634722222 | 0.363888889 | 0 | NNPs |
| HNRNPA1 | 0 | 0.71546890278397 | 0.69375 | 0.684722222 | 0 | NNPs |
| RPS6 | 0 | 0.715098346892387 | 1 | 0.690972222 | 0 | NNPs |
| HSPB1 | 0 | 0.713239742079519 | 0.511805556 | 0.11875 | 0 | NNPs |
| SMOC1 | 0 | 0.710083710270494 | 00:59 | 00:08 | 0 | NNPs |
| SRRM4 | 0 | 0.697858180550497 | 0.563888889 | 0.309722222 | 0 | NNPs |
| TTYH1 | 0 | 0.6978101731539 | 0.502083333 | 0.172222222 | 0 | NNPs |
| ZBTB18 | 0 | 0.696490308033235 | 0.488194444 | 0.10625 | 0 | NNPs |
| CBFA2T2 | 0 | 0.694901993996176 | 0.497916667 | 0.147916667 | 0 | NNPs |
| NEUROD1 | 0 | 0.691019612733924 | 0.45 | 0.122916667 | 0 | NNPs |
| COL4A6 | 0 | 0.689112112809057 | 0.464583333 | 0.075 | 0 | NNPs |
| GSTP1 | 0 | 0.676804908574376 | 0.679166667 | 0.54375 | 0 | NNPs |
| KLHDC8A | 0 | 0.675250503120686 | 0.414583333 | 0.082638889 | 0 | NNPs |
| CHRNA3 | 0 | 0.672258567211644 | 0.369444444 | 0.061 | 0 | NNPs |

| ODC1 | 0 | 0.669131026393504 | 0.688194444 | 0.635416667 | 0 | NNPs |
| --- | --- | --- | --- | --- | --- | --- |
| PRDX1 | 0 | 0.658019899243064 | 0.677083333 | 0.565277778 | 0 | NNPs |
| RPLP0 | 0 | 0.656749367470816 | 1 | 0.689583333 | 0 | NNPs |
| HNRNPM | 0 | 0.654719089130464 | 0.668055556 | 0.502777778 | 0 | NNPs |
| CCND1 | 0 | 0.648586488755929 | 0.334722222 | 00:06 | 0 | NNPs |
| RPLP1 | 0 | 0.644383123924087 | 1 | 1 | 0 | NNPs |
| RPS18 | 0 | 0.641230077432208 | 1 | 0.69375 | 0 | NNPs |
| RPS9 | 0 | 0.640105544867025 | 1 | 0.689583333 | 0 | NNPs |
| RPL12 | 0 | 0.625642275724016 | 1 | 0.691666667 | 0 | NNPs |
| RAB13 | 0 | 0.625340152899444 | 0.511111111 | 0.109722222 | 0 | NNPs |
| RPS20 | 0 | 0.623852248083352 | 0.69375 | 0.682638889 | 0 | NNPs |
| RPS19 | 0 | 0.6159414604758 | 1 | 1 | 0 | NNPs |
| HNRNPC | 0 | 0.615756561782426 | 0.675694444 | 0.574305556 | 0 | NNPs |
| NAP1L1 | 0 | 0.613893996980089 | 0.693055556 | 0.679166667 | 0 | NNPs |
| RPL41 | 0 | 0.611256838936156 | 1 | 1 | 0 | NNPs |
| RPSA | 0 | 0.609172011288571 | 1 | 0.688888889 | 0 | NNPs |
| EEF1B2 | 0 | 0.606227665518057 | 0.691666667 | 0.066666667 | 0 | NNPs |
| NPC2 | 0 | 0.603903159863124 | 0.052777778 | 0.174305556 | 0 | NNPs |
| RBMX | 0 | 0.596444142667938 | 0.677777778 | 0.568055556 | 0 | NNPs |
| GPC3 | 0 | 0.59205154100435 | 0.413194444 | 0.077 | 0 | NNPs |
| PABPC1 | 0 | 0.592049781407172 | 0.06875 | 0.622222222 | 0 | NNPs |
| SH3BGRL | 0 | 0.5882726029739 | 0.604861111 | 0.332638889 | 0 | NNPs |
| UBE2L6 | 0 | 0.585624696439514 | 0.6 | 0.384722222 | 0 | NNPs |
| SOX2 | 0 | 0.583354570124757 | 0.421527778 | 0.094444444 | 0 | NNPs |
| NES | 0 | 0.576132528753989 | 0.560416667 | 0.279166667 | 0 | NNPs |
| SPARC | 0 | 0.572959757883673 | 0.401388889 | 0.091 | 0 | NNPs |
| PCP4 | 0 | 0.567658937843517 | 0.279166667 | 00:06 | 0 | NNPs |
| MFNG | 0 | 0.567272966368541 | 0.4 | 0.061 | 0 | NNPs |
| RPS7 | 0 | 0.566204459663489 | 1 | 0.69375 | 0 | NNPs |
| PHC2 | 0 | 0.565948901792727 | 0.604861111 | 0.336111111 | 0 | NNPs |
| SRSF3 | 0 | 0.564593765480842 | 0.68125 | 0.581944444 | 0 | NNPs |
| NKAIN4 | 0 | 0.558065943953542 | 0.057638889 | 0.264583333 | 0 | NNPs |
| WNT5B | 0 | 0.557098724712804 | 0.456944444 | 0.1 | 0 | NNPs |
| RPS15 | 0 | 0.551310700420598 | 1 | 0.69375 | 0 | NNPs |
| ANP32B | 0 | 0.550844268836466 | 0.630555556 | 0.414583333 | 0 | NNPs |
| RPL27A | 0 | 0.54506495271798 | 0.69375 | 0.684722222 | 0 | NNPs |
| MSI1 | 0 | 0.544042341796082 | 0.588194444 | 0.33125 | 0 | NNPs |
| HMGN3 | 0 | 0.543135760425171 | 0.65 | 0.446527778 | 0 | NNPs |
| OSTC | 0 | 0.539884892184602 | 0.64375 | 0.434722222 | 0 | NNPs |
| WFIKKN1 | 0 | 0.536413632067092 | 00:54 | 0.072 | 0 | NNPs |
| ZBTB16 | 0 | 0.531675331599857 | 0.420833333 | 0.081944444 | 0 | NNPs |
| NIN | 0 | 0.525518725516731 | 0.456944444 | 0.157638889 | 0 | NNPs |
| CCNB1IP1 | 0 | 0.525261982912951 | 0.53125 | 0.200694444 | 0 | NNPs |
| VSX1 | 0 | 0.52461699238808 | 0.203472222 | 0.018 | 0 | NNPs |
| TP53I11 | 0 | 0.523153983866491 | 0.509027778 | 00:38 | 0 | NNPs |
| TSPAN6 | 0 | 0.520875074627857 | 0.536805556 | 0.235416667 | 0 | NNPs |
| TMEM98 | 0 | 0.519579348349013 | 0.577777778 | 0.28125 | 0 | NNPs |
| GJA1 | 0 | 0.519169762670083 | 0.313888889 | 0.047 | 0 | NNPs |
| RCN2 | 0 | 0.51724397598218 | 0.692361111 | 0.68125 | 0 | NNPs |
| FEZF1 | 0 | 0.514440995449079 | 0.475 | 0.14375 | 0 | NNPs |
| ZIC3 | 0 | 0.514149775676388 | 0.432638889 | 00:17 | 0 | NNPs |
| INSM1 | 0 | 0.513786531146175 | 0.589583333 | 0.370138889 | 0 | NNPs |
| SLC6A8 | 0 | 0.510866668809054 | 0.469444444 | 0.152083333 | 0 | NNPs |
| HSPD1 | 0 | 0.509999275269045 | 0.675694444 | 0.572222222 | 0 | NNPs |
| RNF130 | 0 | 0.508852241043765 | 0.571527778 | 0.29375 | 0 | NNPs |
| AC092958.1 | 0 | 0.508751617257326 | 0.392361111 | 0.119444444 | 0 | NNPs |
| SNRPF | 0 | 0.507010133326776 | 0.679166667 | 0.582638889 | 0 | NNPs |
| POLR2L | 0 | 0.504202693812225 | 0.671527778 | 0.561805556 | 0 | NNPs |
| ZFP36L1 | 0 | 0.503386451490033 | 0.261805556 | 0.078 | 0 | NNPs |
| RGS16 | 0 | 0.500824740576584 | 0.248611111 | 0.027 | 0 | NNPs |
| BDH2 | 0 | 0.498730445542603 | 0.442361111 | 0.1125 | 0 | NNPs |
| CCND2 | 0 | 0.491523896837342 | 0.407638889 | 0.072916667 | 0 | NNPs |
| CA4 | 0 | 0.491074817386612 | 0.224305556 | 0.018 | 0 | NNPs |
| TP53I3 | 0 | 0.490528862055218 | 0.552777778 | 0.265972222 | 0 | NNPs |
| H2AFV | 0 | 0.488604078025136 | 0.664583333 | 0.502083333 | 0 | NNPs |
| ENY2 | 0 | 0.485318253263157 | 0.675 | 0.559722222 | 0 | NNPs |
| CDC42EP4 | 0 | 0.484398094732413 | 0.410416667 | 0.113194444 | 0 | NNPs |
| RPL3 | 0 | 0.484211248680971 | 1 | 0.69375 | 0 | NNPs |
| TMEM123 | 0 | 0.482068301635446 | 0.415972222 | 0.0875 | 0 | NNPs |
| RPL18A | 0 | 0.481238385990853 | 1 | 0.693055556 | 0 | NNPs |
| SERPINF1 | 0 | 0.480670998997814 | 0.327777778 | 00:05 | 0 | NNPs |
| RCN1 | 0 | 0.475900452636607 | 0.559027778 | 0.283333333 | 0 | NNPs |
| SNRPE | 0 | 0.475461122025808 | 0.686805556 | 0.619444444 | 0 | NNPs |
| TMEM97 | 0 | 0.475281774136739 | 0.640277778 | 0.436805556 | 0 | NNPs |
| RPL23A | 0 | 0.475226641024365 | 1 | 0.688194444 | 0 | NNPs |
| PTTG1 | 0 | 0.473963574980969 | 0.377777778 | 0.085416667 | 0 | NNPs |
| RACK1 | 0 | 0.472685213981191 | 1 | 0.692361111 | 0 | NNPs |
| ZIC1 | 0 | 0.47128318930171 | 0.640972222 | 0.481944444 | 0 | NNPs |
| TEAD2 | 0 | 0.469462267443075 | 00:58 | 0.075 | 0 | NNPs |
| HNRNPH3 | 0 | 0.468983148288257 | 0.065972222 | 0.052777778 | 0 | NNPs |
| DIO3 | 0 | 0.466822283017138 | 0.278472222 | 0.103472222 | 0 | NNPs |
| CSRP2 | 0 | 0.463433553775241 | 0.65625 | 0.552083333 | 0 | NNPs |
| PRAG1 | 0 | 0.463087353962176 | 0.338194444 | 0.069 | 0 | NNPs |
| RPS12 | 0 | 0.462903287136195 | 1 | 1 | 0 | NNPs |
| SEM1 | 0 | 0.460307917294045 | 0.679166667 | 0.584722222 | 0 | NNPs |
| TOX3 | 0 | 0.458459003208879 | 0.542361111 | 0.266666667 | 0 | NNPs |
| CCNG1 | 0 | 0.458321244918087 | 0.634722222 | 0.446527778 | 0 | NNPs |
| PAICS | 0 | 0.458303661228531 | 0.509722222 | 0.211805556 | 0 | NNPs |
| MIB1 | 0 | 0.457539446900477 | 0.511805556 | 0.272222222 | 0 | NNPs |
| HSP90AB1 | 0 | 0.456559278519312 | 0.69375 | 0.69375 | 0 | NNPs |
| CYYR1 | 0 | 0.454875032152175 | 0.363888889 | 0.074305556 | 0 | NNPs |
| KCNQ2 | 0 | 0.454452333861695 | 0.518055556 | 0.24375 | 0 | NNPs |
| RPL29 | 0 | 0.452347131410858 | 1 | 0.69375 | 0 | NNPs |
| CPLX2 | 0 | 0.452258775482263 | 0.330555556 | 0.127777778 | 0 | NNPs |
| C3orf58 | 0 | 0.451866357925458 | 0.370833333 | 00:14 | 0 | NNPs |
| RPS3 | 0 | 0.451856814940526 | 1 | 1 | 0 | NNPs |
| ZIC4 | 0 | 0.448386776394708 | 0.420833333 | 0.147222222 | 0 | NNPs |
| SNRPD2 | 0 | 0.447782267072181 | 0.68125 | 0.059722222 | 0 | NNPs |
| CHD7 | 0 | 0.446374640273907 | 0.635416667 | 0.045833333 | 0 | NNPs |
| RPL8 | 0 | 0.443848347899526 | 1 | 0.69375 | 0 | NNPs |

| RPS8 | 0 | 0.443192992661892 | 1 | 1 | 0 | NNPs |
| --- | --- | --- | --- | --- | --- | --- |
| RPL35 | 0 | 0.441706692584404 | 1 | 0.689583333 | 0 | NNPs |
| PRDX6 | 0 | 0.441129865745013 | 0.619444444 | 0.425694444 | 0 | NNPs |
| RPL13A | 0 | 0.438733115329758 | 0.69375 | 0.690972222 | 0 | NNPs |
| LDHB | 0 | 0.438216244532713 | 0.69375 | 0.684722222 | 0 | NNPs |
| HDGF | 0 | 0.438004087252662 | 0.605555556 | 0.386805556 | 0 | NNPs |
| ADH5 | 0 | 0.436840052273797 | 0.663888889 | 0.525694444 | 0 | NNPs |
| NKAIN3 | 0 | 0.428941142019388 | 0.418055556 | 0.158333333 | 0 | NNPs |
| IGF2BP1 | 0 | 0.428730136310403 | 0.531944444 | 0.264583333 | 0 | NNPs |
| RPL13 | 0 | 0.423168585158815 | 1 | 1 | 0 | NNPs |
| RPS5 | 0 | 0.42149166534149 | 1 | 0.691666667 | 0 | NNPs |
| HMGA1 | 0 | 0.421078890554174 | 0.589583333 | 0.395138889 | 0 | NNPs |
| MEG3 | 0 | 0.420727127881582 | 0.294444444 | 0.044 | 0 | NNPs |
| CLNS1A | 0 | 0.420256878601114 | 0.635416667 | 0.445833333 | 0 | NNPs |
| SNRPB | 0 | 0.41813818145702 | 0.613888889 | 0.385416667 | 0 | NNPs |
| ASS1 | 0 | 0.41711357809881 | 0.29375 | 0.074 | 0 | NNPs |
| RPL7 | 0 | 0.41641592817379 | 1 | 0.692361111 | 0 | NNPs |
| TCF3 | 0 | 0.416342674896701 | 0.047222222 | 0.186805556 | 0 | NNPs |
| RPL19 | 0 | 0.414821542125309 | 1 | 0.69375 | 0 | NNPs |
| SLC25A3 | 0 | 0.413528371430511 | 0.691666667 | 0.675 | 0 | NNPs |
| CALD1 | 0 | 0.413266662592321 | 0.4125 | 0.154861111 | 0 | NNPs |
| RPL7A | 0 | 0.411540486353442 | 1 | 1 | 0 | NNPs |
| RPL23 | 0 | 0.411367526967735 | 1 | 0.69375 | 0 | NNPs |
| JARID2 | 0 | 0.408556940933621 | 0.478472222 | 0.24375 | 0 | NNPs |
| NNAT | 0 | 0.406896777803861 | 0.68125 | 0.679861111 | 0 | NNPs |
| CDK4 | 0 | 0.406057247536533 | 0.552083333 | 0.295833333 | 0 | NNPs |
| CAST | 0 | 0.403466233916039 | 0.428472222 | 0.185416667 | 0 | NNPs |
| ATP2B1 | 0 | 0.401133779994646 | 0.592361111 | 0.047222222 | 0 | NNPs |
| HOMER3 | 0 | 0.399890857090588 | 0.054166667 | 0.3 | 0 | NNPs |
| TMEM256 | 0 | 0.398595223023913 | 0.567361111 | 0.322222222 | 0 | NNPs |
| PNRC1 | 0 | 0.39808259859587 | 0.644444444 | 0.511111111 | 0 | NNPs |
| RCOR2 | 0 | 0.39666177113349 | 0.454166667 | 0.18125 | 0 | NNPs |
| LRRN1 | 0 | 0.395545821501713 | 0.5125 | 0.272222222 | 0 | NNPs |
| IFITM3 | 0 | 0.394123147171484 | 0.31875 | 0.067 | 0 | NNPs |
| RPS14 | 0 | 0.392195179382972 | 1 | 1 | 0 | NNPs |
| RASGEF1B | 0 | 0.390923539286069 | 0.266666667 | 0.071 | 0 | NNPs |
| MYL12A | 0 | 0.389729849089416 | 0.552777778 | 0.322916667 | 0 | NNPs |
| NONO | 0 | 0.38871633166706 | 0.665277778 | 0.55 | 0 | NNPs |
| ANXA5 | 0 | 0.387359045968848 | 0.499305556 | 0.230555556 | 0 | NNPs |
| RPS17 | 0 | 0.386929634923455 | 0.644444444 | 0.491666667 | 0 | NNPs |
| SIX3 | 0 | 0.386360431879774 | 0.582638889 | 0.369444444 | 0 | NNPs |
| RPN2 | 0 | 0.386143735708404 | 0.540277778 | 0.308333333 | 0 | NNPs |
| RPLP2 | 0 | 0.385641517727629 | 1 | 0.69375 | 0 | NNPs |
| RPL21 | 0 | 0.384179068538797 | 1 | 0.69375 | 0 | NNPs |
| CRYBG3 | 0 | 0.382850877441649 | 0.300694444 | 00:08 | 0 | NNPs |
| ANP32E | 0 | 0.382583748960094 | 0.540277778 | 0.293055556 | 0 | NNPs |
| TIMP2 | 0 | 0.379945541843442 | 0.543055556 | 0.351388889 | 0 | NNPs |
| OTX2 | 0 | 0.37944841817013 | 0.396527778 | 0.127777778 | 0 | NNPs |
| CLIC1 | 0 | 0.376828879718517 | 0.621527778 | 0.36875 | 0 | NNPs |
| IGFBP5 | 0 | 0.376257883571847 | 0.209027778 | 0.049 | 0 | NNPs |
| MED28 | 0 | 0.374972452626038 | 0.572222222 | 0.349305556 | 0 | NNPs |
| SEC11A | 0 | 0.373448347895564 | 0.684027778 | 0.607638889 | 0 | NNPs |
| RPS24 | 0 | 0.37269255963342 | 1 | 1 | 0 | NNPs |
| TOP1 | 0 | 0.37262586137769 | 0.672222222 | 0.571527778 | 0 | NNPs |
| AHCY | 0 | 0.372454535507742 | 0.383333333 | 0.1125 | 0 | NNPs |
| TCEAL9 | 0 | 0.370063167100239 | 0.530555556 | 0.322222222 | 0 | NNPs |
| PCBP4 | 0 | 0.369393073815757 | 0.622916667 | 0.475 | 0 | NNPs |
| HSP90B1 | 0 | 0.368583801809881 | 0.640277778 | 0.525 | 0 | NNPs |
| CNIH4 | 0 | 0.368017205841901 | 0.581944444 | 0.360416667 | 0 | NNPs |
| TJP1 | 0 | 0.367774841108403 | 0.523611111 | 0.270138889 | 0 | NNPs |
| ECI2 | 0 | 0.365254541869784 | 0.471527778 | 0.241666667 | 0 | NNPs |
| MAGOH | 0 | 0.363431983228536 | 0.530555556 | 0.290277778 | 0 | NNPs |
| RPS16 | 0 | 0.36074002242274 | 1 | 0.690972222 | 0 | NNPs |
| RBM3 | 0 | 0.360600849996253 | 0.584722222 | 0.370138889 | 0 | NNPs |
| SINHCAF | 0 | 0.359761717880092 | 00:09 | 0.433333333 | 0 | NNPs |
| EIF3E | 0 | 0.359597108365076 | 0.684027778 | 0.627083333 | 0 | NNPs |
| SERF2 | 0 | 0.359146083925875 | 0.69375 | 0.688194444 | 0 | NNPs |
| PON2 | 0 | 0.358842079600492 | 0.452083333 | 0.204166667 | 0 | NNPs |
| GADD45A | 0 | 0.358463061055038 | 0.292361111 | 0.128472222 | 0 | NNPs |
| YBX3 | 0 | 0.357975382201504 | 0.314583333 | 0.085 | 0 | NNPs |
| ME2 | 0 | 0.356207792343562 | 0.5375 | 0.321527778 | 0 | NNPs |
| RTRAF | 0 | 0.354946217221133 | 0.06875 | 0.636805556 | 0 | NNPs |
| C4orf3 | 0 | 0.354842630225404 | 0.629861111 | 0.454861111 | 0 | NNPs |
| APEX1 | 0 | 0.352815359345595 | 0.665277778 | 0.563888889 | 0 | NNPs |
| SLC16A2 | 0 | 0.352021013333241 | 0.538888889 | 0.359722222 | 0 | NNPs |
| RAN | 0 | 0.351713500461266 | 0.690277778 | 0.660416667 | 0 | NNPs |
| RPS25 | 0 | 0.350854578278444 | 1 | 0.69375 | 0 | NNPs |
| CRB2 | 0 | 0.349994601600506 | 00:57 | 0.133333333 | 0 | NNPs |
| AMOTL2 | 0 | 0.349047087864554 | 0.347916667 | 0.143055556 | 0 | NNPs |
| CHCHD6 | 0 | 0.348398668879542 | 0.497916667 | 0.276388889 | 0 | NNPs |
| IGFBPL1 | 0 | 0.347999927064198 | 0.4875 | 0.318055556 | 0 | NNPs |
| DPPA4 | 0 | 0.347647238197444 | 0.282638889 | 0.071 | 0 | NNPs |
| ANP32A | 0 | 0.347598081058698 | 0.617361111 | 0.433333333 | 0 | NNPs |
| ATP5MC2 | 0 | 0.347043343271901 | 1 | 0.690277778 | 0 | NNPs |
| EIF4A1 | 0 | 0.346656358300578 | 0.635416667 | 0.484722222 | 0 | NNPs |
| RPL27 | 0 | 0.345958501715862 | 0.69375 | 0.686805556 | 0 | NNPs |
| HUNK | 0 | 0.344841420545997 | 0.279166667 | 0.096 | 0 | NNPs |
| NOTCH1 | 0 | 0.344644574944547 | 0.279861111 | 0.046 | 0 | NNPs |
| ELAVL2 | 0 | 0.343098639206862 | 0.560416667 | 0.389583333 | 0 | NNPs |
| MFAP2 | 0 | 0.342526589260629 | 0.427083333 | 0.189583333 | 0 | NNPs |
| BSG | 0 | 0.342002010672054 | 0.623611111 | 0.459722222 | 0 | NNPs |
| AL391069.3 | 0 | 0.341774642358956 | 0.317361111 | 00:11 | 0 | NNPs |
| HSPE1 | 0 | 0.341134712227447 | 0.654166667 | 0.531944444 | 0 | NNPs |
| EIF3D | 0 | 0.340061086326175 | 0.538194444 | 0.311111111 | 0 | NNPs |
| ZEB1 | 0 | 0.340026969758563 | 0.528472222 | 0.325694444 | 0 | NNPs |
| PSMA4 | 0 | 0.339198854845792 | 0.6625 | 0.054861111 | 0 | NNPs |
| RPL36 | 0 | 0.339123262765556 | 1 | 0.693055556 | 0 | NNPs |
| SRP14 | 0 | 0.33745475719203 | 1 | 1 | 0 | NNPs |
| BCL7A | 0 | 0.336876404049423 | 0.579166667 | 0.424305556 | 0 | NNPs |
| DECR1 | 0 | 0.335351902266767 | 0.445833333 | 0.215972222 | 0 | NNPs |

| RPS27A | 0 | 0.335286325670319 | 1 | 1 | 0 | NNPs |
| --- | --- | --- | --- | --- | --- | --- |
| DDR1 | 0 | 0.334994815935488 | 0.556944444 | 0.339583333 | 0 | NNPs |
| CCNG2 | 0 | 0.327347586265684 | 0.5125 | 0.321527778 | 0 | NNPs |
| ZDHHC14 | 0 | 0.327335151964886 | 0.265277778 | 0.072 | 0 | NNPs |
| AGO1 | 0 | 0.326266539823127 | 0.406944444 | 0.18125 | 0 | NNPs |
| CHRNA5 | 0 | 0.325742094750417 | 0.286805556 | 0.087 | 0 | NNPs |
| RPL22 | 0 | 0.325723436354106 | 1 | 0.692361111 | 0 | NNPs |
| PMPCB | 0 | 0.32443319507962 | 0.54375 | 0.33125 | 0 | NNPs |
| HNRNPAB | 0 | 0.323785999356131 | 0.681944444 | 0.624305556 | 0 | NNPs |
| MECOM | 0 | 0.323390482399579 | 0.265972222 | 0.067 | 0 | NNPs |
| JAM2 | 0 | 0.322775207243569 | 0.311111111 | 0.075694444 | 0 | NNPs |
| EIF3G | 0 | 0.32104116529256 | 0.598611111 | 0.4125 | 0 | NNPs |
| PLK3 | 0 | 0.318430745825014 | 0.279861111 | 0.078 | 0 | NNPs |
| ASCL1 | 0 | 0.318120161223564 | 0.228472222 | 0.070138889 | 0 | NNPs |
| CCDC88C | 0 | 0.317941548957894 | 00:43 | 0.080555556 | 0 | NNPs |
| RPL31 | 0 | 0.317794373843886 | 0.69375 | 0.685416667 | 0 | NNPs |
| STK17A | 0 | 0.317730889602529 | 0.4125 | 0.188194444 | 0 | NNPs |
| CCT4 | 0 | 0.317596057304403 | 0.654861111 | 0.529166667 | 0 | NNPs |
| AKR1A1 | 0 | 0.317079879225449 | 0.477777778 | 0.249305556 | 0 | NNPs |
| RPL36A | 0 | 0.317014871790212 | 0.684027778 | 0.619444444 | 0 | NNPs |
| CDH2 | 0 | 0.316003372858792 | 0.675694444 | 0.609027778 | 0 | NNPs |
| RPS21 | 0 | 0.315945079119652 | 0.69375 | 0.690972222 | 0 | NNPs |
| BTG2 | 0 | 0.315742702735001 | 0.255555556 | 0.055 | 0 | NNPs |
| TCF7L2 | 0 | 0.314558215276965 | 0.261111111 | 0.073 | 0 | NNPs |
| MIR217HG | 0 | 0.314242282939917 | 0.309722222 | 0.077777778 | 0 | NNPs |
| TRMT112 | 0 | 0.311902258915696 | 0.640277778 | 0.492361111 | 0 | NNPs |
| RPL35A | 0 | 0.311457443156481 | 1 | 1 | 0 | NNPs |
| HNRNPA1P48 | 0 | 0.31060724638844 | 0.408333333 | 0.161805556 | 0 | NNPs |
| MPST | 0 | 0.306541575016972 | 0.467361111 | 00:34 | 0 | NNPs |
| EEF1A1 | 0 | 0.306015474535747 | 1 | 1 | 0 | NNPs |
| CNN3 | 0 | 0.305591694546559 | 0.671527778 | 0.565277778 | 0 | NNPs |
| RPL32 | 0 | 0.305199709131742 | 1 | 1 | 0 | NNPs |
| RPS10 | 0 | 0.304833485005921 | 0.693055556 | 0.068055556 | 0 | NNPs |
| H2AFY | 0 | 0.304119519588285 | 0.677777778 | 0.592361111 | 0 | NNPs |
| HNRNPA3 | 0 | 0.303213899608417 | 0.06875 | 0.646527778 | 0 | NNPs |
| MSN | 0 | 0.302068824373141 | 0.313888889 | 0.088888889 | 0 | NNPs |
| MRPL11 | 0 | 0.301508132529355 | 0.476388889 | 0.241666667 | 0 | NNPs |
| GLO1 | 0 | 0.300956235773213 | 0.570833333 | 0.36875 | 0 | NNPs |
| CMTM6 | 0 | 0.300011331982335 | 0.379166667 | 0.1375 | 0 | NNPs |
| PPIA | 0 | 0.299916858779331 | 0.693055556 | 0.684027778 | 0 | NNPs |
| LAPTM4B | 0 | 0.298357201221851 | 0.585416667 | 0.401388889 | 0 | NNPs |
| SCAF11 | 0 | 0.297654432760224 | 0.54375 | 0.340972222 | 0 | NNPs |
| SLC16A9 | 0 | 0.297439211796703 | 00:47 | 0.116666667 | 0 | NNPs |
| LINC01833 | 0 | 0.295502760918313 | 00:45 | 0.098611111 | 0 | NNPs |
| B2M | 0 | 0.294798570369027 | 0.278472222 | 0.077777778 | 0 | NNPs |
| MOB1A | 0 | 0.29472846606291 | 0.39375 | 00:23 | 0 | NNPs |
| LMO4 | 0 | 0.2936729923818 | 0.534027778 | 0.350694444 | 0 | NNPs |
| EZR | 0 | 0.293160928305055 | 0.309722222 | 0.092361111 | 0 | NNPs |
| FLNA | 0 | 0.292300173846965 | 0.3 | 0.073611111 | 0 | NNPs |
| FOXG1 | 0 | 0.291957952531438 | 0.556944444 | 0.375694444 | 0 | NNPs |
| C8orf59 | 0 | 0.290976096737746 | 0.654861111 | 0.53125 | 0 | NNPs |
| LMO1 | 0 | 0.290504406470533 | 0.219444444 | 0.063 | 0 | NNPs |
| SERBP1 | 0 | 0.290204989921614 | 0.688888889 | 0.652083333 | 0 | NNPs |
| PAIP2 | 0 | 0.288265322581779 | 0.66875 | 0.586805556 | 0 | NNPs |
| FSTL1 | 0 | 0.288209851856258 | 0.238888889 | 0.071 | 0 | NNPs |
| HNRNPA2B1 | 0 | 0.286577893536457 | 0.691666667 | 0.067361111 | 0 | NNPs |
| SNRPA | 0 | 0.286469249012574 | 0.359027778 | 0.134722222 | 0 | NNPs |
| EMX1 | 0 | 0.286413221265279 | 0.20625 | 0.031 | 0 | NNPs |
| ARL4D | 0 | 0.285986816749387 | 0.265972222 | 0.104861111 | 0 | NNPs |
| SERPING1 | 0 | 0.285955398124898 | 0.29375 | 0.075694444 | 0 | NNPs |
| LINC01315 | 0 | 0.285863013881416 | 0.419444444 | 0.2125 | 0 | NNPs |
| TPBG | 0 | 0.284637494333276 | 0.222916667 | 0.069 | 0 | NNPs |
| SMARCC1 | 0 | 0.284121955516687 | 0.646527778 | 0.538888889 | 0 | NNPs |
| COL4A5 | 0 | 0.28344241347265 | 0.241666667 | 0.053 | 0 | NNPs |
| TAF10 | 0 | 0.283018213382005 | 0.604861111 | 0.429861111 | 0 | NNPs |
| FOXP4 | 0 | 0.282135647953428 | 0.279166667 | 0.088 | 0 | NNPs |
| TLE1 | 0 | 0.281079540866439 | 0.400694444 | 0.196527778 | 0 | NNPs |
| TGIF1 | 0 | 0.279462597816738 | 0.266666667 | 0.082 | 0 | NNPs |
| UBA52 | 0 | 0.279154916090333 | 1 | 0.690972222 | 0 | NNPs |
| RPL26 | 0 | 0.278147862907125 | 1 | 0.69375 | 0 | NNPs |
| UFC1 | 0 | 0.277996192157127 | 0.630555556 | 0.490277778 | 0 | NNPs |
| CRABP2 | 0 | 0.277705916820195 | 0.304861111 | 0.119444444 | 0 | NNPs |
| TCF4 | 0 | 0.277696337640683 | 0.477777778 | 0.274305556 | 0 | NNPs |
| C1orf53 | 0 | 0.277010111599003 | 0.427083333 | 0.238888889 | 0 | NNPs |
| KIF19 | 0 | 0.276951397411776 | 0.203472222 | 0.023 | 0 | NNPs |
| ACAA2 | 0 | 0.27654684567183 | 0.371527778 | 0.146527778 | 0 | NNPs |
| TSPAN3 | 0 | 0.276183449371319 | 0.625694444 | 0.495138889 | 0 | NNPs |
| CST3 | 0 | 0.276123233489658 | 0.065972222 | 0.519444444 | 0 | NNPs |
| SORCS2 | 0 | 0.275796636438274 | 0.269444444 | 0.096 | 0 | NNPs |
| ENO1 | 0 | 0.274763891029761 | 0.051388889 | 0.321527778 | 0 | NNPs |
| RPL28 | 0 | 0.273870173491611 | 1 | 1 | 0 | NNPs |
| YBX1 | 0 | 0.273167558368715 | 1 | 0.69375 | 0 | NNPs |
| COL11A1 | 0 | 0.271575385805176 | 0.23125 | 0.075 | 0 | NNPs |
| Sep-04 | 0 | 0.271446815637579 | 0.175694444 | 0.064 | 0 | NNPs |
| RPS15A | 0 | 0.270593556315211 | 1 | 1 | 0 | NNPs |
| SLC6A15 | 0 | 0.269860929541365 | 0.259027778 | 0.102083333 | 0 | NNPs |
| CD99 | 0 | 0.26976415103139 | 0.290277778 | 0.099 | 0 | NNPs |
| NCALD | 0 | 0.267777188179619 | 0.390277778 | 00:26 | 0 | NNPs |
| PBXIP1 | 0 | 0.266839930332691 | 00:37 | 0.084 | 0 | NNPs |
| KHDRBS1 | 0 | 0.266429983691022 | 0.06875 | 0.647916667 | 0 | NNPs |
| SSR2 | 0 | 0.265255150074605 | 0.679861111 | 0.615972222 | 0 | NNPs |
| RSL1D1 | 0 | 0.264335835526599 | 0.054861111 | 0.365277778 | 0 | NNPs |
| RGS20 | 0 | 0.263936219351531 | 0.270138889 | 0.089 | 0 | NNPs |
| NELL2 | 0 | 0.263507550411177 | 0.4 | 0.200694444 | 0 | NNPs |
| DNALI1 | 0 | 0.263100706712142 | 0.501388889 | 0.290972222 | 0 | NNPs |
| CD63 | 0 | 0.26293679450486 | 0.68125 | 0.633333333 | 0 | NNPs |
| DTWD1 | 0 | 0.262764887483145 | 0.382638889 | 0.179166667 | 0 | NNPs |
| NME4 | 0 | 0.262428455174787 | 0.4625 | 0.264583333 | 0 | NNPs |
| ATP6V0E1 | 0 | 0.262411060443657 | 0.552777778 | 0.374305556 | 0 | NNPs |
| RPL24 | 0 | 0.26137207499816 | 1 | 1 | 0 | NNPs |

| CASC15 | 0 | 0.261257819431671 | 0.343055556 | 0.141666667 | 0 | NNPs |
| --- | --- | --- | --- | --- | --- | --- |
| RPL9 | 0 | 0.261178528947235 | 1 | 0.69375 | 0 | NNPs |
| ZEB2 | 0 | 0.259159998336124 | 0.307638889 | 0.114583333 | 0 | NNPs |
| RFXANK | 0 | 0.258889575000061 | 0.31875 | 0.108333333 | 0 | NNPs |
| RPL15 | 0 | 0.258707146703416 | 1 | 1 | 0 | NNPs |
| RPS29 | 0 | 0.258587253925622 | 1 | 0.690972222 | 0 | NNPs |
| NPTX2 | 0 | 0.258094385579837 | 0.176388889 | 0.051 | 0 | NNPs |
| RPL6 | 0 | 0.257368106266344 | 1 | 1 | 0 | NNPs |
| TBCA | 0 | 0.256956529976796 | 0.690972222 | 0.664583333 | 0 | NNPs |
| UBXN1 | 0 | 0.256584417471436 | 0.672916667 | 0.595833333 | 0 | NNPs |
| E2F1 | 0 | 0.255553284787947 | 0.227777778 | 0.051 | 0 | NNPs |
| IMPDH2 | 0 | 0.25550985627993 | 0.483333333 | 0.278472222 | 0 | NNPs |
| SELENOH | 0 | 0.254981723428226 | 0.067361111 | 0.600694444 | 0 | NNPs |
| SVIL | 0 | 0.253642330290077 | 00:51 | 0.168055556 | 0 | NNPs |
| GDI2 | 0 | 0.252844390872062 | 0.671527778 | 0.604861111 | 0 | NNPs |
| BAZ1A | 0 | 0.252536393360898 | 0.449305556 | 0.247916667 | 0 | NNPs |
| HIST1H1C | 0 | 0.252518626877015 | 0.315277778 | 0.127083333 | 0 | NNPs |
| PSD3 | 0 | 0.251890270038201 | 0.413888889 | 0.224305556 | 0 | NNPs |
| ANTXR1 | 0 | 0.251053976565687 | 00:35 | 00:08 | 0 | NNPs |
| ELAVL1 | 0 | 0.250749340472892 | 0.5125 | 0.320833333 | 0 | NNPs |
| CENPH | 0 | 0.250735257907385 | 0.2875 | 0.101388889 | 0 | NNPs |
| UTRN | 0 | 0.250295649713968 | 00:35 | 00:09 | 0 | NNPs |
| PLXNA1 | 0 | 0.250050077806517 | 0.421527778 | 0.232638889 | 0 | NNPs |
| SNRPD3 | 2.99326972754386e-299 | 0.254606093734642 | 0.626388889 | 0.474305556 | 6.21492593529931e-295 | NNPs |
| LAPTM4A | 7.2077985434606e-290 | 0.266535031248947 | 0.56875 | 0.404861111 | 1.49655521157872e-285 | NNPs |
| PKM | 5.85774360116575e-269 | 0.258204064477027 | 0.591666667 | 0.431944444 | 1.21624330391005e-264 | NNPs |
| NCBP2-AS2 | 8.35666203706436e-268 | 0.252917912247241 | 0.483333333 | 00:45 | 1.73509373875567e-263 | NNPs |
| CALR | 2.56959952387276e-263 | 0.25684580942401 | 0.522222222 | 0.345833333 | 5.33525949141701e-259 | NNPs |
| RALA | 4.69238115425941e-233 | 0.269442917353051 | 0.560416667 | 0.432638889 | 9.74279099058881e-229 | NNPs |
| TMSB4X | 2.64246008329848e-187 | 0.316419043855889 | 0.69375 | 0.69375 | 5.48653987095263e-183 | NNPs |
| ID4 | 1.9418848421934e-138 | 0.40318761544761 | 0.545138889 | 0.044444444 | 4.03193549784616e-134 | NNPs |
| TPM1 | 7.99502779206043e-128 | 0.35372094735743 | 0.516666667 | 0.401388889 | 1.66000762046551e-123 | NNPs |
| SMS | 1.33023032159722e-96 | 0.289708848684785 | 0.660416667 | 0.645138889 | 2.7619572167323e-92 | NNPs |
| GNRH1 | 0 | 4.56070378711341 | 0.535416667 | 0.111805556 | 0 | GnRHGABA |
| DLX6-AS1 | 0 | 1.4800223832291 | 0.442361111 | 0.007 | 0 | GnRHGABA |
| VCAN | 0 | 1.44350170032541 | 0.63125 | 0.355555556 | 0 | GnRHGABA |
| DLX5 | 0 | 1.38754701583214 | 0.572222222 | 0.014 | 0 | GnRHGABA |
| DLX1 | 0 | 1.22367022699675 | 0.484722222 | 0.037 | 0 | GnRHGABA |
| GAD2 | 0 | 1.12432365065994 | 0.047916667 | 0.013 | 0 | GnRHGABA |
| RASD1 | 0 | 1.0406141816454 | 0.374305556 | 0.045 | 0 | GnRHGABA |
| SCGN | 0 | 1.03380819617176 | 0.241666667 | 0.005 | 0 | GnRHGABA |
| ARX | 0 | 0.999331175867213 | 0.467361111 | 0.043 | 0 | GnRHGABA |
| SIX3 | 0 | 0.970951209268559 | 0.617361111 | 0.402083333 | 0 | GnRHGABA |
| DLX2 | 0 | 0.958030659052604 | 0.452777778 | 0.038 | 0 | GnRHGABA |
| TAC1 | 0 | 0.892038673730841 | 0.254166667 | 0.074 | 0 | GnRHGABA |
| DLX6 | 0 | 0.883605930613844 | 0.425694444 | 0.007 | 0 | GnRHGABA |
| GRIA2 | 0 | 0.860199184843782 | 0.480555556 | 0.120138889 | 0 | GnRHGABA |
| ZFHX3 | 0 | 0.821307456505722 | 0.654861111 | 0.497222222 | 0 | GnRHGABA |
| MEF2C | 0 | 0.807996179104729 | 0.313194444 | 0.053 | 0 | GnRHGABA |
| MEST | 0 | 0.778656671551824 | 0.66875 | 0.05625 | 0 | GnRHGABA |
| RALYL | 0 | 0.739401643559462 | 0.3875 | 0.044 | 0 | GnRHGABA |
| TLE4 | 0 | 0.735993512954672 | 0.659027778 | 0.6125 | 0 | GnRHGABA |
| ISL1 | 0 | 0.723122183620473 | 0.358333333 | 0.087 | 0 | GnRHGABA |
| PRKACB | 0 | 0.709632979565152 | 0.520138889 | 0.25625 | 0 | GnRHGABA |
| GRID2 | 0 | 0.701751086131507 | 0.3625 | 00:15 | 0 | GnRHGABA |
| NEFL | 0 | 0.691286352647684 | 0.634027778 | 0.505555556 | 0 | GnRHGABA |
| ECEL1 | 0 | 0.633169679621936 | 0.361805556 | 0.049 | 0 | GnRHGABA |
| RND3 | 0 | 0.631139788391915 | 0.436805556 | 0.158333333 | 0 | GnRHGABA |
| FGF3 | 0 | 0.609775799499387 | 0.193055556 | 0.002 | 0 | GnRHGABA |
| FGF19 | 0 | 0.609449031612205 | 0.217361111 | 0.013 | 0 | GnRHGABA |
| FOXG1 | 0 | 0.591093710135611 | 0.607638889 | 0.402083333 | 0 | GnRHGABA |
| EEF1A2 | 0 | 0.553081971626135 | 0.44375 | 0.196527778 | 0 | GnRHGABA |
| LY6H | 0 | 0.520623508604069 | 0.394444444 | 0.124305556 | 0 | GnRHGABA |
| MAP1B | 0 | 0.493500868121411 | 1 | 0.692361111 | 0 | GnRHGABA |
| SEMA3C | 0 | 0.487622365508498 | 0.236805556 | 0.004 | 0 | GnRHGABA |
| ALCAM | 0 | 0.466236066831384 | 0.259722222 | 0.097 | 0 | GnRHGABA |
| SLC32A1 | 0 | 0.434216622851005 | 0.260416667 | 0.005 | 0 | GnRHGABA |
| MIR7-3HG | 0 | 0.420124276146003 | 0.340972222 | 00:17 | 0 | GnRHGABA |
| C11orf96 | 0 | 0.410574579545999 | 0.228472222 | 0.038 | 0 | GnRHGABA |
| RBFOX1 | 0 | 0.386194089841512 | 0.203472222 | 0.009 | 0 | GnRHGABA |
| SYBU | 0 | 0.379005337595046 | 0.267361111 | 0.094 | 0 | GnRHGABA |
| NRXN3 | 0 | 0.378970296870586 | 0.3 | 0.086805556 | 0 | GnRHGABA |
| RAB31 | 0 | 0.377787264542742 | 0.290277778 | 0.084722222 | 0 | GnRHGABA |
| SPOCK1 | 0 | 0.345443103057986 | 0.204861111 | 0.036 | 0 | GnRHGABA |
| NTRK1 | 0 | 0.341614676689371 | 0.195138889 | 0.024 | 0 | GnRHGABA |
| UNC5D | 0 | 0.328013128983795 | 0.196527778 | 0.028 | 0 | GnRHGABA |
| PRKCB | 0 | 0.292236631045884 | 0.179166667 | 0.039 | 0 | GnRHGABA |
| RGS2 | 1.3080430663977e-303 | 0.314628911712808 | 0.225694444 | 0.087 | 2.71588981876154e-299 | GnRHGABA |
| PAFAH1B3 | 1.64647353349974e-300 | 0.404547710825644 | 0.684722222 | 0.663194444 | 3.41857299760551e-296 | GnRHGABA |
| FEZ1 | 1.65363298231484e-281 | 0.468517196182567 | 0.613888889 | 0.049305556 | 3.43343816118031e-277 | GnRHGABA |
| RIPOR2 | 1.788437423675e-273 | 0.327721936052691 | 00:39 | 0.086805556 | 3.71333262277639e-269 | GnRHGABA |
| ETFB | 6.05266954163977e-273 | 0.489859882659288 | 0.545138889 | 0.365277778 | 1.25671577693067e-268 | GnRHGABA |
| FAT3 | 3.43947882090591e-272 | 0.367628541462087 | 0.253472222 | 0.08125 | 7.14138987584694e-268 | GnRHGABA |
| POU3F2 | 4.79678900163702e-269 | 0.538292109613119 | 0.397222222 | 0.188888889 | 9.95957300409895e-265 | GnRHGABA |
| AC007938.3 | 1.44868115937762e-268 | 0.286128824120381 | 0.220138889 | 0.089 | 3.00789669121574e-264 | GnRHGABA |
| FUT8 | 2.23582082009686e-268 | 0.3295544102702 | 0.289583333 | 0.105555556 | 4.64223476876712e-264 | GnRHGABA |
| BCL11A | 6.54518164872015e-264 | 0.47147249679676 | 00:57 | 0.190972222 | 1.35897606572376e-259 | GnRHGABA |
| LINC01833 | 4.15800784946904e-259 | 0.43980617740554 | 0.320833333 | 0.134027778 | 8.63327169785256e-255 | GnRHGABA |
| LINC00461 | 1.12438275277899e-257 | 0.44018330631997 | 0.384027778 | 00:26 | 2.33455590959503e-253 | GnRHGABA |
| NSG1 | 1.79592650916839e-256 | 0.456541870647095 | 0.611805556 | 0.434027778 | 3.72888221098634e-252 | GnRHGABA |
| GNAO1 | 4.62449177314893e-252 | 0.375183835565142 | 0.379861111 | 00:24 | 9.60183226858912e-248 | GnRHGABA |
| SYT1 | 6.46255539965304e-244 | 0.565327939706687 | 0.669444444 | 0.617361111 | 1.34182037762996e-239 | GnRHGABA |
| FGF14 | 2.23659923005804e-243 | 0.353680702176123 | 0.276388889 | 0.098611111 | 4.64385098136951e-239 | GnRHGABA |
| HCRT | 4.51037149149498e-241 | 0.396945291113331 | 0.272222222 | 00:14 | 9.36488432779103e-237 | GnRHGABA |
| AKR1C1 | 1.22282162886724e-230 | 0.329017550048735 | 0.355555556 | 0.15 | 2.53894454801704e-226 | GnRHGABA |
| DPF1 | 1.81549960694814e-227 | 0.30948466863436 | 0.31875 | 0.127083333 | 3.76952183390642e-223 | GnRHGABA |
| EFNB2 | 1.48730267316606e-221 | 0.328661730583408 | 0.286111111 | 0.113888889 | 3.08808654029469e-217 | GnRHGABA |
| DUSP4 | 1.53520603717214e-217 | 0.351980096828124 | 0.379861111 | 0.182638889 | 3.18754829498051e-213 | GnRHGABA |
| LHFPL6 | 1.58066399061017e-217 | 0.319021400481383 | 0.250694444 | 0.092361111 | 3.28193264370389e-213 | GnRHGABA |
| RAPGEF2 | 2.90258654496519e-217 | 0.349483753833844 | 0.300694444 | 0.127777778 | 6.02664044331123e-213 | GnRHGABA |

| DSEL | 4.04349363318371e-209 | 0.564198842479449 | 0.249305556 | 0.097916667 | 8.39550583057935e-205 | GnRHGABA |
| --- | --- | --- | --- | --- | --- | --- |
| SLCO3A1 | 1.7618981758908e-208 | 0.425259207793354 | 0.379861111 | 0.195138889 | 3.65822918260206e-204 | GnRHGABA |
| NOS1AP | 1.43541938447847e-204 | 0.334668952369131 | 0.31875 | 0.144444444 | 2.98036126799265e-200 | GnRHGABA |
| CXXC4 | 4.56067022767628e-198 | 0.398213539176696 | 0.494444444 | 0.309722222 | 9.46931959372427e-194 | GnRHGABA |
| PPP1R1A | 1.52895753953214e-190 | 0.410436177519937 | 0.538888889 | 0.391666667 | 3.17457453933057e-186 | GnRHGABA |
| SETBP1 | 2.85744106723063e-190 | 0.383528960378574 | 0.396527778 | 0.220833333 | 5.93290488789095e-186 | GnRHGABA |
| AES | 1.19644287730541e-188 | 0.36952905512446 | 0.552083333 | 0.415277778 | 2.48417434614923e-184 | GnRHGABA |
| PBX3 | 2.05896260794209e-186 | 0.494572383844401 | 0.484722222 | 0.334027778 | 4.27502406287017e-182 | GnRHGABA |
| HIP1R | 1.81387690722763e-180 | 0.265307266839301 | 0.2875 | 0.120833333 | 3.76615262247674e-176 | GnRHGABA |
| ARG2 | 2.07608428151373e-175 | 0.320425450898865 | 0.376388889 | 0.195138889 | 4.31057379370696e-171 | GnRHGABA |
| SCG3 | 5.25941340017454e-174 | 0.34856123247038 | 0.653472222 | 0.547916667 | 1.09201200427824e-169 | GnRHGABA |
| NRXN2 | 7.0177337479425e-171 | 0.261412129245148 | 0.289583333 | 0.127083333 | 1.4570920580853e-166 | GnRHGABA |
| PPP1R14B | 3.71223442422635e-160 | 0.303774629916909 | 0.675 | 0.065972222 | 7.70771233502116e-156 | GnRHGABA |
| MAPT | 2.17115898752538e-159 | 0.353817866408995 | 0.559027778 | 0.384027778 | 4.50797740579894e-155 | GnRHGABA |
| CSRNP3 | 2.9394529338223e-158 | 0.379613422431109 | 0.560416667 | 0.435416667 | 6.10318612649524e-154 | GnRHGABA |
| PPM1K | 1.34441385558095e-153 | 0.340768859822385 | 0.332638889 | 0.172916667 | 2.79140648834272e-149 | GnRHGABA |
| GDAP1L1 | 2.4528260020669e-152 | 0.334819855643754 | 0.572916667 | 0.418055556 | 5.0928026280915e-148 | GnRHGABA |
| VGF | 1.103724016311e-149 | 0.268702567013296 | 0.252083333 | 0.110416667 | 2.29166217506653e-145 | GnRHGABA |
| LINC01551 | 1.40125328498563e-147 | 0.325187209477765 | 0.629166667 | 0.429861111 | 2.90942219561566e-143 | GnRHGABA |
| LBH | 2.34275701444397e-146 | 0.332823719089772 | 0.575694444 | 0.452777778 | 4.86426638909002e-142 | GnRHGABA |
| ID4 | 4.33568081621513e-145 | 0.369962521070891 | 0.580555556 | 0.045833333 | 9.00217407870748e-141 | GnRHGABA |
| C1QTNF4 | 4.60945168850703e-143 | 0.290513895479087 | 0.39375 | 0.220833333 | 9.57060454084715e-139 | GnRHGABA |
| PCDH7 | 6.30549018942112e-143 | 0.275832541216069 | 0.296527778 | 0.142361111 | 1.30920892802951e-138 | GnRHGABA |
| SPINT2 | 5.2774459474789e-141 | 0.251391227806806 | 0.242361111 | 0.108333333 | 1.09575610207504e-136 | GnRHGABA |
| MEIS3 | 7.01913347731725e-133 | 0.290853542734767 | 0.472916667 | 0.321527778 | 1.45738268389538e-128 | GnRHGABA |
| ACOT7 | 2.51996999140901e-132 | 0.292720754368354 | 00:55 | 0.230555556 | 5.23221369316254e-128 | GnRHGABA |
| RAB3A | 6.98767966051135e-131 | 0.276756586474193 | 0.435416667 | 0.258333333 | 1.45085192791197e-126 | GnRHGABA |
| PPP2R2B | 1.176444842898e-130 | 0.622051779743916 | 0.511111111 | 00:57 | 2.44265242730911e-126 | GnRHGABA |
| TMEM167A | 4.7565540462116e-130 | 0.30953875426673 | 0.608333333 | 0.536111111 | 9.87603316614915e-126 | GnRHGABA |
| CELF4 | 3.39187715933324e-127 | 0.366625381473917 | 0.529861111 | 0.384722222 | 7.04255454592361e-123 | GnRHGABA |
| APC | 1.01795179455405e-122 | 0.316805293824451 | 0.518055556 | 0.39375 | 2.11357331103258e-118 | GnRHGABA |
| KIDINS220 | 6.8283750129022e-122 | 0.264078885964848 | 0.653472222 | 0.595833333 | 1.41777550392888e-117 | GnRHGABA |
| MIAT | 1.74077476050458e-120 | 0.298953580275137 | 0.56875 | 0.467361111 | 3.61437063523567e-116 | GnRHGABA |
| UCHL1 | 2.31704216379066e-116 | 0.252963081007975 | 0.684722222 | 0.675 | 4.81087464467855e-112 | GnRHGABA |
| FAM89B | 6.36700154061927e-116 | 0.262165376389459 | 0.615972222 | 0.05625 | 1.32198052987878e-111 | GnRHGABA |
| PCSK1N | 5.11323403413877e-114 | 0.297713245701791 | 0.648611111 | 0.51875 | 1.06166078250823e-109 | GnRHGABA |
| LRRN3 | 1.61362611793851e-103 | 0.310206997699812 | 0.433333333 | 0.28125 | 3.35037190867572e-99 | GnRHGABA |
| ATP6V0E2 | 5.79756962195995e-101 | 0.267297332567027 | 0.522222222 | 0.403472222 | 1.20374938060754e-96 | GnRHGABA |
| GNAS | 3.81127817367584e-100 | 0.368144332498346 | 1 | 0.69375 | 7.91335687200315e-96 | GnRHGABA |
| TNRC6C | 6.64872568504044e-90 | 0.261175475402052 | 0.441666667 | 00:46 | 1.38047491398495e-85 | GnRHGABA |
| TTLL7 | 6.10830831075704e-86 | 0.270145659106354 | 0.427083333 | 0.315972222 | 1.26826805456248e-81 | GnRHGABA |
| TUBB2A | 9.48340646391179e-85 | 0.255880545025382 | 0.677083333 | 0.594444444 | 1.969039684102e-80 | GnRHGABA |
| CHD3 | 2.10953617057835e-84 | 0.255926162553044 | 0.525694444 | 0.43125 | 4.38002995097182e-80 | GnRHGABA |
| NUCB2 | 8.94424575230992e-84 | 0.257232609934152 | 0.496527778 | 0.392361111 | 1.85709374555211e-79 | GnRHGABA |
| PLEKHA5 | 3.5603899145588e-73 | 0.250661352396819 | 0.432638889 | 0.325 | 7.39243757959844e-69 | GnRHGABA |
| MEIS2 | 1.31737519363806e-58 | 0.75873097784322 | 0.40625 | 0.360416667 | 2.73526611455071e-54 | GnRHGABA |
| RBP1 | 1.36656143169118e-46 | 0.290884548562089 | 0.478472222 | 0.401388889 | 2.8373915006204e-42 | GnRHGABA |
| CENPF | 0 | 2.82907143329137 | 0.69375 | 0.102777778 | 0 | PNPs |
| HMGB2 | 0 | 2.65350500743169 | 1 | 0.2 | 0 | PNPs |
| TOP2A | 0 | 2.64472255170814 | 0.683333333 | 00:06 | 0 | PNPs |
| NUSAP1 | 0 | 2.30916078488172 | 0.692361111 | 00:04 | 0 | PNPs |
| UBE2C | 0 | 2.2745137454586 | 0.671527778 | 0.076 | 0 | PNPs |
| TPX2 | 0 | 2.12813523073538 | 0.686805556 | 0.046 | 0 | PNPs |
| PTTG1 | 0 | 2.11516897643098 | 0.686111111 | 0.127083333 | 0 | PNPs |
| CCNB1 | 0 | 1.98277686774226 | 0.667361111 | 0.070833333 | 0 | PNPs |
| DLGAP5 | 0 | 1.92950975105974 | 0.663888889 | 00:03 | 0 | PNPs |
| ASPM | 0 | 1.81713677772153 | 0.065972222 | 0.029 | 0 | PNPs |
| CCNB2 | 0 | 1.77495431737538 | 0.067361111 | 0.101388889 | 0 | PNPs |
| MKI67 | 0 | 1.77354526675252 | 0.679166667 | 0.056 | 0 | PNPs |
| CKAP2 | 0 | 1.69591569650189 | 0.689583333 | 0.15 | 0 | PNPs |
| BIRC5 | 0 | 1.65348131342865 | 0.683333333 | 0.097 | 0 | PNPs |
| CKS2 | 0 | 1.64732688110319 | 0.688194444 | 00:23 | 0 | PNPs |
| HMGN2 | 0 | 1.60856115408003 | 1 | 0.632638889 | 0 | PNPs |
| ANP32E | 0 | 1.5414195772936 | 0.69375 | 00:48 | 0 | PNPs |
| MAD2L1 | 0 | 1.54084413214852 | 0.690277778 | 0.087 | 0 | PNPs |
| NUF2 | 0 | 1.53244935318945 | 0.6625 | 0.025 | 0 | PNPs |
| CDK1 | 0 | 1.53011721864372 | 0.611805556 | 0.014 | 0 | PNPs |
| TUBA1B | 0 | 1.52973144145689 | 1 | 0.644444444 | 0 | PNPs |
| CKS1B | 0 | 1.52395869642669 | 0.684722222 | 0.147222222 | 0 | PNPs |
| HIST1H1B | 0 | 1.51725795130004 | 0.413888889 | 0.035 | 0 | PNPs |
| UBE2S | 0 | 1.50381039202571 | 0.692361111 | 0.595138889 | 0 | PNPs |
| HMMR | 0 | 1.48481145771378 | 0.065277778 | 0.071 | 0 | PNPs |
| TUBB4B | 0 | 1.45295653907566 | 0.688194444 | 0.413888889 | 0 | PNPs |
| SMC4 | 0 | 1.44433487499175 | 0.68125 | 0.128472222 | 0 | PNPs |
| KPNA2 | 0 | 1.43545590521083 | 0.686111111 | 0.433333333 | 0 | PNPs |
| MIS18BP1 | 0 | 1.36750805844983 | 0.672222222 | 0.079166667 | 0 | PNPs |
| CDC20 | 0 | 1.35868554734739 | 0.061111111 | 00:04 | 0 | PNPs |
| ECT2 | 0 | 1.28671437594124 | 0.656944444 | 0.048 | 0 | PNPs |
| CDKN3 | 0 | 1.28529276573303 | 0.641666667 | 0.039 | 0 | PNPs |
| GTSE1 | 0 | 1.26730004137705 | 0.652083333 | 0.018 | 0 | PNPs |
| H2AFX | 0 | 1.25700380546562 | 0.670833333 | 0.084027778 | 0 | PNPs |
| H2AFZ | 0 | 1.24252510556138 | 1 | 0.685416667 | 0 | PNPs |
| SOX2 | 0 | 1.24095967423491 | 0.677777778 | 00:21 | 0 | PNPs |
| CENPU | 0 | 1.23008427597837 | 0.677083333 | 0.074305556 | 0 | PNPs |
| NUCKS1 | 0 | 1.22677273694795 | 0.69375 | 0.658333333 | 0 | PNPs |
| CENPE | 0 | 1.20874530292437 | 0.605555556 | 0.048 | 0 | PNPs |
| UBE2T | 0 | 1.19463475745557 | 0.674305556 | 0.161805556 | 0 | PNPs |
| CDCA8 | 0 | 1.19135732535606 | 0.625694444 | 0.022 | 0 | PNPs |
| PCLAF | 0 | 1.17656405123378 | 0.61875 | 0.065 | 0 | PNPs |
| TMPO | 0 | 1.17443443505569 | 0.692361111 | 0.547222222 | 0 | PNPs |
| SGO2 | 0 | 1.16858381660775 | 0.061805556 | 0.051 | 0 | PNPs |
| ARL6IP1 | 0 | 1.16508210507816 | 0.6625 | 0.456944444 | 0 | PNPs |
| CCNA2 | 0 | 1.15704437259402 | 0.632638889 | 0.025 | 0 | PNPs |
| TYMS | 0 | 1.14518664129776 | 0.620138889 | 00:09 | 0 | PNPs |
| TUBA1C | 0 | 1.13199550422635 | 0.453472222 | 0.076 | 0 | PNPs |
| NDC80 | 0 | 1.12015391094246 | 0.629861111 | 00:01 | 0 | PNPs |
| AURKA | 0 | 1.11733140867351 | 0.566666667 | 0.031 | 0 | PNPs |
| VIM | 0 | 1.10621258055479 | 1 | 0.561805556 | 0 | PNPs |
| NCAPG | 0 | 1.10509511750246 | 0.644444444 | 0.037 | 0 | PNPs |
| CDCA3 | 0 | 1.09943472836134 | 0.596527778 | 0.026 | 0 | PNPs |

| NEK2 | 0 | 1.05797784530455 | 0.563888889 | 0.013 | 0 | PNPs |
| --- | --- | --- | --- | --- | --- | --- |
| CENPA | 0 | 1.05035233847091 | 0.056944444 | 0.013 | 0 | PNPs |
| PBK | 0 | 1.00767980812526 | 0.619444444 | 0.014 | 0 | PNPs |
| DTYMK | 0 | 0.9999229929938 | 0.682638889 | 0.227777778 | 0 | PNPs |
| BUB3 | 0 | 0.973521635376333 | 0.664583333 | 0.245833333 | 0 | PNPs |
| ASCL1 | 0 | 0.969333142629439 | 0.561111111 | 0.084027778 | 0 | PNPs |
| KIF20B | 0 | 0.964928760513067 | 0.615277778 | 0.098 | 0 | PNPs |
| BUB1 | 0 | 0.964353244532816 | 0.597916667 | 0.015 | 0 | PNPs |
| TTK | 0 | 0.962295471356694 | 0.584027778 | 00:01 | 0 | PNPs |
| DEK | 0 | 0.956220874592533 | 0.692361111 | 0.572222222 | 0 | PNPs |
| SPC25 | 0 | 0.955101671465152 | 0.609027778 | 0.019 | 0 | PNPs |
| ANP32B | 0 | 0.948971260477077 | 0.692361111 | 0.453472222 | 0 | PNPs |
| H2AFV | 0 | 0.946623032393991 | 0.69375 | 0.532638889 | 0 | PNPs |
| PIMREG | 0 | 0.945446609597024 | 0.608333333 | 0.032 | 0 | PNPs |
| KIF11 | 0 | 0.942402642132509 | 0.061111111 | 0.018 | 0 | PNPs |
| HMGB1 | 0 | 0.935187011800335 | 1 | 0.693055556 | 0 | PNPs |
| LMNB1 | 0 | 0.928571266578353 | 0.681944444 | 0.279166667 | 0 | PNPs |
| SGO1 | 0 | 0.914096090793104 | 0.59375 | 0.024 | 0 | PNPs |
| DLK1 | 0 | 0.895422189944907 | 0.629166667 | 0.323611111 | 0 | PNPs |
| PLK1 | 0 | 0.893746890771601 | 0.536111111 | 0.018 | 0 | PNPs |
| HMGN3 | 0 | 0.886122459163611 | 0.690972222 | 0.484722222 | 0 | PNPs |
| SMC2 | 0 | 0.882442686465643 | 0.665972222 | 0.197916667 | 0 | PNPs |
| KIF4A | 0 | 0.88102737251103 | 0.059722222 | 0.023 | 0 | PNPs |
| PRR11 | 0 | 0.880044648684123 | 0.604861111 | 0.077 | 0 | PNPs |
| BUB1B | 0 | 0.87911542865985 | 0.586111111 | 0.016 | 0 | PNPs |
| SNRPB | 0 | 0.871180529472732 | 0.688194444 | 0.426388889 | 0 | PNPs |
| CKB | 0 | 0.870988660817956 | 0.693055556 | 0.66875 | 0 | PNPs |
| RPA3 | 0 | 0.846499843613306 | 0.665277778 | 0.218055556 | 0 | PNPs |
| CSRP2 | 0 | 0.844228443836794 | 0.692361111 | 0.570833333 | 0 | PNPs |
| KNSTRN | 0 | 0.841687126706918 | 0.607638889 | 0.184722222 | 0 | PNPs |
| KIF2C | 0 | 0.841261893672684 | 0.56875 | 00:01 | 0 | PNPs |
| DBF4 | 0 | 0.838827332928444 | 0.615277778 | 0.116666667 | 0 | PNPs |
| GNG5 | 0 | 0.835266547434629 | 1 | 0.396527778 | 0 | PNPs |
| CEP55 | 0 | 0.827009321561063 | 0.531944444 | 0.005 | 0 | PNPs |
| KIF15 | 0 | 0.818613914610975 | 0.059027778 | 0.027 | 0 | PNPs |
| MZT1 | 0 | 0.811803560399899 | 0.679166667 | 0.357638889 | 0 | PNPs |
| CENPW | 0 | 0.811534182277086 | 0.660416667 | 0.197916667 | 0 | PNPs |
| KNL1 | 0 | 0.809712307866006 | 0.558333333 | 0.017 | 0 | PNPs |
| SFRP2 | 0 | 0.807405968718874 | 0.452777778 | 00:07 | 0 | PNPs |
| PRSS23 | 0 | 0.805402054650821 | 0.661111111 | 0.240277778 | 0 | PNPs |
| ZFP36L1 | 0 | 0.804141318472767 | 0.059027778 | 0.077777778 | 0 | PNPs |
| ORC6 | 0 | 0.803924312064901 | 0.622222222 | 0.127083333 | 0 | PNPs |
| NASP | 0 | 0.803308590379952 | 0.690277778 | 0.527083333 | 0 | PNPs |
| PSRC1 | 0 | 0.803287829453403 | 0.534027778 | 0.028 | 0 | PNPs |
| RTKN2 | 0 | 0.80127663771557 | 0.59375 | 0.045 | 0 | PNPs |
| ZWINT | 0 | 0.799852164605626 | 0.608333333 | 0.045 | 0 | PNPs |
| HIST1H1C | 0 | 0.795799033695076 | 0.448611111 | 0.156944444 | 0 | PNPs |
| DEPDC1 | 0 | 0.78552113685614 | 0.527083333 | 0.007 | 0 | PNPs |
| FBXO5 | 0 | 0.785226100271668 | 0.052083333 | 0.053 | 0 | PNPs |
| KIFC1 | 0 | 0.782138221936584 | 0.577777778 | 0.007 | 0 | PNPs |
| KIF18A | 0 | 0.777174422141132 | 0.536805556 | 0.017 | 0 | PNPs |
| HMGB3 | 0 | 0.776672709861964 | 0.692361111 | 0.581944444 | 0 | PNPs |
| TFDP2 | 0 | 0.775322344033523 | 0.684722222 | 00:57 | 0 | PNPs |
| CKAP2L | 0 | 0.769432073491631 | 0.554166667 | 0.006 | 0 | PNPs |
| C21orf58 | 0 | 0.766869451562429 | 0.579166667 | 0.092 | 0 | PNPs |
| CENPH | 0 | 0.763634241154587 | 0.636111111 | 0.120138889 | 0 | PNPs |
| RAN | 0 | 0.763463394129724 | 1 | 0.665972222 | 0 | PNPs |
| DLL1 | 0 | 0.760501989678971 | 0.061111111 | 00:24 | 0 | PNPs |
| ID4 | 0 | 0.759789403518737 | 0.066666667 | 0.457638889 | 0 | PNPs |
| KIF23 | 0 | 0.758676644505071 | 0.535416667 | 0.005 | 0 | PNPs |
| RANBP1 | 0 | 0.757918618395344 | 0.689583333 | 0.545833333 | 0 | PNPs |
| AURKB | 0 | 0.75665929434894 | 0.554166667 | 0.026 | 0 | PNPs |
| NMU | 0 | 0.746520436920584 | 0.622222222 | 0.189583333 | 0 | PNPs |
| CCDC34 | 0 | 0.744277156313339 | 0.663888889 | 0.300694444 | 0 | PNPs |
| NUDCD2 | 0 | 0.739277388755636 | 0.065277778 | 0.271527778 | 0 | PNPs |
| KIF14 | 0 | 0.736954195975325 | 0.510416667 | 0.011 | 0 | PNPs |
| HSP90B1 | 0 | 0.717978355031011 | 0.684722222 | 0.545833333 | 0 | PNPs |
| SIVA1 | 0 | 0.713483048029686 | 0.670833333 | 0.345833333 | 0 | PNPs |
| GMNN | 0 | 0.713340110618015 | 0.588888889 | 0.086 | 0 | PNPs |
| ATAD2 | 0 | 0.712226099158414 | 0.519444444 | 0.035 | 0 | PNPs |
| DEPDC1B | 0 | 0.711600021324227 | 0.559027778 | 0.036 | 0 | PNPs |
| CENPN | 0 | 0.709704006551483 | 0.588888889 | 0.077 | 0 | PNPs |
| LSM4 | 0 | 0.702579813418942 | 0.690277778 | 0.533333333 | 0 | PNPs |
| CENPK | 0 | 0.695335347484638 | 0.575694444 | 0.054 | 0 | PNPs |
| HNRNPA2B1 | 0 | 0.691204953350387 | 0.69375 | 0.677083333 | 0 | PNPs |
| TACC3 | 0 | 0.684561967768076 | 0.534027778 | 0.015 | 0 | PNPs |
| SNRPD1 | 0 | 0.684160576376722 | 0.690277778 | 0.592361111 | 0 | PNPs |
| HMGA2 | 0 | 0.682954551703565 | 0.575694444 | 0.078472222 | 0 | PNPs |
| HDGF | 0 | 0.681432392561676 | 0.683333333 | 0.425694444 | 0 | PNPs |
| LBR | 0 | 0.681311876773539 | 0.679166667 | 0.449305556 | 0 | PNPs |
| HJURP | 0 | 0.677776439725997 | 0.509722222 | 0.002 | 0 | PNPs |
| LSM5 | 0 | 0.667554310545773 | 0.691666667 | 0.534027778 | 0 | PNPs |
| RACGAP1 | 0 | 0.659278848532829 | 0.529861111 | 0.022 | 0 | PNPs |
| RRM2 | 0 | 0.657981325063032 | 0.520138889 | 0.022 | 0 | PNPs |
| HMGA1 | 0 | 0.653781227372118 | 0.670138889 | 0.429166667 | 0 | PNPs |
| H1FX | 0 | 0.652078681676703 | 0.69375 | 0.678472222 | 0 | PNPs |
| NUDT1 | 0 | 0.650066203947081 | 0.640277778 | 0.21875 | 0 | PNPs |
| SNRPE | 0 | 0.649470782881703 | 0.692361111 | 0.632638889 | 0 | PNPs |
| GPC3 | 0 | 0.644252129116927 | 0.572222222 | 0.115972222 | 0 | PNPs |
| RRM1 | 0 | 0.6435693075541 | 0.622916667 | 0.207638889 | 0 | PNPs |
| CDCA2 | 0 | 0.639580466306694 | 0.504166667 | 0.006 | 0 | PNPs |
| MDK | 0 | 0.633573660778828 | 0.69375 | 0.572916667 | 0 | PNPs |
| EEF1D | 0 | 0.632820592419265 | 0.692361111 | 0.535416667 | 0 | PNPs |
| HNRNPD | 0 | 0.627212958064512 | 0.69375 | 0.640972222 | 0 | PNPs |
| RAD21 | 0 | 0.62718845347561 | 0.684027778 | 0.495138889 | 0 | PNPs |
| TROAP | 0 | 0.626572305492959 | 0.049305556 | 0.035 | 0 | PNPs |
| MXD3 | 0 | 0.624709245997083 | 0.502083333 | 0.014 | 0 | PNPs |
| RAB13 | 0 | 0.623885030918211 | 0.635416667 | 0.181944444 | 0 | PNPs |
| PRC1 | 0 | 0.6238520326765 | 0.511111111 | 0.026 | 0 | PNPs |
| HELLS | 0 | 0.617867588683637 | 0.570138889 | 00:25 | 0 | PNPs |

| SPARC | 0 | 0.614589709065123 | 0.563888889 | 0.120833333 | 0 | PNPs |
| --- | --- | --- | --- | --- | --- | --- |
| SRSF3 | 0 | 0.610601119313047 | 0.692361111 | 0.600694444 | 0 | PNPs |
| USP1 | 0 | 0.608767993996468 | 0.602083333 | 0.183333333 | 0 | PNPs |
| ODC1 | 0 | 0.607082998455521 | 0.69375 | 0.645138889 | 0 | PNPs |
| FEZF2 | 0 | 0.605719348160285 | 0.570833333 | 0.186805556 | 0 | PNPs |
| TRIM59 | 0 | 0.604289232559208 | 0.505555556 | 0.058 | 0 | PNPs |
| GSTP1 | 0 | 0.602027765952984 | 0.693055556 | 0.056944444 | 0 | PNPs |
| CDC25B | 0 | 0.5967769154492 | 0.500694444 | 0.063 | 0 | PNPs |
| EXOSC8 | 0 | 0.594241287042469 | 0.65 | 00:36 | 0 | PNPs |
| DHFR | 0 | 0.592796358169815 | 0.55 | 0.109722222 | 0 | PNPs |
| RPS2 | 0 | 0.59192182438761 | 1 | 0.693055556 | 0 | PNPs |
| PHGDH | 0 | 0.585066701903172 | 0.054861111 | 0.1 | 0 | PNPs |
| MGME1 | 0 | 0.584611991759905 | 00:08 | 0.085 | 0 | PNPs |
| NCAPD2 | 0 | 0.58438030742506 | 0.51875 | 0.071 | 0 | PNPs |
| BARD1 | 0 | 0.584363604126017 | 0.511805556 | 0.069 | 0 | PNPs |
| TTYH1 | 0 | 0.583179623379303 | 0.592361111 | 0.232638889 | 0 | PNPs |
| ESCO2 | 0 | 0.579522888801143 | 0.470138889 | 0.005 | 0 | PNPs |
| CDC25C | 0 | 0.579084789368515 | 0.474305556 | 0.007 | 0 | PNPs |
| QKI | 0 | 0.577045456988217 | 0.630555556 | 0.265972222 | 0 | PNPs |
| G2E3 | 0 | 0.575094014699619 | 0.574305556 | 0.18125 | 0 | PNPs |
| BANF1 | 0 | 0.57374394102044 | 0.688888889 | 0.545138889 | 0 | PNPs |
| ECI2 | 0 | 0.572058564962683 | 0.628472222 | 0.279166667 | 0 | PNPs |
| MCM7 | 0 | 0.571645861130836 | 0.599305556 | 0.188888889 | 0 | PNPs |
| EZH2 | 0 | 0.569347815514104 | 0.672222222 | 0.390972222 | 0 | PNPs |
| SPRY1 | 0 | 0.567617828303134 | 0.643055556 | 0.288888889 | 0 | PNPs |
| KIF22 | 0 | 0.567362524402921 | 0.55 | 0.099 | 0 | PNPs |
| SKA2 | 0 | 0.563914149137392 | 0.686111111 | 0.526388889 | 0 | PNPs |
| HNRNPH3 | 0 | 0.56157363365293 | 0.690972222 | 0.552083333 | 0 | PNPs |
| GINS2 | 0 | 0.559765408638271 | 0.501388889 | 0.081944444 | 0 | PNPs |
| Sep-10 | 0 | 0.558203314372248 | 0.58125 | 0.1125 | 0 | PNPs |
| ANXA5 | 0 | 0.557158505082699 | 0.648611111 | 0.275694444 | 0 | PNPs |
| HNRNPAB | 0 | 0.556902761175586 | 0.69375 | 0.634722222 | 0 | PNPs |
| RAD51AP1 | 0 | 0.553006052831668 | 0.482638889 | 0.019 | 0 | PNPs |
| MND1 | 0 | 0.55226143304157 | 0.488194444 | 00:04 | 0 | PNPs |
| RPLP0 | 0 | 0.551441547181041 | 1 | 0.690277778 | 0 | PNPs |
| NES | 0 | 0.550091138506243 | 0.644444444 | 0.329861111 | 0 | PNPs |
| EZR | 0 | 0.54965004934474 | 0.56875 | 0.121527778 | 0 | PNPs |
| HNRNPA3 | 0 | 0.546336020734057 | 0.692361111 | 0.654166667 | 0 | PNPs |
| SAP30 | 0 | 0.544789236792604 | 0.642361111 | 0.320833333 | 0 | PNPs |
| CIP2A | 0 | 0.54442538479343 | 0.496527778 | 0.029 | 0 | PNPs |
| PARPBP | 0 | 0.542229167168023 | 0.499305556 | 0.031 | 0 | PNPs |
| HNRNPM | 0 | 0.541940296846175 | 0.690972222 | 0.534027778 | 0 | PNPs |
| CLSPN | 0 | 0.541233670306752 | 0.490972222 | 0.08125 | 0 | PNPs |
| BRCA1 | 0 | 0.539909908215763 | 0.497916667 | 0.057 | 0 | PNPs |
| SPC24 | 0 | 0.538774864673029 | 0.483333333 | 0.021 | 0 | PNPs |
| BTG3 | 0 | 0.537496961873128 | 0.568055556 | 0.158333333 | 0 | PNPs |
| B2M | 0 | 0.535139388924032 | 0.560416667 | 0.102777778 | 0 | PNPs |
| EEF1B2 | 0 | 0.534864927637621 | 1 | 0.671527778 | 0 | PNPs |
| PIF1 | 0 | 0.531706606836775 | 0.379166667 | 0.005 | 0 | PNPs |
| VRK1 | 0 | 0.529735918161026 | 0.578472222 | 0.120833333 | 0 | PNPs |
| PARP1 | 0 | 0.528555689970342 | 0.684027778 | 0.542361111 | 0 | PNPs |
| PRDX6 | 0 | 0.528384936153282 | 0.675 | 0.460416667 | 0 | PNPs |
| RPS6 | 0 | 0.52739321649555 | 1 | 0.691666667 | 0 | PNPs |
| HIST1H2BH | 0 | 0.52542736264818 | 0.490277778 | 0.074 | 0 | PNPs |
| SPDL1 | 0 | 0.522467110615077 | 0.552777778 | 0.129166667 | 0 | PNPs |
| MRPL51 | 0 | 0.521711101339404 | 0.691666667 | 0.588888889 | 0 | PNPs |
| SRSF7 | 0 | 0.520182398057192 | 0.686111111 | 0.503472222 | 0 | PNPs |
| SPAG5 | 0 | 0.518700997179355 | 0.046527778 | 0.014 | 0 | PNPs |
| CKAP5 | 0 | 0.51761370160747 | 0.608333333 | 0.246527778 | 0 | PNPs |
| GPSM2 | 0 | 0.513479614035169 | 0.514583333 | 0.106944444 | 0 | PNPs |
| HES6 | 0 | 0.513052425857266 | 0.665972222 | 00:47 | 0 | PNPs |
| SFPQ | 0 | 0.512188366747243 | 0.690972222 | 0.622916667 | 0 | PNPs |
| MIS18A | 0 | 0.508256519326311 | 0.59375 | 0.16875 | 0 | PNPs |
| SRSF2 | 0 | 0.508057621022416 | 0.6625 | 0.372222222 | 0 | PNPs |
| DNAJC9 | 0 | 0.504730656323072 | 0.6 | 0.216666667 | 0 | PNPs |
| COL4A6 | 0 | 0.502692274381549 | 0.525 | 0.148611111 | 0 | PNPs |
| WEE1 | 0 | 0.501472199323501 | 0.050694444 | 0.077083333 | 0 | PNPs |
| ANP32A | 0 | 0.497312284717813 | 0.681944444 | 0.466666667 | 0 | PNPs |
| RPL41 | 0 | 0.496827685234955 | 1 | 1 | 0 | PNPs |
| RNASEH2C | 0 | 0.493284934542037 | 0.647916667 | 0.33125 | 0 | PNPs |
| TCF7L2 | 0 | 0.490764851284643 | 0.494444444 | 0.079861111 | 0 | PNPs |
| HIST1H3G | 0 | 0.486225173686634 | 0.3375 | 0.008 | 0 | PNPs |
| TUBB6 | 0 | 0.486169355092463 | 0.414583333 | 0.022 | 0 | PNPs |
| RPL10A | 0 | 0.484871374222311 | 0.69375 | 0.683333333 | 0 | PNPs |
| CENPM | 0 | 0.483147569021283 | 0.454861111 | 0.021 | 0 | PNPs |
| BCL2L12 | 0 | 0.481911465313668 | 0.513194444 | 0.085 | 0 | PNPs |
| INCENP | 0 | 0.481314049514472 | 0.447222222 | 0.023 | 0 | PNPs |
| CDK5RAP2 | 0 | 0.476510831216357 | 0.604861111 | 0.234722222 | 0 | PNPs |
| GADD45G | 0 | 0.47288567641153 | 0.586805556 | 0.225694444 | 0 | PNPs |
| HSPB1 | 0 | 0.472158775363165 | 0.586111111 | 0.191666667 | 0 | PNPs |
| CEP70 | 0 | 0.471968686235507 | 0.474305556 | 0.106944444 | 0 | PNPs |
| ALYREF | 0 | 0.471485035887669 | 0.061805556 | 00:42 | 0 | PNPs |
| ECI1 | 0 | 0.470478114635118 | 0.603472222 | 0.246527778 | 0 | PNPs |
| RPS18 | 0 | 0.469595863024509 | 1 | 0.69375 | 0 | PNPs |
| ERH | 0 | 0.466530418612869 | 0.693055556 | 0.655555556 | 0 | PNPs |
| SNRPG | 0 | 0.465947623016673 | 0.692361111 | 0.608333333 | 0 | PNPs |
| FANCI | 0 | 0.465275978968907 | 0.452083333 | 0.029 | 0 | PNPs |
| HNRNPA1 | 0 | 0.464510271268033 | 1 | 0.686805556 | 0 | PNPs |
| ANAPC11 | 0 | 0.462654206002059 | 0.690972222 | 0.60625 | 0 | PNPs |
| HIST1H2AH | 0 | 0.460849798115157 | 0.284027778 | 0.021 | 0 | PNPs |
| PAICS | 0 | 0.458452048164334 | 0.613888889 | 0.264583333 | 0 | PNPs |
| MRPL11 | 0 | 0.455077821057895 | 0.63125 | 0.279861111 | 0 | PNPs |
| NPC2 | 0 | 0.45353983426979 | 0.595138889 | 0.240277778 | 0 | PNPs |
| SNRPF | 0 | 0.450362139573141 | 0.693055556 | 0.600694444 | 0 | PNPs |
| CD99 | 0 | 0.448495213187552 | 0.438194444 | 0.104861111 | 0 | PNPs |
| PCNA | 0 | 0.44782783779786 | 0.442361111 | 0.089583333 | 0 | PNPs |
| FRMD4B | 0 | 0.446876009907769 | 0.415972222 | 0.081944444 | 0 | PNPs |
| TGIF1 | 0 | 0.446440576587869 | 0.494444444 | 0.086111111 | 0 | PNPs |
| HIST1H3B | 0 | 0.446007111593891 | 0.286805556 | 0.004 | 0 | PNPs |
| CENPC | 0 | 0.439323488388054 | 0.510416667 | 0.136111111 | 0 | PNPs |

| RFC4 | 0 | 0.439222139165228 | 0.511111111 | 0.116666667 | 0 | PNPs |
| --- | --- | --- | --- | --- | --- | --- |
| CDCA5 | 0 | 0.437021226251177 | 0.415277778 | 0.008 | 0 | PNPs |
| PTX3 | 0 | 0.435282357819173 | 0.355555556 | 00:12 | 0 | PNPs |
| ANLN | 0 | 0.434369481925342 | 0.395138889 | 0.012 | 0 | PNPs |
| RPLP1 | 0 | 0.433133526424811 | 1 | 1 | 0 | PNPs |
| MT2A | 0 | 0.431472453999611 | 0.192361111 | 0.033 | 0 | PNPs |
| PSAT1 | 0 | 0.431357187997102 | 0.533333333 | 0.147916667 | 0 | PNPs |
| DDX39A | 0 | 0.430741644875456 | 0.511111111 | 0.115972222 | 0 | PNPs |
| ARHGAP11A | 0 | 0.430684373243946 | 0.393055556 | 0.011 | 0 | PNPs |
| ASRGL1 | 0 | 0.425404385192793 | 0.596527778 | 0.24375 | 0 | PNPs |
| MAGOHB | 0 | 0.425347812492306 | 0.551388889 | 0.155555556 | 0 | PNPs |
| SEPHS1 | 0 | 0.425151234728752 | 0.594444444 | 0.250694444 | 0 | PNPs |
| CALD1 | 0 | 0.424306916865777 | 0.568055556 | 0.197222222 | 0 | PNPs |
| RPS9 | 0 | 0.423629466959826 | 1 | 0.690277778 | 0 | PNPs |
| ATAD5 | 0 | 0.422545526364912 | 0.511805556 | 0.109027778 | 0 | PNPs |
| MELK | 0 | 0.421689907373865 | 0.417361111 | 0.007 | 0 | PNPs |
| REST | 0 | 0.418586334951246 | 0.442361111 | 0.069 | 0 | PNPs |
| CCDC18 | 0 | 0.416758314695375 | 0.404861111 | 0.039 | 0 | PNPs |
| ACTL6A | 0 | 0.416234061588344 | 0.605555556 | 0.239583333 | 0 | PNPs |
| SPART | 0 | 0.415873494657843 | 0.563194444 | 0.190972222 | 0 | PNPs |
| FOXM1 | 0 | 0.414823264360332 | 0.422916667 | 0.014 | 0 | PNPs |
| GPX8 | 0 | 0.413525919335241 | 0.425 | 0.042 | 0 | PNPs |
| SAE1 | 0 | 0.412155831935553 | 0.538194444 | 00:21 | 0 | PNPs |
| FAM83D | 0 | 0.412058019335764 | 0.363888889 | 0.005 | 0 | PNPs |
| RPL35 | 0 | 0.410151326532748 | 1 | 0.690277778 | 0 | PNPs |
| POC1A | 0 | 0.409269084482501 | 0.420833333 | 00:02 | 0 | PNPs |
| HAT1 | 0 | 0.409138020218692 | 0.513194444 | 0.135416667 | 0 | PNPs |
| OIP5 | 0 | 0.408966305251433 | 0.409027778 | 0.011 | 0 | PNPs |
| HMGN5 | 0 | 0.407748750384444 | 0.457638889 | 0.084722222 | 0 | PNPs |
| RPL23A | 0 | 0.407476902659272 | 1 | 0.689583333 | 0 | PNPs |
| PTGES3 | 0 | 0.40635674627349 | 0.69375 | 0.671527778 | 0 | PNPs |
| HILPDA | 0 | 0.405693693874938 | 0.565277778 | 0.198611111 | 0 | PNPs |
| SOX3 | 0 | 0.404344199957277 | 0.359722222 | 0.081 | 0 | PNPs |
| TIMELESS | 0 | 0.399622170368428 | 0.433333333 | 00:06 | 0 | PNPs |
| H2AFJ | 0 | 0.399119165762571 | 0.4875 | 0.132638889 | 0 | PNPs |
| STK26 | 0 | 0.395726604668156 | 0.477777778 | 0.096527778 | 0 | PNPs |
| YBX3 | 0 | 0.395658416141013 | 0.482638889 | 0.1 | 0 | PNPs |
| COL4A2 | 0 | 0.393033936794595 | 0.507638889 | 00:18 | 0 | PNPs |
| IFITM3 | 0 | 0.392391069075884 | 0.429166667 | 0.09375 | 0 | PNPs |
| TPBG | 0 | 0.392319650385351 | 0.372916667 | 0.074305556 | 0 | PNPs |
| TXNDC12 | 0 | 0.391629816491388 | 0.580555556 | 0.223611111 | 0 | PNPs |
| NENF | 0 | 0.387672835079649 | 0.459722222 | 00:14 | 0 | PNPs |
| ITGB3BP | 0 | 0.386871853919379 | 0.496527778 | 0.115277778 | 0 | PNPs |
| TMEM123 | 0 | 0.386417201467512 | 0.052777778 | 00:21 | 0 | PNPs |
| TEAD2 | 0 | 0.386077855259123 | 0.534027778 | 0.132638889 | 0 | PNPs |
| CRNDE | 0 | 0.384758505661661 | 0.477083333 | 0.140277778 | 0 | PNPs |
| NETO2 | 0 | 0.384086871288907 | 0.565972222 | 0.215972222 | 0 | PNPs |
| NDE1 | 0 | 0.383582876456745 | 00:53 | 0.021 | 0 | PNPs |
| CDCA4 | 0 | 0.381686745458497 | 0.420833333 | 0.053 | 0 | PNPs |
| NEIL3 | 0 | 0.379276044344362 | 0.366666667 | 0.005 | 0 | PNPs |
| BRCA2 | 0 | 0.379115641354455 | 0.378472222 | 0.013 | 0 | PNPs |
| GAS2L3 | 0 | 0.377136730709257 | 0.338888889 | 0.005 | 0 | PNPs |
| RPS15 | 0 | 0.375576819439065 | 1 | 0.69375 | 0 | PNPs |
| ACAA2 | 0 | 0.374051087666895 | 0.547222222 | 0.18125 | 0 | PNPs |
| NCAPH | 0 | 0.373833902009535 | 0.382638889 | 0.005 | 0 | PNPs |
| DIAPH3 | 0 | 0.371432332106321 | 0.379166667 | 0.008 | 0 | PNPs |
| CNTRL | 0 | 0.370513598745846 | 0.045138889 | 0.095138889 | 0 | PNPs |
| NUDT15 | 0 | 0.368408819727686 | 0.511805556 | 00:19 | 0 | PNPs |
| FZD2 | 0 | 0.367910750754154 | 0.445138889 | 0.075694444 | 0 | PNPs |
| LRR1 | 0 | 0.367197365230931 | 0.415972222 | 0.047 | 0 | PNPs |
| COMMD4 | 0 | 0.366712492299516 | 0.552777778 | 0.193055556 | 0 | PNPs |
| NCAPG2 | 0 | 0.366426925421821 | 0.410416667 | 0.052 | 0 | PNPs |
| TMEM237 | 0 | 0.363395286351873 | 0.522222222 | 0.14375 | 0 | PNPs |
| NSMCE4A | 0 | 0.363067013255864 | 0.546527778 | 0.18125 | 0 | PNPs |
| AHCY | 0 | 0.363052245015032 | 0.529861111 | 0.158333333 | 0 | PNPs |
| PPIA | 0 | 0.362257357467338 | 1 | 0.685416667 | 0 | PNPs |
| CHAF1A | 0 | 0.362023312766731 | 0.400694444 | 0.057 | 0 | PNPs |
| CDKN2C | 0 | 0.359224765463068 | 0.321527778 | 0.007 | 0 | PNPs |
| KIF20A | 0 | 0.35780084329936 | 0.344444444 | 0.002 | 0 | PNPs |
| RFC3 | 0 | 0.354469304956486 | 0.415277778 | 0.066 | 0 | PNPs |
| SOX1 | 0 | 0.35436292616302 | 0.452777778 | 0.091666667 | 0 | PNPs |
| PHF19 | 0 | 0.354011889014114 | 0.3375 | 0.023 | 0 | PNPs |
| MCM10 | 0 | 0.352022900436381 | 0.328472222 | 0.004 | 0 | PNPs |
| RHNO1 | 0 | 0.351319905521159 | 0.507638889 | 0.144444444 | 0 | PNPs |
| APOLD1 | 0 | 0.348565392862691 | 0.331944444 | 0.007 | 0 | PNPs |
| CENPL | 0 | 0.347050359388991 | 0.375694444 | 0.026 | 0 | PNPs |
| SKA3 | 0 | 0.346207833038555 | 0.363888889 | 0.005 | 0 | PNPs |
| SPATA5 | 0 | 0.345146063801378 | 0.413888889 | 0.088 | 0 | PNPs |
| C19orf48 | 0 | 0.345122026260719 | 0.44375 | 0.103472222 | 0 | PNPs |
| CIT | 0 | 0.343720771392304 | 0.378472222 | 0.033 | 0 | PNPs |
| BORA | 0 | 0.342950702818791 | 0.336805556 | 0.011 | 0 | PNPs |
| COL2A1 | 0 | 0.342846918951397 | 0.3625 | 0.064 | 0 | PNPs |
| ADD3 | 0 | 0.342505281882744 | 00:59 | 0.076 | 0 | PNPs |
| CEP135 | 0 | 0.341785226992127 | 0.043055556 | 0.077083333 | 0 | PNPs |
| IGFBP5 | 0 | 0.337109859201683 | 0.3 | 0.092 | 0 | PNPs |
| GGCT | 0 | 0.334745444609541 | 0.470138889 | 0.152083333 | 0 | PNPs |
| FANCD2 | 0 | 0.332107358703529 | 0.369444444 | 0.022 | 0 | PNPs |
| YBX1 | 0 | 0.331700813459422 | 0.69375 | 0.69375 | 0 | PNPs |
| NUP37 | 0 | 0.331687505216798 | 0.397222222 | 0.077 | 0 | PNPs |
| CTNNAL1 | 0 | 0.331553167706766 | 0.453472222 | 0.122222222 | 0 | PNPs |
| RANGAP1 | 0 | 0.330731647539685 | 0.426388889 | 0.09375 | 0 | PNPs |
| BDH2 | 0 | 0.330621816410089 | 0.536805556 | 0.172916667 | 0 | PNPs |
| CYYR1 | 0 | 0.328761401397064 | 0.465972222 | 0.125694444 | 0 | PNPs |
| EMC9 | 0 | 0.328347661529065 | 0.449305556 | 0.097916667 | 0 | PNPs |
| ARHGEF39 | 0 | 0.328101764037622 | 0.3375 | 0.008 | 0 | PNPs |
| USP44 | 0 | 0.327317728856504 | 0.420833333 | 00:12 | 0 | PNPs |
| FEN1 | 0 | 0.326982594645148 | 0.455555556 | 0.140277778 | 0 | PNPs |
| GLI3 | 0 | 0.326268472419029 | 0.399305556 | 0.095 | 0 | PNPs |
| GJA1 | 0 | 0.324640683266983 | 0.375694444 | 0.084722222 | 0 | PNPs |
| TTF2 | 0 | 0.324491018456012 | 0.358333333 | 0.041 | 0 | PNPs |

| SRRT | 0 | 0.324402927438766 | 0.534027778 | 0.182638889 | 0 | PNPs |
| --- | --- | --- | --- | --- | --- | --- |
| JAM2 | 0 | 0.324380012124273 | 0.472916667 | 0.113194444 | 0 | PNPs |
| CDT1 | 0 | 0.32320552832901 | 0.338888889 | 00:04 | 0 | PNPs |
| PRIM1 | 0 | 0.322387434169718 | 0.476388889 | 0.122222222 | 0 | PNPs |
| CENPJ | 0 | 0.322061448894915 | 0.432638889 | 0.088888889 | 0 | PNPs |
| CNTLN | 0 | 0.32004131062544 | 0.417361111 | 0.077777778 | 0 | PNPs |
| RNASEH2B | 0 | 0.319517540671067 | 0.393055556 | 0.075 | 0 | PNPs |
| PLIN3 | 0 | 0.315690504605616 | 0.377083333 | 0.089 | 0 | PNPs |
| CCNF | 0 | 0.31145791876212 | 0.345138889 | 0.034 | 0 | PNPs |
| DNA2 | 0 | 0.310416393088157 | 0.371527778 | 0.042 | 0 | PNPs |
| HIST1H2AG | 0 | 0.310372600859517 | 0.299305556 | 0.035 | 0 | PNPs |
| IKBIP | 0 | 0.309626057078636 | 0.440972222 | 0.109722222 | 0 | PNPs |
| DNPH1 | 0 | 0.307584219119541 | 0.452777778 | 0.130555556 | 0 | PNPs |
| AC011447.3 | 0 | 0.305404935581613 | 0.386805556 | 0.089 | 0 | PNPs |
| CEP44 | 0 | 0.301450812346831 | 0.440972222 | 0.111805556 | 0 | PNPs |
| TP53I13 | 0 | 0.301344196489857 | 0.042361111 | 00:12 | 0 | PNPs |
| NCAPD3 | 0 | 0.300593738093973 | 0.422916667 | 0.086111111 | 0 | PNPs |
| POLD3 | 0 | 0.300575022216565 | 0.436805556 | 00:17 | 0 | PNPs |
| TMA16 | 0 | 0.30037304891508 | 0.429861111 | 0.102777778 | 0 | PNPs |
| COL4A5 | 0 | 0.299525235295662 | 0.384027778 | 0.070138889 | 0 | PNPs |
| BLM | 0 | 0.29884343175762 | 00:48 | 0.021 | 0 | PNPs |
| AIF1L | 0 | 0.298803972736488 | 0.420833333 | 0.089583333 | 0 | PNPs |
| SOX21 | 0 | 0.297295400713108 | 0.320833333 | 0.075 | 0 | PNPs |
| MDC1 | 0 | 0.297076788500832 | 0.390277778 | 0.070138889 | 0 | PNPs |
| IQGAP3 | 0 | 0.293076835525214 | 0.297916667 | 0.007 | 0 | PNPs |
| CCDC77 | 0 | 0.292545595875856 | 0.398611111 | 00:12 | 0 | PNPs |
| RMI2 | 0 | 0.291156873790291 | 0.320138889 | 0.031 | 0 | PNPs |
| GINS4 | 0 | 0.286751047449178 | 0.322916667 | 0.031 | 0 | PNPs |
| HAUS6 | 0 | 0.286234785184153 | 0.461111111 | 0.134722222 | 0 | PNPs |
| PCNT | 0 | 0.285149351417595 | 0.45 | 0.129166667 | 0 | PNPs |
| UACA | 0 | 0.282559177824609 | 0.300694444 | 0.017 | 0 | PNPs |
| SAPCD2 | 0 | 0.280599834021473 | 0.288888889 | 0.004 | 0 | PNPs |
| CEP192 | 0 | 0.280106503537255 | 0.359027778 | 0.071 | 0 | PNPs |
| PSMC3IP | 0 | 0.279598356128313 | 0.372222222 | 0.095 | 0 | PNPs |
| HADH | 0 | 0.278597018743393 | 0.422222222 | 0.096527778 | 0 | PNPs |
| PTBP1 | 0 | 0.278014765384779 | 0.427083333 | 0.106944444 | 0 | PNPs |
| GINS1 | 0 | 0.27666929215697 | 0.343055556 | 00:04 | 0 | PNPs |
| CHRAC1 | 0 | 0.276546986129064 | 0.441666667 | 0.113194444 | 0 | PNPs |
| BRIP1 | 0 | 0.276374095592956 | 0.293055556 | 0.006 | 0 | PNPs |
| UCP2 | 0 | 0.275372311577482 | 0.369444444 | 0.077777778 | 0 | PNPs |
| XRCC2 | 0 | 0.272322338532831 | 0.301388889 | 0.013 | 0 | PNPs |
| DTL | 0 | 0.27156042174935 | 0.25625 | 0.005 | 0 | PNPs |
| TCF7L1 | 0 | 0.27126184598442 | 00:48 | 0.045 | 0 | PNPs |
| DLEU2 | 0 | 0.27098458118343 | 00:47 | 0.053 | 0 | PNPs |
| E2F7 | 0 | 0.270932252579692 | 00:43 | 00:02 | 0 | PNPs |
| FLNA | 0 | 0.270506949684768 | 0.411805556 | 0.1125 | 0 | PNPs |
| SHCBP1 | 0 | 0.270465752718898 | 0.30625 | 0.011 | 0 | PNPs |
| SHROOM3 | 0 | 0.269618862756873 | 0.4125 | 0.105555556 | 0 | PNPs |
| COL11A1 | 0 | 0.268553381722403 | 0.371527778 | 0.079861111 | 0 | PNPs |
| ZMYM1 | 0 | 0.268379713793135 | 0.3875 | 0.088194444 | 0 | PNPs |
| E2F1 | 0 | 0.268318292146237 | 0.329166667 | 0.098 | 0 | PNPs |
| MECOM | 0 | 0.268273405216347 | 0.34375 | 0.085416667 | 0 | PNPs |
| TMPO-AS1 | 0 | 0.267949970424353 | 00:53 | 0.087 | 0 | PNPs |
| MCM3 | 0 | 0.266166140835527 | 0.302777778 | 0.076 | 0 | PNPs |
| LMNB2 | 0 | 0.261867920900259 | 00:55 | 0.077777778 | 0 | PNPs |
| IMPA2 | 0 | 0.261555105804289 | 0.304861111 | 00:04 | 0 | PNPs |
| NEDD1 | 0 | 0.260192482338963 | 0.376388889 | 00:09 | 0 | PNPs |
| PCDH18 | 0 | 0.259811667315062 | 0.277083333 | 0.032 | 0 | PNPs |
| SCLT1 | 0 | 0.25934089460225 | 0.366666667 | 0.079861111 | 0 | PNPs |
| REEP4 | 0 | 0.257847262064514 | 0.279861111 | 00:02 | 0 | PNPs |
| MYBL2 | 0 | 0.257812684741419 | 0.24375 | 0.007 | 0 | PNPs |
| GSTO1 | 0 | 0.257703552814581 | 0.394444444 | 0.103472222 | 0 | PNPs |
| CENPQ | 0 | 0.257553898966298 | 0.339583333 | 0.054 | 0 | PNPs |
| SUV39H2 | 0 | 0.254155548289418 | 0.385416667 | 0.084027778 | 0 | PNPs |
| AP3B1 | 0 | 0.250669334834199 | 0.419444444 | 0.114583333 | 0 | PNPs |
| SNRPA | 5.25403296853753e-307 | 0.303040848145962 | 0.513194444 | 0.170138889 | 1.09089486525745e-302 | PNPs |
| UQCC2 | 4.56342928290234e-306 | 0.476166163978952 | 0.643055556 | 0.336111111 | 9.47504822009014e-302 | PNPs |
| RBMX | 5.37879689175027e-304 | 0.49275116734577 | 0.691666667 | 0.588888889 | 1.11679959863411e-299 | PNPs |
| MAGOH | 1.62347791072836e-303 | 0.442741300160202 | 0.651388889 | 0.33125 | 3.37082718604529e-299 | PNPs |
| MFAP2 | 4.12459740150978e-303 | 0.419246567543186 | 0.565277778 | 00:33 | 8.56390158475477e-299 | PNPs |
| RPS19 | 5.89876579531954e-302 | 0.377675402491166 | 0.69375 | 1 | 1.2247607420822e-297 | PNPs |
| PNRC2 | 3.67726685599209e-301 | 0.45490107114831 | 0.622222222 | 0.302083333 | 7.63510917309637e-297 | PNPs |
| IGFBP2 | 1.12923857902847e-300 | 0.604073816005011 | 0.689583333 | 0.535416667 | 2.34463806163681e-296 | PNPs |
| RPL39L | 4.41337696182672e-299 | 0.30123193555124 | 0.432638889 | 00:19 | 9.16349458584082e-295 | PNPs |
| CMC2 | 8.83128203264757e-297 | 0.440514667571119 | 0.629166667 | 0.304861111 | 1.83363908843861e-292 | PNPs |
| OTX2 | 2.21822041141591e-296 | 0.370419976051136 | 0.049305556 | 0.175694444 | 4.60569104022285e-292 | PNPs |
| GJC1 | 3.03689719949693e-296 | 0.257795226692563 | 0.422222222 | 0.122916667 | 6.30550965531547e-292 | PNPs |
| ENY2 | 9.24697590869923e-296 | 0.469176703003731 | 0.690972222 | 0.581944444 | 1.91994960792322e-291 | PNPs |
| HIST1H1D | 1.38850282591832e-295 | 1.09877738930299 | 0.359722222 | 0.114583333 | 2.88294841745421e-291 | PNPs |
| RPSA | 1.85476846931657e-294 | 0.385034495923032 | 1 | 0.690277778 | 3.85105577284199e-290 | PNPs |
| TBL1X | 3.13041932318655e-294 | 0.288556661245936 | 0.469444444 | 0.152083333 | 6.49968964073224e-290 | PNPs |
| TIMM10 | 8.77622804437022e-294 | 0.417087334790085 | 0.061111111 | 0.278472222 | 1.82220822885259e-289 | PNPs |
| C12orf75 | 5.02637264447296e-293 | 0.436797514522393 | 0.624305556 | 0.29375 | 1.04362575217192e-288 | PNPs |
| CBX5 | 2.20058321107154e-291 | 0.420509249439729 | 0.690972222 | 0.656944444 | 4.56907092114784e-287 | PNPs |
| ENO1 | 2.37668679232e-291 | 0.505394541965196 | 0.64375 | 0.352777778 | 4.93471478689402e-287 | PNPs |
| SPA17 | 3.95437563015925e-289 | 0.404351050813326 | 0.561805556 | 0.225694444 | 8.21047012089965e-285 | PNPs |
| H2AFY | 2.45391588484872e-288 | 0.430486187224649 | 0.690972222 | 0.608333333 | 5.0950655517114e-284 | PNPs |
| NPM1 | 2.92568948753222e-288 | 0.428127133637414 | 1 | 0.682638889 | 6.07460908296314e-284 | PNPs |
| FBL | 1.49392253596539e-284 | 0.308501053058031 | 0.500694444 | 00:25 | 3.10183136142493e-280 | PNPs |
| HSPD1 | 1.88062291724067e-284 | 0.477400752076983 | 0.688194444 | 0.591666667 | 3.9047373630668e-280 | PNPs |
| PTMA | 2.69878123138438e-281 | 0.25068884466135 | 1 | 1 | 5.60347947072339e-277 | PNPs |
| HMGN1 | 4.80396209041219e-281 | 0.340647967306186 | 1 | 0.06875 | 9.97446648832283e-277 | PNPs |
| CALU | 1.84814565704523e-276 | 0.382394853112945 | 0.578472222 | 0.249305556 | 3.83730482772301e-272 | PNPs |
| DCP2 | 9.26722748809647e-276 | 0.276666333475138 | 0.48125 | 00:23 | 1.92415444335347e-271 | PNPs |
| GLO1 | 1.80918105092082e-275 | 0.440136415644843 | 0.664583333 | 0.403472222 | 3.7564026160269e-271 | PNPs |
| KMT5A | 8.80323965329846e-273 | 0.334814263207223 | 0.513194444 | 0.185416667 | 1.82781664921436e-268 | PNPs |
| SNRPD3 | 4.42912120620621e-272 | 0.436513123569743 | 0.068055556 | 0.501388889 | 9.19618436044595e-268 | PNPs |
| SFRP1 | 8.02531796224544e-272 | 0.476441832646774 | 0.565277778 | 0.246527778 | 1.66629676850102e-267 | PNPs |
| XRCC5 | 2.43641881804915e-271 | 0.367712198689359 | 0.692361111 | 0.66875 | 5.05873639191545e-267 | PNPs |
| SEM1 | 3.31789546041785e-271 | 0.414527335591366 | 0.689583333 | 0.602777778 | 6.88894634446558e-267 | PNPs |

| CMTM6 | 2.81920920994902e-270 | 0.278131192963409 | 0.511111111 | 0.178472222 | 5.85352408261714e-266 | PNPs |
| --- | --- | --- | --- | --- | --- | --- |
| TCF3 | 6.6368910828057e-269 | 0.345365945923305 | 0.582638889 | 0.2375 | 1.37801769552295e-264 | PNPs |
| RPS20 | 2.46547138452657e-268 | 0.448477974241517 | 0.69375 | 0.684722222 | 5.11905823569252e-264 | PNPs |
| WDR34 | 5.12333728262601e-268 | 0.286535176466521 | 0.481944444 | 0.165277778 | 1.06375851999164e-263 | PNPs |
| NAP1L1 | 6.31487927861745e-268 | 0.413161259317378 | 1 | 0.681944444 | 1.31115838461934e-263 | PNPs |
| PHF6 | 1.14538247706033e-266 | 0.506240143208544 | 0.690972222 | 0.559027778 | 2.37815763712037e-262 | PNPs |
| SMC1A | 2.42561057284195e-266 | 0.440848556185523 | 0.615972222 | 0.313888889 | 5.03629523239173e-262 | PNPs |
| ZNF511 | 4.52121008878573e-265 | 0.261568057907557 | 0.483333333 | 0.1625 | 9.38738850734581e-261 | PNPs |
| SRSF10 | 1.40920324729486e-263 | 0.432357794700026 | 0.068055556 | 0.509027778 | 2.92592870235832e-259 | PNPs |
| SYNE2 | 3.14418844802619e-262 | 0.532049861516521 | 0.672222222 | 0.492361111 | 6.52827847463678e-258 | PNPs |
| CNIH4 | 6.6541676724448e-261 | 0.502647651770173 | 0.656944444 | 0.4 | 1.38160483382971e-256 | PNPs |
| RPL3 | 2.00312445883689e-260 | 0.33047653098688 | 1 | 0.69375 | 4.15908731388303e-256 | PNPs |
| KIAA0586 | 1.45387485403466e-258 | 0.329388670270413 | 0.509027778 | 0.191666667 | 3.01868035943217e-254 | PNPs |
| DESI2 | 4.15946327580393e-257 | 0.36686336887026 | 0.586805556 | 0.259722222 | 8.6362935995517e-253 | PNPs |
| UBB | 9.23388281657321e-256 | 0.386056758640926 | 0.693055556 | 0.688888889 | 1.9172310892051e-251 | PNPs |
| PRKDC | 3.58209278841805e-255 | 0.46572739604009 | 0.672916667 | 0.530555556 | 7.4374992565924e-251 | PNPs |
| PDIA6 | 8.96149346966277e-255 | 0.441575035106143 | 0.675 | 0.477777778 | 1.86067488910608e-250 | PNPs |
| DKC1 | 1.80279758941863e-253 | 0.277131635135885 | 0.488888889 | 00:25 | 3.7431486349099e-249 | PNPs |
| CHEK1 | 2.24284273890914e-250 | 0.297852598010443 | 0.521527778 | 0.197222222 | 4.65681437879704e-246 | PNPs |
| IGF2BP1 | 1.05955684222003e-248 | 0.403325688053649 | 0.622916667 | 00:45 | 2.19995787150144e-244 | PNPs |
| CEP78 | 1.85599231350097e-248 | 0.282678547756002 | 0.523611111 | 0.195138889 | 3.85359684052207e-244 | PNPs |
| RPA2 | 8.71383623969737e-248 | 0.291152053207976 | 0.496527778 | 0.181944444 | 1.80925381844836e-243 | PNPs |
| CEP295 | 9.72887474987409e-248 | 0.250382700056519 | 0.435416667 | 0.143055556 | 2.02000626431636e-243 | PNPs |
| RPS16 | 1.23020065944085e-247 | 0.323852256353906 | 0.69375 | 0.691666667 | 2.55426562919704e-243 | PNPs |
| TMEM98 | 6.61803137344142e-246 | 0.407385931759864 | 0.638194444 | 0.336805556 | 1.37410185406764e-241 | PNPs |
| AC092958.1 | 7.07999857348175e-246 | 0.306062803264678 | 0.469444444 | 0.16875 | 1.47002010381201e-241 | PNPs |
| MOB1A | 1.69225070253479e-244 | 0.283565648254312 | 0.525 | 0.198611111 | 3.51362013367299e-240 | PNPs |
| PMF1 | 2.12929758626581e-244 | 0.361537405491965 | 0.604861111 | 0.285416667 | 4.42106057836369e-240 | PNPs |
| FAT1 | 4.91647214803832e-243 | 0.269025281639456 | 0.470138889 | 00:24 | 1.0208071120972e-238 | PNPs |
| CDK4 | 1.05029124442854e-242 | 0.4033331778256 | 0.640277778 | 0.341666667 | 2.18071971080698e-238 | PNPs |
| TSPAN6 | 1.59213088245277e-240 | 0.361928417847038 | 0.619444444 | 0.290277778 | 3.30574135123668e-236 | PNPs |
| HIST2H2AC | 3.19535897608941e-240 | 0.835039659724249 | 0.475 | 0.209722222 | 6.63452384205445e-236 | PNPs |
| RPS7 | 1.58560002614404e-238 | 0.3298098437476 | 1 | 0.69375 | 3.29218133428288e-234 | PNPs |
| LSM6 | 5.39014747599777e-238 | 0.405780816829616 | 0.658333333 | 0.428472222 | 1.11915632044142e-233 | PNPs |
| LSM3 | 1.05857420241177e-236 | 0.39503855610812 | 0.682638889 | 0.545833333 | 2.19791761646755e-232 | PNPs |
| RPL13 | 1.16140461094179e-236 | 0.263156311132124 | 1 | 1 | 2.41142439369843e-232 | PNPs |
| PSIP1 | 5.69716493414606e-236 | 0.383829507731526 | 0.690972222 | 0.646527778 | 1.18290235527675e-231 | PNPs |
| RPL18A | 1.29678863155157e-235 | 0.295027808073249 | 1 | 0.69375 | 2.69252223569052e-231 | PNPs |
| HMGXB4 | 3.23719359255819e-235 | 0.272984909712033 | 0.507638889 | 0.189583333 | 6.72138505622856e-231 | PNPs |
| CCDC14 | 2.41987676776692e-234 | 0.278510412454823 | 0.50625 | 0.193055556 | 5.02439013291445e-230 | PNPs |
| FGFR1 | 1.27161948304574e-233 | 0.279065332914867 | 0.481944444 | 0.176388889 | 2.64026353264787e-229 | PNPs |
| CENPV | 1.33492123423344e-232 | 0.468067732567504 | 0.682638889 | 0.606944444 | 2.77169695863889e-228 | PNPs |
| SNRPD2 | 2.20454767039442e-229 | 0.367796341350259 | 0.690972222 | 0.613194444 | 4.57730232803993e-225 | PNPs |
| PKM | 6.33150439421344e-229 | 0.401617494820429 | 0.669444444 | 0.459027778 | 1.31461025737054e-224 | PNPs |
| FUS | 4.7324780359688e-225 | 0.377093297178853 | 0.688888889 | 0.585416667 | 9.82604414608202e-221 | PNPs |
| GPN3 | 9.66199179333196e-223 | 0.25402461592656 | 0.478472222 | 0.175694444 | 2.00611935604952e-218 | PNPs |
| TMSB15A | 2.46385039662506e-222 | 0.376018300330073 | 0.692361111 | 0.675694444 | 5.11569257851261e-218 | PNPs |
| HNRNPU | 7.31705212661673e-222 | 0.361932223239084 | 0.69375 | 0.674305556 | 1.51923953304943e-217 | PNPs |
| TJP1 | 4.26016935959426e-218 | 0.357511610363347 | 0.61875 | 0.315277778 | 8.84538964132556e-214 | PNPs |
| RPS5 | 4.90515835959765e-216 | 0.273689829206302 | 0.69375 | 0.691666667 | 1.01845803020326e-211 | PNPs |
| HNRNPR | 9.49660737224519e-216 | 0.350121901748205 | 0.691666667 | 0.636111111 | 1.97178058869927e-211 | PNPs |
| RPS12 | 1.70784474771624e-215 | 0.269234466373946 | 1 | 1 | 3.54599804968322e-211 | PNPs |
| SEC11A | 5.99804618765852e-215 | 0.345169436034681 | 0.691666667 | 0.622222222 | 1.24537432994354e-210 | PNPs |
| RPP30 | 2.20852515685422e-214 | 0.268216289166705 | 0.525694444 | 0.2125 | 4.58556078317642e-210 | PNPs |
| XPO1 | 2.50775199572099e-212 | 0.370188376545417 | 0.629166667 | 0.352777778 | 5.20684546871549e-208 | PNPs |
| XRCC6 | 5.23708066781973e-211 | 0.37363395841292 | 0.672222222 | 0.470833333 | 1.08737505905941e-206 | PNPs |
| SS18 | 2.61105566390019e-210 | 0.3033948240685 | 0.523611111 | 0.221527778 | 5.42133487495597e-206 | PNPs |
| BRD8 | 3.95453676127565e-209 | 0.401652786041439 | 0.613888889 | 0.344444444 | 8.21080467743663e-205 | PNPs |
| FGFBP3 | 2.15609962676748e-208 | 0.31128342444459 | 0.353472222 | 0.117361111 | 4.47670965505731e-204 | PNPs |
| RPS3 | 4.3622193139944e-207 | 0.284796665091927 | 1 | 1 | 9.05727596164657e-203 | PNPs |
| COA1 | 6.62568715550116e-204 | 0.352632862980094 | 0.640972222 | 0.380555556 | 1.37569142409671e-199 | PNPs |
| RPL12 | 4.4636424494516e-203 | 0.31264524949034 | 1 | 0.692361111 | 9.26786081779636e-199 | PNPs |
| NME4 | 4.6568682899118e-203 | 0.355658242240226 | 0.595138889 | 0.296527778 | 9.66905563034386e-199 | PNPs |
| NAE1 | 4.73348791156493e-202 | 0.354054983434307 | 0.651388889 | 0.394444444 | 9.82814095078226e-198 | PNPs |
| LSM8 | 4.45530299661232e-201 | 0.362376872951067 | 0.659027778 | 0.40625 | 9.25054561186616e-197 | PNPs |
| FBLN1 | 9.40193161438946e-201 | 0.426729989772488 | 0.644444444 | 00:06 | 1.95212306109568e-196 | PNPs |
| RPL27A | 5.34709480484935e-199 | 0.358400278141518 | 1 | 0.686805556 | 1.11021729433087e-194 | PNPs |
| CCNB1IP1 | 5.46611115599936e-197 | 0.287256492966074 | 0.5875 | 0.2625 | 1.13492865932015e-192 | PNPs |
| NIN | 5.76649175750397e-197 | 0.280799743345719 | 0.051388889 | 0.213194444 | 1.19729668361055e-192 | PNPs |
| SRSF9 | 6.94526187473772e-196 | 0.298327810705792 | 0.692361111 | 0.660416667 | 1.44204472305179e-191 | PNPs |
| OSTC | 1.95124580921901e-195 | 0.35558745251879 | 0.677777778 | 0.474305556 | 4.05137167368142e-191 | PNPs |
| PRDX4 | 5.72886116718953e-195 | 0.397884813123274 | 0.632638889 | 0.38125 | 1.18948344414356e-190 | PNPs |
| RCN1 | 8.3859997374265e-195 | 0.329598332705243 | 0.629861111 | 0.334027778 | 1.74118512548186e-190 | PNPs |
| G3BP1 | 9.12392539985964e-195 | 0.342684905025689 | 0.063194444 | 0.354861111 | 1.89440063077286e-190 | PNPs |
| ARL6IP6 | 2.73757689473407e-194 | 0.285496728158389 | 0.540972222 | 0.238194444 | 5.68403090653635e-190 | PNPs |
| HNRNPA0 | 5.87761029828431e-194 | 0.337233633412282 | 0.688194444 | 0.617361111 | 1.22036822623277e-189 | PNPs |
| CCAR1 | 3.81263876407266e-190 | 0.329456111807759 | 0.577083333 | 0.286805556 | 7.91618186584406e-186 | PNPs |
| TPM1 | 1.14384618836117e-189 | 0.783803205275877 | 0.630555556 | 0.418055556 | 2.37496784089429e-185 | PNPs |
| AKR1A1 | 4.41451653819903e-189 | 0.309083234173909 | 0.591666667 | 0.288194444 | 9.16586068826264e-185 | PNPs |
| LMO4 | 1.54965851455559e-188 | 0.381555955963203 | 0.640277778 | 0.38125 | 3.21755597377177e-184 | PNPs |
| CAST | 2.15104500943432e-188 | 0.289535758121104 | 0.526388889 | 0.227777778 | 4.46621475308847e-184 | PNPs |
| SMS | 5.15022633761922e-188 | 0.433492070169017 | 0.690972222 | 0.646527778 | 1.06934149447988e-183 | PNPs |
| RPL22L1 | 2.21459203969367e-187 | 0.274096181893443 | 0.505555556 | 0.2125 | 4.59815745201596e-183 | PNPs |
| DMAC1 | 8.17577464020173e-187 | 0.313223062421539 | 0.59375 | 0.29375 | 1.69753608854508e-182 | PNPs |
| TOP1 | 2.17834692388645e-186 | 0.368034355279818 | 0.688888889 | 0.059027778 | 4.52290171806543e-182 | PNPs |
| HSPE1 | 3.08406577613801e-184 | 0.372247638919221 | 0.683333333 | 0.554861111 | 6.40344577099535e-180 | PNPs |
| TRA2B | 4.80041878568067e-184 | 0.317432456553352 | 0.61875 | 0.329861111 | 9.96710952470878e-180 | PNPs |
| SNRPA1 | 2.60177377836287e-181 | 0.311649729578653 | 0.600694444 | 0.310416667 | 5.40206289601483e-177 | PNPs |
| CNN3 | 1.71248118612535e-179 | 0.332331793246585 | 0.688888889 | 0.584722222 | 3.55562468675207e-175 | PNPs |
| RACK1 | 2.87129843895134e-178 | 0.252039403239976 | 1 | 0.693055556 | 5.96167694879467e-174 | PNPs |
| PON2 | 6.41119969010208e-178 | 0.269214562086873 | 0.554166667 | 0.247222222 | 1.3311573916559e-173 | PNPs |
| CTNNA1 | 3.20320674950576e-176 | 0.316998107631498 | 0.625694444 | 0.323611111 | 6.65081817399882e-172 | PNPs |
| ZIC4 | 1.88467582285804e-173 | 0.281030356393099 | 0.463888889 | 0.198611111 | 3.91315241100016e-169 | PNPs |
| HSPH1 | 1.35793674676749e-172 | 0.312046366665238 | 0.579166667 | 0.292361111 | 2.81948406731334e-168 | PNPs |
| RFC1 | 9.92906404253931e-172 | 0.301555293420263 | 0.55 | 0.263194444 | 2.06157156715244e-167 | PNPs |
| GMPS | 1.10785049138832e-171 | 0.319889094387483 | 0.622222222 | 0.348611111 | 2.30022997526956e-167 | PNPs |
| UBALD2 | 1.54184648263585e-171 | 0.322126254776298 | 0.561805556 | 0.284027778 | 3.20133585189681e-167 | PNPs |
| SERBP1 | 2.70152563209228e-170 | 0.295614758683094 | 0.692361111 | 0.659027778 | 5.60917766991321e-166 | PNPs |
| MYO10 | 1.91391069670916e-169 | 0.319543596895404 | 0.620833333 | 0.339583333 | 3.97385277957723e-165 | PNPs |
| ILF3 | 2.94194929428943e-166 | 0.322301354046948 | 0.679166667 | 0.532638889 | 6.10836931973315e-162 | PNPs |

| RBM8A | 7.59701750897795e-164 | 0.315476423655353 | 0.688888889 | 0.613194444 | 1.57736874538909e-159 | PNPs |
| --- | --- | --- | --- | --- | --- | --- |
| FEZF1 | 1.28394919040577e-162 | 0.280257922035315 | 0.477777778 | 0.209027778 | 2.6658637040395e-158 | PNPs |
| SMIM19 | 4.99221275622014e-162 | 0.27754403236528 | 0.595138889 | 0.311805556 | 1.03653313457399e-157 | PNPs |
| RBBP7 | 2.81716055117722e-160 | 0.323550836220626 | 0.6625 | 0.459027778 | 5.84927045240927e-156 | PNPs |
| GLRX5 | 1.02813472737551e-159 | 0.31090957780141 | 0.685416667 | 0.568055556 | 2.13471613444976e-155 | PNPs |
| LARP7 | 5.01396594179826e-159 | 0.312983260195572 | 0.635416667 | 0.369444444 | 1.04104974849557e-154 | PNPs |
| IPO5 | 8.18976559063284e-159 | 0.261210481783687 | 0.572916667 | 0.279861111 | 1.7004410295831e-154 | PNPs |
| PNN | 1.85394262020673e-158 | 0.337750651947651 | 0.671527778 | 0.050694444 | 3.84934106233523e-154 | PNPs |
| HNRNPC | 8.2907681615664e-158 | 0.302744977965181 | 0.689583333 | 0.59375 | 1.72141219338603e-153 | PNPs |
| YEATS4 | 8.8561340867629e-158 | 0.282523652030775 | 0.593055556 | 0.314583333 | 1.83879912043458e-153 | PNPs |
| NSD2 | 1.84460918031953e-157 | 0.29286545967711 | 0.608333333 | 0.340972222 | 3.82996204109744e-153 | PNPs |
| RPL13A | 3.38905178651342e-157 | 0.297406497523976 | 1 | 0.691666667 | 7.03668822433782e-153 | PNPs |
| IDH2 | 6.34627597415784e-157 | 0.334428980991872 | 0.638194444 | 0.404861111 | 1.31767728051439e-152 | PNPs |
| LINC01551 | 1.00560374373058e-156 | 0.435868149347989 | 0.609722222 | 0.04375 | 2.08793505310781e-152 | PNPs |
| SRSF1 | 1.54109919975156e-156 | 0.253805262616842 | 0.559722222 | 0.267361111 | 3.19978426844416e-152 | PNPs |
| NCL | 1.24908400478001e-155 | 0.284119267827779 | 1 | 0.679166667 | 2.59347311912474e-151 | PNPs |
| UBE2I | 1.40383251231741e-155 | 0.287702024134453 | 0.686805556 | 0.6125 | 2.91477744532464e-151 | PNPs |
| CARHSP1 | 9.47664094123054e-153 | 0.334228363419847 | 0.672916667 | 0.504166667 | 1.9676349586277e-148 | PNPs |
| SCAF11 | 1.46610049624652e-147 | 0.296389264030994 | 0.634027778 | 0.375694444 | 3.04406446035664e-143 | PNPs |
| TRIM28 | 1.8426907932402e-147 | 0.285231141586794 | 0.636805556 | 00:54 | 3.82597889400464e-143 | PNPs |
| PSMD10 | 2.15374487393184e-147 | 0.284570445834868 | 0.591666667 | 0.322916667 | 4.47182048174468e-143 | PNPs |
| RHEB | 6.22056843465216e-146 | 0.292397643892405 | 0.676388889 | 0.525 | 1.29157662408683e-141 | PNPs |
| ILF2 | 7.57789621062315e-146 | 0.314232164391476 | 0.690972222 | 0.615277778 | 1.57339859021168e-141 | PNPs |
| RHOA | 1.21129173632418e-145 | 0.288250945822707 | 0.688194444 | 0.558333333 | 2.51500503212989e-141 | PNPs |
| TXNRD1 | 2.15888622788814e-144 | 0.266019664258506 | 0.586111111 | 0.308333333 | 4.48249547496414e-140 | PNPs |
| TCF4 | 2.8330403525898e-140 | 0.279705334319806 | 0.577777778 | 0.309027778 | 5.88224168408221e-136 | PNPs |
| CALR | 3.23753237538869e-140 | 0.322691978813399 | 0.614583333 | 0.375694444 | 6.72208847101955e-136 | PNPs |
| PA2G4 | 2.14027194044298e-139 | 0.309043666491979 | 0.66875 | 0.502083333 | 4.44384662994175e-135 | PNPs |
| PHC2 | 1.10810638715731e-136 | 0.306050863063774 | 0.630555556 | 0.3875 | 2.30076129165473e-132 | PNPs |
| SLC25A24 | 1.92134189222241e-136 | 0.261632681054077 | 0.54375 | 0.275694444 | 3.98928217082139e-132 | PNPs |
| IGF2BP3 | 3.58865723611067e-136 | 0.285397026671137 | 0.610416667 | 0.35 | 7.45112901933658e-132 | PNPs |
| PHPT1 | 5.91405037351286e-136 | 0.311657566798612 | 0.681944444 | 0.590972222 | 1.22793427905247e-131 | PNPs |
| CCNG2 | 1.64358254150718e-135 | 0.324907945557143 | 0.598611111 | 00:51 | 3.41257043093136e-131 | PNPs |
| PTN | 3.29623167229179e-135 | 0.390146261382119 | 0.664583333 | 0.5375 | 6.84396582117945e-131 | PNPs |
| PSMC3 | 3.45317581534201e-135 | 0.291682500323773 | 0.647222222 | 0.445138889 | 7.16982894539461e-131 | PNPs |
| RTRAF | 1.52674403258784e-134 | 0.259446768063318 | 0.693055556 | 0.646527778 | 3.16997863486214e-130 | PNPs |
| RUVBL1 | 2.93808316299672e-134 | 0.25829821254281 | 0.586805556 | 0.317361111 | 6.1003420713301e-130 | PNPs |
| CALM2 | 4.26671224809351e-134 | 0.270686382289615 | 0.69375 | 0.691666667 | 8.85897464071656e-130 | PNPs |
| NEUROG1 | 1.14003635252234e-133 | 0.272512154628706 | 0.459722222 | 0.207638889 | 2.36705747874213e-129 | PNPs |
| ELAVL1 | 9.9105581562205e-131 | 0.259286664984228 | 0.620833333 | 0.352777778 | 2.05772918997606e-126 | PNPs |
| SUPT16H | 1.77713937846593e-130 | 0.300730844728868 | 0.676388889 | 0.536111111 | 3.68987449150881e-126 | PNPs |
| SMC3 | 5.37200121873207e-130 | 0.279023409480113 | 0.067361111 | 0.526388889 | 1.11538861304534e-125 | PNPs |
| RPS17 | 1.50477727118367e-129 | 0.302602410722323 | 0.674305556 | 0.520138889 | 3.12436904815866e-125 | PNPs |
| ZCRB1 | 5.99557131693312e-129 | 0.26931240918855 | 0.622222222 | 00:54 | 1.24486047253482e-124 | PNPs |
| PDIA3 | 7.91534827833388e-129 | 0.300567610298755 | 0.66875 | 0.514583333 | 1.64346376303046e-124 | PNPs |
| EIF4A3 | 2.29754576012053e-128 | 0.297509173624471 | 0.640972222 | 0.420138889 | 4.77039426173826e-124 | PNPs |
| CLNS1A | 6.12735536021582e-126 | 0.269138887038697 | 0.669444444 | 0.48125 | 1.27222279344161e-121 | PNPs |
| PFN1 | 9.84133520783727e-125 | 0.294823372831496 | 0.686111111 | 0.604861111 | 2.04335642920325e-120 | PNPs |
| CCT5 | 1.08347344888637e-121 | 0.265810569753969 | 0.684722222 | 0.578472222 | 2.24961592192277e-117 | PNPs |
| TPR | 2.25256725224672e-121 | 0.288068341554391 | 0.676388889 | 0.054861111 | 4.67700538583987e-117 | PNPs |
| CDH2 | 1.65262249998006e-119 | 0.276369255848565 | 0.683333333 | 0.622222222 | 3.4313400967086e-115 | PNPs |
| TMEM97 | 7.73668809203004e-119 | 0.259352290834868 | 0.669444444 | 0.475694444 | 1.6063685485482e-114 | PNPs |
| KIF5B | 5.83300778426967e-117 | 0.267934946239179 | 0.683333333 | 0.591666667 | 1.21110740624791e-112 | PNPs |
| PRDX3 | 6.53497856747617e-117 | 0.266462415979681 | 0.651388889 | 0.043055556 | 1.35685759996508e-112 | PNPs |
| CNBP | 3.70342705837589e-115 | 0.276282161465649 | 0.066666667 | 0.483333333 | 7.68942560130586e-111 | PNPs |
| NONO | 2.34403666580591e-114 | 0.268233524056974 | 0.681944444 | 0.572222222 | 4.8669233292128e-110 | PNPs |
| MRPL57 | 3.01799900299936e-114 | 0.251895975119751 | 0.658333333 | 0.499305556 | 6.26627132992757e-110 | PNPs |
| BRD7 | 3.47994092279625e-113 | 0.257142778115038 | 0.667361111 | 0.507638889 | 7.22540133800186e-109 | PNPs |
| FUBP1 | 7.96355703219023e-112 | 0.266883532382294 | 0.660416667 | 0.494444444 | 1.65347334659366e-107 | PNPs |
| TRMT112 | 1.32815899159624e-111 | 0.250229163513314 | 0.676388889 | 0.519444444 | 2.75765651425127e-107 | PNPs |
| DDX46 | 2.38617438063171e-109 | 0.263653055239684 | 0.065277778 | 0.457638889 | 4.95441386650561e-105 | PNPs |
| ZIC1 | 3.79993186392851e-109 | 0.361932263565921 | 0.603472222 | 0.515277778 | 7.88979852907477e-105 | PNPs |
| TRIM24 | 1.2538915841915e-108 | 0.259929167610143 | 0.644444444 | 0.450694444 | 2.60345509625681e-104 | PNPs |
| SINHCAF | 6.65374373292299e-105 | 0.255568407808616 | 0.657638889 | 0.469444444 | 1.3815168112668e-100 | PNPs |
| HIST1H1E | 1.16935953485797e-103 | 0.67146381152025 | 0.045138889 | 0.289583333 | 2.42794120222561e-99 | PNPs |
| DHX9 | 1.50677626583484e-97 | 0.252052625393585 | 0.644444444 | 0.454166667 | 3.12851956075287e-93 | PNPs |
| HIST1H4C | 1.53081657218874e-78 | 1.62341021290902 | 0.600694444 | 0.604861111 | 3.17843444883549e-74 | PNPs |
| PTPRZ1 | 3.71868404431263e-76 | 0.250476937124788 | 0.530555556 | 0.31875 | 7.72110368120632e-72 | PNPs |
| DUT | 2.32230183851465e-57 | 0.27635281102402 | 0.609722222 | 0.436111111 | 4.82179530730796e-53 | PNPs |
| CRABP1 | 0 | 2.51343661227136 | 0.468055556 | 0.096527778 | 0 | IRX+ |
| NR2F1 | 0 | 1.39370888038569 | 0.567361111 | 0.078472222 | 0 | IRX+ |
| RND3 | 0 | 1.09891666973903 | 0.486805556 | 0.169444444 | 0 | IRX+ |
| CCND2 | 0 | 1.05021012033311 | 0.449305556 | 0.140972222 | 0 | IRX+ |
| POU3F2 | 0 | 0.945701527111192 | 0.559027778 | 0.192361111 | 0 | IRX+ |
| CRNDE | 0 | 0.866948199936887 | 0.51875 | 0.143055556 | 0 | IRX+ |
| IRX2 | 0 | 0.866304390272955 | 0.433333333 | 0.054 | 0 | IRX+ |
| NR2F2 | 0 | 0.816811389516567 | 0.40625 | 0.045 | 0 | IRX+ |
| SOX3 | 0 | 0.710218114392532 | 0.325 | 0.089 | 0 | IRX+ |
| AL359091.1 | 0 | 0.682046708507277 | 00:38 | 0.007 | 0 | IRX+ |
| IRX3 | 0 | 0.582898677189585 | 0.259027778 | 0.007 | 0 | IRX+ |
| GREM2 | 0 | 0.577081157321026 | 0.214583333 | 0.032 | 0 | IRX+ |
| IRX5 | 0 | 0.535119630515129 | 00:38 | 0.007 | 0 | IRX+ |
| WLS | 0 | 0.514988910341136 | 0.247916667 | 0.028 | 0 | IRX+ |
| SOX21 | 0 | 0.498284813331267 | 0.28125 | 0.082 | 0 | IRX+ |
| GFRA1 | 0 | 0.487408633467849 | 0.270138889 | 0.035 | 0 | IRX+ |
| IRX1 | 0 | 0.487235106300648 | 0.254861111 | 0.009 | 0 | IRX+ |
| C5orf38 | 0 | 0.476764685202968 | 0.276388889 | 0.026 | 0 | IRX+ |
| POU3F4 | 0 | 0.454067007248761 | 0.258333333 | 0.027 | 0 | IRX+ |
| SPSB4 | 0 | 0.382078701565678 | 0.245138889 | 0.059 | 0 | IRX+ |
| GPC4 | 0 | 0.375213090218054 | 0.197222222 | 0.034 | 0 | IRX+ |
| PDPN | 0 | 0.367907975000002 | 0.202083333 | 00:01 | 0 | IRX+ |
| LITAF | 0 | 0.32564337112467 | 0.184027778 | 00:01 | 0 | IRX+ |
| MIR99AHG | 0 | 0.313109740889766 | 0.215972222 | 0.037 | 0 | IRX+ |
| BMP7 | 0 | 0.27533762382196 | 0.183333333 | 0.033 | 0 | IRX+ |
| SFRP2 | 2.46169996961924e-303 | 1.53531464570134 | 0.281944444 | 0.088 | 5.11122764692043e-299 | IRX+ |
| NHSL1 | 7.25489743390652e-274 | 0.423282330470939 | 0.277083333 | 0.087 | 1.50633435420201e-269 | IRX+ |
| RFX4 | 1.13618219020673e-271 | 0.304225660320362 | 0.188888889 | 00:04 | 2.35905508152624e-267 | IRX+ |
| SOX21-AS1 | 2.18434253998282e-243 | 0.366131424438214 | 0.224305556 | 0.064 | 4.53535041576632e-239 | IRX+ |
| CD99 | 4.28844430679297e-241 | 0.742179437220171 | 0.348611111 | 0.1125 | 8.90409691419424e-237 | IRX+ |
| ARL4A | 1.70302115578906e-240 | 0.72456380197281 | 0.391666667 | 0.141666667 | 3.53598282576483e-236 | IRX+ |

| RGS20 | 8.45064917660146e-238 | 0.620773226926633 | 0.335416667 | 0.102777778 | 1.75460828853776e-233 | IRX+ |
| --- | --- | --- | --- | --- | --- | --- |
| THSD7A | 1.00975096222709e-234 | 1.02624552762864 | 0.398611111 | 00:22 | 2.09654592287211e-230 | IRX+ |
| TPD52 | 8.34076139464886e-225 | 0.543114924663247 | 0.254166667 | 0.087 | 1.73179228837094e-220 | IRX+ |
| IGDCC3 | 2.87988520376322e-224 | 0.696504094436378 | 0.494444444 | 0.235416667 | 5.97950564857357e-220 | IRX+ |
| GYPC | 6.46285651240283e-220 | 0.478923674198132 | 0.285416667 | 0.077083333 | 1.3418828976702e-215 | IRX+ |
| TCF12 | 7.75296371709899e-218 | 0.708948710451612 | 0.445138889 | 0.202083333 | 1.60974785658126e-213 | IRX+ |
| ROBO3 | 1.95293802090543e-201 | 1.05038760186238 | 0.209722222 | 0.069 | 4.05488521280595e-197 | IRX+ |
| GNG11 | 3.41690309552203e-198 | 0.363615847960472 | 0.21875 | 0.072 | 7.09451589723239e-194 | IRX+ |
| PTX3 | 4.20377639071774e-197 | 0.941220212645907 | 0.290277778 | 0.089583333 | 8.72830092004724e-193 | IRX+ |
| BTG1 | 1.7603062152743e-195 | 0.862709619844372 | 0.653472222 | 0.558333333 | 3.65492379477403e-191 | IRX+ |
| KLHL13 | 1.42326548866531e-189 | 0.302967260541882 | 0.202083333 | 0.062 | 2.95512613411579e-185 | IRX+ |
| ST18 | 4.93262109286855e-189 | 0.583627134816892 | 0.261111111 | 0.074305556 | 1.0241601175123e-184 | IRX+ |
| AC022639.1 | 2.31041912422295e-184 | 0.29102116367888 | 0.199305556 | 0.064 | 4.79712322762411e-180 | IRX+ |
| COTL1 | 4.61756140512463e-181 | 0.970271178522058 | 0.545833333 | 0.359027778 | 9.58744274546028e-177 | IRX+ |
| DTX4 | 4.96858036096212e-177 | 0.274268437986941 | 0.191666667 | 00:06 | 1.03162634034656e-172 | IRX+ |
| SPATS2L | 8.64851847060047e-171 | 0.274065433441333 | 0.179166667 | 0.055 | 1.79569189005077e-166 | IRX+ |
| EFNB2 | 1.03059328863425e-168 | 0.440382199219494 | 0.335416667 | 0.120138889 | 2.13982084519129e-164 | IRX+ |
| PRTG | 5.20825973188375e-165 | 0.445739900385365 | 0.309722222 | 0.110416667 | 1.08139096813102e-160 | IRX+ |
| ARID5B | 6.62824631844895e-160 | 0.3713144786607 | 0.233333333 | 0.094 | 1.37622278309956e-155 | IRX+ |
| GNG5 | 6.75764206619765e-150 | 0.633099258353505 | 0.616666667 | 0.403472222 | 1.40308922220462e-145 | IRX+ |
| RPS25 | 1.6040129731422e-147 | 0.306846456461785 | 1 | 0.69375 | 3.33041213613515e-143 | IRX+ |
| GCSH | 2.25326994901025e-147 | 0.485583390293275 | 0.509722222 | 0.313888889 | 4.67846439512999e-143 | IRX+ |
| PAPPA | 8.66867215429304e-143 | 0.442849622573558 | 0.179861111 | 0.065 | 1.79987639939586e-138 | IRX+ |
| GPM6B | 5.15079529861792e-139 | 0.577769290966726 | 0.551388889 | 0.402083333 | 1.06945962785204e-134 | IRX+ |
| ZEB2 | 1.93387222094808e-130 | 0.393777533316163 | 0.351388889 | 00:22 | 4.0152988923545e-126 | IRX+ |
| PBX3 | 3.59343462455556e-122 | 0.631850323279553 | 0.524305556 | 0.339583333 | 7.46104831096471e-118 | IRX+ |
| PHLDA1 | 1.55143794967896e-120 | 0.547638781442748 | 0.248611111 | 0.089583333 | 3.22125061491843e-116 | IRX+ |
| TMEM47 | 1.21587595511203e-119 | 0.5524800125581 | 0.475694444 | 0.307638889 | 2.52452324559911e-115 | IRX+ |
| NKAIN4 | 4.0432052353385e-115 | 0.462457724544412 | 0.054861111 | 0.330555556 | 8.39490703013333e-111 | IRX+ |
| FGFBP3 | 8.46476830629736e-114 | 0.518486029536553 | 0.2875 | 0.122916667 | 1.75753984343652e-109 | IRX+ |
| RPS6 | 8.78033585418467e-112 | 0.353955455068193 | 0.690972222 | 0.692361111 | 1.82306113340436e-107 | IRX+ |
| EBF2 | 9.81345128015298e-111 | 0.371679447110258 | 0.179861111 | 0.078 | 2.03756688929816e-106 | IRX+ |
| FAT3 | 4.06699592415214e-109 | 0.300647775851887 | 0.248611111 | 0.089583333 | 8.44430363731708e-105 | IRX+ |
| CRYBG3 | 7.18853453459024e-109 | 0.586649349991562 | 0.264583333 | 0.107638889 | 1.49255542541697e-104 | IRX+ |
| RPS8 | 8.80239934676703e-107 | 0.250706512771053 | 0.69375 | 1 | 1.82764217636924e-102 | IRX+ |
| RPL19 | 1.09563520802425e-103 | 0.270226262629436 | 0.693055556 | 0.69375 | 2.27486738242075e-99 | IRX+ |
| ATP5MC2 | 1.59330161758988e-100 | 0.273667715925737 | 0.69375 | 0.690972222 | 3.30817214860186e-96 | IRX+ |
| CDKN1C | 6.94169284813077e-98 | 0.655159976966175 | 0.661805556 | 0.584027778 | 1.44130368605739e-93 | IRX+ |
| RPL18A | 1.58568127650032e-97 | 0.274895073703464 | 0.693055556 | 0.69375 | 3.29235003439762e-93 | IRX+ |
| TAGLN3 | 2.94550411833765e-96 | 0.735038502933972 | 0.653472222 | 0.6 | 6.11575020090447e-92 | IRX+ |
| RACK1 | 5.95732655702294e-96 | 0.259240426124313 | 0.69375 | 0.693055556 | 1.23691971303467e-91 | IRX+ |
| SLC25A5 | 4.32073226265089e-94 | 0.549030615753868 | 0.654166667 | 0.596527778 | 8.97113639694204e-90 | IRX+ |
| AKAP12 | 5.33704361649098e-92 | 0.617430612185999 | 0.379861111 | 0.216666667 | 1.10813036609202e-87 | IRX+ |
| MIAT | 1.68718260113087e-91 | 0.599716680731798 | 0.568055556 | 0.047222222 | 3.50309723472803e-87 | IRX+ |
| CCNB1IP1 | 2.51024100227506e-91 | 0.361188469438803 | 0.460416667 | 0.272222222 | 5.2120133930237e-87 | IRX+ |
| RPS3 | 2.77770237512028e-91 | 0.254891468931281 | 0.69375 | 1 | 5.76734344146224e-87 | IRX+ |
| DNAJC1 | 4.35511722672254e-91 | 0.404007273040855 | 0.372222222 | 0.20625 | 9.04252989784402e-87 | IRX+ |
| EIF4EBP1 | 4.03806757180074e-90 | 0.359151842530744 | 00:36 | 0.102777778 | 8.38423969932989e-86 | IRX+ |
| RPS7 | 8.54676020733752e-87 | 0.27187512152102 | 0.69375 | 0.69375 | 1.77456382184949e-82 | IRX+ |
| ID2 | 9.14568514902953e-86 | 0.369208856234248 | 0.265972222 | 00:17 | 1.898918607493e-81 | IRX+ |
| RAI14 | 1.86904852485429e-85 | 0.330412708717871 | 0.207638889 | 0.079861111 | 3.88070545215497e-81 | IRX+ |
| ENC1 | 1.67166934803875e-84 | 0.498338757872745 | 0.388194444 | 00:31 | 3.47088706733285e-80 | IRX+ |
| PCDH19 | 9.25947887144856e-83 | 0.356907471199893 | 00:35 | 00:15 | 1.92254559807887e-78 | IRX+ |
| RBM3 | 1.65887782877764e-81 | 0.345750489000487 | 0.554166667 | 0.415972222 | 3.44432803589101e-77 | IRX+ |
| RPL36AL | 9.75854168899114e-79 | 0.277605729298572 | 0.671527778 | 0.654166667 | 2.02616601088523e-74 | IRX+ |
| EBF3 | 4.74018824352983e-78 | 0.36596562820709 | 0.247916667 | 0.113194444 | 9.842052850041e-74 | IRX+ |
| EBF1 | 3.30377902932202e-77 | 0.536795186843765 | 0.301388889 | 0.160416667 | 6.8596363985813e-73 | IRX+ |
| AL391807.1 | 6.69833771092147e-77 | 0.264542640888842 | 00:03 | 0.08125 | 1.39077585891862e-72 | IRX+ |
| ZFP36L1 | 3.73857841491687e-75 | 0.538619133980313 | 0.228472222 | 0.098611111 | 7.7624103628919e-71 | IRX+ |
| CITED2 | 4.77288899363231e-75 | 0.402850744840656 | 0.380555556 | 00:32 | 9.90994941747877e-71 | IRX+ |
| SLC25A6 | 7.42868136581104e-75 | 0.254119060344021 | 0.683333333 | 0.672916667 | 1.54241711198335e-70 | IRX+ |
| NES | 7.73962267693177e-74 | 0.562182810656875 | 0.459027778 | 0.340972222 | 1.60697785641134e-69 | IRX+ |
| SOX2 | 2.09862837248896e-72 | 0.40565659008302 | 0.322916667 | 00:24 | 4.35738208979882e-68 | IRX+ |
| CALD1 | 2.77982619315783e-72 | 0.339450237872135 | 0.370138889 | 0.209722222 | 5.7717531248536e-68 | IRX+ |
| BTG2 | 9.10694683467336e-70 | 0.395396962431727 | 0.202777778 | 0.085416667 | 1.89087537128323e-65 | IRX+ |
| RPL23A | 2.36591326690508e-68 | 0.272719258645375 | 0.690972222 | 0.689583333 | 4.91234571607503e-64 | IRX+ |
| SIPA1L2 | 1.54820235198657e-67 | 0.252384150511798 | 0.251388889 | 0.117361111 | 3.21453254342971e-63 | IRX+ |
| SRGAP3 | 4.26850636381722e-67 | 0.482027577673102 | 0.570833333 | 0.488194444 | 8.86269976319369e-63 | IRX+ |
| RPS20 | 1.23985008561342e-66 | 0.301318688912891 | 0.689583333 | 0.685416667 | 2.57430073275915e-62 | IRX+ |
| CALM1 | 6.15371148476139e-66 | 0.420419458440768 | 0.683333333 | 0.681944444 | 1.27769511558101e-61 | IRX+ |
| PHGDH | 1.72506045188798e-65 | 0.31679286730264 | 0.249305556 | 0.117361111 | 3.581743016255e-61 | IRX+ |
| TBCA | 2.44208889172592e-65 | 0.400004154045994 | 0.683333333 | 0.670138889 | 5.07050916589052e-61 | IRX+ |
| IGFBP5 | 7.92538663909591e-64 | 1.00368339798739 | 0.174305556 | 0.071527778 | 1.64554802787548e-59 | IRX+ |
| RGMB | 1.01666740778714e-63 | 0.419315360748308 | 0.345138889 | 0.203472222 | 2.11090653878844e-59 | IRX+ |
| TOX3 | 2.7785759853359e-62 | 0.314246329378884 | 0.471527778 | 00:47 | 5.76915731835293e-58 | IRX+ |
| HNRNPDL | 1.97629301217378e-61 | 0.278752074053253 | 0.688888889 | 0.682638889 | 4.10337718117643e-57 | IRX+ |
| IFITM3 | 6.84436194724476e-61 | 0.457947877310494 | 0.220138889 | 0.10625 | 1.42109487110643e-56 | IRX+ |
| RHOBTB3 | 8.73092846355171e-57 | 0.292653635899988 | 0.483333333 | 0.357638889 | 1.81280267688724e-52 | IRX+ |
| HNRNPA1 | 1.02225913723632e-56 | 0.261646920570498 | 0.691666667 | 0.686805556 | 2.12251664664377e-52 | IRX+ |
| ANP32B | 2.10102586793827e-56 | 0.341481225149094 | 0.567361111 | 0.461805556 | 4.36236000960024e-52 | IRX+ |
| ARHGAP21 | 3.95546224861598e-56 | 0.328590764682124 | 0.552777778 | 0.44375 | 8.21272626680137e-52 | IRX+ |
| IGF2BP3 | 5.19149148069048e-55 | 0.315001884218237 | 0.469444444 | 0.359027778 | 1.07790937613576e-50 | IRX+ |
| RPL10A | 1.38550993303271e-53 | 0.255974437015818 | 0.686111111 | 0.683333333 | 2.87673427395581e-49 | IRX+ |
| AFDN | 1.63788230178501e-53 | 0.32752158580173 | 0.582638889 | 0.051388889 | 3.40073502319622e-49 | IRX+ |
| CCND1 | 2.19986971373722e-52 | 0.615036674247624 | 0.218055556 | 0.106944444 | 4.5675894866326e-48 | IRX+ |
| IER2 | 6.0638201759455e-51 | 0.390607802831125 | 0.308333333 | 0.186805556 | 1.25903098313156e-46 | IRX+ |
| RPLP0 | 4.31634165562701e-49 | 0.269162145342439 | 0.691666667 | 0.690277778 | 8.96202017957836e-45 | IRX+ |
| GSTP1 | 1.68234042038182e-48 | 0.376461449360718 | 0.623611111 | 0.573611111 | 3.49304341483878e-44 | IRX+ |
| NME1 | 4.92661544793887e-48 | 0.293167437775186 | 0.456944444 | 0.345138889 | 1.02291316545555e-43 | IRX+ |
| MSI2 | 4.44105899485508e-47 | 0.319465692054935 | 0.479861111 | 0.369444444 | 9.22097079101761e-43 | IRX+ |
| VIM | 1.9551525241637e-46 | 1.12232237282738 | 0.591666667 | 0.567361111 | 4.05948318592109e-42 | IRX+ |
| CRB2 | 3.31143941859885e-46 | 0.279737937262256 | 0.307638889 | 0.190972222 | 6.87554166483679e-42 | IRX+ |
| FNDC5 | 2.93744184356558e-44 | 0.258975628587339 | 0.204166667 | 00:15 | 6.09901049979522e-40 | IRX+ |
| PTPRZ1 | 3.50173274423931e-44 | 0.426452297739906 | 0.41875 | 0.325694444 | 7.27064769686408e-40 | IRX+ |
| FGFR1 | 3.36142848786568e-42 | 0.284706847248173 | 0.293055556 | 00:27 | 6.9793339693555e-38 | IRX+ |
| RPS27L | 3.46565661592487e-41 | 0.365842638854783 | 0.5875 | 0.50625 | 7.19574283164481e-37 | IRX+ |
| KCNQ1OT1 | 1.69076987035891e-40 | 0.392001008909316 | 0.623611111 | 0.561111111 | 3.51054548182621e-36 | IRX+ |
| ANK2 | 4.41794207211721e-39 | 0.352140394986657 | 0.276388889 | 0.175694444 | 9.17297312433695e-35 | IRX+ |
| INSM1 | 7.80448178733357e-39 | 0.413550023513236 | 0.495138889 | 0.41875 | 1.62044455350407e-34 | IRX+ |
| ANXA5 | 2.25029213580129e-38 | 0.291733922080519 | 0.397222222 | 0.290277778 | 4.67228156156422e-34 | IRX+ |

| RAB13 | 1.94562000506005e-37 | 0.313900034328087 | 0.304166667 | 0.200694444 | 4.03969081650618e-33 | IRX+ |
| --- | --- | --- | --- | --- | --- | --- |
| SVIP | 4.79579289887954e-37 | 0.364852784823484 | 0.302083333 | 00:29 | 9.95750479594359e-33 | IRX+ |
| QKI | 1.38520415062514e-34 | 0.421336128748505 | 0.355555556 | 0.28125 | 2.87609937794298e-30 | IRX+ |
| EPB41 | 5.32435317208742e-34 | 0.526086670229979 | 0.581944444 | 0.552083333 | 1.10549544912051e-29 | IRX+ |
| PON2 | 6.50955251999024e-33 | 0.332536375068769 | 0.350694444 | 0.259722222 | 1.35157838972557e-28 | IRX+ |
| ENO1 | 2.13902626899017e-32 | 0.250006783238987 | 0.452777778 | 0.363888889 | 4.44126024230429e-28 | IRX+ |
| AC008522.1 | 1.09298016017821e-31 | 0.341530087118848 | 0.238888889 | 0.146527778 | 2.26935470657802e-27 | IRX+ |
| MIR124-2HG | 1.79663005743277e-31 | 0.269066725930223 | 0.367361111 | 00:39 | 3.73034298824766e-27 | IRX+ |
| SRRM4 | 3.95804863755078e-30 | 0.33154247543788 | 0.440277778 | 0.367361111 | 8.21809638614668e-26 | IRX+ |
| SPARC | 4.8774231787398e-29 | 0.269517825082916 | 0.225 | 0.140277778 | 1.01269937460175e-24 | IRX+ |
| KLHL35 | 7.31034112206085e-27 | 0.314561629592359 | 0.251388889 | 0.170833333 | 1.51784612717349e-22 | IRX+ |
| NCALD | 6.03749739807277e-25 | 0.334533826892512 | 0.297916667 | 0.227777778 | 1.25356558476185e-20 | IRX+ |
| CAMK2N1 | 2.16023017867122e-24 | 0.304218748315874 | 0.624305556 | 0.590972222 | 4.48528591997505e-20 | IRX+ |
| UNCX | 4.84329219668398e-23 | 0.296793048543664 | 0.209027778 | 0.1375 | 1.00561275879749e-18 | IRX+ |
| IER5L | 1.11107581825199e-22 | 0.255931921420796 | 0.279166667 | 0.200694444 | 2.30692672143661e-18 | IRX+ |
| SCRT1 | 9.10351349393251e-22 | 0.286243359719098 | 00:31 | 0.146527778 | 1.89016250674521e-17 | IRX+ |
| CNTN2 | 3.96390897900691e-20 | 0.403936292388902 | 0.228472222 | 0.163888889 | 8.23026421311205e-16 | IRX+ |
| PTPRG | 5.04723943733742e-17 | 0.317671711331373 | 0.321527778 | 0.266666667 | 1.04795832437437e-12 | IRX+ |
| PRSS23 | 3.93843252484074e-15 | 0.412015750933826 | 00:47 | 0.258333333 | 8.17736745132682e-11 | IRX+ |
| NIN | 1.19446904277282e-13 | 0.260951852676252 | 0.283333333 | 0.226388889 | 2.48007607350921e-09 | IRX+ |
| NEFL | 1.22829184123694e-13 | 0.691196461646181 | 0.461111111 | 0.51875 | 2.55030234996025e-09 | IRX+ |
| GADD45G | 7.41894031437838e-13 | 0.330775552693014 | 0.301388889 | 0.241666667 | 1.54039457747438e-08 | IRX+ |
| SFRP1 | 3.40319618134151e-12 | 0.454189379459374 | 0.290277778 | 0.261111111 | 7.06605623131938e-08 | IRX+ |
| NEFM | 6.63446589448944e-12 | 0.798210642258199 | 0.446527778 | 0.531944444 | 1.37751415367284e-07 | IRX+ |
| HERPUD1 | 3.39660897124708e-08 | 0.257989835840973 | 0.273611111 | 0.238194444 | 0.00070523792070003 | IRX+ |
| DLL3 | 4.37430931088537e-08 | 0.456156126750374 | 0.38125 | 0.353472222 | 0.000908237842219129 | IRX+ |
