# Supplementary material for: Deciphering the Transcriptional Landscape of Human Pluripotent Stem Cell-Derived GnRH Neurons: The Role of Wnt Signaling in Patterning the Neural Fate
Source: Stem Cells. 2022 Sep 25;40(12):1107–21. doi: 10.1093/stmcls/sxac069 (PMC9806769; doi:10.1093/stmcls/sxac069)
Supplement: sxac069_suppl_Supplementary_Table_S2 [file sxac069_suppl_supplementary_table_s2.docx]

|  | id | module | supermodule | dim_1 | dim_2 |
| --- | --- | --- | --- | --- | --- |
| 1 | AL669831.5 | 1 | 1 | 2.24877239744298 | 1.39115156310629 |
| 2 | AL645608.3 | 1 | 1 | 4.10403133909337 | 0.685803548274743 |
| 3 | AL645608.1 | 15 | 1 | 5.94284702818029 | 0.592365280566919 |
| 4 | SAMD11 | 15 | 1 | 5.91907574216954 | 0.6552841820104 |
| 5 | NOC2L | 1 | 1 | 1.31999064962499 | 1.58636821883749 |
| 6 | HES4 | 10 | 1 | 3.7104489950382 | -1.96913669926096 |
| 7 | ISG15 | 14 | 1 | -5.45231698472865 | -0.842721331091416 |
| 8 | AGRN | 6 | 1 | 2.32606150190465 | -2.06158123833109 |
| 9 | C1orf159 | 2 | 1 | -0.106005415514781 | 1.84763111251425 |
| 10 | TTLL10-AS1 | 6 | 1 | 2.30514027158849 | -2.02943752628733 |
| 11 | TTLL10 | 6 | 1 | 1.25257708112829 | -2.88137327057291 |
| 12 | SDF4 | 3 | 1 | -1.95517002065547 | -0.300278826774848 |
| 13 | B3GALT6 | 17 | 1 | -0.139916151956393 | 0.0683898486478208 |
| 14 | UBE2J2 | 13 | 1 | -1.03543971498378 | -0.607887908043158 |
| 15 | ACAP3 | 1 | 1 | 2.14246083776586 | 1.08515824454855 |
| 16 | PUSL1 | 7 | 1 | -2.91304682214625 | -2.92607484680582 |
| 17 | INTS11 | 13 | 1 | -1.62174557168849 | -0.824072799803507 |
| 18 | MXRA8 | 7 | 1 | -2.75174163301356 | -3.33959327560831 |
| 19 | AURKAIP1 | 13 | 1 | -0.755333885744884 | -1.94267831665446 |
| 20 | CCNL2 | 2 | 1 | 0.46028651277654 | 1.40845049995016 |
| 21 | MRPL20 | 13 | 1 | -1.0919875951565 | -1.81946359497477 |
| 22 | ANKRD65 | 7 | 1 | -2.42840479333766 | -3.72557912689616 |
| 23 | VWA1 | 4 | 1 | -4.96879266221888 | -2.40059648376871 |
| 24 | ATAD3B | 3 | 1 | -1.92031584699519 | -0.0910327873842837 |
| 25 | ATAD3A | 3 | 1 | -1.9290066810406 | 0.307315484462488 |
| 26 | TMEM240 | 5 | 1 | 3.64596010725133 | -0.908440993325603 |
| 27 | SSU72 | 6 | 1 | 1.58833040277593 | -1.49884752851893 |
| 28 | AL645728.1 | 4 | 1 | -3.63274692018397 | 0.25980307239126 |
| 29 | FNDC10 | 5 | 1 | 4.28661705534093 | -0.730971514405501 |
| 30 | AL691432.2 | 17 | 1 | 1.91134311239354 | 0.501381889758813 |
| 31 | CDK11B | 2 | 1 | 0.734802260800527 | 1.24467982429098 |
| 32 | SLC35E2B | 12 | 1 | 2.3142657427036 | 2.93984784263204 |
| 33 | SLC35E2A | 1 | 1 | 3.8333099035465 | 2.21992220061849 |
| 34 | GNB1 | 1 | 1 | 3.47972895185582 | 1.6882523455007 |
| 35 | TMEM52 | 13 | 1 | -1.61899958570368 | -2.48950003010203 |
| 36 | CFAP74 | 4 | 1 | -4.24336574991114 | 0.0551026024205564 |
| 37 | GABRD | 11 | 1 | -3.50127504785426 | -1.9222019754069 |
| 38 | PRKCZ | 10 | 1 | 4.15306021253698 | -1.21582205516268 |
| 39 | FAAP20 | 11 | 1 | -2.72369430978663 | -1.57947949987818 |
| 40 | SKI | 12 | 1 | 2.36833359281652 | 2.10210765975546 |
| 41 | MORN1 | 3 | 1 | -2.33778356035121 | 0.611383692203271 |
| 42 | RER1 | 13 | 1 | -2.44419883211024 | -1.71425770145823 |
| 43 | PEX10 | 3 | 1 | -1.91950820405848 | -0.698345719994795 |
| 44 | PLCH2 | 7 | 1 | -2.54439447839625 | -3.85461817604471 |
| 45 | PANK4 | 2 | 1 | 0.278261974259541 | 1.02127184051107 |
| 46 | HES5 | 4 | 1 | -4.58490251024134 | -1.54200051647593 |
| 47 | AL139246.5 | 4 | 1 | -4.70301650483973 | -1.65508280617167 |
| 48 | FAM213B | 6 | 1 | 2.25196005384557 | -1.82724927288462 |
| 49 | AL831784.1 | 11 | 1 | -4.34535907228358 | -1.52969990593363 |
| 50 | LINC00982 | 4 | 1 | -4.75642608125575 | -1.05291500610281 |
| 51 | PRDM16 | 4 | 1 | -4.6845614763058 | -1.26237542849947 |
| 52 | AL590438.1 | 4 | 1 | -4.51507018525965 | -1.43827174526621 |
| 53 | MEGF6 | 7 | 1 | -3.39026688058741 | -2.59815965515543 |
| 54 | TPRG1L | 5 | 1 | 3.24775744955175 | 0.0158035911900877 |
| 55 | WRAP73 | 7 | 1 | -2.39121197183497 | -2.39206896644999 |
| 56 | TP73 | 13 | 1 | -0.567614302233531 | -3.35567961555887 |
| 57 | SMIM1 | 11 | 1 | -3.08343099077113 | -2.02174101692606 |
| 58 | LRRC47 | 2 | 1 | -0.0799681695259352 | 1.3087390817983 |
| 59 | CEP104 | 2 | 1 | 0.645705058976338 | 2.17922414916586 |
| 60 | C1orf174 | 3 | 1 | -3.68041966874964 | 1.12973131316732 |
| 61 | AJAP1 | 18 | 1 | 6.27600552122228 | -2.38870297295023 |
| 62 | KCNAB2 | 5 | 1 | 4.18745256940954 | -0.776881358744395 |
| 63 | CHD5 | 18 | 1 | 6.07216908018224 | -1.81503336054255 |
| 64 | RPL22 | 21 | 1 | -0.84895889480479 | -4.41466604095865 |
| 65 | ICMT | 16 | 1 | -3.53452824075587 | 1.92513455527853 |
| 66 | GPR153 | 6 | 1 | 2.30838872472875 | -2.41959701401163 |

| 67 | ACOT7 | 10 | 1 | 4.35257269422643 | -1.35234357339312 |
| --- | --- | --- | --- | --- | --- |
| 68 | ESPN | 7 | 1 | -3.39807222803004 | -2.64530847889353 |
| 69 | TNFRSF25 | 7 | 1 | -2.56435321290858 | -3.30383239609171 |
| 70 | PLEKHG5 | 7 | 1 | -2.92616842706568 | -3.44954977852274 |
| 71 | NOL9 | 2 | 1 | -0.375981733397319 | 2.07080544608663 |
| 72 | KLHL21 | 4 | 1 | -4.46696303804286 | -1.27542586547304 |
| 73 | PHF13 | 9 | 1 | -0.0784788282669325 | 3.03465165275167 |
| 74 | THAP3 | 8 | 1 | -0.712211534575297 | -0.138819678844702 |
| 75 | DNAJC11 | 2 | 1 | -0.775213167265727 | 0.941079393802392 |
| 76 | CAMTA1 | 4 | 1 | -3.65057824571498 | 0.438319221912134 |
| 77 | VAMP3 | 11 | 1 | -3.60498021562464 | -2.09005282741953 |
| 78 | PER3 | 12 | 1 | 2.83401729147069 | 2.2249246992452 |
| 79 | PARK7 | 13 | 1 | -0.65416176636584 | -2.02645300251414 |
| 80 | ERRFI1 | 15 | 1 | 4.68923784772985 | -0.327620311798346 |
| 81 | SLC45A1 | 10 | 1 | 4.59937550108068 | -0.725929408492339 |
| 82 | RERE | 5 | 1 | 3.1151542810642 | -0.0887569628374697 |
| 83 | ENO1 | 4 | 1 | -3.42728327234156 | -0.310042306007636 |
| 84 | MIR34AHG | 13 | 1 | -0.476469040111377 | -3.26288495880533 |
| 85 | LINC01759 | 11 | 1 | -3.20169542749293 | -2.31103323322703 |
| 86 | H6PD | 8 | 1 | -2.13011728246577 | 1.47433818954061 |
| 87 | AL928921.2 | 4 | 1 | -4.15250466783412 | 0.266334430156457 |
| 88 | SLC25A33 | 17 | 1 | 0.124426588221715 | 0.201982871471155 |
| 89 | PIK3CD | 6 | 1 | 2.37883473913305 | -1.79710327011515 |
| 90 | PIK3CD-AS2 | 13 | 1 | -1.31757341344722 | -2.80587886196543 |
| 91 | CLSTN1 | 1 | 1 | 4.17190529386632 | 1.4332474626882 |
| 92 | CTNNBIP1 | 6 | 1 | 1.20470906774633 | -1.48091648441721 |
| 93 | LZIC | 8 | 1 | -2.19398473222621 | 1.13022126334738 |
| 94 | NMNAT1 | 3 | 1 | -2.25517343004115 | 0.359598294673669 |
| 95 | RBP7 | 5 | 1 | 3.89512850324743 | -0.817612863303912 |
| 96 | UBE4B | 12 | 1 | 2.72382570783727 | 2.54242290633749 |
| 97 | KIF1B | 1 | 1 | 4.42233158628576 | 0.910485044894922 |
| 98 | AL139424.3 | 1 | 1 | 3.26293875257604 | 0.661594168124902 |
| 99 | AL139424.2 | 5 | 1 | 3.11277200262182 | 0.326725975452173 |
| 100 | PGD | 7 | 1 | -2.85606168229945 | -1.93891666752268 |
| 101 | AL139424.1 | 19 | 1 | -4.7890536638058 | 1.16361965316366 |
| 102 | CENPS-CORT | 19 | 1 | -4.15425919015773 | 1.45238031524252 |
| 103 | CENPS | 19 | 1 | -3.98165939767726 | 1.04892756122183 |
| 104 | DFFA | 9 | 1 | 1.71609915773504 | 3.03097237723898 |
| 105 | CASZ1 | 7 | 1 | -3.47649119813807 | -2.64216552597452 |
| 106 | AL139423.1 | 11 | 1 | -3.60491560418971 | -1.88568566662241 |
| 107 | TARDBP | 8 | 1 | -1.70064769704707 | 1.65220607894491 |
| 108 | SRM | 3 | 1 | -2.31350563485987 | -0.822053744615805 |
| 109 | EXOSC10 | 9 | 1 | -0.143992558316066 | 2.71315492766927 |
| 110 | AL109811.2 | 11 | 1 | -3.42458437402613 | -1.55897526365687 |
| 111 | MTOR | 9 | 1 | 0.338740721150563 | 2.86553563254904 |
| 112 | UBIAD1 | 2 | 1 | -0.175504170552566 | 1.55011810439657 |
| 113 | DISP3 | 7 | 1 | -2.44986461122401 | -3.80763898712565 |
| 114 | FBXO2 | 6 | 1 | 2.34844900648229 | -2.12784920555521 |
| 115 | FBXO44 | 5 | 1 | 4.58681751768224 | -0.467867090524924 |
| 116 | FBXO6 | 1 | 1 | 2.17500711958043 | 0.999316708026636 |
| 117 | MAD2L2 | 6 | 1 | -0.135749541417434 | -1.69419221502711 |
| 118 | DRAXIN | 7 | 1 | -2.40755508859523 | -3.90241585594584 |
| 119 | AGTRAP | 4 | 1 | -4.09088300187953 | -1.0392462454455 |
| 120 | C1orf167 | 15 | 1 | 5.81969954053991 | 0.658374444423425 |
| 121 | MTHFR | 12 | 1 | 2.07445551435582 | 1.91108621734213 |
| 122 | CLCN6 | 1 | 1 | 2.88644339124791 | 1.55846919196676 |
| 123 | NPPA | 15 | 1 | 5.69685961286657 | -0.00476299148966086 |
| 124 | KIAA2013 | 8 | 1 | -1.47966419179805 | -0.0203364930765749 |
| 125 | PLOD1 | 14 | 1 | -5.62668250520594 | -0.533900573076499 |
| 126 | MFN2 | 12 | 1 | 2.86187054197423 | 2.98676909583639 |
| 127 | MIIP | 17 | 1 | 1.40189374963872 | -0.583970501245749 |
| 128 | VPS13D | 12 | 1 | 3.45294715444677 | 2.75946463721822 |
| 129 | DHRS3 | 14 | 1 | -6.92158101518519 | -0.653139799060118 |
| 130 | PDPN | 14 | 1 | -6.83929799516566 | -0.749241619529021 |
| 131 | PRDM2 | 12 | 1 | 1.99682857076757 | 2.67261184829305 |
| 132 | KAZN | 15 | 1 | 5.00877310316198 | -0.335204227985633 |
| 133 | TMEM51 | 7 | 1 | -3.99177144487269 | -2.96463619095255 |

| 134 | FHAD1 | 7 | 1 | -3.11540697534449 | -2.85277043205668 |
| --- | --- | --- | --- | --- | --- |
| 135 | EFHD2 | 5 | 1 | 2.95095802824132 | -0.263542457641852 |
| 136 | CASP9 | 11 | 1 | -3.00994823892482 | -2.19893501621653 |
| 137 | DNAJC16 | 2 | 1 | 0.468559399052785 | 1.67533530372213 |
| 138 | DDI2 | 9 | 1 | -0.0635528119362135 | 2.69241680282186 |
| 139 | PLEKHM2 | 2 | 1 | 0.00542460362546249 | 1.72442282813619 |
| 140 | FBLIM1 | 18 | 1 | 5.90196253339879 | -1.6153234682696 |
| 141 | UQCRHL | 2 | 1 | -0.98030518014796 | 1.98185457366537 |
| 142 | AL450998.2 | 5 | 1 | 3.91415192167394 | -0.947587235988867 |
| 143 | SPEN | 12 | 1 | 2.52856899778478 | 3.01442326682638 |
| 144 | ZBTB17 | 8 | 1 | -1.12898151595957 | 0.00552882331441629 |
| 145 | FAM131C | 6 | 1 | 1.14007510225408 | -2.95102272850443 |
| 146 | EPHA2 | 7 | 1 | -3.9755062909878 | -2.62147401195933 |
| 147 | ARHGEF19 | 3 | 1 | -2.96099804361232 | -0.812665178598654 |
| 148 | FBXO42 | 12 | 1 | 2.66717173139684 | 2.92438782828878 |
| 149 | SZRD1 | 3 | 1 | -3.10844515283473 | -0.0520095072405459 |
| 150 | NECAP2 | 3 | 1 | -2.73069165666468 | -0.258884950699103 |
| 151 | LINC01772 | 1 | 1 | 2.5087614206516 | 1.06474615710806 |
| 152 | AL137802.2 | 15 | 1 | 5.34923960249059 | 0.0693638481480955 |
| 153 | NBPF1 | 9 | 1 | -0.521519974068477 | 3.17223347800802 |
| 154 | CROCC | 8 | 1 | -1.45400677640803 | 0.757093206821191 |
| 155 | MFAP2 | 4 | 1 | -4.31456922014125 | -1.2775699637549 |
| 156 | ATP13A2 | 15 | 1 | 4.83776022474401 | -0.510720535339606 |
| 157 | SDHB | 13 | 1 | -0.443007245854213 | -0.649559660972846 |
| 158 | RCC2 | 3 | 1 | -2.94071219880946 | 0.120644585071313 |
| 159 | ARHGEF10L | 5 | 1 | 3.86749912778966 | -0.211067780079138 |
| 160 | IGSF21 | 7 | 1 | -2.58308289964564 | -3.07006584030558 |
| 161 | PAX7 | 14 | 1 | -6.73393224199183 | -0.405087961973441 |
| 162 | ALDH4A1 | 11 | 1 | -3.34994696100123 | -1.99501489025522 |
| 163 | IFFO2 | 7 | 1 | -3.22743867357142 | -3.7582238755839 |
| 164 | UBR4 | 2 | 1 | 0.0552910415374498 | 2.25033439773153 |
| 165 | AL035413.1 | 4 | 1 | -3.90393947084315 | 0.135771051822412 |
| 166 | EMC1 | 9 | 1 | 0.78036011736028 | 2.88854159492086 |
| 167 | MRTO4 | 3 | 1 | -2.85849522073634 | -0.569070889772666 |
| 168 | AKR7A2 | 13 | 1 | -2.14910887201197 | -1.93723343235422 |
| 169 | CAPZB | 6 | 1 | 1.71158482114904 | -1.60539852005411 |
| 170 | MINOS1 | 13 | 1 | -0.222517254025414 | -2.30028842312266 |
| 171 | NBL1 | 18 | 1 | 5.50920511762731 | -2.07320915085245 |
| 172 | OTUD3 | 1 | 1 | 3.41995717565648 | 2.31520618575643 |
| 173 | UBXN10-AS1 | 5 | 1 | 4.12104298154943 | -0.585033281864417 |
| 174 | CAMK2N1 | 15 | 1 | 4.64254881422155 | 0.21210528987478 |
| 175 | MUL1 | 8 | 1 | -0.70518453438647 | -0.0890503965036989 |
| 176 | PINK1 | 6 | 1 | 2.05096270124547 | -1.20125488859583 |
| 177 | DDOST | 3 | 1 | -2.22737132032283 | -0.944253507228387 |
| 178 | HP1BP3 | 12 | 1 | 2.59082199613683 | 2.72263015883993 |
| 179 | EIF4G3 | 9 | 1 | 0.822023048802541 | 2.85225023406576 |
| 180 | ECE1 | 18 | 1 | 6.11798168699376 | -2.60640595776011 |
| 181 | NBPF3 | 16 | 1 | -4.17899940927394 | 2.77361930984091 |
| 182 | ALPL | 4 | 1 | -4.37136386354335 | -1.02629952055384 |
| 183 | USP48 | 9 | 1 | -0.366390809611156 | 3.09799398559164 |
| 184 | HSPG2 | 4 | 1 | -5.20476983507045 | -0.239272757591498 |
| 185 | CDC42 | 10 | 1 | 3.80629946272008 | -1.47746591192652 |
| 186 | WNT4 | 15 | 1 | 5.57935263197057 | 0.686239496646631 |
| 187 | ZBTB40 | 2 | 1 | -0.96687994916804 | 2.00034631866049 |
| 188 | EPHA8 | 6 | 1 | 1.82810951749914 | -2.59986697060038 |
| 189 | EPHB2 | 7 | 1 | -2.50634573419459 | -3.73878298622538 |
| 190 | KDM1A | 9 | 1 | 0.912010088368581 | 2.61377991813253 |
| 191 | LUZP1 | 4 | 1 | -4.43851970155604 | -0.0346963844912173 |
| 192 | LINC01355 | 12 | 1 | 2.07733704130285 | 2.63715495246481 |
| 193 | HNRNPR | 8 | 1 | -1.66082178075679 | 0.990023509441125 |
| 194 | ZNF436-AS1 | 3 | 1 | -2.81080435235865 | -0.0495888434069277 |
| 195 | TCEA3 | 7 | 1 | -3.31124852617152 | -3.01699672562052 |
| 196 | ASAP3 | 11 | 1 | -3.37239097078211 | -2.04397283417155 |
| 197 | E2F2 | 19 | 1 | -5.00120471437342 | 1.82587732451986 |
| 198 | ID3 | 14 | 1 | -6.63390181978114 | -0.321594937862647 |
| 199 | RPL11 | 21 | 1 | -0.655626014069392 | -4.20422516685892 |
| 200 | ELOA | 1 | 1 | 1.17843820135228 | 1.75067915099691 |

| 201 | PITHD1 | 5 | 1 | 3.24101521055333 | -0.349460109295142 |
| --- | --- | --- | --- | --- | --- |
| 202 | LYPLA2 | 13 | 1 | -1.07946465928919 | -1.13609747511317 |
| 203 | GALE | 5 | 1 | 2.68867541830175 | -0.605279727997077 |
| 204 | HMGCL | 3 | 1 | -1.92891977746852 | -0.935761186452401 |
| 205 | FUCA1 | 5 | 1 | 4.38108183424108 | -0.750316321076644 |
| 206 | PNRC2 | 3 | 1 | -3.42544196565516 | 0.760767594753015 |
| 207 | SRSF10 | 3 | 1 | -3.44967268426783 | 0.640036360202539 |
| 208 | STPG1 | 4 | 1 | -4.40079902131922 | -0.0419004402773501 |
| 209 | NIPAL3 | 12 | 1 | 2.17141915838353 | 2.21052445548605 |
| 210 | RCAN3 | 5 | 1 | 4.32202412168615 | -0.74298007708956 |
| 211 | AL445686.2 | 10 | 1 | 3.67117358724706 | -1.50413797718455 |
| 212 | SRRM1 | 3 | 1 | -2.74596498926051 | 1.08882226127218 |
| 213 | CLIC4 | 3 | 1 | -3.60581968744166 | 0.892230884013879 |
| 214 | SYF2 | 11 | 1 | -2.8068497033871 | -1.57446388822962 |
| 215 | RSRP1 | 2 | 1 | 0.64811678211324 | 1.38032497542928 |
| 216 | RHD | 7 | 1 | -2.26699851472743 | -2.75830791813303 |
| 217 | TMEM50A | 13 | 1 | -0.491843342975452 | -2.41703401428629 |
| 218 | RHCE | 10 | 1 | 2.93989850561254 | -1.94191442352701 |
| 219 | MACO1 | 5 | 1 | 4.64673782865636 | 0.6312523044927 |
| 220 | LDLRAP1 | 4 | 1 | -4.9985639901913 | -0.514173521818411 |
| 221 | SELENON | 6 | 1 | 1.60663892309301 | -2.28012894016672 |
| 222 | AUNIP | 19 | 1 | -4.9967329355038 | 1.77152456420492 |
| 223 | PAQR7 | 5 | 1 | 3.57015444318883 | -0.880232141511333 |
| 224 | STMN1 | 10 | 1 | 3.73443509618871 | -1.69653455835749 |
| 225 | PAFAH2 | 11 | 1 | -3.00374029596217 | -1.8180605612414 |
| 226 | PDIK1L | 1 | 1 | 4.73008944074743 | 0.808737532077539 |
| 227 | ZNF593 | 13 | 1 | -2.24056898076899 | -2.06980834824015 |
| 228 | CEP85 | 16 | 1 | -3.88061045129664 | 2.40482582229208 |
| 229 | SH3BGRL3 | 6 | 1 | 1.06537975351445 | -2.77700970989634 |
| 230 | UBXN11 | 6 | 1 | 1.36306013147466 | -1.29159466844965 |
| 231 | LIN28A | 4 | 1 | -4.0951843114651 | 0.173522845683801 |
| 232 | DHDDS | 9 | 1 | 1.60618545095556 | 2.30973257201742 |
| 233 | HMGN2 | 4 | 1 | -3.89819143731959 | -0.403653278173697 |
| 234 | RPS6KA1 | 14 | 1 | -5.60703919847377 | 0.744121567188012 |
| 235 | ARID1A | 9 | 1 | 0.691413655682729 | 2.81078256743978 |
| 236 | ZDHHC18 | 10 | 1 | 3.98133636991613 | -1.01067048233916 |
| 237 | AL034380.1 | 1 | 1 | 1.88260103742711 | 1.19728197234701 |
| 238 | GPN2 | 2 | 1 | -1.06496922691233 | 0.895882026134241 |
| 239 | GPATCH3 | 8 | 1 | -1.45995079000361 | -0.0323764525072695 |
| 240 | NUDC | 8 | 1 | -1.44860421140559 | 0.294958607135522 |
| 241 | TENT5B | 19 | 1 | -5.27428410966761 | 0.861254230914819 |
| 242 | WDTC1 | 5 | 1 | 2.92022253553502 | 0.159079686580407 |
| 243 | TMEM222 | 13 | 1 | -0.536137178734614 | -1.14237487238814 |
| 244 | SYTL1 | 13 | 1 | -0.436108887389972 | -3.27296195846964 |
| 245 | WASF2 | 11 | 1 | -3.23537491281398 | -1.84461103302408 |
| 246 | AHDC1 | 5 | 1 | 4.13728977720372 | 0.0239963092191099 |
| 247 | IFI6 | 7 | 1 | -3.74610303361781 | -2.62420771938731 |
| 248 | FAM76A | 5 | 1 | 3.07321597616308 | -0.976400061668646 |
| 249 | STX12 | 5 | 1 | 4.1362335828983 | -0.869923179196012 |
| 250 | PPP1R8 | 2 | 1 | 0.279890194340871 | 1.59959019797872 |
| 251 | RPA2 | 19 | 1 | -4.45611594636805 | 1.68020548003744 |
| 252 | XKR8 | 7 | 1 | -2.31656597574122 | -2.38669668060709 |
| 253 | EYA3 | 7 | 1 | -2.1041855665005 | -2.45146773678232 |
| 254 | DNAJC8 | 5 | 1 | 2.46530009786718 | -0.817679441692126 |
| 255 | ATP5IF1 | 6 | 1 | 1.64640499631994 | -1.65067802292277 |
| 256 | AL353622.1 | 11 | 1 | -3.88997171838649 | -1.38206939321924 |
| 257 | SESN2 | 10 | 1 | 4.44253326933019 | -2.43229828697611 |
| 258 | MED18 | 17 | 1 | -0.135862030283286 | -0.0180492244379641 |
| 259 | PHACTR4 | 9 | 1 | 0.743459477826283 | 3.07605184691976 |
| 260 | RCC1 | 16 | 1 | -4.66217254121669 | 2.29477848189901 |
| 261 | TRNAU1AP | 10 | 1 | 3.09206439535253 | -1.83459101540018 |
| 262 | SNHG12 | 10 | 1 | 4.59341217558019 | -1.78105698448588 |
| 263 | TAF12 | 6 | 1 | 0.480834379597829 | -1.63539759737421 |
| 264 | RAB42 | 11 | 1 | -4.02396987398036 | -1.34855966192652 |
| 265 | AL360012.1 | 4 | 1 | -3.42880629022486 | 0.0288986124379514 |
| 266 | GMEB1 | 2 | 1 | 0.82197286169164 | 2.19699038642477 |
| 267 | YTHDF2 | 8 | 1 | -1.01829062421687 | 0.190088287769067 |

| 268 | EPB41 | 1 | 1 | 2.57876206915014 | 1.54259647506307 |
| --- | --- | --- | --- | --- | --- |
| 269 | SRSF4 | 9 | 1 | -0.259662917078211 | 2.5924099840505 |
| 270 | MECR | 3 | 1 | -3.06317923982508 | -0.415891125025046 |
| 271 | PTPRU | 7 | 1 | -2.33320282419093 | -2.77612362724711 |
| 272 | SDC3 | 15 | 1 | 4.75851942579381 | 0.114069239078271 |
| 273 | PUM1 | 9 | 1 | 0.546362951203511 | 2.80523575919698 |
| 274 | NKAIN1 | 6 | 1 | 2.57499839346044 | -2.14605174881388 |
| 275 | SNRNP40 | 13 | 1 | -0.421860426858737 | -1.19670812708308 |
| 276 | ZCCHC17 | 5 | 1 | 3.04608775655858 | -0.40417866331507 |
| 277 | FABP3 | 6 | 1 | 1.94725610296361 | -2.62725983482767 |
| 278 | SERINC2 | 7 | 1 | -2.48593662698634 | -2.87507127148081 |
| 279 | PEF1 | 3 | 1 | -1.80576561410792 | -0.738534449757826 |
| 280 | COL16A1 | 7 | 1 | -2.52793310602076 | -3.68818984848429 |
| 281 | ADGRB2 | 5 | 1 | 3.34285141508214 | -0.360374315799964 |
| 282 | PTP4A2 | 5 | 1 | 3.45922447721593 | -0.674162938417685 |
| 283 | KHDRBS1 | 3 | 1 | -2.78647778947718 | -0.0848967276232363 |
| 284 | TMEM39B | 8 | 1 | -0.933167681292369 | -0.103550358833563 |
| 285 | KPNA6 | 9 | 1 | 0.524593069955037 | 2.78288592475485 |
| 286 | TXLNA | 9 | 1 | 0.978520288869069 | 3.02261699813436 |
| 287 | CCDC28B | 10 | 1 | 4.00950886289708 | -1.41335122209955 |
| 288 | EIF3I | 3 | 1 | -1.90435980279811 | -1.09005150121619 |
| 289 | HDAC1 | 4 | 1 | -4.51703141649134 | -1.24773599130083 |
| 290 | MARCKSL1 | 10 | 1 | 3.37730433027379 | -1.48454092365671 |
| 291 | BSDC1 | 1 | 1 | 3.65404845754735 | 2.10019816535543 |
| 292 | ZBTB8B | 1 | 1 | 4.78728844205968 | 0.793261305270898 |
| 293 | ZBTB8A | 5 | 1 | 3.72062136213414 | -0.00794951540399802 |
| 294 | ZBTB8OS | 13 | 1 | -0.958398089007213 | -1.64247409922053 |
| 295 | RBBP4 | 9 | 1 | 0.899409904881642 | 2.93293871062826 |
| 296 | SYNC | 1 | 1 | 3.96614529173009 | 1.28431214469503 |
| 297 | KIAA1522 | 5 | 1 | 4.08809925596349 | 0.261727110324609 |
| 298 | YARS | 10 | 1 | 4.27553392927282 | -2.19965682846476 |
| 299 | S100PBP | 9 | 1 | 0.0790814308368425 | 3.09202183860372 |
| 300 | FNDC5 | 6 | 1 | 2.14909626524083 | -2.83538506847788 |
| 301 | HPCA | 8 | 1 | -1.46594344098933 | -0.347323998035681 |
| 302 | TMEM54 | 14 | 1 | -6.3941619249142 | -1.02947288674284 |
| 303 | RNF19B | 1 | 1 | 4.1757581381046 | 1.23292793410848 |
| 304 | AK2 | 3 | 1 | -2.70125864465602 | -0.571182980360282 |
| 305 | TRIM62 | 7 | 1 | -3.32374595125086 | -3.66041766029764 |
| 306 | ZNF362 | 2 | 1 | 0.594809010430501 | 1.70309628623556 |
| 307 | AL513327.1 | 8 | 1 | -1.48815594632991 | 1.65427960532736 |
| 308 | PHC2 | 3 | 1 | -2.6846766324795 | -1.18167252761294 |
| 309 | AL513327.2 | 4 | 1 | -4.19817541559108 | -1.37844438773562 |
| 310 | CSMD2 | 15 | 1 | 5.68978334943883 | 0.815572635112512 |
| 311 | SMIM12 | 13 | 1 | -0.830326423243358 | -1.7744192920344 |
| 312 | DLGAP3 | 10 | 1 | 3.52872945348851 | -1.49913231951166 |
| 313 | ZMYM6 | 2 | 1 | 0.0170532016002397 | 1.69482745307516 |
| 314 | ZMYM1 | 16 | 1 | -4.01566241701014 | 2.95574010985922 |
| 315 | SFPQ | 3 | 1 | -2.26200865228541 | 0.414869800983178 |
| 316 | ZMYM4 | 2 | 1 | -0.639344111279323 | 2.29461850303243 |
| 317 | KIAA0319L | 2 | 1 | -0.618417039707973 | 1.46086467879842 |
| 318 | NCDN | 1 | 1 | 1.40661896745794 | 1.72955049651693 |
| 319 | AC004865.2 | 15 | 1 | 5.82430006544225 | 0.842506543575036 |
| 320 | TFAP2E | 5 | 1 | 3.55764128248327 | -0.618664129795325 |
| 321 | PSMB2 | 6 | 1 | -0.275743290976359 | -1.44663970095087 |
| 322 | C1orf216 | 15 | 1 | 5.728460565015 | 0.62604047435354 |
| 323 | CLSPN | 19 | 1 | -4.77720187623866 | 1.75009812491964 |
| 324 | AGO4 | 1 | 1 | 3.75053884069555 | 2.51885332244467 |
| 325 | AGO1 | 7 | 1 | -2.96671007593043 | -2.76324437481333 |
| 326 | AGO3 | 12 | 1 | 2.17873217146032 | 2.68624486106466 |
| 327 | TEKT2 | 6 | 1 | 1.2769624141895 | -1.00002477240731 |
| 328 | ADPRHL2 | 8 | 1 | -1.27732525785334 | -0.038589163841498 |
| 329 | TRAPPC3 | 5 | 1 | 3.43252731840246 | -0.985147922457945 |
| 330 | MAP7D1 | 12 | 1 | 1.86151672880285 | 2.52144421714376 |
| 331 | THRAP3 | 3 | 1 | -2.40183924157984 | 1.00975395816396 |
| 332 | EVA1B | 14 | 1 | -6.30255626161463 | -0.465123160900366 |
| 333 | STK40 | 2 | 1 | -0.14035733063586 | 2.27180780547689 |
| 334 | LSM10 | 10 | 1 | 3.29619719068639 | -1.81770251614023 |

| 335 | OSCP1 | 13 | 1 | -1.10096989591487 | -2.7642589650767 |
| --- | --- | --- | --- | --- | --- |
| 336 | MRPS15 | 3 | 1 | -2.48322604616053 | -1.41373763901163 |
| 337 | GRIK3 | 18 | 1 | 6.27985598127477 | -2.44340120178629 |
| 338 | LINC01137 | 18 | 1 | 6.26566340009801 | -2.5961669526713 |
| 339 | MEAF6 | 10 | 1 | 3.60761882345311 | -1.72183035236765 |
| 340 | SNIP1 | 9 | 1 | 1.78070737402074 | 2.95994962829183 |
| 341 | DNALI1 | 11 | 1 | -3.64966486413844 | -2.38899062496592 |
| 342 | GNL2 | 3 | 1 | -2.23875163514979 | -0.118623717846167 |
| 343 | RSPO1 | 11 | 1 | -4.01280520875819 | -2.23676966053416 |
| 344 | C1orf109 | 3 | 1 | -3.29633425195582 | 0.212657824931848 |
| 345 | CDCA8 | 16 | 1 | -4.44796393831141 | 3.25895775931905 |
| 346 | EPHA10 | 10 | 1 | 4.0731256155216 | -1.15200535875727 |
| 347 | MANEAL | 10 | 1 | 4.50938345472448 | -1.12398521524836 |
| 348 | AL929472.3 | 1 | 1 | 2.75106717626684 | 1.33620418685507 |
| 349 | YRDC | 3 | 1 | -2.21322380025752 | -0.16370539289881 |
| 350 | C1orf122 | 6 | 1 | 2.01420856992833 | -1.50747118812968 |
| 351 | MTF1 | 2 | 1 | 0.784315839215444 | 2.08018864768575 |
| 352 | INPP5B | 9 | 1 | 1.18537355939977 | 3.18386067527364 |
| 353 | SF3A3 | 3 | 1 | -2.04565988500483 | 0.0828476108891844 |
| 354 | FHL3 | 7 | 1 | -2.95525835473902 | -2.06723736149241 |
| 355 | UTP11 | 13 | 1 | -1.30181358774073 | -0.648247166694892 |
| 356 | POU3F1 | 7 | 1 | -2.35909841974147 | -3.53878961426188 |
| 357 | RRAGC | 10 | 1 | 4.36846233884923 | -0.997445761384261 |
| 358 | MYCBP | 11 | 1 | -3.51826022584803 | -1.02505607825686 |
| 359 | AKIRIN1 | 2 | 1 | 0.943344250127004 | 1.17906703132223 |
| 360 | NDUFS5 | 6 | 1 | -0.0264540912664671 | -2.35542128902842 |
| 361 | MACF1 | 9 | 1 | 0.649559512540029 | 2.79245056289266 |
| 362 | PABPC4 | 3 | 1 | -1.86045525987513 | -0.0385183535235049 |
| 363 | HPCAL4 | 18 | 1 | 5.31828476469152 | -0.350040777744544 |
| 364 | PPIE | 4 | 1 | -3.78889702279933 | 0.418532029567468 |
| 365 | TRIT1 | 3 | 1 | -1.9766962381161 | 0.157497183261621 |
| 366 | MYCL | 7 | 1 | -3.6579124780453 | -3.03556500297953 |
| 367 | MFSD2A | 19 | 1 | -4.57002042253382 | 1.13791383880209 |
| 368 | CAP1 | 2 | 1 | 0.411895230218099 | 0.899203554568994 |
| 369 | PPT1 | 3 | 1 | -2.73283289392359 | 0.372601047931421 |
| 370 | RLF | 9 | 1 | 1.45848609487645 | 2.80486930984091 |
| 371 | AL050341.2 | 5 | 1 | 2.83090545217626 | -0.989904969157469 |
| 372 | ZMPSTE24 | 3 | 1 | -2.14953170736201 | -0.0140057645456911 |
| 373 | COL9A2 | 8 | 1 | -1.35046421010859 | -0.20350305658747 |
| 374 | SMAP2 | 15 | 1 | 4.64001680891149 | -0.0841172776835085 |
| 375 | ZFP69B | 1 | 1 | 4.02392794172399 | 2.18673814910482 |
| 376 | RIMS3 | 15 | 1 | 4.48886038343541 | -0.0328300438540102 |
| 377 | NFYC-AS1 | 8 | 1 | -2.17183505018122 | 1.3075503267629 |
| 378 | NFYC | 3 | 1 | -1.9339784236706 | -0.0550888023989321 |
| 379 | CITED4 | 3 | 1 | -2.44341300447352 | -0.696964322747481 |
| 380 | CTPS1 | 19 | 1 | -4.48330424745448 | 1.65044058936666 |
| 381 | SCMH1 | 1 | 1 | 4.011081948682 | 1.18027962821554 |
| 382 | FOXO6 | 18 | 1 | 5.3247158674442 | -0.681056319894087 |
| 383 | HIVEP3 | 15 | 1 | 4.38751961271398 | -0.0492846809046389 |
| 384 | FOXJ3 | 12 | 1 | 2.52967907468908 | 2.37764181274008 |
| 385 | RIMKLA | 18 | 1 | 5.08003737012975 | -0.764867126168501 |
| 386 | PPCS | 5 | 1 | 2.81192280332677 | -1.25217084509302 |
| 387 | CCDC30 | 1 | 1 | 2.99227358381383 | 1.40243734496664 |
| 388 | PPIH | 19 | 1 | -3.97582505662806 | 0.673069969592798 |
| 389 | YBX1 | 3 | 1 | -1.93573533018 | -0.463076784433615 |
| 390 | P3H1 | 2 | 1 | -0.165100634292438 | 2.11574186461996 |
| 391 | C1orf50 | 10 | 1 | 4.17277456800573 | -1.41347072941233 |
| 392 | SVBP | 10 | 1 | 2.95423842947118 | -2.02065192085673 |
| 393 | AL512353.1 | 4 | 1 | -4.19434927423365 | -1.33261839968134 |
| 394 | ZNF691 | 3 | 1 | -2.09873698671229 | -1.09820911210943 |
| 395 | SLC2A1 | 4 | 1 | -5.08889220674403 | -0.586509450496924 |
| 396 | FAM183A | 13 | 1 | -0.585025981262996 | -2.88101003986765 |
| 397 | EBNA1BP2 | 3 | 1 | -1.75042055566676 | -1.22874466997553 |
| 398 | CFAP57 | 7 | 1 | -2.29033397157557 | -3.72513185364176 |
| 399 | AL139289.2 | 4 | 1 | -4.24594162423976 | -0.00587974411417258 |
| 400 | CDC20 | 16 | 1 | -4.29923604448207 | 2.97323335784506 |
| 401 | ELOVL1 | 7 | 1 | -1.68415485341914 | -3.50999604088236 |

| 402 | MED8 | 6 | 1 | 0.00723780672185226 | -1.49933485609461 |
| --- | --- | --- | --- | --- | --- |
| 403 | SZT2 | 9 | 1 | 1.21693314592473 | 2.96295394080709 |
| 404 | PTPRF | 1 | 1 | 3.52826501409643 | 1.46288694518636 |
| 405 | KDM4A | 2 | 1 | -0.247667566601857 | 1.74246348517965 |
| 406 | KDM4A-AS1 | 8 | 1 | -1.81198321302302 | 1.87824262755941 |
| 407 | ST3GAL3 | 16 | 1 | -3.30340240915187 | 2.52557911056112 |
| 408 | DPH2 | 3 | 1 | -2.98425911386378 | -0.730119093479407 |
| 409 | ATP6V0B | 10 | 1 | 3.15418864767186 | -1.90298949104716 |
| 410 | B4GALT2 | 17 | 1 | 1.70240928213231 | -0.635909750284445 |
| 411 | CCDC24 | 6 | 1 | 2.31861068288915 | -1.36546410542895 |
| 412 | SLC6A9 | 11 | 1 | -3.90034435708888 | -1.90086267811228 |
| 413 | AL139220.2 | 11 | 1 | -3.81330059488185 | -2.2822249971049 |
| 414 | DMAP1 | 17 | 1 | 0.0246566681110124 | -0.185186012806189 |
| 415 | ERI3 | 13 | 1 | -1.68202100713618 | -0.704210101784957 |
| 416 | RNF220 | 1 | 1 | 3.22885705511205 | 1.42459787505697 |
| 417 | TMEM53 | 10 | 1 | 3.23015047590368 | -1.71563045603205 |
| 418 | KIF2C | 16 | 1 | -4.40984652955897 | 3.30066098350118 |
| 419 | RPS8 | 21 | 1 | -0.925187751845195 | -4.45048127991129 |
| 420 | PLK3 | 7 | 1 | -3.19735763986476 | -3.64971648079325 |
| 421 | BTBD19 | 7 | 1 | -4.09244464357264 | -2.80085121017863 |
| 422 | EIF2B3 | 8 | 1 | -1.55404053647883 | 0.654103056369531 |
| 423 | HECTD3 | 7 | 1 | -2.54495213945277 | -3.69151006561686 |
| 424 | UROD | 13 | 1 | -1.64951203782923 | -2.07879446369578 |
| 425 | ZSWIM5 | 1 | 1 | 3.60756780187719 | 1.17873944419454 |
| 426 | HPDL | 7 | 1 | -2.82803295572169 | -3.33204232078959 |
| 427 | MUTYH | 4 | 1 | -3.45577786882289 | -0.396349354805243 |
| 428 | TOE1 | 3 | 1 | -3.37338422258265 | 0.353270665584314 |
| 429 | TESK2 | 11 | 1 | -4.00004075487025 | -1.82584492784907 |
| 430 | MMACHC | 4 | 1 | -3.5919625612057 | 0.360196963725793 |
| 431 | PRDX1 | 7 | 1 | -2.32137201745875 | -2.79055510384012 |
| 432 | AKR1A1 | 11 | 1 | -3.288140997485 | -1.19500325542856 |
| 433 | NASP | 8 | 1 | -1.86561296899684 | 0.701559797702539 |
| 434 | GPBP1L1 | 3 | 1 | -2.59912847001918 | -0.0573584876673342 |
| 435 | TMEM69 | 8 | 1 | -1.62644408662684 | -0.00689862591196311 |
| 436 | IPP | 8 | 1 | -2.21717487295039 | 1.1853317179067 |
| 437 | MAST2 | 1 | 1 | 3.87009502927892 | 2.04417933600973 |
| 438 | PIK3R3 | 9 | 1 | 0.782771959706471 | 2.91324867385458 |
| 439 | POMGNT1 | 17 | 1 | 0.466367974682973 | 0.00576616900991189 |
| 440 | RAD54L | 19 | 1 | -5.62521003206141 | 1.45302118438314 |
| 441 | LRRC41 | 8 | 1 | -1.0407369585789 | -0.402612014831793 |
| 442 | UQCRH | 13 | 1 | -0.160551071361377 | -2.47813938480784 |
| 443 | FAAH | 18 | 1 | 5.10684992353551 | -1.0235715575235 |
| 444 | MKNK1 | 6 | 1 | 1.44606842081182 | -2.58769868236948 |
| 445 | ATPAF1 | 9 | 1 | -0.262568226278498 | 2.45373596328329 |
| 446 | EFCAB14 | 2 | 1 | -0.181477844909503 | 2.41380228179525 |
| 447 | CYP4X1 | 18 | 1 | 6.10768534223668 | -2.06843386513163 |
| 448 | STIL | 16 | 1 | -4.8141157480038 | 2.33656038421224 |
| 449 | CMPK1 | 1 | 1 | 3.99951960126989 | 0.839794532237756 |
| 450 | SPATA6 | 7 | 1 | -3.021459564761 | -2.46907029968668 |
| 451 | BEND5 | 17 | 1 | 0.385038450165914 | -0.247799738468421 |
| 452 | ELAVL4 | 5 | 1 | 3.06065012495153 | -0.790184556664717 |
| 453 | AL592182.3 | 18 | 1 | 6.03870226423375 | -2.17557631355692 |
| 454 | DMRTA2 | 13 | 1 | -1.42514739950068 | -2.67564879280497 |
| 455 | FAF1 | 5 | 1 | 2.45138765852086 | -0.338598891319525 |
| 456 | CDKN2C | 16 | 1 | -4.4801518770016 | 3.32642783301901 |
| 457 | RNF11 | 5 | 1 | 3.91631342451207 | -0.180628522457373 |
| 458 | EPS15 | 12 | 1 | 2.29690386335485 | 2.24171127456259 |
| 459 | OSBPL9 | 2 | 1 | 0.490264311238454 | 1.08134128230642 |
| 460 | NRDC | 9 | 1 | 0.710240021153615 | 3.09362925666403 |
| 461 | RAB3B | 5 | 1 | 3.95608116666906 | -0.302917703212988 |
| 462 | TXNDC12 | 4 | 1 | -3.65410874803431 | 0.0768883981091856 |
| 463 | BTF3L4 | 17 | 1 | 0.356002107068227 | 0.00804700034688699 |
| 464 | ZFYVE9 | 15 | 1 | 5.42357470075719 | 0.680208817897546 |
| 465 | CC2D1B | 1 | 1 | 2.84825517217748 | 1.33842338698934 |
| 466 | ORC1 | 19 | 1 | -4.7411439271725 | 1.32140649932455 |
| 467 | PRPF38A | 8 | 1 | -2.09542869050868 | 1.20189036506246 |
| 468 | TUT4 | 1 | 1 | 4.1258895544254 | 1.44799412864279 |

| 469 | AL591167.1 | 4 | 1 | -4.33699630220301 | 0.0219991363866209 |
| --- | --- | --- | --- | --- | --- |
| 470 | GPX7 | 11 | 1 | -3.34861920793421 | -1.70667074543406 |
| 471 | SHISAL2A | 10 | 1 | 3.49537040273778 | -1.70925430876185 |
| 472 | COA7 | 15 | 1 | 4.98826529066198 | 0.124872580943811 |
| 473 | ZYG11B | 1 | 1 | 3.3257210401737 | 1.68802942412924 |
| 474 | SCP2 | 3 | 1 | -2.66571043451197 | -0.969459428610098 |
| 475 | PODN | 14 | 1 | -6.91057990510829 | -0.771824940265906 |
| 476 | CPT2 | 3 | 1 | -2.28838203866847 | -0.838233008207572 |
| 477 | AL606760.2 | 9 | 1 | 1.01146163026922 | 2.54464162963461 |
| 478 | C1orf123 | 13 | 1 | -0.826688394144847 | -2.02869032246043 |
| 479 | MAGOH | 3 | 1 | -3.22833488901026 | -0.870712892310512 |
| 480 | LRP8 | 1 | 1 | 3.17259217779271 | 0.804187551913965 |
| 481 | GLIS1 | 7 | 1 | -2.66539667566188 | -3.69900046211649 |
| 482 | NDC1 | 16 | 1 | -4.45950387437709 | 2.32089723723959 |
| 483 | YIPF1 | 6 | 1 | 0.422965839310811 | -1.26116673571039 |
| 484 | HSPB11 | 19 | 1 | -4.5317351671017 | 1.54228796142172 |
| 485 | LRRC42 | 7 | 1 | -2.63649915178187 | -2.7097220264094 |
| 486 | TMEM59 | 6 | 1 | 1.91119696180456 | -1.73164342266489 |
| 487 | TCEANC2 | 8 | 1 | -1.86805258710749 | 1.57246889251302 |
| 488 | CYB5RL | 19 | 1 | -5.18422292192347 | 1.34788955825399 |
| 489 | MRPL37 | 13 | 1 | -2.18241260965235 | -1.54003940445353 |
| 490 | SSBP3 | 6 | 1 | 1.69824434797399 | -1.57669024092127 |
| 491 | SSBP3-AS1 | 1 | 1 | 1.66769637148015 | 1.24263323920797 |
| 492 | TTC4 | 3 | 1 | -2.37926290948756 | 0.97165598052572 |
| 493 | PARS2 | 3 | 1 | -1.99678955991633 | -0.441789283575308 |
| 494 | DHCR24 | 5 | 1 | 3.19175864736669 | -0.208187683643592 |
| 495 | USP24 | 9 | 1 | 1.83898235838048 | 3.03872288840841 |
| 496 | PLPP3 | 4 | 1 | -4.71097491701014 | -0.584643914522421 |
| 497 | PRKAA2 | 1 | 1 | 3.70499207059972 | 2.09253753799032 |
| 498 | DAB1 | 18 | 1 | 5.59649111311071 | -0.665307461442244 |
| 499 | OMA1 | 4 | 1 | -4.68871997316249 | -0.314773841919196 |
| 500 | MYSM1 | 12 | 1 | 3.18550850431554 | 2.9013003267629 |
| 501 | JUN | 10 | 1 | 4.33006121198766 | -1.67286007028986 |
| 502 | AC093424.1 | 5 | 1 | 3.39992882291906 | -0.70457099300791 |
| 503 | FGGY | 14 | 1 | -5.88114951570399 | -0.468015386881125 |
| 504 | HOOK1 | 15 | 1 | 5.18461634199254 | 0.510070339618432 |
| 505 | LINC01748 | 7 | 1 | -2.34547399003871 | -3.7009876809733 |
| 506 | AC099792.1 | 7 | 1 | -2.02639423330195 | -3.61640821319987 |
| 507 | NFIA | 13 | 1 | -0.825373396471812 | -3.27501164299418 |
| 508 | TM2D1 | 5 | 1 | 3.46735717336767 | -0.961841496797097 |
| 509 | AC099791.2 | 10 | 1 | 4.23889376203649 | -1.30551756841112 |
| 510 | PATJ | 18 | 1 | 5.47725559751622 | -0.953737761052144 |
| 511 | L1TD1 | 11 | 1 | -3.97046421487696 | -2.25852535587717 |
| 512 | KANK4 | 14 | 1 | -5.95498345811732 | 0.0739138283116697 |
| 513 | USP1 | 16 | 1 | -4.44690869768031 | 1.99683274406027 |
| 514 | DOCK7 | 12 | 1 | 2.30282594244115 | 2.79228629249166 |
| 515 | ATG4C | 16 | 1 | -3.50154064615138 | 2.38727082389425 |
| 516 | FOXD3-AS1 | 20 | 1 | 3.27586032430761 | -3.8464966855662 |
| 517 | FOXD3 | 20 | 1 | 3.36608149091832 | -3.80449019295145 |
| 518 | ALG6 | 17 | 1 | 1.82083870451085 | -0.125562473358405 |
| 519 | ITGB3BP | 19 | 1 | -4.50055621583827 | 1.55630625861715 |
| 520 | EFCAB7 | 1 | 1 | 3.33917881528966 | 1.07418538707327 |
| 521 | PGM1 | 4 | 1 | -4.78241799791224 | -0.760731249274504 |
| 522 | ROR1 | 11 | 1 | -3.87596009691127 | -2.18999968391825 |
| 523 | CACHD1 | 7 | 1 | -3.39838956315882 | -2.38026760441233 |
| 524 | RAVER2 | 4 | 1 | -3.54942058046229 | -0.409779056133521 |
| 525 | JAK1 | 1 | 1 | 3.30376912634008 | 0.897189155994165 |
| 526 | AK4 | 14 | 1 | -5.6547329278744 | 0.0425363935811399 |
| 527 | DNAJC6 | 1 | 1 | 3.79823090116613 | 0.721952573238122 |
| 528 | LEPROT | 7 | 1 | -1.91075967271693 | -2.84803830009867 |
| 529 | LEPR | 2 | 1 | -0.330224782422854 | 1.53791131156515 |
| 530 | PDE4B | 18 | 1 | 5.37756134550206 | -1.94809304100443 |
| 531 | SGIP1 | 5 | 1 | 4.30162598173253 | 0.18513037341665 |
| 532 | AL139147.1 | 1 | 1 | 3.34327508489721 | 1.50525392669271 |
| 533 | WDR78 | 2 | 1 | -0.0274826886213561 | 0.677234546123254 |
| 534 | MIER1 | 1 | 1 | 3.74199415723913 | 1.96406830924581 |
| 535 | SLC35D1 | 3 | 1 | -2.41963265855677 | 0.936170355258691 |

| 536 | SERBP1 | 3 | 1 | -3.27126501520045 | -0.765458076419127 |
| --- | --- | --- | --- | --- | --- |
| 537 | GADD45A | 7 | 1 | -2.7278849931515 | -3.97000323158671 |
| 538 | GNG12 | 14 | 1 | -6.44499944169886 | -0.157701595844519 |
| 539 | DIRAS3 | 15 | 1 | 5.68479515592687 | 0.33031179088186 |
| 540 | WLS | 14 | 1 | -6.69352315385707 | -0.437218143762839 |
| 541 | DEPDC1 | 16 | 1 | -4.38701127488978 | 3.44870080131124 |
| 542 | DEPDC1-AS1 | 16 | 1 | -4.42121289689906 | 3.05110968726705 |
| 543 | AL035412.1 | 16 | 1 | -4.28819535692103 | 3.31492270606588 |
| 544 | LRRC7 | 18 | 1 | 5.31881739179723 | -0.755223079742682 |
| 545 | LRRC40 | 1 | 1 | 3.18043591062658 | 2.01881374496053 |
| 546 | SRSF11 | 8 | 1 | -0.463783577278926 | -0.0221766076700808 |
| 547 | ANKRD13C | 15 | 1 | 5.20570446531408 | 1.3343852915151 |
| 548 | HHLA3 | 19 | 1 | -3.97663877923854 | 0.534574524341333 |
| 549 | CTH | 15 | 1 | 6.06427361051671 | 0.752710596500146 |
| 550 | AL354872.2 | 15 | 1 | 6.09569622556798 | 0.786286369739282 |
| 551 | PTGER3 | 15 | 1 | 6.03495718519323 | 0.788461343227136 |
| 552 | ZRANB2 | 8 | 1 | -2.033631190852 | 1.3769091524465 |
| 553 | NEGR1 | 1 | 1 | 4.66039015333287 | 1.22131003516744 |
| 554 | FPGT | 3 | 1 | -2.57326029260524 | -0.564276947798026 |
| 555 | CRYZ | 4 | 1 | -4.19541238267787 | -0.683379410924208 |
| 556 | TYW3 | 11 | 1 | -3.69327734430201 | -0.833634301246894 |
| 557 | LHX8 | 4 | 1 | -5.36302350480921 | -1.82384185415674 |
| 558 | SLC44A5 | 9 | 1 | 1.42949821035497 | 2.43073333877157 |
| 559 | ACADM | 11 | 1 | -3.03560422380336 | -1.70109628063608 |
| 560 | RABGGTB | 17 | 1 | 1.26393128912084 | -0.33840535503794 |
| 561 | ST6GALNAC3 | 1 | 1 | 2.73536707441442 | 1.45565284865926 |
| 562 | ST6GALNAC5 | 3 | 1 | -2.26045058687098 | 0.127624408183801 |
| 563 | PIGK | 2 | 1 | 0.883412852688954 | 0.913952127872217 |
| 564 | AK5 | 10 | 1 | 4.62143637220495 | -0.573604657472861 |
| 565 | AC118549.1 | 2 | 1 | 0.528099909230397 | 2.18277563232015 |
| 566 | USP33 | 1 | 1 | 4.2726147321903 | 1.09071542399954 |
| 567 | MIGA1 | 12 | 1 | 2.60869123022191 | 2.50847949164938 |
| 568 | NEXN-AS1 | 14 | 1 | -5.77743552644618 | 0.0943620361668943 |
| 569 | NEXN | 14 | 1 | -5.52977107484706 | -0.249821349205268 |
| 570 | FUBP1 | 2 | 1 | -0.833911583021953 | 2.54636801856588 |
| 571 | DNAJB4 | 3 | 1 | -3.33810924013026 | 0.235507027087915 |
| 572 | AC103591.3 | 4 | 1 | -4.11376474817164 | -0.169283374370825 |
| 573 | IFI44L | 11 | 1 | -3.25438855607875 | -2.41263650280405 |
| 574 | LINC01781 | 4 | 1 | -4.54051612337001 | -0.797209656955493 |
| 575 | ADGRL2 | 1 | 1 | 2.66148449461095 | 1.57211197990011 |
| 576 | TTLL7 | 1 | 1 | 3.70353485624425 | 1.39431323188375 |
| 577 | AL359504.2 | 3 | 1 | -3.36521028001673 | 0.310296431956994 |
| 578 | PRKACB | 18 | 1 | 5.55419518034093 | -0.541908367695105 |
| 579 | SAMD13 | 14 | 1 | -4.96325324495204 | 0.0614388145787595 |
| 580 | RPF1 | 3 | 1 | -2.88756559808619 | -0.933426539571774 |
| 581 | GNG5 | 7 | 1 | -2.84088252504237 | -2.11746571880747 |
| 582 | SPATA1 | 11 | 1 | -3.57253883798487 | -1.58452425819803 |
| 583 | CTBS | 4 | 1 | -5.08695958574183 | -0.25411306244303 |
| 584 | SSX2IP | 3 | 1 | -2.66004608590968 | 1.02213527339529 |
| 585 | WDR63 | 11 | 1 | -3.86403177698024 | -2.01962409836222 |
| 586 | SYDE2 | 14 | 1 | -5.36510203798182 | 0.123906389652002 |
| 587 | C1orf52 | 10 | 1 | 3.7597391752445 | -1.40429548841883 |
| 588 | BCL10 | 1 | 1 | 3.02419735471837 | 1.65009774344991 |
| 589 | DDAH1 | 14 | 1 | -5.01891683061488 | 0.462895409045923 |
| 590 | AC092807.3 | 14 | 1 | -6.11040900667079 | -0.166706665577185 |
| 591 | CYR61 | 14 | 1 | -5.66214297731288 | 0.179460541187036 |
| 592 | ZNHIT6 | 2 | 1 | -0.516949132279231 | 2.37172474044393 |
| 593 | COL24A1 | 7 | 1 | -3.26384614427455 | -2.89954768043925 |
| 594 | ODF2L | 5 | 1 | 3.17171527425878 | 0.253320590434778 |
| 595 | SH3GLB1 | 1 | 1 | 2.93870688955419 | 1.20160164016317 |
| 596 | SELENOF | 3 | 1 | -2.55381177385218 | -0.691486328067076 |
| 597 | HS2ST1 | 8 | 1 | -2.09772704561122 | 1.40575923102926 |
| 598 | LMO4 | 11 | 1 | -3.87569926698573 | -1.61165438515116 |
| 599 | PKN2-AS1 | 11 | 1 | -3.7095801683224 | -2.3550985894816 |
| 600 | PKN2 | 3 | 1 | -2.80359719713099 | 0.470481411395776 |
| 601 | GTF2B | 6 | 1 | 0.572748377724812 | -1.82261739593912 |
| 602 | KYAT3 | 3 | 1 | -2.68516825158961 | -1.17800529223849 |

| 603 | RBMXL1 | 2 | 1 | 0.388799980088399 | 1.77481426375936 |
| --- | --- | --- | --- | --- | --- |
| 604 | GBP4 | 11 | 1 | -4.36196015794642 | -1.62179325443674 |
| 605 | AC099063.4 | 1 | 1 | 2.8614750055515 | 0.370141521869409 |
| 606 | LRRC8B | 9 | 1 | 1.32712711374395 | 2.51112808364462 |
| 607 | LRRC8C-DT | 4 | 1 | -4.87483333070643 | -0.811233966769469 |
| 608 | LRRC8C | 4 | 1 | -4.42559598405726 | -0.831484100939524 |
| 609 | LRRC8D | 2 | 1 | -0.893812701300456 | 1.39928855079244 |
| 610 | ZNF326 | 3 | 1 | -2.40663622339137 | 0.794437543330896 |
| 611 | BARHL2 | 6 | 1 | 2.32132388631933 | -2.43968747478892 |
| 612 | LINC01763 | 5 | 1 | 4.07373453657262 | -0.676712214173567 |
| 613 | ZNF644 | 12 | 1 | 2.86263467352025 | 2.60834516662191 |
| 614 | CDC7 | 16 | 1 | -3.81033371408351 | 2.33420433181356 |
| 615 | TGFBR3 | 7 | 1 | -3.73526452501185 | -3.36114917618204 |
| 616 | EPHX4 | 14 | 1 | -5.70597766359217 | -0.260024055065405 |
| 617 | BTBD8 | 5 | 1 | 4.26287628690831 | 0.174803630290735 |
| 618 | GLMN | 8 | 1 | -1.52326367814906 | 0.37586785930227 |
| 619 | RPAP2 | 9 | 1 | 0.686418428822682 | 2.74482597487997 |
| 620 | GFI1 | 13 | 1 | -0.139047905997111 | -3.04291759354044 |
| 621 | EVI5 | 8 | 1 | -1.23829583843119 | 1.01106835979055 |
| 622 | RPL5 | 21 | 1 | -0.637431785658671 | -4.16228495460917 |
| 623 | FAM69A | 5 | 1 | 3.48336483518712 | -0.139073535026801 |
| 624 | MTF2 | 9 | 1 | 1.2133387474262 | 2.40406383651327 |
| 625 | TMED5 | 3 | 1 | -3.69309161622889 | 0.734980479655969 |
| 626 | CCDC18 | 16 | 1 | -4.42917750795253 | 3.22077740806173 |
| 627 | DR1 | 9 | 1 | 0.193158641263173 | 2.902745858608 |
| 628 | FNBP1L | 5 | 1 | 4.29936005155675 | 0.424680963931787 |
| 629 | BCAR3 | 14 | 1 | -6.57845710237391 | -0.362348064007056 |
| 630 | DNTTIP2 | 8 | 1 | -0.838811919287517 | 0.523814932285058 |
| 631 | GCLM | 16 | 1 | -3.75575850923426 | 2.4170383371694 |
| 632 | ARHGAP29 | 10 | 1 | 4.56490828077428 | -1.10552598458697 |
| 633 | ABCD3 | 9 | 1 | -0.222674915761425 | 2.90178193229269 |
| 634 | F3 | 14 | 1 | -5.65354941804774 | 0.260107413707483 |
| 635 | SLC44A3 | 7 | 1 | -2.65036796052821 | -3.86528597694803 |
| 636 | CNN3 | 7 | 1 | -2.7215909810818 | -2.2045459590571 |
| 637 | AC105942.1 | 2 | 1 | 0.874790206357167 | 0.664066807208764 |
| 638 | TMEM56 | 2 | 1 | 1.17747987787359 | 1.05697609561514 |
| 639 | RWDD3 | 8 | 1 | -2.03237818200953 | 1.11837734359335 |
| 640 | PTBP2 | 2 | 1 | -0.0621295424259444 | 1.23736156600546 |
| 641 | DPYD | 7 | 1 | -3.01096747835047 | -2.74649558884074 |
| 642 | MIR137HG | 1 | 1 | 3.28212620298497 | 1.3291374601705 |
| 643 | BX005019.1 | 1 | 1 | 3.25541688482396 | 1.26683296340536 |
| 644 | SNX7 | 4 | 1 | -4.94614909608729 | -0.446717127384436 |
| 645 | PLPPR5 | 15 | 1 | 5.8988969473087 | 0.791820661006677 |
| 646 | PLPPR4 | 15 | 1 | 5.96815372984044 | 0.706462160526025 |
| 647 | PALMD | 6 | 1 | 2.01667644064061 | -2.33917389732767 |
| 648 | FRRS1 | 14 | 1 | -5.78708194215663 | -0.465169563116324 |
| 649 | AGL | 2 | 1 | -1.00751380641825 | 1.91461767333578 |
| 650 | SLC35A3 | 9 | 1 | 1.76742340604894 | 3.23658312934469 |
| 651 | AC118553.2 | 9 | 1 | 0.678611352845357 | 2.87027801650594 |
| 652 | SASS6 | 16 | 1 | -3.98452948053248 | 2.34598793166708 |
| 653 | TRMT13 | 2 | 1 | -0.607244119242503 | 1.9054461874349 |
| 654 | DBT | 9 | 1 | 1.27042748014562 | 2.88864864486288 |
| 655 | RTCA | 6 | 1 | 0.0588477400981645 | -0.791025697411788 |
| 656 | CDC14A | 4 | 1 | -3.31028936822779 | -0.238435550750983 |
| 657 | VCAM1 | 14 | 1 | -6.78863357026942 | -0.446193351568473 |
| 658 | EXTL2 | 17 | 1 | 1.02348531763189 | 0.287897125659692 |
| 659 | SLC30A7 | 2 | 1 | -0.317049421564414 | 2.3225547232015 |
| 660 | DPH5 | 3 | 1 | -2.63798426111109 | -0.811268738629115 |
| 661 | AC093157.1 | 4 | 1 | -3.66323159654505 | 0.0464431800229429 |
| 662 | AL109741.1 | 16 | 1 | -4.44228766878016 | 2.42596758979391 |
| 663 | S1PR1 | 4 | 1 | -4.67844747026332 | -0.558133377851737 |
| 664 | LINC01307 | 10 | 1 | 2.97511650602453 | -1.53031019789149 |
| 665 | OLFM3 | 15 | 1 | 5.64809490720861 | 0.207994953571069 |
| 666 | COL11A1 | 4 | 1 | -4.52519248445399 | -1.12489846271445 |
| 667 | RNPC3 | 1 | 1 | 4.26738716642492 | 1.98191703933309 |
| 668 | PRMT6 | 3 | 1 | -2.35765503366358 | 0.850573913036096 |
| 669 | NTNG1 | 20 | 1 | 3.6839814333164 | -3.44629131180216 |

| 670 | VAV3 | 10 | 1 | 4.26262260954015 | -2.68129227978159 |
| --- | --- | --- | --- | --- | --- |
| 671 | SLC25A24 | 11 | 1 | -3.21197865922816 | -1.99664591175486 |
| 672 | FAM102B | 12 | 1 | 2.14861513654821 | 1.88593210357259 |
| 673 | HENMT1 | 17 | 1 | 1.4154734758579 | -0.640824630083335 |
| 674 | PRPF38B | 2 | 1 | -0.487743824914767 | 1.0015748895986 |
| 675 | STXBP3 | 11 | 1 | -3.40449617822535 | -1.23277829510142 |
| 676 | AKNAD1 | 14 | 1 | -6.37811969240077 | -1.29500473958422 |
| 677 | GPSM2 | 16 | 1 | -4.28220437486537 | 3.23053588050436 |
| 678 | CLCC1 | 2 | 1 | -1.19500772674449 | 2.66127313750814 |
| 679 | WDR47 | 1 | 1 | 3.67965318243139 | 0.785892859874475 |
| 680 | TAF13 | 2 | 1 | 0.319746687813924 | 2.09064330237936 |
| 681 | AL356488.3 | 11 | 1 | -3.60760329683192 | -0.988709121169341 |
| 682 | TMEM167B | 1 | 1 | 1.5692820696079 | 1.45049728530477 |
| 683 | C1orf194 | 10 | 1 | 2.89440752546422 | -1.71791766506602 |
| 684 | KIAA1324 | 1 | 1 | 3.3361392168247 | 0.628549710689294 |
| 685 | SARS | 10 | 1 | 4.04814602415197 | -1.95609722954203 |
| 686 | CELSR2 | 7 | 1 | -2.66820620019801 | -3.80562554222513 |
| 687 | PSRC1 | 16 | 1 | -4.28843854387171 | 3.25046147483419 |
| 688 | SORT1 | 9 | 1 | -0.269370632440521 | 3.12206782477926 |
| 689 | PSMA5 | 3 | 1 | -2.26068829019435 | -1.19448427778651 |
| 690 | ATXN7L2 | 11 | 1 | -2.85230611284144 | -1.79820458513666 |
| 691 | AMIGO1 | 18 | 1 | 5.3484785703861 | -0.657830341877234 |
| 692 | GNAI3 | 8 | 1 | -0.659718230561091 | 0.609257356105554 |
| 693 | AMPD2 | 6 | 1 | 2.57646848241918 | -1.33740274530817 |
| 694 | GSTM4 | 4 | 1 | -5.16865609605677 | -0.54154495817591 |
| 695 | GSTM1 | 4 | 1 | -4.32504437883265 | 0.186257139621484 |
| 696 | AC000032.1 | 6 | 1 | 2.2074325231754 | -1.78422133547236 |
| 697 | GSTM3 | 6 | 1 | 1.84972740690343 | -1.67254565578867 |
| 698 | AHCYL1 | 3 | 1 | -2.04547785241969 | 0.236951724467981 |
| 699 | STRIP1 | 1 | 1 | 4.1666257528507 | 2.07828988212179 |
| 700 | AL160006.1 | 12 | 1 | 2.16620995084874 | 2.39969910758566 |
| 701 | RBM15-AS1 | 16 | 1 | -4.40212796647914 | 2.47696937697958 |
| 702 | RBM15 | 16 | 1 | -3.59372542817958 | 2.33528627532552 |
| 703 | SLC16A4 | 16 | 1 | -4.56641744096644 | 2.15885509627889 |
| 704 | AL355488.1 | 9 | 1 | -0.375211641386821 | 3.16793812888693 |
| 705 | LAMTOR5 | 13 | 1 | -1.65742503126033 | -1.58772836548258 |
| 706 | KCNA2 | 15 | 1 | 5.86259199659459 | 0.144563929019678 |
| 707 | AL365361.1 | 1 | 1 | 4.70835186521642 | 1.18392600196432 |
| 708 | AL360270.1 | 1 | 1 | 1.91965366880529 | 1.14499081748556 |
| 709 | LRIF1 | 5 | 1 | 3.6784014848911 | -0.258894487442267 |
| 710 | DRAM2 | 17 | 1 | 1.65784443895452 | -0.435319348396552 |
| 711 | CEPT1 | 1 | 1 | 1.46106125394933 | 1.69933714049886 |
| 712 | PIFO | 13 | 1 | -0.390923828080966 | -3.07641230446268 |
| 713 | WDR77 | 4 | 1 | -3.45062302072413 | -0.228738769115698 |
| 714 | ATP5PB | 13 | 1 | -1.67271338422663 | -1.97471533638407 |
| 715 | RAP1A | 5 | 1 | 3.42067123930089 | 0.124192253528345 |
| 716 | DDX20 | 8 | 1 | -1.11527942140467 | 0.564008490024316 |
| 717 | KCND3 | 18 | 1 | 5.68789173643224 | -1.58228950125147 |
| 718 | LINC01750 | 18 | 1 | 5.49525906126134 | -0.483746125520957 |
| 719 | CTTNBP2NL | 12 | 1 | 2.16699720899694 | 3.00011147636007 |
| 720 | WNT2B | 1 | 1 | 3.45762492696874 | 1.5068706430776 |
| 721 | ST7L | 2 | 1 | -0.267521020219399 | 0.924415842471826 |
| 722 | CAPZA1 | 9 | 1 | 0.850842013760732 | 2.43775214332174 |
| 723 | MOV10 | 4 | 1 | -4.68340419252284 | -0.659419193090689 |
| 724 | RHOC | 4 | 1 | -5.20165847261317 | -0.730543195428145 |
| 725 | SLC16A1 | 11 | 1 | -3.734430059985 | -0.978506400408042 |
| 726 | SLC16A1-AS1 | 1 | 1 | 2.10597421209447 | 0.946739450870263 |
| 727 | LRIG2 | 1 | 1 | 3.46059419195287 | 2.01282824653219 |
| 728 | MAGI3 | 1 | 1 | 2.8385007528507 | 0.88841988223623 |
| 729 | PHTF1 | 7 | 1 | -3.01827763040431 | -2.48457668644358 |
| 730 | RSBN1 | 1 | 1 | 3.94006277601354 | 0.809951917110193 |
| 731 | AP4B1-AS1 | 12 | 1 | 2.71279646436803 | 2.30018200057577 |
| 732 | AP4B1 | 10 | 1 | 4.77449299375646 | -0.754098220886481 |
| 733 | DCLRE1B | 12 | 1 | 3.28018380682103 | 2.67998160499166 |
| 734 | HIPK1 | 12 | 1 | 2.27989269773595 | 1.91234125274252 |
| 735 | OLFML3 | 4 | 1 | -4.40587352235682 | -0.451121553005469 |
| 736 | SYT6 | 5 | 1 | 3.11006785909765 | -0.438716962160361 |

| 737 | TRIM33 | 9 | 1 | 1.75314070264928 | 3.00531782287191 |
| --- | --- | --- | --- | --- | --- |
| 738 | BCAS2 | 17 | 1 | 0.392791464730428 | -0.207466109813941 |
| 739 | NRAS | 3 | 1 | -2.72279118020899 | 0.226938501773584 |
| 740 | CSDE1 | 2 | 1 | 0.358057215615437 | 1.76680602210592 |
| 741 | SIKE1 | 2 | 1 | -0.116490736798121 | 2.37227405685018 |
| 742 | TSPAN2 | 10 | 1 | 4.78345514814489 | -1.48706142527033 |
| 743 | VANGL1 | 2 | 1 | 0.81902183572881 | 0.635472670970666 |
| 744 | CASQ2 | 20 | 1 | 3.39288355390661 | -3.57635484558512 |
| 745 | NHLH2 | 20 | 1 | 3.40970636884801 | -3.64782701355387 |
| 746 | SLC22A15 | 5 | 1 | 4.08429457227819 | 0.193880096851098 |
| 747 | ATP1A1 | 6 | 1 | 2.32409740965001 | -1.33738849979807 |
| 748 | CD58 | 11 | 1 | -3.88797424753077 | -0.896517140948963 |
| 749 | IGSF3 | 12 | 1 | 1.78692056219213 | 1.94238771575521 |
| 750 | PTGFRN | 8 | 1 | -1.56052003820307 | 1.8953999437673 |
| 751 | CD101 | 4 | 1 | -3.68424032647975 | -0.0602749429361941 |
| 752 | AL445231.1 | 16 | 1 | -4.418023809985 | 2.38325943130087 |
| 753 | TTF2 | 16 | 1 | -4.48063491304286 | 2.33658994811605 |
| 754 | TRIM45 | 7 | 1 | -3.40406821687586 | -2.84220682007242 |
| 755 | VTCN1 | 11 | 1 | -4.02313683946498 | -1.97307060581614 |
| 756 | AL358072.1 | 3 | 1 | -3.36059688051112 | 0.186184779582727 |
| 757 | MAN1A2 | 9 | 1 | 0.793218508168385 | 2.4715432085378 |
| 758 | TENT5C | 7 | 1 | -2.69042275865443 | -3.90795360428263 |
| 759 | GDAP2 | 2 | 1 | 0.301841035290883 | 2.29760111945699 |
| 760 | WDR3 | 3 | 1 | -2.62623737771876 | 0.50576414245199 |
| 761 | SPAG17 | 11 | 1 | -3.62193892915614 | -2.17990110737253 |
| 762 | WARS2 | 11 | 1 | -3.31205962617762 | -1.88639222008158 |
| 763 | AL359915.2 | 3 | 1 | -2.54501412828334 | 0.866456524310815 |
| 764 | ZNF697 | 1 | 1 | 3.85863853971593 | 1.8368860639913 |
| 765 | PHGDH | 4 | 1 | -4.49420808275111 | 0.13120462077688 |
| 766 | NOTCH2 | 4 | 1 | -4.57521556337245 | 0.135754720149743 |
| 767 | SEC22B | 4 | 1 | -3.67328213174708 | -0.69890682321955 |
| 768 | NBPF26 | 4 | 1 | -4.55732272584803 | 0.409101263461816 |
| 769 | LINC00623 | 10 | 1 | 3.67722226659887 | -1.71331958395411 |
| 770 | FAM72B | 16 | 1 | -4.30311320741542 | 3.36541833060812 |
| 771 | SRGAP2C | 5 | 1 | 4.0602066663944 | 0.107952252803552 |
| 772 | AC244021.1 | 5 | 1 | 3.89380146543615 | -0.627950622858298 |
| 773 | AC239800.2 | 18 | 1 | 5.59306265394323 | -0.524405344547522 |
| 774 | AC239800.3 | 18 | 1 | 5.19104649107091 | -0.637598796905768 |
| 775 | FAM72C | 16 | 1 | -4.26450942476161 | 3.38361825126241 |
| 776 | AC245595.1 | 5 | 1 | 2.53198243658178 | -0.189039512695563 |
| 777 | NBPF15 | 17 | 1 | 1.63528086225622 | 0.27159608977865 |
| 778 | LINC01632 | 5 | 1 | 3.18460632841222 | -0.706227957429183 |
| 779 | SRGAP2B | 5 | 1 | 3.75364853422277 | -0.12470130545069 |
| 780 | FAM72D | 16 | 1 | -4.28930304963954 | 3.3691638864858 |
| 781 | AC245014.3 | 19 | 1 | -4.23479746301539 | 0.852673784671533 |
| 782 | NBPF20 | 12 | 1 | 2.98829819242589 | 2.38280309814047 |
| 783 | RNF115 | 17 | 1 | 1.57678522150152 | -0.095806165756476 |
| 784 | POLR3C | 17 | 1 | 0.927871003552602 | -0.280860050739539 |
| 785 | NUDT17 | 17 | 1 | -0.118501246169879 | 0.0535325564725279 |
| 786 | RBM8A | 13 | 1 | -0.289407033667638 | -1.12602047544886 |
| 787 | LIX1L-AS1 | 1 | 1 | 2.29037810842626 | 0.704900876460779 |
| 788 | LIX1L | 1 | 1 | 2.6916444448673 | 0.982944027362573 |
| 789 | ANKRD34A | 1 | 1 | 2.88893915693395 | 0.704092160640466 |
| 790 | POLR3GL | 10 | 1 | 3.85638023893468 | -1.18272446018626 |
| 791 | TXNIP | 2 | 1 | 0.0722354499541978 | 0.802793399272668 |
| 792 | LINC01719 | 17 | 1 | 1.60663892309301 | 0.155736223636377 |
| 793 | NBPF10 | 4 | 1 | -4.48534701784022 | -0.32724104744364 |
| 794 | NOTCH2NL | 4 | 1 | -4.42714951951869 | 0.118234650073755 |
| 795 | NBPF12 | 1 | 1 | 1.76162435094945 | 0.964555398402917 |
| 796 | PRKAB2 | 5 | 1 | 3.92712141553991 | 0.357710734782922 |
| 797 | AC242426.2 | 17 | 1 | 2.24584080259435 | 0.119149938998926 |
| 798 | CHD1L | 4 | 1 | -4.45392535646327 | 0.315284148631799 |
| 799 | LINC00624 | 12 | 1 | 1.89079381506078 | 1.59751166480612 |
| 800 | BCL9 | 17 | 1 | 1.60772301237218 | 0.137766138492334 |
| 801 | ACP6 | 3 | 1 | -3.33866046388514 | -0.865864585311902 |
| 802 | GPR89B | 7 | 1 | -2.2130517812527 | -2.9174309812205 |
| 803 | NBPF14 | 4 | 1 | -4.3179671617306 | 0.152144090114343 |

| 804 | PDE4DIP | 5 | 1 | 4.41089893858068 | 0.501128927646387 |
| --- | --- | --- | --- | --- | --- |
| 805 | NBPF9 | 17 | 1 | 1.46746219675176 | 0.589381948886621 |
| 806 | AC245297.3 | 15 | 1 | 4.60947157423131 | -0.256917043747199 |
| 807 | AC245297.2 | 1 | 1 | 4.67794586698644 | 0.756831304012048 |
| 808 | NBPF19 | 4 | 1 | -4.51470588167079 | 0.166399613795984 |
| 809 | HIST2H2BF | 19 | 1 | -5.76226352174647 | 1.67501200812887 |
| 810 | HIST2H3D | 19 | 1 | -5.08751605470546 | 0.653220788417566 |
| 811 | AC239868.3 | 1 | 1 | 3.60314442197912 | 1.45452632087301 |
| 812 | HIST2H4B | 19 | 1 | -5.70689938981898 | 1.62076915877889 |
| 813 | AC239868.2 | 17 | 1 | 1.86582018415563 | -0.118593557895911 |
| 814 | HIST2H2BE | 1 | 1 | 2.56186605970495 | 0.199193254886377 |
| 815 | HIST2H2AC | 19 | 1 | -5.73004506547816 | 1.69050707000326 |
| 816 | HIST2H2AB | 19 | 1 | -5.73854564149745 | 1.6967481531484 |
| 817 | BOLA1 | 17 | 1 | 0.94295765440099 | -0.406916245044959 |
| 818 | SV2A | 5 | 1 | 4.16850687543981 | -0.0901436648981692 |
| 819 | SF3B4 | 17 | 1 | 0.686170950337575 | -0.54575751644541 |
| 820 | OTUD7B | 17 | 1 | 0.670626118584798 | 0.0715466298444151 |
| 821 | VPS45 | 17 | 1 | 1.67632593195073 | 0.0157934584004759 |
| 822 | PLEKHO1 | 17 | 1 | 1.48602248708837 | -0.46508364302088 |
| 823 | ANP32E | 19 | 1 | -4.36353515107997 | 0.731191293178308 |
| 824 | CA14 | 4 | 1 | -4.77338384111293 | -0.0458564005510927 |
| 825 | APH1A | 17 | 1 | 0.534736707612203 | -0.656305059017432 |
| 826 | C1orf54 | 7 | 1 | -3.0559186788357 | -2.35768364292551 |
| 827 | CIART | 2 | 1 | 0.794024839802907 | 0.686654106555688 |
| 828 | MRPS21 | 6 | 1 | 0.285541668339894 | -1.05260561329294 |
| 829 | PRPF3 | 8 | 1 | -1.72850297410853 | 1.05703319686483 |
| 830 | RPRD2 | 17 | 1 | 1.48018743078344 | 0.222219840465295 |
| 831 | TARS2 | 17 | 1 | 0.578542366429494 | 0.105024949489343 |
| 832 | ADAMTSL4 | 8 | 1 | -1.86908589322932 | 1.37944306510519 |
| 833 | MCL1 | 17 | 1 | -0.0260302277363081 | 0.158807412563074 |
| 834 | ENSA | 6 | 1 | 1.2127431778156 | -0.838638446451914 |
| 835 | GOLPH3L | 13 | 1 | -0.3329600768245 | -0.843337661744845 |
| 836 | CTSK | 17 | 1 | 2.08212472478978 | 0.630938307224023 |
| 837 | ARNT | 17 | 1 | 1.17671693842046 | 0.330515400348413 |
| 838 | CTXND2 | 11 | 1 | -4.34436295946009 | -1.56586311680247 |
| 839 | SETDB1 | 17 | 1 | 0.111803009911702 | 0.0553438820226072 |
| 840 | CERS2 | 6 | 1 | -0.191521957711055 | -0.901837302297172 |
| 841 | MINDY1 | 17 | 1 | 0.570353105469869 | -0.0411043010370852 |
| 842 | PRUNE1 | 17 | 1 | 1.5929762272083 | 0.217523113666284 |
| 843 | C1orf56 | 1 | 1 | 2.39254976789586 | 0.119030491290796 |
| 844 | CDC42SE1 | 17 | 1 | 2.12698389570348 | -0.0712331972735049 |
| 845 | MLLT11 | 10 | 1 | 3.89189697782628 | -1.07969573062826 |
| 846 | GABPB2 | 8 | 1 | -2.07344590146907 | 1.71107043403219 |
| 847 | SEMA6C | 10 | 1 | 3.36074425260656 | -1.41839431148936 |
| 848 | SCNM1 | 17 | 1 | 1.38726104776494 | -0.46812616211344 |
| 849 | LYSMD1 | 5 | 1 | 2.97969676534765 | -0.416617139400733 |
| 850 | VPS72 | 17 | 1 | 0.684637501641438 | -0.615551336826575 |
| 851 | PIP5K1A | 17 | 1 | 0.495274677678273 | 0.106169239459741 |
| 852 | PSMD4 | 6 | 1 | 0.17730693499677 | -0.774224876584304 |
| 853 | AL391069.2 | 11 | 1 | -3.47371147592433 | -1.07304586392809 |
| 854 | ZNF687 | 17 | 1 | -0.00753979404337601 | 0.0741387166364073 |
| 855 | PI4KB | 17 | 1 | 0.984418883725331 | -0.0809352241175295 |
| 856 | RFX5 | 11 | 1 | -3.84313748796351 | -2.14560149532725 |
| 857 | AL391069.3 | 11 | 1 | -3.94357346017726 | -1.95431373936106 |
| 858 | SELENBP1 | 11 | 1 | -3.92832182367213 | -2.11326394897867 |
| 859 | PSMB4 | 6 | 1 | 0.0353234855376939 | -0.914653356345189 |
| 860 | POGZ | 17 | 1 | 1.22097183744542 | 0.23879887240957 |
| 861 | CGN | 17 | 1 | 1.74586524049871 | 0.319244281230676 |
| 862 | TUFT1 | 1 | 1 | 3.56820346395604 | 1.68882097381185 |
| 863 | SNX27 | 17 | 1 | 0.939481988354848 | 0.0916752971989988 |
| 864 | CELF3 | 5 | 1 | 2.9083888677799 | -0.763629107894194 |
| 865 | RIIAD1 | 6 | 1 | 2.38173820058935 | -1.73909495693613 |
| 866 | AL589765.7 | 6 | 1 | 2.54949571172826 | -1.17676766139437 |
| 867 | MRPL9 | 6 | 1 | -0.176900863841846 | -1.02015899789501 |
| 868 | TDRKH | 17 | 1 | 1.63995482008092 | 0.28909458297323 |
| 869 | C2CD4D | 17 | 1 | 1.48980332891576 | -0.188957854332221 |
| 870 | THEM4 | 15 | 1 | 4.84367157499425 | 0.398968831477868 |

| 871 | S100A10 | 6 | 1 | 1.73210813085668 | -2.45599804741312 |
| --- | --- | --- | --- | --- | --- |
| 872 | S100A11 | 14 | 1 | -6.25423024614222 | 0.0247300423962949 |
| 873 | TCHH | 17 | 1 | 1.68711711446874 | 0.51576306003164 |
| 874 | LOR | 15 | 1 | 5.26396013776891 | -0.0573829255716921 |
| 875 | S100A6 | 6 | 1 | 1.06098689119451 | -2.96122489792277 |
| 876 | S100A4 | 13 | 1 | -1.67062424142726 | -2.53514109474589 |
| 877 | S100A2 | 7 | 1 | -2.3454854341305 | -3.71775112968851 |
| 878 | S100A13 | 13 | 1 | -1.53823123891719 | -2.6865172229426 |
| 879 | AL162258.2 | 11 | 1 | -4.03635929544337 | -1.41811154705454 |
| 880 | CHTOP | 3 | 1 | -2.81869576890834 | 0.080864862380731 |
| 881 | SNAPIN | 13 | 1 | -0.55876581390269 | -1.52635328394343 |
| 882 | ILF2 | 6 | 1 | -0.0761271568096419 | -0.922776228906405 |
| 883 | INTS3 | 2 | 1 | -0.0882982911623259 | 1.95599235671591 |
| 884 | GATAD2B | 17 | 1 | 1.49470497648351 | 0.234296814380395 |
| 885 | AL358472.5 | 17 | 1 | -0.0754071476019164 | -0.331386610092414 |
| 886 | DENND4B | 17 | 1 | 1.64539243261449 | -0.254763527931464 |
| 887 | SLC39A1 | 11 | 1 | -3.1162180753506 | -1.49504958015848 |
| 888 | AL358472.4 | 5 | 1 | 3.15240146200292 | -0.963974869968188 |
| 889 | CREB3L4 | 6 | 1 | -0.0707014652050276 | -0.857753059984934 |
| 890 | JTB | 6 | 1 | 1.29403234998815 | -0.794094219030631 |
| 891 | AL358472.2 | 15 | 1 | 5.26580263654821 | 0.146595732150781 |
| 892 | RAB13 | 11 | 1 | -3.74762533624537 | -1.45524160486628 |
| 893 | RPS27 | 21 | 1 | -0.751972243384196 | -4.34609423500468 |
| 894 | TPM3 | 17 | 1 | 0.735332563325093 | -0.110598369659674 |
| 895 | C1orf43 | 17 | 1 | 0.391860499783681 | -0.892301295110774 |
| 896 | UBAP2L | 17 | 1 | 0.910073414250539 | 0.258859650073755 |
| 897 | HAX1 | 6 | 1 | 0.995280399724172 | -0.859727106215251 |
| 898 | ATP8B2 | 17 | 1 | 1.75147344152562 | 0.552606121478784 |
| 899 | SHE | 15 | 1 | 6.06197191755407 | 0.647322074352014 |
| 900 | UBE2Q1 | 17 | 1 | 1.09824766199224 | -0.0405271492617251 |
| 901 | ADAR | 17 | 1 | 1.43210960905187 | 0.279496923862207 |
| 902 | KCNN3 | 1 | 1 | 4.6210091261112 | 0.945309058604944 |
| 903 | PMVK | 17 | 1 | 1.77182389776342 | -0.690259128035796 |
| 904 | PBXIP1 | 7 | 1 | -3.17967031915553 | -3.09417210441996 |
| 905 | PYGO2 | 17 | 1 | 0.854048982068227 | -0.117961867870581 |
| 906 | AL451085.2 | 17 | 1 | 1.19099821130864 | -0.477524384083045 |
| 907 | SHC1 | 4 | 1 | -3.85401771982081 | -0.242789014400733 |
| 908 | CKS1B | 16 | 1 | -4.46012423951991 | 2.61079515594076 |
| 909 | FLAD1 | 17 | 1 | 0.51122827808492 | -0.695453806938422 |
| 910 | ADAM15 | 14 | 1 | -5.10828851182826 | -0.0197487912790896 |
| 911 | EFNA4 | 11 | 1 | -4.02785752733119 | -1.25707040649821 |
| 912 | EFNA3 | 5 | 1 | 3.67342449705236 | -0.797144926311267 |
| 913 | EFNA1 | 10 | 1 | 4.61584545652501 | -2.22564195019175 |
| 914 | SLC50A1 | 17 | 1 | 1.61627890150182 | -0.336936875404608 |
| 915 | DPM3 | 13 | 1 | -0.426096990898921 | -1.01119436246325 |
| 916 | KRTCAP2 | 6 | 1 | 0.891180649205373 | -1.20592157465388 |
| 917 | TRIM46 | 5 | 1 | 3.21531273405187 | -0.380995317520392 |
| 918 | MUC1 | 6 | 1 | 2.39600850622289 | -0.936630598427546 |
| 919 | AC234582.1 | 11 | 1 | -3.97148726899989 | -1.46046177965571 |
| 920 | THBS3 | 6 | 1 | 2.26015473882787 | -1.16425247413088 |
| 921 | MTX1 | 17 | 1 | 0.411447956963704 | -0.76334622425009 |
| 922 | GBA | 17 | 1 | 0.856963053151296 | -0.293658240856421 |
| 923 | FAM189B | 17 | 1 | 1.34832097570531 | -0.251265212120307 |
| 924 | SCAMP3 | 17 | 1 | 1.27497948686712 | -0.191435142578375 |
| 925 | CLK2 | 17 | 1 | 0.23902745525472 | 0.190743462024438 |
| 926 | HCN3 | 5 | 1 | 4.21663929502599 | 0.454545275150049 |
| 927 | PKLR | 1 | 1 | 2.4962840227329 | 0.687163726268518 |
| 928 | FDPS | 6 | 1 | 1.8935544637882 | -1.53240697008539 |
| 929 | RUSC1 | 5 | 1 | 2.96349073927038 | -0.51610390764643 |
| 930 | ASH1L | 17 | 1 | 1.48758949319951 | 0.347830668864953 |
| 931 | ASH1L-AS1 | 17 | 1 | 0.531106129094289 | 0.0871136941296934 |
| 932 | MSTO1 | 17 | 1 | 1.1633321193897 | 0.0514553346021055 |
| 933 | YY1AP1 | 17 | 1 | 1.53516544382207 | -0.0325661740915896 |
| 934 | DAP3 | 6 | 1 | 0.130388244792149 | -0.730599760236037 |
| 935 | GON4L | 17 | 1 | 0.817564025326894 | 0.0806627430302976 |
| 936 | SYT11 | 1 | 1 | 3.2022633699619 | 0.749765888629663 |
| 937 | RIT1 | 17 | 1 | 0.765942707463429 | 0.04454656022619 |

| 938 | KHDC4 | 17 | 1 | 1.26216699163549 | 0.402390018878686 |
| --- | --- | --- | --- | --- | --- |
| 939 | ARHGEF2 | 17 | 1 | 0.621864095135854 | -0.0452761493342043 |
| 940 | AL355388.2 | 4 | 1 | -3.83053301294215 | -0.408976419986975 |
| 941 | SSR2 | 6 | 1 | -0.074102625445201 | -1.01214332056214 |
| 942 | UBQLN4 | 17 | 1 | 1.04385294000737 | 0.171895042834985 |
| 943 | LAMTOR2 | 6 | 1 | 1.72438015024297 | -0.785113840522063 |
| 944 | MEX3A | 17 | 1 | 0.812322392865346 | 0.242908851085412 |
| 945 | LMNA | 17 | 1 | 0.581624224587605 | -0.114830359043372 |
| 946 | SEMA4A | 5 | 1 | 4.38048101942174 | 0.36228634494375 |
| 947 | SLC25A44 | 1 | 1 | 3.70340587179296 | 1.50082506316732 |
| 948 | PMF1 | 13 | 1 | -0.38898848016627 | -0.886504106381726 |
| 949 | SMG5 | 17 | 1 | 0.801253333493398 | 0.162787691531885 |
| 950 | GLMP | 7 | 1 | -2.09640286882289 | -2.65680764538218 |
| 951 | CCT3 | 6 | 1 | -0.0144047292030592 | -0.787729597808612 |
| 952 | TSACC | 4 | 1 | -3.95233152826197 | -0.535327031435263 |
| 953 | C1orf61 | 4 | 1 | -4.6686374994076 | -1.88145290237833 |
| 954 | MEF2D | 17 | 1 | 1.25535072366826 | 0.189001695094812 |
| 955 | IQGAP3 | 16 | 1 | -4.48024390657313 | 2.93917144912313 |
| 956 | NAXE | 13 | 1 | -0.469639495209529 | -1.4567136607783 |
| 957 | GPATCH4 | 4 | 1 | -3.9395537229336 | -0.130273684086096 |
| 958 | NES | 11 | 1 | -3.7860760541714 | -1.13463641625811 |
| 959 | CRABP2 | 11 | 1 | -4.29447101076014 | -1.67143724781443 |
| 960 | ISG20L2 | 17 | 1 | 0.778891101285146 | 0.0864763416631101 |
| 961 | RRNAD1 | 8 | 1 | -0.639978900746181 | -0.125769718708289 |
| 962 | MRPL24 | 17 | 1 | 0.604851975842641 | -0.710098295630705 |
| 963 | HDGF | 3 | 1 | -3.30344747026332 | -0.959946162225497 |
| 964 | PRCC | 17 | 1 | 0.632000341817067 | -0.234983249725592 |
| 965 | NTRK1 | 18 | 1 | 6.31748940031163 | -2.44269393307139 |
| 966 | INSRR | 18 | 1 | 6.27139355222814 | -2.39886592251231 |
| 967 | ARHGEF11 | 1 | 1 | 3.8833587316715 | 1.52183522361349 |
| 968 | ETV3 | 17 | 1 | 1.36664153616063 | 0.570680276332605 |
| 969 | KIRREL1 | 11 | 1 | -4.08556769807704 | -1.73228119236399 |
| 970 | IFI16 | 14 | 1 | -6.58948539216883 | -0.439361705602896 |
| 971 | AIM2 | 7 | 1 | -2.41667793710597 | -3.27868710380961 |
| 972 | CADM3 | 5 | 1 | 4.48650766889684 | 0.353102461276758 |
| 973 | CADM3-AS1 | 1 | 1 | 3.26612474004857 | 1.43032397407125 |
| 974 | ACKR1 | 5 | 1 | 3.2040169386112 | 0.42656149047445 |
| 975 | DUSP23 | 11 | 1 | -3.86995218713649 | -1.83410207849909 |
| 976 | CFAP45 | 13 | 1 | -0.431470796660258 | -3.29969654900004 |
| 977 | TAGLN2 | 7 | 1 | -2.60504172761805 | -2.42883978706766 |
| 978 | IGSF9 | 17 | 1 | 1.51091338674657 | -0.265107973636878 |
| 979 | PIGM | 17 | 1 | 0.940058246060536 | 0.269233242450464 |
| 980 | KCNJ9 | 5 | 1 | 4.28211094419591 | 0.461193815646875 |
| 981 | IGSF8 | 6 | 1 | 1.99221731702916 | -2.73019038063456 |
| 982 | ATP1A2 | 14 | 1 | -5.88678143937953 | 0.115904704509485 |
| 983 | PEA15 | 5 | 1 | 3.06026269476049 | -0.624396278681052 |
| 984 | DCAF8 | 17 | 1 | -0.00578957815535264 | 0.0824878968579649 |
| 985 | PEX19 | 13 | 1 | -0.318915464178397 | -0.796796566964877 |
| 986 | COPA | 2 | 1 | -0.276266521037294 | 1.2679425634725 |
| 987 | NCSTN | 7 | 1 | -2.96420310457118 | -3.27262912613322 |
| 988 | NHLH1 | 7 | 1 | -2.41366718728907 | -3.75807414871622 |
| 989 | VANGL2 | 17 | 1 | 1.59911359827153 | 0.531767145572412 |
| 990 | F11R | 2 | 1 | -0.76661174018748 | 1.07360471862386 |
| 991 | TSTD1 | 6 | 1 | 1.87778808157079 | -1.75629107576777 |
| 992 | ARHGAP30 | 8 | 1 | -2.15628813226588 | 1.38743056434225 |
| 993 | KLHDC9 | 17 | 1 | 1.59848619978063 | -0.46014390808512 |
| 994 | PFDN2 | 17 | 1 | 1.57744373361699 | -0.601654871525061 |
| 995 | NIT1 | 17 | 1 | 0.451849236890004 | -0.760153963388693 |
| 996 | DEDD | 17 | 1 | 1.40897227804296 | -0.115016385139716 |
| 997 | UFC1 | 6 | 1 | -0.143108017937018 | -1.00196304840018 |
| 998 | USP21 | 17 | 1 | 1.09904910604589 | -0.191372080864203 |
| 999 | PPOX | 17 | 1 | 1.58429266492956 | -0.351329787792456 |
| 1000 | B4GALT3 | 17 | 1 | 1.57979752103917 | -0.493182822288764 |
| 1001 | NDUFS2 | 17 | 1 | 0.37189425508611 | -0.770810528816474 |
| 1002 | PCP4L1 | 15 | 1 | 5.74401117841832 | 0.640972033916223 |
| 1003 | SDHC | 6 | 1 | -0.00101278741724686 | -0.791763565661204 |
| 1004 | CFAP126 | 13 | 1 | -0.205597914949729 | -3.14786182266642 |

| 1005 | DUSP12 | 17 | 1 | 1.54637195150487 | -0.563404007972968 |
| --- | --- | --- | --- | --- | --- |
| 1006 | ATF6 | 17 | 1 | 1.43377126733892 | 0.376361028133142 |
| 1007 | NOS1AP | 18 | 1 | 5.9671833662235 | -1.71402738911082 |
| 1008 | C1orf226 | 1 | 1 | 1.81007219831579 | 1.16799224990438 |
| 1009 | SPATA46 | 1 | 1 | 2.21440078298681 | 0.962749854503381 |
| 1010 | UHMK1 | 17 | 1 | 0.844958081647084 | 0.181480184970605 |
| 1011 | UAP1 | 17 | 1 | 0.806336536809133 | -0.607966973604453 |
| 1012 | DDR2 | 12 | 1 | 2.49939705412023 | 1.81922830718588 |
| 1013 | HSD17B7 | 6 | 1 | 2.36538840810888 | -1.58287267309595 |
| 1014 | RGS5 | 14 | 1 | -4.8931887002743 | 0.0236875571591734 |
| 1015 | RGS5.1 | 4 | 1 | -3.97572635133631 | 0.0756423034055113 |
| 1016 | NUF2 | 16 | 1 | -4.42043803651698 | 3.06866850036215 |
| 1017 | PBX1 | 1 | 1 | 3.71793581525914 | 0.515435711322534 |
| 1018 | RXRG | 15 | 1 | 4.52802731077306 | -0.112577124656928 |
| 1019 | MGST3 | 6 | 1 | 2.26106978933446 | -2.43361602646281 |
| 1020 | ALDH9A1 | 11 | 1 | -3.57182429750331 | -1.52759961706568 |
| 1021 | TMCO1 | 6 | 1 | 0.222362652226613 | -0.985628984095347 |
| 1022 | UCK2 | 3 | 1 | -3.39096997697718 | 1.12080325263571 |
| 1023 | FAM78B | 7 | 1 | -2.72860549409754 | -3.73581515175272 |
| 1024 | AL626787.1 | 2 | 1 | -0.702285334662272 | 1.16193021911215 |
| 1025 | AL390115.1 | 7 | 1 | -2.59890411813624 | -3.6540556989329 |
| 1026 | POGK | 17 | 1 | 1.59629704038732 | 0.465842143474328 |
| 1027 | TADA1 | 17 | 1 | 1.18430568258397 | -0.00678031069208396 |
| 1028 | ILDR2 | 4 | 1 | -4.66001676042445 | -0.0508843503611208 |
| 1029 | POU2F1 | 1 | 1 | 3.3987600950443 | 2.43252123969625 |
| 1030 | CREG1 | 17 | 1 | 1.5463236717426 | -0.147799595417273 |
| 1031 | MPZL1 | 17 | 1 | 1.66610015909307 | 0.295828954158532 |
| 1032 | MPC2 | 6 | 1 | 1.31332136671178 | -0.93687591251899 |
| 1033 | DCAF6 | 17 | 1 | 1.28863848726384 | 0.190924660144555 |
| 1034 | GPR161 | 17 | 1 | 0.789828195973561 | 0.112616316257226 |
| 1035 | TIPRL | 17 | 1 | 1.7378326801502 | -0.504440172733557 |
| 1036 | SFT2D2 | 2 | 1 | -0.280408583775832 | 0.2904080309255 |
| 1037 | TBX19 | 7 | 1 | -2.18444143255122 | -2.74888430458475 |
| 1038 | LINC00626 | 18 | 1 | 6.14855124036901 | -2.78655336719919 |
| 1039 | ATP1B1 | 10 | 1 | 3.79944635908239 | -1.39748738628794 |
| 1040 | NME7 | 17 | 1 | 0.834832325383351 | -0.0543797455446841 |
| 1041 | BLZF1 | 17 | 1 | 0.840419187947438 | 0.236062661586511 |
| 1042 | CCDC181 | 17 | 1 | 0.774663463040517 | -0.127553149284613 |
| 1043 | SLC19A2 | 8 | 1 | -1.29295609910853 | 1.78845585959982 |
| 1044 | C1orf112 | 16 | 1 | -4.69658778627284 | 2.24067820685934 |
| 1045 | METTL18 | 17 | 1 | 0.942545786305592 | -0.439724548877967 |
| 1046 | SCYL3 | 17 | 1 | 1.36652530710332 | 0.353340045390832 |
| 1047 | KIFAP3 | 10 | 1 | 3.49303413908117 | -1.43504463058878 |
| 1048 | GORAB | 17 | 1 | 0.657914951249288 | 0.094646588741052 |
| 1049 | PRRX1 | 14 | 1 | -5.61288522203334 | 0.109496609149682 |
| 1050 | FMO4 | 11 | 1 | -3.96775077302821 | -1.36395750862528 |
| 1051 | PRRC2C | 17 | 1 | 0.869372144147084 | 0.137384668765771 |
| 1052 | MYOCOS | 12 | 1 | 2.4160826353275 | 1.99864496368002 |
| 1053 | METTL13 | 17 | 1 | 0.651884868546651 | -0.264906688751471 |
| 1054 | DNM3 | 15 | 1 | 5.17472149412267 | -0.173529788078559 |
| 1055 | DNM3OS | 1 | 1 | 3.11368300001256 | 1.74062360900472 |
| 1056 | PIGC | 6 | 1 | 1.36082304994695 | -0.729476644815695 |
| 1057 | SUCO | 17 | 1 | 0.701045885487721 | 0.0259046711308836 |
| 1058 | PRDX6 | 11 | 1 | -3.79981277902491 | -1.41584877592493 |
| 1059 | ANKRD45 | 17 | 1 | 1.14259256403081 | -0.0824475131647707 |
| 1060 | KLHL20 | 17 | 1 | 1.06723870317571 | 0.124195233760583 |
| 1061 | CENPL | 16 | 1 | -4.68574546296962 | 2.89076818603109 |
| 1062 | DARS2 | 16 | 1 | -4.41793893297084 | 2.99211706298422 |
| 1063 | ZBTB37 | 17 | 1 | 1.49308408777349 | 0.388889089999902 |
| 1064 | RC3H1 | 1 | 1 | 2.16756941358678 | 0.981271044193017 |
| 1065 | RABGAP1L | 5 | 1 | 4.37481190244787 | 0.221533433376062 |
| 1066 | CACYBP | 6 | 1 | 1.70944775621526 | -0.867385707022441 |
| 1067 | MRPS14 | 6 | 1 | -0.190209225014522 | -0.980515278102648 |
| 1068 | TNR | 18 | 1 | 6.11675669233434 | -2.18591116291453 |
| 1069 | COP1 | 17 | 1 | 1.13692833463781 | -0.0836828194277407 |
| 1070 | AL590723.1 | 17 | 1 | 1.67155028860204 | 0.546193019328821 |
| 1071 | PAPPA2 | 6 | 1 | 2.37937881033056 | -0.946465234430325 |

| 1072 | ASTN1 | 1 | 1 | 3.15697504560582 | 0.964257732806909 |
| --- | --- | --- | --- | --- | --- |
| 1073 | BRINP2 | 6 | 1 | 2.3065171388828 | -1.81808118444849 |
| 1074 | RASAL2 | 1 | 1 | 4.82992245237462 | 0.784598723827112 |
| 1075 | C1orf220 | 12 | 1 | 2.83184243719213 | 2.56418146270345 |
| 1076 | RALGPS2 | 17 | 1 | 1.24033321420781 | 0.0533759750707029 |
| 1077 | ANGPTL1 | 4 | 1 | -4.58132813890345 | -0.72888620120455 |
| 1078 | FAM20B | 1 | 1 | 1.5162419227802 | 1.81470288413595 |
| 1079 | TOR3A | 6 | 1 | -0.234420822196378 | -0.80900850814749 |
| 1080 | ABL2 | 17 | 1 | 1.29551316778295 | 0.243023411212671 |
| 1081 | SOAT1 | 16 | 1 | -3.68418453653224 | 1.87560977119039 |
| 1082 | TOR1AIP2 | 17 | 1 | 1.62187375108831 | 0.223083154140222 |
| 1083 | AL353708.3 | 4 | 1 | -3.62450455148585 | 0.299577847896325 |
| 1084 | TOR1AIP1 | 17 | 1 | 0.562871172829793 | 0.033164278445947 |
| 1085 | CEP350 | 17 | 1 | 1.30720700304143 | 0.401747123180139 |
| 1086 | QSOX1 | 5 | 1 | 4.32859827558629 | 0.434339062152612 |
| 1087 | LHX4 | 12 | 1 | 2.39208032171361 | 1.81107391494344 |
| 1088 | ACBD6 | 17 | 1 | 1.46025921384923 | -0.302092417301428 |
| 1089 | XPR1 | 1 | 1 | 4.61887432615392 | 1.07890738624166 |
| 1090 | KIAA1614 | 2 | 1 | -0.478790641025378 | 1.56617249625753 |
| 1091 | STX6 | 17 | 1 | 0.622243776723073 | 0.336568013606775 |
| 1092 | IER5 | 7 | 1 | -3.46136043985255 | -3.49171028953959 |
| 1093 | CACNA1E | 15 | 1 | 5.26008345167272 | 0.58200480121206 |
| 1094 | GLUL | 7 | 1 | -3.54223631341822 | -3.00750718933512 |
| 1095 | RGS16 | 4 | 1 | -4.66195319612391 | -1.88462231975962 |
| 1096 | LINC01686 | 4 | 1 | -4.6848203988827 | -1.78802017790247 |
| 1097 | NPL | 4 | 1 | -4.87388204057582 | -1.78341089111735 |
| 1098 | DHX9 | 8 | 1 | -1.62623439748652 | 1.78949703353475 |
| 1099 | LAMC1 | 14 | 1 | -4.83765862901576 | 0.17123557704519 |
| 1100 | NMNAT2 | 5 | 1 | 4.20858170072667 | 0.386751667438257 |
| 1101 | SMG7 | 17 | 1 | 1.57801271955602 | 0.269004837451684 |
| 1102 | ARPC5 | 5 | 1 | 2.99749471227758 | -0.731504126610053 |
| 1103 | RGL1 | 17 | 1 | 1.40221275369756 | 0.322023407397973 |
| 1104 | COLGALT2 | 11 | 1 | -3.67172835786708 | -1.62878189903666 |
| 1105 | TSEN15 | 4 | 1 | -3.90938566644557 | 0.21018923896383 |
| 1106 | C1orf21 | 1 | 1 | 4.80679823438756 | 0.855487839160669 |
| 1107 | EDEM3 | 17 | 1 | 1.94663573782079 | 0.285722152171838 |
| 1108 | FAM129A | 15 | 1 | 5.82503105680578 | 0.8004337705953 |
| 1109 | RNF2 | 5 | 1 | 3.41050149480932 | -0.375035329880011 |
| 1110 | TRMT1L | 17 | 1 | 0.776945248051808 | 0.173984185634363 |
| 1111 | SWT1 | 1 | 1 | 2.88992549459569 | 1.02910758632253 |
| 1112 | IVNS1ABP | 17 | 1 | 0.897336140080617 | 0.220932737766015 |
| 1113 | HMCN1 | 4 | 1 | -4.23919175584681 | -1.08631706160475 |
| 1114 | TPR | 17 | 1 | 0.674860015317128 | -0.0178534231798769 |
| 1115 | ODR4 | 17 | 1 | 0.595627739831136 | 0.116412536082971 |
| 1116 | PLA2G4A | 14 | 1 | -6.79435466249354 | -0.422663762392295 |
| 1117 | LINC01351 | 15 | 1 | 4.80796314756505 | 0.201061502872217 |
| 1118 | RGS2 | 18 | 1 | 5.69199396650426 | -1.68923710209299 |
| 1119 | UCHL5 | 17 | 1 | 1.4557691959583 | -0.277487202705634 |
| 1120 | TROVE2 | 17 | 1 | 1.38283587972753 | 0.217267886577356 |
| 1121 | GLRX2 | 5 | 1 | 2.63154746572606 | -0.102460607113135 |
| 1122 | CDC73 | 8 | 1 | -0.312915556327178 | -0.2174826107638 |
| 1123 | B3GALT2 | 1 | 1 | 3.69842196981542 | 0.776574984966028 |
| 1124 | KCNT2 | 1 | 1 | 1.56874789278142 | 1.19122614043783 |
| 1125 | ASPM | 16 | 1 | -4.44730781038172 | 3.24513758796285 |
| 1126 | ZBTB41 | 17 | 1 | 1.79328609983556 | 0.335908070979822 |
| 1127 | CRB1 | 4 | 1 | -5.35520099122889 | -1.86934183460642 |
| 1128 | DENND1B | 7 | 1 | -1.76745413263209 | -3.17761240822245 |
| 1129 | C1orf53 | 7 | 1 | -1.73000119645961 | -3.21531425339152 |
| 1130 | LHX9 | 7 | 1 | -1.77752052266963 | -3.38963304382731 |
| 1131 | NEK7 | 7 | 1 | -2.97870825250514 | -2.50272355896403 |
| 1132 | ATP6V1G3 | 7 | 1 | -3.42869900186427 | -3.486494167866 |
| 1133 | PTPRC | 4 | 1 | -4.45272181947596 | -0.896245411289674 |
| 1134 | MIR181A1HG | 12 | 1 | 1.54848708192937 | 1.88513220923971 |
| 1135 | AC104461.1 | 11 | 1 | -4.31085227449305 | -1.67831610065867 |
| 1136 | ZNF281 | 17 | 1 | 1.57582212965123 | 0.17851211207937 |
| 1137 | KIF14 | 16 | 1 | -4.29611037691005 | 3.37764920371603 |
| 1138 | DDX59 | 17 | 1 | 1.02813114206426 | -0.00471328121591819 |

| 1139 | CAMSAP2 | 1 | 1 | 3.86329199354284 | 2.03655470985006 |
| --- | --- | --- | --- | --- | --- |
| 1140 | INAVA | 4 | 1 | -4.24453137834437 | -0.857706966968072 |
| 1141 | KIF21B | 5 | 1 | 4.32938171903722 | 0.578136221347558 |
| 1142 | TMEM9 | 6 | 1 | -0.147044785515143 | -0.982321432949793 |
| 1143 | TNNI1 | 7 | 1 | -1.82329224069483 | -3.54154597145487 |
| 1144 | PHLDA3 | 10 | 1 | 4.68451668302648 | -2.24870823246409 |
| 1145 | NAV1 | 17 | 1 | 1.47963429967992 | 0.642403975902307 |
| 1146 | IPO9-AS1 | 4 | 1 | -4.65067456682093 | -1.59543673616816 |
| 1147 | AL645504.1 | 2 | 1 | 0.616047277852223 | 2.19179882186483 |
| 1148 | IPO9 | 17 | 1 | 1.15116132776372 | 0.288051263271081 |
| 1149 | TIMM17A | 6 | 1 | 0.145002856656239 | -0.485400154413474 |
| 1150 | RNPEP | 6 | 1 | -0.0434622766531249 | -0.942347902031434 |
| 1151 | ARL8A | 5 | 1 | 3.08314587156408 | -0.621888830484641 |
| 1152 | UBE2T | 16 | 1 | -4.16421459634669 | 2.50933088439535 |
| 1153 | PPP1R12B | 1 | 1 | 3.29689885656469 | 1.10965646880697 |
| 1154 | SYT2 | 14 | 1 | -6.89006875474818 | -0.855029679061663 |
| 1155 | KDM5B | 17 | 1 | 0.718865528508351 | -0.0199245653765322 |
| 1156 | PCAT6 | 10 | 1 | 3.21729995290868 | -1.26950468045641 |
| 1157 | RABIF | 10 | 1 | 2.87615396062963 | -1.53337119442393 |
| 1158 | KLHL12 | 17 | 1 | 0.929205909176991 | -0.239110811771643 |
| 1159 | ADIPOR1 | 17 | 1 | 0.636258378430531 | -0.468512549223197 |
| 1160 | CYB5R1 | 6 | 1 | 2.34456612150304 | -1.47393850904871 |
| 1161 | TMEM183A | 17 | 1 | 0.418796494408772 | -0.550962343038809 |
| 1162 | PPFIA4 | 7 | 1 | -2.75288437326319 | -3.86613260132242 |
| 1163 | BTG2 | 11 | 1 | -3.94882510621913 | -2.02624379021097 |
| 1164 | PRELP | 4 | 1 | -5.25703500230677 | -1.65021411997248 |
| 1165 | ATP2B4 | 4 | 1 | -5.29154132326014 | -1.71220765930582 |
| 1166 | ZBED6 | 9 | 1 | 0.526826038762258 | 2.77508176940511 |
| 1167 | SNRPE | 11 | 1 | -3.32993720491297 | -1.28654111844469 |
| 1168 | SOX13 | 14 | 1 | -6.71076034028895 | -0.991380862118495 |
| 1169 | ETNK2 | 18 | 1 | 5.68988586942785 | -1.90891431194712 |
| 1170 | PLEKHA6 | 1 | 1 | 3.54306723157995 | 0.837057367740381 |
| 1171 | PPP1R15B | 17 | 1 | 0.748022034569905 | 0.211742059169519 |
| 1172 | PIK3C2B | 6 | 1 | 2.25431753675573 | -2.29470454079081 |
| 1173 | MDM4 | 17 | 1 | 1.77331854383581 | 0.38564159053396 |
| 1174 | LRRN2 | 5 | 1 | 4.26722790281408 | 0.0910824694020628 |
| 1175 | NFASC | 5 | 1 | 3.28540159742467 | -0.0981982789652468 |
| 1176 | CNTN2 | 6 | 1 | 1.28580261747472 | -2.93793521744181 |
| 1177 | TMEM81 | 17 | 1 | 1.83484150449865 | 0.287673846660364 |
| 1178 | RBBP5 | 17 | 1 | 1.50910069028966 | 0.137882844386804 |
| 1179 | DSTYK | 17 | 1 | 0.14029766599767 | 0.244256154475915 |
| 1180 | TMCC2 | 7 | 1 | -2.30224226434596 | -3.50067387443949 |
| 1181 | NUAK2 | 11 | 1 | -3.95649884660609 | -1.43548260551859 |
| 1182 | KLHDC8A | 7 | 1 | -3.1578211637295 | -3.32061372620035 |
| 1183 | LEMD1 | 7 | 1 | -2.45440791566737 | -3.25545798164774 |
| 1184 | BLACAT1 | 11 | 1 | -4.4280688615597 | -1.65894488912989 |
| 1185 | AC098936.1 | 11 | 1 | -4.37990401704676 | -1.64641861540247 |
| 1186 | CDK18 | 7 | 1 | -2.822240814761 | -2.57309864384104 |
| 1187 | ELK4 | 17 | 1 | -0.122303337053134 | 0.202008739886987 |
| 1188 | SLC45A3 | 4 | 1 | -5.0759017320431 | -1.67932109457423 |
| 1189 | NUCKS1 | 16 | 1 | -3.97877739389308 | 2.41326464789938 |
| 1190 | SLC41A1 | 8 | 1 | -1.34868942220576 | 1.86897935050558 |
| 1191 | SRGAP2 | 1 | 1 | 3.09550835172765 | 2.14550890105795 |
| 1192 | IKBKE | 19 | 1 | -5.68509791810878 | 1.24145330566 |
| 1193 | RASSF5 | 15 | 1 | 5.32679821531408 | -0.0936135492937685 |
| 1194 | EIF2D | 11 | 1 | -3.40766666849024 | -1.30469395142962 |
| 1195 | DYRK3 | 3 | 1 | -2.26039694269068 | 0.309224382816064 |
| 1196 | MAPKAPK2 | 11 | 1 | -3.91172216852076 | -2.0256023250239 |
| 1197 | YOD1 | 17 | 1 | 0.84684075395696 | 0.165834084926355 |
| 1198 | CD55 | 7 | 1 | -2.65759681184657 | -2.48642991405894 |
| 1199 | CD46 | 17 | 1 | 1.04954041521184 | 0.310406223712671 |
| 1200 | PLXNA2 | 7 | 1 | -3.50889776666529 | -3.11222897392679 |
| 1201 | CAMK1G | 17 | 1 | 1.25026263277166 | 0.0946798481328367 |
| 1202 | LAMB3 | 18 | 1 | 6.05189873258703 | -1.78643052202631 |
| 1203 | UTP25 | 17 | 1 | 0.804516568585561 | 0.116652742801416 |
| 1204 | SYT14 | 5 | 1 | 3.11944533865087 | -0.588728769840491 |
| 1205 | SERTAD4-AS1 | 19 | 1 | -4.92301867921717 | 0.640863076625574 |

| 1206 | SERTAD4 | 18 | 1 | 6.04835726301305 | -2.25031422001292 |
| --- | --- | --- | --- | --- | --- |
| 1207 | HHAT | 11 | 1 | -3.84220789392359 | -1.93362389427592 |
| 1208 | KCNH1 | 18 | 1 | 5.9745247511112 | -2.15326593738962 |
| 1209 | RCOR3 | 17 | 1 | 1.66090048830144 | 0.181666032253015 |
| 1210 | TRAF5 | 13 | 1 | -1.49425791223414 | -2.64925752502848 |
| 1211 | LINC00467 | 11 | 1 | -3.82249830682643 | -2.05317281109263 |
| 1212 | RD3 | 6 | 1 | 1.08194400350683 | -2.8681545100825 |
| 1213 | SLC30A1 | 11 | 1 | -3.59191297014125 | -1.84560160261561 |
| 1214 | NEK2 | 16 | 1 | -4.27504085023768 | 3.29753007072042 |
| 1215 | AC096637.2 | 16 | 1 | -4.40808080156214 | 2.52909506934713 |
| 1216 | LPGAT1 | 17 | 1 | 1.72617043535344 | 0.252942696986902 |
| 1217 | INTS7 | 17 | 1 | 1.2605902103626 | 0.0763936795575498 |
| 1218 | DTL | 19 | 1 | -5.62457297761805 | 1.50933040755819 |
| 1219 | PPP2R5A | 4 | 1 | -4.9619314523495 | -0.447448714794409 |
| 1220 | AL360091.3 | 8 | 1 | -1.80584298093684 | 1.66174663680624 |
| 1221 | TMEM206 | 7 | 1 | -3.42102526147731 | -2.96503077370096 |
| 1222 | NENF | 4 | 1 | -5.07007716615565 | -0.427112086834204 |
| 1223 | AC092803.2 | 10 | 1 | 4.67935253660314 | -0.678178935469878 |
| 1224 | ATF3 | 10 | 1 | 4.38138128797643 | -2.50813542229105 |
| 1225 | NSL1 | 17 | 1 | 0.706376865311788 | -0.361630364479315 |
| 1226 | TATDN3 | 17 | 1 | 1.21456588785283 | -0.263190551819098 |
| 1227 | FLVCR1 | 17 | 1 | 1.10990859548681 | 0.367410437045801 |
| 1228 | VASH2 | 12 | 1 | 2.7744925169193 | 2.79758061545919 |
| 1229 | ANGEL2 | 17 | 1 | 0.15510012189977 | 0.166087166248071 |
| 1230 | RPS6KC1 | 17 | 1 | 1.56207837144964 | 0.259604827342737 |
| 1231 | PROX1 | 14 | 1 | -6.48568223436244 | -1.20475791317393 |
| 1232 | SMYD2 | 6 | 1 | 1.6021315006458 | -0.815455741407168 |
| 1233 | PTPN14 | 14 | 1 | -5.40665029008754 | 0.236054674564111 |
| 1234 | CENPF | 16 | 1 | -4.44474385698207 | 2.55580867904257 |
| 1235 | KCNK2 | 18 | 1 | 5.95698858778112 | -1.72511528355051 |
| 1236 | KCTD3 | 17 | 1 | 1.25933362524144 | 0.276432887492883 |
| 1237 | ESRRG | 18 | 1 | 5.76521421949498 | -1.74665950161387 |
| 1238 | GPATCH2 | 17 | 1 | 0.976255550786183 | -0.021651907982123 |
| 1239 | SPATA17 | 2 | 1 | 0.0685027984821062 | 0.516682402072656 |
| 1240 | RRP15 | 17 | 1 | 0.428740516110585 | -0.501516117872489 |
| 1241 | TGFB2 | 20 | 1 | 3.38758827726476 | -3.78535495621134 |
| 1242 | LYPLAL1 | 17 | 1 | 0.743966176911519 | -0.493651046575797 |
| 1243 | EPRS | 17 | 1 | 2.07674361745946 | 0.0768542446477293 |
| 1244 | BPNT1 | 17 | 1 | 0.952806249066518 | -0.382969900192511 |
| 1245 | IARS2 | 17 | 1 | 0.241020723982976 | 0.0297426738126157 |
| 1246 | RAB3GAP2 | 17 | 1 | 1.74020124952428 | 0.317783967433679 |
| 1247 | MARK1 | 1 | 1 | 3.94417406599157 | 1.57864011901449 |
| 1248 | C1orf115 | 11 | 1 | -3.74642656762965 | -1.21659703475405 |
| 1249 | HLX | 15 | 1 | 5.77321268598668 | 0.633968011317956 |
| 1250 | DUSP10 | 4 | 1 | -4.82315848787196 | -1.62003491741587 |
| 1251 | AL513314.2 | 9 | 1 | 0.987465873166249 | 2.55289925712179 |
| 1252 | TAF1A | 17 | 1 | 0.924154534741567 | -0.297288998188269 |
| 1253 | TAF1A-AS1 | 17 | 1 | 0.555130377217458 | -0.290353759350073 |
| 1254 | MIA3 | 17 | 1 | 1.30171562711828 | -0.0167904935495974 |
| 1255 | AL592148.3 | 17 | 1 | 1.63991047422521 | 0.504074350772607 |
| 1256 | AIDA | 17 | 1 | -0.0715839417732497 | 0.0084563054425596 |
| 1257 | BROX | 17 | 1 | 1.08022667448156 | -0.0679020724909426 |
| 1258 | AL392172.1 | 4 | 1 | -4.2893011422909 | 0.228315845905053 |
| 1259 | TLR5 | 15 | 1 | 6.04311396162145 | 0.729602114139306 |
| 1260 | SUSD4 | 15 | 1 | 5.9941027311527 | 0.663849727092492 |
| 1261 | CAPN8 | 15 | 1 | 6.10553432981603 | 0.832146541057336 |
| 1262 | CAPN2 | 6 | 1 | 0.0188264397823076 | -0.789969816030753 |
| 1263 | TP53BP2 | 2 | 1 | 1.01755095998876 | 0.530714289127099 |
| 1264 | FBXO28 | 17 | 1 | 0.119839176341222 | 0.132712379871118 |
| 1265 | DEGS1 | 17 | 1 | 1.99717403928869 | -0.494120135130179 |
| 1266 | NVL | 17 | 1 | 1.10898770372503 | -0.276562198223364 |
| 1267 | CNIH4 | 11 | 1 | -3.62036035974391 | -1.581868752064 |
| 1268 | WDR26 | 17 | 1 | 1.3085079340183 | 0.444199100909936 |
| 1269 | DNAH14 | 17 | 1 | 1.46087254564397 | 0.262022630153406 |
| 1270 | LBR | 16 | 1 | -3.87781785447962 | 2.52534689086508 |
| 1271 | ENAH | 17 | 1 | 1.58069469015233 | 0.34541942256521 |
| 1272 | SRP9 | 6 | 1 | 1.04280139486425 | -0.865769314737809 |

| 1273 | EPHX1 | 10 | 1 | 2.87087728063695 | -1.89140067917277 |
| --- | --- | --- | --- | --- | --- |
| 1274 | TMEM63A | 14 | 1 | -6.656633600787 | -0.431521489443076 |
| 1275 | PYCR2 | 6 | 1 | 0.0108647493564348 | -0.746474861325514 |
| 1276 | SDE2 | 17 | 1 | 0.825177207394765 | 0.154704467235315 |
| 1277 | H3F3A | 6 | 1 | 1.02781285326116 | -0.84564519596268 |
| 1278 | ACBD3 | 17 | 1 | 1.31482519189946 | -0.0151098809855105 |
| 1279 | LIN9 | 16 | 1 | -4.03461955507167 | 2.29597033637594 |
| 1280 | PARP1 | 13 | 1 | -0.491126179889514 | -0.904468128150147 |
| 1281 | ITPKB | 14 | 1 | -5.77728484590419 | -0.106254919590247 |
| 1282 | PSEN2 | 6 | 1 | -0.00817507524855332 | -0.880567888887417 |
| 1283 | COQ8A | 17 | 1 | 1.29945255796544 | -0.490953608574164 |
| 1284 | CDC42BPA | 17 | 1 | 1.55082418005101 | 0.363873616634118 |
| 1285 | ZNF678 | 17 | 1 | 1.35195948164098 | 0.385483042178857 |
| 1286 | SNAP47 | 17 | 1 | 0.758356109067128 | -0.33306859356333 |
| 1287 | JMJD4 | 6 | 1 | -0.0568357708013792 | -0.851694523514998 |
| 1288 | ARF1 | 17 | 1 | 0.74654127637975 | -0.232545598568213 |
| 1289 | C1orf35 | 17 | 1 | 0.663208856984303 | -0.514629288734686 |
| 1290 | MRPL55 | 6 | 1 | 1.27124728242986 | -0.813151477994216 |
| 1291 | GUK1 | 17 | 1 | 1.92026974241369 | -0.777913055540812 |
| 1292 | IBA57 | 17 | 1 | 0.751384630604909 | 0.183012262759912 |
| 1293 | OBSCN | 17 | 1 | 1.10441495458715 | -0.461596056045783 |
| 1294 | TRIM11 | 17 | 1 | 0.91600133459203 | -0.206892593921912 |
| 1295 | HIST3H2A | 17 | 1 | 0.436372116013692 | -0.0345223985331179 |
| 1296 | HIST3H2BB | 17 | 1 | 1.66592015306585 | 0.0429139294011472 |
| 1297 | RNF187 | 17 | 1 | 1.69137372057073 | -0.357249899925482 |
| 1298 | RHOU | 1 | 1 | 1.62711895982854 | 1.00625242370199 |
| 1299 | RAB4A | 6 | 1 | 2.38404156248205 | -1.4857857070582 |
| 1300 | CCSAP | 1 | 1 | 3.75805164854162 | 0.64512862342428 |
| 1301 | ACTA1 | 6 | 1 | 1.66823674718969 | -2.70191417557169 |
| 1302 | NUP133 | 17 | 1 | 0.831841125889943 | 0.0891109265668272 |
| 1303 | ABCB10 | 17 | 1 | 1.4495083240711 | 0.372233525691736 |
| 1304 | TAF5L | 17 | 1 | 1.13452066461675 | 0.282465354381311 |
| 1305 | URB2 | 17 | 1 | -0.148651205197646 | 0.19519580977987 |
| 1306 | GALNT2 | 7 | 1 | -2.55335687120326 | -3.27675066811014 |
| 1307 | PGBD5 | 17 | 1 | 1.21992196123235 | 0.170357958255517 |
| 1308 | COG2 | 17 | 1 | -0.0820624383247633 | -0.000149890007269504 |
| 1309 | CAPN9 | 7 | 1 | -3.40056322534449 | -2.8332708917277 |
| 1310 | C1orf198 | 3 | 1 | -3.43336342294581 | -0.85651807782222 |
| 1311 | TTC13 | 15 | 1 | 4.71601892988317 | 0.485045329509485 |
| 1312 | ARV1 | 17 | 1 | 1.39101017992132 | -0.307503028930915 |
| 1313 | FAM89A | 4 | 1 | -4.75427125413783 | -2.30600546223093 |
| 1314 | TRIM67 | 18 | 1 | 5.60844160596959 | -1.05921129805018 |
| 1315 | C1orf131 | 17 | 1 | 0.896279707356618 | -0.420891299070609 |
| 1316 | GNPAT | 6 | 1 | 0.149438753529714 | -0.580141081632865 |
| 1317 | EXOC8 | 17 | 1 | 1.48137833158605 | 0.337393061099756 |
| 1318 | SPRTN | 17 | 1 | 0.647516205712483 | 0.0144165911061643 |
| 1319 | EGLN1 | 17 | 1 | 1.22874452154271 | 0.140737430034387 |
| 1320 | AL445524.1 | 4 | 1 | -3.76444743593104 | -0.779525733711016 |
| 1321 | TSNAX | 17 | 1 | 0.651370122834371 | -0.415349139990103 |
| 1322 | SIPA1L2 | 14 | 1 | -6.8557040544308 | -0.59149743539263 |
| 1323 | NTPCR | 6 | 1 | -0.160144843355491 | -0.864981274278653 |
| 1324 | PCNX2 | 1 | 1 | 3.2957863954746 | 1.05468560832571 |
| 1325 | KCNK1 | 4 | 1 | -4.06440757234462 | -0.661610483350051 |
| 1326 | SLC35F3 | 15 | 1 | 5.68722130338781 | -0.146727546276343 |
| 1327 | AL355472.1 | 4 | 1 | -3.90410135705836 | 0.0217122592313169 |
| 1328 | COA6 | 13 | 1 | -0.494847476676776 | -0.982658564271224 |
| 1329 | TARBP1 | 1 | 1 | 3.46263505498998 | 2.26817192214559 |
| 1330 | IRF2BP2 | 1 | 1 | 4.1422174123966 | 2.1041459001882 |
| 1331 | TOMM20 | 17 | 1 | 0.563104525014089 | 0.119038597522485 |
| 1332 | RBM34 | 17 | 1 | 1.57191921751134 | 0.0390244283063291 |
| 1333 | ARID4B | 17 | 1 | 1.08582140485875 | 0.138780371127832 |
| 1334 | GGPS1 | 17 | 1 | 1.05340994875066 | -0.117532177986395 |
| 1335 | TBCE | 17 | 1 | 1.30562461893194 | 0.213778630672204 |
| 1336 | AL357556.4 | 8 | 1 | -2.51421450097926 | 1.45280160087179 |
| 1337 | TBCE.1 | 17 | 1 | 0.247646406098531 | 0.292343274532068 |
| 1338 | B3GALNT2 | 7 | 1 | -3.67329143007167 | -2.70740352493693 |
| 1339 | GNG4 | 17 | 1 | 1.11326970140569 | -0.135117515148413 |

| 1340 | LYST | 5 | 1 | 2.98323585073583 | -0.212024613441718 |
| --- | --- | --- | --- | --- | --- |
| 1341 | NID1 | 14 | 1 | -5.64227651079066 | 0.383747950969446 |
| 1342 | GPR137B | 8 | 1 | -1.01635496337779 | 0.0496769108159422 |
| 1343 | ERO1B | 10 | 1 | 4.66427208463781 | -1.9226183734553 |
| 1344 | LGALS8 | 17 | 1 | 0.701754763528035 | 0.289348141132104 |
| 1345 | HEATR1 | 2 | 1 | -0.193116959378793 | 0.258094326434839 |
| 1346 | ACTN2 | 14 | 1 | -5.45894978959925 | 0.22484053748678 |
| 1347 | MTR | 8 | 1 | -1.52280925233729 | 1.36090244430135 |
| 1348 | RYR2 | 10 | 1 | 4.7170555738651 | -1.84063248258997 |
| 1349 | LINC01139 | 13 | 1 | -0.430571794704272 | -2.69890807491709 |
| 1350 | CHRM3 | 10 | 1 | 4.27744366209142 | -2.69860707146097 |
| 1351 | FMN2 | 1 | 1 | 2.56939579527013 | 1.64132465499471 |
| 1352 | GREM2 | 20 | 1 | 3.33596874754064 | -3.60088168007303 |
| 1353 | RGS7 | 15 | 1 | 4.97521473447912 | -0.0684032283442141 |
| 1354 | FH | 17 | 1 | 0.578361883565114 | -0.74328850132395 |
| 1355 | KMO | 1 | 1 | 1.66284693758123 | 1.14744271415304 |
| 1356 | OPN3 | 13 | 1 | -1.20043562372096 | -2.3066845975535 |
| 1357 | CHML | 17 | 1 | 1.76385213415258 | 0.410637394366968 |
| 1358 | EXO1 | 19 | 1 | -5.49164651353724 | 1.71987642425131 |
| 1359 | MAP1LC3C | 4 | 1 | -4.95523665864833 | -1.68044011217524 |
| 1360 | PLD5 | 11 | 1 | -4.21203134973414 | -2.08675669056345 |
| 1361 | CEP170 | 1 | 1 | 3.74096824209325 | 0.666279093204248 |
| 1362 | SDCCAG8 | 1 | 1 | 2.55181385557287 | 1.11284746306967 |
| 1363 | AKT3 | 1 | 1 | 2.33887197057836 | 1.03346277850698 |
| 1364 | AL591885.1 | 8 | 1 | -2.68213747461207 | 1.595626012264 |
| 1365 | ZBTB18 | 7 | 1 | -3.67470262964137 | -2.8336348376887 |
| 1366 | ADSS | 17 | 1 | 0.844063535138295 | -0.0513129077570559 |
| 1367 | DESI2 | 13 | 1 | -0.528524056271388 | -0.772313459934485 |
| 1368 | COX20 | 5 | 1 | 3.22463728468053 | -0.695848687710059 |
| 1369 | HNRNPU | 8 | 1 | -1.49906097371943 | 1.74720944541525 |
| 1370 | EFCAB2 | 17 | 1 | 1.6220244316303 | 0.0315799273831724 |
| 1371 | KIF26B | 18 | 1 | 6.11565233747594 | -2.13258968216349 |
| 1372 | SMYD3 | 6 | 1 | 2.5531733183109 | -1.49723349434305 |
| 1373 | TFB2M | 13 | 1 | -0.480385586813762 | -0.858891613008273 |
| 1374 | CNST | 1 | 1 | 3.42117716352575 | 1.11875166076254 |
| 1375 | SCCPDH | 17 | 1 | 0.667138472005055 | -0.579263850273383 |
| 1376 | AHCTF1 | 17 | 1 | 1.12357617895238 | 0.233800546107995 |
| 1377 | ZNF695 | 16 | 1 | -4.00111912210353 | 2.2520505346639 |
| 1378 | ZNF670 | 17 | 1 | 0.74434150735967 | -0.14398966652323 |
| 1379 | ZNF669 | 17 | 1 | 1.23546780626409 | -0.123200162472022 |
| 1380 | ZNF124 | 17 | 1 | 1.1185243276798 | 0.30672051089834 |
| 1381 | AL390728.6 | 1 | 1 | 1.9863951353275 | 1.17038644927572 |
| 1382 | ZNF496 | 17 | 1 | 0.128171250029729 | 0.129422918735254 |
| 1383 | TRIM58 | 13 | 1 | -1.37081085164912 | -2.99597535950114 |
| 1384 | OR2L2 | 17 | 1 | 1.54452539960973 | 0.0858972229344724 |
| 1385 | SH3BP5L | 17 | 1 | 1.34369291345708 | 0.125545755801904 |
| 1386 | PGBD2 | 17 | 1 | 0.608356728955434 | 0.118463651118982 |
| 1387 | SH3YL1 | 14 | 1 | -5.64520524461634 | 0.140172497211206 |
| 1388 | ACP1 | 3 | 1 | -2.90414951761134 | -0.989131665946734 |
| 1389 | ALKAL2 | 14 | 1 | -6.5151541085995 | -1.04163007360865 |
| 1390 | TMEM18 | 11 | 1 | -3.27845166643031 | -1.25383408290316 |
| 1391 | AC092159.2 | 4 | 1 | -3.66078303773768 | -0.351046963752997 |
| 1392 | AC116609.3 | 17 | 1 | 0.372094228669332 | 0.355038777766931 |
| 1393 | PXDN | 2 | 1 | -1.33992014844783 | 1.98549880164694 |
| 1394 | MYT1L | 15 | 1 | 4.89674212019079 | 0.585250750957239 |
| 1395 | TRAPPC12 | 5 | 1 | 2.81889702360265 | -0.575007184566748 |
| 1396 | ADI1 | 11 | 1 | -3.60693286378749 | -0.965189783992064 |
| 1397 | RNASEH1 | 8 | 1 | -0.967390641764476 | 0.731306806979883 |
| 1398 | RNASEH1-AS1 | 3 | 1 | -3.12919114549525 | -0.335378392757666 |
| 1399 | RPS7 | 21 | 1 | -0.930669591025188 | -4.41257797104288 |
| 1400 | LINC01249 | 17 | 1 | 2.11712051908605 | 0.475577727733362 |
| 1401 | SOX11 | 1 | 1 | 3.36744309942357 | 0.42826785224508 |
| 1402 | AC010729.1 | 7 | 1 | -3.60464881379969 | -2.61651991230418 |
| 1403 | LINC00487 | 5 | 1 | 3.26313902418248 | -0.637979491771948 |
| 1404 | CMPK2 | 8 | 1 | -1.20041321237452 | -0.251867457451117 |
| 1405 | AC017076.1 | 1 | 1 | 1.93113709966771 | 0.94762398379873 |
| 1406 | RNF144A | 12 | 1 | 2.91224314252965 | 2.65428508895467 |

| 1407 | ID2 | 14 | 1 | -6.52654479463465 | -0.845222271205676 |
| --- | --- | --- | --- | --- | --- |
| 1408 | KIDINS220 | 5 | 1 | 4.59937740842931 | 0.56160856860708 |
| 1409 | MBOAT2 | 5 | 1 | 3.61525203268163 | 0.227383986888635 |
| 1410 | ASAP2 | 9 | 1 | 0.231015160485433 | 2.69597710746359 |
| 1411 | ITGB1BP1 | 5 | 1 | 2.92807938139074 | -0.362491413177741 |
| 1412 | CPSF3 | 3 | 1 | -3.33927176912196 | 0.162043944774377 |
| 1413 | IAH1 | 6 | 1 | 0.959316745206044 | -2.77787123543192 |
| 1414 | ADAM17 | 8 | 1 | -1.58214484174617 | 1.32668365615438 |
| 1415 | AC073195.1 | 4 | 1 | -3.78340767343409 | -0.0753778062479616 |
| 1416 | YWHAQ | 10 | 1 | 3.86776138822667 | -1.45819650513102 |
| 1417 | TAF1B | 16 | 1 | -3.59977410753138 | 1.61238445418905 |
| 1418 | GRHL1 | 18 | 1 | 5.12916733305089 | -0.53182314258982 |
| 1419 | KLF11 | 1 | 1 | 2.82812310735814 | 1.30192197936605 |
| 1420 | RRM2 | 19 | 1 | -4.93204282243617 | 1.75213350432943 |
| 1421 | AC007240.1 | 19 | 1 | -4.9180500360287 | 2.10751284736227 |
| 1422 | C2orf48 | 19 | 1 | -4.97850535829432 | 1.98569120544027 |
| 1423 | HPCAL1 | 10 | 1 | 4.68735195676915 | -0.939658804954779 |
| 1424 | ODC1 | 11 | 1 | -3.24537657220729 | -1.23744855743815 |
| 1425 | NOL10 | 3 | 1 | -2.62041638811 | 0.104886666713464 |
| 1426 | RN7SL832P | 3 | 1 | -2.51252578218348 | 1.09124530929159 |
| 1427 | ATP6V1C2 | 1 | 1 | 1.65080429594152 | 1.1751691259725 |
| 1428 | PDIA6 | 3 | 1 | -3.22581814248927 | -0.545959009947074 |
| 1429 | LINC01954 | 18 | 1 | 6.29467894117467 | -2.32065640312601 |
| 1430 | KCNF1 | 18 | 1 | 6.24806667844884 | -2.35932527405192 |
| 1431 | ROCK2 | 12 | 1 | 1.85643435995214 | 2.46423901694845 |
| 1432 | E2F6 | 2 | 1 | -0.541646555260493 | 1.56809534209799 |
| 1433 | GREB1 | 12 | 1 | 2.7576353697025 | 2.00212492126058 |
| 1434 | LPIN1 | 12 | 1 | 3.26379300634496 | 2.63002552169393 |
| 1435 | MIR3681HG | 19 | 1 | -4.88685105760463 | 0.74724556582998 |
| 1436 | TRIB2 | 15 | 1 | 5.25710798780553 | 0.716210738597619 |
| 1437 | FAM84A | 18 | 1 | 5.32448746244542 | -0.70558586639334 |
| 1438 | NBAS | 1 | 1 | 3.12007571737401 | 1.86553348678182 |
| 1439 | DDX1 | 2 | 1 | 0.572250142499135 | 0.918987409053552 |
| 1440 | MYCNOS | 4 | 1 | -4.62335465867884 | -0.738926499308837 |
| 1441 | MYCN | 14 | 1 | -4.80083440263636 | 0.523880497394311 |
| 1442 | FAM49A | 10 | 1 | 4.04758812467687 | -1.19966621619631 |
| 1443 | VSNL1 | 15 | 1 | 6.0428931860172 | 0.589015737949121 |
| 1444 | SMC6 | 9 | 1 | -0.437808782056644 | 3.05603946822714 |
| 1445 | GEN1 | 16 | 1 | -4.5321481080807 | 2.51685322898458 |
| 1446 | KCNS3 | 19 | 1 | -5.40578673799403 | 0.864550486980188 |
| 1447 | AC079148.1 | 12 | 1 | 2.50371052305333 | 2.49797143119405 |
| 1448 | RDH14 | 8 | 1 | -0.478456258968188 | -0.148494764389289 |
| 1449 | LINC01376 | 13 | 1 | -0.450444907144382 | -3.230053051533 |
| 1450 | LINC00954 | 4 | 1 | -3.67367719133265 | 0.163047686992395 |
| 1451 | TTC32 | 4 | 1 | -3.93823288400538 | -0.0910622916834475 |
| 1452 | AC013400.1 | 16 | 1 | -4.34053157289393 | 2.38096965926717 |
| 1453 | WDR35 | 8 | 1 | -1.6904573293484 | 0.981701270519006 |
| 1454 | MATN3 | 14 | 1 | -4.83585666139491 | 0.357485429225671 |
| 1455 | LAPTM4A | 3 | 1 | -2.62715314348109 | -1.2857031367438 |
| 1456 | SDC1 | 16 | 1 | -4.26457332094081 | 1.89738740104269 |
| 1457 | PUM2 | 12 | 1 | 2.46989943067663 | 3.05104221480917 |
| 1458 | RHOB | 10 | 1 | 4.78932501356237 | -1.0056471518891 |
| 1459 | HS1BP3 | 6 | 1 | 2.06718184034459 | -1.27211181742121 |
| 1460 | LDAH | 3 | 1 | -1.83912001569636 | -0.0688490114824892 |
| 1461 | LINC01830 | 6 | 1 | 1.4645183233463 | -2.78278539997507 |
| 1462 | AC068490.1 | 13 | 1 | -1.04092077930339 | -2.86958430630137 |
| 1463 | AC096570.1 | 11 | 1 | -4.10690377672084 | -1.84620295387675 |
| 1464 | KLHL29 | 15 | 1 | 5.09821774046056 | 0.495432988582361 |
| 1465 | ATAD2B | 9 | 1 | 0.676616266175435 | 2.50997747558187 |
| 1466 | UBXN2A | 8 | 1 | -1.83636473138697 | 1.90454043525289 |
| 1467 | WDCP | 8 | 1 | -1.42634557206996 | 0.64016331809591 |
| 1468 | FKBP1B | 10 | 1 | 3.34590055029027 | -1.8938280187266 |
| 1469 | SF3B6 | 13 | 1 | -0.451719403461291 | -1.85418097597529 |
| 1470 | FAM228B | 5 | 1 | 4.282406583234 | -0.418704047025931 |
| 1471 | TP53I3 | 11 | 1 | -3.63058732469447 | -2.45312772613932 |
| 1472 | ITSN2 | 9 | 1 | 1.87794138471715 | 3.10007895606588 |
| 1473 | NCOA1 | 2 | 1 | 0.632162406846211 | 1.95391596931051 |

| 1474 | PTRHD1 | 7 | 1 | -2.65794013460047 | -2.02974246365 |
| --- | --- | --- | --- | --- | --- |
| 1475 | CENPO | 16 | 1 | -4.5708124490536 | 2.31880940574239 |
| 1476 | ADCY3 | 1 | 1 | 2.58676911871068 | 0.125504986224878 |
| 1477 | AC012073.1 | 16 | 1 | -4.43222259004481 | 2.49958147185873 |
| 1478 | DNAJC27 | 12 | 1 | 2.00433422605626 | 2.28208746093343 |
| 1479 | EFR3B | 5 | 1 | 3.65142680685155 | -0.0690123878138186 |
| 1480 | POMC | 7 | 1 | -3.08148477990992 | -3.83543978554178 |
| 1481 | DNMT3A | 1 | 1 | 3.54701425115697 | 1.08371175902914 |
| 1482 | DTNB | 1 | 1 | 4.56708742658727 | 0.975806132732141 |
| 1483 | ASXL2 | 12 | 1 | 2.68628717939489 | 2.87435306685995 |
| 1484 | KIF3C | 5 | 1 | 4.37612797300451 | 0.275204793391931 |
| 1485 | RAB10 | 9 | 1 | 1.42429722826116 | 2.17870391982626 |
| 1486 | GAREM2 | 5 | 1 | 3.01675750295751 | -0.793161607505572 |
| 1487 | HADHA | 6 | 1 | 1.58987750093572 | -1.12152215761114 |
| 1488 | HADHB | 11 | 1 | -3.09893463571437 | -1.86512426954676 |
| 1489 | SELENOI | 1 | 1 | 3.37402822057836 | 1.77026046889852 |
| 1490 | DRC1 | 5 | 1 | 2.73199440519445 | -1.30747772675921 |
| 1491 | KCNK3 | 5 | 1 | 4.29764534513585 | -0.727625026525748 |
| 1492 | CENPA | 16 | 1 | -4.31866811235316 | 3.29402627128195 |
| 1493 | DPYSL5 | 12 | 1 | 2.18734885732763 | 2.09758986610006 |
| 1494 | MAPRE3 | 5 | 1 | 4.23959614317052 | -0.773014097632659 |
| 1495 | AGBL5 | 3 | 1 | -3.10717462022669 | 0.460286156116235 |
| 1496 | AC013403.2 | 1 | 1 | 1.73378457109563 | 1.07311751025747 |
| 1497 | OST4 | 13 | 1 | -0.952824220255687 | -2.60259471756388 |
| 1498 | KHK | 6 | 1 | 1.90038587133519 | -1.81067876440455 |
| 1499 | TCF23 | 1 | 1 | 1.97768284360997 | 1.54428996223043 |
| 1500 | SLC5A6 | 8 | 1 | -1.85525189359553 | 0.43212105411123 |
| 1501 | ATRAID | 11 | 1 | -3.21317790468104 | -1.41254793030192 |
| 1502 | CAD | 19 | 1 | -4.64620994050868 | 1.51493252891134 |
| 1503 | SLC30A3 | 20 | 1 | 3.52706266920202 | -3.53758369308878 |
| 1504 | MPV17 | 3 | 1 | -2.22578869779475 | -1.00137667280604 |
| 1505 | GTF3C2 | 8 | 1 | -1.599469289378 | 1.52639712470602 |
| 1506 | AC074117.1 | 2 | 1 | -1.03215394933589 | 1.81633581298422 |
| 1507 | EIF2B4 | 9 | 1 | 1.34380079786413 | 2.45608724731039 |
| 1508 | SNX17 | 13 | 1 | -2.57525323350794 | -1.61819420677592 |
| 1509 | ZNF513 | 2 | 1 | 0.172942355080769 | 1.9792336382253 |
| 1510 | PPM1G | 2 | 1 | 0.988351359769032 | 0.497487679897058 |
| 1511 | NRBP1 | 2 | 1 | -0.82589410264857 | 0.89053227084707 |
| 1512 | IFT172 | 12 | 1 | 2.58433653394811 | 1.78321541923116 |
| 1513 | FNDC4 | 18 | 1 | 5.27595879118078 | -1.47228507620264 |
| 1514 | C2orf16 | 12 | 1 | 2.03802253286474 | 2.05795897620748 |
| 1515 | ZNF512 | 9 | 1 | 1.46838547269933 | 2.61091317313741 |
| 1516 | CCDC121 | 3 | 1 | -2.63908241708644 | 0.545918718753564 |
| 1517 | GPN1 | 11 | 1 | -2.80613182504542 | -1.24766044241358 |
| 1518 | SUPT7L | 17 | 1 | -0.0191055836237212 | -0.190551861347449 |
| 1519 | SLC4A1AP | 2 | 1 | 1.07453609983556 | 0.756253615794885 |
| 1520 | LINC01460 | 1 | 1 | 2.74859453718297 | 0.96672798770498 |
| 1521 | MRPL33 | 13 | 1 | -0.51688434203036 | -2.22047577721049 |
| 1522 | RBKS | 13 | 1 | -1.51527403314479 | -1.89770774465967 |
| 1523 | BABAM2 | 13 | 1 | -0.494993642166926 | -0.700561433257353 |
| 1524 | AC092164.1 | 11 | 1 | -3.73017047365077 | -0.991538181127799 |
| 1525 | PPP1CB | 8 | 1 | -1.68941985090144 | 1.88465108054708 |
| 1526 | SPDYA | 1 | 1 | 1.92672277967565 | 1.08045353072714 |
| 1527 | WDR43 | 3 | 1 | -2.52152584512599 | 0.368842498241174 |
| 1528 | CLIP4 | 1 | 1 | 3.87704493085973 | 1.68893541472982 |
| 1529 | ALK | 7 | 1 | -2.74512098749049 | -3.6977339826243 |
| 1530 | YPEL5 | 2 | 1 | 0.451382115288899 | 1.02049268859457 |
| 1531 | LBH | 10 | 1 | 4.7709787039005 | -0.545964314760459 |
| 1532 | LCLAT1 | 9 | 1 | 1.51923944036596 | 2.88232387679647 |
| 1533 | GALNT14 | 6 | 1 | 1.88918425123327 | -2.72166179043223 |
| 1534 | EHD3 | 12 | 1 | 2.07918407003515 | 1.93042935508322 |
| 1535 | XDH | 18 | 1 | 5.19822718183629 | -0.432220324100745 |
| 1536 | MEMO1 | 1 | 1 | 3.37199403326146 | 1.00536860602926 |
| 1537 | DPY30 | 6 | 1 | 0.164101466342137 | -2.23664472919871 |
| 1538 | SPAST | 1 | 1 | 3.3298804907047 | 1.22320689338278 |
| 1539 | AL121658.1 | 4 | 1 | -3.95206473787196 | 0.146919623790491 |
| 1540 | SLC30A6 | 8 | 1 | -1.63301311452754 | 1.37959422248434 |

| 1541 | YIPF4 | 12 | 1 | 2.11874248067968 | 2.22798909324239 |
| --- | --- | --- | --- | --- | --- |
| 1542 | BIRC6 | 9 | 1 | 1.52389003316991 | 2.82174386161398 |
| 1543 | TTC27 | 4 | 1 | -4.41005633790858 | -0.254982932628882 |
| 1544 | LTBP1 | 4 | 1 | -4.12094329316981 | -0.960909682484162 |
| 1545 | RASGRP3 | 10 | 1 | 4.65241934339635 | -2.1112694583552 |
| 1546 | FAM98A | 7 | 1 | -2.79904149492152 | -1.9701025329249 |
| 1547 | AC009414.2 | 10 | 1 | 4.70440413038366 | -1.88974277597834 |
| 1548 | CRIM1 | 20 | 1 | 3.67990066091649 | -3.31517301422526 |
| 1549 | FEZ2 | 13 | 1 | -1.95635972936518 | -2.1852692208903 |
| 1550 | STRN | 1 | 1 | 3.36676122228734 | 1.18211354392599 |
| 1551 | HEATR5B | 1 | 1 | 3.69312597791784 | 0.840721861301172 |
| 1552 | GPATCH11 | 10 | 1 | 3.76091839353673 | -1.23995916468073 |
| 1553 | EIF2AK2 | 8 | 1 | -1.3435597272671 | 1.55406130927633 |
| 1554 | CEBPZ | 2 | 1 | 0.504477396413014 | 0.866583124576318 |
| 1555 | NDUFAF7 | 2 | 1 | -0.819659695223643 | 1.00263573306631 |
| 1556 | PRKD3 | 8 | 1 | -2.07835982759364 | 1.50708140510153 |
| 1557 | CDC42EP3 | 5 | 1 | 3.86964584867589 | -0.378503008903754 |
| 1558 | CYP1B1-AS1 | 12 | 1 | 2.00548769514196 | 2.05559910911154 |
| 1559 | ATL2 | 16 | 1 | -3.26821731050379 | 2.64710749763082 |
| 1560 | HNRNPLL | 3 | 1 | -2.96007607896693 | 0.0792412318570493 |
| 1561 | GALM | 4 | 1 | -3.99485896547206 | -1.06637796682287 |
| 1562 | SRSF7 | 8 | 1 | -1.75956974943049 | 0.529055372653711 |
| 1563 | GEMIN6 | 19 | 1 | -3.89782379587062 | 0.7725857653005 |
| 1564 | DHX57 | 9 | 1 | 0.616469755574391 | 2.77833737510275 |
| 1565 | MORN2 | 13 | 1 | -1.05944798906214 | -2.66049705368449 |
| 1566 | SOS1 | 12 | 1 | 2.55927111189 | 3.01555289405416 |
| 1567 | MAP4K3 | 12 | 1 | 2.95388008634679 | 2.53604401725363 |
| 1568 | AC007388.1 | 5 | 1 | 4.11070896665685 | -0.685377403320563 |
| 1569 | TMEM178A | 5 | 1 | 4.42271257917516 | -0.81121601087023 |
| 1570 | THUMPD2 | 17 | 1 | 0.845175280972646 | -0.184093340458167 |
| 1571 | SLC8A1-AS1 | 1 | 1 | 2.57573915998571 | 0.580094949184167 |
| 1572 | SLC8A1 | 5 | 1 | 3.80364204923742 | -0.632565929712546 |
| 1573 | PKDCC | 14 | 1 | -6.47882197816737 | -0.928201782675278 |
| 1574 | EML4 | 2 | 1 | -0.888865933016612 | 2.33504690307211 |
| 1575 | COX7A2L | 13 | 1 | -1.44180749376185 | -2.32672618252207 |
| 1576 | MTA3 | 4 | 1 | -5.05913804490931 | -0.552260681213629 |
| 1577 | ZFP36L2 | 14 | 1 | -5.19492076356776 | 0.44414760249685 |
| 1578 | AC010883.1 | 4 | 1 | -4.8418233247555 | -0.693546950043929 |
| 1579 | THADA | 2 | 1 | -0.602385804251506 | 1.42128838676046 |
| 1580 | PLEKHH2 | 19 | 1 | -5.38349555452235 | 1.45126237052511 |
| 1581 | DYNC2LI1 | 13 | 1 | -2.30506633241542 | -1.96652756554056 |
| 1582 | LRPPRC | 2 | 1 | -0.850878045157268 | 2.19875849860738 |
| 1583 | PPM1B | 9 | 1 | 1.87261010686986 | 2.83476413863729 |
| 1584 | SLC3A1 | 1 | 1 | 1.99616791288488 | 1.30118002074789 |
| 1585 | PREPL | 1 | 1 | 4.17598272840612 | 1.1860367216451 |
| 1586 | CAMKMT | 7 | 1 | -3.19272850473292 | -3.53391848427225 |
| 1587 | LINC01833 | 7 | 1 | -3.57837317903407 | -2.78607819897105 |
| 1588 | SIX3-AS1 | 18 | 1 | 6.29919029752843 | -2.39144693237711 |
| 1589 | SIX3 | 7 | 1 | -3.46471904237635 | -2.92887125832011 |
| 1590 | AC012354.1 | 7 | 1 | -3.11615942438014 | -3.27631984573771 |
| 1591 | SRBD1 | 8 | 1 | -2.81943915803797 | 1.92117895263265 |
| 1592 | PRKCE | 2 | 1 | 0.96547974626653 | 1.66418637412618 |
| 1593 | RHOQ | 15 | 1 | 4.96857668439977 | 0.0718808926923155 |
| 1594 | PIGF | 13 | 1 | -2.06313334424861 | -2.14955435615946 |
| 1595 | CRIPT | 3 | 1 | -2.67987178285487 | -1.34366099459101 |
| 1596 | SOCS5 | 12 | 1 | 2.63487173597448 | 2.98146070617269 |
| 1597 | MCFD2 | 2 | 1 | -0.105962857798411 | 2.36143602508138 |
| 1598 | TTC7A | 1 | 1 | 3.75887037794225 | 1.96030654090475 |
| 1599 | CALM2 | 5 | 1 | 3.82150007764928 | -0.448544575990927 |
| 1600 | AC106869.1 | 10 | 1 | 4.07950808088414 | -1.38214702826906 |
| 1601 | EPCAM | 10 | 1 | 4.63715864698522 | -1.39593250972201 |
| 1602 | MSH2 | 1 | 1 | 3.65132309476964 | 0.811193839488733 |
| 1603 | KCNK12 | 18 | 1 | 5.49022699873082 | -2.00850914341379 |
| 1604 | MSH6 | 9 | 1 | 0.325599863930867 | 2.96111001151632 |
| 1605 | FBXO11 | 1 | 1 | 3.48743917028539 | 2.46448697227072 |
| 1606 | FOXN2 | 18 | 1 | 5.12607981245153 | -1.71401189190317 |
| 1607 | AC093635.1 | 4 | 1 | -3.89369963128932 | -0.0840905151980044 |

| 1608 | PPP1R21 | 15 | 1 | 4.64329983274572 | 0.17124106067251 |
| --- | --- | --- | --- | --- | --- |
| 1609 | STON1 | 14 | 1 | -5.4015738817013 | -0.150564595283759 |
| 1610 | NRXN1 | 5 | 1 | 4.27676655332677 | -0.624966009678137 |
| 1611 | ASB3 | 3 | 1 | -2.06536088903315 | -0.0589587531702639 |
| 1612 | CHAC2 | 16 | 1 | -4.415591463641 | 1.90654219764303 |
| 1613 | ERLEC1 | 2 | 1 | 0.414493039055989 | 1.28351081985067 |
| 1614 | PSME4 | 8 | 1 | -1.35841487367518 | 1.66471709388326 |
| 1615 | ACYP2 | 10 | 1 | 3.28896404783361 | -1.59548614841868 |
| 1616 | SPTBN1 | 15 | 1 | 4.72445036451452 | 0.453566328464258 |
| 1617 | EML6 | 9 | 1 | 1.3664901403629 | 2.12554420608114 |
| 1618 | RTN4 | 1 | 1 | 2.86998201887243 | 0.422175780711877 |
| 1619 | CLHC1 | 2 | 1 | -1.06509046275981 | 2.02554668563436 |
| 1620 | AC012358.1 | 1 | 1 | 1.29006685297124 | 1.41476859229635 |
| 1621 | RPS27A | 21 | 1 | -0.81140492875941 | -4.38349018913676 |
| 1622 | MTIF2 | 3 | 1 | -2.89730165918238 | -0.40651260239054 |
| 1623 | AC012358.3 | 12 | 1 | 2.63003160040014 | 1.97256030219625 |
| 1624 | CCDC88A | 12 | 1 | 3.13634873907201 | 2.5341926969869 |
| 1625 | CFAP36 | 13 | 1 | -1.163917884425 | -2.57352946621348 |
| 1626 | PPP4R3B | 2 | 1 | -0.0817505272186537 | 2.10655202048849 |
| 1627 | AC015982.1 | 4 | 1 | -3.6223363729275 | -0.602313413442862 |
| 1628 | PNPT1 | 3 | 1 | -2.58177588899501 | 0.48581613677572 |
| 1629 | EFEMP1 | 4 | 1 | -4.31212590654261 | -0.976985647501242 |
| 1630 | MIR217HG | 7 | 1 | -3.60623525102503 | -2.98706374985148 |
| 1631 | AC007743.1 | 15 | 1 | 5.59904934446447 | 0.424476043163049 |
| 1632 | CCDC85A | 15 | 1 | 5.59158255140416 | 0.43048121112417 |
| 1633 | VRK2 | 11 | 1 | -3.6592540593899 | -1.6525337539332 |
| 1634 | FANCL | 16 | 1 | -4.04096244295008 | 2.32953108924459 |
| 1635 | AC007250.1 | 4 | 1 | -4.62489198167689 | 0.379708663402307 |
| 1636 | LINC01122 | 15 | 1 | 5.07549978773229 | -0.337217315258277 |
| 1637 | BCL11A | 18 | 1 | 5.85713173429601 | -1.63652019125391 |
| 1638 | PAPOLG | 9 | 1 | 1.21355022470586 | 2.94252170699667 |
| 1639 | REL | 1 | 1 | 3.9666106847965 | 1.49739183562826 |
| 1640 | PUS10 | 3 | 1 | -3.29619239290126 | -1.00266464722325 |
| 1641 | PEX13 | 3 | 1 | -2.00098143537409 | 0.242888823924768 |
| 1642 | KIAA1841 | 5 | 1 | 3.16668511907689 | 0.34479297774862 |
| 1643 | C2orf74 | 6 | 1 | 2.60537220518224 | -2.2002758823054 |
| 1644 | USP34 | 1 | 1 | 3.33622099439733 | 0.943781272350061 |
| 1645 | AC016727.1 | 10 | 1 | 3.71083737890355 | -1.42399565798212 |
| 1646 | XPO1 | 8 | 1 | -2.43694160898097 | 1.33940853255819 |
| 1647 | FAM161A | 5 | 1 | 4.30766798536412 | 0.0481395282132505 |
| 1648 | CCT4 | 11 | 1 | -2.95873378236659 | -1.0837376706021 |
| 1649 | COMMD1 | 6 | 1 | 0.760725632115529 | -2.31045029980112 |
| 1650 | B3GNT2 | 7 | 1 | -3.635318026141 | -2.83837376457621 |
| 1651 | TMEM17 | 8 | 1 | -1.63952647169001 | 0.254948512492883 |
| 1652 | AC092155.1 | 7 | 1 | -2.88694070299037 | -3.90835033279825 |
| 1653 | EHBP1 | 1 | 1 | 4.34666039030187 | 1.23748125213217 |
| 1654 | AC009501.1 | 6 | 1 | 2.42938210050695 | -1.96346078735758 |
| 1655 | OTX1 | 4 | 1 | -4.24524758775599 | -1.24607236606051 |
| 1656 | WDPCP | 16 | 1 | -3.68615601976283 | 2.82804812568258 |
| 1657 | MDH1 | 6 | 1 | 1.46358312646978 | -1.29164762717654 |
| 1658 | UGP2 | 16 | 1 | -3.05760858972438 | 2.61035098212789 |
| 1659 | VPS54 | 2 | 1 | -0.90109066684611 | 2.08229078429769 |
| 1660 | AC012368.2 | 5 | 1 | 4.21500517408483 | -0.192951246322882 |
| 1661 | PELI1 | 2 | 1 | -0.807335958079173 | 1.97684611457418 |
| 1662 | AC012368.1 | 4 | 1 | -4.50174067933924 | -0.531103148283255 |
| 1663 | LGALSL | 15 | 1 | 4.90743805448644 | 0.232679263530481 |
| 1664 | AC008074.3 | 5 | 1 | 3.78099180738561 | -0.320114835323584 |
| 1665 | AFTPH | 12 | 1 | 3.21879436056249 | 2.68891634124349 |
| 1666 | SERTAD2 | 7 | 1 | -3.20563147981532 | -2.90317998749186 |
| 1667 | SLC1A4 | 1 | 1 | 4.71288563291662 | 0.877504006801355 |
| 1668 | CEP68 | 8 | 1 | -1.35647819955714 | 1.58307637351583 |
| 1669 | RAB1A | 5 | 1 | 4.17713429014318 | -0.633500441135657 |
| 1670 | ACTR2 | 12 | 1 | 2.59517313520543 | 2.74061860221456 |
| 1671 | SPRED2 | 1 | 1 | 3.05833508054845 | 1.69672693389486 |
| 1672 | LINC01873 | 1 | 1 | 2.81135632078283 | 1.49843420165609 |
| 1673 | MEIS1 | 1 | 1 | 4.931820645734 | 1.2249828733785 |
| 1674 | MEIS1-AS2 | 1 | 1 | 2.68810464422338 | 0.898680106578576 |

| 1675 | ETAA1 | 8 | 1 | -1.49575851877101 | 1.96794857162069 |
| --- | --- | --- | --- | --- | --- |
| 1676 | C1D | 6 | 1 | -0.119477227524592 | -0.847264646710646 |
| 1677 | PNO1 | 3 | 1 | -2.99186609704859 | -0.662244781078589 |
| 1678 | PPP3R1 | 1 | 1 | 3.66417337934606 | 0.781166807590234 |
| 1679 | AC017083.1 | 2 | 1 | 0.814782038136647 | 0.870789185939538 |
| 1680 | CNRIP1 | 5 | 1 | 4.45017696897619 | -0.469787015737784 |
| 1681 | FBXO48 | 16 | 1 | -3.40876482446559 | 2.54658092635702 |
| 1682 | APLF | 4 | 1 | -4.08936427553065 | 0.162848726688135 |
| 1683 | PROKR1 | 7 | 1 | -2.91451357324488 | -3.83354912620951 |
| 1684 | ARHGAP25 | 7 | 1 | -2.89028476198085 | -3.18088541847636 |
| 1685 | ANTXR1 | 11 | 1 | -3.6236004682339 | -1.66726259809901 |
| 1686 | GFPT1 | 1 | 1 | 3.94425226728551 | 0.775044337688196 |
| 1687 | NFU1 | 5 | 1 | 2.6167981771671 | -0.532361968578589 |
| 1688 | AAK1 | 5 | 1 | 3.43870498220556 | 0.195287481723535 |
| 1689 | GMCL1 | 8 | 1 | -1.5311726184643 | 1.78004993575643 |
| 1690 | SNRNP27 | 1 | 1 | 2.7731440214359 | 0.522361413417566 |
| 1691 | MXD1 | 1 | 1 | 3.52008844892614 | 0.975529447971094 |
| 1692 | PCBP1 | 13 | 1 | -0.825089201525523 | -0.909361398936999 |
| 1693 | LINC01816 | 11 | 1 | -3.81109140832789 | -1.43923054558207 |
| 1694 | TIA1 | 2 | 1 | -1.10366176088221 | 1.86753977912496 |
| 1695 | PCYOX1 | 1 | 1 | 3.31124116460912 | 0.836696163593042 |
| 1696 | SNRPG | 11 | 1 | -2.7482867093838 | -1.49369029623438 |
| 1697 | FAM136A | 3 | 1 | -2.90725062806971 | -0.69970190447737 |
| 1698 | AC022201.2 | 11 | 1 | -3.72990869005091 | -2.30272219997813 |
| 1699 | TGFA | 6 | 1 | 1.79950834791295 | -2.58033452850748 |
| 1700 | ADD2 | 1 | 1 | 3.94497515241735 | 1.28070987838339 |
| 1701 | VAX2 | 4 | 1 | -5.34964679201014 | -1.93159530979563 |
| 1702 | TEX261 | 8 | 1 | -0.776974186495616 | 0.637545482097375 |
| 1703 | NAGK | 5 | 1 | 4.16112591306798 | -0.759135051788581 |
| 1704 | MPHOSPH10 | 2 | 1 | 0.148219510718511 | 1.12487925666403 |
| 1705 | PAIP2B | 8 | 1 | -1.45226834733851 | 1.90013231414388 |
| 1706 | ZNF638 | 2 | 1 | -0.118351936534717 | 2.18994225638937 |
| 1707 | AC007878.1 | 9 | 1 | 0.912258758946584 | 2.59262289184164 |
| 1708 | CYP26B1 | 7 | 1 | -2.05853270013697 | -3.59557805878092 |
| 1709 | EXOC6B | 9 | 1 | 1.8552084116184 | 2.79393591064047 |
| 1710 | SPR | 14 | 1 | -5.4113395067013 | -0.81654666286875 |
| 1711 | EMX1 | 7 | 1 | -3.68903611619837 | -2.90442334038187 |
| 1712 | SFXN5 | 7 | 1 | -3.55807302911647 | -2.8887946448939 |
| 1713 | RAB11FIP5 | 11 | 1 | -3.94925759752162 | -2.21929000240732 |
| 1714 | PRADC1 | 6 | 1 | 2.59544135610692 | -2.10418413502146 |
| 1715 | CCT7 | 3 | 1 | -1.73913501222499 | -0.859878829838526 |
| 1716 | FBXO41 | 15 | 1 | 4.69789673368566 | 0.500308171687829 |
| 1717 | ALMS1 | 9 | 1 | -0.155080467656924 | 2.67854322570394 |
| 1718 | TPRKB | 11 | 1 | -2.92530606706507 | -1.12175570351053 |
| 1719 | STAMBP | 2 | 1 | -0.373172931567504 | 0.936175004420984 |
| 1720 | ACTG2 | 14 | 1 | -6.1309463830746 | 0.190675155101526 |
| 1721 | DGUOK | 13 | 1 | -1.08715747316249 | -1.8884124599116 |
| 1722 | TET3 | 1 | 1 | 3.01245070974462 | 1.81871594565939 |
| 1723 | BOLA3 | 6 | 1 | 1.57194651643865 | -1.20533637625147 |
| 1724 | BOLA3-AS1 | 10 | 1 | 3.98914267103307 | -1.11691304963995 |
| 1725 | MOB1A | 11 | 1 | -3.68166564424403 | -1.53041051250864 |
| 1726 | MTHFD2 | 14 | 1 | -5.45447133500941 | 0.00912518876622903 |
| 1727 | SLC4A5 | 12 | 1 | 2.5539024023258 | 2.31661786216329 |
| 1728 | DCTN1 | 1 | 1 | 3.33522941152685 | 0.805443064151513 |
| 1729 | DCTN1-AS1 | 15 | 1 | 5.62881638090245 | 0.509429232059228 |
| 1730 | WDR54 | 6 | 1 | 1.53439368288152 | -1.55483589989115 |
| 1731 | RTKN | 7 | 1 | -2.72200272996791 | -2.14466391426493 |
| 1732 | INO80B | 2 | 1 | -1.01673923452265 | 1.35898269790243 |
| 1733 | WBP1 | 11 | 1 | -3.11279390771754 | -2.05854235512186 |
| 1734 | MOGS | 8 | 1 | -1.47121094186671 | 0.754628912387597 |
| 1735 | MRPL53 | 13 | 1 | -0.610173717335536 | -2.45109854561259 |
| 1736 | CCDC142 | 12 | 1 | 2.67703558485143 | 2.66743387359213 |
| 1737 | TTC31 | 8 | 1 | -2.96823166330226 | 1.25285400527548 |
| 1738 | PCGF1 | 2 | 1 | -0.798533305720164 | 1.52535881179403 |
| 1739 | AUP1 | 13 | 1 | -0.672773167685344 | -1.19507606250216 |
| 1740 | LOXL3 | 4 | 1 | -4.70671533067591 | -1.39083628279139 |
| 1741 | DOK1 | 4 | 1 | -5.20693324525721 | -0.39938060861994 |

| 1742 | AC019069.1 | 11 | 1 | -3.79054330308802 | -2.21354101520945 |
| --- | --- | --- | --- | --- | --- |
| 1743 | HK2 | 7 | 1 | -3.19827650506861 | -3.58655558449198 |
| 1744 | POLE4 | 14 | 1 | -5.61512731035121 | 0.391454116283166 |
| 1745 | EVA1A | 14 | 1 | -5.93429730852015 | -0.364498539985907 |
| 1746 | MRPL19 | 3 | 1 | -3.43268869836695 | -0.841391108156932 |
| 1747 | GCFC2 | 4 | 1 | -3.61423800905116 | -0.159264489235175 |
| 1748 | AC005034.3 | 8 | 1 | -2.22004936654933 | 1.06457366126608 |
| 1749 | AC005034.4 | 11 | 1 | -3.72743247468836 | -0.911598233664227 |
| 1750 | LRRTM4 | 10 | 1 | 4.10466124097936 | -1.74897985083033 |
| 1751 | CTNNA2 | 5 | 1 | 4.5184004453861 | 0.480422035632837 |
| 1752 | LRRTM1 | 1 | 1 | 4.06044794599645 | 1.52042593139242 |
| 1753 | SUCLG1 | 10 | 1 | 3.16352082769506 | -1.89393816811014 |
| 1754 | DNAH6 | 2 | 1 | 0.179123922988103 | 1.3141387857778 |
| 1755 | TRABD2A | 7 | 1 | -3.47114251573451 | -3.73998938423563 |
| 1756 | TMSB10 | 10 | 1 | 3.40337945501439 | -1.93593893867899 |
| 1757 | KCMF1 | 2 | 1 | 0.0235760955058794 | 1.70251120704244 |
| 1758 | TCF7L1 | 4 | 1 | -5.01587651689418 | -0.171968086780799 |
| 1759 | TGOLN2 | 9 | 1 | -0.125762649074866 | 2.60408987182211 |
| 1760 | ELMOD3 | 1 | 1 | 4.02521063367955 | 1.62205113547872 |
| 1761 | MAT2A | 9 | 1 | -0.0916404130018492 | 3.08961500304769 |
| 1762 | GGCX | 8 | 1 | -2.04755352456935 | 1.61504019874166 |
| 1763 | RNF181 | 13 | 1 | -0.66565896590121 | -1.87615083080698 |
| 1764 | TMEM150A | 13 | 1 | -2.42210863550074 | -1.85302095037867 |
| 1765 | USP39 | 4 | 1 | -3.15982531030543 | -0.0768865190165163 |
| 1766 | C2orf68 | 2 | 1 | -0.736591145590617 | 2.1092192091329 |
| 1767 | ATOH8 | 16 | 1 | -4.16599105317958 | 3.04745020049642 |
| 1768 | ST3GAL5 | 15 | 1 | 4.39075066129796 | -0.149632676662696 |
| 1769 | POLR1A | 2 | 1 | -0.58463223297961 | 2.42793168204855 |
| 1770 | PTCD3 | 8 | 1 | -1.58227621038325 | 1.77891148704122 |
| 1771 | IMMT | 2 | 1 | -0.580828800991847 | 1.79770054000448 |
| 1772 | MRPL35 | 8 | 1 | -1.52392362077601 | 0.822798744617212 |
| 1773 | REEP1 | 15 | 1 | 4.9428970960819 | 0.479368940768945 |
| 1774 | KDM3A | 2 | 1 | -0.264994798422529 | 1.99924196380209 |
| 1775 | CHMP3 | 1 | 1 | 1.32265628854863 | 1.68748463767599 |
| 1776 | RNF103 | 1 | 1 | 3.16680885831945 | 2.30197633880209 |
| 1777 | RMND5A | 2 | 1 | -1.08756564576991 | 1.85113991874288 |
| 1778 | CD8A | 15 | 1 | 4.69440104047887 | 0.39058007377218 |
| 1779 | PLGLB1 | 12 | 1 | 1.95783473531835 | 2.56653083938192 |
| 1780 | CYTOR | 14 | 1 | -5.77059291322596 | 0.282514230190027 |
| 1781 | KRCC1 | 2 | 1 | 0.334853842660115 | 2.09529699462484 |
| 1782 | EIF2AK3 | 10 | 1 | 4.57982708494298 | -2.15465734821726 |
| 1783 | AC062029.1 | 16 | 1 | -4.32462190111048 | 2.23445428985189 |
| 1784 | RPIA | 3 | 1 | -3.20979641397364 | 0.465197221217859 |
| 1785 | MAL | 20 | 1 | 3.27375127355687 | -3.52574692589213 |
| 1786 | MRPS5 | 2 | 1 | 0.653513029023335 | 0.932945505557763 |
| 1787 | AC092835.1 | 9 | 1 | 1.03704740087621 | 2.7798050798757 |
| 1788 | FAHD2A | 3 | 1 | -3.01703380067713 | -0.895322031965298 |
| 1789 | LINC00342 | 1 | 1 | 3.70071364919774 | 1.91203107017111 |
| 1790 | ANKRD36C | 1 | 1 | 4.30410505811803 | 1.93238271850179 |
| 1791 | DUSP2 | 14 | 1 | -6.91503499467738 | -0.799031927408469 |
| 1792 | STARD7 | 9 | 1 | 1.92459751646154 | 2.8355432905538 |
| 1793 | TMEM127 | 1 | 1 | 3.14195395986669 | 1.91070450919698 |
| 1794 | CIAO1 | 8 | 1 | -1.0219318719662 | 0.298396245418298 |
| 1795 | SNRNP200 | 2 | 1 | -1.35045431573756 | 2.14567174094747 |
| 1796 | NCAPH | 16 | 1 | -4.64610885103114 | 3.02189697402548 |
| 1797 | ARID5A | 14 | 1 | -6.45670197923548 | -0.756654261769545 |
| 1798 | KANSL3 | 8 | 1 | -3.00014232118495 | 1.3936315931661 |
| 1799 | LMAN2L | 3 | 1 | -1.82530187089808 | -0.38615606647898 |
| 1800 | CNNM3 | 9 | 1 | -0.265919726536347 | 2.71845640319418 |
| 1801 | ANKRD39 | 10 | 1 | 3.23010303060643 | -1.78070889336039 |
| 1802 | SEMA4C | 12 | 1 | 2.69337011854284 | 2.63468612807821 |
| 1803 | FAHD2B | 11 | 1 | -3.86099503953822 | -1.18728421551157 |
| 1804 | ANKRD36 | 1 | 1 | 4.35503412763707 | 1.77250208037924 |
| 1805 | AC092683.1 | 1 | 1 | 3.27357722799413 | 2.42938055175375 |
| 1806 | ANKRD36B | 1 | 1 | 4.03270985166662 | 2.02506817954611 |
| 1807 | COX5B | 6 | 1 | 0.502425923749135 | -2.3334294400828 |
| 1808 | ACTR1B | 5 | 1 | 3.34658433477514 | -0.863468325855029 |

| 1809 | TMEM131 | 2 | 1 | -0.77638147790797 | 2.15443553107809 |
| --- | --- | --- | --- | --- | --- |
| 1810 | VWA3B | 14 | 1 | -6.5508305879391 | -1.06464502138068 |
| 1811 | AC092675.1 | 14 | 1 | -6.56349204500087 | -1.06677554470469 |
| 1812 | CNGA3 | 6 | 1 | 1.58348466436498 | -2.52404664379526 |
| 1813 | INPP4A | 1 | 1 | 4.11458422224157 | 1.72656096595358 |
| 1814 | COA5 | 4 | 1 | -3.4527122827328 | -0.50710390430857 |
| 1815 | UNC50 | 13 | 1 | -2.17608939130671 | -1.29564465505053 |
| 1816 | MGAT4A | 5 | 1 | 3.91413380186193 | -0.118529304088843 |
| 1817 | KIAA1211L | 17 | 1 | 2.14300443212621 | -0.430339737953436 |
| 1818 | TSGA10 | 5 | 1 | 3.46749235670202 | -0.334447904648078 |
| 1819 | C2orf15 | 5 | 1 | 3.50134970228307 | -0.370685025276435 |
| 1820 | LIPT1 | 4 | 1 | -4.06601022203334 | -0.0175943813936831 |
| 1821 | MITD1 | 3 | 1 | -2.30975697954066 | -0.782869636239302 |
| 1822 | MRPL30 | 2 | 1 | -0.462646395162417 | 2.00335969108175 |
| 1823 | TXNDC9 | 6 | 1 | 1.84731866399877 | -1.41156373840739 |
| 1824 | EIF5B | 8 | 1 | -1.86413691957362 | 0.820660129962671 |
| 1825 | REV1 | 1 | 1 | 4.43927361051671 | 1.12640036719869 |
| 1826 | AFF3 | 10 | 1 | 4.7255799917423 | -0.774276807131064 |
| 1827 | LONRF2 | 1 | 1 | 4.50472475568883 | 1.45822872298788 |
| 1828 | PDCL3 | 13 | 1 | -0.346827753082587 | -2.38040469509531 |
| 1829 | NPAS2 | 15 | 1 | 5.92263008634679 | 0.836244002757776 |
| 1830 | RPL31 | 21 | 1 | -1.11572425086863 | -4.67417608124186 |
| 1831 | TBC1D8-AS1 | 5 | 1 | 3.85165287534826 | -0.590163781465781 |
| 1832 | CNOT11 | 13 | 1 | -1.47472535093196 | -0.779054536642325 |
| 1833 | RNF149 | 2 | 1 | 0.42584009449117 | 2.25663890021871 |
| 1834 | CREG2 | 6 | 1 | 1.98800350706212 | -2.6889757953303 |
| 1835 | MAP4K4 | 12 | 1 | 1.52104128877752 | 1.97724475043844 |
| 1836 | MFSD9 | 2 | 1 | 0.17728297392957 | 2.1943124689443 |
| 1837 | LINC01965 | 14 | 1 | -6.30960296113856 | -0.28812949282099 |
| 1838 | LINC01102 | 20 | 1 | 3.76155354063146 | -3.37782297951151 |
| 1839 | LINC01114 | 14 | 1 | -6.5353915544308 | -1.0619605026858 |
| 1840 | POU3F3 | 1 | 1 | 3.09452821294896 | 2.16931761878561 |
| 1841 | AC018730.1 | 4 | 1 | -4.54038022477992 | -1.48591725450922 |
| 1842 | AC010884.1 | 11 | 1 | -4.09209559877284 | -1.89354936701227 |
| 1843 | MRPS9 | 4 | 1 | -3.26672576387294 | -0.256541176857245 |
| 1844 | LINC01918 | 11 | 1 | -3.94565366227992 | -1.62009452206064 |
| 1845 | AC012360.3 | 4 | 1 | -3.85838674028285 | -0.276704653324378 |
| 1846 | C2orf49 | 1 | 1 | 4.14839769880407 | 1.5390230573995 |
| 1847 | FHL2 | 13 | 1 | -1.43510292489894 | -2.82541750294138 |
| 1848 | NCK2 | 2 | 1 | 0.504636838837789 | 0.893763557849634 |
| 1849 | C2orf40 | 7 | 1 | -3.07365249116786 | -2.8724018178599 |
| 1850 | UXS1 | 16 | 1 | -3.56635640581019 | 1.83556856292318 |
| 1851 | ST6GAL2 | 9 | 1 | 1.08757950346105 | 2.20105017798971 |
| 1852 | SULT1C4 | 11 | 1 | -3.79569242914088 | -2.35470078808237 |
| 1853 | GCC2 | 2 | 1 | -0.152372792557551 | 2.19014825004171 |
| 1854 | LIMS1 | 19 | 1 | -3.88647292573817 | 0.491902486263025 |
| 1855 | RANBP2 | 9 | 1 | 0.144984230204747 | 2.57286538261007 |
| 1856 | CCDC138 | 16 | 1 | -4.18186734636195 | 2.13440002578329 |
| 1857 | SH3RF3 | 18 | 1 | 5.47138239423864 | -1.442036076607 |
| 1858 | Sep-10 | 4 | 1 | -4.2998049112118 | 0.503786579547632 |
| 1859 | SOWAHC | 2 | 1 | -0.189738009319379 | 2.3865469850881 |
| 1860 | RGPD5 | 1 | 1 | 2.23008848707311 | 0.987382546840417 |
| 1861 | NPHP1 | 3 | 1 | -2.49547408540614 | 0.595999614177453 |
| 1862 | SMIM37 | 13 | 1 | -0.194403585240914 | -2.20028863769938 |
| 1863 | BUB1 | 16 | 1 | -4.40590499360926 | 3.20233525413107 |
| 1864 | MIR4435-2HG | 14 | 1 | -5.88656638582118 | 0.210764304576623 |
| 1865 | BCL2L11 | 4 | 1 | -3.88168834169276 | 0.350004807887781 |
| 1866 | ANAPC1 | 8 | 1 | -1.46105884034999 | 1.96252478736471 |
| 1867 | MERTK | 4 | 1 | -5.0483834596432 | -0.457086130203498 |
| 1868 | FBLN7 | 19 | 1 | -4.53813193757899 | 1.79561533111166 |
| 1869 | ZC3H8 | 15 | 1 | 4.80864216367833 | 0.303954140125024 |
| 1870 | ZC3H6 | 5 | 1 | 4.4317248014652 | 0.48795236724447 |
| 1871 | TTL | 12 | 1 | 2.6671939043247 | 2.03226198333334 |
| 1872 | POLR1B | 2 | 1 | -0.985761746958568 | 1.93791212218832 |
| 1873 | CHCHD5 | 13 | 1 | -1.9436505885876 | -2.0899083457606 |
| 1874 | AC079922.2 | 5 | 1 | 2.3979113249027 | -0.539852216066611 |
| 1875 | SLC20A1 | 17 | 1 | 2.17498113195531 | -0.118354602875006 |

| 1876 | CKAP2L | 16 | 1 | -4.43229173143275 | 3.33392609733175 |
| --- | --- | --- | --- | --- | --- |
| 1877 | CBWD2 | 2 | 1 | 1.16714216749303 | 1.01279665607046 |
| 1878 | RABL2A | 12 | 1 | 1.92289377729528 | 2.08311332839559 |
| 1879 | SLC35F5 | 2 | 1 | -0.905401274756267 | 1.75923909324239 |
| 1880 | AC104653.1 | 7 | 1 | -3.11497019250758 | -2.75703702789713 |
| 1881 | AC110769.2 | 2 | 1 | -1.10230331142314 | 1.77151836532186 |
| 1882 | ACTR3 | 2 | 1 | 0.666632249756978 | 1.8647316851003 |
| 1883 | DPP10 | 15 | 1 | 5.62230373899572 | 0.746511594234216 |
| 1884 | DPP10-AS1 | 15 | 1 | 4.92752911131017 | 0.2298771299703 |
| 1885 | DDX18 | 2 | 1 | 0.509777083798573 | 0.837981716571557 |
| 1886 | CCDC93 | 12 | 1 | 3.22532226125829 | 2.68780697959493 |
| 1887 | INSIG2 | 1 | 1 | 3.9975674299442 | 1.42411770003866 |
| 1888 | EN1 | 14 | 1 | -6.5779073091305 | -1.05166745108534 |
| 1889 | C2orf76 | 13 | 1 | -2.06779192407496 | -2.05328391415049 |
| 1890 | DBI | 6 | 1 | 0.220727219983266 | -2.38927017074991 |
| 1891 | CFAP221 | 6 | 1 | 1.99255420248143 | -1.74672345739771 |
| 1892 | TMEM177 | 4 | 1 | -3.72183631379969 | -0.257401391090644 |
| 1893 | PTPN4 | 5 | 1 | 2.81380964796178 | -0.443774356664908 |
| 1894 | EPB41L5 | 3 | 1 | -2.43306086976893 | 0.8470646299703 |
| 1895 | AC012363.1 | 2 | 1 | -1.1556501241482 | 1.33458413261007 |
| 1896 | TMEM185B | 11 | 1 | -3.28874395807154 | -1.2113363586085 |
| 1897 | RALB | 1 | 1 | 3.08492590467565 | 1.00923289435934 |
| 1898 | INHBB | 4 | 1 | -5.30131123979457 | -2.18613885265757 |
| 1899 | GLI2 | 4 | 1 | -4.58075498063929 | -0.799736141385329 |
| 1900 | CLASP1 | 9 | 1 | 0.750929012700246 | 2.71505655425619 |
| 1901 | AC012447.1 | 8 | 1 | -1.20726744850047 | 0.72371782439779 |
| 1902 | NIFK-AS1 | 8 | 1 | -1.76618419607051 | 1.10942472594808 |
| 1903 | NIFK | 3 | 1 | -2.72292731721766 | -0.830825439812434 |
| 1904 | TSN | 8 | 1 | -1.12746225316889 | 0.662928954540002 |
| 1905 | CNTNAP5 | 10 | 1 | 3.98733927290075 | -1.81085126024653 |
| 1906 | GYPC | 4 | 1 | -4.74037621934779 | -2.11751256805826 |
| 1907 | AC012508.1 | 7 | 1 | -3.4397716375149 | -3.67441640716959 |
| 1908 | BIN1 | 5 | 1 | 3.09425355474584 | -0.597616030992758 |
| 1909 | CYP27C1 | 11 | 1 | -3.78555677850611 | -1.55038092953135 |
| 1910 | ERCC3 | 9 | 1 | 0.384067490502522 | 2.61399354117941 |
| 1911 | MAP3K2 | 12 | 1 | 2.42271353284948 | 2.58932031768392 |
| 1912 | IWS1 | 8 | 1 | -0.688345864609553 | 0.542036668239343 |
| 1913 | LIMS2 | 7 | 1 | -3.73590420205958 | -3.11478315216471 |
| 1914 | WDR33 | 2 | 1 | -0.625864133433177 | 0.846096769748437 |
| 1915 | SFT2D3 | 3 | 1 | -3.15437172372706 | -0.794924429775965 |
| 1916 | POLR2D | 16 | 1 | -3.39226387460597 | 1.89157880920004 |
| 1917 | AMMECR1L | 9 | 1 | 0.19976000468366 | 2.71852792876791 |
| 1918 | AC012306.2 | 17 | 1 | 0.371770158215688 | 0.608453647075403 |
| 1919 | SAP130 | 9 | 1 | 0.478842749997304 | 2.80514110702108 |
| 1920 | UGGT1 | 9 | 1 | 0.911179914876149 | 3.04695071357321 |
| 1921 | HS6ST1 | 8 | 1 | -1.30596266706355 | 0.442335859714258 |
| 1922 | LINC02572 | 4 | 1 | -4.37641452272303 | -1.0913724593537 |
| 1923 | CCDC74B | 6 | 1 | 2.36260367910497 | -1.45884053331782 |
| 1924 | SMPD4 | 16 | 1 | -3.20216272790797 | 2.5598606027944 |
| 1925 | MZT2B | 6 | 1 | 0.439323082371877 | -2.12134180885721 |
| 1926 | CCDC115 | 5 | 1 | 2.92867971937291 | -0.602281107725394 |
| 1927 | IMP4 | 3 | 1 | -2.73581527192958 | -0.868266928167832 |
| 1928 | PTPN18 | 19 | 1 | -4.44638036210902 | 1.56185640472006 |
| 1929 | POTEI | 1 | 1 | 3.18167020361059 | 1.44084014075826 |
| 1930 | PRSS40A | 1 | 1 | 2.41777969877355 | 0.652290240703332 |
| 1931 | AMER3 | 15 | 1 | 4.84524179975621 | -0.354971750797522 |
| 1932 | ARHGEF4 | 5 | 1 | 3.90970922987096 | -0.709697096766722 |
| 1933 | FAM168B | 8 | 1 | -1.4330475183285 | 1.60294045585226 |
| 1934 | PLEKHB2 | 2 | 1 | 1.28258348981969 | 1.13913907188009 |
| 1935 | MZT2A | 6 | 1 | 0.969377651616261 | -1.74990932566096 |
| 1936 | CCDC74A | 6 | 1 | 2.53871633092992 | -1.09238588732649 |
| 1937 | AC097532.2 | 15 | 1 | 4.66290070097081 | 0.474959150729883 |
| 1938 | GPR39 | 10 | 1 | 4.45806433241002 | -1.34610094052721 |
| 1939 | LYPD1 | 18 | 1 | 5.54533554594152 | -2.07474385124613 |
| 1940 | NCKAP5 | 14 | 1 | -5.81177400071986 | -1.29882992726733 |
| 1941 | MGAT5 | 18 | 1 | 5.39291836301915 | -0.739185839118254 |
| 1942 | TMEM163 | 6 | 1 | 2.50433589498632 | -1.81046752554346 |

| 1943 | CCNT2 | 2 | 1 | 0.580696299477742 | 2.23611368316244 |
| --- | --- | --- | --- | --- | --- |
| 1944 | RAB3GAP1 | 2 | 1 | 0.619841888352559 | 2.25106920379232 |
| 1945 | ZRANB3 | 19 | 1 | -3.88080428560145 | 1.40292824882101 |
| 1946 | R3HDM1 | 9 | 1 | 0.105693146392034 | 3.14376105445455 |
| 1947 | UBXN4 | 3 | 1 | -2.34201024492152 | -0.457835211576712 |
| 1948 | MCM6 | 19 | 1 | -5.27773021180995 | 0.779190675197351 |
| 1949 | DARS | 3 | 1 | -2.20355258901484 | 0.473899022517907 |
| 1950 | CXCR4 | 20 | 1 | 3.37173034231298 | -3.77505932671 |
| 1951 | THSD7B | 7 | 1 | -1.93930445630915 | -3.62947855812479 |
| 1952 | HNMT | 7 | 1 | -3.88730667550929 | -2.76871667725016 |
| 1953 | SPOPL | 9 | 1 | 0.427860393925832 | 2.61063541549276 |
| 1954 | NXPH2 | 19 | 1 | -5.63541339357264 | 1.29237546104025 |
| 1955 | LRP1B | 15 | 1 | 5.9797189382755 | 0.518414036212671 |
| 1956 | KYNU | 11 | 1 | -3.58566449602015 | -2.31187949997355 |
| 1957 | ARHGAP15 | 7 | 1 | -1.89616833646662 | -3.54896627289225 |
| 1958 | AC096558.2 | 7 | 1 | -2.6322355123318 | -3.8199549756663 |
| 1959 | GTDC1 | 2 | 1 | 0.842808738156484 | 1.82039202827047 |
| 1960 | ZEB2 | 4 | 1 | -4.42239974458583 | -2.51520298344065 |
| 1961 | ZEB2-AS1 | 4 | 1 | -5.27902816255458 | -2.10813532692362 |
| 1962 | ACVR2A | 9 | 1 | 0.947806969090627 | 2.37853469031881 |
| 1963 | ORC4 | 12 | 1 | 2.07689429800145 | 2.53236140388082 |
| 1964 | MBD5 | 12 | 1 | 2.24865175764196 | 2.75343598502706 |
| 1965 | EPC2 | 12 | 1 | 3.51619316618078 | 2.70418728965353 |
| 1966 | AC105402.3 | 1 | 1 | 2.47851254026525 | 0.948726669727075 |
| 1967 | KIF5C | 10 | 1 | 3.73872329275243 | -1.56454925161768 |
| 1968 | LYPD6 | 19 | 1 | -4.42647670228846 | 0.657169000087488 |
| 1969 | MMADHC | 3 | 1 | -2.41751025636561 | -0.815113670350802 |
| 1970 | RND3 | 20 | 1 | 3.40665556470983 | -3.68234024864603 |
| 1971 | RBM43 | 4 | 1 | -4.67610715349086 | -0.943426455529225 |
| 1972 | RIF1 | 2 | 1 | -0.569704905346705 | 2.66827477592062 |
| 1973 | ARL5A | 4 | 1 | -3.56465433557399 | 0.174107805667627 |
| 1974 | CACNB4 | 15 | 1 | 4.8171532301151 | 0.661803738055932 |
| 1975 | STAM2 | 1 | 1 | 3.25570918600194 | 2.25108517783712 |
| 1976 | FMNL2 | 2 | 1 | 0.138280585213826 | 1.92109550612997 |
| 1977 | PRPF40A | 3 | 1 | -2.17971931417353 | -0.105123146595252 |
| 1978 | ARL6IP6 | 11 | 1 | -2.99063013513453 | -1.36860234958102 |
| 1979 | RPRM | 11 | 1 | -3.48794625718959 | -1.05487941128184 |
| 1980 | GALNT13 | 15 | 1 | 5.39052750150792 | 1.23128689902853 |
| 1981 | AC009227.1 | 1 | 1 | 3.50097466985814 | 1.34037102836202 |
| 1982 | LINC01876 | 2 | 1 | -0.752359435156657 | 1.72267641204427 |
| 1983 | NR4A2 | 18 | 1 | 6.15613199751012 | -2.23282037598063 |
| 1984 | GPD2 | 19 | 1 | -4.62797044237025 | 1.56034721511434 |
| 1985 | ACVR1C | 7 | 1 | -2.80583594758876 | -3.50132141930033 |
| 1986 | CCDC148 | 15 | 1 | 5.3720266966068 | 0.925316587863672 |
| 1987 | PKP4 | 16 | 1 | -3.28629229982264 | 2.43317641395162 |
| 1988 | PKP4-AS1 | 1 | 1 | 3.01883794347875 | 1.81004823821615 |
| 1989 | DAPL1 | 11 | 1 | -3.82247446496852 | -1.45762132030894 |
| 1990 | TANC1 | 1 | 1 | 2.82995964567296 | 1.55493201392721 |
| 1991 | WDSUB1 | 6 | 1 | 2.33541347066991 | -1.16983015757967 |
| 1992 | BAZ2B | 2 | 1 | -0.00323608537085252 | 1.82771457809042 |
| 1993 | AC009506.1 | 3 | 1 | -3.36161755044825 | -0.965860947193396 |
| 1994 | Mar-07 | 2 | 1 | -1.02936516721614 | 1.3232289709432 |
| 1995 | CD302 | 9 | 1 | 1.02365352193944 | 2.24425949233602 |
| 1996 | PLA2R1 | 7 | 1 | -3.30149243791468 | -3.58547507149149 |
| 1997 | LINC02478 | 4 | 1 | -4.34314607103236 | -1.34300134998728 |
| 1998 | RBMS1 | 7 | 1 | -1.83374606092341 | -3.09590493065287 |
| 1999 | TANK | 1 | 1 | 2.58964802305333 | 0.386123792110193 |
| 2000 | PSMD14 | 5 | 1 | 4.24624086897008 | -0.58893145543505 |
| 2001 | TBR1 | 5 | 1 | 3.88392902891271 | -0.724448277773154 |
| 2002 | AC009487.1 | 10 | 1 | 3.08162547628515 | -1.3132371149676 |
| 2003 | SLC4A10 | 15 | 1 | 5.60784079115026 | 0.976442233501184 |
| 2004 | GCA | 11 | 1 | -3.12526343782313 | -1.94646046978403 |
| 2005 | KCNH7 | 5 | 1 | 3.07347060720556 | 0.161065951762903 |
| 2006 | AC011900.1 | 1 | 1 | 1.89793969671361 | 0.767759696422326 |
| 2007 | FIGN | 1 | 1 | 2.92165972272985 | 1.65214242118429 |
| 2008 | COBLL1 | 14 | 1 | -5.44861815889247 | 0.500341431079614 |
| 2009 | AC019197.1 | 1 | 1 | 3.33670450727575 | 1.11859716552328 |

| 2010 | SCN3A | 15 | 1 | 4.89966084043615 | 0.590581790385949 |
| --- | --- | --- | --- | --- | --- |
| 2011 | SCN2A | 15 | 1 | 5.11419321577184 | 0.583192721782434 |
| 2012 | CSRNP3 | 1 | 1 | 3.49517323057287 | 2.54221691268514 |
| 2013 | GALNT3 | 18 | 1 | 6.0363390592777 | -1.95332406383921 |
| 2014 | TTC21B | 2 | 1 | 0.153719201489613 | 2.1438495077474 |
| 2015 | AC010127.1 | 5 | 1 | 3.93997789899938 | -0.233513458790076 |
| 2016 | SCN9A | 5 | 1 | 4.09884621183507 | -0.441693916143668 |
| 2017 | B3GALT1 | 12 | 1 | 3.22794796506994 | 2.94086207526754 |
| 2018 | STK39 | 1 | 1 | 2.73578836004369 | 1.55889810699056 |
| 2019 | CERS6 | 1 | 1 | 4.20318390409581 | 1.37250937598776 |
| 2020 | NOSTRIN | 19 | 1 | -5.00534556825526 | 2.05418290275167 |
| 2021 | SPC25 | 16 | 1 | -4.84325908143885 | 2.4503406442983 |
| 2022 | LRP2 | 14 | 1 | -5.54835246522792 | -0.0312827072756411 |
| 2023 | BBS5 | 2 | 1 | -0.220390439227893 | 0.892460600314844 |
| 2024 | FASTKD1 | 9 | 1 | 1.73774947206609 | 2.89664377349447 |
| 2025 | PPIG | 8 | 1 | -2.11501322706111 | 2.10400404113363 |
| 2026 | CCDC173 | 4 | 1 | -4.05893300493129 | 0.214187637744653 |
| 2027 | PHOSPHO2 | 5 | 1 | 3.88083244840734 | 0.249835864482629 |
| 2028 | KLHL23 | 5 | 1 | 3.37935377637975 | -0.550439133466971 |
| 2029 | SSB | 3 | 1 | -2.3562083097256 | -0.324033661903632 |
| 2030 | METTL5 | 13 | 1 | -1.64360247571833 | -1.55490402800013 |
| 2031 | UBR3 | 1 | 1 | 3.02765060941808 | 2.26728691237997 |
| 2032 | SP5 | 14 | 1 | -5.58309720476039 | 0.139445678172815 |
| 2033 | AC007405.3 | 10 | 1 | 3.40232206861608 | -2.02087066990305 |
| 2034 | ERICH2 | 10 | 1 | 3.77352978269689 | -1.86048494202067 |
| 2035 | GAD1 | 18 | 1 | 6.20431877653234 | -2.36158488613535 |
| 2036 | GORASP2 | 8 | 1 | -1.18545751293071 | 0.640775100169885 |
| 2037 | TLK1 | 11 | 1 | -3.25556539018519 | -1.892644330086 |
| 2038 | METTL8 | 8 | 1 | -1.360615357951 | 1.73066081183981 |
| 2039 | CYBRD1 | 14 | 1 | -5.80524848420985 | 0.0425771631581663 |
| 2040 | DYNC1I2 | 5 | 1 | 2.89865709821813 | -0.493099763216269 |
| 2041 | SLC25A12 | 5 | 1 | 4.41215778867833 | 0.53244687694143 |
| 2042 | HAT1 | 19 | 1 | -4.57993100602992 | 0.760434047160852 |
| 2043 | METAP1D | 4 | 1 | -5.30151341874964 | -1.68464009386469 |
| 2044 | DLX1 | 18 | 1 | 6.20551754514806 | -2.67371497970988 |
| 2045 | DLX2 | 18 | 1 | 6.18633963148229 | -2.83840261322428 |
| 2046 | ITGA6 | 4 | 1 | -4.61359141786463 | -0.380043490947974 |
| 2047 | PDK1 | 5 | 1 | 3.832295670911 | 0.167308584628808 |
| 2048 | MAP3K20 | 4 | 1 | -4.82551883180506 | -0.182948931278479 |
| 2049 | CDCA7 | 14 | 1 | -6.03306935747035 | 0.186640755115259 |
| 2050 | SP3 | 9 | 1 | -0.273238374114706 | 2.57437743323873 |
| 2051 | OLA1 | 13 | 1 | -0.692102596358134 | -0.847325696768057 |
| 2052 | SP9 | 18 | 1 | 6.2888009695255 | -2.52536056858469 |
| 2053 | CIR1 | 17 | 1 | 0.326565936013387 | -0.385772749008429 |
| 2054 | SCRN3 | 8 | 1 | -1.48711751420863 | -0.2458508453982 |
| 2055 | GPR155 | 1 | 1 | 2.93981362859838 | 0.313346044002282 |
| 2056 | AC010894.2 | 11 | 1 | -3.38484595735438 | -1.36370892745425 |
| 2057 | WIPF1 | 4 | 1 | -3.94000385721095 | -0.179959698738349 |
| 2058 | CHRNA1 | 7 | 1 | -2.60533593614466 | -3.90910015922953 |
| 2059 | CHN1 | 5 | 1 | 4.22127319852941 | -0.58857710582186 |
| 2060 | ATF2 | 1 | 1 | 3.44180728475683 | 2.53921069282125 |
| 2061 | ATP5MC3 | 13 | 1 | -0.469056338266208 | -2.03958414417673 |
| 2062 | LNPK | 5 | 1 | 4.31261517088048 | 0.0588591255528806 |
| 2063 | MTX2 | 6 | 1 | 2.41400648634069 | -1.18661300760676 |
| 2064 | LINC01116 | 6 | 1 | 2.23479773084752 | -2.20607648712565 |
| 2065 | AC074286.1 | 6 | 1 | 2.22452379743688 | -1.92889963013102 |
| 2066 | HNRNPA3 | 3 | 1 | -2.6361667962826 | 0.725074545322168 |
| 2067 | NFE2L2 | 7 | 1 | -3.39763282258876 | -2.44971512180735 |
| 2068 | AGPS | 2 | 1 | -0.119507000044658 | 2.22522462981771 |
| 2069 | TTC30B | 2 | 1 | -0.230798581809475 | 0.888441816745508 |
| 2070 | TTC30A | 2 | 1 | -0.236262894429936 | 0.803014294086206 |
| 2071 | RBM45 | 13 | 1 | -0.671581343010737 | -0.972885511042369 |
| 2072 | OSBPL6 | 18 | 1 | 5.31004883329503 | -0.31190673929621 |
| 2073 | AC009948.1 | 8 | 1 | -1.3525153251446 | 1.79054392951559 |
| 2074 | PRKRA | 13 | 1 | -0.453536346749141 | -0.770765691222441 |
| 2075 | PJVK | 3 | 1 | -2.66308711488612 | 0.00621606486867654 |
| 2076 | FKBP7 | 3 | 1 | -2.44880960901149 | -0.764495073975814 |

| 2077 | PLEKHA3 | 2 | 1 | -0.453411832646205 | 0.875036970554101 |
| --- | --- | --- | --- | --- | --- |
| 2078 | TTN-AS1 | 9 | 1 | 1.18070842306249 | 2.93005384582113 |
| 2079 | TTN | 1 | 1 | 1.51834346334569 | 1.68182410377096 |
| 2080 | AC009948.3 | 2 | 1 | -0.631516888932063 | 2.04362596648763 |
| 2081 | AC010680.5 | 15 | 1 | 5.41673351804845 | 1.17823781150411 |
| 2082 | CCDC141 | 18 | 1 | 6.20300318281286 | -2.36534951549937 |
| 2083 | SESTD1 | 1 | 1 | 4.01359583418004 | 1.82108463424276 |
| 2084 | ZNF385B | 11 | 1 | -4.14122460801966 | -2.34078226906229 |
| 2085 | CWC22 | 8 | 1 | -1.959527716235 | 1.26517285483907 |
| 2086 | SCHLAP1 | 3 | 1 | -2.88827465494044 | 0.760547295985925 |
| 2087 | UBE2E3 | 5 | 1 | 4.22178532163732 | -0.698435201467764 |
| 2088 | AC104076.1 | 4 | 1 | -4.34706662614711 | -0.293551369728339 |
| 2089 | AC068196.1 | 1 | 1 | 3.19317533056371 | 1.44715155738424 |
| 2090 | LINC01934 | 1 | 1 | 2.93091393987768 | 1.90543569701742 |
| 2091 | ITGA4 | 15 | 1 | 5.84498573820226 | 0.930675522266137 |
| 2092 | CERKL | 7 | 1 | -2.2238554807461 | -3.65812931877543 |
| 2093 | NEUROD1 | 7 | 1 | -3.22726176698573 | -3.85842882019449 |
| 2094 | SSFA2 | 8 | 1 | -1.78417669256098 | 1.61424721854757 |
| 2095 | PDE1A | 15 | 1 | 5.65156723539464 | 0.825465218005884 |
| 2096 | DNAJC10 | 2 | 1 | -0.797888681486918 | 1.31370486396383 |
| 2097 | FRZB | 15 | 1 | 5.0668041853153 | -0.240706785740149 |
| 2098 | NCKAP1 | 12 | 1 | 2.63563419859044 | 2.38465274947714 |
| 2099 | NUP35 | 16 | 1 | -4.15243934114344 | 2.55880202430319 |
| 2100 | AC096667.1 | 5 | 1 | 4.28325583021276 | -0.768375008525145 |
| 2101 | ZNF804A | 15 | 1 | 5.12734819929235 | 0.709866897044885 |
| 2102 | FSIP2 | 9 | 1 | 1.7542014269077 | 2.80075754302572 |
| 2103 | LINC01473 | 3 | 1 | -2.6990558954037 | -0.0727813683169009 |
| 2104 | ZC3H15 | 3 | 1 | -2.31254623849757 | -0.000931664528143528 |
| 2105 | ITGAV | 14 | 1 | -6.53235600908168 | -0.418764843763602 |
| 2106 | FAM171B | 5 | 1 | 3.72344519178502 | -0.156776829780829 |
| 2107 | CALCRL | 4 | 1 | -4.94366715867884 | -0.71041226309706 |
| 2108 | TFPI | 14 | 1 | -5.06194280107386 | 0.282183662830102 |
| 2109 | GULP1 | 3 | 1 | -2.94534824808009 | 0.0859662451131223 |
| 2110 | COL3A1 | 14 | 1 | -6.61461947877772 | -0.300435229362738 |
| 2111 | COL5A2 | 4 | 1 | -4.51527522523768 | -0.867611847044718 |
| 2112 | WDR75 | 3 | 1 | -2.50074385126002 | 0.243981377063501 |
| 2113 | ASNSD1 | 17 | 1 | 1.00672818700902 | 0.227071420131433 |
| 2114 | OSGEPL1 | 8 | 1 | -2.15672491510279 | 1.08465232032369 |
| 2115 | AC013468.1 | 8 | 1 | -1.6019244046963 | 1.8664111055715 |
| 2116 | ORMDL1 | 3 | 1 | -2.37792585809596 | -0.0833728156702639 |
| 2117 | PMS1 | 2 | 1 | -0.665977284506633 | 1.46238245147299 |
| 2118 | C2orf88 | 8 | 1 | -2.16844366510279 | 1.47523583549093 |
| 2119 | HIBCH | 3 | 1 | -3.36345313508876 | -0.776836260380042 |
| 2120 | INPP1 | 15 | 1 | 5.36876894514196 | 0.227590218959558 |
| 2121 | MFSD6 | 1 | 1 | 4.21776558439367 | 0.722873464999902 |
| 2122 | NEMP2 | 19 | 1 | -4.85781978090174 | 1.62226046699117 |
| 2123 | NAB1 | 16 | 1 | -3.54130171258815 | 1.65153302329611 |
| 2124 | GLS | 9 | 1 | 1.4768052248203 | 2.36785830634664 |
| 2125 | STAT1 | 19 | 1 | -3.9736432882107 | 0.786736861644494 |
| 2126 | AC092614.1 | 4 | 1 | -3.50966261346705 | -0.684033542098296 |
| 2127 | MYO1B | 4 | 1 | -4.99099086244471 | -1.76693455797602 |
| 2128 | TMEFF2 | 18 | 1 | 5.97337891142003 | -2.13120148998667 |
| 2129 | SLC39A10 | 1 | 1 | 1.41215909998052 | 1.27364029067587 |
| 2130 | DNAH7 | 2 | 1 | 0.536311283513234 | 0.957850710330713 |
| 2131 | STK17B | 16 | 1 | -4.31896851976283 | 3.31097115653585 |
| 2132 | AC114760.2 | 16 | 1 | -4.37341807802088 | 3.35599221366476 |
| 2133 | HECW2 | 12 | 1 | 2.76027156393163 | 2.91112198012899 |
| 2134 | CCDC150 | 16 | 1 | -4.61503098924525 | 2.58405245917867 |
| 2135 | GTF3C3 | 3 | 1 | -2.60069535692103 | 0.0595198430402158 |
| 2136 | C2orf66 | 7 | 1 | -3.53063200433619 | -2.61691592556406 |
| 2137 | PGAP1 | 2 | 1 | 0.486775710984395 | 2.21290578025411 |
| 2138 | ANKRD44 | 1 | 1 | 4.65081287901037 | 1.29285945075582 |
| 2139 | SF3B1 | 2 | 1 | -0.40330240149863 | 1.27903713363241 |
| 2140 | HSPD1 | 3 | 1 | -3.20742796380885 | -0.942845705123437 |
| 2141 | HSPE1 | 11 | 1 | -2.73037861307032 | -1.42373864275385 |
| 2142 | MOB4 | 6 | 1 | -0.0723331274545927 | -0.6447247110026 |
| 2143 | RFTN2 | 7 | 1 | -3.41911600549586 | -2.57091341835428 |

| 2144 | PLCL1 | 5 | 1 | 3.69333197157018 | 0.106658355174768 |
| --- | --- | --- | --- | --- | --- |
| 2145 | SATB2 | 4 | 1 | -5.18635915239222 | -1.91341005188395 |
| 2146 | C2orf69 | 16 | 1 | -3.10450123269923 | 2.60083068984579 |
| 2147 | TYW5 | 9 | 1 | -0.506394520596339 | 2.96640504973959 |
| 2148 | MAIP1 | 13 | 1 | -1.36705790479548 | -0.815857588471663 |
| 2149 | SPATS2L | 14 | 1 | -6.39017079790004 | -0.857418264420521 |
| 2150 | KCTD18 | 2 | 1 | -1.04707519729502 | 0.908737079082239 |
| 2151 | SGO2 | 16 | 1 | -4.33208011110194 | 3.31246174949239 |
| 2152 | BZW1 | 8 | 1 | -2.12506829221614 | 1.32460631507467 |
| 2153 | CLK1 | 1 | 1 | 3.96453835050695 | 1.90854920524191 |
| 2154 | PPIL3 | 4 | 1 | -3.83288262803919 | 0.288248554645288 |
| 2155 | NIF3L1 | 16 | 1 | -3.98878143747218 | 2.38137997764181 |
| 2156 | ORC2 | 3 | 1 | -2.68348835428126 | 0.665743724284875 |
| 2157 | FAM126B | 1 | 1 | 3.25603343526952 | 0.648162023006189 |
| 2158 | NDUFB3 | 13 | 1 | -0.297814194307401 | -2.29641519409586 |
| 2159 | CFLAR | 6 | 1 | 0.877394214078114 | -2.95026193481852 |
| 2160 | TRAK2 | 12 | 1 | 1.53261555711858 | 2.10298408645223 |
| 2161 | STRADB | 6 | 1 | 1.4469858555042 | -1.29274584275652 |
| 2162 | TMEM237 | 16 | 1 | -4.54651759584315 | 2.17947115081381 |
| 2163 | ALS2 | 12 | 1 | 2.68971826116674 | 2.56703676360677 |
| 2164 | AC069148.1 | 14 | 1 | -6.18732522447474 | -0.364996119560492 |
| 2165 | FZD7 | 14 | 1 | -5.88292287309535 | -0.846008519800198 |
| 2166 | SUMO1 | 6 | 1 | -0.0306080581701537 | -2.29629002434183 |
| 2167 | NOP58 | 8 | 1 | -1.58449922521479 | 0.317586676059472 |
| 2168 | AC064836.3 | 15 | 1 | 5.26042677442662 | 0.0616189994199156 |
| 2169 | BMPR2 | 1 | 1 | 3.01946522276037 | 1.83199395316671 |
| 2170 | FAM117B | 1 | 1 | 3.93233658353917 | 2.29488910811971 |
| 2171 | ICA1L | 1 | 1 | 4.23165203611486 | 0.673118845401513 |
| 2172 | WDR12 | 3 | 1 | -2.63626526315577 | 0.321031347690332 |
| 2173 | CARF | 9 | 1 | 0.703257515832112 | 2.90881647246908 |
| 2174 | NBEAL1 | 8 | 1 | -1.86931549032099 | 0.575530067859399 |
| 2175 | CYP20A1 | 8 | 1 | -1.71585641820796 | 1.6985222257955 |
| 2176 | ABI2 | 12 | 1 | 2.85879756490819 | 2.92355670112203 |
| 2177 | RAPH1 | 1 | 1 | 3.92540384809606 | 1.81434883254598 |
| 2178 | PARD3B | 4 | 1 | -4.90261767824061 | -0.366138442577613 |
| 2179 | NRP2 | 5 | 1 | 3.50686599294774 | -0.674452289165747 |
| 2180 | INO80D | 12 | 1 | 2.96799375097387 | 2.71944846290182 |
| 2181 | NDUFS1 | 12 | 1 | 1.97634173910253 | 2.7400716699941 |
| 2182 | EEF1B2 | 11 | 1 | -2.93911670167811 | -1.81744103056361 |
| 2183 | ZDBF2 | 12 | 1 | 2.67775942365758 | 2.35740937369894 |
| 2184 | ADAM23 | 5 | 1 | 3.39228059332006 | 0.202574626384485 |
| 2185 | MDH1B | 13 | 1 | -1.42292343099482 | -2.67513905388285 |
| 2186 | FASTKD2 | 8 | 1 | -0.691878274277522 | 0.816661373553979 |
| 2187 | KLF7 | 1 | 1 | 3.20620204488866 | 1.68239559310507 |
| 2188 | CREB1 | 9 | 1 | 1.83072330038183 | 3.03894485610555 |
| 2189 | METTL21A | 4 | 1 | -4.52190421541102 | -0.27524571043421 |
| 2190 | CCNYL1 | 3 | 1 | -2.80581758935816 | 0.853008047519433 |
| 2191 | AC096772.1 | 4 | 1 | -4.63790105302699 | -0.509139075102103 |
| 2192 | FZD5 | 4 | 1 | -4.68568442781337 | -0.639616533340705 |
| 2193 | PLEKHM3 | 1 | 1 | 3.65753246824376 | 1.77637709754537 |
| 2194 | C2orf80 | 18 | 1 | 5.38239599744909 | -1.20031954270769 |
| 2195 | IDH1 | 5 | 1 | 3.24343229810827 | -0.029095812858832 |
| 2196 | IDH1-AS1 | 4 | 1 | -3.8979072423733 | -0.654619648279441 |
| 2197 | PIKFYVE | 9 | 1 | 0.604185893937276 | 2.93511332648824 |
| 2198 | MAP2 | 5 | 1 | 3.85625769178502 | 0.0857805170400022 |
| 2199 | UNC80 | 5 | 1 | 3.63161231557958 | -0.00789593082834494 |
| 2200 | RPE | 8 | 1 | -2.09786270578273 | 1.37004722732137 |
| 2201 | KANSL1L | 12 | 1 | 2.97747780363195 | 2.25406231063436 |
| 2202 | LANCL1 | 2 | 1 | 0.289331868096517 | 2.32100452560018 |
| 2203 | CPS1 | 11 | 1 | -3.54924128969081 | -2.2898697696345 |
| 2204 | ERBB4 | 14 | 1 | -6.68068192918666 | -0.558335050167334 |
| 2205 | IKZF2 | 9 | 1 | 1.57738520185582 | 2.85466112273763 |
| 2206 | AC079610.2 | 3 | 1 | -3.43905495126612 | 0.875004903255212 |
| 2207 | SPAG16 | 13 | 1 | -0.938158080176189 | -0.73313893300463 |
| 2208 | VWC2L | 18 | 1 | 5.57397343198888 | -2.13326619488169 |
| 2209 | BARD1 | 19 | 1 | -4.93327496965296 | 1.80954088347982 |
| 2210 | AC016708.1 | 16 | 1 | -4.44509194810755 | 2.21297134536337 |

| 2211 | ABCA12 | 19 | 1 | -4.99852393587001 | 1.79795064109396 |
| --- | --- | --- | --- | --- | --- |
| 2212 | ATIC | 3 | 1 | -2.59623239953883 | -0.971356949927104 |
| 2213 | FN1 | 11 | 1 | -4.00807450731166 | -1.36304213029314 |
| 2214 | MREG | 3 | 1 | -2.51456736047633 | 0.947929636417138 |
| 2215 | TMEM169 | 2 | 1 | 0.570701435013936 | 2.05051626342367 |
| 2216 | XRCC5 | 8 | 1 | -1.81145201642878 | 1.08615543025564 |
| 2217 | LINC01963 | 12 | 1 | 3.43828894178502 | 2.73002494948934 |
| 2218 | Mar-04 | 1 | 1 | 1.19663371126287 | 1.82153858321737 |
| 2219 | SMARCAL1 | 8 | 1 | -0.977592393950297 | 0.618042007861841 |
| 2220 | AC098820.3 | 4 | 1 | -5.27657006700404 | -1.78269933086802 |
| 2221 | RPL37A | 21 | 1 | -1.09519712646373 | -4.67712508064677 |
| 2222 | AC073321.1 | 7 | 1 | -3.50642488916285 | -2.81994305473734 |
| 2223 | IGFBP2 | 11 | 1 | -3.46712707002528 | -2.2028633199351 |
| 2224 | IGFBP5 | 4 | 1 | -4.83868955095179 | -0.707128106774581 |
| 2225 | AC007563.2 | 14 | 1 | -5.47202657182582 | -0.605381800951254 |
| 2226 | TNS1 | 18 | 1 | 5.99861742536657 | -1.92345724922587 |
| 2227 | AC010136.1 | 4 | 1 | -4.07772515733607 | -0.985763169051898 |
| 2228 | ARPC2 | 10 | 1 | 3.14480735342138 | -1.86981408220698 |
| 2229 | AAMP | 8 | 1 | -0.743438825205638 | -0.336922510685218 |
| 2230 | PNKD | 13 | 1 | -0.623937323883845 | -1.88010315519739 |
| 2231 | SLC11A1 | 7 | 1 | -3.54904149492152 | -3.29850183350016 |
| 2232 | CTDSP1 | 7 | 1 | -3.6983873697079 | -2.7378723464625 |
| 2233 | USP37 | 1 | 1 | 3.26990057508581 | 2.23631347793173 |
| 2234 | CNOT9 | 17 | 1 | 2.25052787344091 | -0.128081961693061 |
| 2235 | PLCD4 | 1 | 1 | 3.93966032545202 | 0.62914253848623 |
| 2236 | ZNF142 | 9 | 1 | 1.05613674203985 | 3.08982195037435 |
| 2237 | BCS1L | 10 | 1 | 3.25389434377782 | -1.67085258585383 |
| 2238 | STK36 | 2 | 1 | -0.764617249564006 | 1.1163140692098 |
| 2239 | TTLL4 | 8 | 1 | -1.71439968068965 | 1.5419253267629 |
| 2240 | CDK5R2 | 5 | 1 | 4.17384983579747 | -0.746853470029128 |
| 2241 | CFAP65 | 13 | 1 | -1.56212733705409 | -2.78339134079386 |
| 2242 | CNPPD1 | 10 | 1 | 3.15756084005468 | -1.85375635248591 |
| 2243 | RETREG2 | 5 | 1 | 3.12642838041417 | -0.297765298904669 |
| 2244 | ZFAND2B | 10 | 1 | 3.16790486852758 | -1.69197629314829 |
| 2245 | ANKZF1 | 5 | 1 | 3.13933421651952 | 0.215853468356836 |
| 2246 | STK16 | 13 | 1 | -0.719205334976985 | -0.834742508055461 |
| 2247 | TUBA4A | 7 | 1 | -2.21567474325068 | -3.67646203857828 |
| 2248 | DNAJB2 | 8 | 1 | -1.06765775401957 | 0.00315721410344827 |
| 2249 | PTPRN | 10 | 1 | 3.69490791837804 | -1.57941941839625 |
| 2250 | DNPEP | 14 | 1 | -5.50561355073817 | -0.738339587272895 |
| 2251 | AC053503.2 | 20 | 1 | 3.41607071439855 | -3.76396618706156 |
| 2252 | SPEG | 10 | 1 | 3.90475775282018 | -1.26287121993471 |
| 2253 | GMPPA | 13 | 1 | -0.62758614142306 | -1.77828149420191 |
| 2254 | ASIC4 | 6 | 1 | 2.18363763372533 | -2.72307549339701 |
| 2255 | TMEM198 | 13 | 1 | -1.37607239206202 | -1.3940165184157 |
| 2256 | OBSL1 | 6 | 1 | 2.6002285627567 | -1.71151415926386 |
| 2257 | STK11IP | 1 | 1 | 3.72694398443334 | 1.19740785735677 |
| 2258 | SLC4A3 | 15 | 1 | 4.40695120374791 | -0.106901987613928 |
| 2259 | EPHA4 | 2 | 1 | 0.259105220242665 | 1.96800722259115 |
| 2260 | PAX3 | 14 | 1 | -6.95306180436976 | -0.861464637489808 |
| 2261 | AC010980.2 | 5 | 1 | 4.23539568464391 | -0.247517987312567 |
| 2262 | FARSB | 17 | 1 | 1.18361426870458 | 0.174268022952783 |
| 2263 | ACSL3 | 5 | 1 | 3.18528701345556 | -0.122565432609809 |
| 2264 | SCG2 | 5 | 1 | 3.90270592252843 | -0.841738428147328 |
| 2265 | AP1S3 | 4 | 1 | -3.96157239396937 | -0.654692053021681 |
| 2266 | WDFY1 | 11 | 1 | -3.58312891443141 | -2.31302355152537 |
| 2267 | MRPL44 | 13 | 1 | -1.01325510461695 | -0.572457595886481 |
| 2268 | SERPINE2 | 8 | 1 | -1.0668228717602 | 0.0390030302388548 |
| 2269 | AC073052.2 | 1 | 1 | 2.09142233411901 | 1.0249837793691 |
| 2270 | CUL3 | 9 | 1 | 0.390714898511098 | 2.71683229583334 |
| 2271 | DOCK10 | 19 | 1 | -5.40983508546717 | 1.00075556415151 |
| 2272 | NYAP2 | 10 | 1 | 3.54071642439 | -1.36136372310091 |
| 2273 | AC016717.2 | 5 | 1 | 3.2181553987705 | 0.414555207668054 |
| 2274 | IRS1 | 18 | 1 | 6.24024464170568 | -2.41547070366312 |
| 2275 | RHBDD1 | 13 | 1 | -0.98577837665446 | -2.77412973267008 |
| 2276 | MFF | 8 | 1 | -1.14899878223307 | -0.0947931848185183 |
| 2277 | TM4SF20 | 1 | 1 | 1.61156238596074 | 1.53075923102926 |

| 2278 | AGFG1 | 2 | 1 | 0.207247838183568 | 2.08745493072103 |
| --- | --- | --- | --- | --- | --- |
| 2279 | DAW1 | 5 | 1 | 2.76436687986486 | -0.24685661417414 |
| 2280 | PID1 | 20 | 1 | 3.8013217596256 | -3.3488308034556 |
| 2281 | DNER | 10 | 1 | 3.39353062193029 | -1.69561193806101 |
| 2282 | TRIP12 | 2 | 1 | -0.70207448323138 | 1.77452386993002 |
| 2283 | SLC16A14 | 12 | 1 | 2.20870734731786 | 2.02701987403463 |
| 2284 | SP110 | 14 | 1 | -5.5141022058285 | -0.518534197630179 |
| 2285 | SP100 | 7 | 1 | -2.25534389932521 | -2.5370153031962 |
| 2286 | CAB39 | 1 | 1 | 3.66703368703954 | 2.17043604034017 |
| 2287 | ITM2C | 10 | 1 | 4.59170605222814 | -0.891047527240408 |
| 2288 | PSMD1 | 9 | 1 | 0.206390455170796 | 2.67597426551412 |
| 2289 | HTR2B | 1 | 1 | 1.56050278226964 | 1.3531087793691 |
| 2290 | ARMC9 | 2 | 1 | -0.670429453448131 | 1.13294853347372 |
| 2291 | NCL | 8 | 1 | -1.55881570299037 | 1.77564682143759 |
| 2292 | PTMA | 13 | 1 | -0.4048859478987 | -1.21631361466814 |
| 2293 | PDE6D | 10 | 1 | 3.12231232206456 | -1.67140380960871 |
| 2294 | COPS7B | 17 | 1 | 1.07629813234441 | -0.227931364597571 |
| 2295 | NPPC | 14 | 1 | -6.81082986314662 | -0.53988952857424 |
| 2296 | DIS3L2 | 9 | 1 | 1.91462708990209 | 2.95796693938802 |
| 2297 | ECEL1 | 18 | 1 | 6.31016470472448 | -2.47222839218546 |
| 2298 | PRSS56 | 7 | 1 | -2.77319072206385 | -3.70439754349161 |
| 2299 | EIF4E2 | 11 | 1 | -3.12859033067591 | -1.22087995630671 |
| 2300 | AC073254.1 | 1 | 1 | 1.97407056371801 | 1.5171998895986 |
| 2301 | EFHD1 | 19 | 1 | -5.58256839235194 | 1.28163661140035 |
| 2302 | GIGYF2 | 9 | 1 | 1.15433980505101 | 2.65208424705099 |
| 2303 | KCNJ13 | 8 | 1 | -2.56699894388087 | 1.52733077186178 |
| 2304 | INPP5D | 14 | 1 | -5.95125983674891 | 0.0995241560323118 |
| 2305 | ATG16L1 | 1 | 1 | 3.93369413892858 | 2.1742404856069 |
| 2306 | DGKD | 1 | 1 | 3.97717596571081 | 2.1798271097524 |
| 2307 | USP40 | 11 | 1 | -3.44906328637965 | -2.21550260406901 |
| 2308 | HJURP | 16 | 1 | -4.69962428529628 | 3.12964429038595 |
| 2309 | ARL4C | 1 | 1 | 4.82686831037633 | 1.09303344863485 |
| 2310 | SH3BP4 | 14 | 1 | -6.3428695054806 | -1.15816552859713 |
| 2311 | AGAP1 | 1 | 1 | 4.01382137815587 | 2.01145376342367 |
| 2312 | GBX2 | 14 | 1 | -6.88923715074427 | -0.79378029626776 |
| 2313 | ASB18 | 5 | 1 | 2.67625285665624 | -0.00564764362741721 |
| 2314 | AC093915.1 | 14 | 1 | -6.49562190492518 | -1.16328768354822 |
| 2315 | ACKR3 | 14 | 1 | -6.30125401933558 | -0.528257801355612 |
| 2316 | COPS8 | 13 | 1 | -0.579687729672267 | -0.759389340104354 |
| 2317 | LRRFIP1 | 12 | 1 | 2.5833144334995 | 2.43795813697408 |
| 2318 | RAMP1 | 7 | 1 | -2.84148524721034 | -3.54878030640055 |
| 2319 | UBE2F | 11 | 1 | -2.82384441812403 | -1.44368611198832 |
| 2320 | SCLY | 3 | 1 | -2.4578540178097 | -0.548897906364691 |
| 2321 | ILKAP | 13 | 1 | -1.33845971544154 | -0.612998171867621 |
| 2322 | HES6 | 7 | 1 | -3.6635858865536 | -2.93096052032877 |
| 2323 | TRAF3IP1 | 2 | 1 | -1.04023353774913 | 1.90419091361593 |
| 2324 | ASB1 | 3 | 1 | -2.72030447442897 | -0.683995812358153 |
| 2325 | TWIST2 | 7 | 1 | -3.48221252878077 | -3.6020742498057 |
| 2326 | HDAC4 | 12 | 1 | 1.78737212698095 | 2.72026266234945 |
| 2327 | AC062017.1 | 4 | 1 | -3.96093128641017 | -0.209933682502997 |
| 2328 | NDUFA10 | 3 | 1 | -2.13994096238978 | -1.11646337729861 |
| 2329 | COPS9 | 13 | 1 | -0.35914852489598 | -2.37915979248453 |
| 2330 | GPC1 | 2 | 1 | -0.0754401089704771 | 0.800216571269739 |
| 2331 | DUSP28 | 17 | 1 | 2.21191169302099 | -0.396911128582251 |
| 2332 | CAPN10-DT | 3 | 1 | -2.9271619172848 | -0.226861640037787 |
| 2333 | KIF1A | 5 | 1 | 3.78952051679723 | -0.868495776476157 |
| 2334 | AGXT | 6 | 1 | 2.0653484014713 | -2.59477446896006 |
| 2335 | SNED1 | 12 | 1 | 2.53958918134801 | 2.06567157882284 |
| 2336 | PASK | 5 | 1 | 2.86820842306249 | -0.130824192585242 |
| 2337 | PPP1R7 | 5 | 1 | 3.3669693617069 | -0.943280554534686 |
| 2338 | HDLBP | 2 | 1 | -1.20009075124629 | 2.14327730315756 |
| 2339 | Sep-02 | 4 | 1 | -3.55013297517665 | -0.279803796829474 |
| 2340 | FARP2 | 1 | 1 | 1.15109945814245 | 1.74620498794149 |
| 2341 | STK25 | 13 | 1 | -0.906475052431895 | -0.470159723581565 |
| 2342 | THAP4 | 6 | 1 | 0.507603123589681 | -1.77534679991175 |
| 2343 | ATG4B | 17 | 1 | 1.62773646394841 | 0.740960494456994 |
| 2344 | DTYMK | 16 | 1 | -4.57874034364588 | 2.5806969560964 |

| 2345 | ING5 | 4 | 1 | -4.30047057588465 | 0.191661492763269 |
| --- | --- | --- | --- | --- | --- |
| 2346 | D2HGDH | 2 | 1 | 0.00658817569844528 | 1.27611126082967 |
| 2347 | AC114730.2 | 1 | 1 | 2.26975275556676 | 1.07568146365713 |
| 2348 | CHL1 | 15 | 1 | 5.25695969144933 | 0.463548199115503 |
| 2349 | CNTN6 | 14 | 1 | -6.41907857377894 | -1.22770048600603 |
| 2350 | CNTN4 | 15 | 1 | 5.76141001264684 | 0.580519215045678 |
| 2351 | CNTN4-AS1 | 1 | 1 | 3.75528837720983 | 1.08679987567495 |
| 2352 | IL5RA | 1 | 1 | 4.13424326459996 | 0.750195399699914 |
| 2353 | TRNT1 | 3 | 1 | -2.6915092321194 | 0.532557979999292 |
| 2354 | CRBN | 2 | 1 | 1.11131836454503 | 0.858144775806177 |
| 2355 | SUMF1 | 8 | 1 | -2.25178860147364 | 0.871956244884241 |
| 2356 | LRRN1 | 11 | 1 | -3.54058311899073 | -2.09842990261484 |
| 2357 | SETMAR | 2 | 1 | -0.36260718126662 | 1.05185939448904 |
| 2358 | ITPR1 | 4 | 1 | -4.18332527597316 | -1.32811976891924 |
| 2359 | ARL8B | 12 | 1 | 2.46177650968664 | 2.66338671821188 |
| 2360 | GRM7 | 15 | 1 | 5.58076073209874 | 0.542663947520959 |
| 2361 | AC068313.1 | 15 | 1 | 5.50499416868322 | 0.42812205928396 |
| 2362 | LMCD1-AS1 | 1 | 1 | 2.81022883932226 | 1.46204985755514 |
| 2363 | LMCD1 | 14 | 1 | -6.60945342500575 | -0.29564480406214 |
| 2364 | OXTR | 14 | 1 | -6.27301953752406 | -0.409467443050635 |
| 2365 | RAD18 | 16 | 1 | -4.44982980211146 | 2.01774372237753 |
| 2366 | SRGAP3 | 2 | 1 | 0.941644802495168 | 1.42334093230795 |
| 2367 | SRGAP3-AS4 | 7 | 1 | -2.47882793863185 | -2.19879935604502 |
| 2368 | THUMPD3-AS1 | 5 | 1 | 3.38236190359227 | -1.01113788706232 |
| 2369 | THUMPD3 | 3 | 1 | -2.76956008394129 | 0.7659859814031 |
| 2370 | SETD5 | 12 | 1 | 2.48183204214208 | 2.00106825011801 |
| 2371 | LHFPL4 | 5 | 1 | 3.80590654890172 | -0.0213489375727297 |
| 2372 | MTMR14 | 8 | 1 | -1.39469157178767 | -0.127339883865607 |
| 2373 | CPNE9 | 6 | 1 | 1.37361218015783 | -2.85075674873759 |
| 2374 | BRPF1 | 2 | 1 | -0.388530314163043 | 2.15834201949667 |
| 2375 | OGG1 | 1 | 1 | 1.80405475179784 | 1.5877508081777 |
| 2376 | CAMK1 | 10 | 1 | 4.76374126951329 | -1.51622240168024 |
| 2377 | TADA3 | 3 | 1 | -1.77951453645594 | -0.802517458023322 |
| 2378 | ARPC4 | 13 | 1 | -1.03959570844538 | -0.793200477184546 |
| 2379 | TTLL3 | 7 | 1 | -2.52663062532313 | -3.13955961090494 |
| 2380 | RPUSD3 | 6 | 1 | -0.0121853353536864 | -0.993040680112136 |
| 2381 | JAGN1 | 13 | 1 | -2.26173900087245 | -1.69357202869822 |
| 2382 | IL17RE | 7 | 1 | -2.30424712617762 | -3.35933290344645 |
| 2383 | IL17RC | 7 | 1 | -1.91597424466975 | -3.6408356271403 |
| 2384 | CRELD1 | 6 | 1 | 1.85670901815526 | -1.22366841060091 |
| 2385 | PRRT3 | 1 | 1 | 3.01293160001867 | 0.898223415790307 |
| 2386 | AC018809.2 | 12 | 1 | 2.18315960447423 | 2.25400389808248 |
| 2387 | EMC3 | 6 | 1 | 2.02718640844457 | -1.19047696811129 |
| 2388 | AC022007.1 | 10 | 1 | 3.47781588117711 | -1.12582269233633 |
| 2389 | FANCD2 | 19 | 1 | -4.67822812517054 | 1.81206621306967 |
| 2390 | BRK1 | 10 | 1 | 3.85613943616979 | -1.58923278671671 |
| 2391 | VHL | 12 | 1 | 3.35381938497655 | 2.62948431151937 |
| 2392 | GHRL | 2 | 1 | -1.02550117690928 | 2.15095223563741 |
| 2393 | SEC13 | 13 | 1 | -1.17070446927912 | -0.554324015201819 |
| 2394 | ATP2B2 | 18 | 1 | 6.06582285444371 | -2.21067033630778 |
| 2395 | SLC6A1 | 15 | 1 | 5.49358011762731 | 0.604742423473108 |
| 2396 | ATG7 | 2 | 1 | -0.20863745008476 | 2.35683621543478 |
| 2397 | VGLL4 | 11 | 1 | -2.88318537195094 | -1.15041425746847 |
| 2398 | TAMM41 | 3 | 1 | -2.87291072328456 | -0.749477490009558 |
| 2399 | SYN2 | 18 | 1 | 5.4170854238712 | -0.387703224243415 |
| 2400 | TSEN2 | 2 | 1 | -0.448834091619327 | 1.74748076575826 |
| 2401 | MKRN2 | 3 | 1 | -1.95796667058833 | -0.139294906677497 |
| 2402 | RAF1 | 2 | 1 | -0.916113838747813 | 1.96423997062277 |
| 2403 | RPL32 | 21 | 1 | -0.823129877642467 | -4.3913022122996 |
| 2404 | IQSEC1 | 1 | 1 | 3.84619452039831 | 0.890702620921838 |
| 2405 | NUP210 | 19 | 1 | -4.75040839631922 | 1.62679351943563 |
| 2406 | WNT7A | 14 | 1 | -6.85141156633265 | -0.64082993489672 |
| 2407 | CHCHD4 | 17 | 1 | -0.0844991507089873 | -0.14937399250437 |
| 2408 | TMEM43 | 3 | 1 | -1.7335219236172 | 0.10025169032644 |
| 2409 | AC090004.1 | 8 | 1 | -0.818346247271373 | 0.0168067373616575 |
| 2410 | XPC | 2 | 1 | -0.847002133921458 | 2.01531995910238 |
| 2411 | LSM3 | 19 | 1 | -3.97343395669825 | 1.1924928583486 |

| 2412 | SLC6A6 | 1 | 1 | 3.24731327573888 | 2.32904566901754 |
| --- | --- | --- | --- | --- | --- |
| 2413 | FGD5-AS1 | 9 | 1 | -0.0811181666410704 | 3.07224620956014 |
| 2414 | NR2C2 | 12 | 1 | 2.28977395574682 | 3.08025302070211 |
| 2415 | MRPS25 | 8 | 1 | -1.23275671918757 | 0.188161984859216 |
| 2416 | RBSN | 12 | 1 | 1.96977235357396 | 2.772543565212 |
| 2417 | CAPN7 | 9 | 1 | 1.09264184515111 | 2.66320814269613 |
| 2418 | SH3BP5-AS1 | 1 | 1 | 3.07598187009923 | 1.33472742217611 |
| 2419 | SH3BP5 | 15 | 1 | 4.82189680616491 | -0.36616162878443 |
| 2420 | METTL6 | 1 | 1 | 4.06789375822179 | 1.72907961982321 |
| 2421 | EAF1 | 1 | 1 | 3.07077170889013 | 0.677502290187585 |
| 2422 | HACL1 | 3 | 1 | -2.00490497072108 | -0.0024869285242678 |
| 2423 | ANKRD28 | 9 | 1 | 1.95929695646398 | 3.11113824027609 |
| 2424 | DPH3 | 11 | 1 | -3.142717585162 | -1.43828897100855 |
| 2425 | OXNAD1 | 1 | 1 | 2.76407004873388 | 0.308377996860254 |
| 2426 | RFTN1 | 5 | 1 | 4.10450007001989 | -0.528712704004538 |
| 2427 | PLCL2 | 1 | 1 | 3.61653543989293 | 0.494499460635888 |
| 2428 | TBC1D5 | 1 | 1 | 3.25902177373998 | 1.52779664176534 |
| 2429 | SATB1 | 1 | 1 | 1.21606017629735 | 1.56172289031576 |
| 2430 | KCNH8 | 12 | 1 | 2.46143223325841 | 2.22057665961813 |
| 2431 | RAB5A | 2 | 1 | 0.27581043521993 | 1.61703361648153 |
| 2432 | KAT2B | 15 | 1 | 5.18277241270177 | 0.576489464221704 |
| 2433 | SGO1 | 16 | 1 | -4.57439969499476 | 3.15704764503072 |
| 2434 | ZNF385D | 7 | 1 | -3.09710739572413 | -3.84292326790262 |
| 2435 | UBE2E2 | 15 | 1 | 5.51513220350377 | 0.187536493716943 |
| 2436 | UBE2E1-AS1 | 11 | 1 | -3.65530703981288 | -2.41895864826609 |
| 2437 | UBE2E1 | 9 | 1 | 1.34598685781591 | 2.04170765060018 |
| 2438 | NKIRAS1 | 5 | 1 | 4.08143307249181 | 0.124981180606592 |
| 2439 | RPL15 | 21 | 1 | -1.10400472600825 | -4.52663670402933 |
| 2440 | NR1D2 | 2 | 1 | 1.03128673116796 | 1.67623271125387 |
| 2441 | THRB | 14 | 1 | -6.36264298875697 | -1.10770080906321 |
| 2442 | RARB | 12 | 1 | 2.29491879026525 | 2.16250695365499 |
| 2443 | TOP2B | 9 | 1 | 0.887840166493581 | 2.66084064620565 |
| 2444 | NGLY1 | 8 | 1 | -1.16838298757441 | 0.835835472522485 |
| 2445 | LRRC3B | 15 | 1 | 5.66036058942907 | 0.249764219699609 |
| 2446 | NEK10 | 1 | 1 | 4.02093340437047 | 0.86638106483053 |
| 2447 | SLC4A7 | 9 | 1 | 0.399775758191274 | 2.71431674140524 |
| 2448 | LINC02084 | 7 | 1 | -2.1523063035763 | -3.65685854774882 |
| 2449 | EOMES | 7 | 1 | -1.36292873342402 | -3.33231840950419 |
| 2450 | LINC01980 | 11 | 1 | -3.5599686952389 | -2.22532366138865 |
| 2451 | LINC01981 | 11 | 1 | -3.84488724191554 | -2.05338595730234 |
| 2452 | LINC01967 | 2 | 1 | -1.1082777829922 | 1.41383566039633 |
| 2453 | CMC1 | 7 | 1 | -2.13306354005702 | -2.9933894715922 |
| 2454 | AZI2 | 2 | 1 | 0.554842188759969 | 1.4143573202474 |
| 2455 | RBMS3 | 3 | 1 | -1.95649205167659 | -0.497050508083594 |
| 2456 | TGFBR2 | 7 | 1 | -3.76590918023951 | -3.25065980774332 |
| 2457 | STT3B | 16 | 1 | -3.1716322751797 | 2.15165914672445 |
| 2458 | CMTM8 | 14 | 1 | -4.99843715150721 | 0.0128904141766905 |
| 2459 | CMTM7 | 14 | 1 | -6.62243627031214 | -0.63356850963999 |
| 2460 | CMTM6 | 11 | 1 | -3.6707241388119 | -1.20623032671381 |
| 2461 | DYNC1LI1 | 5 | 1 | 3.18266631643407 | -0.829506679596198 |
| 2462 | CNOT10 | 8 | 1 | -1.65585576017268 | 1.74553097861837 |
| 2463 | TRIM71 | 12 | 1 | 2.51578809301488 | 1.70143403190206 |
| 2464 | GLB1 | 13 | 1 | -2.21337364633448 | -1.93126319271494 |
| 2465 | CRTAP | 11 | 1 | -3.35008691270716 | -2.00779710632731 |
| 2466 | SUSD5 | 14 | 1 | -5.68610356767543 | 0.0307025470120786 |
| 2467 | FBXL2 | 5 | 1 | 3.5032956747257 | 0.21072270053457 |
| 2468 | UBP1 | 9 | 1 | 1.34416522066228 | 2.9759069837911 |
| 2469 | CLASP2 | 5 | 1 | 4.35251213590734 | 0.462867633281457 |
| 2470 | PDCD6IP | 2 | 1 | -0.615127995804622 | 1.65871776717733 |
| 2471 | ARPP21 | 18 | 1 | 5.20938469450109 | -0.698372959317458 |
| 2472 | STAC | 4 | 1 | -4.12354777772792 | -0.82431147915293 |
| 2473 | DCLK3 | 18 | 1 | 5.64489389936559 | -0.576149239362967 |
| 2474 | TRANK1 | 1 | 1 | 4.6383431104862 | 0.833803430972803 |
| 2475 | EPM2AIP1 | 12 | 1 | 3.29962159673803 | 2.80790557044576 |
| 2476 | MLH1 | 8 | 1 | -1.26854275186427 | 0.4580584921224 |
| 2477 | LRRFIP2 | 5 | 1 | 3.7739927915775 | 0.226016417918908 |
| 2478 | AC126118.1 | 12 | 1 | 2.34152223150365 | 1.99063839095663 |

| 2479 | GOLGA4 | 2 | 1 | -0.252861262027844 | 2.23098124641012 |
| --- | --- | --- | --- | --- | --- |
| 2480 | CTDSPL | 19 | 1 | -4.37854884584315 | 1.38853396552633 |
| 2481 | PLCD1 | 7 | 1 | -3.47900508363612 | -2.41235850674082 |
| 2482 | DLEC1 | 7 | 1 | -3.19170211274989 | -3.78014336449076 |
| 2483 | ACAA1 | 3 | 1 | -1.60209201295741 | -1.12519322258402 |
| 2484 | MYD88 | 4 | 1 | -5.03693936784632 | -2.23454306942393 |
| 2485 | OXSR1 | 3 | 1 | -2.23753856141932 | 0.688431040225732 |
| 2486 | ACVR2B-AS1 | 11 | 1 | -3.92545364816554 | -1.81081370932032 |
| 2487 | ACVR2B | 12 | 1 | 2.40415979902379 | 2.5133575357778 |
| 2488 | EXOG | 5 | 1 | 4.55042292158239 | 0.629412905154931 |
| 2489 | SCN5A | 7 | 1 | -3.86982320268519 | -2.61196635586191 |
| 2490 | SCN11A | 2 | 1 | -1.14722000081904 | 1.88528837340902 |
| 2491 | WDR48 | 2 | 1 | 0.205653592749761 | 2.12380804198812 |
| 2492 | TTC21A | 15 | 1 | 4.77473713438146 | 0.464973823009241 |
| 2493 | CSRNP1 | 7 | 1 | -2.08688054998286 | -3.59056554657389 |
| 2494 | SLC25A38 | 3 | 1 | -1.93741630037196 | -1.22783709985186 |
| 2495 | RPSA | 21 | 1 | -1.15048907716639 | -4.24688444954325 |
| 2496 | MYRIP | 6 | 1 | 2.40231515447728 | -1.85246454101969 |
| 2497 | EIF1B | 10 | 1 | 4.11117674390905 | -1.30206774097849 |
| 2498 | RPL14 | 21 | 1 | -0.72260476668246 | -4.28701029640604 |
| 2499 | ZNF620 | 1 | 1 | 4.3214199690067 | 1.17395843642782 |
| 2500 | ZNF621 | 2 | 1 | -0.907945558623149 | 1.60290445464681 |
| 2501 | CTNNB1 | 6 | 1 | 2.09463430921666 | -1.99390493256022 |
| 2502 | ULK4 | 11 | 1 | -2.72600220163233 | -1.71403108459879 |
| 2503 | TRAK1 | 12 | 1 | 1.90033508817785 | 2.72437895911764 |
| 2504 | SEC22C | 8 | 1 | -1.27749739606745 | 1.34646143096517 |
| 2505 | SS18L2 | 6 | 1 | 1.10900534669988 | -1.94914577824045 |
| 2506 | NKTR | 9 | 1 | 0.967744126721547 | 2.70435823577474 |
| 2507 | LINC02158 | 11 | 1 | -4.03704713304408 | -2.17572222572733 |
| 2508 | HIGD1A | 11 | 1 | -2.90238021333583 | -1.50270591598917 |
| 2509 | POMGNT2 | 5 | 1 | 3.52581955473058 | -0.135028048576605 |
| 2510 | SNRK | 15 | 1 | 5.40064550916784 | 0.553206340251672 |
| 2511 | ANO10 | 6 | 1 | 1.51135422269933 | -2.14967499596049 |
| 2512 | ABHD5 | 1 | 1 | 3.69078208486669 | 2.53966702598165 |
| 2513 | AC006058.1 | 1 | 1 | 4.39124180357091 | 2.10777820724081 |
| 2514 | TCAIM | 2 | 1 | -0.170765035048797 | 1.54789699691366 |
| 2515 | ZNF445 | 1 | 1 | 3.29356218855016 | 2.10150684493612 |
| 2516 | ZNF852 | 12 | 1 | 2.56121708433263 | 2.7113121904714 |
| 2517 | ZKSCAN7 | 9 | 1 | 1.79744888822667 | 2.77775992530416 |
| 2518 | ZNF660 | 8 | 1 | -1.48282896001704 | 1.69271244185995 |
| 2519 | ZNF197 | 9 | 1 | 1.93117000143163 | 2.93929590362142 |
| 2520 | ZNF35 | 9 | 1 | 1.56288565675847 | 2.67603291648458 |
| 2521 | AC124045.1 | 12 | 1 | 2.01443411390416 | 2.77536667960714 |
| 2522 | ZNF501 | 2 | 1 | -0.474112615183665 | 1.27993930953573 |
| 2523 | KIAA1143 | 3 | 1 | -3.54137967546351 | 0.640340343890893 |
| 2524 | KIF15 | 16 | 1 | -4.60695289094813 | 2.89570559638571 |
| 2525 | TMEM42 | 17 | 1 | 0.19258944670789 | -0.081535085262549 |
| 2526 | ZDHHC3 | 2 | 1 | 0.229697450801061 | 1.35111107009481 |
| 2527 | EXOSC7 | 3 | 1 | -2.27494452913172 | -0.486297025503409 |
| 2528 | TMEM158 | 7 | 1 | -1.78036509473689 | -2.9257305703776 |
| 2529 | LARS2 | 3 | 1 | -2.29844997842677 | 0.949868575511682 |
| 2530 | LIMD1 | 11 | 1 | -3.78153179605372 | -0.817071571172965 |
| 2531 | SACM1L | 8 | 1 | -1.29857324083216 | 1.70913280623983 |
| 2532 | LZTFL1 | 3 | 1 | -2.56554316003688 | 0.550640360294092 |
| 2533 | FYCO1 | 7 | 1 | -3.67960809190638 | -2.68970428329874 |
| 2534 | ALS2CL | 7 | 1 | -2.82648633439906 | -3.84665022713114 |
| 2535 | TMIE | 7 | 1 | -2.80767463167079 | -3.89194522720743 |
| 2536 | MYL3 | 7 | 1 | -2.64247034509547 | -3.88336001259257 |
| 2537 | PTH1R | 7 | 1 | -2.92665432412989 | -3.96478997093607 |
| 2538 | CCDC12 | 13 | 1 | -0.927886173323466 | -0.755362196983588 |
| 2539 | SETD2 | 9 | 1 | 1.29033245126836 | 3.02560748237203 |
| 2540 | KIF9 | 13 | 1 | -0.831360981062724 | -2.72069595676829 |
| 2541 | AC099778.1 | 2 | 1 | 0.76226450483434 | 0.969479099689233 |
| 2542 | PTPN23 | 1 | 1 | 4.12719036619298 | 0.901553288875329 |
| 2543 | SCAP | 2 | 1 | 0.539925589963124 | 0.791775242267358 |
| 2544 | ELP6 | 19 | 1 | -3.95186422784693 | 1.02868856566976 |
| 2545 | CSPG5 | 14 | 1 | -5.78069041688807 | -0.224572106422675 |

| 2546 | SMARCC1 | 3 | 1 | -2.1778464170254 | 0.714937106548059 |
| --- | --- | --- | --- | --- | --- |
| 2547 | DHX30 | 4 | 1 | -3.4379737230099 | -0.392651125015509 |
| 2548 | MAP4 | 1 | 1 | 3.99974514524572 | 0.877448097644555 |
| 2549 | CDC25A | 16 | 1 | -3.972640499667 | 2.07029046195577 |
| 2550 | NME6 | 8 | 1 | -0.988162085608317 | 0.759797111926782 |
| 2551 | PLXNB1 | 2 | 1 | -0.853840455607249 | 1.80950202125143 |
| 2552 | TMA7 | 13 | 1 | -0.455704659417941 | -2.38032005649973 |
| 2553 | ATRIP | 2 | 1 | -0.998068198756053 | 2.7880259909017 |
| 2554 | SHISA5 | 5 | 1 | 2.7551050333225 | -0.546807303251517 |
| 2555 | PFKFB4 | 5 | 1 | 3.33506466428869 | -0.263274713577521 |
| 2556 | UQCRC1 | 13 | 1 | -0.834115073279216 | -1.84460286718775 |
| 2557 | CELSR3 | 5 | 1 | 3.27334262411229 | -0.283596380771887 |
| 2558 | NCKIPSD | 5 | 1 | 3.37313272039525 | -0.157080932678473 |
| 2559 | IP6K2 | 5 | 1 | 3.20399738828771 | 0.0335155882222532 |
| 2560 | PRKAR2A | 5 | 1 | 3.76778008024328 | -0.763500928105605 |
| 2561 | ARIH2 | 2 | 1 | -0.0342484714544554 | 2.3473726667745 |
| 2562 | P4HTM | 10 | 1 | 3.56423331777685 | -1.81565074545313 |
| 2563 | WDR6 | 1 | 1 | 3.49619676153295 | 2.29437770026754 |
| 2564 | DALRD3 | 6 | 1 | 1.29365827123754 | -2.04638491493632 |
| 2565 | NDUFAF3 | 13 | 1 | -0.401078671411349 | -2.359705313267 |
| 2566 | IMPDH2 | 3 | 1 | -2.09206305463679 | -1.01112328392435 |
| 2567 | QRICH1 | 9 | 1 | 1.38327063600652 | 2.99310316222738 |
| 2568 | QARS | 8 | 1 | -0.775063201979472 | -0.178590758861792 |
| 2569 | USP19 | 2 | 1 | 0.616384580537007 | 1.93120016234945 |
| 2570 | LAMB2 | 11 | 1 | -3.92828439195521 | -1.54504142624308 |
| 2571 | CCDC71 | 13 | 1 | -1.08617888410456 | -0.786709851743472 |
| 2572 | KLHDC8B | 13 | 1 | -0.818996235922648 | -1.32623086792399 |
| 2573 | C3orf62 | 1 | 1 | 2.72479774038427 | 1.52487458365988 |
| 2574 | USP4 | 9 | 1 | 1.51813103239171 | 2.55090059417318 |
| 2575 | RHOA | 11 | 1 | -2.64272616823085 | -1.59151099544932 |
| 2576 | TCTA | 5 | 1 | 4.03959394971959 | -0.76851972860266 |
| 2577 | DAG1 | 2 | 1 | -0.0579865129745741 | 2.28689088004659 |
| 2578 | BSN | 1 | 1 | 3.53522588293187 | 1.94213904517721 |
| 2579 | APEH | 17 | 1 | 0.207416817351506 | -0.163730546058905 |
| 2580 | GMPPB | 16 | 1 | -3.44117783029444 | 2.42723263877462 |
| 2581 | IP6K1 | 12 | 1 | 2.73834921400182 | 2.68316687720846 |
| 2582 | INKA1 | 18 | 1 | 5.75679184476964 | -2.03923962932993 |
| 2583 | TRAIP | 16 | 1 | -4.41848109681971 | 2.99510587829183 |
| 2584 | CAMKV | 18 | 1 | 6.30267120878331 | -2.33886001926829 |
| 2585 | RBM6 | 12 | 1 | 2.38228608648412 | 2.6288348593099 |
| 2586 | RBM5 | 9 | 1 | 2.01920081655614 | 3.02463974135946 |
| 2587 | RBM5-AS1 | 12 | 1 | 1.91169120351903 | 1.56219424384664 |
| 2588 | SEMA3F | 15 | 1 | 4.8667252210819 | -0.375871881069434 |
| 2589 | GNAI2 | 13 | 1 | -1.01625774820216 | -1.43149082285334 |
| 2590 | IFRD2 | 3 | 1 | -2.50365947206385 | -0.337459846557868 |
| 2591 | NAA80 | 10 | 1 | 3.20634032766454 | -1.40679453236033 |
| 2592 | HYAL2 | 3 | 1 | -1.98071406801112 | -0.892839725287449 |
| 2593 | TUSC2 | 6 | 1 | -0.0678323956287642 | -0.439020260395301 |
| 2594 | RASSF1 | 5 | 1 | 4.31410624067418 | -0.210188492359412 |
| 2595 | ZMYND10 | 13 | 1 | -0.979873523310496 | -2.70822201591898 |
| 2596 | NPRL2 | 10 | 1 | 4.59506775419347 | -0.802263385774386 |
| 2597 | CYB561D2 | 3 | 1 | -2.09356580694087 | -1.04557350200583 |
| 2598 | TMEM115 | 8 | 1 | -0.687448904112651 | -0.219820602955115 |
| 2599 | CACNA2D2 | 18 | 1 | 5.66436697523229 | -0.866857498111022 |
| 2600 | HEMK1 | 3 | 1 | -2.14574287851222 | 0.168953195987451 |
| 2601 | CISH | 7 | 1 | -3.5934798570431 | -2.80520294529368 |
| 2602 | MAPKAPK3 | 11 | 1 | -3.76707004030116 | -1.70008580309321 |
| 2603 | DOCK3 | 1 | 1 | 4.28072907011144 | 0.81641437190603 |
| 2604 | MANF | 3 | 1 | -2.79160045106776 | -1.10165513974596 |
| 2605 | RBM15B | 8 | 1 | -1.1718599053181 | -0.043736024917853 |
| 2606 | DCAF1 | 2 | 1 | -0.520946160153224 | 2.47596420425009 |
| 2607 | RAD54L2 | 9 | 1 | 1.55461384336583 | 2.90824832099508 |
| 2608 | TEX264 | 6 | 1 | 1.87551905195348 | -1.49695078951288 |
| 2609 | GRM2 | 6 | 1 | 2.51743198911779 | -2.35211931091715 |
| 2610 | RRP9 | 3 | 1 | -2.67826698739894 | -1.07097998184134 |
| 2611 | PCBP4 | 7 | 1 | -2.22432254274256 | -3.19476066452433 |
| 2612 | ABHD14B | 11 | 1 | -4.3083727212704 | -1.9196647248881 |

| 2613 | ABHD14A | 6 | 1 | 2.67432047407262 | -1.69318030697275 |
| --- | --- | --- | --- | --- | --- |
| 2614 | RPL29 | 21 | 1 | -1.02760724742778 | -4.43264923912455 |
| 2615 | DUSP7 | 1 | 1 | 3.32149745504491 | 0.537031904636133 |
| 2616 | POC1A | 16 | 1 | -4.6459028573788 | 2.44133319037985 |
| 2617 | ALAS1 | 2 | 1 | 1.12294866125219 | 1.24519266265463 |
| 2618 | TWF2 | 13 | 1 | -1.8894456477917 | -1.70266900879313 |
| 2619 | WDR82 | 12 | 1 | 2.40114952604406 | 2.71442617553304 |
| 2620 | PHF7 | 16 | 1 | -2.89287756402857 | 2.45910014289449 |
| 2621 | TNNC1 | 7 | 1 | -3.21649168451197 | -2.57753907066752 |
| 2622 | NISCH | 1 | 1 | 3.32716108839147 | 1.33817567008566 |
| 2623 | NT5DC2 | 19 | 1 | -5.26574824769862 | 0.902599588809717 |
| 2624 | SMIM4 | 13 | 1 | -0.903145000532939 | -2.14429138523508 |
| 2625 | PBRM1 | 12 | 1 | 2.72061969320409 | 2.65947975295614 |
| 2626 | GNL3 | 3 | 1 | -2.33959768731959 | -0.0264841161387087 |
| 2627 | GLT8D1 | 3 | 1 | -1.85975109060176 | -0.592133714975608 |
| 2628 | SPCS1 | 13 | 1 | -0.441836968258693 | -2.03688453060557 |
| 2629 | NEK4 | 8 | 1 | -1.79427574594386 | 1.42200960296224 |
| 2630 | SFMBT1 | 16 | 1 | -4.01474259813197 | 2.09899415153097 |
| 2631 | RFT1 | 4 | 1 | -3.64683578927882 | -0.000472649158728244 |
| 2632 | TKT | 3 | 1 | -1.97916804273493 | -1.08260020536352 |
| 2633 | DCP1A | 2 | 1 | 0.154258772536443 | 1.15426482337545 |
| 2634 | CACNA1D | 1 | 1 | 4.16727234403722 | 0.929709569392907 |
| 2635 | CHDH | 1 | 1 | 4.28461815397374 | 0.705973283229577 |
| 2636 | ACTR8 | 2 | 1 | 0.693963780804799 | 2.28488101142477 |
| 2637 | SELENOK | 13 | 1 | -0.208734281555488 | -2.40949331146647 |
| 2638 | WNT5A | 14 | 1 | -6.62107251603968 | -0.321625872673285 |
| 2639 | WNT5A-AS1 | 14 | 1 | -6.58492206056483 | -0.328103824676764 |
| 2640 | ERC2 | 1 | 1 | 4.58881165067785 | 0.679739252506006 |
| 2641 | CCDC66 | 1 | 1 | 1.48067308942907 | 1.125983849941 |
| 2642 | FAM208A | 9 | 1 | 1.31813634912603 | 3.07413877624105 |
| 2643 | ARHGEF3 | 1 | 1 | 3.60900641958349 | 1.61481227058004 |
| 2644 | IL17RD | 4 | 1 | -4.14302299936183 | -0.272567852558386 |
| 2645 | HESX1 | 4 | 1 | -4.39527677019007 | 0.157645837245691 |
| 2646 | APPL1 | 8 | 1 | -2.37244056184657 | 2.12267960685323 |
| 2647 | DNAH12 | 6 | 1 | 1.30636943857305 | -2.05153189522196 |
| 2648 | PDE12 | 9 | 1 | 1.39197494070165 | 3.00960172790121 |
| 2649 | ARF4 | 13 | 1 | -0.817023918227031 | -1.41240660768915 |
| 2650 | ARF4-AS1 | 4 | 1 | -3.90745376069911 | -0.5577325068133 |
| 2651 | DENND6A | 2 | 1 | -0.977117285326793 | 2.25411905425619 |
| 2652 | SLMAP | 9 | 1 | 0.270526304646657 | 2.78401841300558 |
| 2653 | FLNB | 4 | 1 | -4.05774401147731 | -0.164922758163703 |
| 2654 | ABHD6 | 1 | 1 | 3.19054700414769 | 1.6583324827535 |
| 2655 | RPP14 | 9 | 1 | -0.198149088964536 | 2.48481001037191 |
| 2656 | PXK | 15 | 1 | 4.64783360044591 | -0.223148687900794 |
| 2657 | PDHB | 6 | 1 | 1.85376049558751 | -1.37547369224001 |
| 2658 | KCTD6 | 5 | 1 | 3.78121639768712 | -0.661795227946532 |
| 2659 | C3orf67 | 2 | 1 | -0.918447479800059 | 2.24891986030172 |
| 2660 | FHIT | 7 | 1 | -2.68372915704615 | -3.33110247475077 |
| 2661 | PTPRG | 7 | 1 | -2.30132387597926 | -2.89154361111094 |
| 2662 | C3orf14 | 6 | 1 | 1.32575203458898 | -2.1669027648585 |
| 2663 | FEZF2 | 11 | 1 | -4.07594703157313 | -2.08350454193522 |
| 2664 | CADPS | 1 | 1 | 2.0655925420963 | 1.378181115566 |
| 2665 | THOC7 | 6 | 1 | 0.115388199492619 | -2.31211100441386 |
| 2666 | PSMD6 | 13 | 1 | -0.830877408579661 | -1.30883715969492 |
| 2667 | AC092040.1 | 20 | 1 | 3.71454884092443 | -3.43602739197184 |
| 2668 | PRICKLE2 | 20 | 1 | 3.70913435499303 | -3.43971286636759 |
| 2669 | ADAMTS9 | 11 | 1 | -3.98248432596095 | -1.93542300087382 |
| 2670 | ADAMTS9-AS2 | 11 | 1 | -4.14826487024195 | -2.10549686771799 |
| 2671 | MAGI1 | 9 | 1 | 0.839129343434499 | 2.73020090240072 |
| 2672 | SLC25A26 | 3 | 1 | -2.37806175668605 | -0.904911158531723 |
| 2673 | LRIG1 | 1 | 1 | 2.72426153699987 | 1.58620704787801 |
| 2674 | SUCLG2 | 4 | 1 | -4.27538941820033 | -0.907611804241133 |
| 2675 | FAM19A1 | 5 | 1 | 4.10529495756261 | -0.524517878116858 |
| 2676 | TMF1 | 12 | 1 | 1.82607604543798 | 2.4749795355184 |
| 2677 | UBA3 | 3 | 1 | -2.04335354287989 | -0.302498026909125 |
| 2678 | ARL6IP5 | 10 | 1 | 3.34204317609899 | -1.71632425409724 |
| 2679 | LMOD3 | 10 | 1 | 3.18908049146764 | -1.72405634743143 |

| 2680 | FRMD4B | 16 | 1 | -4.31984876115687 | 3.06433881896566 |
| --- | --- | --- | --- | --- | --- |
| 2681 | MITF | 4 | 1 | -4.62136481721766 | 0.633178368983972 |
| 2682 | FOXP1 | 1 | 1 | 1.66922177354924 | 0.784863368449914 |
| 2683 | EIF4E3 | 2 | 1 | -0.878156468466594 | 1.32121862548422 |
| 2684 | GPR27 | 10 | 1 | 4.53080917875402 | -0.55019123774935 |
| 2685 | RYBP | 12 | 1 | 2.78330947439306 | 2.67679108756613 |
| 2686 | SHQ1 | 11 | 1 | -3.75192569215663 | -1.72484950643946 |
| 2687 | GXYLT2 | 4 | 1 | -4.4633004518307 | -0.99629843902756 |
| 2688 | PPP4R2 | 3 | 1 | -2.61716555078395 | 0.72758437770437 |
| 2689 | EBLN2 | 8 | 1 | -1.60908554513819 | 1.59139455932211 |
| 2690 | PDZRN3 | 14 | 1 | -6.66912387330897 | -0.877351538436306 |
| 2691 | CNTN3 | 18 | 1 | 5.51485611478917 | -1.94183920246531 |
| 2692 | LINC00960 | 11 | 1 | -3.33225844819911 | -1.73623798710276 |
| 2693 | ZNF717 | 8 | 1 | -1.46432791669734 | 0.87651015895437 |
| 2694 | ROBO2 | 18 | 1 | 5.27755714933507 | -1.76835237366129 |
| 2695 | ROBO1 | 15 | 1 | 5.49169088880651 | 0.785354510722864 |
| 2696 | GBE1 | 14 | 1 | -6.31280683000453 | -0.741807385505927 |
| 2697 | CADM2 | 15 | 1 | 5.69682766477697 | 0.78078855651449 |
| 2698 | VGLL3 | 14 | 1 | -6.35788272340663 | -0.0461680732386233 |
| 2699 | CHMP2B | 13 | 1 | -1.74735091646083 | -2.44277511459757 |
| 2700 | CGGBP1 | 9 | 1 | 0.476499214574025 | 3.07488383430074 |
| 2701 | ZNF654 | 1 | 1 | 3.62067128698461 | 2.35610546248983 |
| 2702 | C3orf38 | 2 | 1 | 0.999733581944631 | 1.09647049087118 |
| 2703 | EPHA3 | 15 | 1 | 5.30085779707067 | 0.391649977145898 |
| 2704 | PROS1 | 5 | 1 | 3.05793668310277 | -0.880122876884234 |
| 2705 | ARL13B | 2 | 1 | 0.446700051232503 | 2.11094583648275 |
| 2706 | DHFR2 | 9 | 1 | 0.753167405530141 | 2.75026096480917 |
| 2707 | NSUN3 | 2 | 1 | -0.0111981632269164 | 1.59680093902181 |
| 2708 | MTRNR2L12 | 8 | 1 | -1.21506266315348 | 0.436595455585229 |
| 2709 | EPHA6 | 4 | 1 | -4.99349759538539 | -1.50901518684794 |
| 2710 | AC110491.1 | 2 | 1 | 0.312616541787312 | 2.49001135009359 |
| 2711 | ARL6 | 5 | 1 | 2.52314330618017 | -0.677971377195609 |
| 2712 | CRYBG3 | 7 | 1 | -2.85000060518153 | -3.82812820297648 |
| 2713 | RIOX2 | 7 | 1 | -2.95259545762904 | -3.71874795776774 |
| 2714 | CLDND1 | 6 | 1 | -0.0494432600296278 | -1.46498964649607 |
| 2715 | CPOX | 5 | 1 | 3.06184746305578 | -0.235208793701422 |
| 2716 | ST3GAL6 | 14 | 1 | -6.08765672166713 | -0.469434841694129 |
| 2717 | DCBLD2 | 9 | 1 | -0.451149746970012 | 2.94461526053976 |
| 2718 | COL8A1 | 14 | 1 | -6.24720786531337 | 0.0578656353337644 |
| 2719 | CMSS1 | 19 | 1 | -4.10524986703761 | 0.638926640926111 |
| 2720 | FILIP1L | 10 | 1 | 4.51877142469518 | -2.27422021252085 |
| 2721 | TBC1D23 | 2 | 1 | 1.0241274980747 | 0.980189577518213 |
| 2722 | NIT2 | 13 | 1 | -2.08703266103633 | -2.15031646114756 |
| 2723 | TOMM70 | 1 | 1 | 3.5694086698734 | 2.56349290984701 |
| 2724 | LNP1 | 16 | 1 | -4.03878305871852 | 2.89053858893942 |
| 2725 | TMEM45A | 17 | 1 | 1.90830422918432 | -0.110412880005133 |
| 2726 | TFG | 3 | 1 | -2.06108067949183 | -0.523827209295523 |
| 2727 | SENP7 | 1 | 1 | 4.30832983533971 | 1.2075108446462 |
| 2728 | TRMT10C | 3 | 1 | -2.38605783899195 | -0.757628559293044 |
| 2729 | PCNP | 3 | 1 | -2.44418667276271 | 0.66889764445852 |
| 2730 | ZBTB11 | 12 | 1 | 3.10058642904393 | 2.83221401351522 |
| 2731 | ZBTB11-AS1 | 8 | 1 | -0.738760009602382 | 0.0357675113065122 |
| 2732 | RPL24 | 21 | 1 | -0.832385346487834 | -4.39420019012858 |
| 2733 | CEP97 | 9 | 1 | -0.170593902663066 | 3.17222823279928 |
| 2734 | NXPE3 | 1 | 1 | 3.24517275373571 | 0.693527356563318 |
| 2735 | AC020651.1 | 1 | 1 | 3.48828746358983 | 1.24376310485433 |
| 2736 | NFKBIZ | 14 | 1 | -5.85594676454432 | 0.316993252216089 |
| 2737 | ALCAM | 18 | 1 | 6.18470026533239 | -2.15450583321024 |
| 2738 | CBLB | 1 | 1 | 3.15330912153356 | 1.09293831962179 |
| 2739 | LINC00882 | 10 | 1 | 4.78472830335729 | -0.657174064935935 |
| 2740 | DUBR | 6 | 1 | 1.96579433958165 | -2.40855275017191 |
| 2741 | BBX | 1 | 1 | 2.41744543592565 | 1.36524333137106 |
| 2742 | CD47 | 5 | 1 | 4.1636960653507 | -0.297842308105719 |
| 2743 | IFT57 | 13 | 1 | -1.71435557325251 | -1.80049399954249 |
| 2744 | CIP2A | 16 | 1 | -4.60849116762049 | 2.89905847686361 |
| 2745 | DZIP3 | 15 | 1 | 5.59015442411535 | 0.819471851764428 |
| 2746 | DPPA4 | 11 | 1 | -3.81664322336085 | -1.90816019398142 |

| 2747 | NECTIN3 | 8 | 1 | -1.35458229501612 | 0.0875327147824644 |
| --- | --- | --- | --- | --- | --- |
| 2748 | PLCXD2 | 15 | 1 | 5.21908260862462 | 0.257379189906824 |
| 2749 | PHLDB2 | 7 | 1 | -3.78911326845057 | -3.19764314514567 |
| 2750 | ABHD10 | 12 | 1 | 1.99987246076696 | 2.07176841872762 |
| 2751 | TAGLN3 | 10 | 1 | 3.66169239561193 | -1.79142896753718 |
| 2752 | AC112487.1 | 11 | 1 | -3.94977615793116 | -2.27933762890269 |
| 2753 | CD200 | 15 | 1 | 4.71613527814977 | -0.362521215500128 |
| 2754 | ATG3 | 1 | 1 | 3.35224868337743 | 0.763740316806543 |
| 2755 | CCDC80 | 14 | 1 | -6.16946195085414 | 0.0232598938329099 |
| 2756 | AC074044.1 | 2 | 1 | 0.553733005925343 | 0.887184397159326 |
| 2757 | GTPBP8 | 3 | 1 | -1.8339389415539 | -0.717626079143775 |
| 2758 | NEPRO | 2 | 1 | -0.927509710387065 | 1.06928433555197 |
| 2759 | BOC | 4 | 1 | -5.15271781404383 | -0.439066394390357 |
| 2760 | CFAP44 | 8 | 1 | -2.00566063840754 | 1.64401711600851 |
| 2761 | SPICE1 | 2 | 1 | -1.11782871206172 | 1.3793391146047 |
| 2762 | USF3 | 9 | 1 | 0.622556045457051 | 2.78512920516561 |
| 2763 | NAA50 | 8 | 1 | -2.31525133569606 | 1.26479233878683 |
| 2764 | ATP6V1A | 5 | 1 | 4.49757458250158 | 0.557150021968591 |
| 2765 | GRAMD1C | 4 | 1 | -4.45902989824183 | 0.0108712472302793 |
| 2766 | CCDC191 | 1 | 1 | 2.60756732504003 | 1.44087828773092 |
| 2767 | QTRT2 | 3 | 1 | -3.12459681947596 | 0.156001583514917 |
| 2768 | ZBTB20 | 1 | 1 | 3.65210987608068 | 1.28813447135519 |
| 2769 | ZBTB20-AS2 | 7 | 1 | -3.04464291055567 | -2.7851170144694 |
| 2770 | GAP43 | 10 | 1 | 3.69430448095434 | -1.65170310360361 |
| 2771 | LSAMP | 6 | 1 | 1.54844523946874 | -2.27088616711069 |
| 2772 | TUSC7 | 1 | 1 | 2.40193916837804 | 0.857102052150476 |
| 2773 | AC092691.1 | 1 | 1 | 3.86960197965734 | 1.39980830329488 |
| 2774 | LINC02024 | 12 | 1 | 2.31731368581884 | 1.82471336501669 |
| 2775 | IGSF11 | 7 | 1 | -2.12039385755427 | -3.6576961122172 |
| 2776 | UPK1B | 2 | 1 | 0.777134910031484 | 1.85009159225057 |
| 2777 | B4GALT4 | 7 | 1 | -3.26051066835292 | -2.5864310107844 |
| 2778 | ARHGAP31 | 7 | 1 | -3.78075407464869 | -3.23824749809672 |
| 2779 | TMEM39A | 3 | 1 | -3.19173215349086 | -0.69818264168669 |
| 2780 | POGLUT1 | 8 | 1 | -0.329153031305148 | -0.464059784235251 |
| 2781 | AC073352.1 | 16 | 1 | -4.17418263872035 | 2.67702569144796 |
| 2782 | TIMMDC1 | 3 | 1 | -1.95472751577265 | -0.719219862641585 |
| 2783 | COX17 | 6 | 1 | 0.881429448529408 | -2.00460110527445 |
| 2784 | MAATS1 | 2 | 1 | -0.268277047396376 | 1.21904172080587 |
| 2785 | GSK3B | 1 | 1 | 3.59636189024083 | 2.18400468009542 |
| 2786 | AC092910.3 | 8 | 1 | -2.4090099187649 | 1.26245941299032 |
| 2787 | GPR156 | 2 | 1 | 1.13095821420781 | 1.4642361559255 |
| 2788 | LRRC58 | 9 | 1 | -0.381175577834918 | 2.671225205837 |
| 2789 | FSTL1 | 7 | 1 | -3.47710679490931 | -2.801555379452 |
| 2790 | NDUFB4 | 6 | 1 | 0.505288496419118 | -2.36689363342692 |
| 2791 | RABL3 | 8 | 1 | -2.23234425504573 | 1.31773509162496 |
| 2792 | GTF2E1 | 9 | 1 | 0.168200656577275 | 2.60128583091329 |
| 2793 | STXBP5L | 15 | 1 | 5.28574444334142 | 0.960603968082178 |
| 2794 | POLQ | 19 | 1 | -4.97918151338465 | 1.97674430984091 |
| 2795 | GOLGB1 | 2 | 1 | 0.125833764477895 | 2.11551560538839 |
| 2796 | IQCB1 | 4 | 1 | -4.70494626481898 | 0.568126574931848 |
| 2797 | EAF2 | 14 | 1 | -5.82387803514369 | 0.0691909350736021 |
| 2798 | SLC15A2 | 9 | 1 | 0.878261938496755 | 2.60245479720663 |
| 2799 | CCDC58 | 3 | 1 | -3.32753060777552 | -0.962097830297244 |
| 2800 | FAM162A | 13 | 1 | -0.33199636657603 | -2.57980333191324 |
| 2801 | WDR5B | 3 | 1 | -1.74075268228419 | 0.0546742238385557 |
| 2802 | AC083798.2 | 21 | 1 | -1.13087551315196 | -4.66104327064921 |
| 2803 | KPNA1 | 5 | 1 | 3.23260547201268 | 0.00179655927251565 |
| 2804 | PARP9 | 14 | 1 | -6.84994433839686 | -0.545859947027457 |
| 2805 | DTX3L | 14 | 1 | -6.62007258851893 | -0.543623103918326 |
| 2806 | PARP14 | 4 | 1 | -4.63860724885829 | -0.617745652021659 |
| 2807 | HSPBAP1 | 2 | 1 | -1.01078270395167 | 1.9940110601766 |
| 2808 | DIRC2 | 14 | 1 | -5.68296216447718 | 0.0505511321408628 |
| 2809 | LINC02035 | 1 | 1 | 3.26665784399144 | 1.6052969850881 |
| 2810 | SEMA5B | 14 | 1 | -6.81786750276454 | -0.781084492029441 |
| 2811 | PDIA5 | 14 | 1 | -6.54020999391444 | -0.578774972976935 |
| 2812 | ADCY5 | 15 | 1 | 5.01736523191564 | 0.546010867534387 |
| 2813 | AC112503.1 | 1 | 1 | 3.37426878492467 | 0.660771027980554 |

| 2814 | HACD2 | 1 | 1 | 3.31023766080968 | 1.62866987365316 |
| --- | --- | --- | --- | --- | --- |
| 2815 | MYLK-AS1 | 4 | 1 | -3.94107507188685 | -0.47064120990206 |
| 2816 | MYLK | 14 | 1 | -5.03058074434169 | 0.479164973674524 |
| 2817 | CCDC14 | 16 | 1 | -3.22503779847987 | 2.16063846725057 |
| 2818 | KALRN | 12 | 1 | 1.55241383592717 | 2.06691159385275 |
| 2819 | UMPS | 8 | 1 | -1.81072245557673 | 0.668103591380823 |
| 2820 | ITGB5 | 14 | 1 | -6.31616471727259 | -0.22135965448786 |
| 2821 | HEG1 | 14 | 1 | -5.62399409730799 | -0.161377235473883 |
| 2822 | SLC12A8 | 14 | 1 | -6.84895013292201 | -0.535234942259085 |
| 2823 | ZNF148 | 1 | 1 | 3.60478307287328 | 2.51810254233907 |
| 2824 | SNX4 | 10 | 1 | 3.38130499403112 | -1.52929834944178 |
| 2825 | OSBPL11 | 9 | 1 | -0.262023759268894 | 2.96905769485067 |
| 2826 | ALG1L | 11 | 1 | -4.10762141664393 | -2.04262553078104 |
| 2827 | ROPN1B | 18 | 1 | 6.12294699232213 | -2.15624187809397 |
| 2828 | KLF15 | 4 | 1 | -4.49144767244227 | -0.261153384269965 |
| 2829 | ZXDC | 2 | 1 | -0.424826935128047 | 1.55504812377523 |
| 2830 | CHST13 | 7 | 1 | -3.43951509912379 | -3.72622404915262 |
| 2831 | TXNRD3 | 7 | 1 | -3.58977650125392 | -2.67062006813456 |
| 2832 | CHCHD6 | 11 | 1 | -2.90343569238551 | -1.40860603672434 |
| 2833 | PLXNA1 | 7 | 1 | -2.69116400201686 | -3.70684657913615 |
| 2834 | TPRA1 | 7 | 1 | -3.56878827531703 | -2.72172449451853 |
| 2835 | MCM2 | 19 | 1 | -5.11762593706019 | 0.93216611522268 |
| 2836 | PODXL2 | 5 | 1 | 3.24451900999181 | -0.745127125801337 |
| 2837 | ABTB1 | 13 | 1 | 0.288223877354787 | -3.06511245590616 |
| 2838 | MGLL | 18 | 1 | 5.7588355688297 | -1.398404791178 |
| 2839 | SEC61A1 | 2 | 1 | -0.870637759760692 | 1.60194338935446 |
| 2840 | RUVBL1 | 4 | 1 | -3.49631141145594 | -0.570242538275015 |
| 2841 | EEFSEC | 2 | 1 | 1.42024745027654 | 0.87294890063833 |
| 2842 | GATA2 | 20 | 1 | 3.24350048582189 | -3.97853241783549 |
| 2843 | RPN1 | 5 | 1 | 3.60112024824254 | 0.363796726642358 |
| 2844 | RAB7A | 17 | 1 | 0.549786701604054 | -0.0127643190043093 |
| 2845 | ACAD9 | 8 | 1 | -1.61489079912074 | 0.275973693309533 |
| 2846 | KIAA1257 | 14 | 1 | -5.98890136201747 | -0.0768501005785586 |
| 2847 | EFCC1 | 14 | 1 | -6.64247249086268 | -0.337133034290564 |
| 2848 | ISY1 | 3 | 1 | -2.82273601014979 | -1.02643576187063 |
| 2849 | AC108673.3 | 10 | 1 | 3.91327644865148 | -1.06354574781824 |
| 2850 | CNBP | 3 | 1 | -2.56759355981715 | -0.409895046772254 |
| 2851 | COPG1 | 12 | 1 | 1.50821234266393 | 1.95729245322775 |
| 2852 | HMCES | 13 | 1 | -1.05223850925334 | -0.51618529778887 |
| 2853 | H1FX | 16 | 1 | -4.2763035150326 | 2.1428934492452 |
| 2854 | H1FX-AS1 | 11 | 1 | -3.28691647966273 | -1.43243949037958 |
| 2855 | EFCAB12 | 7 | 1 | -2.04073546846278 | -2.92281971794535 |
| 2856 | MBD4 | 2 | 1 | 0.878728762074635 | 1.61986030715536 |
| 2857 | IFT122 | 8 | 1 | -2.56571434457667 | 2.59279193061422 |
| 2858 | TMCC1 | 1 | 1 | 4.22204424421422 | 1.23849858420919 |
| 2859 | TMCC1-AS1 | 4 | 1 | -5.02867959459193 | -0.220287068905127 |
| 2860 | TRH | 6 | 1 | 1.51924516241185 | -2.73299644810129 |
| 2861 | COL6A6 | 9 | 1 | 1.40243662874334 | 2.77544368880819 |
| 2862 | ATP2C1 | 1 | 1 | 3.67603208105199 | 0.685561672626245 |
| 2863 | NUDT16 | 13 | 1 | -1.64793895204432 | -2.50983260494639 |
| 2864 | MRPL3 | 3 | 1 | -3.07527326066859 | -0.410041137756598 |
| 2865 | CPNE4 | 6 | 1 | 1.50029541532628 | -2.80161724907328 |
| 2866 | DNAJC13 | 9 | 1 | 0.624445810719655 | 2.9695376314504 |
| 2867 | UBA5 | 3 | 1 | -2.51369546373255 | -0.561467959703696 |
| 2868 | NPHP3 | 8 | 1 | -2.14756105859645 | 1.09716691154073 |
| 2869 | TMEM108 | 15 | 1 | 5.2225282339298 | 0.38449777740072 |
| 2870 | CDV3 | 8 | 1 | -1.25327752550013 | 1.34667052405905 |
| 2871 | TOPBP1 | 9 | 1 | -0.715031370714976 | 2.89077009337972 |
| 2872 | SRPRB | 14 | 1 | -5.46819613893397 | -0.0762973271029116 |
| 2873 | RAB6B | 10 | 1 | 3.52344442884557 | -1.45684681755472 |
| 2874 | RYK | 11 | 1 | -3.54973958452113 | -1.75762705427576 |
| 2875 | AMOTL2 | 11 | 1 | -4.36916802842982 | -1.50955299955775 |
| 2876 | ANAPC13 | 13 | 1 | -0.294323392406776 | -0.763549029053939 |
| 2877 | CEP63 | 3 | 1 | -3.0369396062649 | -0.844980715932143 |
| 2878 | EPHB1 | 15 | 1 | 4.82258965055578 | 0.507161036906946 |
| 2879 | KY | 15 | 1 | 5.76322676221959 | 0.809474007068384 |
| 2880 | PPP2R3A | 12 | 1 | 2.89520670454137 | 2.43392099517416 |

| 2881 | MSL2 | 12 | 1 | 2.67776824514501 | 2.93919052260946 |
| --- | --- | --- | --- | --- | --- |
| 2882 | PCCB | 11 | 1 | -3.21854804475672 | -1.57641093355585 |
| 2883 | STAG1 | 9 | 1 | -0.0641855897463103 | 3.10429467338155 |
| 2884 | SLC35G2 | 10 | 1 | 3.01283790151708 | -1.37364266735483 |
| 2885 | AC096992.2 | 1 | 1 | 3.16925598661535 | 1.01112498420309 |
| 2886 | NCK1-DT | 5 | 1 | 4.23575378934972 | 0.0498649634702085 |
| 2887 | NCK1 | 1 | 1 | 3.56063462774389 | 0.832877055583703 |
| 2888 | SOX14 | 20 | 1 | 3.31702877561681 | -3.87399206978251 |
| 2889 | DZIP1L | 16 | 1 | -4.53106616456874 | 1.91700662749838 |
| 2890 | DBR1 | 2 | 1 | 0.119769796534703 | 1.73163379806112 |
| 2891 | ARMC8 | 1 | 1 | 3.48716117422216 | 2.11852444785665 |
| 2892 | NME9 | 15 | 1 | 4.78507544080846 | 0.0382325209958433 |
| 2893 | MRAS | 5 | 1 | 3.90670277158849 | -0.773846789421332 |
| 2894 | ESYT3 | 16 | 1 | -4.26083491762049 | 3.28781022208761 |
| 2895 | CEP70 | 16 | 1 | -4.12308143098719 | 2.9285220064504 |
| 2896 | FAIM | 20 | 1 | 3.40823604147069 | -3.58763180595804 |
| 2897 | PIK3CB | 1 | 1 | 3.71709825079076 | 1.53135813849996 |
| 2898 | MRPS22 | 3 | 1 | -2.50542877633937 | -0.336783155025733 |
| 2899 | COPB2 | 3 | 1 | -2.3267094941891 | -0.637419893564475 |
| 2900 | RBP1 | 6 | 1 | 2.03138948957555 | -2.58343051296641 |
| 2901 | NMNAT3 | 1 | 1 | 1.55885077993505 | 1.47090472358297 |
| 2902 | AC110716.1 | 18 | 1 | 6.26180531065099 | -2.43495510441233 |
| 2903 | CLSTN2 | 6 | 1 | 2.48044826070897 | -1.91927443367411 |
| 2904 | SLC25A36 | 1 | 1 | 3.91851975004308 | 1.19961537498068 |
| 2905 | SPSB4 | 14 | 1 | -5.58094571550257 | -1.00215425265003 |
| 2906 | PXYLP1 | 1 | 1 | 4.60512806455724 | 0.89466942924093 |
| 2907 | ZBTB38 | 1 | 1 | 4.36830307523839 | 2.15914239066671 |
| 2908 | RASA2 | 12 | 1 | 2.27803613226049 | 2.50109829085897 |
| 2909 | RNF7 | 13 | 1 | -0.703041598395183 | -2.48913048130442 |
| 2910 | ATP1B3 | 2 | 1 | 0.0117380468093614 | 0.532640472827661 |
| 2911 | TFDP2 | 11 | 1 | -3.80095814187892 | -1.67472182136942 |
| 2912 | GK5 | 12 | 1 | 2.21850039045446 | 2.77504624503683 |
| 2913 | XRN1 | 12 | 1 | 1.75851632635228 | 2.68656458037924 |
| 2914 | ATR | 2 | 1 | -1.0185606332577 | 1.80646981376241 |
| 2915 | TRPC1 | 9 | 1 | 1.34727074186437 | 3.07181562560629 |
| 2916 | U2SURP | 2 | 1 | 0.523433998032735 | 1.48993505614828 |
| 2917 | AC026304.1 | 4 | 1 | -3.71844695527919 | -0.112401887001288 |
| 2918 | CHST2 | 2 | 1 | 0.159493997498677 | 0.689635888515222 |
| 2919 | C3orf58 | 7 | 1 | -3.01377533395655 | -3.53122197014262 |
| 2920 | PLOD2 | 11 | 1 | -4.00236438234217 | -1.27122436386515 |
| 2921 | PLSCR4 | 4 | 1 | -4.97521851976283 | -0.274651511730445 |
| 2922 | PLSCR1 | 14 | 1 | -5.51492188890345 | 0.433689967571008 |
| 2923 | AC092957.1 | 11 | 1 | -4.1698868127621 | -1.94989798885752 |
| 2924 | ZIC4 | 11 | 1 | -4.23637960870631 | -1.85352264267374 |
| 2925 | ZIC4-AS1 | 4 | 1 | -4.53311036546595 | -1.58098439794947 |
| 2926 | ZIC1 | 11 | 1 | -3.82794354875453 | -2.1933052383082 |
| 2927 | AC092958.1 | 4 | 1 | -4.69696687181361 | -1.47488747459818 |
| 2928 | LINC02032 | 4 | 1 | -4.38574431856044 | -1.4464208922999 |
| 2929 | GYG1 | 14 | 1 | -5.89195798356898 | -0.751643507899535 |
| 2930 | HLTF | 5 | 1 | 3.60435654203527 | -0.592463298858893 |
| 2931 | HPS3 | 1 | 1 | 3.02610470335119 | 1.2541359819753 |
| 2932 | CP | 1 | 1 | 2.21564103643529 | 1.13698782104086 |
| 2933 | WWTR1 | 14 | 1 | -6.70308278520472 | -0.463034375728858 |
| 2934 | WWTR1-AS1 | 14 | 1 | -5.16306374986537 | -0.105397149147284 |
| 2935 | COMMD2 | 6 | 1 | 1.716537609502 | -2.07033978325297 |
| 2936 | RNF13 | 3 | 1 | -2.42277358491786 | -0.925447129802597 |
| 2937 | PFN2 | 8 | 1 | -1.15302424152262 | 0.00284464734624612 |
| 2938 | TSC22D2 | 12 | 1 | 2.73780585806005 | 2.18055309432577 |
| 2939 | SERP1 | 11 | 1 | -3.00645850618251 | -1.47235427719523 |
| 2940 | EIF2A | 3 | 1 | -2.30834649522669 | -0.896711703548026 |
| 2941 | SELENOT | 5 | 1 | 3.34247757475011 | -0.536618187250388 |
| 2942 | ERICH6-AS1 | 13 | 1 | -1.3055673690594 | -2.63926039558817 |
| 2943 | SIAH2 | 10 | 1 | 4.20853592435949 | -1.75907204967905 |
| 2944 | MED12L | 18 | 1 | 5.47306086103551 | -0.4097593269961 |
| 2945 | P2RY14 | 12 | 1 | 1.81736327688329 | 2.51602234023641 |
| 2946 | IGSF10 | 18 | 1 | 5.04235054533117 | -0.754538967432273 |
| 2947 | MBNL1 | 11 | 1 | -3.34618519266017 | -2.30626211983134 |

| 2948 | MBNL1-AS1 | 11 | 1 | -4.06586645563014 | -2.24029062611033 |
| --- | --- | --- | --- | --- | --- |
| 2949 | P2RY1 | 18 | 1 | 5.5110795644962 | -1.82032476288248 |
| 2950 | RAP2B | 9 | 1 | 0.462937131329701 | 2.73700465339254 |
| 2951 | ARHGEF26 | 6 | 1 | 1.89586378614538 | -2.65709434372355 |
| 2952 | DHX36 | 1 | 1 | 3.94934584180944 | 0.824430362163293 |
| 2953 | GPR149 | 15 | 1 | 4.70561529676549 | -0.400893493713629 |
| 2954 | MME | 20 | 1 | 3.4718105940067 | -3.7267137609141 |
| 2955 | PLCH1 | 2 | 1 | -0.708841934994533 | 1.29176367896627 |
| 2956 | SLC33A1 | 9 | 1 | 1.27619971315496 | 2.82454694884847 |
| 2957 | GMPS | 7 | 1 | -3.45626018960841 | -2.78986500126292 |
| 2958 | AC084036.1 | 5 | 1 | 3.71095754186742 | -0.444621785702002 |
| 2959 | SSR3 | 3 | 1 | -3.33081100900538 | -0.9411563567536 |
| 2960 | TIPARP-AS1 | 11 | 1 | -3.04651521165736 | -1.79999236685206 |
| 2961 | TIPARP | 15 | 1 | 5.76961590330236 | 0.528823749004114 |
| 2962 | LEKR1 | 14 | 1 | -5.56875394304164 | 0.230760113178003 |
| 2963 | CCNL1 | 2 | 1 | -0.598553016976192 | 1.35296668189596 |
| 2964 | VEPH1 | 14 | 1 | -5.08247731645472 | 0.268547192989099 |
| 2965 | PTX3 | 14 | 1 | -6.69470666368373 | -0.399123056949866 |
| 2966 | SHOX2 | 20 | 1 | 3.36444570104711 | -3.80717073303629 |
| 2967 | RSRC1 | 2 | 1 | -0.312950492099597 | 1.79717840331625 |
| 2968 | AC106707.1 | 6 | 1 | 2.10705734769933 | -2.216913922848 |
| 2969 | MLF1 | 13 | 1 | -0.0765522720373412 | -2.71945975643564 |
| 2970 | GFM1 | 12 | 1 | 2.92729331533544 | 2.25016678947042 |
| 2971 | LXN | 11 | 1 | -3.50659607370265 | -1.54954646450449 |
| 2972 | MFSD1 | 10 | 1 | 3.29816533605687 | -1.71245859486033 |
| 2973 | AC080013.1 | 12 | 1 | 2.20503665487401 | 1.6028031267507 |
| 2974 | SCHIP1 | 20 | 1 | 3.13811923544042 | -3.47131238800455 |
| 2975 | IFT80 | 3 | 1 | -2.84953878839381 | 0.563802615581262 |
| 2976 | SMC4 | 16 | 1 | -4.52122662980921 | 2.42423118728231 |
| 2977 | TRIM59 | 16 | 1 | -4.40109513719447 | 3.01160325187277 |
| 2978 | KPNA4 | 12 | 1 | 2.03934837858312 | 2.61802519935201 |
| 2979 | PPM1L | 9 | 1 | 1.24996699373357 | 2.3029652990682 |
| 2980 | B3GALNT1 | 5 | 1 | 3.74821259061925 | -0.251074000419867 |
| 2981 | NMD3 | 3 | 1 | -1.80482529123194 | -0.409462078632605 |
| 2982 | SLITRK3 | 15 | 1 | 5.49393202345006 | -0.180582507671607 |
| 2983 | BCHE | 14 | 1 | -6.49887965639003 | -1.13534846824576 |
| 2984 | WDR49 | 4 | 1 | -4.86231826265223 | -0.647567882360709 |
| 2985 | PDCD10 | 13 | 1 | -1.85061500985987 | -1.94748018604685 |
| 2986 | SERPINI1 | 7 | 1 | -2.92272661645777 | -3.73403726440836 |
| 2987 | GOLIM4 | 11 | 1 | -3.08824346979029 | -1.79262153726984 |
| 2988 | LINC02082 | 18 | 1 | 5.58858992139928 | -0.575525506557715 |
| 2989 | AC092954.1 | 18 | 1 | 5.52647615949743 | -0.659763558925879 |
| 2990 | AC092954.2 | 18 | 1 | 5.48318077604406 | -0.469742729486716 |
| 2991 | MECOM | 11 | 1 | -3.97154806573756 | -2.39146540981699 |
| 2992 | MYNN | 1 | 1 | 3.70838715116613 | 1.60786308425497 |
| 2993 | LRRC34 | 8 | 1 | -1.37062130887873 | 1.47167362350057 |
| 2994 | SEC62 | 10 | 1 | 3.34608794729345 | -1.81356553656031 |
| 2995 | PHC3 | 9 | 1 | 1.96004821340673 | 3.05621232169698 |
| 2996 | AC008040.5 | 9 | 1 | 1.43587030450933 | 2.93956436294149 |
| 2997 | PRKCI | 2 | 1 | 0.600339248582051 | 2.13754429000448 |
| 2998 | SKIL | 5 | 1 | 3.48296333829992 | -0.232526346267951 |
| 2999 | SLC7A14 | 15 | 1 | 5.14202810804479 | -0.411800190033209 |
| 3000 | RPL22L1 | 4 | 1 | -4.46035169084437 | -0.0016563974039675 |
| 3001 | EIF5A2 | 4 | 1 | -3.64661596734889 | -0.235603972496283 |
| 3002 | TNIK | 5 | 1 | 3.71572257558935 | -0.159323795856726 |
| 3003 | PLD1 | 6 | 1 | 1.92365027944677 | -2.5418182454722 |
| 3004 | FNDC3B | 1 | 1 | 2.56899930517309 | 1.5672942556722 |
| 3005 | NCEH1 | 14 | 1 | -5.38318608720668 | -0.391045614303839 |
| 3006 | ECT2 | 16 | 1 | -4.45761607606776 | 3.12700070518087 |
| 3007 | NLGN1 | 2 | 1 | 0.85160662214391 | 1.87286581176351 |
| 3008 | LINC01209 | 2 | 1 | 1.35060490648382 | 0.776535169063318 |
| 3009 | TBL1XR1 | 1 | 1 | 3.40621639768712 | 0.41426171439718 |
| 3010 | KCNMB2-AS1 | 11 | 1 | -3.09520243127711 | -2.03477297645975 |
| 3011 | ZMAT3 | 14 | 1 | -5.9426328988827 | 0.151141539989221 |
| 3012 | PIK3CA | 12 | 1 | 2.79641677419774 | 2.0082875646932 |
| 3013 | KCNMB3 | 1 | 1 | 2.34635354559057 | 0.919533149181116 |
| 3014 | ZNF639 | 1 | 1 | 2.97318245451085 | 1.20795835631918 |

| 3015 | MFN1 | 8 | 1 | -1.38800595720179 | 1.79181398528646 |
| --- | --- | --- | --- | --- | --- |
| 3016 | GNB4 | 13 | 1 | -1.03760634382136 | -2.71510694843699 |
| 3017 | ACTL6A | 4 | 1 | -3.74057410676844 | 0.262485281406152 |
| 3018 | MRPL47 | 3 | 1 | -2.26920531709559 | -0.572704031290305 |
| 3019 | NDUFB5 | 3 | 1 | -1.88558207471736 | -1.37745146376063 |
| 3020 | USP13 | 16 | 1 | -3.96265600641139 | 2.66732181685995 |
| 3021 | PEX5L | 20 | 1 | 3.21482612173192 | -3.39667306763102 |
| 3022 | TTC14 | 2 | 1 | -0.773781761721446 | 1.40573395865988 |
| 3023 | CCDC39 | 8 | 1 | -2.09685729463465 | 1.54259909766744 |
| 3024 | FXR1 | 3 | 1 | -2.87622974832423 | 0.10778035300802 |
| 3025 | DNAJC19 | 11 | 1 | -2.96263335664637 | -1.46973471266199 |
| 3026 | SOX2 | 4 | 1 | -4.54625199754603 | -0.504727943958533 |
| 3027 | ATP11B | 8 | 1 | -1.67826257665522 | 1.76540674346517 |
| 3028 | DCUN1D1 | 9 | 1 | 1.85590960065954 | 2.83645667213033 |
| 3029 | MCCC1 | 2 | 1 | -0.716472432211711 | 1.49794520515035 |
| 3030 | MCF2L2 | 12 | 1 | 2.28265692274206 | 2.47712005752157 |
| 3031 | B3GNT5 | 3 | 1 | -3.4596600385464 | 1.02537299769949 |
| 3032 | KLHL24 | 12 | 1 | 1.53546346704595 | 2.01364483016561 |
| 3033 | YEATS2 | 9 | 1 | 0.00265251080624862 | 2.64350356238912 |
| 3034 | PARL | 3 | 1 | -2.29897044618495 | -1.00033061874081 |
| 3035 | ABCC5 | 1 | 1 | 3.88140179197423 | 2.0510167040212 |
| 3036 | DVL3 | 2 | 1 | 0.737505987092183 | 1.54114593642782 |
| 3037 | AP2M1 | 5 | 1 | 3.33350326101415 | -0.723928942503226 |
| 3038 | ABCF3 | 7 | 1 | -3.26315520723231 | -3.34741340500284 |
| 3039 | VWA5B2 | 7 | 1 | -2.86195395906336 | -3.7460054002421 |
| 3040 | ALG3 | 3 | 1 | -2.00248943288691 | -1.06010837775637 |
| 3041 | EEF1AKMT4 | 3 | 1 | -3.45583842714198 | -0.794754645945323 |
| 3042 | CAMK2N2 | 10 | 1 | 4.25705458204381 | -1.51846991402079 |
| 3043 | PSMD2 | 8 | 1 | -0.576800450877025 | 0.145475045619714 |
| 3044 | EIF4G1 | 2 | 1 | -0.923129603461101 | 1.75779856818746 |
| 3045 | FAM131A | 5 | 1 | 3.75573612730138 | 0.105921880183923 |
| 3046 | POLR2H | 3 | 1 | -2.69326566179164 | -0.593368633808386 |
| 3047 | EPHB3 | 8 | 1 | -0.877876147822215 | 0.427512780605066 |
| 3048 | MAGEF1 | 17 | 1 | 0.234067126914189 | -0.11629043442179 |
| 3049 | AC107294.3 | 10 | 1 | 2.93592978994481 | -1.3789174698012 |
| 3050 | VPS8 | 1 | 1 | 3.73523975889318 | 2.51249398368429 |
| 3051 | C3orf70 | 15 | 1 | 5.09283615629308 | 0.00325061458181131 |
| 3052 | MAP3K13 | 5 | 1 | 3.31049658338659 | -1.04152938646246 |
| 3053 | TMEM41A | 10 | 1 | 3.98724295179479 | -2.28875361305643 |
| 3054 | SENP2 | 12 | 1 | 2.1713156847202 | 2.94052685874532 |
| 3055 | IGF2BP2 | 9 | 1 | 0.443171933098958 | 2.63957323211263 |
| 3056 | TRA2B | 8 | 1 | -2.96595381219752 | 1.44628371375631 |
| 3057 | ETV5 | 14 | 1 | -6.50955890138514 | -0.349298282684577 |
| 3058 | TBCCD1 | 2 | 1 | -0.871316418246104 | 2.85663570540975 |
| 3059 | DNAJB11 | 17 | 1 | 0.0219300536357622 | -0.218276783050788 |
| 3060 | EIF4A2 | 15 | 1 | 4.85618568937413 | 0.59018613475393 |
| 3061 | RFC4 | 19 | 1 | -4.82279466112025 | 1.72189654487203 |
| 3062 | ST6GAL1 | 5 | 1 | 3.39296175520055 | 0.0204972185475706 |
| 3063 | RPL39L | 16 | 1 | -4.10012148340113 | 2.13229645865988 |
| 3064 | RTP1 | 10 | 1 | 4.78635622541539 | -0.603619470419181 |
| 3065 | MASP1 | 15 | 1 | 5.86640383283727 | 0.791104332385766 |
| 3066 | SST | 20 | 1 | 3.62561775724523 | -3.44934235435892 |
| 3067 | BCL6 | 19 | 1 | -4.39505837877162 | 0.643569604335534 |
| 3068 | LPP-AS2 | 4 | 1 | -4.40886567552455 | -0.592002197326911 |
| 3069 | LPP | 3 | 1 | -2.40568827112086 | 0.411350146709192 |
| 3070 | P3H2 | 14 | 1 | -6.04262803514369 | -0.0714097462313296 |
| 3071 | IL1RAP | 18 | 1 | 5.47335506956212 | -2.09827350002695 |
| 3072 | GMNC | 7 | 1 | -3.21049474199183 | -3.62252603393961 |
| 3073 | CCDC50 | 7 | 1 | -2.7561414094723 | -2.37228284698893 |
| 3074 | FGF12 | 10 | 1 | 4.33020283262365 | -1.77479271513392 |
| 3075 | MB21D2 | 1 | 1 | 1.34679390470617 | 1.5498222269399 |
| 3076 | HRASLS | 7 | 1 | -3.05567859132655 | -3.98400436264445 |
| 3077 | ATP13A5 | 7 | 1 | -3.09062860925563 | -3.91942749840189 |
| 3078 | ATP13A5-AS1 | 7 | 1 | -3.08494471032984 | -3.92990027290751 |
| 3079 | OPA1 | 12 | 1 | 2.4464576391422 | 2.8693350710256 |
| 3080 | HES1 | 14 | 1 | -5.88230584581263 | 0.0886648454053282 |
| 3081 | ATP13A3 | 9 | 1 | 1.26614774744146 | 2.94103683608602 |

| 3082 | TMEM44-AS1 | 11 | 1 | -3.38039039094813 | -1.49626146179606 |
| --- | --- | --- | --- | --- | --- |
| 3083 | TMEM44 | 1 | 1 | 2.92851091901891 | 1.05462886470388 |
| 3084 | LSG1 | 3 | 1 | -2.42348288019068 | 0.473817244945276 |
| 3085 | FAM43A | 7 | 1 | -2.84415791948207 | -3.78650699478556 |
| 3086 | XXYLT1 | 3 | 1 | -1.95027266462214 | -0.0932996116297365 |
| 3087 | ACAP2 | 12 | 1 | 2.10744835416906 | 3.2680486597402 |
| 3088 | PPP1R2 | 10 | 1 | 2.99722887556188 | -1.34732089859415 |
| 3089 | MUC20-OT1 | 1 | 1 | 1.91310240308873 | 1.23919905799459 |
| 3090 | TNK2 | 6 | 1 | 2.54816414396398 | -1.58827160221506 |
| 3091 | TNK2-AS1 | 6 | 1 | 1.8023872522556 | -2.67952094894816 |
| 3092 | TFRC | 12 | 1 | 2.78535868208043 | 2.18494261878561 |
| 3093 | ZDHHC19 | 16 | 1 | -3.9231028409756 | 2.28042759078573 |
| 3094 | SLC51A | 1 | 1 | 2.69710017721288 | 1.42694486754965 |
| 3095 | PCYT1A | 2 | 1 | 0.0128966120921831 | 2.28433717864584 |
| 3096 | TCTEX1D2 | 6 | 1 | 2.00602628271215 | -1.97255133015085 |
| 3097 | UBXN7 | 12 | 1 | 2.53916670362584 | 2.63607229369711 |
| 3098 | RNF168 | 9 | 1 | -0.429690659240558 | 3.0687774576528 |
| 3099 | FBXO45 | 15 | 1 | 4.37438847105138 | 0.0164101876599668 |
| 3100 | PIGX | 5 | 1 | 3.66663862745397 | -0.342721148552191 |
| 3101 | CEP19 | 10 | 1 | 4.09221912901037 | -0.968748903991473 |
| 3102 | PAK2 | 1 | 1 | 3.39255572836034 | 0.790117994724023 |
| 3103 | SENP5 | 12 | 1 | 1.56589199583165 | 1.9468759455068 |
| 3104 | NCBP2 | 3 | 1 | -2.02978120763667 | -0.0800207100527407 |
| 3105 | NCBP2-AS1 | 17 | 1 | 1.88465334455602 | 0.625096694408166 |
| 3106 | NCBP2-AS2 | 7 | 1 | -3.18568561990626 | -3.3188916288035 |
| 3107 | MELTF | 6 | 1 | 1.40012051145665 | -0.843815296115172 |
| 3108 | MELTF-AS1 | 17 | 1 | 1.80849982778661 | -0.549794330419791 |
| 3109 | DLG1 | 8 | 1 | -1.20761315544017 | 1.52637900489401 |
| 3110 | BDH1 | 5 | 1 | 2.47101308385961 | -0.0830328307764651 |
| 3111 | RUBCN | 1 | 1 | 4.13911367933385 | 0.913805858073938 |
| 3112 | FYTTD1 | 1 | 1 | 3.5684218553745 | 2.05367983954977 |
| 3113 | LRCH3 | 9 | 1 | 1.19941426794164 | 2.68913473266195 |
| 3114 | RPL35A | 21 | 1 | -0.724748537138774 | -4.32290469032694 |
| 3115 | LMLN | 2 | 1 | -0.0338933022774001 | 1.41358031409811 |
| 3116 | ZNF718 | 16 | 1 | -3.75690148790248 | 2.11132492202352 |
| 3117 | ZNF732 | 19 | 1 | -5.02608703096278 | 1.52848924773763 |
| 3118 | ZNF141 | 9 | 1 | 1.66071344892614 | 2.98140944617819 |
| 3119 | ZNF721 | 2 | 1 | -0.00816860814459519 | 2.21528209823202 |
| 3120 | PIGG | 2 | 1 | 0.177071168824361 | 2.26995672362875 |
| 3121 | AC116565.1 | 10 | 1 | 4.88718439619176 | -0.982253692271006 |
| 3122 | AC116565.2 | 10 | 1 | 4.94048525373571 | -0.969671121956599 |
| 3123 | ATP5ME | 6 | 1 | 0.542902663155721 | -2.3841228328364 |
| 3124 | MYL5 | 3 | 1 | -2.60586999376185 | -0.566877766670478 |
| 3125 | PCGF3 | 2 | 1 | -0.0326044412410994 | 1.9937988676412 |
| 3126 | AC139887.4 | 1 | 1 | 1.25489903967016 | 1.71217598098348 |
| 3127 | AC139887.2 | 2 | 1 | -0.0809138270176192 | 1.91356505530905 |
| 3128 | CPLX1 | 10 | 1 | 4.60145737211339 | -1.03984443766047 |
| 3129 | GAK | 12 | 1 | 2.27025176565282 | 2.99877490180563 |
| 3130 | TMEM175 | 10 | 1 | 4.41619755308263 | -1.30310897451807 |
| 3131 | FGFRL1 | 4 | 1 | -5.06248258073695 | -0.913949199179006 |
| 3132 | RNF212 | 5 | 1 | 2.60622788946264 | -0.0987008057253481 |
| 3133 | SPON2 | 7 | 1 | -1.99119435270198 | -3.46253524643351 |
| 3134 | CTBP1-AS | 9 | 1 | 1.27218903581731 | 2.6467164911611 |
| 3135 | CTBP1 | 1 | 1 | 2.63394714872472 | 0.435461775241601 |
| 3136 | CTBP1-DT | 12 | 1 | 3.25063587705724 | 2.7791341699941 |
| 3137 | MAEA | 8 | 1 | -0.659728810385539 | -0.136155053200019 |
| 3138 | UVSSA | 12 | 1 | 3.1359119562351 | 2.94472254890035 |
| 3139 | NKX1-1 | 15 | 1 | 5.8860533384525 | 0.297699705539453 |
| 3140 | AC147067.1 | 6 | 1 | 0.863127246304677 | -2.99009333473612 |
| 3141 | SLBP | 5 | 1 | 2.68390776197545 | -0.719550087274802 |
| 3142 | TMEM129 | 8 | 1 | -0.913549170092418 | 0.0967200555188535 |
| 3143 | TACC3 | 16 | 1 | -4.41015218217738 | 3.16408909934591 |
| 3144 | FGFR3 | 14 | 1 | -6.90363954027064 | -0.787628389121783 |
| 3145 | LETM1 | 1 | 1 | 4.10961271803014 | 1.55037082809042 |
| 3146 | NSD2 | 2 | 1 | -0.826556489065959 | 2.72626246589254 |
| 3147 | NELFA | 2 | 1 | 1.39105965177648 | 0.967820064006555 |
| 3148 | C4orf48 | 10 | 1 | 3.22218038122289 | -1.96011494022776 |

| 3149 | NAT8L | 5 | 1 | 3.90716053526037 | 0.383207456050622 |
| --- | --- | --- | --- | --- | --- |
| 3150 | POLN | 1 | 1 | 2.82487870733373 | 0.673099652705896 |
| 3151 | HAUS3 | 1 | 1 | 3.47624541799657 | 2.21004642623495 |
| 3152 | MXD4 | 7 | 1 | -3.10734365899928 | -2.51453624588419 |
| 3153 | RNF4 | 2 | 1 | -1.16948251445658 | 2.54844583648275 |
| 3154 | FAM193A | 12 | 1 | 2.11237217466466 | 3.02551855224203 |
| 3155 | TNIP2 | 11 | 1 | -3.42049835641749 | -2.08354245048929 |
| 3156 | SH3BP2 | 1 | 1 | 3.90632177869909 | 1.54557980674337 |
| 3157 | ADD1 | 12 | 1 | 2.9647910742008 | 2.41833581107687 |
| 3158 | MFSD10 | 6 | 1 | 1.00829841177099 | -1.87244872671534 |
| 3159 | NOP14 | 11 | 1 | -3.55043672044642 | -0.909274279298079 |
| 3160 | GRK4 | 5 | 1 | 3.90399481336706 | -0.112118049682868 |
| 3161 | HTT | 12 | 1 | 2.83707977811925 | 1.97855962890218 |
| 3162 | MSANTD1 | 18 | 1 | 5.76586844007604 | -2.00386558395792 |
| 3163 | RGS12 | 12 | 1 | 2.7687552122318 | 2.78991331237386 |
| 3164 | DOK7 | 7 | 1 | -2.9921614976681 | -3.91395030838419 |
| 3165 | LRPAP1 | 11 | 1 | -2.6833395810879 | -1.51329283338953 |
| 3166 | ADRA2C | 13 | 1 | -0.142623238459899 | -3.19614230019022 |
| 3167 | TMEM128 | 13 | 1 | -1.24541281183131 | -1.65222977024485 |
| 3168 | LYAR | 19 | 1 | -3.98658774812586 | 0.745099917827356 |
| 3169 | NSG1 | 10 | 1 | 4.34734465162389 | -1.390029593529 |
| 3170 | STX18 | 17 | 1 | -0.326502055124118 | 0.0606996573788999 |
| 3171 | STK32B | 18 | 1 | 5.53765131513707 | -1.7248809776919 |
| 3172 | EVC | 14 | 1 | -5.46581386049159 | -0.916753725306881 |
| 3173 | CRMP1 | 10 | 1 | 3.86235834638707 | -1.09305800420214 |
| 3174 | JAKMIP1 | 18 | 1 | 5.34548069517247 | -0.457425191225302 |
| 3175 | WFS1 | 8 | 1 | -1.60739658792384 | 1.1545289911611 |
| 3176 | PPP2R2C | 5 | 1 | 4.28923012296788 | -0.261074586929572 |
| 3177 | MAN2B2 | 1 | 1 | 2.56219436208837 | 0.646326915202844 |
| 3178 | MRFAP1 | 13 | 1 | -1.54557977636225 | -0.851980685414565 |
| 3179 | LINC02482 | 6 | 1 | 1.58002711813085 | -1.47012756687571 |
| 3180 | AC093323.1 | 2 | 1 | -0.0915142597711821 | 2.34551848548483 |
| 3181 | MRFAP1L1 | 12 | 1 | 2.99786831419103 | 2.97932209151815 |
| 3182 | BLOC1S4 | 13 | 1 | -2.00544117410548 | -1.7014594517367 |
| 3183 | KIAA0232 | 1 | 1 | 3.54120112936132 | 2.28917612212728 |
| 3184 | TBC1D14 | 9 | 1 | 1.63345099966161 | 2.59004511016439 |
| 3185 | CCDC96 | 2 | 1 | -0.680599376753642 | 1.02732159274648 |
| 3186 | TADA2B | 9 | 1 | 1.81431271116369 | 2.9972385324819 |
| 3187 | GRPEL1 | 8 | 1 | -1.64913176019557 | 0.379602686343896 |
| 3188 | LINC02447 | 7 | 1 | -3.38057707269557 | -2.47840438705851 |
| 3189 | SORCS2 | 7 | 1 | -3.03739403207667 | -3.38199625832011 |
| 3190 | PSAPL1 | 11 | 1 | -3.92829535920985 | -2.13081608635355 |
| 3191 | AFAP1 | 5 | 1 | 4.44983555357091 | 0.64078165668081 |
| 3192 | ABLIM2 | 15 | 1 | 4.66094185392492 | 0.0431404270512937 |
| 3193 | AC104825.1 | 1 | 1 | 3.03639079610936 | 1.3324538626058 |
| 3194 | TRMT44 | 2 | 1 | 0.126284107371495 | 2.16894735473226 |
| 3195 | AC105345.1 | 1 | 1 | 4.52269674818151 | 1.7845794119222 |
| 3196 | WDR1 | 8 | 1 | -0.370634697572066 | -0.450465097250235 |
| 3197 | ZNF518B | 8 | 1 | -1.36340437848933 | 1.68197383063863 |
| 3198 | CLNK | 4 | 1 | -4.66920255144007 | -1.60140357834269 |
| 3199 | HS3ST1 | 6 | 1 | 2.27424408475988 | -2.1098981938975 |
| 3200 | AC007370.2 | 12 | 1 | 2.14153386633031 | 2.55156101363729 |
| 3201 | RAB28 | 2 | 1 | -0.370967783228232 | 0.388819948611963 |
| 3202 | BOD1L1 | 12 | 1 | 2.38389636556737 | 2.33928383964132 |
| 3203 | CPEB2 | 1 | 1 | 3.67029549162023 | 1.16585125106405 |
| 3204 | C1QTNF7 | 1 | 1 | 1.56505812685125 | 1.35993279594015 |
| 3205 | CC2D2A | 8 | 1 | -1.25406263788111 | 1.06450893062185 |
| 3206 | FBXL5 | 12 | 1 | 2.98835207502477 | 2.33096112388204 |
| 3207 | FAM200B | 10 | 1 | 3.22534991781347 | -1.58354918581415 |
| 3208 | PROM1 | 19 | 1 | -4.87315534074671 | 0.917298332629907 |
| 3209 | TAPT1 | 1 | 1 | 3.87185122053258 | 2.31064452308248 |
| 3210 | LDB2 | 18 | 1 | 5.51512791196935 | -1.97917996269633 |
| 3211 | QDPR | 14 | 1 | -5.53748248536952 | -0.0726867757456423 |
| 3212 | LAP3 | 11 | 1 | -3.75429819543727 | -1.62105075937678 |
| 3213 | MED28 | 11 | 1 | -2.97138427217372 | -1.32641957622935 |
| 3214 | FAM184B | 9 | 1 | 1.21824993173711 | 2.84095729964803 |
| 3215 | DCAF16 | 3 | 1 | -2.60284779985316 | 0.842081681667077 |

| 3216 | NCAPG | 16 | 1 | -4.61686299760707 | 3.01058878081869 |
| --- | --- | --- | --- | --- | --- |
| 3217 | LCORL | 12 | 1 | 2.82229544202916 | 2.73957480567526 |
| 3218 | SLIT2 | 4 | 1 | -4.18968890626796 | -0.852314285160792 |
| 3219 | PACRGL | 3 | 1 | -2.22146926839717 | 0.413268820224512 |
| 3220 | KCNIP4 | 18 | 1 | 5.24361874143712 | -1.42420624119211 |
| 3221 | KCNIP4-IT1 | 18 | 1 | 5.30379988233678 | -1.94496284347941 |
| 3222 | ADGRA3 | 3 | 1 | -2.55452822168238 | 0.725540415225732 |
| 3223 | DHX15 | 3 | 1 | -2.69084738214381 | 0.75764538425039 |
| 3224 | CCDC149 | 12 | 1 | 2.68201448003881 | 2.87927807944845 |
| 3225 | LGI2 | 10 | 1 | 4.80628944913976 | -1.58631788116862 |
| 3226 | SEPSECS | 3 | 1 | -2.97255300004847 | -0.36388610226084 |
| 3227 | SEPSECS-AS1 | 8 | 1 | -2.79934094865687 | 2.20467104095052 |
| 3228 | PI4K2B | 19 | 1 | -5.00748895128138 | 1.41090311187338 |
| 3229 | ZCCHC4 | 8 | 1 | -2.06574831922419 | 1.04234911578726 |
| 3230 | ANAPC4 | 8 | 1 | -1.47897575815089 | -0.133975847782385 |
| 3231 | SEL1L3 | 6 | 1 | 1.33586551229589 | -2.72975395542551 |
| 3232 | SMIM20 | 11 | 1 | -3.06959079225428 | -1.49290840250422 |
| 3233 | RBPJ | 2 | 1 | -0.755501851633861 | 1.23330916541647 |
| 3234 | TBC1D19 | 5 | 1 | 3.36078692953222 | 0.203228131709802 |
| 3235 | STIM2 | 1 | 1 | 4.76116587202184 | 1.05408717769216 |
| 3236 | PCDH7 | 18 | 1 | 5.6158330587589 | -0.714268236579192 |
| 3237 | ARAP2 | 14 | 1 | -6.63949607332118 | -0.33874086958338 |
| 3238 | NWD2 | 7 | 1 | -2.72607825715907 | -3.92221580368449 |
| 3239 | RELL1 | 4 | 1 | -4.23354862649806 | -0.500115587534201 |
| 3240 | PGM2 | 13 | 1 | -2.00994406659968 | -2.39195094925333 |
| 3241 | TBC1D1 | 19 | 1 | -4.14577744920619 | 1.1837972559316 |
| 3242 | KLF3 | 15 | 1 | 4.79888989011876 | -0.218953355373633 |
| 3243 | TLR10 | 20 | 1 | 3.41012002508275 | -3.82743750435282 |
| 3244 | FAM114A1 | 14 | 1 | -5.48789571245082 | 0.411311284480798 |
| 3245 | KLHL5 | 18 | 1 | 5.5818188337528 | -2.18271194321085 |
| 3246 | WDR19 | 2 | 1 | 0.906142130299733 | 1.62701191085409 |
| 3247 | RFC1 | 19 | 1 | -4.46421550233729 | 1.60449899810384 |
| 3248 | RPL9 | 21 | 1 | -1.11795477588542 | -4.63470397812296 |
| 3249 | LIAS | 2 | 1 | -0.0385873617685576 | 0.889053121982324 |
| 3250 | UGDH | 3 | 1 | -2.84386418779261 | 0.64291538375448 |
| 3251 | UGDH-AS1 | 12 | 1 | 2.64431430379979 | 2.37544645446371 |
| 3252 | SMIM14 | 15 | 1 | 5.12008883039586 | 0.543021456180322 |
| 3253 | UBE2K | 1 | 1 | 3.65983368436925 | 2.32067598479818 |
| 3254 | PDS5A | 2 | 1 | -1.40163372476466 | 1.98856176513265 |
| 3255 | N4BP2 | 1 | 1 | 3.76120258848302 | 2.08241094726156 |
| 3256 | RHOH | 7 | 1 | -3.01564143617518 | -2.36466894966532 |
| 3257 | LINC02265 | 18 | 1 | 5.35090996305578 | -1.8670284591334 |
| 3258 | RBM47 | 14 | 1 | -5.83691476304896 | 0.163920418201196 |
| 3259 | NSUN7 | 16 | 1 | -4.30621598680384 | 2.61317242759298 |
| 3260 | APBB2 | 5 | 1 | 3.20968534032934 | -0.253288670601142 |
| 3261 | UCHL1 | 10 | 1 | 4.38720156232946 | -1.05876068693568 |
| 3262 | LIMCH1 | 14 | 1 | -6.65039847810633 | -0.491251929821265 |
| 3263 | TMEM33 | 12 | 1 | 2.17888118307226 | 2.25663007873129 |
| 3264 | SLC30A9 | 2 | 1 | -0.688313082054927 | 2.24441208022665 |
| 3265 | BEND4 | 20 | 1 | 3.73405124227636 | -3.35843883377482 |
| 3266 | SHISA3 | 14 | 1 | -6.86965630967982 | -0.780349902035487 |
| 3267 | ATP8A1 | 1 | 1 | 4.30039669553869 | 1.52678455489706 |
| 3268 | GRXCR1 | 18 | 1 | 6.30869986097448 | -2.51226054054667 |
| 3269 | KCTD8 | 7 | 1 | -2.88534902055628 | -3.86825667244364 |
| 3270 | GUF1 | 8 | 1 | -1.20684801061518 | 1.62728108542989 |
| 3271 | GNPDA2 | 12 | 1 | 2.84426380674474 | 2.91108860152792 |
| 3272 | AC096586.2 | 5 | 1 | 3.78297592680089 | -0.354893132271063 |
| 3273 | GABRA2 | 15 | 1 | 5.66526915113561 | 0.160648600040185 |
| 3274 | GABRB1 | 15 | 1 | 5.70511414091222 | 0.014762178836572 |
| 3275 | COMMD8 | 5 | 1 | 2.44813539068334 | -0.328336103977454 |
| 3276 | ATP10D | 8 | 1 | -1.22555701454051 | 0.967172757564294 |
| 3277 | NFXL1 | 12 | 1 | 2.93362165968053 | 2.91816844123434 |
| 3278 | NIPAL1 | 19 | 1 | -4.12195132692225 | 0.612277404247033 |
| 3279 | SLAIN2 | 9 | 1 | 1.51034857313268 | 2.51580490249227 |
| 3280 | SLC10A4 | 18 | 1 | 6.06304384748571 | -2.11495946270396 |
| 3281 | FRYL | 2 | 1 | -0.851756856039836 | 1.51028193610738 |
| 3282 | OCIAD1 | 6 | 1 | -0.243272181794717 | -0.753633081139815 |

| 3283 | OCIAD2 | 10 | 1 | 3.38894273321264 | -1.62821517807413 |
| --- | --- | --- | --- | --- | --- |
| 3284 | DCUN1D4 | 12 | 1 | 3.02981092016332 | 2.6055182375295 |
| 3285 | SGCB | 2 | 1 | 1.11253906767003 | 1.30940212386678 |
| 3286 | SPATA18 | 7 | 1 | -3.01612852533229 | -2.54426191669871 |
| 3287 | USP46 | 9 | 1 | 1.4763249305927 | 2.47789634841512 |
| 3288 | USP46-AS1 | 11 | 1 | -3.93547104318507 | -2.18331800323893 |
| 3289 | RASL11B | 2 | 1 | -0.0945679100311537 | 0.784538523135888 |
| 3290 | FIP1L1 | 8 | 1 | -1.15693329294093 | 0.529212609706628 |
| 3291 | LNX1 | 5 | 1 | 3.32690884153478 | -0.385364397587073 |
| 3292 | CHIC2 | 8 | 1 | -0.474431604341342 | -0.123057886184943 |
| 3293 | GSX2 | 4 | 1 | -5.29054568727381 | -1.68666945320536 |
| 3294 | PDGFRA | 14 | 1 | -5.46447823961146 | -0.0442399225847842 |
| 3295 | KIT | 20 | 1 | 3.41296745817296 | -3.70396171432902 |
| 3296 | KDR | 7 | 1 | -2.16368876417048 | -3.11636124473978 |
| 3297 | SRD5A3 | 19 | 1 | -4.27470420320399 | 1.4960862554891 |
| 3298 | TMEM165 | 11 | 1 | -3.42899154146083 | -1.31230457049776 |
| 3299 | CLOCK | 9 | 1 | 1.3759853986942 | 3.10586442130636 |
| 3300 | NMU | 16 | 1 | -4.35832856614955 | 2.43888534682821 |
| 3301 | EXOC1 | 1 | 1 | 3.29395963232152 | 1.18564738410543 |
| 3302 | CEP135 | 16 | 1 | -4.36971972902186 | 2.41805471557211 |
| 3303 | KIAA1211 | 2 | 1 | 0.871020450993703 | 2.06427349227499 |
| 3304 | AASDH | 2 | 1 | -0.441241637066676 | 1.17461051124166 |
| 3305 | AC068620.1 | 11 | 1 | -4.10432504136927 | -1.38257254582812 |
| 3306 | PPAT | 3 | 1 | -2.93690274675257 | 0.430394307552087 |
| 3307 | PAICS | 11 | 1 | -3.80710671861537 | -0.993740252377284 |
| 3308 | SRP72 | 3 | 1 | -2.48056052644618 | -0.237934454502356 |
| 3309 | ARL9 | 11 | 1 | -3.79553531129725 | -2.23443148952891 |
| 3310 | REST | 4 | 1 | -4.50772593934901 | 0.143401161609399 |
| 3311 | NOA1 | 13 | 1 | -1.19143460710414 | -0.911404173048031 |
| 3312 | POLR2B | 2 | 1 | -0.0367773475206633 | 1.46143473762106 |
| 3313 | IGFBP7 | 14 | 1 | -6.03407309968837 | 0.168512598453271 |
| 3314 | IGFBP7-AS1 | 6 | 1 | 1.74172224084966 | -1.37541104775835 |
| 3315 | ADGRL3 | 1 | 1 | 4.56380201856725 | 1.1283945001943 |
| 3316 | AC020741.1 | 5 | 1 | 3.34902907888524 | 0.166771546779382 |
| 3317 | EPHA5 | 18 | 1 | 5.04166389982335 | -1.72712956291605 |
| 3318 | EPHA5-AS1 | 10 | 1 | 4.12842394392125 | -1.29174239856173 |
| 3319 | AC104806.2 | 12 | 1 | 2.21696425954931 | 2.61457718986105 |
| 3320 | CENPC | 16 | 1 | -3.98003362138636 | 2.67017091888021 |
| 3321 | UBA6 | 9 | 1 | 1.29501868764989 | 2.31279911178182 |
| 3322 | YTHDC1 | 8 | 1 | -1.34854052980311 | 1.80612076896261 |
| 3323 | AMBN | 4 | 1 | -5.36432813127406 | -1.79701898914744 |
| 3324 | UTP3 | 2 | 1 | -0.540550396040752 | 1.31199754851888 |
| 3325 | RUFY3 | 5 | 1 | 3.92385031263463 | 0.025793627677667 |
| 3326 | GRSF1 | 8 | 1 | -1.81723581273921 | 0.627206579623926 |
| 3327 | MOB1B | 2 | 1 | -0.835660144881084 | 2.25167550223898 |
| 3328 | DCK | 5 | 1 | 2.43404246847265 | -0.170099421562445 |
| 3329 | SLC4A4 | 14 | 1 | -6.41778539140589 | -0.122045918525946 |
| 3330 | NPFFR2 | 10 | 1 | 4.52586342374913 | -2.36684296947886 |
| 3331 | ADAMTS3 | 14 | 1 | -6.55674575288661 | -0.246041163028968 |
| 3332 | COX18 | 2 | 1 | -0.669817999199702 | 1.82316817420553 |
| 3333 | ANKRD17 | 9 | 1 | 1.76292277853124 | 3.01127876418661 |
| 3334 | MTHFD2L | 4 | 1 | -3.86722444017298 | -0.303652807297003 |
| 3335 | AC093677.2 | 4 | 1 | -3.96776293237574 | -0.00576697212625754 |
| 3336 | PARM1 | 6 | 1 | 1.25627304594152 | -2.9528313718455 |
| 3337 | LINC02562 | 11 | 1 | -3.80223129709132 | -1.71003876549174 |
| 3338 | RCHY1 | 10 | 1 | 3.73700834791295 | -1.00426963639427 |
| 3339 | THAP6 | 9 | 1 | 1.54235328714483 | 2.9527923025472 |
| 3340 | CDKL2 | 10 | 1 | 2.93572212736242 | -1.49125276428629 |
| 3341 | G3BP2 | 5 | 1 | 3.61073948423497 | -0.885783622199488 |
| 3342 | USO1 | 11 | 1 | -3.06718991716273 | -2.13285349232126 |
| 3343 | SDAD1 | 3 | 1 | -1.79306696374781 | 0.266004816470849 |
| 3344 | ART3 | 6 | 1 | 1.30295123140447 | -2.86963377815653 |
| 3345 | NUP54 | 3 | 1 | -3.46874235589869 | 0.879914299426782 |
| 3346 | SCARB2 | 1 | 1 | 3.54806710760228 | 1.30553950446676 |
| 3347 | SHROOM3 | 4 | 1 | -4.43491767366297 | -0.577684327186835 |
| 3348 | AC107072.2 | 4 | 1 | -4.26187203843959 | -0.786301202298892 |
| 3349 | Sep-11 | 3 | 1 | -3.01011227090724 | 0.348433748660791 |

| 3350 | CCNI | 10 | 1 | 3.54134465734594 | -1.78326283317972 |
| --- | --- | --- | --- | --- | --- |
| 3351 | CCNG2 | 3 | 1 | -2.71175073106654 | 0.534698263583887 |
| 3352 | CNOT6L | 1 | 1 | 4.3459823278629 | 1.68455042022299 |
| 3353 | MRPL1 | 3 | 1 | -2.64261697252162 | -0.242330833496344 |
| 3354 | FRAS1 | 12 | 1 | 2.85977007429235 | 3.09746064323019 |
| 3355 | ANXA3 | 14 | 1 | -5.56906388719447 | 0.172577277599084 |
| 3356 | AC098818.2 | 16 | 1 | -4.40851186235316 | 2.50050081389974 |
| 3357 | BMP2K | 9 | 1 | -0.475354939893558 | 2.966954842983 |
| 3358 | PAQR3 | 1 | 1 | 2.38107229749791 | 1.04553522246908 |
| 3359 | LINC01088 | 7 | 1 | -3.67099021394618 | -3.43382678848673 |
| 3360 | ANTXR2 | 11 | 1 | -3.88626121003993 | -2.36530528885294 |
| 3361 | PRDM8 | 4 | 1 | -4.99916099031337 | -2.44070087295939 |
| 3362 | CFAP299 | 11 | 1 | -3.42760489900477 | -2.30052910667826 |
| 3363 | BMP3 | 15 | 1 | 5.84213615934484 | 0.776730195461023 |
| 3364 | PRKG2 | 10 | 1 | 4.97590805570714 | -1.47091065269877 |
| 3365 | RASGEF1B | 7 | 1 | -3.32718537767298 | -3.74267040115763 |
| 3366 | AC124016.2 | 10 | 1 | 3.7873590139591 | -1.41633008343149 |
| 3367 | HNRNPD | 3 | 1 | -2.93510364015467 | 0.612706438480127 |
| 3368 | AC124016.1 | 10 | 1 | 3.16772057096593 | -1.29343788248469 |
| 3369 | HNRNPDL | 13 | 1 | -1.48814533670314 | -2.53400228840281 |
| 3370 | ENOPH1 | 5 | 1 | 3.94266535322301 | -0.757466866792929 |
| 3371 | TMEM150C | 10 | 1 | 4.76208378355138 | -0.598468317808402 |
| 3372 | SCD5 | 18 | 1 | 5.23586727659337 | -0.42822103125025 |
| 3373 | SEC31A | 8 | 1 | -1.33994840104945 | 1.08597554343771 |
| 3374 | THAP9-AS1 | 2 | 1 | 0.716574147149251 | 1.51943530219625 |
| 3375 | THAP9 | 16 | 1 | -4.09016512353785 | 1.92489780562948 |
| 3376 | LIN54 | 9 | 1 | -0.187639914647414 | 3.13426007407736 |
| 3377 | COPS4 | 13 | 1 | -0.505919084147288 | -0.841462477268469 |
| 3378 | COQ2 | 19 | 1 | -4.28392766435511 | 1.42884911674093 |
| 3379 | HPSE | 19 | 1 | -4.15979860742457 | 0.910741344867456 |
| 3380 | MRPS18C | 13 | 1 | -0.458220467165782 | -1.79731343609263 |
| 3381 | ABRAXAS1 | 11 | 1 | -3.48546694238551 | -2.35409401279856 |
| 3382 | NKX6-1 | 20 | 1 | 3.47783900777929 | -3.64870129448344 |
| 3383 | WDFY3 | 12 | 1 | 2.15056730787389 | 2.52047051566671 |
| 3384 | MAPK10 | 15 | 1 | 5.1389215139591 | 0.798901812015283 |
| 3385 | PTPN13 | 4 | 1 | -4.94472573717005 | -0.49297358018328 |
| 3386 | AFF1 | 1 | 1 | 1.59914292375676 | 1.35055245536398 |
| 3387 | KLHL8 | 9 | 1 | 0.746051206990407 | 3.01666988509725 |
| 3388 | HSD17B11 | 11 | 1 | -3.52719996889003 | -1.20707879883219 |
| 3389 | NUDT9 | 8 | 1 | -1.40714178998835 | 0.259186521945703 |
| 3390 | SPARCL1 | 14 | 1 | -6.12643931825526 | 0.0371400870663999 |
| 3391 | SPP1 | 15 | 1 | 6.03442646543615 | 0.591893450198877 |
| 3392 | PKD2 | 8 | 1 | -1.58301125486262 | 1.92574657577108 |
| 3393 | PPM1K | 18 | 1 | 5.29223228971593 | -0.785771406413806 |
| 3394 | HERC5 | 19 | 1 | -5.33203862626918 | 0.954611913142907 |
| 3395 | PYURF | 13 | 1 | -0.693635598019435 | -1.91880248409678 |
| 3396 | HERC3 | 18 | 1 | 5.12135483305089 | -0.740154518903983 |
| 3397 | NAP1L5 | 5 | 1 | 4.25000216047399 | -0.626050545992148 |
| 3398 | FAM13A-AS1 | 1 | 1 | 2.31388522665136 | 1.06903256553243 |
| 3399 | FAM13A | 7 | 1 | -3.27555106599696 | -2.55979715210367 |
| 3400 | TIGD2 | 7 | 1 | -2.66679476220973 | -3.56897483688761 |
| 3401 | GPRIN3 | 7 | 1 | -2.68277166803248 | -3.98712240082194 |
| 3402 | AC097478.1 | 13 | 1 | -0.373739063934161 | -3.28172360283304 |
| 3403 | SNCA | 10 | 1 | 3.6442434934818 | -1.13556025844981 |
| 3404 | MMRN1 | 7 | 1 | -3.32416270692713 | -2.45857392174174 |
| 3405 | CCSER1 | 5 | 1 | 4.09365250150792 | 0.317738787112939 |
| 3406 | GRID2 | 18 | 1 | 5.56244875471227 | -0.964539586724532 |
| 3407 | SMARCAD1 | 1 | 1 | 3.26780559103124 | 2.35190691131185 |
| 3408 | PDLIM5 | 14 | 1 | -5.68334601838954 | -0.657622053446067 |
| 3409 | BMPR1B | 14 | 1 | -6.80010484178431 | -0.516345097841513 |
| 3410 | UNC5C | 15 | 1 | 5.52669168989293 | 0.614186541019189 |
| 3411 | AC106881.1 | 15 | 1 | 5.66052986662023 | 0.212777630267846 |
| 3412 | RAP1GDS1 | 5 | 1 | 3.09160090963476 | 0.212579027591455 |
| 3413 | TSPAN5 | 5 | 1 | 3.94459129850499 | -0.0632839046137453 |
| 3414 | AC114811.2 | 12 | 1 | 3.13995148222081 | 2.83595337050985 |
| 3415 | EIF4E | 2 | 1 | 1.48529221098058 | 0.811222926555383 |
| 3416 | METAP1 | 2 | 1 | 0.612732425137685 | 1.1579183496816 |

| 3417 | ADH5 | 11 | 1 | -3.02099131067164 | -1.71136663776804 |
| --- | --- | --- | --- | --- | --- |
| 3418 | AP002026.1 | 11 | 1 | -3.52149437387355 | -2.20163296085764 |
| 3419 | ADH4 | 11 | 1 | -3.54010938127406 | -1.09910254103113 |
| 3420 | ADH1A | 11 | 1 | -3.55751179178126 | -1.97480235916544 |
| 3421 | TRMT10A | 4 | 1 | -3.86539171655543 | -0.188716693939459 |
| 3422 | MTTP | 14 | 1 | -6.81540463884242 | -0.509607716621649 |
| 3423 | LAMTOR3 | 9 | 1 | 0.525172188683675 | 2.66158188956808 |
| 3424 | DNAJB14 | 12 | 1 | 2.85779072324865 | 2.09454263824056 |
| 3425 | H2AFZ | 19 | 1 | -3.97302673776515 | 1.31772221702169 |
| 3426 | DDIT4L | 4 | 1 | -4.59241937120326 | -0.163147314609778 |
| 3427 | PPP3CA | 5 | 1 | 3.80466677229039 | -0.879353649141085 |
| 3428 | AP001816.1 | 10 | 1 | 3.3670320657932 | -2.05096755844523 |
| 3429 | SLC39A8 | 19 | 1 | -5.06150029619105 | 1.23037971633505 |
| 3430 | AC098487.1 | 19 | 1 | -4.82882092912562 | 1.45704020637106 |
| 3431 | NFKB1 | 14 | 1 | -5.25429700334437 | 0.518041626392114 |
| 3432 | UBE2D3 | 3 | 1 | -2.4177260251797 | 0.0940663971288084 |
| 3433 | CISD2 | 10 | 1 | 3.20949603597753 | -1.20077378970553 |
| 3434 | SLC9B1 | 17 | 1 | 1.01781763116948 | -0.0623693309443117 |
| 3435 | SLC9B2 | 18 | 1 | 5.02192570249669 | -0.667678727926505 |
| 3436 | BDH2 | 11 | 1 | -3.7995185704983 | -1.71540640217234 |
| 3437 | CENPE | 16 | 1 | -4.33010791261561 | 3.12017430442404 |
| 3438 | CXXC4 | 5 | 1 | 4.0702746061527 | -0.63164196831156 |
| 3439 | AC004069.1 | 4 | 1 | -4.03105042894252 | -0.721471264185202 |
| 3440 | TET2 | 12 | 1 | 2.12909866850011 | 2.99476803916525 |
| 3441 | PPA2 | 3 | 1 | -2.51576136072047 | -1.37583593946863 |
| 3442 | GSTCD | 8 | 1 | -2.64523457010157 | 2.16275133269857 |
| 3443 | TBCK | 12 | 1 | 2.12040115873449 | 2.89903701919149 |
| 3444 | AIMP1 | 3 | 1 | -3.17307828386195 | -0.789203292848361 |
| 3445 | PAPSS1 | 10 | 1 | 3.43537904302709 | -1.57929317575861 |
| 3446 | HADH | 19 | 1 | -4.38131545503505 | 1.01598335879873 |
| 3447 | LEF1 | 4 | 1 | -4.10837076623805 | -0.0398888431208254 |
| 3448 | RPL34 | 21 | 1 | -0.621362939671351 | -4.15056882721354 |
| 3449 | OSTC | 11 | 1 | -3.16442821939357 | -1.53973601681162 |
| 3450 | COL25A1 | 18 | 1 | 5.73385883848302 | -1.69580893141199 |
| 3451 | SEC24B-AS1 | 17 | 1 | 0.333717420502828 | 0.657286898074853 |
| 3452 | SEC24B | 9 | 1 | 1.02116741220586 | 2.91676534789633 |
| 3453 | MCUB | 13 | 1 | -0.837697431639506 | -2.65004573685099 |
| 3454 | CASP6 | 11 | 1 | -3.15598510225184 | -1.88167892319132 |
| 3455 | PLA2G12A | 1 | 1 | 3.75378777067296 | 0.540411130367029 |
| 3456 | GAR1 | 4 | 1 | -3.40969871003993 | -0.251115008415473 |
| 3457 | ELOVL6 | 5 | 1 | 4.10302902738683 | -0.198083563865912 |
| 3458 | FAM241A | 5 | 1 | 3.65221597234838 | -0.980505748810065 |
| 3459 | AC109347.1 | 4 | 1 | -3.72146604974635 | -0.267259105266822 |
| 3460 | AP1AR | 12 | 1 | 2.06670810262792 | 2.35066713470052 |
| 3461 | TIFA | 4 | 1 | -4.0361604543484 | -0.613910808386099 |
| 3462 | NEUROG2 | 7 | 1 | -3.23068450410731 | -3.70099507194926 |
| 3463 | AC023886.1 | 6 | 1 | 1.50325502435796 | -2.09509621483256 |
| 3464 | ZGRF1 | 16 | 1 | -4.08302496393092 | 2.14335359710287 |
| 3465 | LARP7 | 4 | 1 | -3.44383667428858 | -0.157467170776618 |
| 3466 | ANK2 | 1 | 1 | 4.10717608015172 | 0.951365963397729 |
| 3467 | AC017007.5 | 7 | 1 | -3.70544122178919 | -2.68775628429819 |
| 3468 | CAMK2D | 15 | 1 | 5.04248405973546 | 0.683219925342309 |
| 3469 | UGT8 | 1 | 1 | 1.37364496271245 | 1.3186541952474 |
| 3470 | NDST4 | 15 | 1 | 5.91674877683751 | 0.836974636493432 |
| 3471 | TRAM1L1 | 5 | 1 | 4.38046862165563 | -0.366803213180792 |
| 3472 | NDST3 | 15 | 1 | 4.68310429136388 | -0.0757853828089358 |
| 3473 | SNHG8 | 13 | 1 | -1.31771884878047 | -0.927465195925725 |
| 3474 | METTL14 | 9 | 1 | 1.08824492971532 | 3.01255191939901 |
| 3475 | SEC24D | 14 | 1 | -5.23406146486171 | 0.38461972850393 |
| 3476 | USP53 | 15 | 1 | 5.99934793989293 | 0.696237579761255 |
| 3477 | C4orf3 | 11 | 1 | -3.01326797922023 | -1.67420665842463 |
| 3478 | PDE5A | 8 | 1 | -1.85384009797938 | 1.6698173441274 |
| 3479 | MAD2L1 | 16 | 1 | -4.59158585985072 | 2.75474275725912 |
| 3480 | AC108866.1 | 3 | 1 | -2.64142106492884 | -0.87486743477155 |
| 3481 | PRDM5 | 14 | 1 | -5.27233479936488 | 0.0821132816655515 |
| 3482 | NDNF | 14 | 1 | -6.92667315919764 | -0.6914054892676 |
| 3483 | TNIP3 | 15 | 1 | 5.63424707929723 | 0.351213113246667 |

| 3484 | ANXA5 | 11 | 1 | -3.66254161317713 | -0.958927921177638 |
| --- | --- | --- | --- | --- | --- |
| 3485 | EXOSC9 | 16 | 1 | -4.39652608354457 | 1.90535582679342 |
| 3486 | CCNA2 | 16 | 1 | -4.52871631105311 | 3.19799222129415 |
| 3487 | BBS7 | 13 | 1 | -0.46500195582278 | -2.36972068172861 |
| 3488 | TRPC3 | 15 | 1 | 5.66601301710241 | 0.0626792468411802 |
| 3489 | KIAA1109 | 1 | 1 | 3.39214827100866 | 1.87300075667928 |
| 3490 | BBS12 | 6 | 1 | 1.69418014566533 | -0.910595927545619 |
| 3491 | FGF2 | 3 | 1 | -3.21923826654322 | 0.272382632671106 |
| 3492 | NUDT6 | 16 | 1 | -4.42848466356166 | 2.2618230738027 |
| 3493 | SPATA5 | 16 | 1 | -4.43744634111293 | 2.35229601043295 |
| 3494 | SPRY1 | 11 | 1 | -3.68604539354212 | -2.2155060611384 |
| 3495 | LINC01091 | 6 | 1 | 1.911641374036 | -2.60274992805887 |
| 3496 | ANKRD50 | 1 | 1 | 3.22844220678441 | 1.66263379233907 |
| 3497 | FAT4 | 18 | 1 | 5.77184416334264 | -2.35436819416453 |
| 3498 | AC093772.1 | 14 | 1 | -6.96651385743983 | -0.774785145344031 |
| 3499 | INTU | 8 | 1 | -1.35655973871119 | 1.78175605910848 |
| 3500 | HSPA4L | 9 | 1 | 0.974448099538014 | 3.13906897681783 |
| 3501 | PLK4 | 16 | 1 | -4.78798984010585 | 2.45085324424337 |
| 3502 | MFSD8 | 9 | 1 | 1.45202256719701 | 3.00505055564474 |
| 3503 | ABHD18 | 12 | 1 | 2.70932580511205 | 2.08570350783895 |
| 3504 | LARP1B | 12 | 1 | 1.48043002168767 | 1.97709311622213 |
| 3505 | PGRMC2 | 7 | 1 | -3.01877377946742 | -2.73287437778879 |
| 3506 | JADE1 | 9 | 1 | -0.493792265847995 | 3.16899384635519 |
| 3507 | SCLT1 | 16 | 1 | -3.89424918611415 | 2.96999301093649 |
| 3508 | C4orf33 | 10 | 1 | 3.36079098264806 | -1.30440793854166 |
| 3509 | AC096711.2 | 1 | 1 | 1.81222917120092 | 1.56062544959615 |
| 3510 | AC105383.1 | 15 | 1 | 5.81547905485265 | 0.870876923976648 |
| 3511 | PCDH10 | 15 | 1 | 5.94973494093053 | 0.7800480284078 |
| 3512 | PABPC4L | 7 | 1 | -3.90116141755946 | -2.64688990932871 |
| 3513 | PCDH18 | 4 | 1 | -4.65977738817103 | 0.282002703128564 |
| 3514 | SLC7A11 | 16 | 1 | -4.20518754442103 | 2.45636858123373 |
| 3515 | ELF2 | 5 | 1 | 3.664491191312 | 0.367261425433862 |
| 3516 | NDUFC1 | 13 | 1 | -0.661201432541682 | -1.84281907898356 |
| 3517 | NAA15 | 2 | 1 | -0.731526270703151 | 1.15539802688192 |
| 3518 | AC097376.2 | 12 | 1 | 2.62839438001745 | 1.90192498344015 |
| 3519 | SETD7 | 12 | 1 | 2.53105570356481 | 1.82460989135336 |
| 3520 | AC112236.1 | 6 | 1 | 2.22090627233617 | -2.51506923061777 |
| 3521 | MGST2 | 7 | 1 | -3.16583727319606 | -2.77323017937113 |
| 3522 | MAML3 | 9 | 1 | 1.6160395292484 | 2.76040734427999 |
| 3523 | SCOC | 10 | 1 | 3.47168017904393 | -1.37912376147677 |
| 3524 | CLGN | 5 | 1 | 3.38445331136815 | -0.922655553715122 |
| 3525 | ELMOD2 | 9 | 1 | 0.151185825272725 | 2.90367640632223 |
| 3526 | TBC1D9 | 1 | 1 | 2.8957243112766 | 1.75124277251791 |
| 3527 | RNF150 | 1 | 1 | 3.31485725919835 | 2.31343616622518 |
| 3528 | INPP4B | 15 | 1 | 5.5277884153568 | 0.8818466820104 |
| 3529 | USP38 | 9 | 1 | 0.668719425603078 | 2.83800567763876 |
| 3530 | GAB1 | 3 | 1 | -2.68091677148707 | 0.576159731326806 |
| 3531 | SMARCA5 | 8 | 1 | -1.55587814767726 | 1.44949664252828 |
| 3532 | SMARCA5-AS1 | 7 | 1 | -2.49655245263941 | -2.54195736271311 |
| 3533 | HHIP-AS1 | 14 | 1 | -6.15873597581752 | -0.480009271921408 |
| 3534 | HHIP | 14 | 1 | -6.59620402772792 | -0.869247432024968 |
| 3535 | ANAPC10 | 4 | 1 | -3.6373772474087 | -0.627184762777579 |
| 3536 | ABCE1 | 3 | 1 | -2.44106267412074 | 0.418102518497217 |
| 3537 | OTUD4 | 9 | 1 | 0.427067473336385 | 2.67727150100301 |
| 3538 | SMAD1 | 2 | 1 | 0.5765823272907 | 1.21858586448263 |
| 3539 | ZNF827 | 12 | 1 | 3.32054711858861 | 2.87490190642904 |
| 3540 | LSM6 | 19 | 1 | -3.91627739389308 | 0.644703046260583 |
| 3541 | REELD1 | 8 | 1 | -0.686285838679149 | 0.0296613730771421 |
| 3542 | POU4F2 | 20 | 1 | 3.46004892866247 | -3.68915115219523 |
| 3543 | TTC29 | 13 | 1 | -1.26258228738673 | -2.78782711845804 |
| 3544 | TMEM184C | 9 | 1 | 0.17356382290952 | 2.76541604178976 |
| 3545 | ARHGAP10 | 19 | 1 | -5.61560510118373 | 1.2562717356069 |
| 3546 | DCLK2 | 1 | 1 | 3.25433017294042 | 2.47866381782125 |
| 3547 | LRBA | 9 | 1 | 1.37280537168615 | 3.02953423636984 |
| 3548 | MAB21L2 | 15 | 1 | 5.80660654584996 | 0.564203158794153 |
| 3549 | RPS3A | 21 | 1 | -0.765519127444102 | -4.3450044236796 |
| 3550 | SH3D19 | 14 | 1 | -5.44392274339564 | 0.137298003612268 |

| 3551 | FBXW7 | 4 | 1 | -5.29140542467005 | -1.35204486471583 |
| --- | --- | --- | --- | --- | --- |
| 3552 | TIGD4 | 4 | 1 | -4.25853465516932 | 0.365546719012964 |
| 3553 | ARFIP1 | 2 | 1 | -0.328784130708053 | 2.22664131301473 |
| 3554 | FHDC1 | 18 | 1 | 5.49373508970372 | -0.360409065307868 |
| 3555 | TRIM2 | 1 | 1 | 4.39785648862951 | 1.13819040435384 |
| 3556 | MND1 | 19 | 1 | -4.70686171968348 | 1.54492224830221 |
| 3557 | TMEM131L | 4 | 1 | -5.30151103456385 | -1.65605823618342 |
| 3558 | RNF175 | 4 | 1 | -4.48719714601405 | 0.192564264713037 |
| 3559 | SFRP2 | 14 | 1 | -6.24482129533656 | -0.329490705551398 |
| 3560 | DCHS2 | 18 | 1 | 5.36039520780675 | -0.766252502025855 |
| 3561 | PLRG1 | 13 | 1 | -1.10428528507121 | -0.622288598837149 |
| 3562 | LRAT | 13 | 1 | -1.75227843244441 | -2.41085217815806 |
| 3563 | AC104407.1 | 18 | 1 | 6.19102026502721 | -2.64638517719675 |
| 3564 | NPY2R | 18 | 1 | 6.21558739225499 | -2.58797167164255 |
| 3565 | MAP9 | 1 | 1 | 3.38753344099157 | 1.79199446815084 |
| 3566 | GUCY1A1 | 15 | 1 | 5.3581311849796 | 0.258793488918054 |
| 3567 | GUCY1B1 | 5 | 1 | 4.41960073988073 | -0.299733801903021 |
| 3568 | PDGFC | 14 | 1 | -5.28499864061244 | -0.184014960350287 |
| 3569 | GLRB | 18 | 1 | 5.0366618780338 | -0.930692929001343 |
| 3570 | GRIA2 | 18 | 1 | 5.79386354963414 | -1.42728941065241 |
| 3571 | C4orf46 | 4 | 1 | -3.84628365953334 | -0.357662602485907 |
| 3572 | ETFDH | 3 | 1 | -2.68700908143885 | 0.732712880550134 |
| 3573 | PPID | 3 | 1 | -2.80586217363246 | -0.604486151756537 |
| 3574 | FNIP2 | 1 | 1 | 3.61294843237035 | 2.0769606985433 |
| 3575 | RAPGEF2 | 18 | 1 | 5.73540092985265 | -1.23460318070818 |
| 3576 | LINC02477 | 6 | 1 | 2.23554708044164 | -2.31645534855295 |
| 3577 | FSTL5 | 6 | 1 | 2.01256586591832 | -2.50196383816172 |
| 3578 | AC023136.1 | 6 | 1 | 1.95987869779699 | -2.58237276894022 |
| 3579 | NAF1 | 3 | 1 | -3.20670078714259 | -0.833046629251731 |
| 3580 | TMA16 | 11 | 1 | -3.67377112825282 | -1.05488283854891 |
| 3581 | Mar-01 | 18 | 1 | 5.5721843389713 | -0.870909501956594 |
| 3582 | AC093788.1 | 11 | 1 | -3.68438814599879 | -1.05582454723288 |
| 3583 | APELA | 14 | 1 | -6.77776740510829 | -0.521416946472418 |
| 3584 | TMEM192 | 1 | 1 | 4.0624563841068 | 0.78183664458822 |
| 3585 | KLHL2 | 15 | 1 | 4.40256048719518 | -0.0342860661165835 |
| 3586 | MSMO1 | 6 | 1 | 2.56874228994481 | -1.59961698872019 |
| 3587 | CPE | 10 | 1 | 3.6326902059757 | -1.47544352632929 |
| 3588 | SPOCK3 | 18 | 1 | 6.06062866727941 | -2.19460080486704 |
| 3589 | DDX60 | 4 | 1 | -4.38997052629359 | 0.426787392078149 |
| 3590 | PALLD | 4 | 1 | -4.2170903535641 | -0.0120084725039126 |
| 3591 | CBR4 | 12 | 1 | 1.90813495199315 | 2.54838813918661 |
| 3592 | SH3RF1 | 15 | 1 | 5.46225430051915 | 0.00780750888417947 |
| 3593 | NEK1 | 1 | 1 | 4.30253817121618 | 1.25015820640157 |
| 3594 | CLCN3 | 5 | 1 | 4.06566311399572 | -0.0522150240557314 |
| 3595 | HPF1 | 19 | 1 | -3.911356672839 | 0.761250153957116 |
| 3596 | MFAP3L | 7 | 1 | -3.79024671037562 | -2.66647301536967 |
| 3597 | AADAT | 1 | 1 | 1.47238542120092 | 1.69206298965048 |
| 3598 | GALNTL6 | 15 | 1 | 5.65322853605382 | 0.596969262538659 |
| 3599 | AC105285.1 | 4 | 1 | -4.23041890581019 | -1.06202316206862 |
| 3600 | GALNT7 | 11 | 1 | -4.05554698427088 | -1.50796870809962 |
| 3601 | AC097534.1 | 8 | 1 | -2.05333183725245 | 1.52931179183553 |
| 3602 | HMGB2 | 16 | 1 | -4.39234279115565 | 2.46763433593343 |
| 3603 | AC097534.2 | 16 | 1 | -3.31528829057582 | 2.21371974128317 |
| 3604 | SAP30 | 4 | 1 | -3.76819632966883 | -0.492967500509513 |
| 3605 | SCRG1 | 20 | 1 | 3.33300496618383 | -3.22979412895609 |
| 3606 | AC106895.2 | 11 | 1 | -3.93418668229945 | -2.36558698040415 |
| 3607 | FBXO8 | 7 | 1 | -3.03499244172938 | -2.70808003765513 |
| 3608 | CEP44 | 16 | 1 | -4.32589219529994 | 2.48056925910543 |
| 3609 | AC131094.1 | 11 | 1 | -4.18380950410731 | -1.91180561405588 |
| 3610 | GPM6A | 10 | 1 | 3.68548108617894 | -1.54709909779001 |
| 3611 | WDR17 | 1 | 1 | 3.66786099950902 | 0.391857282100427 |
| 3612 | SPCS3 | 2 | 1 | 1.11099256555669 | 1.49490512984823 |
| 3613 | VEGFC | 10 | 1 | 4.8120114950382 | -1.2675014875548 |
| 3614 | NEIL3 | 16 | 1 | -4.62017034013636 | 3.12654055732321 |
| 3615 | AGA | 11 | 1 | -3.66536019761927 | -1.56606672626902 |
| 3616 | AC098864.1 | 4 | 1 | -4.57707332094081 | -1.35704620105196 |
| 3617 | TENM3 | 2 | 1 | -0.788519904211833 | 2.06435765403341 |

| 3618 | DCTD | 11 | 1 | -3.38710998018153 | -1.55659298521448 |
| --- | --- | --- | --- | --- | --- |
| 3619 | WWC2-AS2 | 19 | 1 | -4.98396705110438 | 1.95174183028768 |
| 3620 | WWC2 | 14 | 1 | -5.04542993028529 | 0.251116529880273 |
| 3621 | CDKN2AIP | 2 | 1 | 0.685080840989278 | 1.1604658521993 |
| 3622 | ING2 | 2 | 1 | 1.4489914325916 | 0.791838661609399 |
| 3623 | RWDD4 | 13 | 1 | -1.75151167829402 | -2.2524089656489 |
| 3624 | TRAPPC11 | 2 | 1 | -0.168635167375876 | 2.33068622726034 |
| 3625 | STOX2 | 1 | 1 | 4.13188435117833 | 1.98150767463278 |
| 3626 | IRF2 | 4 | 1 | -4.92525456865199 | -0.891467181192529 |
| 3627 | LINC02427 | 11 | 1 | -3.28020714242823 | -2.24130664688517 |
| 3628 | LINC02365 | 6 | 1 | 1.91376067678563 | -2.77897082191874 |
| 3629 | CASP3 | 10 | 1 | 4.03728653471105 | -2.24361000877787 |
| 3630 | PRIMPOL | 16 | 1 | -3.12930129487879 | 2.62975849288534 |
| 3631 | CENPU | 16 | 1 | -4.63923667390712 | 2.16820849555563 |
| 3632 | ACSL1 | 10 | 1 | 4.97228075544469 | -0.942619244726193 |
| 3633 | HELT | 4 | 1 | -5.40258763749964 | -1.80272255760599 |
| 3634 | SLC25A4 | 10 | 1 | 3.45314861814611 | -1.73949299675394 |
| 3635 | CFAP97 | 1 | 1 | 2.87882377187841 | 1.31063307899069 |
| 3636 | SNX25 | 1 | 1 | 2.87328530828588 | 1.29731167930197 |
| 3637 | UFSP2 | 11 | 1 | -3.04195426423915 | -1.18008832556177 |
| 3638 | CCDC110 | 6 | 1 | 2.27025081197851 | -1.73635165316035 |
| 3639 | AC106897.1 | 3 | 1 | -3.46017693002589 | 0.495216504512536 |
| 3640 | PDLIM3 | 14 | 1 | -6.20081828554042 | 0.0226494826657652 |
| 3641 | SORBS2 | 5 | 1 | 3.94998718778722 | -0.15138564926554 |
| 3642 | AC093797.1 | 1 | 1 | 2.40065409223668 | 0.657905594287622 |
| 3643 | AC108472.1 | 1 | 1 | 2.4155373720371 | 0.748497859416711 |
| 3644 | AC096659.1 | 1 | 1 | 2.63915134946935 | 0.390983597217309 |
| 3645 | FAM149A | 6 | 1 | 2.52539516965978 | -2.03835116249491 |
| 3646 | KLKB1 | 2 | 1 | -0.176518149868323 | 1.83715547698568 |
| 3647 | FAT1 | 3 | 1 | -2.65037225206263 | 0.92448903697561 |
| 3648 | FRG1 | 10 | 1 | 3.03889133016698 | -1.74997739416529 |
| 3649 | PLEKHG4B | 11 | 1 | -4.07203386743434 | -1.1806219063418 |
| 3650 | CCDC127 | 5 | 1 | 3.44762970487706 | 0.249787227092492 |
| 3651 | SDHA | 17 | 1 | -0.219543596924021 | 0.000533477245080349 |
| 3652 | PDCD6 | 13 | 1 | -0.263266219951882 | -2.27548955303599 |
| 3653 | EXOC3-AS1 | 3 | 1 | -2.52591775377162 | 0.113083855090845 |
| 3654 | EXOC3 | 9 | 1 | 1.43470455686681 | 2.32202090400289 |
| 3655 | SLC9A3 | 8 | 1 | -0.545090928868129 | -0.21183429581095 |
| 3656 | CEP72 | 16 | 1 | -3.65842102487452 | 2.42521490233968 |
| 3657 | TPPP | 5 | 1 | 4.04573370496862 | 0.31279994624685 |
| 3658 | ZDHHC11B | 1 | 1 | 2.55819798986547 | 0.498929873882043 |
| 3659 | ZDHHC11 | 1 | 1 | 1.84951974432103 | 0.88904346602987 |
| 3660 | BRD9 | 2 | 1 | -0.342557095185592 | 1.51356019157003 |
| 3661 | TRIP13 | 19 | 1 | -4.93332313020594 | 1.95784987586569 |
| 3662 | SLC12A7 | 7 | 1 | -3.08797596414454 | -2.51556514126231 |
| 3663 | CLPTM1L | 8 | 1 | -1.4842771144665 | -0.0603031955378176 |
| 3664 | LPCAT1 | 5 | 1 | 3.58671523611181 | -0.597603514017356 |
| 3665 | MRPL36 | 6 | 1 | 1.55876387636297 | -1.40951131206919 |
| 3666 | NDUFS6 | 13 | 1 | -0.743636653021647 | -1.08351255875994 |
| 3667 | IRX2 | 14 | 1 | -6.58888314683802 | -1.09710664969851 |
| 3668 | C5orf38 | 14 | 1 | -6.65787194688685 | -1.00100782436301 |
| 3669 | IRX1 | 14 | 1 | -6.54739497621424 | -1.11582836431433 |
| 3670 | ADAMTS16 | 15 | 1 | 5.66114260236852 | 0.0852533735616087 |
| 3671 | ICE1 | 9 | 1 | 0.203828289910481 | 2.58002866881918 |
| 3672 | MED10 | 17 | 1 | -0.074387431339099 | -0.34261415821482 |
| 3673 | UBE2QL1 | 5 | 1 | 3.92038990537755 | -0.413369401516211 |
| 3674 | NSUN2 | 8 | 1 | -1.93150447328456 | 0.692309037624109 |
| 3675 | SRD5A1 | 10 | 1 | 2.93908812086217 | -1.68200735670496 |
| 3676 | TENT4A | 9 | 1 | 0.290138080521748 | 3.17755068915914 |
| 3677 | ADCY2 | 6 | 1 | 2.4613251833164 | -1.80392800194193 |
| 3678 | C5orf49 | 6 | 1 | 1.98214770834081 | -2.41792057377268 |
| 3679 | MTRR | 9 | 1 | 1.20588506738775 | 2.95037974494528 |
| 3680 | FASTKD3 | 2 | 1 | 1.33918680231206 | 0.928385034976709 |
| 3681 | SEMA5A | 14 | 1 | -5.65178130586512 | 0.383953467784631 |
| 3682 | SNHG18 | 14 | 1 | -5.46118710954554 | -0.569100662292731 |
| 3683 | FAM173B | 2 | 1 | 0.344769790574239 | 1.07186926978658 |
| 3684 | AC012640.1 | 16 | 1 | -4.11567471940882 | 2.80206169265341 |

| 3685 | CCT5 | 8 | 1 | -1.63184843976863 | 0.210634366451013 |
| --- | --- | --- | --- | --- | --- |
| 3686 | CMBL | 18 | 1 | 6.10771633665197 | -2.80350242477824 |
| 3687 | AC012640.2 | 4 | 1 | -3.92048285920985 | -0.421872182907355 |
| 3688 | Mar-06 | 5 | 1 | 3.770115151807 | 0.332558409152734 |
| 3689 | ROPN1L | 13 | 1 | -0.641632482603862 | -2.7889673553126 |
| 3690 | ANKRD33B | 7 | 1 | -3.44254301507838 | -3.55228792053629 |
| 3691 | DAP | 11 | 1 | -3.72335432489283 | -1.5506529651301 |
| 3692 | AC012629.2 | 14 | 1 | -5.67866872270472 | 0.247737542567956 |
| 3693 | CTNND2 | 12 | 1 | 1.64210034887426 | 2.05407704490255 |
| 3694 | TRIO | 1 | 1 | 3.59053398649328 | 2.25031198638509 |
| 3695 | OTULINL | 5 | 1 | 3.28646422903173 | -0.54788775664736 |
| 3696 | OTULIN | 9 | 1 | 0.41490407268636 | 2.96448959487509 |
| 3697 | ANKH | 1 | 1 | 4.24601055662267 | 1.83658637183737 |
| 3698 | FBXL7 | 7 | 1 | -3.41648863275416 | -2.90080521923472 |
| 3699 | Mar-11 | 6 | 1 | 2.55775548498266 | -2.66684208733012 |
| 3700 | ZNF622 | 2 | 1 | 0.866020932599233 | 0.814315692363489 |
| 3701 | RETREG1 | 10 | 1 | 3.73176623861425 | -1.03949448388983 |
| 3702 | MYO10 | 11 | 1 | -3.81863068063624 | -2.36099873405863 |
| 3703 | BASP1 | 10 | 1 | 4.13390804807775 | -1.11859017533232 |
| 3704 | AC026790.1 | 15 | 1 | 5.58241726438634 | 0.781244055209863 |
| 3705 | BASP1-AS1 | 15 | 1 | 5.69776178876989 | 0.79192866462301 |
| 3706 | LINC02223 | 15 | 1 | 4.99148251096837 | -0.426183744491828 |
| 3707 | CDH18 | 5 | 1 | 4.01752449552648 | -0.721770106972945 |
| 3708 | CDH12 | 15 | 1 | 5.66097237150304 | 0.684154168544519 |
| 3709 | CDH10 | 11 | 1 | -3.72348879297145 | -2.45072029453684 |
| 3710 | AC091885.2 | 11 | 1 | -3.60099528749354 | -2.12450466972758 |
| 3711 | LINC02211 | 1 | 1 | 1.66917742769353 | 1.40720380920004 |
| 3712 | CDH9 | 6 | 1 | 2.32875491659276 | -1.83857707601954 |
| 3713 | PURPL | 7 | 1 | -2.78822587449916 | -2.68055425506998 |
| 3714 | CDH6 | 18 | 1 | 5.67653681318395 | -2.27811168056895 |
| 3715 | DROSHA | 9 | 1 | 0.256468251153157 | 2.92494763511251 |
| 3716 | C5orf22 | 2 | 1 | -0.302460830853059 | 2.18588365691732 |
| 3717 | PDZD2 | 1 | 1 | 2.95294190923803 | 1.27507080215048 |
| 3718 | GOLPH3 | 12 | 1 | 2.04055954496496 | 2.3310140528066 |
| 3719 | AC025181.2 | 6 | 1 | 0.99810971300237 | -2.48305962902476 |
| 3720 | MTMR12 | 12 | 1 | 2.4499292520725 | 2.00467814582418 |
| 3721 | ZFR | 2 | 1 | 0.296569779320882 | 1.44596661704611 |
| 3722 | SUB1 | 13 | 1 | -0.908157155112102 | -2.05031143051554 |
| 3723 | NPR3 | 10 | 1 | 4.48779703657262 | -2.29175887924601 |
| 3724 | TARS | 6 | 1 | 2.54743863622777 | -1.67721430879999 |
| 3725 | ADAMTS12 | 11 | 1 | -3.47187088449366 | -1.7255774579661 |
| 3726 | AMACR | 1 | 1 | 3.71637178938024 | 1.05099631923269 |
| 3727 | AC139792.1 | 1 | 1 | 2.45883418600194 | 0.5117534555776 |
| 3728 | AC025754.2 | 19 | 1 | -5.28137610872157 | 1.07913221496176 |
| 3729 | RAI14 | 14 | 1 | -5.16084931810267 | 0.045782641349542 |
| 3730 | RAD1 | 5 | 1 | 2.38399340192907 | -0.160751327099097 |
| 3731 | BRIX1 | 8 | 1 | -1.46466408689387 | 0.0233320750577329 |
| 3732 | DNAJC21 | 17 | 1 | 1.46457339803808 | 0.526683584628808 |
| 3733 | PRLR | 18 | 1 | 5.6626351026737 | -0.691146268667472 |
| 3734 | SPEF2 | 1 | 1 | 2.94897963087194 | 1.23786129134725 |
| 3735 | UGT3A1 | 7 | 1 | -3.63806270082362 | -3.2211304746287 |
| 3736 | LMBRD2 | 1 | 1 | 3.45887471716039 | 2.04327429908346 |
| 3737 | SKP2 | 19 | 1 | -4.78372214753993 | 1.602128879009 |
| 3738 | NADK2 | 14 | 1 | -5.46337960679896 | 0.104388729511011 |
| 3739 | SLC1A3 | 14 | 1 | -6.37460015733607 | -0.277313097538245 |
| 3740 | NIPBL | 2 | 1 | -0.452755838826968 | 2.39615978377889 |
| 3741 | CPLANE1 | 1 | 1 | 3.31829310934179 | 1.99699129241537 |
| 3742 | NUP155 | 9 | 1 | -0.613787964180781 | 3.10196246284078 |
| 3743 | WDR70 | 2 | 1 | -0.803440794543101 | 1.81065715926717 |
| 3744 | GDNF-AS1 | 15 | 1 | 5.85500837843053 | 0.733489290652978 |
| 3745 | GDNF | 15 | 1 | 5.86600233595006 | 0.73248042243551 |
| 3746 | LIFR | 1 | 1 | 2.96696569006078 | 1.45076192992758 |
| 3747 | OSMR | 17 | 1 | 2.11022521535985 | 0.719176665721643 |
| 3748 | RICTOR | 12 | 1 | 2.66352583448522 | 1.98346413749288 |
| 3749 | DAB2 | 14 | 1 | -5.54180119951136 | 0.126805201946008 |
| 3750 | TTC33 | 1 | 1 | 4.21761633436315 | 1.50425542968343 |
| 3751 | PRKAA1 | 1 | 1 | 3.77611471693151 | 2.24227966445516 |

| 3752 | RPL37 | 21 | 1 | -0.765911981657817 | -4.35385499817301 |
| --- | --- | --- | --- | --- | --- |
| 3753 | PLCXD3 | 10 | 1 | 4.71419502775304 | -1.26879091483523 |
| 3754 | OXCT1 | 5 | 1 | 3.33781124631994 | -0.541231437744391 |
| 3755 | C5orf51 | 2 | 1 | -0.739091113404109 | 1.79569901603292 |
| 3756 | GHR | 14 | 1 | -5.47956441362269 | 0.268471614299524 |
| 3757 | CCDC152 | 14 | 1 | -6.10614989717372 | 0.206842557368982 |
| 3758 | SELENOP | 14 | 1 | -5.87021993120082 | -0.0128708325045229 |
| 3759 | ZNF131 | 9 | 1 | 1.295590176984 | 2.48385395186971 |
| 3760 | NIM1K | 5 | 1 | 3.97944523374669 | -0.824939458789122 |
| 3761 | AC114947.2 | 1 | 1 | 2.35533834974401 | 0.528854862628686 |
| 3762 | HMGCS1 | 6 | 1 | 2.50190760175817 | -1.63972692114283 |
| 3763 | TMEM267 | 3 | 1 | -2.1839784236706 | -0.366259499611151 |
| 3764 | C5orf34 | 16 | 1 | -4.7974502893246 | 2.53136481422018 |
| 3765 | PAIP1 | 8 | 1 | -0.724382505015208 | 0.572115198550928 |
| 3766 | NNT-AS1 | 2 | 1 | 0.301923647328542 | 1.02054907458853 |
| 3767 | NNT | 12 | 1 | 3.35452653448217 | 2.61391557830404 |
| 3768 | FGF10 | 14 | 1 | -6.87355779130824 | -0.618865802110922 |
| 3769 | MRPS30-DT | 13 | 1 | -1.50222788770564 | -2.71784315926005 |
| 3770 | MRPS30 | 3 | 1 | -2.39954923112757 | -0.351171656669867 |
| 3771 | HCN1 | 7 | 1 | -3.63825486619837 | -3.47664128166605 |
| 3772 | EMB | 2 | 1 | -1.09330599029429 | 1.80427326339315 |
| 3773 | PARP8 | 15 | 1 | 5.2646105436527 | 0.62970794814657 |
| 3774 | AC008808.2 | 4 | 1 | -5.06869767625697 | -1.74356190782954 |
| 3775 | LINC02106 | 4 | 1 | -4.96235631425746 | -1.8108384452479 |
| 3776 | AC010478.1 | 18 | 1 | 6.08842016737096 | -2.89232848507334 |
| 3777 | ISL1 | 18 | 1 | 6.13582350294225 | -2.81877468449045 |
| 3778 | PELO | 17 | 1 | 0.424487247868703 | 0.052720741210687 |
| 3779 | ITGA2 | 4 | 1 | -5.09268449266322 | -0.5847921810763 |
| 3780 | MOCS2 | 13 | 1 | -1.50321863611109 | -0.745801209630263 |
| 3781 | FST | 14 | 1 | -6.46303866823085 | -0.165290459217322 |
| 3782 | NDUFS4 | 6 | 1 | 1.86030460874669 | -1.09536398869921 |
| 3783 | ARL15 | 15 | 1 | 4.49544645826452 | -0.0368522248880984 |
| 3784 | SNX18 | 2 | 1 | -0.077169373825862 | 1.46457685607504 |
| 3785 | CDC20B | 13 | 1 | -0.563403889731242 | -3.37285314423014 |
| 3786 | GPX8 | 4 | 1 | -4.57129548509486 | 0.403504744945276 |
| 3787 | MCIDAS | 13 | 1 | -0.570525452689006 | -3.38912163597513 |
| 3788 | CCNO | 13 | 1 | -0.606518790320231 | -3.36482129913737 |
| 3789 | DHX29 | 2 | 1 | 1.42388941328161 | 0.909258142886865 |
| 3790 | MTREX | 9 | 1 | 0.969163551732228 | 2.99944962638448 |
| 3791 | PLPP1 | 10 | 1 | 3.85221602003209 | -1.18013740998675 |
| 3792 | SLC38A9 | 8 | 1 | -1.64917908628352 | 1.5014470972402 |
| 3793 | IL6ST | 1 | 1 | 3.40643621961706 | 1.33621133941244 |
| 3794 | ANKRD55 | 15 | 1 | 5.70209862272374 | 0.202668324886072 |
| 3795 | MAP3K1 | 12 | 1 | 3.30898524801366 | 2.87981070655416 |
| 3796 | SETD9 | 3 | 1 | -3.19449876268275 | -0.484060867847693 |
| 3797 | MIER3 | 9 | 1 | 0.923523917599843 | 3.00710190909933 |
| 3798 | GPBP1 | 9 | 1 | 1.57228888551824 | 2.91937269347738 |
| 3799 | PLK2 | 18 | 1 | 5.29555918256871 | -0.694749011816275 |
| 3800 | RAB3C | 15 | 1 | 5.20004393140905 | 0.164258495746362 |
| 3801 | PDE4D | 18 | 1 | 5.3039958624088 | -0.794253430487406 |
| 3802 | DEPDC1B | 16 | 1 | -4.57665942628749 | 3.04555954116415 |
| 3803 | ERCC8 | 8 | 1 | -1.15241818149455 | 1.59477771895956 |
| 3804 | NDUFAF2 | 3 | 1 | -1.94301711042292 | -0.0221133671419741 |
| 3805 | SMIM15 | 11 | 1 | -3.1182556005276 | -1.09299608987738 |
| 3806 | ZSWIM6 | 9 | 1 | 0.838544741078542 | 2.66921486037801 |
| 3807 | KIF2A | 7 | 1 | -2.67611788232691 | -3.62612996918131 |
| 3808 | DIMT1 | 11 | 1 | -3.75974009950526 | -2.37031386715342 |
| 3809 | IPO11 | 3 | 1 | -3.77690981347926 | 1.25765718597006 |
| 3810 | RNF180 | 9 | 1 | 0.705840721532033 | 2.89948715346884 |
| 3811 | RGS7BP | 18 | 1 | 6.05065132658117 | -2.15062270981241 |
| 3812 | SHISAL2B | 19 | 1 | -5.07608293016322 | 1.08780480998586 |
| 3813 | SREK1IP1 | 13 | 1 | -2.11478506048091 | -1.92626629692484 |
| 3814 | CWC27 | 3 | 1 | -2.16435204465754 | -0.106505318702948 |
| 3815 | ADAMTS6 | 11 | 1 | -3.77115891893275 | -2.38848064762522 |
| 3816 | CENPK | 19 | 1 | -4.92868016679652 | 2.0312150873525 |
| 3817 | PPWD1 | 3 | 1 | -2.78681610544093 | -0.258053585113776 |
| 3818 | TRIM23 | 1 | 1 | 3.37967778722875 | 2.40296973365377 |

| 3819 | TRAPPC13 | 2 | 1 | -0.138736248210742 | 1.42855824607443 |
| --- | --- | --- | --- | --- | --- |
| 3820 | SHLD3 | 1 | 1 | 1.87962295095556 | 0.816490665851343 |
| 3821 | SGTB | 5 | 1 | 2.96081949751012 | -0.0874824367182375 |
| 3822 | NLN | 1 | 1 | 3.24329377691381 | 1.97116984504293 |
| 3823 | ERBIN | 2 | 1 | -1.22667150218852 | 2.54541339057516 |
| 3824 | SREK1 | 2 | 1 | 1.14907278101079 | 1.0866851963384 |
| 3825 | MAST4 | 3 | 1 | -3.23063252885707 | 0.229871050296533 |
| 3826 | MAST4-AS1 | 4 | 1 | -4.14671777208216 | -0.00244383436609519 |
| 3827 | PIK3R1 | 15 | 1 | 4.68166471998327 | 0.0312186636311887 |
| 3828 | SLC30A5 | 3 | 1 | -2.38964127023585 | 0.271439806400049 |
| 3829 | CCNB1 | 16 | 1 | -4.3614241929806 | 3.12913979667257 |
| 3830 | CENPH | 19 | 1 | -4.2341208310879 | 0.728672162471521 |
| 3831 | MRPS36 | 6 | 1 | 0.946343317433522 | -2.25781641823221 |
| 3832 | CDK7 | 3 | 1 | -2.22284315545924 | -0.312743648113501 |
| 3833 | AK6 | 3 | 1 | -2.57861422021754 | -0.908717958414745 |
| 3834 | TAF9 | 5 | 1 | 2.7174446729862 | -1.05663618368078 |
| 3835 | RAD17 | 9 | 1 | 0.922953143521474 | 2.39624537604879 |
| 3836 | OCLN | 5 | 1 | 4.10786749403112 | -0.201271399082434 |
| 3837 | GTF2H2C | 1 | 1 | 2.84465028326146 | 1.6960054792745 |
| 3838 | SMN1 | 2 | 1 | -0.404886052206828 | 0.949602381168115 |
| 3839 | NAIP | 1 | 1 | 3.02666546384923 | 2.05489005225729 |
| 3840 | GTF2H2 | 1 | 1 | 4.58989693204992 | 1.38294495719503 |
| 3841 | LINC02197 | 15 | 1 | 5.74054028074376 | 0.812265411792505 |
| 3842 | BDP1 | 12 | 1 | 3.11772157232396 | 2.90774478095602 |
| 3843 | MCCC2 | 3 | 1 | -3.13870690782435 | -0.749593391241324 |
| 3844 | MAP1B | 10 | 1 | 3.66315771619909 | -1.504207535805 |
| 3845 | MRPS27 | 3 | 1 | -2.40335677583583 | -1.03127299171854 |
| 3846 | LINC02056 | 7 | 1 | -3.52586577852137 | -2.94058285576273 |
| 3847 | TNPO1 | 9 | 1 | 1.89510394613378 | 2.92025102752279 |
| 3848 | AC008972.1 | 4 | 1 | -4.03083990533717 | -0.550653203548682 |
| 3849 | FCHO2 | 1 | 1 | 3.28479291479223 | 1.16034688132833 |
| 3850 | FOXD1 | 14 | 1 | -5.75221847017176 | 0.225602284847009 |
| 3851 | AC099522.2 | 3 | 1 | -3.34484861810572 | 0.293035999713647 |
| 3852 | BTF3 | 21 | 1 | -0.811641738013103 | -4.28905497414042 |
| 3853 | UTP15 | 8 | 1 | -1.92658101041682 | 1.07238532680105 |
| 3854 | ENC1 | 6 | 1 | 1.55558647195928 | -2.89003871303965 |
| 3855 | HEXB | 14 | 1 | -5.57167790849574 | 0.235641733585107 |
| 3856 | GFM2 | 2 | 1 | -0.148912660971953 | 1.55444564002584 |
| 3857 | NSA2 | 21 | 1 | -1.14948706348307 | -4.54442988258768 |
| 3858 | FAM169A | 1 | 1 | 3.85837866346471 | 2.10915269034933 |
| 3859 | HMGCR | 5 | 1 | 3.25030900518529 | -0.737901373924506 |
| 3860 | COL4A3BP | 1 | 1 | 3.07374836485021 | 1.25416626113485 |
| 3861 | POLK | 12 | 1 | 2.13167430441015 | 2.51398171561788 |
| 3862 | AC010245.1 | 1 | 1 | 2.02921821157567 | 1.17913140433859 |
| 3863 | POC5 | 16 | 1 | -3.7263665052212 | 2.80382312911581 |
| 3864 | SV2C | 6 | 1 | 2.5733800081455 | -2.13702712875773 |
| 3865 | IQGAP2 | 14 | 1 | -5.85493634660609 | 0.10295333045553 |
| 3866 | F2R | 8 | 1 | -1.32720981557734 | 1.54303254264425 |
| 3867 | CRHBP | 13 | 1 | -0.182700425580813 | -3.21094761711527 |
| 3868 | AGGF1 | 2 | 1 | -0.758414313391521 | 2.29237808364462 |
| 3869 | ZBED3 | 2 | 1 | -0.880031332568004 | 0.964730397639978 |
| 3870 | ZBED3-AS1 | 8 | 1 | -1.75469051320918 | 1.35941113608907 |
| 3871 | WDR41 | 8 | 1 | -1.57689569909938 | 0.472021714626062 |
| 3872 | OTP | 20 | 1 | 3.3821687845432 | -3.72138558250834 |
| 3873 | TBCA | 13 | 1 | -1.55538652856715 | -2.44624124390055 |
| 3874 | AP3B1 | 4 | 1 | -3.74784682710536 | -0.401034816326392 |
| 3875 | SCAMP1-AS1 | 3 | 1 | -2.99749945123561 | -0.00558076721597922 |
| 3876 | SCAMP1 | 15 | 1 | 4.75149275343053 | 0.331559673724878 |
| 3877 | LHFPL2 | 14 | 1 | -4.86032937486537 | 0.371499673305261 |
| 3878 | ARSB | 1 | 1 | 2.38678504507177 | 0.583300725398767 |
| 3879 | BHMT | 7 | 1 | -1.77672408540614 | -3.50959597450663 |
| 3880 | JMY | 9 | 1 | 1.00470305959813 | 2.11506976264547 |
| 3881 | HOMER1 | 1 | 1 | 3.79690386335485 | 2.16092766898702 |
| 3882 | TENT2 | 8 | 1 | -1.44591568429835 | 1.21527971404623 |
| 3883 | MTX3 | 8 | 1 | -1.84819744546778 | 1.43033541816305 |
| 3884 | SERINC5 | 4 | 1 | -5.18783305604823 | -1.66122822386194 |
| 3885 | ZFYVE16 | 8 | 1 | -1.8005008550442 | 1.78513206618856 |

| 3886 | AC008771.1 | 5 | 1 | 3.47603727857702 | -0.661561905564559 |
| --- | --- | --- | --- | --- | --- |
| 3887 | DHFR | 19 | 1 | -4.72485898454554 | 1.09520377296041 |
| 3888 | MSH3 | 2 | 1 | -1.30403660257228 | 1.91019262450765 |
| 3889 | CKMT2-AS1 | 2 | 1 | -1.34004984815486 | 2.03309024947714 |
| 3890 | ZCCHC9 | 4 | 1 | -3.62526272256739 | 0.147187606273401 |
| 3891 | SSBP2 | 7 | 1 | -2.6736960264004 | -3.56853662353922 |
| 3892 | ATG10 | 13 | 1 | -1.53254984338649 | -1.94664059502054 |
| 3893 | RPS23 | 21 | 1 | -0.763270423010661 | -4.33412228447367 |
| 3894 | ATP6AP1L | 9 | 1 | 0.695082321568654 | 2.40282286780905 |
| 3895 | TMEM167A | 2 | 1 | -0.441393315986468 | 1.21716941970419 |
| 3896 | XRCC4 | 16 | 1 | -3.87154601533778 | 2.78356088775228 |
| 3897 | VCAN | 18 | 1 | 5.93766094724767 | -1.75898145061899 |
| 3898 | VCAN-AS1 | 18 | 1 | 5.89738251249425 | -1.9652813516276 |
| 3899 | HAPLN1 | 14 | 1 | -6.1490390153683 | 0.144195333896387 |
| 3900 | EDIL3 | 10 | 1 | 4.15492130796544 | -1.30653245669771 |
| 3901 | COX7C | 21 | 1 | -0.460671112135722 | -3.73696266037394 |
| 3902 | RASA1 | 9 | 1 | 0.574785604878591 | 2.66265668052267 |
| 3903 | CCNH | 8 | 1 | -0.902287826136424 | 0.21078385490011 |
| 3904 | TMEM161B | 1 | 1 | 3.06183601896398 | 1.50732649940084 |
| 3905 | TMEM161B-AS1 | 8 | 1 | -1.27339409311183 | 0.874835506854761 |
| 3906 | LINC00461 | 18 | 1 | 6.09140326063268 | -2.87690876346995 |
| 3907 | MEF2C | 18 | 1 | 6.2843949941837 | -2.4290538869517 |
| 3908 | MEF2C-AS1 | 7 | 1 | -2.52517221887477 | -3.65552984100748 |
| 3909 | CETN3 | 3 | 1 | -3.67554901559718 | 1.08629752772878 |
| 3910 | MBLAC2 | 15 | 1 | 5.30651117841832 | -0.143127604545844 |
| 3911 | POLR3G | 13 | 1 | -1.40694008787043 | -1.94192241054941 |
| 3912 | LYSMD3 | 1 | 1 | 2.86860562841527 | 1.12673367637228 |
| 3913 | ADGRV1 | 14 | 1 | -6.15575097520716 | -0.756349935354483 |
| 3914 | ARRDC3 | 11 | 1 | -3.55137060602076 | -1.87042836767603 |
| 3915 | AC114316.2 | 15 | 1 | 6.13439728300206 | 0.779532448230493 |
| 3916 | NR2F1-AS1 | 14 | 1 | -6.80397914369471 | -0.785446494044554 |
| 3917 | NR2F1 | 14 | 1 | -6.56732390840419 | -1.05549341362883 |
| 3918 | FAM172A | 1 | 1 | 4.96364285032384 | 0.941423789439905 |
| 3919 | POU5F2 | 12 | 1 | 2.39956475774877 | 2.75608266967367 |
| 3920 | KIAA0825 | 9 | 1 | 1.49329330007665 | 2.26127041953634 |
| 3921 | SLF1 | 15 | 1 | 4.70331265012853 | -0.30288551670481 |
| 3922 | MCTP1 | 12 | 1 | 2.74273420850866 | 2.33853186744283 |
| 3923 | FAM81B | 13 | 1 | -0.35352156330712 | -3.27391777855326 |
| 3924 | TTC37 | 1 | 1 | 1.48409046213262 | 1.45914734977316 |
| 3925 | ARSK | 3 | 1 | -2.46698378045924 | 0.115609542308557 |
| 3926 | RHOBTB3 | 14 | 1 | -6.14692567308314 | -0.656056224526656 |
| 3927 | GLRX | 10 | 1 | 3.30833269636266 | -1.79158697945048 |
| 3928 | ELL2 | 6 | 1 | 1.59854437391393 | -2.70479307991434 |
| 3929 | PCSK1 | 15 | 1 | 5.69260336439245 | 0.182914153514612 |
| 3930 | CAST | 11 | 1 | -4.12085221727259 | -1.57218740803171 |
| 3931 | ERAP1 | 2 | 1 | -0.0432502480304976 | 2.34408272880148 |
| 3932 | AC009126.1 | 15 | 1 | 5.02469660322301 | 0.582396403728235 |
| 3933 | ERAP2 | 15 | 1 | 4.8641297964298 | 0.586007610736596 |
| 3934 | LNPEP | 1 | 1 | 4.88875080625646 | 0.819644824443567 |
| 3935 | LIX1 | 14 | 1 | -6.09254907091029 | -0.1962463699 |
| 3936 | RIOK2 | 3 | 1 | -2.14296661336787 | -0.257292672218573 |
| 3937 | RGMB | 6 | 1 | 2.43519450704686 | -2.17600010258127 |
| 3938 | RGMB-AS1 | 6 | 1 | 2.2242634443485 | -2.54348467213084 |
| 3939 | AC008522.1 | 6 | 1 | 2.21369983236425 | -2.22678051811625 |
| 3940 | CHD1 | 9 | 1 | 0.300343110963033 | 2.89668883460592 |
| 3941 | FAM174A | 10 | 1 | 3.30050875227086 | -1.22743089419771 |
| 3942 | ST8SIA4 | 15 | 1 | 5.43674637357824 | 0.819804564891565 |
| 3943 | LINC00492 | 7 | 1 | -3.01967953164942 | -3.41537104469706 |
| 3944 | AC099487.1 | 4 | 1 | -4.95933364351161 | -2.14404068809916 |
| 3945 | LINC00491 | 2 | 1 | -0.535640046194865 | 1.65912570136617 |
| 3946 | PAM | 5 | 1 | 3.86831451933019 | -0.32655255419184 |
| 3947 | PPIP5K2 | 12 | 1 | 2.56682683508031 | 2.91905058997701 |
| 3948 | C5orf30 | 15 | 1 | 4.95442654173009 | -0.287503703655493 |
| 3949 | NUDT12 | 2 | 1 | -0.837085053519084 | 1.45909561294149 |
| 3950 | EFNA5 | 8 | 1 | -1.47072361429103 | 1.58947433608602 |
| 3951 | FBXL17 | 1 | 1 | 4.05613543073766 | 1.57573355811666 |
| 3952 | LINC01023 | 4 | 1 | -3.77366755922206 | -0.446907981457007 |

| 3953 | FER | 1 | 1 | 3.73065210859411 | 0.899049178539026 |
| --- | --- | --- | --- | --- | --- |
| 3954 | PJA2 | 1 | 1 | 3.68113829176061 | 0.493232861934411 |
| 3955 | MAN2A1 | 11 | 1 | -3.66937612016566 | -1.85158000809122 |
| 3956 | SLC25A46 | 1 | 1 | 3.37017919103734 | 2.47582997459005 |
| 3957 | WDR36 | 2 | 1 | -0.442763507560565 | 2.44550456183981 |
| 3958 | CAMK4 | 1 | 1 | 3.18747664968602 | 1.44729770797323 |
| 3959 | STARD4 | 8 | 1 | -2.18949376066096 | 1.19557251113485 |
| 3960 | STARD4-AS1 | 12 | 1 | 1.58956719915502 | 2.04506458419393 |
| 3961 | NREP | 5 | 1 | 4.26750446836583 | 0.585438386378991 |
| 3962 | EPB41L4A | 1 | 1 | 3.70652653257482 | 0.761327163158166 |
| 3963 | EPB41L4A-AS1 | 17 | 1 | 0.108309224053548 | 0.429214135585534 |
| 3964 | EPB41L4A-DT | 5 | 1 | 3.49675728361242 | -0.320075019420874 |
| 3965 | APC | 1 | 1 | 4.56085564176671 | 0.745705024181116 |
| 3966 | SRP19 | 13 | 1 | -0.735151723221614 | -1.45955811363627 |
| 3967 | REEP5 | 10 | 1 | 3.74500943700902 | -1.52516435009409 |
| 3968 | DCP2 | 16 | 1 | -3.46289037187464 | 2.34985174316 |
| 3969 | MCC | 14 | 1 | -5.73648093660243 | -0.421285881819022 |
| 3970 | YTHDC2 | 12 | 1 | 2.0920133737766 | 2.90703143256735 |
| 3971 | KCNN2 | 18 | 1 | 5.68679691831701 | -0.413905396284354 |
| 3972 | TRIM36 | 15 | 1 | 4.34144617597692 | -0.191070183338416 |
| 3973 | PGGT1B | 8 | 1 | -1.87327347715266 | 1.58821787017416 |
| 3974 | CCDC112 | 10 | 1 | 3.82021881620519 | -1.37756730538775 |
| 3975 | FEM1C | 9 | 1 | 1.81169678251378 | 3.13014639991354 |
| 3976 | TMED7 | 2 | 1 | 0.22350393216245 | 2.26397861617635 |
| 3977 | CDO1 | 10 | 1 | 4.0254371313297 | -0.982320620836508 |
| 3978 | ATG12 | 12 | 1 | 2.55697752515905 | 1.81495131629537 |
| 3979 | AP3S1 | 5 | 1 | 3.59937788526647 | -0.980030804099333 |
| 3980 | AC034236.2 | 3 | 1 | -3.08687876184352 | 0.344877616344201 |
| 3981 | COMMD10 | 3 | 1 | -1.76462291200526 | -0.673680856050742 |
| 3982 | SEMA6A | 7 | 1 | -3.10382078607447 | -2.90161333900858 |
| 3983 | SEMA6A-AS1 | 7 | 1 | -3.17255829294093 | -2.89659045559336 |
| 3984 | DTWD2 | 11 | 1 | -3.4697804303921 | -1.98578892570902 |
| 3985 | DMXL1 | 12 | 1 | 2.85473753492467 | 2.84756125587057 |
| 3986 | TNFAIP8 | 19 | 1 | -5.12950633485682 | 1.33640517371725 |
| 3987 | HSD17B4 | 2 | 1 | -0.650207296208217 | 2.35277046340536 |
| 3988 | PRR16 | 7 | 1 | -3.71890853364833 | -2.54523263794352 |
| 3989 | AC114284.1 | 8 | 1 | -1.29030428846247 | -0.172395213665259 |
| 3990 | SRFBP1 | 8 | 1 | -1.31929717977412 | 1.37534560340475 |
| 3991 | ZNF474 | 13 | 1 | -0.0276382567442198 | -2.99328432899882 |
| 3992 | SNCAIP | 15 | 1 | 5.02794577161901 | -0.376874490799201 |
| 3993 | SNX2 | 13 | 1 | -0.679878696993663 | -1.0983577660935 |
| 3994 | SNX24 | 7 | 1 | -2.82520483453639 | -2.05819760185648 |
| 3995 | PPIC | 14 | 1 | -5.57021830995448 | -0.416388078750861 |
| 3996 | CEP120 | 2 | 1 | 0.264568641587422 | 2.33693517821859 |
| 3997 | CSNK1G3 | 2 | 1 | -0.807650253371074 | 1.45563997405599 |
| 3998 | ZNF608 | 12 | 1 | 2.83803249876134 | 2.197179452358 |
| 3999 | GRAMD2B | 4 | 1 | -4.31117509324916 | -1.12195730132033 |
| 4000 | ALDH7A1 | 3 | 1 | -2.47021769006617 | 0.999761001048791 |
| 4001 | PHAX | 2 | 1 | 0.95251108686559 | 1.03782309669088 |
| 4002 | LMNB1-DT | 14 | 1 | -6.11224769075282 | -0.539210363449347 |
| 4003 | LMNB1 | 16 | 1 | -3.92517398317225 | 2.25365246909689 |
| 4004 | Mar-03 | 12 | 1 | 1.96028782407872 | 2.37211908477377 |
| 4005 | MEGF10 | 4 | 1 | -4.90026591737635 | -0.681598811568511 |
| 4006 | PRRC1 | 2 | 1 | -0.896521911219432 | 1.15549911635946 |
| 4007 | LINC01184 | 3 | 1 | -2.29348777253993 | 0.578844562946069 |
| 4008 | SLC12A2 | 7 | 1 | -3.42423580606349 | -3.43198190552164 |
| 4009 | FBN2 | 14 | 1 | -5.05751059968837 | 0.392873302875268 |
| 4010 | SLC27A6 | 4 | 1 | -4.16305445154078 | 0.0515649475438474 |
| 4011 | ISOC1 | 17 | 1 | 2.0901286749088 | -0.472215368570578 |
| 4012 | ADAMTS19 | 11 | 1 | -4.02409504373439 | -1.7282504878657 |
| 4013 | HINT1 | 13 | 1 | -0.231182804525568 | -2.48889194351603 |
| 4014 | LYRM7 | 9 | 1 | 1.18132688085668 | 2.07041420119833 |
| 4015 | CDC42SE2 | 9 | 1 | 0.557039871617482 | 2.80526842254232 |
| 4016 | RAPGEF6 | 1 | 1 | 4.12247778455846 | 1.92958511489462 |
| 4017 | FNIP1 | 1 | 1 | 3.25062157194249 | 1.15811385291647 |
| 4018 | ACSL6 | 18 | 1 | 5.475277676984 | -0.432790680946601 |
| 4019 | P4HA2 | 14 | 1 | -5.49711440523036 | -0.242127343239081 |

| 4020 | PDLIM4 | 7 | 1 | -2.43189428766139 | -3.35717759949137 |
| --- | --- | --- | --- | --- | --- |
| 4021 | IRF1 | 14 | 1 | -5.75094865281947 | 0.0706222214085935 |
| 4022 | RAD50 | 12 | 1 | 1.89695383588903 | 2.74079002517294 |
| 4023 | KIF3A | 5 | 1 | 4.13467289487951 | -0.671833976330054 |
| 4024 | Sep-08 | 15 | 1 | 5.06611944715612 | -0.326885624946845 |
| 4025 | SHROOM1 | 10 | 1 | 4.45587708036535 | -2.01105008942057 |
| 4026 | GDF9 | 17 | 1 | 0.712307467862294 | 0.187155739246118 |
| 4027 | UQCRQ | 6 | 1 | 0.117971941634343 | -2.33625172001292 |
| 4028 | LEAP2 | 8 | 1 | -1.86277435739405 | 1.42039407867025 |
| 4029 | AFF4 | 9 | 1 | 0.595195069714711 | 2.59208168166708 |
| 4030 | HSPA4 | 2 | 1 | 0.031926736041234 | 2.30085052627157 |
| 4031 | FSTL4 | 18 | 1 | 5.95066143552892 | -2.06840418201853 |
| 4032 | C5orf15 | 8 | 1 | -1.38927839715846 | -0.290221854271186 |
| 4033 | VDAC1 | 13 | 1 | -0.998428866461589 | -1.69964228493143 |
| 4034 | TCF7 | 1 | 1 | 1.40606679002874 | 1.26587905067037 |
| 4035 | SKP1 | 6 | 1 | 0.000604197188542321 | -2.28966055733134 |
| 4036 | PPP2CA | 2 | 1 | 0.592148616715596 | 1.68075169700216 |
| 4037 | AC104109.2 | 11 | 1 | -3.94495390375025 | -1.26277820688654 |
| 4038 | UBE2B | 13 | 1 | -0.287743266866281 | -0.670796631993544 |
| 4039 | CDKN2AIPNL | 13 | 1 | -0.561100140408351 | -0.880326534779561 |
| 4040 | JADE2 | 15 | 1 | 6.02810169736974 | 0.496343866763818 |
| 4041 | SAR1B | 2 | 1 | 1.46042789499395 | 0.932945267139184 |
| 4042 | SEC24A | 12 | 1 | 2.10556055585973 | 2.50429524558614 |
| 4043 | CAMLG | 5 | 1 | 4.32195545713537 | -0.702087789000762 |
| 4044 | DDX46 | 8 | 1 | -1.50655017812617 | 0.474766270099389 |
| 4045 | C5orf24 | 12 | 1 | 2.59453321973913 | 2.56729258674215 |
| 4046 | TXNDC15 | 10 | 1 | 3.67668439428441 | -1.36479832154681 |
| 4047 | PCBD2 | 4 | 1 | -3.9038362355984 | -0.251230760635626 |
| 4048 | C5orf66 | 8 | 1 | -1.4398504348553 | 1.76621593612264 |
| 4049 | H2AFY | 3 | 1 | -2.75329445321925 | -0.892862979480815 |
| 4050 | NEUROG1 | 7 | 1 | -3.38522551973231 | -3.36084447723795 |
| 4051 | CXCL14 | 4 | 1 | -4.01349733789332 | -0.789994604112399 |
| 4052 | TGFBI | 14 | 1 | -6.06620238740809 | 0.185571805415857 |
| 4053 | SMAD5 | 9 | 1 | -0.35900661368735 | 2.49002255576681 |
| 4054 | SMIM32 | 15 | 1 | 6.0806186346256 | 0.543536440311181 |
| 4055 | TRPC7-AS1 | 13 | 1 | -0.189341634706332 | -3.26133547645975 |
| 4056 | SPOCK1 | 18 | 1 | 5.78135944883458 | -2.2642634950297 |
| 4057 | KLHL3 | 1 | 1 | 3.18893195669286 | 1.00629128593038 |
| 4058 | HNRNPA0 | 8 | 1 | -1.45530055482753 | 0.52254606860708 |
| 4059 | FAM13B | 9 | 1 | 1.08622075597875 | 3.067784921108 |
| 4060 | NME5 | 6 | 1 | 1.77633024732702 | -1.10046008151938 |
| 4061 | BRD8 | 16 | 1 | -3.79462359865077 | 2.8027793325765 |
| 4062 | KIF20A | 16 | 1 | -4.31076978166468 | 3.33091057914327 |
| 4063 | CDC23 | 8 | 1 | -3.00146435220607 | 1.75035657065939 |
| 4064 | CDC25C | 16 | 1 | -4.56493972261317 | 3.18206061500143 |
| 4065 | FAM53C | 11 | 1 | -3.30375932176478 | -1.12473969084193 |
| 4066 | KDM3B | 9 | 1 | 0.114129379197286 | 2.68077005523275 |
| 4067 | REEP2 | 10 | 1 | 4.21528746168248 | -1.21842159253527 |
| 4068 | EGR1 | 14 | 1 | -5.90700696428187 | 0.260599390445459 |
| 4069 | ETF1 | 3 | 1 | -2.51100800950892 | 0.437813774524438 |
| 4070 | HSPA9 | 3 | 1 | -1.86629007776149 | 0.00332762378286111 |
| 4071 | CTNNA1 | 7 | 1 | -3.70850537736781 | -2.84844969135691 |
| 4072 | AC034243.1 | 11 | 1 | -4.00449822862513 | -2.28302369934489 |
| 4073 | LRRTM2 | 12 | 1 | 2.86287809888952 | 2.6966887869222 |
| 4074 | SIL1 | 11 | 1 | -2.97475384195216 | -1.2884414039271 |
| 4075 | MATR3.1 | 12 | 1 | 1.81000043432348 | 2.62482632773947 |
| 4076 | PAIP2 | 3 | 1 | -3.37189696748622 | -1.01788451445271 |
| 4077 | SPATA24 | 11 | 1 | -2.66231463868983 | -1.79117982012202 |
| 4078 | DNAJC18 | 12 | 1 | 1.73461867849462 | 2.0606078066213 |
| 4079 | UBE2D2 | 6 | 1 | 1.02071644346349 | -1.98868940693308 |
| 4080 | CXXC5 | 5 | 1 | 3.91318251173131 | -0.553524061264289 |
| 4081 | PSD2 | 15 | 1 | 4.78564144651525 | 0.232452408252466 |
| 4082 | NRG2 | 15 | 1 | 4.63002182523839 | -0.0832994423525454 |
| 4083 | PURA | 1 | 1 | 3.01277877370946 | 1.68388952392172 |
| 4084 | IGIP | 1 | 1 | 3.73448730985753 | 1.1484550394399 |
| 4085 | CYSTM1 | 6 | 1 | 0.830591574116872 | -2.76892052513529 |
| 4086 | PFDN1 | 13 | 1 | -1.08603237588771 | -0.987175985397589 |

| 4087 | AC008438.1 | 14 | 1 | -5.94047854860194 | -0.307355805458319 |
| --- | --- | --- | --- | --- | --- |
| 4088 | ANKHD1 | 8 | 1 | -1.72342382390864 | 1.53679598945211 |
| 4089 | ANKHD1-EIF4EBP3 | 9 | 1 | 0.361384585305379 | 2.77174557822775 |
| 4090 | SRA1 | 13 | 1 | -2.1776707025326 | -1.5627356610911 |
| 4091 | APBB3 | 10 | 1 | 4.31171728651159 | -1.0912321500199 |
| 4092 | SLC35A4 | 3 | 1 | -1.83653508146174 | -0.285245164455664 |
| 4093 | NDUFA2 | 13 | 1 | -0.562749222115352 | -2.11932872158457 |
| 4094 | IK | 2 | 1 | 0.0391493586742143 | 1.11455310958456 |
| 4095 | DND1 | 1 | 1 | 2.43910337965123 | 0.695123330531824 |
| 4096 | HARS | 6 | 1 | 0.917787805005238 | -0.983593917609942 |
| 4097 | ZMAT2 | 8 | 1 | -0.573892072037532 | 0.451857701717126 |
| 4098 | PCDHA2 | 1 | 1 | 3.15604998151891 | 1.47407068389486 |
| 4099 | PCDHA4 | 9 | 1 | 1.32362653295629 | 2.71696724074911 |
| 4100 | PCDHA5 | 12 | 1 | 2.39360071699254 | 2.04225029128622 |
| 4101 | PCDHA6 | 12 | 1 | 3.5334777979099 | 2.7587906278951 |
| 4102 | PCDHA9 | 12 | 1 | 2.69751955549352 | 2.03601946013998 |
| 4103 | PCDHA10 | 12 | 1 | 2.17058803121679 | 2.48615230697225 |
| 4104 | PCDHA11 | 2 | 1 | 0.845754995747731 | 2.07464613097738 |
| 4105 | PCDHA12 | 5 | 1 | 3.15603400747411 | -0.026772244991553 |
| 4106 | PCDHAC1 | 12 | 1 | 2.09882809202306 | 2.76750315803121 |
| 4107 | PCDHAC2 | 1 | 1 | 2.91506148855321 | 1.94559539931844 |
| 4108 | PCDHB2 | 9 | 1 | 0.903354063435719 | 2.22238673347067 |
| 4109 | PCDHB3 | 12 | 1 | 1.77496053259008 | 2.69303001540731 |
| 4110 | AC244517.1 | 12 | 1 | 1.92005445043676 | 2.06755055564474 |
| 4111 | PCDHB4 | 12 | 1 | 1.97238661329381 | 2.38948334830831 |
| 4112 | PCDHB5 | 9 | 1 | 0.713626578255818 | 2.84437765258383 |
| 4113 | PCDHB7 | 12 | 1 | 2.46321369688146 | 2.48246969359945 |
| 4114 | PCDHB16 | 12 | 1 | 1.74589861909978 | 1.97150696891378 |
| 4115 | PCDHB9 | 12 | 1 | 1.94715715925329 | 2.14695347922872 |
| 4116 | PCDHB10 | 2 | 1 | -0.495277568892314 | 1.75678266662191 |
| 4117 | PCDHB11 | 7 | 1 | -2.81529639680751 | -2.6954432569163 |
| 4118 | PCDHB12 | 2 | 1 | 0.710449054642842 | 2.27649296897482 |
| 4119 | PCDHB13 | 9 | 1 | 0.845535412236379 | 2.41753424781393 |
| 4120 | PCDHB14 | 9 | 1 | 1.11509527246587 | 2.29575456756185 |
| 4121 | PCDHB15 | 11 | 1 | -3.56299756486781 | -2.23481820446421 |
| 4122 | TAF7 | 3 | 1 | -1.86419330556758 | 0.139002219615686 |
| 4123 | PCDHGA1 | 7 | 1 | -3.66911982019313 | -3.11317168098856 |
| 4124 | PCDHGA2 | 8 | 1 | -1.68143330533869 | 1.5864559568746 |
| 4125 | PCDHGA3 | 7 | 1 | -3.59518907983668 | -2.91486869675089 |
| 4126 | PCDHGA6 | 11 | 1 | -3.61096547563441 | -2.25608430725504 |
| 4127 | PCDHGB6 | 1 | 1 | 3.01851893941991 | 1.10151447433065 |
| 4128 | PCDHGA10 | 17 | 1 | 1.5825785545551 | 0.315613285480249 |
| 4129 | PCDHGC3 | 8 | 1 | -1.2837076040066 | 1.27717937606405 |
| 4130 | DIAPH1 | 2 | 1 | -0.851260468558147 | 2.72561730521749 |
| 4131 | HDAC3 | 3 | 1 | -2.00401006658442 | -0.380341394962561 |
| 4132 | RELL2 | 10 | 1 | 4.46982647459142 | -0.936183347524893 |
| 4133 | ARAP3 | 4 | 1 | -4.95816825349696 | -2.19048450809885 |
| 4134 | PCDH1 | 1 | 1 | 3.9946606306278 | 1.32670344489645 |
| 4135 | DELE1 | 2 | 1 | 0.0716410724364976 | 2.32627596038412 |
| 4136 | RNF14 | 12 | 1 | 2.35143591444127 | 2.18913926261495 |
| 4137 | GNPDA1 | 4 | 1 | -5.01330350358851 | -1.88751344305445 |
| 4138 | NDFIP1 | 10 | 1 | 2.94835330526464 | -1.75083838325907 |
| 4139 | SPRY4 | 14 | 1 | -6.67194198091395 | -0.440582021297705 |
| 4140 | NR3C1 | 14 | 1 | -5.76911996324427 | 0.231693402705896 |
| 4141 | YIPF5 | 2 | 1 | 1.3832297472202 | 0.960442201076257 |
| 4142 | KCTD16 | 1 | 1 | 4.88070560972326 | 1.18322385924886 |
| 4143 | PRELID2 | 4 | 1 | -4.47587417085536 | 0.432441369472253 |
| 4144 | SH3RF2 | 16 | 1 | -4.15820120294459 | 2.80953134673666 |
| 4145 | LARS | 3 | 1 | -2.3867668958462 | 0.549708024440515 |
| 4146 | AC091959.3 | 8 | 1 | -0.922595843867137 | 0.666481629787195 |
| 4147 | RBM27 | 9 | 1 | 0.384892716809438 | 2.88250817435812 |
| 4148 | TCERG1 | 2 | 1 | -0.97552047689326 | 1.66964663642477 |
| 4149 | PPP2R2B | 10 | 1 | 4.13664127866857 | -1.22606582862307 |
| 4150 | STK32A | 6 | 1 | 1.71422792951696 | -2.87354634624888 |
| 4151 | DPYSL3 | 1 | 1 | 3.68496729414098 | 0.665562764583337 |
| 4152 | JAKMIP2 | 1 | 1 | 4.04352833311193 | 0.716777101932275 |
| 4153 | AC091948.1 | 15 | 1 | 4.84699036161535 | 0.442750827251184 |

| 4154 | FBXO38 | 2 | 1 | 0.568578377171681 | 1.88601912635397 |
| --- | --- | --- | --- | --- | --- |
| 4155 | AC114939.1 | 9 | 1 | 0.645898595257924 | 2.5357955850942 |
| 4156 | ABLIM3 | 18 | 1 | 6.00807835142247 | -1.77714405876566 |
| 4157 | GRPEL2 | 12 | 1 | 2.46144248525731 | 1.92244042533468 |
| 4158 | CSNK1A1 | 2 | 1 | 0.129531845256017 | 1.4122399248464 |
| 4159 | ARHGEF37 | 3 | 1 | -3.2725639196194 | 0.240710274158227 |
| 4160 | SLC26A2 | 9 | 1 | 1.75617100278966 | 3.11272086280416 |
| 4161 | TIGD6 | 8 | 1 | -0.87132148464091 | 0.37813414710592 |
| 4162 | HMGXB3 | 2 | 1 | 0.163813367291615 | 2.25081171172689 |
| 4163 | CAMK2A | 15 | 1 | 5.0157559065067 | 0.640818373141992 |
| 4164 | ARSI | 4 | 1 | -3.98997877557643 | -0.772267117323172 |
| 4165 | TCOF1 | 8 | 1 | -1.57902131994136 | 1.51888336318563 |
| 4166 | RPS14 | 21 | 1 | -0.889328524664714 | -4.44125471931864 |
| 4167 | NDST1-AS1 | 11 | 1 | -4.23999093492396 | -1.62019888979365 |
| 4168 | NDST1 | 2 | 1 | -0.510489538506343 | 2.0367242254598 |
| 4169 | RBM22 | 8 | 1 | -0.837187871531322 | 0.361412660060632 |
| 4170 | DCTN4 | 12 | 1 | 2.46742416898839 | 2.25412215369772 |
| 4171 | SMIM3 | 14 | 1 | -5.24499391038783 | 0.278113976894128 |
| 4172 | ZNF300 | 1 | 1 | 4.19387985746495 | 1.99647392409872 |
| 4173 | TNIP1 | 3 | 1 | -2.71313999612696 | -0.847074738861811 |
| 4174 | ANXA6 | 4 | 1 | -4.33617137391932 | -1.14424589198996 |
| 4175 | CCDC69 | 14 | 1 | -5.40202878435023 | -0.514901622356665 |
| 4176 | SLC36A1 | 12 | 1 | 2.33423759023778 | 2.16213335174154 |
| 4177 | SPARC | 4 | 1 | -4.4459025712765 | -0.829434259952796 |
| 4178 | AC011374.2 | 1 | 1 | 2.83073426763646 | 0.494731441913354 |
| 4179 | ATOX1 | 6 | 1 | 1.55910028497808 | -2.25446830612589 |
| 4180 | G3BP1 | 3 | 1 | -2.80030058343775 | 0.853657261310327 |
| 4181 | LINC01933 | 20 | 1 | 3.46412755529516 | -3.61852990013529 |
| 4182 | GRIA1 | 15 | 1 | 5.2251064924442 | -0.125070317806494 |
| 4183 | FAM114A2 | 2 | 1 | 0.368207171364949 | 1.65366233962606 |
| 4184 | GALNT10 | 1 | 1 | 2.77538777868383 | 1.57302226203512 |
| 4185 | SAP30L-AS1 | 8 | 1 | -1.31776700933345 | 1.868730441509 |
| 4186 | SAP30L | 8 | 1 | -1.9251905532635 | 0.89819087165426 |
| 4187 | LARP1 | 12 | 1 | 2.58372952024572 | 2.47736443656515 |
| 4188 | FAXDC2 | 11 | 1 | -3.65573047120936 | -1.6778890929835 |
| 4189 | CNOT8 | 9 | 1 | 0.610184982224629 | 2.43900956290792 |
| 4190 | GEMIN5 | 8 | 1 | -1.47501765210993 | 1.39372028487753 |
| 4191 | MRPL22 | 3 | 1 | -2.52487681825526 | -1.17418475371767 |
| 4192 | SGCD | 10 | 1 | 4.812680020734 | -1.73534159284998 |
| 4193 | MED7 | 8 | 1 | -1.29250477273829 | 0.242540136752832 |
| 4194 | CYFIP2 | 1 | 1 | 4.17033220808141 | 1.44061078208517 |
| 4195 | FNDC9 | 15 | 1 | 5.67914511243932 | 0.923198119579065 |
| 4196 | ADAM19 | 7 | 1 | -2.53794668634303 | -3.317863806309 |
| 4197 | THG1L | 11 | 1 | -3.54208849389918 | -1.78422723633219 |
| 4198 | LSM11 | 1 | 1 | 4.77217508833043 | 0.706587449489343 |
| 4199 | CLINT1 | 3 | 1 | -2.76217220743067 | 0.0494297899587034 |
| 4200 | EBF1 | 20 | 1 | 3.02829767744176 | -3.29548393112589 |
| 4201 | AC136424.2 | 7 | 1 | -2.13857053239711 | -3.45297895294596 |
| 4202 | LINC02202 | 7 | 1 | -2.43149112184413 | -3.84621773582865 |
| 4203 | RNF145 | 6 | 1 | 2.31047774831884 | -2.28021870476176 |
| 4204 | UBLCP1 | 3 | 1 | -2.93686889131434 | -0.512077941717398 |
| 4205 | TTC1 | 6 | 1 | -0.15262465943463 | -0.961781467469227 |
| 4206 | PWWP2A | 1 | 1 | 4.26687313596837 | 1.02848590987753 |
| 4207 | CCNJL | 11 | 1 | -3.68370817621119 | -2.42187379223276 |
| 4208 | SLU7 | 2 | 1 | 0.0502617208205919 | 1.50015916007589 |
| 4209 | PTTG1 | 16 | 1 | -4.40329240281947 | 2.47946561950277 |
| 4210 | GABRB2 | 18 | 1 | 6.28180672208898 | -2.36961911541392 |
| 4211 | GABRA1 | 18 | 1 | 5.59977890531652 | -0.397326274933112 |
| 4212 | GABRG2 | 10 | 1 | 4.35853601972692 | -1.49963931662012 |
| 4213 | CCNG1 | 11 | 1 | -3.23598454912074 | -2.02687130791117 |
| 4214 | NUDCD2 | 16 | 1 | -4.4164368959225 | 2.54162968772482 |
| 4215 | HMMR | 16 | 1 | -4.4020736070431 | 2.87913955825399 |
| 4216 | MAT2B | 4 | 1 | -3.58196829278834 | -0.210955484928382 |
| 4217 | AC109466.1 | 14 | 1 | -6.6258079858578 | -1.06817902547289 |
| 4218 | TENM2 | 18 | 1 | 5.57555891553991 | -1.40071843487192 |
| 4219 | AC008708.2 | 12 | 1 | 2.28783036748998 | 2.28900732177328 |
| 4220 | AC008637.1 | 11 | 1 | -3.59542892892726 | -1.1680768869536 |

| 4221 | WWC1 | 4 | 1 | -5.42680428941615 | -1.34382916909624 |
| --- | --- | --- | --- | --- | --- |
| 4222 | RARS | 8 | 1 | -0.944194421366527 | 0.306786195216882 |
| 4223 | FBLL1 | 10 | 1 | 4.40350319425695 | -1.10386197191645 |
| 4224 | PANK3 | 9 | 1 | 1.943943276807 | 2.99210299628805 |
| 4225 | SLIT3 | 10 | 1 | 4.75978924314611 | -1.60628990751673 |
| 4226 | SPDL1 | 16 | 1 | -4.14912627656825 | 2.98178924697469 |
| 4227 | KCNIP1 | 18 | 1 | 5.92131830732457 | -1.64603005272318 |
| 4228 | RANBP17 | 1 | 1 | 3.85774780790441 | 2.12476529258322 |
| 4229 | AC091980.2 | 20 | 1 | 3.380879655286 | -3.72665940147806 |
| 4230 | TLX3 | 20 | 1 | 3.30069948713414 | -3.83053456169535 |
| 4231 | NPM1 | 11 | 1 | -3.10214208085902 | -1.29594461542536 |
| 4232 | FGF18 | 7 | 1 | -3.16623805482753 | -2.4394880376475 |
| 4233 | FBXW11 | 5 | 1 | 3.35900403539769 | -0.195281847538245 |
| 4234 | STK10 | 6 | 1 | 2.23894692937963 | -1.35107253414561 |
| 4235 | UBTD2 | 8 | 1 | -1.22383515556224 | 1.53706492560934 |
| 4236 | SH3PXD2B | 7 | 1 | -3.01891277749903 | -2.53217993599345 |
| 4237 | NEURL1B | 7 | 1 | -3.55113933999903 | -3.53300033432413 |
| 4238 | DUSP1 | 17 | 1 | 0.154737487240956 | -0.258892878116858 |
| 4239 | ERGIC1 | 12 | 1 | 2.22705246488683 | 2.35603822845052 |
| 4240 | AC008429.3 | 7 | 1 | -3.57648395021327 | -3.06845770698954 |
| 4241 | RPL26L1 | 13 | 1 | -2.20563446004756 | -1.49050860029627 |
| 4242 | ATP6V0E1 | 7 | 1 | -2.11735259015925 | -2.65873919350077 |
| 4243 | CREBRF | 12 | 1 | 2.15267707388036 | 2.49824823516439 |
| 4244 | BNIP1 | 4 | 1 | -4.91738627870448 | -0.727819725097907 |
| 4245 | STC2 | 14 | 1 | -5.64065764863856 | 0.249184862552392 |
| 4246 | BOD1 | 3 | 1 | -1.81523321588404 | -0.16299281937052 |
| 4247 | CPEB4 | 1 | 1 | 2.72465755025976 | 1.34703411239217 |
| 4248 | NSG2 | 15 | 1 | 4.81425167600744 | -0.0147753201143862 |
| 4249 | DRD1 | 4 | 1 | -4.84262822587855 | -1.6025231323855 |
| 4250 | SFXN1 | 9 | 1 | 0.822945967122243 | 2.59951461928915 |
| 4251 | HRH2 | 7 | 1 | -3.26827834566004 | -3.53838358742167 |
| 4252 | CPLX2 | 7 | 1 | -2.65499900300868 | -3.9836877427714 |
| 4253 | THOC3 | 3 | 1 | -3.40628002603419 | 0.430371061740625 |
| 4254 | SIMC1 | 2 | 1 | -0.179495483831241 | 1.73262514251302 |
| 4255 | KIAA1191 | 8 | 1 | -0.977732524470164 | 0.0621332682950376 |
| 4256 | ARL10 | 1 | 1 | 3.51447774450414 | 2.27911104339193 |
| 4257 | NOP16 | 3 | 1 | -2.55835746248133 | -0.424302651705039 |
| 4258 | HIGD2A | 13 | 1 | -1.49248681981929 | -2.36769150120188 |
| 4259 | CLTB | 10 | 1 | 3.46726228277318 | -1.8054776034968 |
| 4260 | FAF2 | 8 | 1 | -1.33010886628993 | 0.424685255466211 |
| 4261 | RNF44 | 2 | 1 | -1.13539223392375 | 1.95396341460775 |
| 4262 | GPRIN1 | 15 | 1 | 5.05628468076818 | -0.217818065704596 |
| 4263 | LINC01574 | 18 | 1 | 5.11417891065709 | -1.23253531676699 |
| 4264 | UNC5A | 7 | 1 | -2.64099214990504 | -3.70805083137919 |
| 4265 | ZNF346 | 2 | 1 | 0.788746848508046 | 2.0939375318868 |
| 4266 | FGFR4 | 1 | 1 | 3.52294851820104 | 2.01154197829794 |
| 4267 | NSD1 | 9 | 1 | 1.3821220545017 | 2.65526522773336 |
| 4268 | MXD3 | 16 | 1 | -4.69915793855555 | 3.11911858695577 |
| 4269 | PRELID1 | 13 | 1 | -2.13479481656916 | -1.36006788832117 |
| 4270 | LMAN2 | 3 | 1 | -1.84439359624751 | -0.998508854927313 |
| 4271 | RGS14 | 7 | 1 | -2.6862099023617 | -2.13456152302195 |
| 4272 | F12 | 10 | 1 | 4.12408186475866 | -1.45858107429911 |
| 4273 | GRK6 | 17 | 1 | 1.43262256662481 | -0.486214711488974 |
| 4274 | PRR7 | 5 | 1 | 4.31866480390661 | -0.715841113747847 |
| 4275 | DBN1 | 1 | 1 | 1.27400304357641 | 1.60606826919149 |
| 4276 | PDLIM7 | 13 | 1 | -1.52531586606867 | -2.75303469520975 |
| 4277 | DDX41 | 17 | 1 | 0.205835267707036 | -0.0186208329813601 |
| 4278 | FAM193B | 2 | 1 | 1.30674780885808 | 0.85124947207998 |
| 4279 | AC139795.3 | 4 | 1 | -3.91543458421595 | 0.132917300639856 |
| 4280 | TMED9 | 3 | 1 | -2.17408715207942 | -0.652593850316298 |
| 4281 | B4GALT7 | 3 | 1 | -1.81280241926081 | -0.685462027014983 |
| 4282 | N4BP3 | 14 | 1 | -5.18969891984828 | 0.174855605540979 |
| 4283 | RMND5B | 2 | 1 | 0.88236059229009 | 1.3085440554006 |
| 4284 | NHP2 | 3 | 1 | -2.62770269830592 | -0.598832591594947 |
| 4285 | HNRNPAB | 3 | 1 | -2.88934777696498 | 0.477665082393396 |
| 4286 | PHYKPL | 11 | 1 | -3.16038511712916 | -2.18584929329325 |
| 4287 | CLK4 | 1 | 1 | 4.23934580366246 | 1.41370309966634 |

| 4288 | ZNF354A | 5 | 1 | 3.30952312032811 | -0.238792165340674 |
| --- | --- | --- | --- | --- | --- |
| 4289 | ZNF354C | 9 | 1 | 1.71660758535497 | 2.9702278532369 |
| 4290 | ADAMTS2 | 19 | 1 | -4.4776728006161 | 0.753881946979272 |
| 4291 | RUFY1 | 17 | 1 | 0.491550460263417 | 0.168686286388147 |
| 4292 | HNRNPH1 | 7 | 1 | -2.53528402765162 | -2.24563549381663 |
| 4293 | CANX | 2 | 1 | -0.917175337866618 | 1.69085611480306 |
| 4294 | MAML1 | 8 | 1 | -1.60678564031489 | 1.71958030837606 |
| 4295 | MGAT4B | 6 | 1 | 1.74910975973241 | -1.10351934951712 |
| 4296 | SQSTM1 | 6 | 1 | 2.52771235983007 | -1.53578089100291 |
| 4297 | MRNIP | 10 | 1 | 3.63556410352819 | -1.23607458096911 |
| 4298 | AC008393.1 | 10 | 1 | 3.41109205762975 | -1.22738538605143 |
| 4299 | TBC1D9B | 1 | 1 | 3.0679917482578 | 2.03681267875265 |
| 4300 | RNF130 | 7 | 1 | -3.47178958375819 | -2.77038775307108 |
| 4301 | RASGEF1C | 7 | 1 | -2.69007228334315 | -3.71405349594523 |
| 4302 | MAPK9 | 1 | 1 | 4.36450888197057 | 0.995325938640344 |
| 4303 | GFPT2 | 4 | 1 | -4.65561793764003 | -0.288906737388861 |
| 4304 | CNOT6 | 9 | 1 | 0.0738912967883806 | 2.76558865684103 |
| 4305 | SCGB3A1 | 11 | 1 | -4.15728329141505 | -2.0106345258372 |
| 4306 | FLT4 | 19 | 1 | -5.59942220170863 | 1.29244269507955 |
| 4307 | MGAT1 | 2 | 1 | -0.299621079311444 | 1.24916590827535 |
| 4308 | AC008443.5 | 4 | 1 | -4.23654387910731 | 0.0667964376790403 |
| 4309 | TRIM7 | 6 | 1 | 1.98927785436742 | -1.23838300925661 |
| 4310 | TRIM41 | 8 | 1 | -1.18083702047236 | 1.24232043403219 |
| 4311 | RACK1 | 21 | 1 | -1.03125576694377 | -4.42175041061808 |
| 4312 | TRIM52 | 12 | 1 | 2.90495564024083 | 2.66885174888204 |
| 4313 | AC008443.3 | 9 | 1 | 1.22695960085027 | 3.01500214713644 |
| 4314 | DUSP22 | 2 | 1 | 1.07014502565496 | 1.44609464782308 |
| 4315 | EXOC2 | 1 | 1 | 3.18424774686925 | 2.16531027930807 |
| 4316 | GMDS | 4 | 1 | -5.00559972246058 | -0.107299610199225 |
| 4317 | GMDS-DT | 4 | 1 | -4.24005006273158 | -0.363279803814185 |
| 4318 | WRNIP1 | 12 | 1 | 2.10175468008153 | 1.9720748819692 |
| 4319 | SERPINB1 | 7 | 1 | -2.34228418786891 | -2.42879103046824 |
| 4320 | SERPINB9 | 10 | 1 | 4.46068789045446 | -2.12357066494395 |
| 4321 | SERPINB6 | 11 | 1 | -3.64482496698268 | -1.77789358717371 |
| 4322 | LINC01011 | 15 | 1 | 5.34222723524205 | 0.26637627261709 |
| 4323 | NQO2 | 10 | 1 | 4.16574980299108 | -1.43397425037791 |
| 4324 | RIPK1 | 8 | 1 | -2.12500916440852 | 1.93526949065756 |
| 4325 | AL031963.3 | 3 | 1 | -3.51258419473536 | 0.371759191928613 |
| 4326 | BPHL | 7 | 1 | -2.13952396829493 | -2.61983345371653 |
| 4327 | TUBB2A | 10 | 1 | 3.52628399412267 | -1.81019686085154 |
| 4328 | TUBB2B | 10 | 1 | 3.9304287580692 | -1.62581013065745 |
| 4329 | PSMG4 | 2 | 1 | 0.14394979159467 | 2.38203825133871 |
| 4330 | SLC22A23 | 7 | 1 | -2.29249762018092 | -3.64896927696635 |
| 4331 | PXDC1 | 14 | 1 | -6.65971635301478 | -0.508503033937705 |
| 4332 | AL391422.3 | 14 | 1 | -6.73246453721888 | -0.457657619537604 |
| 4333 | AL138831.2 | 11 | 1 | -4.04773925264247 | -2.24865554195811 |
| 4334 | PRPF4B | 8 | 1 | -1.85610841234095 | 0.878573314128625 |
| 4335 | ECI2 | 4 | 1 | -4.35264609773524 | -1.19973851663042 |
| 4336 | CDYL | 4 | 1 | -3.4232077451504 | -0.300063534797919 |
| 4337 | RPP40 | 4 | 1 | -3.51794718225367 | -0.538776233019126 |
| 4338 | PPP1R3G | 7 | 1 | -2.70056794603236 | -2.68253730160166 |
| 4339 | LYRM4 | 7 | 1 | -2.46465943773158 | -2.45074294430186 |
| 4340 | FARS2 | 7 | 1 | -2.56192611177333 | -2.77618847710062 |
| 4341 | NRN1 | 5 | 1 | 4.05808188001745 | -0.756344243110907 |
| 4342 | F13A1 | 7 | 1 | -1.8915778251446 | -3.51396261078287 |
| 4343 | LY86-AS1 | 7 | 1 | -3.19496296365626 | -3.52812085968424 |
| 4344 | RREB1 | 14 | 1 | -5.72655175645716 | -0.200952573837531 |
| 4345 | SSR1 | 1 | 1 | 3.73716689626806 | 0.935319200931299 |
| 4346 | CAGE1 | 16 | 1 | -4.29641698320277 | 2.5720549978597 |
| 4347 | RIOK1 | 2 | 1 | 0.678994849129842 | 0.8299747862203 |
| 4348 | AL031058.1 | 4 | 1 | -4.20244239290126 | -0.854116070242417 |
| 4349 | DSP | 4 | 1 | -4.28522084672816 | -0.942801422597659 |
| 4350 | SNRNP48 | 3 | 1 | -3.52659247835047 | 0.704123870311487 |
| 4351 | BLOC1S5 | 3 | 1 | -1.79097555597193 | 0.00854219573568094 |
| 4352 | EEF1E1 | 6 | 1 | 1.41441859285466 | -1.16337241155077 |
| 4353 | SLC35B3 | 3 | 1 | -2.37194441278346 | -0.513929291786444 |
| 4354 | TFAP2A | 7 | 1 | -2.520003780917 | -3.72653661590983 |

| 4355 | GCNT2 | 1 | 1 | 4.29272152464025 | 1.49014081138204 |
| --- | --- | --- | --- | --- | --- |
| 4356 | C6orf52 | 11 | 1 | -2.89965151269801 | -1.47094558102061 |
| 4357 | PAK1IP1 | 8 | 1 | -1.80998407323725 | 0.319524184642541 |
| 4358 | TMEM14C | 13 | 1 | -1.92299996335871 | -2.05366443020273 |
| 4359 | TMEM14B | 13 | 1 | -0.342904620066955 | -2.27361939770151 |
| 4360 | MAK | 1 | 1 | 1.36060692350499 | 1.41597546714376 |
| 4361 | SYCP2L | 11 | 1 | -3.56289647539027 | -1.80830316168238 |
| 4362 | ELOVL2 | 19 | 1 | -5.14556954820521 | 1.44319714683126 |
| 4363 | SMIM13 | 1 | 1 | 4.66176821272008 | 0.703745380817163 |
| 4364 | NEDD9 | 14 | 1 | -6.593814119891 | -0.407217457117331 |
| 4365 | TMEM170B | 1 | 1 | 3.27334214727514 | 1.06567503589224 |
| 4366 | HIVEP1 | 1 | 1 | 3.65720726530187 | 0.780480281291711 |
| 4367 | PHACTR1 | 5 | 1 | 4.0809791235172 | -0.101811452926886 |
| 4368 | TBC1D7 | 3 | 1 | -2.77202890832789 | -1.25025926453043 |
| 4369 | AL008729.2 | 1 | 1 | 2.21338011304967 | 0.839965716777551 |
| 4370 | GFOD1 | 15 | 1 | 4.81350971738927 | 0.451443687854516 |
| 4371 | SIRT5 | 8 | 1 | -1.37997733075984 | 1.10855831283163 |
| 4372 | AL441883.1 | 4 | 1 | -3.73250149209864 | -0.518401249470008 |
| 4373 | NOL7 | 3 | 1 | -2.85487030465968 | -0.684885486187232 |
| 4374 | RANBP9 | 1 | 1 | 3.04440738241308 | 2.0318311609609 |
| 4375 | MCUR1 | 6 | 1 | 1.54518605749242 | -2.00546799522806 |
| 4376 | RNF182 | 5 | 1 | 4.03494574110143 | -0.47367985469271 |
| 4377 | CD83 | 14 | 1 | -5.92042706926234 | 0.346390859065759 |
| 4378 | JARID2 | 7 | 1 | -3.19576953370936 | -2.92835102898051 |
| 4379 | DTNBP1 | 7 | 1 | -2.76495312173732 | -3.67908845764567 |
| 4380 | MYLIP | 7 | 1 | -2.7584721895016 | -3.6586681447642 |
| 4381 | ATXN1 | 18 | 1 | 5.24003006498449 | -0.506592943491233 |
| 4382 | RBM24 | 11 | 1 | -3.17289946992762 | -2.35091255527903 |
| 4383 | CAP2 | 6 | 1 | 0.926691427632497 | -0.730699806632292 |
| 4384 | FAM8A1 | 1 | 1 | 3.58329345266454 | 1.82479395049642 |
| 4385 | NUP153 | 9 | 1 | 0.139345720215962 | 2.80666722434591 |
| 4386 | KIF13A | 2 | 1 | -1.00101386030085 | 1.42600669044088 |
| 4387 | TPMT | 5 | 1 | 2.2748656419956 | -0.499073400320304 |
| 4388 | KDM1B | 5 | 1 | 3.13775731603734 | 0.379502431331384 |
| 4389 | DEK | 16 | 1 | -4.13238714654811 | 1.93952001708578 |
| 4390 | RNF144B | 3 | 1 | -2.99539040048487 | 0.125604049144494 |
| 4391 | AL022068.1 | 4 | 1 | -4.34055303056605 | -0.867292667897236 |
| 4392 | ID4 | 4 | 1 | -4.32929442842372 | 0.0925188817365049 |
| 4393 | E2F3 | 3 | 1 | -3.27514146287806 | 0.638963715015161 |
| 4394 | CDKAL1 | 8 | 1 | -1.27331195791133 | 0.90205468314718 |
| 4395 | AL512380.1 | 18 | 1 | 5.88006521741979 | -2.02517710548807 |
| 4396 | SOX4 | 18 | 1 | 5.62338329832189 | -0.852533071460021 |
| 4397 | CASC15 | 7 | 1 | -2.87615846116908 | -2.54135654789377 |
| 4398 | NBAT1 | 7 | 1 | -2.84634707887538 | -2.55696223598887 |
| 4399 | NRSN1 | 15 | 1 | 4.85598541776769 | 0.333426610408532 |
| 4400 | MRS2 | 1 | 1 | 1.26324070970647 | 1.91640271323751 |
| 4401 | GPLD1 | 1 | 1 | 4.98821379224889 | 0.849651829181421 |
| 4402 | ALDH5A1 | 15 | 1 | 4.93652464429967 | 0.678597227512109 |
| 4403 | KIAA0319 | 15 | 1 | 4.63616205732457 | 0.301673547206628 |
| 4404 | TDP2 | 2 | 1 | -0.15457864870198 | 1.32604326385092 |
| 4405 | ACOT13 | 11 | 1 | -3.16323040445216 | -1.77394591194559 |
| 4406 | C6orf62 | 12 | 1 | 2.26591898481481 | 2.08874000686239 |
| 4407 | GMNN | 19 | 1 | -4.93905709703334 | 1.91550888198446 |
| 4408 | RIPOR2 | 18 | 1 | 5.59791637937658 | -1.11523537260462 |
| 4409 | CARMIL1 | 18 | 1 | 5.46813799421422 | -1.63565187078882 |
| 4410 | SCGN | 18 | 1 | 6.29380728284948 | -2.45633397919108 |
| 4411 | HIST1H1A | 19 | 1 | -5.76789115388758 | 1.67872466224264 |
| 4412 | HIST1H3A | 19 | 1 | -5.73413489778407 | 1.66645063537191 |
| 4413 | HIST1H4A | 19 | 1 | -5.69165395219691 | 1.64323271888326 |
| 4414 | HIST1H4B | 19 | 1 | -5.76231645067103 | 1.67919124740194 |
| 4415 | HIST1H3B | 19 | 1 | -5.7343923898495 | 1.65546740668844 |
| 4416 | HIST1H2AB | 19 | 1 | -5.77882073839076 | 1.64834965842794 |
| 4417 | HIST1H2BB | 19 | 1 | -5.77229617555506 | 1.63367857116293 |
| 4418 | HIST1H3C | 19 | 1 | -5.7452885957516 | 1.6497556127889 |
| 4419 | HIST1H1C | 19 | 1 | -5.20285008867152 | 1.0469713367803 |
| 4420 | HIST1H4C | 19 | 1 | -4.89169000108607 | 1.52951921599935 |
| 4421 | HIST1H1T | 19 | 1 | -4.96052239854701 | 2.01753963607382 |

| 4422 | HIST1H2BC | 19 | 1 | -5.75401423890956 | 1.68124140876363 |
| --- | --- | --- | --- | --- | --- |
| 4423 | HIST1H2AC | 17 | 1 | 0.32410772125356 | -0.192263945163977 |
| 4424 | HIST1H1E | 19 | 1 | -5.7525260301388 | 1.63862647193502 |
| 4425 | HIST1H2BD | 1 | 1 | 1.2115761188709 | 1.83918847220968 |
| 4426 | HIST1H2BE | 17 | 1 | 1.24299217741124 | 0.445682064471948 |
| 4427 | HIST1H4D | 19 | 1 | -5.76464389284022 | 1.6754874147756 |
| 4428 | AL031777.3 | 19 | 1 | -5.24276803453334 | 1.41892542022299 |
| 4429 | HIST1H2BF | 19 | 1 | -5.68561719377406 | 1.6067344107015 |
| 4430 | HIST1H4E | 19 | 1 | -5.1492020936764 | 1.64023055213522 |
| 4431 | HIST1H2AE | 6 | 1 | 1.36893905679815 | -2.77710221630503 |
| 4432 | HIST1H3E | 3 | 1 | -3.17599128206141 | 0.349722758708703 |
| 4433 | HIST1H1D | 19 | 1 | -5.79134200532801 | 1.64762605804037 |
| 4434 | HIST1H3F | 19 | 1 | -5.75988267381556 | 1.64615191596578 |
| 4435 | HIST1H2BH | 19 | 1 | -4.27255557496913 | 0.814473763881433 |
| 4436 | HIST1H3G | 19 | 1 | -5.59399198015101 | 1.67078675407003 |
| 4437 | HIST1H2BI | 19 | 1 | -5.69591163118251 | 1.70969428199362 |
| 4438 | HIST1H4H | 19 | 1 | -5.07298110444911 | 1.04061915057729 |
| 4439 | BTN2A2 | 11 | 1 | -3.82371400316127 | -1.89368550402094 |
| 4440 | BTN3A1 | 4 | 1 | -4.60668586213954 | -1.58002440554072 |
| 4441 | BTN2A1 | 3 | 1 | -1.88743935544856 | -0.143783732475531 |
| 4442 | HCG11 | 17 | 1 | -0.0620217027223845 | -0.357789977611792 |
| 4443 | HMGN4 | 3 | 1 | -2.55644820649989 | 0.521119252620447 |
| 4444 | ABT1 | 3 | 1 | -2.15325747449763 | -0.751452564419997 |
| 4445 | AL513548.3 | 9 | 1 | 0.712305202885793 | 2.53956784385275 |
| 4446 | ZNF322 | 1 | 1 | 3.11274982969396 | 0.82684601920675 |
| 4447 | AL513548.1 | 1 | 1 | 2.75578714887731 | 1.37802876609396 |
| 4448 | HIST1H2BJ | 11 | 1 | -3.88486503084071 | -0.912570762606156 |
| 4449 | HIST1H2AG | 19 | 1 | -5.42863343675502 | 1.54094614165853 |
| 4450 | HIST1H4I | 19 | 1 | -5.73750136812098 | 1.5631565012319 |
| 4451 | HIST1H2BK | 19 | 1 | -5.08512996156581 | 0.883288518367517 |
| 4452 | HIST1H2AH | 19 | 1 | -5.74844382722743 | 1.62757100242208 |
| 4453 | PRSS16 | 13 | 1 | -1.70102988202937 | -1.73292194229533 |
| 4454 | ZNF184 | 2 | 1 | 0.675436988278554 | 1.95354594367574 |
| 4455 | HIST1H2BL | 19 | 1 | -5.78524754960902 | 1.71150173324178 |
| 4456 | HIST1H2AI | 19 | 1 | -5.78337214906581 | 1.65794195312093 |
| 4457 | HIST1H3H | 19 | 1 | -4.96085856874354 | 1.02272678989004 |
| 4458 | HIST1H2AJ | 19 | 1 | -5.7716123910702 | 1.63814391273092 |
| 4459 | HIST1H2BM | 19 | 1 | -5.76168464143641 | 1.63052047866415 |
| 4460 | HIST1H4J | 2 | 1 | 0.995283856793569 | 0.702862278400171 |
| 4461 | HIST1H2BN | 16 | 1 | -4.36527560670741 | 2.73521770614217 |
| 4462 | HIST1H2AK | 19 | 1 | -5.7275712343014 | 1.63640703338217 |
| 4463 | HIST1H2AL | 19 | 1 | -5.75809548814662 | 1.64029015677999 |
| 4464 | HIST1H1B | 19 | 1 | -5.73707316835292 | 1.66913212913107 |
| 4465 | HIST1H3I | 19 | 1 | -5.74129889924891 | 1.6349588789327 |
| 4466 | HIST1H4L | 19 | 1 | -5.6235730500973 | 1.68857897895406 |
| 4467 | HIST1H3J | 19 | 1 | -5.74514649827845 | 1.66652883666586 |
| 4468 | HIST1H2AM | 19 | 1 | -5.73382495363124 | 1.56434096473287 |
| 4469 | HIST1H2BO | 19 | 1 | -5.64350388963588 | 1.72231163161825 |
| 4470 | OR2B6 | 4 | 1 | -3.82527373750575 | -0.593102230848563 |
| 4471 | AL121944.1 | 4 | 1 | -4.29562161882289 | 0.0212372698171018 |
| 4472 | ZSCAN16-AS1 | 4 | 1 | -3.89590095003016 | -0.501784010948432 |
| 4473 | ZKSCAN8 | 2 | 1 | -0.560797438219859 | 2.13659586089681 |
| 4474 | ZSCAN9 | 5 | 1 | 3.07443000356786 | -0.178758545936835 |
| 4475 | ZKSCAN4 | 2 | 1 | 0.631860628529714 | 1.42390121596884 |
| 4476 | ZSCAN26 | 2 | 1 | 0.641473784848378 | 1.13776697295736 |
| 4477 | PGBD1 | 1 | 1 | 3.61695720235936 | 2.15105833190511 |
| 4478 | ZKSCAN3 | 9 | 1 | -0.158902257875278 | 2.64147223609518 |
| 4479 | ZSCAN12 | 9 | 1 | 0.731578603192494 | 2.61683262961935 |
| 4480 | ZSCAN23 | 8 | 1 | -1.53196369130976 | 1.69169868606161 |
| 4481 | ZBED9 | 2 | 1 | 0.929205193921254 | 1.90684236663412 |
| 4482 | AL049543.1 | 17 | 1 | 2.19509340803258 | 0.064586774287927 |
| 4483 | AL662890.1 | 4 | 1 | -3.55664251764186 | 0.0765391744954466 |
| 4484 | HCG14 | 4 | 1 | -3.91719387491114 | -0.433567806305182 |
| 4485 | TRIM27 | 8 | 1 | -1.31365214307673 | 1.10569919723104 |
| 4486 | GABBR1 | 5 | 1 | 4.47978617231481 | 0.571243063388574 |
| 4487 | HLA-A | 11 | 1 | -3.48501275499232 | -1.45733694654871 |
| 4488 | ZNRD1 | 11 | 1 | -3.1416439863003 | -1.44647614580561 |

| 4489 | PPP1R11 | 5 | 1 | 2.32738758604162 | -0.685796081246626 |
| --- | --- | --- | --- | --- | --- |
| 4490 | TRIM26 | 2 | 1 | -0.514880404070689 | 2.51692093986105 |
| 4491 | HCG18 | 9 | 1 | -0.18141777087815 | 2.50483144897054 |
| 4492 | TRIM39 | 2 | 1 | -0.265903387413098 | 0.83258070128988 |
| 4493 | HLA-E | 11 | 1 | -3.70457361657984 | -2.12945245129038 |
| 4494 | GNL1 | 1 | 1 | 3.78433729688756 | 1.37195529120992 |
| 4495 | ABCF1 | 3 | 1 | -2.31340168435939 | 0.341910616336572 |
| 4496 | PPP1R10 | 2 | 1 | -0.477949246958568 | 1.29846967834066 |
| 4497 | MRPS18B | 5 | 1 | 2.84448267500036 | -0.576520457090628 |
| 4498 | ATAT1 | 10 | 1 | 4.57483841459386 | -0.632142796339285 |
| 4499 | C6orf136 | 5 | 1 | 3.90841414014928 | -0.149683221401465 |
| 4500 | DHX16 | 8 | 1 | -1.58899663408168 | 1.30343784469198 |
| 4501 | PPP1R18 | 1 | 1 | 2.72256518880956 | 1.29364956992697 |
| 4502 | NRM | 13 | 1 | -0.99863158185847 | -2.19709943157603 |
| 4503 | MDC1 | 16 | 1 | -3.77149651964076 | 2.43576707023214 |
| 4504 | TUBB | 10 | 1 | 3.59858681242101 | -1.80771128755976 |
| 4505 | AL662797.1 | 1 | 1 | 2.62250687162511 | 1.53988041060995 |
| 4506 | FLOT1 | 6 | 1 | 2.36240269224279 | -1.35622398477961 |
| 4507 | IER3 | 14 | 1 | -5.00825856645472 | 0.228356734691369 |
| 4508 | DDR1 | 11 | 1 | -3.22722528894313 | -2.26059614044596 |
| 4509 | CCHCR1 | 19 | 1 | -4.12611912210353 | 1.41620196479391 |
| 4510 | TCF19 | 19 | 1 | -5.59522126634486 | 1.5840178884847 |
| 4511 | AL662844.4 | 16 | 1 | -3.43873094041712 | 2.79739774840902 |
| 4512 | HCG27 | 16 | 1 | -4.237444147662 | 3.06088151115011 |
| 4513 | HLA-C | 4 | 1 | -5.22625802476771 | -0.756789087476027 |
| 4514 | HLA-B | 14 | 1 | -6.33878110368617 | -0.459188326420081 |
| 4515 | MICA | 14 | 1 | -5.11405633409388 | 0.272963658748376 |
| 4516 | DDX39B | 2 | 1 | 0.461062028809712 | 1.69512333053182 |
| 4517 | ATP6V1G2 | 10 | 1 | 3.97948147337072 | -1.51366214376856 |
| 4518 | NFKBIL1 | 17 | 1 | -0.242750026945694 | -0.211968406261695 |
| 4519 | PRRC2A | 9 | 1 | -0.274878777757957 | 2.54846133369039 |
| 4520 | BAG6 | 17 | 1 | 2.19693209211461 | -0.266453310074103 |
| 4521 | APOM | 11 | 1 | -3.54659770448573 | -1.03645737391878 |
| 4522 | C6orf47 | 8 | 1 | -0.767917797163798 | -0.0633977494852663 |
| 4523 | GPANK1 | 16 | 1 | -4.25981019456752 | 2.27670015472006 |
| 4524 | CSNK2B | 13 | 1 | -1.14681576211817 | -2.06669007164408 |
| 4525 | LY6G5C | 5 | 1 | 4.52414442579381 | 0.436328903613794 |
| 4526 | DDAH2 | 6 | 1 | 0.341765954896138 | -2.31638823372294 |
| 4527 | CLIC1 | 13 | 1 | -1.6051392408169 | -2.62422309738566 |
| 4528 | VARS | 8 | 1 | -1.45662222822077 | 0.263743535457361 |
| 4529 | LSM2 | 13 | 1 | -2.55028842409022 | -1.62252847773005 |
| 4530 | HSPA1A | 17 | 1 | 1.55626787225835 | -0.587046845974219 |
| 4531 | HSPA1B | 17 | 1 | 1.48241306821935 | -0.565998657764685 |
| 4532 | C6orf48 | 2 | 1 | -0.708217397526576 | 1.88232196944784 |
| 4533 | EHMT2 | 2 | 1 | 0.681117847367452 | 2.17823781150411 |
| 4534 | ZBTB12 | 9 | 1 | 0.647530332013295 | 2.87733854430746 |
| 4535 | NELFE | 3 | 1 | -2.98958800752528 | -0.868117566378605 |
| 4536 | SKIV2L | 2 | 1 | -0.805205390051677 | 0.968995586810815 |
| 4537 | DXO | 13 | 1 | -0.403091818288638 | -0.620690568508399 |
| 4538 | ATF6B | 2 | 1 | 1.01685382406347 | 1.09384705203603 |
| 4539 | FKBPL | 16 | 1 | -4.3230359407223 | 2.22045315879415 |
| 4540 | EGFL8 | 9 | 1 | 0.265048578187154 | 2.76314009803365 |
| 4541 | AGPAT1 | 5 | 1 | 3.63352562467687 | 0.229206339297998 |
| 4542 | RNF5 | 5 | 1 | 2.54415966550939 | -0.503112479271186 |
| 4543 | PBX2 | 2 | 1 | -0.066495448545291 | 1.52616061347555 |
| 4544 | NOTCH4 | 18 | 1 | 5.32194687406652 | -0.761435522856009 |
| 4545 | AL662796.1 | 4 | 1 | -3.99919007738002 | -1.02981023413111 |
| 4546 | HLA-DRB1 | 18 | 1 | 6.09916807691686 | -2.14150915962626 |
| 4547 | HLA-DQB1 | 17 | 1 | 0.332766249581502 | -0.0144882641451479 |
| 4548 | BRD2 | 2 | 1 | 0.58274938146703 | 1.12726940291952 |
| 4549 | HLA-DPB1 | 11 | 1 | -3.06298349817164 | -1.99718926769663 |
| 4550 | COL11A2 | 18 | 1 | 5.2998979238712 | -0.693351372303259 |
| 4551 | RXRB | 2 | 1 | 0.142770632907079 | 0.732683197437036 |
| 4552 | SLC39A7 | 8 | 1 | -1.03769855220683 | -0.234395667137396 |
| 4553 | HSD17B8 | 11 | 1 | -3.82464812715419 | -2.26360557896067 |
| 4554 | RING1 | 5 | 1 | 3.65892340223424 | 0.0946064152104734 |
| 4555 | VPS52 | 2 | 1 | -0.859207853869273 | 2.24947251456808 |

| 4556 | RPS18 | 21 | 1 | -1.09355793912776 | -4.47090898376871 |
| --- | --- | --- | --- | --- | --- |
| 4557 | B3GALT4 | 6 | 1 | 2.22611023466222 | -1.13992766958643 |
| 4558 | WDR46 | 3 | 1 | -1.75585709531672 | -0.297200366081488 |
| 4559 | PFDN6 | 8 | 1 | -1.38046179731257 | 0.013979093013513 |
| 4560 | RGL2 | 5 | 1 | 3.15387631933324 | -0.858871380956662 |
| 4561 | TAPBP | 7 | 1 | -1.64574573953517 | -3.15176354271342 |
| 4562 | ZBTB22 | 17 | 1 | -0.206727147296741 | -0.371071442188513 |
| 4563 | DAXX | 16 | 1 | -3.98298738916285 | 1.91069950240682 |
| 4564 | KIFC1 | 16 | 1 | -4.64672110994227 | 3.07374371665548 |
| 4565 | PHF1 | 5 | 1 | 2.90061523000829 | -0.576716913999808 |
| 4566 | CUTA | 6 | 1 | 0.740564897462056 | -2.31091140133311 |
| 4567 | SYNGAP1 | 1 | 1 | 3.37643648664586 | 1.30761183875631 |
| 4568 | ZBTB9 | 1 | 1 | 2.44419123212926 | 0.537077442584741 |
| 4569 | BAK1 | 3 | 1 | -2.71976302583583 | -0.402314855875266 |
| 4570 | UQCC2 | 19 | 1 | -4.01884458978541 | 0.843628779826867 |
| 4571 | LEMD2 | 2 | 1 | -0.632041618422343 | 1.3325017847402 |
| 4572 | MLN | 14 | 1 | -6.93673966844447 | -0.738846554579032 |
| 4573 | HMGA1 | 11 | 1 | -3.14803670366175 | -1.22259680849482 |
| 4574 | SMIM29 | 10 | 1 | 4.24645067731969 | -1.39400543195177 |
| 4575 | NUDT3 | 5 | 1 | 4.4617631582462 | 0.657652632175195 |
| 4576 | RPS10 | 21 | 1 | -0.812282845572307 | -4.38046060425211 |
| 4577 | PACSIN1 | 10 | 1 | 4.03455425779454 | -1.39476652366091 |
| 4578 | C6orf106 | 2 | 1 | -0.965661511019542 | 1.74687065261434 |
| 4579 | AL451165.2 | 13 | 1 | -2.43495582063563 | -1.91455231529642 |
| 4580 | SNRPC | 3 | 1 | -2.44770692308314 | -0.625835075201285 |
| 4581 | UHRF1BP1 | 12 | 1 | 2.12683154623143 | 2.93169201987814 |
| 4582 | TAF11 | 6 | 1 | 0.0717548279010515 | -1.84255735498835 |
| 4583 | ANKS1A | 1 | 1 | 4.32015730421178 | 1.62142981666158 |
| 4584 | SCUBE3 | 1 | 1 | 3.04418469946019 | 1.99672140258383 |
| 4585 | ZNF76 | 8 | 1 | -1.35051296670802 | 0.608904615817773 |
| 4586 | PPARD | 3 | 1 | -1.97094045598872 | -0.00312738043237937 |
| 4587 | FANCE | 5 | 1 | 2.71967126409643 | -0.190644308151496 |
| 4588 | RPL10A | 21 | 1 | -1.13908831794627 | -4.33838306290079 |
| 4589 | TEAD3 | 7 | 1 | -3.65167282541163 | -2.81528232914377 |
| 4590 | FKBP5 | 19 | 1 | -5.05769418199427 | 1.44060839789938 |
| 4591 | ARMC12 | 14 | 1 | -5.70059894044764 | -0.527868553222907 |
| 4592 | LHFPL5 | 1 | 1 | 1.63570536653631 | 1.32669653075765 |
| 4593 | SRPK1 | 3 | 1 | -2.28602598626979 | 0.914725915370691 |
| 4594 | MAPK14 | 9 | 1 | 0.976519480153249 | 2.87658538001608 |
| 4595 | BRPF3 | 12 | 1 | 2.60403205434911 | 2.81692399161886 |
| 4596 | KCTD20 | 9 | 1 | 0.220326766177342 | 2.89622344153951 |
| 4597 | STK38 | 1 | 1 | 1.96997477094762 | 1.04826189654897 |
| 4598 | SRSF3 | 11 | 1 | -3.37008689363368 | -1.30604822856356 |
| 4599 | CDKN1A | 13 | 1 | -0.397226765946223 | -3.27868758064677 |
| 4600 | CPNE5 | 18 | 1 | 5.91938616315954 | -1.62354324680735 |
| 4601 | PPIL1 | 8 | 1 | -1.14803271015055 | 0.219826594768274 |
| 4602 | C6orf89 | 1 | 1 | 3.18449117223851 | 1.9497474588735 |
| 4603 | MTCH1 | 6 | 1 | 1.74336566011541 | -1.57956437689234 |
| 4604 | PIM1 | 5 | 1 | 3.49238635580175 | -0.770666866721404 |
| 4605 | TBC1D22B | 9 | 1 | 1.69668306390874 | 2.81885661262106 |
| 4606 | RNF8 | 2 | 1 | -0.317219399110152 | 1.74702180999349 |
| 4607 | CMTR1 | 9 | 1 | 0.54599447528951 | 2.9504569925649 |
| 4608 | CCDC167 | 13 | 1 | -0.24348147419162 | -2.146317347111 |
| 4609 | MDGA1 | 15 | 1 | 5.18089415113561 | 0.505850092349756 |
| 4610 | ZFAND3 | 1 | 1 | 1.3349242357456 | 1.51330317634176 |
| 4611 | BTBD9 | 1 | 1 | 3.25950695554845 | 1.01329447406362 |
| 4612 | GLO1 | 11 | 1 | -2.91509674508937 | -1.43151335340906 |
| 4613 | SAYSD1 | 6 | 1 | 1.40703536550634 | -1.04912839752604 |
| 4614 | MOCS1 | 2 | 1 | -0.0314853640354414 | 1.39277638572286 |
| 4615 | OARD1 | 3 | 1 | -1.87196646650202 | 0.0697214164121031 |
| 4616 | NFYA | 2 | 1 | -0.181874431864097 | 2.36888040679525 |
| 4617 | FOXP4-AS1 | 11 | 1 | -4.40689395387538 | -1.66375313621928 |
| 4618 | FOXP4 | 11 | 1 | -3.7499329896725 | -1.71883134466578 |
| 4619 | MDFI | 14 | 1 | -6.27526496370204 | -0.474405183614981 |
| 4620 | TFEB | 7 | 1 | -2.44847987611659 | -3.47340975624491 |
| 4621 | FRS3 | 10 | 1 | 3.43500114957921 | -1.60471777540613 |
| 4622 | USP49 | 9 | 1 | 1.16152621786229 | 3.06925453322958 |

| 4623 | MED20 | 3 | 1 | -2.54176853616603 | -0.485712959589255 |
| --- | --- | --- | --- | --- | --- |
| 4624 | BYSL | 3 | 1 | -2.87513588388331 | -1.12714545351435 |
| 4625 | CCND3 | 10 | 1 | 4.41053272764318 | -1.06313303035666 |
| 4626 | TAF8 | 4 | 1 | -4.23940037210353 | 0.0144268431050657 |
| 4627 | AL512274.1 | 14 | 1 | -5.18198798616297 | 0.12084175723623 |
| 4628 | C6orf132 | 11 | 1 | -3.4907605500973 | -2.32923506122996 |
| 4629 | GUCA1A | 13 | 1 | -1.76706336458094 | -2.58827017170359 |
| 4630 | MRPS10 | 3 | 1 | -2.44345186670191 | 1.04146434444021 |
| 4631 | TRERF1 | 1 | 1 | 3.01413108389013 | 1.35931195396017 |
| 4632 | UBR2 | 9 | 1 | 0.497440472050832 | 2.7840866007192 |
| 4633 | TBCC | 5 | 1 | 2.58160330335729 | -0.422594114364874 |
| 4634 | BICRAL | 1 | 1 | 4.21311356107824 | 1.57328166144918 |
| 4635 | RPL7L1 | 3 | 1 | -2.07342110593684 | 0.0927307762486814 |
| 4636 | C6orf226 | 5 | 1 | 2.70452715437047 | -1.26163060885836 |
| 4637 | CNPY3 | 3 | 1 | -2.9786321969784 | -0.874145410181773 |
| 4638 | PPP2R5D | 16 | 1 | -3.54079578836329 | 2.87444795745443 |
| 4639 | MEA1 | 6 | 1 | -0.234195117101743 | -1.29952506643702 |
| 4640 | KLHDC3 | 17 | 1 | 1.72517074625127 | -0.60027800423075 |
| 4641 | RRP36 | 3 | 1 | -1.90722344835169 | -1.02814340514113 |
| 4642 | CUL7 | 8 | 1 | -1.74561236818202 | 0.744447843013513 |
| 4643 | MRPL2 | 3 | 1 | -1.6829928012646 | -0.968285202206862 |
| 4644 | AL355385.1 | 11 | 1 | -3.76915930230982 | -1.98771141392161 |
| 4645 | PTK7 | 11 | 1 | -3.36801265199549 | -1.6473708592074 |
| 4646 | SRF | 8 | 1 | -1.62072025258906 | 0.889810816226709 |
| 4647 | CUL9 | 1 | 1 | 4.09502245466344 | 2.2397066511495 |
| 4648 | DNPH1 | 4 | 1 | -4.37079928834803 | -0.55097545606066 |
| 4649 | TTBK1 | 1 | 1 | 3.30944944898717 | 2.06637419837545 |
| 4650 | CRIP3 | 16 | 1 | -3.64125536401637 | 2.25039447921346 |
| 4651 | ZNF318 | 12 | 1 | 2.86665369550817 | 2.63700188773702 |
| 4652 | ABCC10 | 1 | 1 | 3.23080827276342 | 1.81638063567709 |
| 4653 | DLK2 | 15 | 1 | 4.94899775068395 | -0.212389453472388 |
| 4654 | TJAP1 | 11 | 1 | -3.82502602060206 | -1.59109614712168 |
| 4655 | LRRC73 | 5 | 1 | 3.62847186605565 | -0.374762579025519 |
| 4656 | POLR1C | 8 | 1 | -0.764226302698924 | -0.128332003177893 |
| 4657 | YIPF3 | 13 | 1 | -1.09282515962489 | -1.24019385796953 |
| 4658 | XPO5 | 2 | 1 | -0.604368701771571 | 2.52321733611654 |
| 4659 | POLH | 9 | 1 | -0.174505062595679 | 2.81027020591329 |
| 4660 | POLH-AS1 | 1 | 1 | 2.52012301962011 | 1.17107285636495 |
| 4661 | MAD2L1BP | 17 | 1 | 0.272696092530415 | -0.12565801960398 |
| 4662 | RSPH9 | 10 | 1 | 2.708929315015 | -1.93822608810831 |
| 4663 | MRPS18A | 3 | 1 | -2.26051281412013 | -0.951783302398217 |
| 4664 | VEGFA | 4 | 1 | -4.39592717607386 | -1.32151605111529 |
| 4665 | MRPL14 | 3 | 1 | -2.40286825616725 | -1.45665971857477 |
| 4666 | TMEM63B | 5 | 1 | 3.92011763136022 | -0.883682501660478 |
| 4667 | SLC29A1 | 17 | 1 | 1.93551327268712 | -0.0582933269159914 |
| 4668 | HSP90AB1 | 11 | 1 | -3.52307771165736 | -1.80048774105479 |
| 4669 | SLC35B2 | 3 | 1 | -1.88409183938868 | -1.19162537080218 |
| 4670 | NFKBIE | 2 | 1 | 0.524612024232076 | 0.735702411113489 |
| 4671 | TMEM151B | 15 | 1 | 4.78974510709874 | -0.189424856724036 |
| 4672 | AARS2 | 8 | 1 | -1.5737067313946 | 1.90001548904012 |
| 4673 | CDC5L | 8 | 1 | -1.20558945854075 | 0.528781906543481 |
| 4674 | SUPT3H | 11 | 1 | -3.33197020013697 | -1.75847349983622 |
| 4675 | AL096865.1 | 14 | 1 | -6.69329427202113 | -0.303527637542975 |
| 4676 | ENPP4 | 1 | 1 | 3.67077924291723 | 0.538372532306421 |
| 4677 | RCAN2 | 10 | 1 | 4.33193756620519 | -1.0238546721356 |
| 4678 | CYP39A1 | 4 | 1 | -4.27968667467005 | -0.707509010257018 |
| 4679 | SLC25A27 | 15 | 1 | 4.34003330747716 | -0.0477438412325503 |
| 4680 | TNFRSF21 | 15 | 1 | 5.27682615797155 | 0.251045600352991 |
| 4681 | CD2AP | 8 | 1 | -1.85873590429194 | 1.77006258147787 |
| 4682 | MUT | 12 | 1 | 1.77920962850683 | 1.78974928039144 |
| 4683 | CENPQ | 19 | 1 | -4.90003655870326 | 1.97602357047628 |
| 4684 | C6orf141 | 14 | 1 | -4.86051057298548 | 0.4807635702474 |
| 4685 | TFAP2B | 20 | 1 | 3.31221629659765 | -3.79019103866984 |
| 4686 | MCM3 | 19 | 1 | -5.114321932391 | 0.864759222446191 |
| 4687 | PAQR8 | 5 | 1 | 2.93954564611547 | -0.600734426798117 |
| 4688 | EFHC1 | 5 | 1 | 3.40727473775976 | 0.218038217006433 |
| 4689 | TRAM2 | 4 | 1 | -4.94492219407923 | -0.309110804619086 |

| 4690 | TRAM2-AS1 | 4 | 1 | -3.61308239419825 | -0.120480462135565 |
| --- | --- | --- | --- | --- | --- |
| 4691 | TMEM14A | 10 | 1 | 3.19864250700109 | -1.73953519684244 |
| 4692 | GSTA1 | 15 | 1 | 5.74925543348424 | 0.609851256786096 |
| 4693 | GSTA4 | 10 | 1 | 3.84323765318029 | -1.24329577309061 |
| 4694 | ICK | 5 | 1 | 3.08515788595311 | -0.181207819999945 |
| 4695 | FBXO9 | 1 | 1 | 3.34118653814428 | 1.00252045768331 |
| 4696 | ELOVL5 | 2 | 1 | -1.12623505313761 | 2.02008999961447 |
| 4697 | GCLC | 9 | 1 | 0.864723220273183 | 2.43105496543478 |
| 4698 | LRRC1 | 14 | 1 | -5.27210591752894 | 0.448207394061792 |
| 4699 | HCRTR2 | 18 | 1 | 5.68736578504674 | -0.492907597841513 |
| 4700 | HMGCLL1 | 10 | 1 | 3.97798611204259 | -1.39193500501086 |
| 4701 | BMP5 | 14 | 1 | -6.19649671037562 | 0.0713957704884886 |
| 4702 | DST | 1 | 1 | 3.38672257940404 | 1.19711507934164 |
| 4703 | BEND6 | 10 | 1 | 4.43114496748082 | -2.05660663467814 |
| 4704 | KIAA1586 | 9 | 1 | 0.874211326047109 | 2.45629443305563 |
| 4705 | ZNF451 | 12 | 1 | 2.58804585020177 | 2.77730192321371 |
| 4706 | BAG2 | 17 | 1 | 1.93123246709935 | 0.131730452953088 |
| 4707 | PRIM2 | 16 | 1 | -4.25126956422694 | 2.29913820403646 |
| 4708 | AL021368.2 | 12 | 1 | 2.57096411268346 | 2.55822075980734 |
| 4709 | KHDRBS2 | 1 | 1 | 2.95554734747045 | 1.82290472167562 |
| 4710 | FKBP1C | 7 | 1 | -3.95357297380336 | -2.90085028034617 |
| 4711 | PTP4A1 | 9 | 1 | -0.095459014371707 | 2.65447844642233 |
| 4712 | PHF3 | 9 | 1 | 1.80241371671789 | 3.06647004264425 |
| 4713 | AL391807.1 | 10 | 1 | 4.58596207182042 | -1.84412155968119 |
| 4714 | ADGRB3 | 15 | 1 | 5.21493889371984 | 0.616740123210657 |
| 4715 | LMBRD1 | 5 | 1 | 4.49342609922521 | 0.188340679584253 |
| 4716 | COL9A1 | 19 | 1 | -4.46108125169642 | 1.44293703216146 |
| 4717 | FAM135A | 12 | 1 | 3.2501731065952 | 2.88792933600973 |
| 4718 | SDHAF4 | 5 | 1 | 3.90500475446813 | -0.132582410396826 |
| 4719 | SMAP1 | 10 | 1 | 4.38466097395055 | -1.09886942726542 |
| 4720 | B3GAT2 | 5 | 1 | 4.14102484266393 | 0.156471148906457 |
| 4721 | OGFRL1 | 15 | 1 | 5.49581553022496 | 0.854700700221765 |
| 4722 | AL136164.1 | 14 | 1 | -5.36998341997035 | -0.410961344064963 |
| 4723 | LINC00472 | 4 | 1 | -5.08864139039881 | -0.742984770955336 |
| 4724 | RIMS1 | 1 | 1 | 4.66532446424596 | 0.658222333369958 |
| 4725 | AC019205.1 | 5 | 1 | 4.05600191633336 | -0.242140098633063 |
| 4726 | AL603910.1 | 18 | 1 | 5.48877455274694 | -0.985518357874644 |
| 4727 | EEF1A1 | 3 | 1 | -1.69354496915705 | -1.22754143101145 |
| 4728 | SLC17A5 | 5 | 1 | 3.51275922338598 | -0.66072839421202 |
| 4729 | AL590428.1 | 14 | 1 | -5.63727496583827 | -0.729230269016516 |
| 4730 | CD109 | 4 | 1 | -5.07790635545619 | -0.71233805936743 |
| 4731 | COL12A1 | 14 | 1 | -6.15822480638392 | 0.148573652683008 |
| 4732 | COX7A2 | 6 | 1 | 0.609516516133473 | -2.35513387543131 |
| 4733 | TMEM30A | 1 | 1 | 3.23603798429601 | 1.06753231662344 |
| 4734 | FILIP1 | 16 | 1 | -4.22382973153956 | 2.52528037208151 |
| 4735 | SENP6 | 9 | 1 | 0.890887394353078 | 2.97055329459738 |
| 4736 | MYO6 | 5 | 1 | 3.05791737119787 | 0.0245695270879148 |
| 4737 | IRAK1BP1 | 1 | 1 | 1.66919745485418 | 1.62415541785787 |
| 4738 | PHIP | 2 | 1 | -0.672182843283488 | 1.91971935409139 |
| 4739 | HMGN3 | 11 | 1 | -3.39000962694056 | -1.41112469059397 |
| 4740 | HMGN3-AS1 | 11 | 1 | -3.48046658952601 | -2.17833815437723 |
| 4741 | LCA5 | 7 | 1 | -1.88599918802149 | -3.32751880508829 |
| 4742 | AL451064.1 | 6 | 1 | 2.14017011205785 | -2.15761326176096 |
| 4743 | SH3BGRL2 | 1 | 1 | 1.5971802620136 | 1.45964898246359 |
| 4744 | ELOVL4 | 5 | 1 | 3.34513308088414 | -0.287765666069281 |
| 4745 | TTK | 16 | 1 | -4.44739078004725 | 3.30099095481466 |
| 4746 | BCKDHB | 19 | 1 | -3.89610718210108 | 0.540849701343286 |
| 4747 | AL359715.1 | 4 | 1 | -4.22232244928248 | 0.348433629451501 |
| 4748 | TENT5A | 14 | 1 | -6.79306529481776 | -0.576287909569037 |
| 4749 | IBTK | 12 | 1 | 1.70462705175512 | 2.55887045043539 |
| 4750 | TPBG | 4 | 1 | -4.33179353196986 | -1.16987569552828 |
| 4751 | UBE3D | 2 | 1 | 0.527870073720143 | 2.27966727393697 |
| 4752 | DOPEY1 | 1 | 1 | 3.55363871137731 | 2.40698303359579 |
| 4753 | PGM3 | 3 | 1 | -3.44722770173915 | 0.384374753413904 |
| 4754 | RWDD2A | 2 | 1 | 0.16305904070966 | 0.588740841327417 |
| 4755 | PRSS35 | 4 | 1 | -4.91288779695399 | -0.246163590969336 |
| 4756 | SNAP91 | 5 | 1 | 4.05609203855626 | -0.0410957775728823 |

| 4757 | RIPPLY2 | 15 | 1 | 5.14995552579992 | -0.0283058725016238 |
| --- | --- | --- | --- | --- | --- |
| 4758 | CYB5R4 | 7 | 1 | -2.67923568208583 | -2.25607441288401 |
| 4759 | MRAP2 | 5 | 1 | 4.19071365873449 | -0.141198917450202 |
| 4760 | CEP162 | 9 | 1 | 0.730754449769185 | 2.68809498923849 |
| 4761 | SNX14 | 5 | 1 | 4.10418058912389 | 0.60100545066427 |
| 4762 | SYNCRIP | 8 | 1 | -1.87908159215815 | 1.20226515906881 |
| 4763 | HTR1E | 7 | 1 | -3.29349921663172 | -3.75816331726481 |
| 4764 | AL139274.2 | 5 | 1 | 3.38532163183324 | -0.481610342087042 |
| 4765 | ZNF292 | 9 | 1 | 1.65484501402013 | 3.06711687224935 |
| 4766 | GJB7 | 7 | 1 | -2.6225166173733 | -2.64859603268076 |
| 4767 | SMIM8 | 1 | 1 | 3.2045405058109 | 1.66119612830709 |
| 4768 | CFAP206 | 6 | 1 | 1.8555255083286 | -1.08893455487658 |
| 4769 | AL049697.1 | 12 | 1 | 3.49158646146886 | 2.73572577613424 |
| 4770 | SLC35A1 | 5 | 1 | 4.03830887357824 | 0.251325503764856 |
| 4771 | RARS2 | 8 | 1 | -1.63996301610835 | -0.0994092665331484 |
| 4772 | ORC3 | 3 | 1 | -2.66716788728602 | 0.871762410579431 |
| 4773 | AKIRIN2 | 5 | 1 | 2.74389196912877 | 0.18189562934469 |
| 4774 | CNR1 | 15 | 1 | 4.8250787405216 | 0.243569508968103 |
| 4775 | RNGTT | 1 | 1 | 3.55666519682042 | 2.65927209037374 |
| 4776 | PNRC1 | 7 | 1 | -2.83821652849086 | -2.31278632503916 |
| 4777 | SRSF12 | 1 | 1 | 4.30488277952306 | 0.672481433330285 |
| 4778 | PM20D2 | 9 | 1 | -0.101439267591311 | 3.07230819839071 |
| 4779 | UBE2J1 | 9 | 1 | 1.97482205907934 | 2.82098020690511 |
| 4780 | RRAGD | 18 | 1 | 5.28605772535436 | -0.530132099212897 |
| 4781 | ANKRD6 | 1 | 1 | 3.72724939863317 | 2.43719710486959 |
| 4782 | LYRM2 | 2 | 1 | -0.295670245037152 | 1.26551784652303 |
| 4783 | MDN1 | 2 | 1 | -0.896409616068675 | 1.93837250846456 |
| 4784 | CASP8AP2 | 9 | 1 | -0.135539047614409 | 3.08132471221517 |
| 4785 | BACH2 | 18 | 1 | 5.18566204588048 | -0.476628079237235 |
| 4786 | MAP3K7 | 2 | 1 | -0.701176122025325 | 1.95019306319784 |
| 4787 | CASC6 | 6 | 1 | 2.44517470876806 | -1.48816917759348 |
| 4788 | EPHA7 | 4 | 1 | -4.48286078889735 | -1.04479621273447 |
| 4789 | MANEA-DT | 1 | 1 | 3.06671215574376 | 0.831217543063867 |
| 4790 | MANEA | 19 | 1 | -4.13289330919154 | 1.42700852531027 |
| 4791 | FUT9 | 1 | 1 | 4.86840416471593 | 1.12884558814596 |
| 4792 | UFL1 | 3 | 1 | -3.2263254972256 | -0.828186883749259 |
| 4793 | GPR63 | 4 | 1 | -3.70245145280726 | -0.103024943889868 |
| 4794 | NDUFAF4 | 13 | 1 | -1.29317949731715 | -0.974407768966448 |
| 4795 | KLHL32 | 15 | 1 | 4.49990345518224 | -0.208993776859534 |
| 4796 | MMS22L | 19 | 1 | -4.87243913133509 | 1.71842159408163 |
| 4797 | AL589740.1 | 18 | 1 | 5.15178896467321 | -1.79261092764307 |
| 4798 | AL589826.2 | 14 | 1 | -6.62348149736293 | -1.00645735782553 |
| 4799 | POU3F2 | 18 | 1 | 5.46407534162633 | -2.06417129856516 |
| 4800 | FAXC | 15 | 1 | 4.93954922239415 | -0.202690406860602 |
| 4801 | COQ3 | 6 | 1 | 2.24955417196386 | -0.977893545450461 |
| 4802 | PNISR | 2 | 1 | 0.135985925599263 | 1.68648089545797 |
| 4803 | USP45 | 7 | 1 | -3.16558717210658 | -2.79074583870341 |
| 4804 | TSTD3 | 11 | 1 | -3.0870888086117 | -1.17586745244433 |
| 4805 | CCNC | 8 | 1 | -1.93546281774409 | 1.25874294417928 |
| 4806 | PRDM13 | 7 | 1 | -3.08515714128382 | -3.95072255951334 |
| 4807 | SIM1 | 7 | 1 | -2.89890526254542 | -3.94263993126322 |
| 4808 | ASCC3 | 8 | 1 | -1.41410790403254 | 1.20074810164999 |
| 4809 | GRIK2 | 15 | 1 | 5.07790161649816 | 0.412242666660058 |
| 4810 | HACE1 | 8 | 1 | -1.44054768998988 | 1.60422934669088 |
| 4811 | LIN28B | 12 | 1 | 2.28466154615514 | 1.56677688735555 |
| 4812 | BVES | 6 | 1 | 2.0433757452213 | -2.20772693973948 |
| 4813 | PREP | 1 | 1 | 1.41924442331426 | 1.38298763412069 |
| 4814 | ATG5 | 8 | 1 | -0.564581051663234 | -0.250866635860694 |
| 4815 | PRDM1 | 11 | 1 | -4.17212651689418 | -2.06372891289164 |
| 4816 | RTN4IP1 | 8 | 1 | -0.40658020992644 | -0.195656045498145 |
| 4817 | QRSL1 | 2 | 1 | -1.02913276870616 | 1.51898969787191 |
| 4818 | CD24 | 10 | 1 | 4.12662054578893 | -1.14291882437636 |
| 4819 | C6orf203 | 6 | 1 | 1.94748760740392 | -2.38111375194956 |
| 4820 | BEND3 | 8 | 1 | -2.04537723977931 | 1.30035437720846 |
| 4821 | SOBP | 15 | 1 | 4.60774208585851 | 0.341560021816003 |
| 4822 | SCML4 | 18 | 1 | 5.52357508222692 | -1.52450166565348 |
| 4823 | SEC63 | 2 | 1 | -0.169067524567916 | 1.73716725486349 |

| 4824 | OSTM1 | 1 | 1 | 4.32015301267736 | 1.8144091524465 |
| --- | --- | --- | --- | --- | --- |
| 4825 | OSTM1-AS1 | 4 | 1 | -4.73063300569422 | -0.828959620715869 |
| 4826 | NR2E1 | 4 | 1 | -5.13778041322596 | -1.59040199142863 |
| 4827 | SNX3 | 13 | 1 | -0.719076112107112 | -1.91298364025522 |
| 4828 | FOXO3 | 1 | 1 | 3.62935735265844 | 1.66589225905966 |
| 4829 | ARMC2 | 6 | 1 | 1.4076495317661 | -1.64573411566187 |
| 4830 | SESN1 | 9 | 1 | 1.56639863531225 | 2.91985000747274 |
| 4831 | CEP57L1 | 16 | 1 | -3.95042918641932 | 2.84573592322897 |
| 4832 | CD164 | 2 | 1 | -0.602759525374248 | 1.43487943786215 |
| 4833 | AL359711.2 | 4 | 1 | -4.00888608415492 | -0.393022521557105 |
| 4834 | PPIL6 | 13 | 1 | -0.861156091288402 | -2.7282048307078 |
| 4835 | MICAL1 | 10 | 1 | 3.19435907880895 | -1.39313815456797 |
| 4836 | ZBTB24 | 9 | 1 | -0.237497833751812 | 2.87397088187765 |
| 4837 | AK9 | 5 | 1 | 3.600854888364 | 0.128881827769983 |
| 4838 | FIG4 | 5 | 1 | 3.3984007982456 | 0.195053712306726 |
| 4839 | GPR6 | 6 | 1 | 2.08160974065893 | -2.39709900242258 |
| 4840 | WASF1 | 1 | 1 | 3.44621564428441 | 1.1612268843038 |
| 4841 | CDC40 | 2 | 1 | -0.590226277903392 | 0.78742279189657 |
| 4842 | CDK19 | 9 | 1 | -0.16045712699063 | 3.14439429420065 |
| 4843 | AMD1 | 2 | 1 | 1.10182477514379 | 0.943730012355554 |
| 4844 | GTF3C6 | 13 | 1 | -2.16193400342829 | -1.58967511278559 |
| 4845 | RPF2 | 3 | 1 | -2.59276030977137 | -0.361734911026251 |
| 4846 | SLC16A10 | 14 | 1 | -5.33617185075648 | 0.440020457683313 |
| 4847 | MFSD4B | 9 | 1 | 1.47970618288152 | 2.70010556357931 |
| 4848 | AL080317.2 | 9 | 1 | 1.54708065073125 | 3.09823550361227 |
| 4849 | REV3L | 12 | 1 | 3.38726665060155 | 2.78666056769918 |
| 4850 | TRAF3IP2-AS1 | 1 | 1 | 2.93617202322118 | 1.6175712503774 |
| 4851 | TRAF3IP2 | 1 | 1 | 4.00486208479039 | 1.03108336108755 |
| 4852 | Z97989.1 | 1 | 1 | 4.59711195509069 | 1.19375480788778 |
| 4853 | FYN | 1 | 1 | 1.52200020830266 | 1.54494489806722 |
| 4854 | TUBE1 | 15 | 1 | 5.63274742643468 | 0.691019193111169 |
| 4855 | FAM229B | 10 | 1 | 4.10100294630162 | -1.26360000592638 |
| 4856 | LAMA4 | 15 | 1 | 5.33468557874791 | 0.0577580489499449 |
| 4857 | Z99289.1 | 15 | 1 | 5.94901873151891 | 0.792142645297754 |
| 4858 | MARCKS | 12 | 1 | 2.50095225851171 | 2.3518425382955 |
| 4859 | LINC01268 | 1 | 1 | 2.1165015844547 | 1.22838653701376 |
| 4860 | HDAC2 | 10 | 1 | 3.83000017683141 | -1.08321010989119 |
| 4861 | HDAC2-AS2 | 3 | 1 | -2.87225388009913 | 0.433606997905481 |
| 4862 | HS3ST5 | 4 | 1 | -5.25135062654383 | -1.67696998936106 |
| 4863 | NT5DC1 | 4 | 1 | -4.43382571657069 | -0.866994629772198 |
| 4864 | TSPYL4 | 5 | 1 | 3.54647399465673 | -0.49754060727526 |
| 4865 | DSE | 9 | 1 | 0.601323142453359 | 2.7539569296224 |
| 4866 | TSPYL1 | 1 | 1 | 3.34094358961217 | 1.62529887336324 |
| 4867 | CALHM5 | 14 | 1 | -6.83235190828212 | -0.717779218377364 |
| 4868 | RWDD1 | 13 | 1 | -0.751475677088573 | -2.31716404777933 |
| 4869 | RSPH4A | 6 | 1 | 1.73719216863744 | -1.08518150371481 |
| 4870 | ZUP1 | 2 | 1 | 0.809035315915273 | 1.05058588164877 |
| 4871 | KPNA5 | 1 | 1 | 4.30255056898229 | 1.27350558417867 |
| 4872 | FAM162B | 20 | 1 | 3.29527808706395 | -3.86606989723612 |
| 4873 | DCBLD1 | 4 | 1 | -4.3104102464474 | -0.81172575724293 |
| 4874 | GOPC | 10 | 1 | 3.99561239759557 | -1.39438284856249 |
| 4875 | NUS1 | 2 | 1 | -0.156348809794261 | 2.33105816024374 |
| 4876 | SLC35F1 | 4 | 1 | -5.21917174775965 | -0.800808846177352 |
| 4877 | CEP85L | 2 | 1 | 0.301967516347096 | 2.36052002090048 |
| 4878 | PLN | 4 | 1 | -5.37833093126185 | -1.80038271767069 |
| 4879 | MCM9 | 8 | 1 | -2.78211830575831 | 1.49871863502096 |
| 4880 | ASF1A | 3 | 1 | -2.38397358377345 | -0.940293087782157 |
| 4881 | AL365275.1 | 4 | 1 | -5.13526318986781 | -0.379730983795416 |
| 4882 | FAM184A | 1 | 1 | 4.31062294523351 | 0.751664534984338 |
| 4883 | MAN1A1 | 7 | 1 | -2.34127424676783 | -3.49097905975748 |
| 4884 | TBC1D32 | 9 | 1 | 1.31192769090764 | 2.29829539435934 |
| 4885 | GJA1 | 7 | 1 | -3.72026537378199 | -3.36191473823954 |
| 4886 | HSF2 | 12 | 1 | 2.43938709776037 | 2.12709917205404 |
| 4887 | SERINC1 | 1 | 1 | 3.90405727903478 | 1.3074773706777 |
| 4888 | PKIB | 6 | 1 | 2.26998139898412 | -2.22667203766276 |
| 4889 | FABP7 | 5 | 1 | 4.05979992429845 | -0.69098211747576 |
| 4890 | SMPDL3A | 5 | 1 | 4.20285154859655 | -0.827649026335967 |

| 4891 | CLVS2 | 1 | 1 | 4.65591026823156 | 1.24856032508444 |
| --- | --- | --- | --- | --- | --- |
| 4892 | NKAIN2 | 18 | 1 | 5.68161369840734 | -1.81019376141001 |
| 4893 | RNF217 | 1 | 1 | 3.23224164526098 | 2.01070441382955 |
| 4894 | TPD52L1 | 18 | 1 | 6.29181553403966 | -2.45209799629618 |
| 4895 | HDDC2 | 11 | 1 | -2.65279243905909 | -1.64451669079233 |
| 4896 | HEY2 | 7 | 1 | -3.04599021394618 | -3.51407991272379 |
| 4897 | NCOA7 | 10 | 1 | 3.78192450086706 | -1.29452927333285 |
| 4898 | HINT3 | 5 | 1 | 3.82289387266271 | -0.190462037147772 |
| 4899 | TRMT11 | 3 | 1 | -2.93855617959864 | 0.429100648341882 |
| 4900 | CENPW | 16 | 1 | -4.40094731767543 | 2.57979764121603 |
| 4901 | RSPO3 | 6 | 1 | 1.31106843034856 | -2.84251819473673 |
| 4902 | RNF146 | 6 | 1 | 2.14451004545324 | -1.36857421618868 |
| 4903 | ECHDC1 | 8 | 1 | -1.84626589734919 | 0.489596859393823 |
| 4904 | AL096711.2 | 1 | 1 | 3.77581001798742 | 0.857879773555505 |
| 4905 | KIAA0408 | 1 | 1 | 3.28466226141088 | 2.44728840964864 |
| 4906 | SOGA3 | 9 | 1 | 1.39347769300573 | 2.31929506438802 |
| 4907 | PTPRK | 7 | 1 | -2.24297307451136 | -2.74666295868326 |
| 4908 | ARHGAP18 | 6 | 1 | 1.45107568781011 | -2.3054798684733 |
| 4909 | L3MBTL3 | 5 | 1 | 3.90533854047887 | 0.0611521162373899 |
| 4910 | AL355581.1 | 15 | 1 | 5.61944080869787 | 0.237131253658044 |
| 4911 | SAMD3 | 5 | 1 | 4.45052696745031 | -0.395047589363349 |
| 4912 | TMEM200A | 15 | 1 | 4.6984369901859 | -0.158702894272101 |
| 4913 | EPB41L2 | 14 | 1 | -5.35672830064662 | 0.295391217647302 |
| 4914 | AKAP7 | 14 | 1 | -6.07548545320399 | -0.477124258102667 |
| 4915 | MED23 | 12 | 1 | 2.7014055399143 | 2.43179096358846 |
| 4916 | ENPP1 | 14 | 1 | -6.48736022432215 | -0.264046057285559 |
| 4917 | CTGF | 14 | 1 | -6.55086635072596 | -0.244015379966986 |
| 4918 | AL133346.1 | 14 | 1 | -5.61255000551112 | -0.121457918705237 |
| 4919 | MOXD1 | 7 | 1 | -3.50829122980006 | -3.6083534799235 |
| 4920 | STX7 | 5 | 1 | 3.85487820188634 | -0.426042958320868 |
| 4921 | SLC18B1 | 4 | 1 | -5.00023292024501 | -1.64068995338846 |
| 4922 | RPS12 | 21 | 1 | -0.93469582517512 | -4.42811427933146 |
| 4923 | EYA4 | 4 | 1 | -4.34319280107386 | -0.939280319185746 |
| 4924 | AL450270.1 | 4 | 1 | -4.60548900087245 | -0.645755364717734 |
| 4925 | TBPL1 | 7 | 1 | -2.03159569223292 | -2.88295315128733 |
| 4926 | SLC2A12 | 4 | 1 | -4.24898289163478 | 0.0495501317365049 |
| 4927 | SGK1 | 15 | 1 | 6.01190306226842 | 0.758302585063684 |
| 4928 | HBS1L | 9 | 1 | 0.281922951146291 | 2.96207465308737 |
| 4929 | MYB | 19 | 1 | -5.71100924928553 | 1.73893036025594 |
| 4930 | AHI1 | 5 | 1 | 3.94430805723302 | 0.382990614352929 |
| 4931 | MTFR2 | 16 | 1 | -4.66075013597377 | 3.06942810195516 |
| 4932 | BCLAF1 | 2 | 1 | -0.153414912716224 | 2.44384302276205 |
| 4933 | MAP7 | 5 | 1 | 4.63172365705602 | 0.63846768516134 |
| 4934 | PEX7 | 4 | 1 | -4.62466548402674 | -0.585073336185706 |
| 4935 | IFNGR1 | 8 | 1 | -1.46455155332453 | 0.164610878406274 |
| 4936 | OLIG3 | 20 | 1 | 3.05093266050451 | -3.88579045158793 |
| 4937 | PERP | 6 | 1 | 1.79640318433873 | -2.71396206242014 |
| 4938 | ARFGEF3 | 5 | 1 | 3.34563995878331 | -0.197672291816962 |
| 4939 | HEBP2 | 6 | 1 | 0.763551130696462 | -2.7439416490214 |
| 4940 | NHSL1 | 14 | 1 | -6.61164639909632 | -0.776422321023238 |
| 4941 | AL590617.2 | 11 | 1 | -3.40381191690333 | -1.14778621417452 |
| 4942 | CCDC28A | 3 | 1 | -2.80884145219691 | -0.770454942406905 |
| 4943 | REPS1 | 2 | 1 | 0.566622450753377 | 1.50487960952352 |
| 4944 | ABRACL | 6 | 1 | 0.532454862996266 | -2.28115604740549 |
| 4945 | HECA | 1 | 1 | 4.07357479612462 | 2.00747741836141 |
| 4946 | CITED2 | 10 | 1 | 4.34583450834386 | -1.67929528576304 |
| 4947 | VTA1 | 9 | 1 | -0.0963899793184538 | 2.58868230956625 |
| 4948 | ADGRG6 | 14 | 1 | -6.39598582704432 | -1.28688774925638 |
| 4949 | HIVEP2 | 2 | 1 | 0.319275095864461 | 2.11117185729574 |
| 4950 | LINC01277 | 9 | 1 | 1.14725030939214 | 2.3185948290212 |
| 4951 | AIG1 | 7 | 1 | -2.70312951524623 | -3.2777231774943 |
| 4952 | ADAT2 | 3 | 1 | -2.96319530923732 | 0.594531909404504 |
| 4953 | FUCA2 | 11 | 1 | -2.99855493028529 | -1.66456608396937 |
| 4954 | PHACTR2 | 4 | 1 | -4.29850886781581 | -1.06851977032591 |
| 4955 | LTV1 | 3 | 1 | -2.23627160508998 | 0.174222961841333 |
| 4956 | PLAGL1 | 4 | 1 | -4.44338630159266 | -0.284671171726477 |
| 4957 | SF3B5 | 11 | 1 | -2.7116484494961 | -1.63714300018717 |

| 4958 | UTRN | 7 | 1 | -3.62830017526515 | -2.79518781525065 |
| --- | --- | --- | --- | --- | --- |
| 4959 | EPM2A | 8 | 1 | -1.55340014417536 | 1.45152582305502 |
| 4960 | AL023806.1 | 2 | 1 | 0.814826264783071 | 1.39941157477926 |
| 4961 | FBXO30 | 9 | 1 | 0.807062282963918 | 2.83817352431844 |
| 4962 | AL356599.1 | 12 | 1 | 1.90147687475316 | 1.85930837768148 |
| 4963 | SHPRH | 2 | 1 | -0.513097092703654 | 1.92502130645345 |
| 4964 | GRM1 | 15 | 1 | 5.63372160474889 | 0.665417925296533 |
| 4965 | RAB32 | 3 | 1 | -3.33324454744227 | -1.06660258692671 |
| 4966 | STXBP5-AS1 | 15 | 1 | 5.60848070661657 | 0.611812368808496 |
| 4967 | STXBP5 | 1 | 1 | 4.10363890211217 | 1.55914534705709 |
| 4968 | SAMD5 | 15 | 1 | 5.12471701185338 | 0.592249766765344 |
| 4969 | AL033504.1 | 5 | 1 | 4.0896723417484 | -0.412006779732001 |
| 4970 | AL365271.1 | 1 | 1 | 3.59409953634374 | 1.26921834128927 |
| 4971 | SASH1 | 15 | 1 | 5.6562817243778 | 0.95680858272146 |
| 4972 | UST | 4 | 1 | -4.62958930452235 | -1.70907179933955 |
| 4973 | TAB2 | 9 | 1 | 0.615099146767781 | 2.97659649032186 |
| 4974 | PPIL4 | 8 | 1 | -1.51185737569697 | 0.307975188670862 |
| 4975 | GINM1 | 2 | 1 | 0.700273170872853 | 1.61311926024984 |
| 4976 | KATNA1 | 16 | 1 | -4.32238505800135 | 2.98917259353231 |
| 4977 | LATS1 | 9 | 1 | 1.05771018545263 | 3.01673616546224 |
| 4978 | AL358852.1 | 11 | 1 | -4.26689027269252 | -1.5884879670756 |
| 4979 | NUP43 | 3 | 1 | -3.43306539972193 | 0.918601170955407 |
| 4980 | PCMT1 | 10 | 1 | 3.26252914945714 | -1.63146756512095 |
| 4981 | AL355312.2 | 1 | 1 | 3.17837144415014 | 1.94616903441976 |
| 4982 | LRP11 | 2 | 1 | -0.38251775522597 | 2.21017731803488 |
| 4983 | ULBP2 | 5 | 1 | 3.18714024107091 | -0.473360194982779 |
| 4984 | ULBP3 | 14 | 1 | -6.09229014833338 | -0.821872837068331 |
| 4985 | PPP1R14C | 5 | 1 | 3.88476206342809 | -0.729522182764304 |
| 4986 | PLEKHG1 | 14 | 1 | -4.84111569841273 | 0.21840526240896 |
| 4987 | MTHFD1L | 14 | 1 | -5.6772634835995 | 0.0570731319768308 |
| 4988 | AKAP12 | 1 | 1 | 3.32698227445714 | 0.396627441821802 |
| 4989 | ZBTB2 | 17 | 1 | 0.219847991868184 | 0.0694059290273069 |
| 4990 | RMND1 | 8 | 1 | -0.603837028340175 | 0.105968848644006 |
| 4991 | ARMT1 | 13 | 1 | -2.17805157621272 | -1.76305256706644 |
| 4992 | CCDC170 | 20 | 1 | 3.25989152471654 | -3.93659268242289 |
| 4993 | SYNE1 | 1 | 1 | 4.36725641767614 | 1.47001375335287 |
| 4994 | FBXO5 | 19 | 1 | -4.94797252138026 | 2.00100912231039 |
| 4995 | MTRF1L | 3 | 1 | -2.74449919183619 | -0.277427836479437 |
| 4996 | RGS17 | 15 | 1 | 4.57996202985875 | -0.275685771526587 |
| 4997 | OPRM1 | 18 | 1 | 4.99033619444005 | -1.85890154463221 |
| 4998 | IPCEF1 | 6 | 1 | 2.64367343465917 | -1.59075914246012 |
| 4999 | CNKSR3 | 4 | 1 | -4.27276824434169 | -0.357612892212165 |
| 5000 | SCAF8 | 2 | 1 | -0.876439437464549 | 2.11518372672628 |
| 5001 | TIAM2 | 2 | 1 | -0.208959564760997 | 1.26056040900778 |
| 5002 | AL355297.4 | 8 | 1 | -2.18246220071681 | 1.3320135034902 |
| 5003 | ARID1B | 12 | 1 | 2.30511571447484 | 2.16823948997091 |
| 5004 | TMEM242 | 11 | 1 | -2.99207399804957 | -1.31217802983691 |
| 5005 | ZDHHC14 | 7 | 1 | -3.90699456651576 | -2.61006949764658 |
| 5006 | SNX9 | 3 | 1 | -2.50631330926783 | 0.100405947147119 |
| 5007 | SYNJ2 | 4 | 1 | -3.72632192094691 | 0.381391064105737 |
| 5008 | SERAC1 | 2 | 1 | 0.392687573834584 | 2.15767468589376 |
| 5009 | GTF2H5 | 3 | 1 | -2.80796287973292 | -0.507301791729224 |
| 5010 | TULP4 | 1 | 1 | 3.61998511831395 | 0.602986947475183 |
| 5011 | TMEM181 | 1 | 1 | 3.09547521154516 | 1.60114754813741 |
| 5012 | DYNLT1 | 10 | 1 | 3.01896406690709 | -1.99925790649821 |
| 5013 | EZR | 11 | 1 | -3.67395924051173 | -0.934983304859889 |
| 5014 | RSPH3 | 1 | 1 | 2.85876585523717 | 1.82221235412191 |
| 5015 | SOD2 | 12 | 1 | 2.55444742719762 | 2.33863891738485 |
| 5016 | WTAP | 2 | 1 | 0.923173919125722 | 1.85611976760458 |
| 5017 | ACAT2 | 6 | 1 | 2.41035558263891 | -1.68161074739863 |
| 5018 | TCP1 | 3 | 1 | -1.71363566835292 | -0.0736270390169741 |
| 5019 | MRPL18 | 8 | 1 | -1.15401695688136 | -0.0459136210100771 |
| 5020 | IGF2R | 8 | 1 | -1.60200129468806 | 1.09364296573232 |
| 5021 | PLG | 3 | 1 | -2.96703813989527 | 0.551057712016809 |
| 5022 | AL139393.2 | 14 | 1 | -5.82820914705165 | -0.0141671739237429 |
| 5023 | MAP3K4 | 9 | 1 | -0.0787511916912337 | 3.1172222055776 |
| 5024 | AGPAT4 | 2 | 1 | 0.280881240769551 | 1.96577610152792 |

| 5025 | PACRG | 10 | 1 | 4.68150497953527 | -0.801322131575835 |
| --- | --- | --- | --- | --- | --- |
| 5026 | CAHM | 3 | 1 | -3.32528589685328 | 0.338003770290124 |
| 5027 | QKI | 14 | 1 | -5.2211859079159 | 0.0154446520192503 |
| 5028 | C6orf118 | 4 | 1 | -4.46881745775111 | -1.06068158072401 |
| 5029 | PDE10A | 1 | 1 | 3.73594261686437 | 2.44088639396261 |
| 5030 | LINC00473 | 7 | 1 | -3.34961150606044 | -3.5935395799296 |
| 5031 | SFT2D1 | 4 | 1 | -4.10047076661952 | 0.207938805995691 |
| 5032 | MPC1 | 10 | 1 | 3.13951493780248 | -1.51559834104944 |
| 5033 | RPS6KA2 | 1 | 1 | 3.29790855924718 | 1.85041178840231 |
| 5034 | RNASET2 | 4 | 1 | -4.96273015458949 | -0.212252839626563 |
| 5035 | Z94721.1 | 16 | 1 | -4.50442813356288 | 3.01209653991293 |
| 5036 | FGFR1OP | 16 | 1 | -3.62605498750575 | 2.22988475936483 |
| 5037 | LINC02538 | 4 | 1 | -5.34644578416713 | -1.89399395805765 |
| 5038 | LINC02487 | 14 | 1 | -6.95589993913539 | -0.84561824721266 |
| 5039 | AFDN-DT | 11 | 1 | -3.44182299096949 | -1.6278550468104 |
| 5040 | AFDN | 7 | 1 | -2.8393556924618 | -2.21543525082041 |
| 5041 | DACT2 | 13 | 1 | -0.035509884551837 | -3.15785108429362 |
| 5042 | WDR27 | 9 | 1 | 1.33628894369237 | 3.06083764213155 |
| 5043 | C6orf120 | 3 | 1 | -2.98183868844874 | -0.154747887672675 |
| 5044 | PHF10 | 3 | 1 | -3.18225549180873 | 0.442256347118127 |
| 5045 | AL354892.2 | 4 | 1 | -4.76348565538295 | -1.44114772898127 |
| 5046 | TCTE3 | 4 | 1 | -3.67472861726649 | 0.225498215137231 |
| 5047 | ERMARD | 11 | 1 | -3.84755753000148 | -0.836773603381407 |
| 5048 | LINC00242 | 4 | 1 | -3.24515174348719 | 0.0453794755322813 |
| 5049 | DLL1 | 4 | 1 | -4.57132075746424 | -1.47235791307856 |
| 5050 | FAM120B | 2 | 1 | -0.751512393549754 | 2.06883038657736 |
| 5051 | PSMB1 | 13 | 1 | -1.30605803449519 | -2.21540258747507 |
| 5052 | TBP | 1 | 1 | 1.33019401113622 | 1.96096982139181 |
| 5053 | PDCD2 | 3 | 1 | -2.34200619180567 | -0.824707812787783 |
| 5054 | FAM20C | 17 | 1 | 1.67512477914922 | 0.309409991679895 |
| 5055 | PDGFA | 18 | 1 | 5.53510357420079 | -0.594698324026358 |
| 5056 | DNAAF5 | 3 | 1 | -3.10815857370265 | -0.536488576950324 |
| 5057 | SUN1 | 2 | 1 | 0.429101243420766 | 2.15914119857382 |
| 5058 | ADAP1 | 10 | 1 | 4.70488478223912 | -1.08952633959223 |
| 5059 | COX19 | 1 | 1 | 3.0668509153568 | 1.78757752555441 |
| 5060 | C7orf50 | 3 | 1 | -1.81974266488917 | -0.50947217565943 |
| 5061 | AC073957.2 | 15 | 1 | 6.05306602995031 | 0.67324437278341 |
| 5062 | GPER1 | 7 | 1 | -2.67495726068385 | -3.79594908577372 |
| 5063 | ZFAND2A | 10 | 1 | 4.41880394498937 | -1.91780374866892 |
| 5064 | AC091729.3 | 4 | 1 | -3.81822584588893 | -0.401473476709616 |
| 5065 | UNCX | 6 | 1 | 1.99394609014623 | -2.71950100284983 |
| 5066 | AC102953.2 | 12 | 1 | 2.78639246503942 | 2.31477893966268 |
| 5067 | INTS1 | 2 | 1 | -0.630739078120067 | 2.3193079389913 |
| 5068 | MAFK | 1 | 1 | 4.91616226713292 | 0.775724903522241 |
| 5069 | PSMG3 | 6 | 1 | 1.65146829168432 | -1.17510585409571 |
| 5070 | PSMG3-AS1 | 11 | 1 | -3.23352597673304 | -1.43547080379893 |
| 5071 | ELFN1 | 7 | 1 | -3.13492177446253 | -2.85130236965586 |
| 5072 | MAD1L1 | 17 | 1 | -0.00498709102041917 | 0.125861422000635 |
| 5073 | MRM2 | 8 | 1 | -1.35968790967829 | 0.12125839370321 |
| 5074 | NUDT1 | 19 | 1 | -4.05286477525599 | 1.31057275909017 |
| 5075 | EIF3B | 3 | 1 | -2.82169507463343 | 0.134820119319665 |
| 5076 | CHST12 | 17 | 1 | 0.713467672272847 | -0.755131839932692 |
| 5077 | LFNG | 4 | 1 | -4.40638564546473 | -0.7855057261603 |
| 5078 | BRAT1 | 8 | 1 | -1.36831472833522 | 0.122278586803186 |
| 5079 | IQCE | 12 | 1 | 2.18116618673436 | 1.71945633071493 |
| 5080 | TTYH3 | 1 | 1 | 2.8749084619724 | 0.358409062801111 |
| 5081 | GNA12 | 1 | 1 | 3.7504925874912 | 1.4385706343038 |
| 5082 | SDK1 | 1 | 1 | 3.7867052702152 | 1.37397421973776 |
| 5083 | FOXK1 | 2 | 1 | -0.531012311772182 | 1.7143422521932 |
| 5084 | MMD2 | 7 | 1 | -2.99085401018031 | -3.96883569580485 |
| 5085 | RBAK-RBAKDN | 17 | 1 | 1.73760594408147 | -0.710587470950377 |
| 5086 | RBAK | 9 | 1 | 0.915284052296803 | 2.87853778976034 |
| 5087 | WIPI2 | 8 | 1 | -1.02959673126109 | 0.139486566959131 |
| 5088 | SLC29A4 | 5 | 1 | 4.0747854856693 | -0.703447728575957 |
| 5089 | TNRC18 | 12 | 1 | 1.96257068197362 | 1.61489619391988 |
| 5090 | AC093620.1 | 11 | 1 | -3.93896888215907 | -2.26330207210947 |
| 5091 | AC092171.1 | 9 | 1 | 1.55379547159307 | 2.99759496825765 |

| 5092 | AC092171.5 | 10 | 1 | 3.56573082487218 | -1.91780577522684 |
| --- | --- | --- | --- | --- | --- |
| 5093 | FBXL18 | 1 | 1 | 3.73478294889562 | 2.2297972597463 |
| 5094 | ACTB | 6 | 1 | 2.27074624578588 | -2.48115204197336 |
| 5095 | FSCN1 | 17 | 1 | 2.07996298353307 | 0.278834120212304 |
| 5096 | RNF216 | 2 | 1 | -0.0572393389499922 | 2.02027858871053 |
| 5097 | CCZ1 | 17 | 1 | 0.507451787396596 | 0.440766350208032 |
| 5098 | RSPH10B | 6 | 1 | 2.33842898885839 | -1.67426632267405 |
| 5099 | PMS2 | 2 | 1 | -0.409071326450183 | 2.00622858184408 |
| 5100 | AIMP2 | 3 | 1 | -2.55011771638758 | -0.400841876091254 |
| 5101 | EIF2AK1 | 2 | 1 | -0.612236812905147 | 2.18402971404623 |
| 5102 | USP42 | 12 | 1 | 2.86987353841893 | 2.48328413146566 |
| 5103 | CYTH3 | 2 | 1 | -0.659993961647822 | 1.9061261572225 |
| 5104 | FAM220A | 1 | 1 | 3.40826179067724 | 2.04518999236654 |
| 5105 | RAC1 | 6 | 1 | -0.214518334910943 | -1.37577263933588 |
| 5106 | KDELR2 | 8 | 1 | -1.41936753709681 | 0.0796234168393491 |
| 5107 | ZDHHC4 | 3 | 1 | -1.88736186941035 | -1.12430435341765 |
| 5108 | C7orf26 | 2 | 1 | 0.924756660863088 | 1.34587992805074 |
| 5109 | ZNF853 | 2 | 1 | 1.13359238187902 | 1.17514361518453 |
| 5110 | ZNF316 | 9 | 1 | 1.92362047712438 | 3.09262408393453 |
| 5111 | ZNF12 | 9 | 1 | 1.08918262998693 | 2.90132607596945 |
| 5112 | RSPH10B2 | 6 | 1 | 2.34524036924474 | -1.70967332941462 |
| 5113 | CCZ1B | 2 | 1 | -0.0290401877916594 | 1.39719595092367 |
| 5114 | C1GALT1 | 17 | 1 | 1.93951608221166 | 0.335107580600488 |
| 5115 | AC004982.2 | 1 | 1 | 4.02955175916784 | 2.31431426185201 |
| 5116 | MIOS | 1 | 1 | 3.34912778417699 | 2.37876905578207 |
| 5117 | RPA3 | 19 | 1 | -4.194888338641 | 0.911481515346277 |
| 5118 | AC006042.2 | 8 | 1 | -2.6291723104275 | 1.30649532455038 |
| 5119 | GLCCI1 | 9 | 1 | 1.19489134828679 | 2.55101980346273 |
| 5120 | ICA1 | 10 | 1 | 4.59387852232091 | -2.10104702335764 |
| 5121 | NXPH1 | 18 | 1 | 6.30866123716466 | -2.35514686924387 |
| 5122 | NDUFA4 | 13 | 1 | -0.80466972310908 | -2.53840289932658 |
| 5123 | PHF14 | 2 | 1 | 0.758138909741567 | 1.02708329337667 |
| 5124 | THSD7A | 10 | 1 | 4.87942387144201 | -1.50334177833964 |
| 5125 | TMEM106B | 1 | 1 | 1.13689865152471 | 1.57911433356832 |
| 5126 | ARL4A | 14 | 1 | -6.8397219033993 | -0.802495821537268 |
| 5127 | ETV1 | 6 | 1 | 2.26808382551305 | -2.23666356426646 |
| 5128 | DGKB | 4 | 1 | -5.26017735917933 | -2.1206334672587 |
| 5129 | SOSTDC1 | 14 | 1 | -5.61991761644252 | 0.246364132343042 |
| 5130 | ANKMY2 | 5 | 1 | 4.1529743818485 | -0.60210306865145 |
| 5131 | BZW2 | 10 | 1 | 4.55482889692418 | -0.701479717315924 |
| 5132 | TSPAN13 | 10 | 1 | 3.64614082853429 | -1.78604523760248 |
| 5133 | SNX13 | 9 | 1 | 1.66947890798681 | 3.08393730300497 |
| 5134 | HDAC9 | 2 | 1 | -0.437330216363742 | 1.20891131537985 |
| 5135 | TWISTNB | 3 | 1 | -2.39305876214869 | 0.185753599582422 |
| 5136 | TMEM196 | 5 | 1 | 3.87501169721715 | -0.219901248039496 |
| 5137 | AC004130.1 | 4 | 1 | -4.11491368730433 | -0.945399872185481 |
| 5138 | ITGB8 | 4 | 1 | -4.89004634340174 | -0.416104539455664 |
| 5139 | SP8 | 18 | 1 | 6.1372158674442 | -2.779502852978 |
| 5140 | SP4 | 1 | 1 | 3.51394368688695 | 1.99331845420431 |
| 5141 | DNAH11 | 4 | 1 | -4.69843839128382 | -0.640221639694464 |
| 5142 | CDCA7L | 19 | 1 | -4.94404147584803 | 0.905556217609155 |
| 5143 | RAPGEF5 | 18 | 1 | 5.62899996320836 | -1.1830496035235 |
| 5144 | TOMM7 | 13 | 1 | -0.175439715579822 | -2.38636325222422 |
| 5145 | FAM126A | 8 | 1 | -1.86143063028224 | 1.4589437403066 |
| 5146 | KLHL7-DT | 19 | 1 | -3.96051548440821 | 0.754321233211267 |
| 5147 | KLHL7 | 9 | 1 | 1.35402859727971 | 2.34677423614095 |
| 5148 | NUPL2 | 8 | 1 | -0.80744414050944 | 0.723563090739953 |
| 5149 | MALSU1 | 3 | 1 | -3.34548614938624 | -0.487745925010932 |
| 5150 | IGF2BP3 | 4 | 1 | -3.71530579049952 | -0.190230055870307 |
| 5151 | TRA2A | 1 | 1 | 2.2490174917423 | 0.8020206846578 |
| 5152 | CCDC126 | 15 | 1 | 4.45445801298253 | -0.122553988518012 |
| 5153 | MPP6 | 3 | 1 | -3.39481256921656 | 0.716468945918786 |
| 5154 | GSDME | 14 | 1 | -6.3791768403805 | -0.762447743834746 |
| 5155 | OSBPL3 | 14 | 1 | -6.27373288591273 | -0.0845992408411623 |
| 5156 | CYCS | 17 | 1 | 1.11439801733129 | -0.529470636667502 |
| 5157 | NFE2L3 | 7 | 1 | -1.89843641240962 | -3.36790643555094 |
| 5158 | HNRNPA2B1 | 3 | 1 | -2.75326703508265 | 0.465718523441064 |

| 5159 | CBX3 | 5 | 1 | 3.2727584985935 | -0.912006332697165 |
| --- | --- | --- | --- | --- | --- |
| 5160 | SNX10 | 10 | 1 | 3.50615097562902 | -1.43160794598032 |
| 5161 | AC004540.2 | 5 | 1 | 4.08033300916784 | -0.727496250690711 |
| 5162 | AC004540.1 | 5 | 1 | 4.08849073927037 | -0.746563210310233 |
| 5163 | AC004947.1 | 10 | 1 | 4.62572218458287 | -2.12566362244059 |
| 5164 | SKAP2 | 18 | 1 | 4.8785021452152 | -1.80875651461054 |
| 5165 | HIBADH | 13 | 1 | -1.69429014642604 | -2.51937340122629 |
| 5166 | TAX1BP1 | 2 | 1 | -0.0393354894674559 | 1.75834907668661 |
| 5167 | JAZF1 | 15 | 1 | 5.08109165708654 | 0.379127994952905 |
| 5168 | CREB5 | 10 | 1 | 4.2868216184818 | -2.59232781750132 |
| 5169 | TRIL | 2 | 1 | -1.10018764455683 | 1.82417787688802 |
| 5170 | AC005162.3 | 15 | 1 | 5.34973885099523 | 0.310515777049768 |
| 5171 | CPVL | 15 | 1 | 5.6719100622379 | 0.0312159814221739 |
| 5172 | CHN2 | 15 | 1 | 4.76458336393468 | 0.117780224261987 |
| 5173 | AC004593.1 | 1 | 1 | 3.999043717786 | 1.37348498481344 |
| 5174 | WIPF3 | 18 | 1 | 6.12632586042516 | -2.79872427803446 |
| 5175 | SCRN1 | 18 | 1 | 5.48146511594884 | -1.94878075939585 |
| 5176 | FKBP14 | 4 | 1 | -4.46458838899501 | -0.81029323411156 |
| 5177 | PLEKHA8 | 9 | 1 | 0.562000706597493 | 2.85593094008993 |
| 5178 | MTURN | 5 | 1 | 4.43276955167882 | 0.569881335674035 |
| 5179 | ZNRF2 | 2 | 1 | 0.182111725016759 | 1.78925766128134 |
| 5180 | GGCT | 19 | 1 | -4.64798854310878 | 1.24924172538351 |
| 5181 | GARS | 10 | 1 | 4.31968571226232 | -2.10640309673716 |
| 5182 | MINDY4 | 2 | 1 | -0.615062102869823 | 0.946113602100122 |
| 5183 | ADCYAP1R1 | 18 | 1 | 6.05630327741735 | -1.84184919220377 |
| 5184 | PPP1R17 | 5 | 1 | 4.16175247709386 | -0.64677743536402 |
| 5185 | PDE1C | 18 | 1 | 5.33293749372594 | -0.611330702127707 |
| 5186 | LSM5 | 19 | 1 | -3.89562176187403 | 0.466482297359216 |
| 5187 | AVL9 | 1 | 1 | 2.76512648145788 | 1.71733917373251 |
| 5188 | AC018645.2 | 8 | 1 | -2.62602780778773 | 1.95344866889547 |
| 5189 | KBTBD2 | 12 | 1 | 2.95123578588598 | 2.13134874480795 |
| 5190 | FKBP9 | 14 | 1 | -5.76836083848841 | -0.675914376200926 |
| 5191 | NT5C3A | 5 | 1 | 3.32952715437047 | -0.75706122738291 |
| 5192 | RP9 | 11 | 1 | -2.9923782201565 | -1.70672379356791 |
| 5193 | BBS9 | 1 | 1 | 3.48440767805211 | 0.579508201060998 |
| 5194 | DPY19L1 | 7 | 1 | -3.4083573671139 | -2.97028218132426 |
| 5195 | HERPUD2 | 8 | 1 | -1.48248241861232 | 1.67207326072286 |
| 5196 | AC018647.2 | 2 | 1 | -0.0829289110458632 | 2.37183441299032 |
| 5197 | Sep-07 | 10 | 1 | 3.35820676366918 | -1.52793787342478 |
| 5198 | EEPD1 | 6 | 1 | 2.09282018224828 | -1.95493064743449 |
| 5199 | KIAA0895 | 2 | 1 | 0.843977108403371 | 1.85380710738729 |
| 5200 | ANLN | 16 | 1 | -4.57628415544398 | 3.18014849799703 |
| 5201 | AC007349.2 | 7 | 1 | -3.71402286012538 | -3.49589143616129 |
| 5202 | ELMO1 | 8 | 1 | -1.45039020498164 | -0.29696498733927 |
| 5203 | EPDR1 | 11 | 1 | -3.56047223527796 | -2.24461637360026 |
| 5204 | STARD3NL | 6 | 1 | 0.239900395079778 | -1.20916078907419 |
| 5205 | AMPH | 1 | 1 | 4.62653423826329 | 0.637751714168298 |
| 5206 | VPS41 | 12 | 1 | 2.1742203382694 | 2.76858986991476 |
| 5207 | POU6F2 | 15 | 1 | 5.0937268881046 | 0.794502273975122 |
| 5208 | YAE1D1 | 8 | 1 | -1.51285944898493 | -0.103448911728156 |
| 5209 | RALA | 7 | 1 | -2.87759183366664 | -3.94693909508158 |
| 5210 | CDK13 | 9 | 1 | 0.407609239026235 | 2.70333875793051 |
| 5211 | MPLKIP | 3 | 1 | -2.41678212602503 | -0.668175055803549 |
| 5212 | SUGCT | 4 | 1 | -4.82602714021571 | -0.535223527969611 |
| 5213 | INHBA | 18 | 1 | 5.46464754621618 | -0.61016769629885 |
| 5214 | INHBA-AS1 | 18 | 1 | 5.46675945798986 | -1.0339909635203 |
| 5215 | GLI3 | 4 | 1 | -4.37548612077601 | -0.645554467262519 |
| 5216 | PSMA2 | 13 | 1 | -0.486831069186999 | -2.11522804123331 |
| 5217 | MRPL32 | 13 | 1 | -1.23614196498759 | -1.40634272915293 |
| 5218 | HECW1 | 5 | 1 | 3.88831020872228 | 0.273270861087549 |
| 5219 | STK17A | 11 | 1 | -3.2259216161526 | -2.3914595685618 |
| 5220 | COA1 | 19 | 1 | -3.99801348169215 | 0.639954821048486 |
| 5221 | BLVRA | 13 | 1 | -1.99292598684199 | -1.987905486645 |
| 5222 | SPDYE1 | 1 | 1 | 1.99184943716161 | 0.952411905704248 |
| 5223 | AC004951.1 | 12 | 1 | 2.43267036954992 | 1.77915038245748 |
| 5224 | LINC00957 | 5 | 1 | 3.76602341215246 | -0.89141535588522 |
| 5225 | DBNL | 2 | 1 | 0.140788242026494 | 1.1553229250295 |

| 5226 | PGAM2 | 7 | 1 | -2.44208286722071 | -3.35571084839274 |
| --- | --- | --- | --- | --- | --- |
| 5227 | AEBP1 | 14 | 1 | -5.64860747774012 | -0.571240618051779 |
| 5228 | POLD2 | 8 | 1 | -1.53675626237757 | 0.139499083934533 |
| 5229 | GCK | 15 | 1 | 4.57430340330236 | -0.155961140217078 |
| 5230 | YKT6 | 2 | 1 | -0.721837863520457 | 1.81389774459432 |
| 5231 | CAMK2B | 18 | 1 | 5.06293751279943 | -0.730811833562148 |
| 5232 | NUDCD3 | 1 | 1 | 3.40090157072179 | 1.35878814834188 |
| 5233 | DDX56 | 8 | 1 | -0.715419665173366 | -0.19496337992121 |
| 5234 | TMED4 | 7 | 1 | -2.62639258821376 | -2.0576041780131 |
| 5235 | OGDH | 2 | 1 | 0.469135895177052 | 1.93547715323995 |
| 5236 | ZMIZ2 | 1 | 1 | 3.83820892850988 | 1.88676966804098 |
| 5237 | PPIA | 11 | 1 | -2.96412847955592 | -1.21497107964922 |
| 5238 | H2AFV | 4 | 1 | -3.84332679231532 | -0.616563513101828 |
| 5239 | PURB | 9 | 1 | -0.0588629545725127 | 2.6424588121755 |
| 5240 | SNHG15 | 5 | 1 | 2.79643394033544 | -0.720721139730704 |
| 5241 | CCM2 | 7 | 1 | -2.71194075067408 | -2.86046026569773 |
| 5242 | NACAD | 10 | 1 | 3.4433055071079 | -1.18554915410448 |
| 5243 | TBRG4 | 8 | 1 | -0.569648161724879 | -0.328163250507605 |
| 5244 | ADCY1 | 1 | 1 | 3.5985469965183 | 1.04767991679739 |
| 5245 | IGFBP3 | 14 | 1 | -6.93917869050868 | -0.793074882747424 |
| 5246 | TNS3 | 4 | 1 | -4.61429332216151 | -0.0322016916887881 |
| 5247 | HUS1 | 3 | 1 | -2.74722789247401 | 0.426974193034875 |
| 5248 | C7orf57 | 13 | 1 | -0.43405164797671 | -3.2812312684672 |
| 5249 | UPP1 | 14 | 1 | -6.38702558000453 | -0.346999689163458 |
| 5250 | ABCA13 | 18 | 1 | 5.37256599943273 | -2.02677689415385 |
| 5251 | VWC2 | 18 | 1 | 6.19949318449132 | -2.17281494957377 |
| 5252 | IKZF1 | 14 | 1 | -5.98509667833216 | 0.0710443415028928 |
| 5253 | FIGNL1 | 1 | 1 | 3.71789623777501 | 1.30711902755331 |
| 5254 | DDC | 10 | 1 | 3.69803644697301 | -1.26590762955118 |
| 5255 | GRB10 | 1 | 1 | 3.16498543302648 | 2.28506030219625 |
| 5256 | VSTM2A | 18 | 1 | 5.83940722028844 | -2.05702362877299 |
| 5257 | SEC61G | 13 | 1 | -0.180061809912993 | -2.34757922512461 |
| 5258 | EGFR | 14 | 1 | -6.53806184251673 | -1.11629216295649 |
| 5259 | LANCL2 | 1 | 1 | 4.13873220960729 | 1.47595514434408 |
| 5260 | VOPP1 | 15 | 1 | 4.68736101667516 | -0.350950583042395 |
| 5261 | ZNF713 | 1 | 1 | 4.06830336134069 | 1.91506327766012 |
| 5262 | NIPSNAP2 | 17 | 1 | 1.06018437425725 | -0.174214287819159 |
| 5263 | MRPS17 | 8 | 1 | -1.18875746448405 | -0.220273836673987 |
| 5264 | PSPH | 3 | 1 | -2.82275579889186 | -0.49148352366854 |
| 5265 | CCT6A | 3 | 1 | -2.38184617479212 | -0.569825603784812 |
| 5266 | PHKG1 | 1 | 1 | 2.55303336660497 | 0.48789931911062 |
| 5267 | CHCHD2 | 13 | 1 | -0.462456196740939 | -1.96714864593912 |
| 5268 | ZNF680 | 8 | 1 | -1.32947121580012 | 0.907463208614099 |
| 5269 | ZNF107 | 16 | 1 | -3.42639230211146 | 1.56411947387289 |
| 5270 | ZNF138 | 1 | 1 | 3.51604129354589 | 2.49810494559835 |
| 5271 | ZNF273 | 3 | 1 | -3.31656430681117 | 1.33142007964681 |
| 5272 | ZNF117 | 9 | 1 | 1.32156087438695 | 2.59301151412557 |
| 5273 | ERV3-1 | 2 | 1 | 0.566838696404622 | 1.44872416633199 |
| 5274 | AC104073.4 | 19 | 1 | -4.72932170350917 | 1.79779185432028 |
| 5275 | ZNF92 | 9 | 1 | 1.0765880493366 | 2.15081800597738 |
| 5276 | VKORC1L1 | 12 | 1 | 2.94427229444616 | 2.5117637075765 |
| 5277 | GUSB | 19 | 1 | -4.00276182611354 | 1.26247848647665 |
| 5278 | ASL | 6 | 1 | 2.68932224790685 | -1.35168076974322 |
| 5279 | CRCP | 1 | 1 | 3.5834570078098 | 0.598054782329309 |
| 5280 | TPST1 | 5 | 1 | 3.51259066145055 | -0.334637149395239 |
| 5281 | AC008267.5 | 4 | 1 | -3.65965937097438 | -0.441328092636359 |
| 5282 | KCTD7 | 1 | 1 | 3.80479313413732 | 0.443945304332483 |
| 5283 | RABGEF1 | 12 | 1 | 2.90955568830602 | 2.42252506393026 |
| 5284 | AC027644.3 | 4 | 1 | -3.57499979455836 | -0.651850476087821 |
| 5285 | AC073335.2 | 11 | 1 | -3.69187234361537 | -1.16034801227022 |
| 5286 | TMEM248 | 8 | 1 | -1.14005820472605 | 0.296585694728601 |
| 5287 | SBDS | 10 | 1 | 3.84968639890783 | -1.60103414875437 |
| 5288 | TYW1 | 3 | 1 | -2.05676876027949 | 0.41814138072561 |
| 5289 | AC006480.2 | 1 | 1 | 3.42055608312719 | 0.980224148212182 |
| 5290 | AUTS2 | 12 | 1 | 2.43488742391698 | 2.96527947562765 |
| 5291 | GALNT17 | 15 | 1 | 5.46464182417028 | 0.481884137569177 |
| 5292 | POM121 | 9 | 1 | 0.470225468083547 | 2.86340822356771 |

| 5293 | AC211476.2 | 16 | 1 | -4.11326478441127 | 2.79397620338033 |
| --- | --- | --- | --- | --- | --- |
| 5294 | NSUN5 | 8 | 1 | -0.413458049491717 | -0.196769817890418 |
| 5295 | FZD9 | 14 | 1 | -6.306620344714 | -0.654096215190184 |
| 5296 | BAZ1B | 2 | 1 | 0.372984662457631 | 2.46885384696554 |
| 5297 | BCL7B | 8 | 1 | -0.849362358645274 | -0.225187583984625 |
| 5298 | TBL2 | 3 | 1 | -1.93033538778193 | -0.355987235130561 |
| 5299 | VPS37D | 8 | 1 | -0.696586415366008 | -0.32593409639765 |
| 5300 | DNAJC30 | 3 | 1 | -1.81572030504115 | -0.88900469352473 |
| 5301 | BUD23 | 3 | 1 | -2.63185761888392 | -0.777623309911978 |
| 5302 | STX1A | 10 | 1 | 3.21643401662938 | -1.80517290455271 |
| 5303 | ABHD11 | 4 | 1 | -4.71887134034999 | -1.42933116775919 |
| 5304 | CLDN3 | 4 | 1 | -4.71132205446131 | -1.23317800384928 |
| 5305 | CLDN4 | 11 | 1 | -3.61442564447291 | -1.74082581621577 |
| 5306 | METTL27 | 11 | 1 | -3.65760348756678 | -1.78786264282633 |
| 5307 | ELN | 4 | 1 | -4.31798814256556 | -0.855832609506142 |
| 5308 | LIMK1 | 6 | 1 | 2.42274190466039 | -1.11002956253458 |
| 5309 | EIF4H | 3 | 1 | -1.9650219531811 | -0.00218211037088645 |
| 5310 | RFC2 | 19 | 1 | -4.54838584382899 | 1.60489548820089 |
| 5311 | CLIP2 | 1 | 1 | 4.24496342222326 | 1.63148178237509 |
| 5312 | GTF2I | 1 | 1 | 2.95669318716161 | 1.76746143477987 |
| 5313 | GTF2IRD2 | 9 | 1 | 0.998732104703114 | 2.70704139846395 |
| 5314 | CASTOR2 | 1 | 1 | 3.16917802373998 | 1.16183222907613 |
| 5315 | RCC1L | 3 | 1 | -2.93175528962977 | -0.568054600776923 |
| 5316 | GTF2IRD2B | 9 | 1 | 1.94055248777501 | 2.85385598319601 |
| 5317 | AC211486.5 | 1 | 1 | 2.69514252225988 | 1.87157167571615 |
| 5318 | TRIM73 | 2 | 1 | -0.223993934885337 | 2.01701988357137 |
| 5319 | POM121C | 9 | 1 | 0.470083072587178 | 2.77491821425985 |
| 5320 | HIP1 | 3 | 1 | -2.83284018953211 | 0.317288652835595 |
| 5321 | RHBDD2 | 6 | 1 | 2.03593350927465 | -1.214452549042 |
| 5322 | POR | 17 | 1 | 1.49841631929509 | -0.489264532150519 |
| 5323 | TMEM120A | 10 | 1 | 3.9789774564945 | -1.41895262819697 |
| 5324 | MDH2 | 6 | 1 | -0.0493052454746504 | -1.12179508727957 |
| 5325 | SRRM3 | 5 | 1 | 3.88358189146154 | 0.0685300387723325 |
| 5326 | HSPB1 | 11 | 1 | -3.81553505380519 | -1.84001199823786 |
| 5327 | YWHAG | 1 | 1 | 3.68236733953588 | 0.487189666209924 |
| 5328 | CCDC146 | 6 | 1 | 1.15541543047063 | -2.0046176753657 |
| 5329 | PTPN12 | 9 | 1 | 0.486055627270863 | 2.65396632331442 |
| 5330 | APTR | 6 | 1 | 1.33047641794317 | -1.65264753920008 |
| 5331 | RSBN1L | 5 | 1 | 3.67900921384923 | -0.860177355976594 |
| 5332 | TMEM60 | 16 | 1 | -3.34009574373133 | 2.28042830604147 |
| 5333 | PHTF2 | 1 | 1 | 4.21180416624181 | 1.58038963454794 |
| 5334 | AC004990.1 | 5 | 1 | 4.20309473554723 | -0.0942862473147036 |
| 5335 | MAGI2 | 15 | 1 | 5.27413488905065 | 0.543072954593408 |
| 5336 | GNAI1 | 5 | 1 | 4.39292933027379 | -0.375325366081488 |
| 5337 | SEMA3C | 18 | 1 | 6.13715864698522 | -2.21747504097391 |
| 5338 | CACNA2D1 | 5 | 1 | 3.20081616918676 | -0.541933729471457 |
| 5339 | AC006145.1 | 1 | 1 | 1.84800936262243 | 0.955222383914697 |
| 5340 | PCLO | 1 | 1 | 4.59710098783605 | 0.851240769801843 |
| 5341 | SEMA3E | 14 | 1 | -6.92037127931483 | -0.680428042234671 |
| 5342 | SEMA3A | 1 | 1 | 3.0014417318546 | 1.75713290351461 |
| 5343 | SEMA3D | 14 | 1 | -6.11976693590052 | 0.0690519370419859 |
| 5344 | GRM3 | 6 | 1 | 2.18972398321264 | -2.23752938133646 |
| 5345 | KIAA1324L | 12 | 1 | 2.62250329534643 | 2.03806532996725 |
| 5346 | AC005076.1 | 19 | 1 | -3.95639441926844 | 0.955736175952661 |
| 5347 | DMTF1 | 9 | 1 | 0.386786892815755 | 2.78775657790731 |
| 5348 | TMEM243 | 3 | 1 | -3.56958435495265 | 0.825577155528772 |
| 5349 | TP53TG1 | 4 | 1 | -4.29713176210292 | -1.45435659510065 |
| 5350 | CROT | 4 | 1 | -4.41251300294764 | -1.17274536115099 |
| 5351 | ABCB1 | 10 | 1 | 4.58943440000646 | -1.67512635809351 |
| 5352 | RUNDC3B | 15 | 1 | 4.79168821851842 | -0.319106324733985 |
| 5353 | SLC25A40 | 16 | 1 | -3.18343685586817 | 2.36687673705648 |
| 5354 | DBF4 | 16 | 1 | -4.55595849473841 | 2.52343954223226 |
| 5355 | ADAM22 | 1 | 1 | 3.19815255682103 | 1.70888079780172 |
| 5356 | SRI | 13 | 1 | -1.27261374910243 | -2.50485740524699 |
| 5357 | AC003991.2 | 4 | 1 | -3.86099837739833 | -0.326922102989447 |
| 5358 | AC003991.1 | 2 | 1 | -0.681690260962321 | 1.79023970740865 |
| 5359 | STEAP1 | 14 | 1 | -5.57303164918788 | 0.228493706165063 |

| 5360 | GTPBP10 | 9 | 1 | 0.443127706452535 | 2.80546225684713 |
| --- | --- | --- | --- | --- | --- |
| 5361 | CLDN12 | 9 | 1 | 0.448323741360829 | 2.59696234839987 |
| 5362 | CDK14 | 6 | 1 | 1.09258438627355 | -2.79118572098185 |
| 5363 | FZD1 | 7 | 1 | -3.71192215402491 | -3.39151512009074 |
| 5364 | MTERF1 | 2 | 1 | -0.194417923883273 | 0.955292002139795 |
| 5365 | AKAP9 | 1 | 1 | 3.93753887693517 | 1.98018111365865 |
| 5366 | CYP51A1 | 2 | 1 | -0.579073891238048 | 1.23758377212118 |
| 5367 | KRIT1 | 8 | 1 | -1.42000220735438 | 1.57470263618063 |
| 5368 | ANKIB1 | 1 | 1 | 3.33317328970067 | 2.38489951270651 |
| 5369 | GATAD1 | 2 | 1 | -0.28954572622545 | 1.54713358062338 |
| 5370 | PEX1 | 2 | 1 | 0.368277147217915 | 2.14566315787863 |
| 5371 | FAM133B | 8 | 1 | -1.70803795774348 | 1.88877357619833 |
| 5372 | CDK6 | 18 | 1 | 5.70906998197667 | -2.33594928604533 |
| 5373 | AC002454.1 | 14 | 1 | -6.50720189531214 | -0.321554347099555 |
| 5374 | VPS50 | 1 | 1 | 4.08999659101598 | 1.389970675884 |
| 5375 | GNGT1 | 14 | 1 | -5.7193891855038 | -0.821879773558867 |
| 5376 | TFPI2 | 14 | 1 | -6.28076623399623 | -0.00303856951166404 |
| 5377 | GNG11 | 14 | 1 | -5.78004334886439 | -0.640457167448294 |
| 5378 | BET1 | 3 | 1 | -3.28162048776515 | -0.711273416103613 |
| 5379 | COL1A2 | 14 | 1 | -6.19950936754115 | 0.117580667911279 |
| 5380 | CASD1 | 12 | 1 | 2.60494638959997 | 2.18698991912435 |
| 5381 | SGCE | 1 | 1 | 2.91249252836339 | 1.79435481208395 |
| 5382 | PEG10 | 10 | 1 | 3.60388328115575 | -1.64579372030665 |
| 5383 | PPP1R9A | 12 | 1 | 2.55019856969945 | 2.96420039313863 |
| 5384 | PON1 | 6 | 1 | 2.14048697034948 | -2.43291376453806 |
| 5385 | PON2 | 11 | 1 | -3.51308153589137 | -1.69591180902888 |
| 5386 | AC004012.1 | 6 | 1 | 1.49981392900579 | -1.85010896545817 |
| 5387 | ASB4 | 9 | 1 | 1.16284324209325 | 2.12713755744528 |
| 5388 | DYNC1I1 | 5 | 1 | 3.94018913786046 | -0.617367490352881 |
| 5389 | SLC25A13 | 11 | 1 | -3.26557372529872 | -1.91721282822062 |
| 5390 | SEM1 | 11 | 1 | -2.82882379014857 | -1.57577626329829 |
| 5391 | DLX6-AS1 | 18 | 1 | 6.26891781370275 | -2.42382357937266 |
| 5392 | DLX6 | 18 | 1 | 6.27545620481603 | -2.47251711708475 |
| 5393 | DLX5 | 18 | 1 | 6.29011847059362 | -2.5063117585795 |
| 5394 | SDHAF3 | 19 | 1 | -3.97658346612818 | 0.499248877940881 |
| 5395 | TAC1 | 18 | 1 | 5.5578439382755 | -1.65968958956171 |
| 5396 | ASNS | 10 | 1 | 4.52031303922765 | -1.93615911823679 |
| 5397 | LMTK2 | 12 | 1 | 3.11963750402562 | 2.74464739936422 |
| 5398 | TECPR1 | 12 | 1 | 2.42436362783544 | 2.22860087531637 |
| 5399 | BRI3 | 10 | 1 | 2.80655385534398 | -1.95540676933695 |
| 5400 | BAIAP2L1 | 19 | 1 | -5.04877065141566 | 1.04924704211782 |
| 5401 | NPTX2 | 7 | 1 | -2.55715440233119 | -3.95451269966532 |
| 5402 | TMEM130 | 15 | 1 | 4.5913174299442 | 0.344683782039392 |
| 5403 | TRRAP | 9 | 1 | 0.27675630132787 | 2.66250290053915 |
| 5404 | SMURF1 | 1 | 1 | 3.77337433378331 | 1.66636909621786 |
| 5405 | ARPC1A | 6 | 1 | 0.936719909115956 | -2.19637261253763 |
| 5406 | ARPC1B | 13 | 1 | -1.75118468721278 | -2.27873240333964 |
| 5407 | PDAP1 | 13 | 1 | -0.729540035322978 | -2.06074808460642 |
| 5408 | BUD31 | 6 | 1 | 0.860927596493886 | -1.35493455749918 |
| 5409 | CPSF4 | 3 | 1 | -2.00508664567836 | -0.277724608005774 |
| 5410 | ATP5MF | 6 | 1 | 0.199898496075795 | -2.32341848236491 |
| 5411 | ZNF789 | 1 | 1 | 4.65081669370763 | 0.765163675723779 |
| 5412 | ZNF394 | 2 | 1 | -0.26808313113518 | 0.763990060268152 |
| 5413 | ZKSCAN5 | 2 | 1 | 0.349261656209157 | 2.466071740566 |
| 5414 | FAM200A | 2 | 1 | -0.238027768403961 | 1.08178807872366 |
| 5415 | ZNF655 | 9 | 1 | 1.56961251775853 | 3.06416930335592 |
| 5416 | ZSCAN25 | 2 | 1 | -0.322367206410243 | 1.61209859031271 |
| 5417 | CYP3A5 | 12 | 1 | 2.71538712064855 | 2.66501869338583 |
| 5418 | AC069294.1 | 8 | 1 | -2.48502062280543 | 1.35296477454733 |
| 5419 | TRIM4 | 17 | 1 | 0.601664140626119 | 0.0434441723210691 |
| 5420 | ZKSCAN1 | 9 | 1 | -0.133584007815196 | 2.57204569953512 |
| 5421 | ZNF3 | 2 | 1 | -0.390940383271052 | 1.07722033637594 |
| 5422 | COPS6 | 6 | 1 | 1.42173589746587 | -1.4294828019755 |
| 5423 | MCM7 | 19 | 1 | -4.32396291215785 | 1.00013245719503 |
| 5424 | AP4M1 | 15 | 1 | 5.10140730421178 | 0.181761638103235 |
| 5425 | TAF6 | 2 | 1 | -0.435688957766368 | 1.93831767219137 |
| 5426 | CNPY4 | 3 | 1 | -2.01208172757991 | -0.235976799549353 |

| 5427 | LAMTOR4 | 6 | 1 | 0.216346367999242 | -2.22396527153422 |
| --- | --- | --- | --- | --- | --- |
| 5428 | GAL3ST4 | 5 | 1 | 4.11120106260412 | -0.647458478035224 |
| 5429 | GPC2 | 5 | 1 | 3.110132232114 | 0.105618969379175 |
| 5430 | STAG3 | 1 | 1 | 3.17122413198583 | 1.02779628413748 |
| 5431 | CASTOR3 | 5 | 1 | 3.75991751234166 | -0.78085095358063 |
| 5432 | SPDYE3 | 9 | 1 | 1.10728456060521 | 2.45399464744161 |
| 5433 | PILRB | 9 | 1 | -0.115418181017711 | 3.21893824714254 |
| 5434 | MEPCE | 16 | 1 | -3.57646678407557 | 2.2380923189504 |
| 5435 | PPP1R35 | 3 | 1 | -3.73127578218348 | 1.08020676749777 |
| 5436 | TSC22D4 | 7 | 1 | -2.95180939157374 | -2.91937385422159 |
| 5437 | NYAP1 | 15 | 1 | 4.48062302152745 | -0.130054160179389 |
| 5438 | SAP25 | 5 | 1 | 2.82381083051793 | 0.0818287290913938 |
| 5439 | LRCH4 | 1 | 1 | 1.78634668867223 | 1.50901474135946 |
| 5440 | PCOLCE | 6 | 1 | 2.65122820417516 | -1.76754985672404 |
| 5441 | MOSPD3 | 10 | 1 | 2.89321114103429 | -2.10140190941263 |
| 5442 | TFR2 | 15 | 1 | 4.58987738172643 | 0.425450817523706 |
| 5443 | ACTL6B | 10 | 1 | 4.07764793912999 | -1.40521214825083 |
| 5444 | GNB2 | 10 | 1 | 3.06376172582738 | -1.97893069607188 |
| 5445 | GIGYF1 | 1 | 1 | 2.52628661672704 | 0.535924331126916 |
| 5446 | POP7 | 6 | 1 | 1.64421834032171 | -1.19800434929301 |
| 5447 | EPHB4 | 4 | 1 | -4.18245719392665 | -0.69881133657862 |
| 5448 | SLC12A9 | 3 | 1 | -1.81941507776149 | -0.31382267099787 |
| 5449 | TRIP6 | 4 | 1 | -5.07021830995448 | -0.168063505710852 |
| 5450 | SRRT | 3 | 1 | -3.16856645067103 | 0.276525870738733 |
| 5451 | ACHE | 10 | 1 | 4.52700687925451 | -1.72800926786829 |
| 5452 | AC254629.1 | 7 | 1 | -2.39187882860072 | -3.79931555611063 |
| 5453 | MUC12 | 14 | 1 | -6.53233169038661 | -0.369186147274268 |
| 5454 | AC105446.1 | 6 | 1 | 1.53067435304754 | -2.63927195888926 |
| 5455 | TRIM56 | 4 | 1 | -4.79731820543177 | -0.574464394869101 |
| 5456 | AP1S1 | 10 | 1 | 4.02735163251989 | -1.32962711078097 |
| 5457 | VGF | 10 | 1 | 5.03389622251622 | -1.32754884582926 |
| 5458 | NAT16 | 1 | 1 | 4.70553041975133 | 0.64305605071615 |
| 5459 | PLOD3 | 8 | 1 | -1.84481893499263 | 0.839949861942041 |
| 5460 | ZNHIT1 | 10 | 1 | 2.93081332723729 | -1.81788800579478 |
| 5461 | CLDN15 | 1 | 1 | 3.21204210798375 | 0.417065874515283 |
| 5462 | FIS1 | 6 | 1 | 0.912000909253285 | -2.26263569218088 |
| 5463 | AC006329.1 | 4 | 1 | -4.49116872270472 | -0.80996035707165 |
| 5464 | IFT22 | 13 | 1 | -0.966398582056834 | -2.66115878445078 |
| 5465 | COL26A1 | 2 | 1 | 0.852469816609548 | 0.925856725154626 |
| 5466 | CUX1 | 9 | 1 | 0.239462718173192 | 2.70568265098165 |
| 5467 | SH2B2 | 10 | 1 | 4.55102564375036 | -0.974384031416667 |
| 5468 | PRKRIP1 | 2 | 1 | 0.63216276447408 | 1.26817669051717 |
| 5469 | ORAI2 | 1 | 1 | 2.95839692632787 | 1.16086210387777 |
| 5470 | LRWD1 | 17 | 1 | 0.223837598963902 | -0.290144845070136 |
| 5471 | POLR2J | 13 | 1 | -0.854557678297832 | -2.00518904549052 |
| 5472 | POLR2J3.1 | 17 | 1 | 1.4947210697376 | 0.601032153545129 |
| 5473 | RASA4 | 1 | 1 | 2.13870669881933 | 1.15412677901815 |
| 5474 | FAM185A | 9 | 1 | 1.32122565786474 | 2.26702751296591 |
| 5475 | FBXL13 | 1 | 1 | 2.56184078733556 | 1.44875182288717 |
| 5476 | LRRC17 | 11 | 1 | -3.94545863588221 | -1.61437736374308 |
| 5477 | NFE4 | 1 | 1 | 1.3664080051624 | 1.74676527160238 |
| 5478 | ARMC10 | 11 | 1 | -3.19301174600489 | -1.48459051472117 |
| 5479 | NAPEPLD | 12 | 1 | 3.2478332666599 | 2.77215065139364 |
| 5480 | PMPCB | 11 | 1 | -3.15953634698756 | -1.85673718077112 |
| 5481 | DNAJC2 | 3 | 1 | -2.49440573175318 | 0.151377812801111 |
| 5482 | PSMC2 | 13 | 1 | -1.15626041610606 | -0.818863733829749 |
| 5483 | RELN | 15 | 1 | 4.66230799238317 | 0.147875324664819 |
| 5484 | ORC5 | 8 | 1 | -0.925547525481059 | 0.601122871814477 |
| 5485 | LHFPL3 | 15 | 1 | 5.00644375364415 | 0.522038237033594 |
| 5486 | KMT2E | 1 | 1 | 3.54690910856359 | 0.682158008990991 |
| 5487 | AC005070.3 | 1 | 1 | 2.39055229704015 | 0.537096516071069 |
| 5488 | SRPK2 | 10 | 1 | 3.24639393369787 | -1.58695541244913 |
| 5489 | AC004884.2 | 2 | 1 | 0.460935130520986 | 2.07839669364523 |
| 5490 | PUS7 | 4 | 1 | -4.31984971483119 | -0.160758122028601 |
| 5491 | EFCAB10 | 13 | 1 | -1.15171550233729 | -2.34938011986185 |
| 5492 | ATXN7L1 | 12 | 1 | 3.43391920606725 | 2.75528039115499 |
| 5493 | SYPL1 | 14 | 1 | -6.07868789155848 | -0.55938444954325 |

| 5494 | NAMPT | 12 | 1 | 2.6481816915714 | 1.79718650954794 |
| --- | --- | --- | --- | --- | --- |
| 5495 | CCDC71L | 2 | 1 | 0.256413832112477 | 0.933703199802148 |
| 5496 | PRKAR2B | 10 | 1 | 4.10934378187291 | -1.06831236106325 |
| 5497 | HBP1 | 11 | 1 | -2.73365567644007 | -1.79263900143076 |
| 5498 | COG5 | 8 | 1 | -1.55531559903987 | 1.3116847433431 |
| 5499 | GPR22 | 18 | 1 | 5.17393471281163 | -1.32712860328127 |
| 5500 | DUS4L | 3 | 1 | -2.54938267190821 | 0.648895160136926 |
| 5501 | BCAP29 | 11 | 1 | -3.46568916757472 | -1.23999898058344 |
| 5502 | AC002467.1 | 4 | 1 | -3.83849118669398 | -0.448486044229758 |
| 5503 | CBLL1 | 9 | 1 | 0.338774457379506 | 2.78851451057028 |
| 5504 | DLD | 8 | 1 | -0.603727206782176 | -0.240445538582099 |
| 5505 | NRCAM | 5 | 1 | 3.37510134260289 | 0.100942269740808 |
| 5506 | PNPLA8 | 5 | 1 | 4.1388085035526 | 0.495180384097803 |
| 5507 | THAP5 | 2 | 1 | 1.30604530851476 | 0.95456339496206 |
| 5508 | DNAJB9 | 5 | 1 | 3.92818571607702 | -0.576993568958533 |
| 5509 | IMMP2L | 13 | 1 | -1.85674487073786 | -2.37816105705668 |
| 5510 | LRRN3 | 15 | 1 | 4.71990276853673 | -0.10601781231333 |
| 5511 | DOCK4 | 1 | 1 | 4.07573153059118 | 0.876517192302453 |
| 5512 | DOCK4-AS1 | 12 | 1 | 2.44434072057836 | 1.89690412658285 |
| 5513 | ZNF277 | 2 | 1 | 1.09634102861516 | 1.82797898429464 |
| 5514 | IFRD1 | 10 | 1 | 4.05286289732091 | -2.23598740917612 |
| 5515 | TMEM168 | 8 | 1 | -1.96829877813227 | 1.60097374099325 |
| 5516 | BMT2 | 9 | 1 | 1.31004704515569 | 3.03606356757711 |
| 5517 | GPR85 | 18 | 1 | 5.31614853422277 | -0.584724619211447 |
| 5518 | SMIM30 | 11 | 1 | -3.78654550035365 | -0.86338995692302 |
| 5519 | FOXP2 | 10 | 1 | 4.80200268308751 | -1.45997600180079 |
| 5520 | MDFIC | 19 | 1 | -4.59184192140467 | 1.52826084273885 |
| 5521 | TES | 5 | 1 | 2.86937095205419 | -1.17941377979685 |
| 5522 | CAPZA2 | 8 | 1 | -0.986842677191569 | -0.162741406978858 |
| 5523 | ST7-AS1 | 3 | 1 | -3.11096237619288 | -0.743920727791083 |
| 5524 | ST7 | 10 | 1 | 4.56271578352086 | -0.899367364747268 |
| 5525 | CTTNBP2 | 15 | 1 | 5.12634684126012 | 0.630318418918359 |
| 5526 | LSM8 | 4 | 1 | -3.39226411302455 | -0.0651245556490542 |
| 5527 | KCND2 | 15 | 1 | 5.95555330793493 | 0.676291362224328 |
| 5528 | TSPAN12 | 4 | 1 | -5.0053827615536 | -0.578960283817542 |
| 5529 | ING3 | 4 | 1 | -3.4330270143307 | -0.150236710132849 |
| 5530 | FAM3C | 2 | 1 | 0.999660268231557 | 1.14121164458822 |
| 5531 | PTPRZ1 | 14 | 1 | -5.73131392915614 | -0.680913820089591 |
| 5532 | AASS | 11 | 1 | -3.68265031297572 | -1.7214447699206 |
| 5533 | FEZF1 | 7 | 1 | -3.51142285783656 | -2.45323847156931 |
| 5534 | FEZF1-AS1 | 13 | 1 | -1.71741066892512 | -2.62205587250162 |
| 5535 | CADPS2 | 11 | 1 | -3.6603948922909 | -1.51904956919123 |
| 5536 | IQUB | 13 | 1 | -0.232460443124845 | -2.94271002632548 |
| 5537 | NDUFA5 | 5 | 1 | 3.59060861150853 | -1.02869848829676 |
| 5538 | LMOD2 | 8 | 1 | -1.40406714399226 | 0.489969507632959 |
| 5539 | WASL | 2 | 1 | -0.257708399750962 | 1.42579592841696 |
| 5540 | AC006333.2 | 4 | 1 | -3.82998155076869 | -0.573933615507376 |
| 5541 | TMEM229A | 15 | 1 | 5.59328867475621 | 0.506561294971216 |
| 5542 | GPR37 | 15 | 1 | 5.69666458646886 | 0.0500676788670896 |
| 5543 | POT1 | 16 | 1 | -3.68486331422694 | 2.42502249854635 |
| 5544 | POT1-AS1 | 5 | 1 | 3.915463223859 | -0.462589486660254 |
| 5545 | GRM8 | 15 | 1 | 5.49273706953161 | 0.414398328243005 |
| 5546 | AC000099.1 | 1 | 1 | 3.10887886564367 | 1.67852486747335 |
| 5547 | ZNF800 | 2 | 1 | 1.12574256937139 | 1.33764995711874 |
| 5548 | GCC1 | 9 | 1 | 1.57272841016881 | 2.931554452358 |
| 5549 | ARF5 | 6 | 1 | 1.48566628973119 | -1.48965434652735 |
| 5550 | SND1 | 2 | 1 | 0.152352943822072 | 1.13158048766683 |
| 5551 | SND1-IT1 | 12 | 1 | 2.13731671850316 | 2.9221047319753 |
| 5552 | LRRC4 | 9 | 1 | 0.242301687403844 | 3.08160056251119 |
| 5553 | RBM28 | 3 | 1 | -2.26406286676295 | 0.914377585826623 |
| 5554 | PRRT4 | 15 | 1 | 5.35762144605748 | 0.0482899703366636 |
| 5555 | IMPDH1 | 18 | 1 | 5.01285625974767 | -1.05218426805903 |
| 5556 | HILPDA | 16 | 1 | -4.48224566896327 | 2.25174106734823 |
| 5557 | METTL2B | 1 | 1 | 3.45447231809728 | 2.61549438613485 |
| 5558 | AC090114.2 | 1 | 1 | 3.16718579809301 | 1.52333201545309 |
| 5559 | FAM71F2 | 1 | 1 | 1.22733832876317 | 1.77597870009969 |
| 5560 | FAM71F1 | 6 | 1 | 1.44305814783208 | -2.75753842216898 |

| 5561 | CALU | 4 | 1 | -3.56034849603541 | -0.362684591831458 |
| --- | --- | --- | --- | --- | --- |
| 5562 | CCDC136 | 12 | 1 | 2.55339957754247 | 1.99602951186727 |
| 5563 | FLNC | 7 | 1 | -2.75314520318873 | -3.94038592201639 |
| 5564 | KCP | 7 | 1 | -2.88189195115931 | -3.52818737846781 |
| 5565 | ATP6V1F | 6 | 1 | 0.496052756711171 | -1.42382268530298 |
| 5566 | TNPO3 | 3 | 1 | -2.58652709443934 | 0.668240801273095 |
| 5567 | TSPAN33 | 4 | 1 | -4.55058215577967 | -1.16286037784983 |
| 5568 | SMO | 4 | 1 | -4.0693578573025 | -1.04716044587065 |
| 5569 | AHCYL2 | 12 | 1 | 2.40941406767003 | 2.35548652785848 |
| 5570 | STRIP2 | 17 | 1 | 1.94857288877599 | 0.0829183616025327 |
| 5571 | SMKR1 | 6 | 1 | 1.90134812872045 | -1.48739920479227 |
| 5572 | AC078846.1 | 6 | 1 | 2.47460557500951 | -1.36363033873011 |
| 5573 | NRF1 | 8 | 1 | -2.48166631181605 | 2.07515062469076 |
| 5574 | UBE2H | 2 | 1 | 0.785966530247853 | 1.68683470862936 |
| 5575 | ZC3HC1 | 3 | 1 | -2.45597313363917 | -0.76003147584368 |
| 5576 | KLHDC10 | 1 | 1 | 4.66245771925084 | 0.9895206846578 |
| 5577 | TMEM209 | 16 | 1 | -3.48842476327784 | 1.83065070289205 |
| 5578 | CEP41 | 9 | 1 | 0.650556221409963 | 3.08588351386617 |
| 5579 | MEST | 18 | 1 | 6.23232485334508 | -2.27492295128275 |
| 5580 | COPG2 | 18 | 1 | 5.58529163877599 | -0.762883930625212 |
| 5581 | AC007938.3 | 18 | 1 | 5.60634733717077 | -0.787145479740393 |
| 5582 | AC016831.7 | 12 | 1 | 2.9863176492893 | 2.32020701545309 |
| 5583 | AC016831.1 | 18 | 1 | 6.20404936353795 | -2.34419189316202 |
| 5584 | AC016831.5 | 6 | 1 | 2.23995019476049 | -2.21631131988932 |
| 5585 | AC058791.1 | 6 | 1 | 2.21233440916173 | -2.19022737366129 |
| 5586 | MKLN1 | 8 | 1 | -1.54658125360377 | 1.60641087668966 |
| 5587 | AC008264.2 | 1 | 1 | 1.60550524274938 | 1.16648687499593 |
| 5588 | PODXL | 14 | 1 | -6.75747416932948 | -0.483147128643286 |
| 5589 | PLXNA4 | 1 | 1 | 3.92604185621373 | 1.38852681296896 |
| 5590 | CHCHD3 | 3 | 1 | -3.09997199495204 | -0.75869057995249 |
| 5591 | EXOC4 | 9 | 1 | 1.13491798917882 | 2.95649088996481 |
| 5592 | SLC35B4 | 9 | 1 | 0.891641631528066 | 2.37484516280721 |
| 5593 | AKR1B1 | 3 | 1 | -3.59478376825221 | 0.994604841647851 |
| 5594 | BPGM | 6 | 1 | 2.08382822553747 | -1.21556432467867 |
| 5595 | CALD1 | 11 | 1 | -3.87051866968043 | -0.925309348078263 |
| 5596 | AGBL3 | 2 | 1 | 1.02182473222844 | 1.32266224998068 |
| 5597 | CYREN | 4 | 1 | -4.19700168092616 | -0.129418834270728 |
| 5598 | CNOT4 | 12 | 1 | 2.04361917059057 | 2.6624156393392 |
| 5599 | NUP205 | 16 | 1 | -3.41761373002894 | 1.77131165641378 |
| 5600 | STMP1 | 7 | 1 | -2.43047450502284 | -3.07625494820048 |
| 5601 | SLC13A4 | 7 | 1 | -3.39360950906642 | -2.94321666580607 |
| 5602 | AC091736.1 | 7 | 1 | -2.39329217393763 | -3.10912261825968 |
| 5603 | MTPN | 7 | 1 | -3.37426422555812 | -3.19643126350809 |
| 5604 | PTN | 8 | 1 | -2.23248611410029 | 1.32955373900961 |
| 5605 | AC078842.2 | 12 | 1 | 2.01329781095617 | 2.48001326697897 |
| 5606 | DGKI | 15 | 1 | 5.14727236311071 | -0.363596304477942 |
| 5607 | CREB3L2 | 14 | 1 | -4.97799609620936 | 0.147492186008203 |
| 5608 | TRIM24 | 8 | 1 | -1.70319722612269 | 1.64298786300253 |
| 5609 | KIAA1549 | 1 | 1 | 3.92406345884435 | 2.04963339942526 |
| 5610 | ZC3HAV1 | 19 | 1 | -4.77791808565028 | 1.06887997764181 |
| 5611 | TTC26 | 3 | 1 | -3.03482292611964 | 0.527488724170434 |
| 5612 | UBN2 | 12 | 1 | 2.18169929067724 | 3.03348602431844 |
| 5613 | FMC1 | 6 | 1 | 0.376611009045766 | -2.30321000439097 |
| 5614 | LUC7L2 | 2 | 1 | 0.565444066926167 | 0.843059913097131 |
| 5615 | AC083880.1 | 3 | 1 | -3.08902524431117 | -0.22996298891474 |
| 5616 | HIPK2 | 9 | 1 | 0.87959004919164 | 2.82244671958517 |
| 5617 | KDM7A | 2 | 1 | 0.575787439747975 | 2.05276765006613 |
| 5618 | SLC37A3 | 9 | 1 | 1.27101649324529 | 2.56378831046652 |
| 5619 | MKRN1 | 1 | 1 | 3.94147469083898 | 1.67023433822225 |
| 5620 | DENND2A | 2 | 1 | 0.586829200192616 | 2.11721529143881 |
| 5621 | ADCK2 | 10 | 1 | 4.69247080366246 | -1.24766157490183 |
| 5622 | NDUFB2 | 6 | 1 | 0.173077240391896 | -2.2318259320872 |
| 5623 | BRAF | 9 | 1 | 1.35119153539769 | 2.88012303489279 |
| 5624 | MRPS33 | 3 | 1 | -2.20998667199977 | -1.09374973100592 |
| 5625 | TMEM178B | 1 | 1 | 3.34156800787084 | 2.30402101653646 |
| 5626 | AC005692.1 | 12 | 1 | 1.81316281835668 | 2.66244067329 |
| 5627 | AC073878.1 | 12 | 1 | 2.2349896578037 | 1.70633019584249 |

| 5628 | AGK | 2 | 1 | 0.450765505238698 | 1.08800615447592 |
| --- | --- | --- | --- | --- | --- |
| 5629 | KIAA1147 | 2 | 1 | -1.08747397382624 | 2.1397880472524 |
| 5630 | SSBP1 | 3 | 1 | -2.44126914461024 | -1.10518290025164 |
| 5631 | TAS2R4 | 12 | 1 | 2.83886887113683 | 2.50518836158346 |
| 5632 | CLEC5A | 2 | 1 | 0.167029931946919 | 1.43913902419637 |
| 5633 | EPHB6 | 10 | 1 | 4.76145960371129 | -1.41650246006418 |
| 5634 | GSTK1 | 14 | 1 | -5.42365287263758 | -0.761554955662978 |
| 5635 | CASP2 | 9 | 1 | -0.323335632876231 | 2.92224706786703 |
| 5636 | FAM131B | 1 | 1 | 2.91646410505407 | 0.912386790691125 |
| 5637 | AC093673.2 | 13 | 1 | -1.34450505693324 | -2.74435780865122 |
| 5638 | AC093673.1 | 10 | 1 | 4.39832235853307 | -1.6780875764506 |
| 5639 | ZYX | 7 | 1 | -2.09833036382563 | -2.48384462219645 |
| 5640 | TCAF1 | 1 | 1 | 3.39965344946019 | 1.81965221541952 |
| 5641 | TPK1 | 5 | 1 | 4.25232197324865 | -0.349177583278906 |
| 5642 | CNTNAP2 | 10 | 1 | 4.77499653379552 | -0.5440506480353 |
| 5643 | C7orf33 | 7 | 1 | -2.13833270986445 | -3.5663293443339 |
| 5644 | CUL1 | 1 | 1 | 3.07029034177892 | 0.610137597499597 |
| 5645 | EZH2 | 16 | 1 | -4.2900040002621 | 2.45246972220968 |
| 5646 | GHET1 | 15 | 1 | 4.48735834638707 | 0.353434935985315 |
| 5647 | PDIA4 | 4 | 1 | -3.76923249681361 | 0.397488967357385 |
| 5648 | ZNF786 | 12 | 1 | 1.96393133680456 | 2.46780385154318 |
| 5649 | ZNF425 | 2 | 1 | 0.208185121222661 | 1.50618542808126 |
| 5650 | ZNF398 | 12 | 1 | 3.37906815092199 | 2.7233742632253 |
| 5651 | ZNF212 | 2 | 1 | -0.368680961624457 | 0.907259956775415 |
| 5652 | ZNF777 | 2 | 1 | 0.362428799077199 | 1.93932189124655 |
| 5653 | ZNF746 | 2 | 1 | 0.543430939122365 | 1.59436000960897 |
| 5654 | ZNF467 | 6 | 1 | 2.66140486280553 | -1.77446572405268 |
| 5655 | SSPO | 6 | 1 | 2.53371836225622 | -1.16744659763743 |
| 5656 | ZNF862 | 1 | 1 | 3.26663161794774 | 2.09598602431844 |
| 5657 | ATP6V0E2-AS1 | 15 | 1 | 4.80004622022741 | -0.193883284153235 |
| 5658 | ATP6V0E2 | 10 | 1 | 4.14052702467077 | -1.34236274582316 |
| 5659 | RARRES2 | 14 | 1 | -5.87019656618006 | -0.0150414548533083 |
| 5660 | REPIN1 | 3 | 1 | -2.4191007467068 | -1.08518050533701 |
| 5661 | ZNF775 | 13 | 1 | -0.829236135081126 | -0.57695184570719 |
| 5662 | TMEM176B | 7 | 1 | -2.14272092302211 | -3.60229359489847 |
| 5663 | TMEM176A | 7 | 1 | -2.12251304109461 | -3.61788831573893 |
| 5664 | KCNH2 | 5 | 1 | 3.79767014066808 | -0.226794704021704 |
| 5665 | NOS3 | 7 | 1 | -2.02772341688044 | -3.57424555641581 |
| 5666 | ABCB8 | 1 | 1 | 4.36116148511998 | 1.31126822608541 |
| 5667 | CDK5 | 10 | 1 | 3.36289383451574 | -1.8661152563708 |
| 5668 | SLC4A2 | 4 | 1 | -4.2960903497494 | -0.587498977007163 |
| 5669 | FASTK | 17 | 1 | 0.102632775708363 | -0.0451474033014895 |
| 5670 | TMUB1 | 6 | 1 | -0.229328321487381 | -1.53472410065104 |
| 5671 | AGAP3 | 5 | 1 | 3.05390717069738 | -0.229123517097724 |
| 5672 | ABCF2.1 | 3 | 1 | -1.99366222341426 | -0.522088213981879 |
| 5673 | SMARCD3 | 10 | 1 | 4.72201038877599 | -0.834085627617133 |
| 5674 | AC021097.1 | 1 | 1 | 2.81089879552953 | 1.82467474120687 |
| 5675 | NUB1 | 2 | 1 | 0.307704999848531 | 1.70182932990621 |
| 5676 | WDR86 | 18 | 1 | 5.51207424680822 | -1.7359905682223 |
| 5677 | RHEB | 13 | 1 | -0.549383327559306 | -2.02776120525767 |
| 5678 | PRKAG2 | 15 | 1 | 4.84451796095006 | 0.218846694408166 |
| 5679 | PRKAG2-AS1 | 14 | 1 | -6.33377955873378 | -0.983084253193629 |
| 5680 | GALNT11 | 5 | 1 | 3.88662458936803 | 0.239264861522424 |
| 5681 | KMT2C | 12 | 1 | 2.45548607389562 | 2.36475648063253 |
| 5682 | LINC01003 | 10 | 1 | 3.48342920820348 | -1.756768747391 |
| 5683 | XRCC2 | 19 | 1 | -4.82613490541346 | 2.08695544379781 |
| 5684 | ACTR3B | 10 | 1 | 4.55447746793859 | -0.701642229379904 |
| 5685 | DPP6 | 15 | 1 | 4.62292219678991 | 0.0625449575765012 |
| 5686 | PAXIP1 | 9 | 1 | 0.00463707725636764 | 3.09273351806234 |
| 5687 | PAXIP1-AS1 | 17 | 1 | 1.85851503889196 | 0.419445411144006 |
| 5688 | AC144652.1 | 5 | 1 | 4.04912544767492 | -0.0853538952486635 |
| 5689 | INSIG1 | 6 | 1 | 2.50315667669408 | -1.58461914879252 |
| 5690 | AC008060.1 | 14 | 1 | -6.92519305665858 | -0.855815819622767 |
| 5691 | EN2 | 14 | 1 | -6.54944632966883 | -1.10320305746962 |
| 5692 | CNPY1 | 14 | 1 | -6.55389092882045 | -1.09979687553812 |
| 5693 | RBM33 | 9 | 1 | 0.749489739342854 | 2.8026584543569 |
| 5694 | LINC01006 | 6 | 1 | 0.944811716481374 | -0.713045238675368 |

| 5695 | RNF32 | 3 | 1 | -3.16367267091639 | 0.498896972118127 |
| --- | --- | --- | --- | --- | --- |
| 5696 | LMBR1 | 1 | 1 | 3.37198640386693 | 2.01920475143026 |
| 5697 | NOM1 | 2 | 1 | -1.07463596780665 | 2.03548468726705 |
| 5698 | MNX1 | 4 | 1 | -5.38677237947352 | -2.05328772884775 |
| 5699 | UBE3C | 9 | 1 | 1.03912438432805 | 2.91183294433187 |
| 5700 | DNAJB6 | 10 | 1 | 3.73574544469945 | -1.17385648113657 |
| 5701 | AC006372.3 | 18 | 1 | 5.48780037443273 | -0.393329902710211 |
| 5702 | PTPRN2 | 1 | 1 | 3.53168989698522 | 2.03424109595846 |
| 5703 | AC005481.1 | 9 | 1 | 0.467978134556935 | 2.57476367133688 |
| 5704 | NCAPG2 | 16 | 1 | -4.75124143083461 | 2.3464747824056 |
| 5705 | ESYT2 | 12 | 1 | 2.93653584997289 | 2.31291593688558 |
| 5706 | WDR60 | 2 | 1 | -0.556874707535579 | 1.2606536306722 |
| 5707 | VIPR2 | 7 | 1 | -3.76576589067347 | -2.98250995499064 |
| 5708 | PLCXD1 | 15 | 1 | 4.61807180921666 | 0.407523528514612 |
| 5709 | GTPBP6 | 3 | 1 | -2.49006603677638 | -0.800023629488242 |
| 5710 | LINC00685 | 5 | 1 | 2.79286481420629 | -0.695853664697898 |
| 5711 | PPP2R3B | 16 | 1 | -3.83977054078944 | 2.44498719352316 |
| 5712 | AL672277.1 | 11 | 1 | -4.00833605249293 | -1.61496321779658 |
| 5713 | IL3RA | 1 | 1 | 2.57396365682714 | 0.895062581477868 |
| 5714 | SLC25A6 | 21 | 1 | -0.963060125902965 | -4.40874038559366 |
| 5715 | ASMTL | 13 | 1 | -1.06563870628245 | -1.44659046751429 |
| 5716 | AKAP17A | 1 | 1 | 1.32584692518346 | 1.3664783396108 |
| 5717 | DHRSX | 11 | 1 | -3.94885514696009 | -1.09114833098818 |
| 5718 | ZBED1 | 2 | 1 | 0.738068297310994 | 1.82766999381613 |
| 5719 | CD99 | 14 | 1 | -5.62029384096034 | -0.714470639051688 |
| 5720 | GYG2 | 1 | 1 | 2.50105883161657 | 1.09861971515249 |
| 5721 | ARSD | 1 | 1 | 1.79483653585546 | 0.98442830222677 |
| 5722 | ARSE | 7 | 1 | -2.94328449685939 | -3.92880307060648 |
| 5723 | MXRA5 | 14 | 1 | -4.90382407625087 | 0.0991752304417967 |
| 5724 | PRKX | 1 | 1 | 3.82680393735997 | 1.80911244529318 |
| 5725 | BX890604.1 | 1 | 1 | 4.01191212217443 | 1.81495346206259 |
| 5726 | NLGN4X | 15 | 1 | 5.47167660276525 | 0.821836129604089 |
| 5727 | PUDP | 5 | 1 | 3.97149492780797 | -0.447092815460456 |
| 5728 | PNPLA4 | 11 | 1 | -3.43210504968531 | -1.41453795057703 |
| 5729 | ANOS1 | 4 | 1 | -5.10345624406703 | -1.31094764095713 |
| 5730 | FAM9B | 17 | 1 | 0.370707765027211 | -0.0756861410753847 |
| 5731 | TBL1X | 11 | 1 | -4.10940121133692 | -1.41471396309305 |
| 5732 | GPR143 | 11 | 1 | -3.94751141984828 | -2.24882136207987 |
| 5733 | SHROOM2 | 17 | 1 | 0.294686630173848 | 0.281604067264306 |
| 5734 | WWC3 | 3 | 1 | -2.68725560624964 | 0.873798862872827 |
| 5735 | CLCN4 | 1 | 1 | 3.69475199262731 | 0.677644506870019 |
| 5736 | MID1 | 14 | 1 | -5.05896495302088 | 0.340147034106958 |
| 5737 | AC073529.1 | 16 | 1 | -4.38317130525477 | 2.61114730018209 |
| 5738 | HCCS | 3 | 1 | -2.84864185769923 | -0.937336496235621 |
| 5739 | ARHGAP6 | 7 | 1 | -3.45085071046717 | -3.56164418083597 |
| 5740 | MSL3 | 13 | 1 | -0.434886470631434 | -2.32664237839152 |
| 5741 | PRPS2 | 19 | 1 | -5.34342931230433 | 1.52241839545797 |
| 5742 | TMSB4X | 7 | 1 | -2.50363443811305 | -3.64543972832133 |
| 5743 | RAB9A | 3 | 1 | -2.77655099351771 | -1.28945068937708 |
| 5744 | TRAPPC2 | 1 | 1 | 4.48736740629308 | 0.861926213680017 |
| 5745 | OFD1 | 3 | 1 | -1.94578240831263 | -0.0224678359644533 |
| 5746 | GPM6B | 14 | 1 | -6.75604413469203 | -0.749700754107726 |
| 5747 | AC003035.1 | 8 | 1 | -1.2450604291714 | 0.907069460330713 |
| 5748 | GLRA2 | 15 | 1 | 5.5675423292362 | -0.0916256747858645 |
| 5749 | FANCB | 19 | 1 | -4.88031838853724 | 1.97583307403158 |
| 5750 | MOSPD2 | 12 | 1 | 2.43178273717992 | 2.5083571829183 |
| 5751 | PIR | 4 | 1 | -4.14419673402674 | -0.850556056173337 |
| 5752 | CA5B | 12 | 1 | 2.18981815855138 | 2.93032182830404 |
| 5753 | ZRSR2 | 5 | 1 | 4.40623022596471 | -0.379788621486914 |
| 5754 | AP1S2 | 10 | 1 | 4.00718428175084 | -1.42706112486292 |
| 5755 | CTPS2 | 7 | 1 | -3.12803171594508 | -2.37858603817393 |
| 5756 | SYAP1 | 3 | 1 | -2.42799733598597 | -0.37318466526438 |
| 5757 | TXLNG | 1 | 1 | 4.06878115217321 | 1.0211653866155 |
| 5758 | RBBP7 | 3 | 1 | -1.84209822137721 | 0.280945912776697 |
| 5759 | REPS2 | 18 | 1 | 5.63254095594518 | -1.68022535664011 |
| 5760 | NHS | 7 | 1 | -3.10373328645594 | -2.64360986572672 |
| 5761 | SCML1 | 14 | 1 | -6.14332316835292 | -0.487834706129325 |

| 5762 | RAI2 | 15 | 1 | 5.27667118589513 | 0.0734098710400938 |
| --- | --- | --- | --- | --- | --- |
| 5763 | LINC01456 | 10 | 1 | 3.81636883298986 | -1.01298231554199 |
| 5764 | SCML2 | 16 | 1 | -3.28456018884547 | 2.21664370673727 |
| 5765 | CDKL5 | 1 | 1 | 3.92076708356969 | 1.96090735572408 |
| 5766 | PHKA2 | 8 | 1 | -2.12874268014796 | 1.56781400817465 |
| 5767 | PDHA1 | 13 | 1 | -0.714481964901759 | -1.22897581678797 |
| 5768 | SH3KBP1 | 18 | 1 | 5.5773956922733 | -1.93537627083231 |
| 5769 | BCLAF3 | 12 | 1 | 2.38127066175573 | 1.93800844329427 |
| 5770 | MAP7D2 | 5 | 1 | 4.23634983579747 | -0.0136940918581606 |
| 5771 | EIF1AX | 17 | 1 | 1.24581386129491 | 0.486397997318017 |
| 5772 | RPS6KA3 | 12 | 1 | 2.36020494978063 | 2.85921610969137 |
| 5773 | CNKSR2 | 12 | 1 | 1.8458659795963 | 2.45405305999349 |
| 5774 | KLHL34 | 15 | 1 | 4.85156990568273 | 0.0761081137044309 |
| 5775 | MBTPS2 | 2 | 1 | -0.978376016215159 | 1.82989539283346 |
| 5776 | SMS | 4 | 1 | -4.81713937242396 | -1.02996593636442 |
| 5777 | PHEX | 18 | 1 | 5.96380116026037 | -1.76676939350535 |
| 5778 | PHEX-AS1 | 6 | 1 | 2.00272823850744 | -2.45582304817606 |
| 5779 | PTCHD1 | 18 | 1 | 5.69684959928624 | -1.20978654843737 |
| 5780 | PRDX4 | 4 | 1 | -3.88498233278163 | -0.708906679572356 |
| 5781 | ACOT9 | 14 | 1 | -5.59610198457606 | 0.112569467006433 |
| 5782 | SAT1 | 10 | 1 | 3.3962342886173 | -1.33080916029383 |
| 5783 | APOO | 6 | 1 | 2.05174399892919 | -1.71095941883494 |
| 5784 | KLHL15 | 1 | 1 | 3.58681537191503 | 2.65902318137716 |
| 5785 | EIF2S3 | 8 | 1 | -1.37810955961116 | 0.979184166370141 |
| 5786 | ZFX | 9 | 1 | -0.356898829535319 | 2.86758698600363 |
| 5787 | PDK3 | 8 | 1 | -1.26183556039698 | 1.74604620116781 |
| 5788 | PCYT1B | 1 | 1 | 4.83982874433629 | 0.929782883105981 |
| 5789 | POLA1 | 19 | 1 | -5.14878819902308 | 1.5866931833608 |
| 5790 | ARX | 18 | 1 | 6.26434732954137 | -2.55049060207773 |
| 5791 | IL1RAPL1 | 18 | 1 | 5.59403349439733 | -0.697957291425955 |
| 5792 | GK | 5 | 1 | 3.61354352514379 | 0.17429281848501 |
| 5793 | TAB3 | 12 | 1 | 1.76502872984044 | 2.61461438315939 |
| 5794 | DMD | 15 | 1 | 5.34112097303502 | 0.737539545474756 |
| 5795 | TMEM47 | 14 | 1 | -6.35399983842738 | -0.614984168829215 |
| 5796 | CFAP47 | 15 | 1 | 6.03420759718053 | 0.774310962138879 |
| 5797 | LANCL3 | 18 | 1 | 6.21824289838903 | -2.42957101684977 |
| 5798 | DYNLT3 | 10 | 1 | 3.41545464079015 | -1.43078730923106 |
| 5799 | SRPX | 7 | 1 | -3.43530224283107 | -3.46101246696879 |
| 5800 | RPGR | 2 | 1 | -1.0787412973202 | 1.35386623519491 |
| 5801 | TSPAN7 | 10 | 1 | 4.46995712797277 | -1.61732612472941 |
| 5802 | MID1IP1 | 5 | 1 | 3.91740967313878 | -0.601560278953803 |
| 5803 | BCOR | 2 | 1 | -0.269642926946952 | 1.3004280485494 |
| 5804 | ATP6AP2 | 10 | 1 | 3.27266480009191 | -1.57520936352183 |
| 5805 | MED14 | 2 | 1 | -1.04849772174723 | 1.99082626479696 |
| 5806 | USP9X | 9 | 1 | 1.61657835523717 | 2.89049591201376 |
| 5807 | DDX3X | 8 | 1 | -1.2742545457638 | 1.78961195128988 |
| 5808 | CASK | 1 | 1 | 4.1225206999027 | 2.15253438132833 |
| 5809 | GPR82 | 6 | 1 | 2.58455516378515 | -1.67138503414561 |
| 5810 | MAOA | 15 | 1 | 5.96681238691442 | 0.793007389484155 |
| 5811 | MAOB | 10 | 1 | 4.53426338712804 | -0.529994919122946 |
| 5812 | FUNDC1 | 3 | 1 | -2.42847989519007 | -0.922740309657347 |
| 5813 | KDM6A | 9 | 1 | 1.49450792352788 | 2.95436872619222 |
| 5814 | LINC01186 | 4 | 1 | -4.50667212922938 | -0.0972126565592409 |
| 5815 | KRBOX4 | 2 | 1 | 0.315890803738759 | 0.850521818576562 |
| 5816 | ZNF674-AS1 | 4 | 1 | -3.26075576265223 | 0.141184226451623 |
| 5817 | CHST7 | 7 | 1 | -2.40967486818202 | -2.33422158581187 |
| 5818 | SLC9A7 | 15 | 1 | 5.25833298246495 | 1.17622985023092 |
| 5819 | RP2 | 3 | 1 | -2.65583895166285 | 1.0435122408254 |
| 5820 | JADE3 | 4 | 1 | -4.53339885194667 | -0.302604659618628 |
| 5821 | NDUFB11 | 6 | 1 | 0.296355977460072 | -2.34602986198832 |
| 5822 | RBM10 | 2 | 1 | -0.663817838028743 | 1.24504341262411 |
| 5823 | UBA1 | 2 | 1 | 1.0828957704746 | 0.99148704188894 |
| 5824 | CDK16 | 5 | 1 | 3.50557495634191 | 0.2105078853948 |
| 5825 | USP11 | 1 | 1 | 4.11348463575475 | 1.71333398002218 |
| 5826 | ZNF41 | 1 | 1 | 3.19478203336828 | 2.17822732108663 |
| 5827 | ARAF | 8 | 1 | -1.42034135778315 | 0.133296624599206 |
| 5828 | SYN1 | 15 | 1 | 4.5102274564945 | 0.251023904262292 |

| 5829 | TIMP1 | 14 | 1 | -5.78260205705531 | -0.707388415159476 |
| --- | --- | --- | --- | --- | --- |
| 5830 | ELK1 | 10 | 1 | 3.24188901464574 | -1.47539673668314 |
| 5831 | UXT | 13 | 1 | -0.0646632911718626 | -2.47575841766764 |
| 5832 | UXT-AS1 | 2 | 1 | -0.492509395078494 | 1.01863743442129 |
| 5833 | ZNF182 | 9 | 1 | 0.880826368733571 | 2.96348919051717 |
| 5834 | ZNF630 | 1 | 1 | 4.17963053266637 | 0.664468542514551 |
| 5835 | SLC38A5 | 7 | 1 | -3.29115651567347 | -3.80505190712382 |
| 5836 | PORCN | 10 | 1 | 4.50987936536901 | -0.706222458900702 |
| 5837 | EBP | 6 | 1 | 2.30568028967016 | -1.59319148880411 |
| 5838 | TBC1D25 | 10 | 1 | 4.09752489606969 | -1.46686510664393 |
| 5839 | RBM3 | 11 | 1 | -3.69754599054225 | -1.66471181732584 |
| 5840 | WDR13 | 17 | 1 | 1.32546676675908 | 0.0239223994595884 |
| 5841 | SUV39H1 | 16 | 1 | -4.46338151414759 | 2.42136563438009 |
| 5842 | HDAC6 | 1 | 1 | 3.76035954038732 | 1.89475692885946 |
| 5843 | PCSK1N | 10 | 1 | 3.87065483610265 | -1.59676437002589 |
| 5844 | TIMM17B | 6 | 1 | 1.27474905531041 | -1.75177554708887 |
| 5845 | PQBP1 | 13 | 1 | -1.22533606012232 | -1.46641735655237 |
| 5846 | SLC35A2 | 3 | 1 | -1.85233579595454 | -0.529911800445807 |
| 5847 | PIM2 | 5 | 1 | 3.80033852140539 | -0.319425626816046 |
| 5848 | OTUD5 | 2 | 1 | 0.117075368567632 | 1.5277344145162 |
| 5849 | GRIPAP1 | 12 | 1 | 3.13425447027318 | 2.54141511100363 |
| 5850 | CCDC120 | 5 | 1 | 3.82542969267003 | -0.0865519486086489 |
| 5851 | PRAF2 | 5 | 1 | 2.35768868009679 | -0.694878905238402 |
| 5852 | WDR45 | 13 | 1 | -1.55633269269831 | -1.93363915306498 |
| 5853 | GPKOW | 1 | 1 | 4.06903053800695 | 1.42584170478414 |
| 5854 | SYP | 5 | 1 | 4.3964727071964 | 0.507010475574243 |
| 5855 | CCDC22 | 8 | 1 | -0.695812568262889 | -0.292855843128455 |
| 5856 | PPP1R3F | 5 | 1 | 2.4626839307987 | -0.253490134300482 |
| 5857 | AC232271.1 | 9 | 1 | -0.515049681261851 | 2.93210209983419 |
| 5858 | USP27X | 1 | 1 | 4.67628790418737 | 0.694049850879419 |
| 5859 | CLCN5 | 12 | 1 | 1.82920552770726 | 2.58983267921041 |
| 5860 | DGKK | 15 | 1 | 5.43555904905431 | 0.553357974467981 |
| 5861 | SHROOM4 | 18 | 1 | 6.09667469541662 | -2.80595777851511 |
| 5862 | NUDT10 | 17 | 1 | -0.246379478097631 | -0.153931959690344 |
| 5863 | CENPVL3 | 3 | 1 | -2.1617582889355 | -0.0403256855623843 |
| 5864 | GSPT2 | 15 | 1 | 4.544698014661 | 0.185385004459131 |
| 5865 | MAGED1 | 15 | 1 | 4.77926851789586 | -0.403111978592169 |
| 5866 | AC239585.2 | 18 | 1 | 5.22267033140294 | -1.04406352263857 |
| 5867 | GPR173 | 12 | 1 | 1.79865004102819 | 2.2807485021932 |
| 5868 | TSPYL2 | 12 | 1 | 2.06976725141637 | 2.96077646392416 |
| 5869 | KANTR | 1 | 1 | 3.27993465940587 | 2.17672886031698 |
| 5870 | KDM5C | 12 | 1 | 1.98915339986913 | 2.45274819511007 |
| 5871 | IQSEC2 | 15 | 1 | 5.49924398939245 | 0.155027762828576 |
| 5872 | SMC1A | 16 | 1 | -3.42219780404933 | 2.0963219560964 |
| 5873 | RIBC1 | 13 | 1 | -1.13111548145182 | -2.74967811924387 |
| 5874 | HSD17B10 | 3 | 1 | -1.83314512689479 | -1.17537675720621 |
| 5875 | HUWE1 | 9 | 1 | 1.5045870689594 | 2.77013792174886 |
| 5876 | PHF8 | 8 | 1 | -1.25621257741816 | 1.58429755347799 |
| 5877 | WNK3 | 1 | 1 | 4.14750076810949 | 1.90612448829244 |
| 5878 | TSR2 | 3 | 1 | -3.11189626176722 | 0.32019330638479 |
| 5879 | FGD1 | 7 | 1 | -3.13687704522975 | -3.20233832222391 |
| 5880 | GNL3L | 2 | 1 | -0.817019269064738 | 2.09144224303793 |
| 5881 | MAGED2 | 8 | 1 | -0.324824564353301 | -0.441237076343787 |
| 5882 | TRO | 12 | 1 | 2.41677786390416 | 1.81163753646444 |
| 5883 | APEX2 | 3 | 1 | -2.29678724725611 | -0.304372056545508 |
| 5884 | FAM104B | 3 | 1 | -3.13597248514064 | -0.344668372692359 |
| 5885 | MAGEH1 | 10 | 1 | 4.89381243269079 | -0.78921077323128 |
| 5886 | USP51 | 12 | 1 | 2.83230735342138 | 2.5772720732076 |
| 5887 | RRAGB | 1 | 1 | 3.97335030119054 | 1.53252185958456 |
| 5888 | AL445472.1 | 7 | 1 | -2.85516737420924 | -2.63044355732371 |
| 5889 | UBQLN2 | 1 | 1 | 3.94989086668126 | 2.27563728469442 |
| 5890 | NBDY | 6 | 1 | 2.2034909872257 | -1.6166747651713 |
| 5891 | SPIN3 | 1 | 1 | 3.79837585966222 | 2.4907242216451 |
| 5892 | SPIN2B | 10 | 1 | 3.72514702360265 | -1.15158520561625 |
| 5893 | SPIN2A | 1 | 1 | 4.43522383253209 | 1.03066505569051 |
| 5894 | SPIN4 | 9 | 1 | -0.244908732556476 | 3.14065159934591 |
| 5895 | ARHGEF9 | 1 | 1 | 3.69421435873143 | 1.11549867766927 |

| 5896 | AMER1 | 9 | 1 | 1.13068391363256 | 2.56001128333639 |
| --- | --- | --- | --- | --- | --- |
| 5897 | ZC4H2 | 15 | 1 | 5.02694679777257 | -0.0969277463572146 |
| 5898 | ZC3H12B | 1 | 1 | 3.70992113630407 | 2.48424948829244 |
| 5899 | LAS1L | 3 | 1 | -2.92579124887355 | 0.0624665178639768 |
| 5900 | MSN | 11 | 1 | -3.38620613534815 | -2.32356582504679 |
| 5901 | HEPH | 7 | 1 | -3.57480572183497 | -2.61953495365549 |
| 5902 | EDA2R | 3 | 1 | -3.20133374650843 | -0.283384009422553 |
| 5903 | AR | 5 | 1 | 4.10262705366246 | -0.617454066099417 |
| 5904 | OPHN1 | 5 | 1 | 3.53774239103429 | -0.0660306654589297 |
| 5905 | YIPF6 | 6 | 1 | 0.160008564397023 | -1.16807006222178 |
| 5906 | STARD8 | 14 | 1 | -6.11231873948939 | 0.152018085895288 |
| 5907 | EFNB1 | 4 | 1 | -5.01907847841151 | -1.34209705811907 |
| 5908 | PJA1 | 5 | 1 | 4.34676624815099 | -0.781168519677413 |
| 5909 | FAM155B | 1 | 1 | 1.16170419733159 | 1.84593023437093 |
| 5910 | EDA | 7 | 1 | -2.63892100770838 | -3.8442081056254 |
| 5911 | OTUD6A | 14 | 1 | -6.72379134614833 | -0.640175386490119 |
| 5912 | IGBP1 | 17 | 1 | 0.354444756909535 | 0.379924313007104 |
| 5913 | PDZD11 | 6 | 1 | 1.80026127378576 | -1.71633933407236 |
| 5914 | KIF4A | 16 | 1 | -4.45154641588099 | 2.81933154243063 |
| 5915 | GDPD2 | 4 | 1 | -4.38713334520228 | -0.57085232359339 |
| 5916 | DLG3 | 9 | 1 | 0.145563497944997 | 2.70240272658895 |
| 5917 | SLC7A3 | 19 | 1 | -5.22454474885829 | 1.6744526781423 |
| 5918 | FOXO4 | 1 | 1 | 2.54722262899511 | 1.4541727461202 |
| 5919 | MED12 | 8 | 1 | -1.26587592084773 | 1.24390091079305 |
| 5920 | NLGN3 | 5 | 1 | 4.24407793562047 | -0.154420896591437 |
| 5921 | ZMYM3 | 1 | 1 | 3.21054507772558 | 2.02485098022054 |
| 5922 | NONO | 8 | 1 | -2.07689987142451 | 0.874381200252282 |
| 5923 | TAF1 | 12 | 1 | 2.23966671506994 | 3.05902327674459 |
| 5924 | OGT | 12 | 1 | 2.15149618665807 | 2.70364059585165 |
| 5925 | NHSL2 | 14 | 1 | -6.65204547365077 | -0.750040142955077 |
| 5926 | RTL5 | 2 | 1 | 0.372289255067037 | 2.03069819587301 |
| 5927 | PIN4 | 6 | 1 | 0.126655533715413 | -2.3443176589625 |
| 5928 | ERCC6L | 16 | 1 | -4.71338771303065 | 2.33257331031393 |
| 5929 | RPS4X | 21 | 1 | -0.654459312752559 | -4.17821966034342 |
| 5930 | CITED1 | 7 | 1 | -2.49656270463832 | -3.61180363518168 |
| 5931 | HDAC8 | 2 | 1 | -0.68230746705897 | 1.40827383178304 |
| 5932 | NAP1L2 | 15 | 1 | 4.82100416700475 | 0.388905421672571 |
| 5933 | CHIC1 | 9 | 1 | 2.01779152433507 | 3.12827338355612 |
| 5934 | JPX | 9 | 1 | 1.96788884679906 | 2.84576429503988 |
| 5935 | FTX | 12 | 1 | 2.49861909429662 | 2.02224196570944 |
| 5936 | AL353804.1 | 12 | 1 | 1.8277208952152 | 2.61951030868124 |
| 5937 | SLC16A2 | 7 | 1 | -2.72994326074488 | -3.57052932602335 |
| 5938 | RLIM | 12 | 1 | 2.11248399297826 | 3.08382214683126 |
| 5939 | NEXMIF | 1 | 1 | 4.68530870954625 | 0.694025770602929 |
| 5940 | ABCB7 | 11 | 1 | -3.09546802957423 | -1.7435639939921 |
| 5941 | UPRT | 13 | 1 | -0.912557229594066 | -0.511396779836905 |
| 5942 | ZDHHC15 | 1 | 1 | 3.63946320097081 | 2.47594107764791 |
| 5943 | PBDC1 | 13 | 1 | -0.743183657721355 | -0.848042613984835 |
| 5944 | MAGEE1 | 1 | 1 | 4.64685751478307 | 0.745580212054956 |
| 5945 | AC233296.1 | 5 | 1 | 3.98481251279943 | -0.582937075914633 |
| 5946 | ATRX | 12 | 1 | 2.18428303281896 | 3.01374496596884 |
| 5947 | MAGT1 | 4 | 1 | -4.4150335641659 | -0.661195158185256 |
| 5948 | COX7B | 6 | 1 | 0.304277851983235 | -2.31735823971201 |
| 5949 | ATP7A | 9 | 1 | 1.62273658792608 | 2.69226612227987 |
| 5950 | PGK1 | 13 | 1 | -1.35986457784541 | -1.35873632055689 |
| 5951 | TAF9B | 8 | 1 | -0.76227436979182 | 0.199576631961572 |
| 5952 | CYSLTR1 | 15 | 1 | 5.38800884763829 | 0.830188647685754 |
| 5953 | LPAR4 | 4 | 1 | -4.51218532045253 | -0.0184554300921084 |
| 5954 | ITM2A | 11 | 1 | -4.38803170640834 | -2.03143940788676 |
| 5955 | BRWD3 | 12 | 1 | 2.18354703466527 | 2.68574608939718 |
| 5956 | HMGN5 | 4 | 1 | -4.0532605500973 | -0.264156504692328 |
| 5957 | SH3BGRL | 11 | 1 | -3.22122047860987 | -1.87937836271693 |
| 5958 | POU3F4 | 14 | 1 | -6.52897666414149 | -1.14688712281157 |
| 5959 | RPS6KA6 | 1 | 1 | 3.25047637502782 | 1.80003728049825 |
| 5960 | HDX | 9 | 1 | 1.7213280348026 | 2.87863172668051 |
| 5961 | APOOL | 2 | 1 | 0.245629891558812 | 2.10426081794332 |
| 5962 | SATL1 | 10 | 1 | 4.94341970960729 | -0.608952089370978 |

| 5963 | ZNF711 | 1 | 1 | 4.00698448698156 | 1.79624284881185 |
| --- | --- | --- | --- | --- | --- |
| 5964 | CHM | 12 | 1 | 2.6477265505039 | 2.15988625663351 |
| 5965 | DACH2 | 15 | 1 | 5.20899654905431 | 0.349602595744836 |
| 5966 | KLHL4 | 5 | 1 | 3.93560673277013 | -0.740744396271002 |
| 5967 | PABPC5 | 2 | 1 | 1.07852567712896 | 1.03211845534872 |
| 5968 | PCDH11X | 15 | 1 | 5.79560638944738 | 0.611459270892846 |
| 5969 | NAP1L3 | 15 | 1 | 4.79411913435094 | -0.174733384670508 |
| 5970 | FAM133A | 15 | 1 | 5.2093389181339 | 0.447519914088952 |
| 5971 | DIAPH2 | 15 | 1 | 5.64437986890905 | -0.135268493713629 |
| 5972 | RPA4 | 18 | 1 | 5.64796902219884 | -0.31203155142237 |
| 5973 | PCDH19 | 14 | 1 | -5.69532559831507 | -0.410639508785498 |
| 5974 | TSPAN6 | 11 | 1 | -3.86063049753077 | -1.49159751755167 |
| 5975 | CSTF2 | 8 | 1 | -1.36119268854029 | 0.0189658560140012 |
| 5976 | TRMT2B | 4 | 1 | -4.406069979266 | -0.706168591202986 |
| 5977 | TMEM35A | 17 | 1 | 1.29863955061071 | 0.457290545879114 |
| 5978 | CENPI | 16 | 1 | -4.55017827470668 | 3.15897835868429 |
| 5979 | DRP2 | 18 | 1 | 5.98645283262365 | -1.82678936344553 |
| 5980 | TIMM8A | 4 | 1 | -4.0651709886349 | 0.221982852397668 |
| 5981 | RPL36A | 21 | 1 | -0.933754906252696 | -4.4984513364451 |
| 5982 | GLA | 16 | 1 | -4.26920197923548 | 1.79331626075338 |
| 5983 | HNRNPH2 | 2 | 1 | 0.825906052991078 | 0.883601323543298 |
| 5984 | ARMCX4 | 1 | 1 | 4.06465460340612 | 2.024825231014 |
| 5985 | ARMCX1 | 5 | 1 | 2.4269218591892 | -0.148399933399451 |
| 5986 | ARMCX6 | 7 | 1 | -1.68600748498805 | -3.04201374870707 |
| 5987 | ARMCX3 | 5 | 1 | 3.37155272047155 | 0.143288031993616 |
| 5988 | ARMCX2 | 1 | 1 | 3.33427001516454 | 0.600852981982934 |
| 5989 | ZMAT1 | 2 | 1 | -0.0218152405775328 | 2.17144669669698 |
| 5990 | TCEAL2 | 17 | 1 | 1.64727140943639 | -0.296996697010291 |
| 5991 | BEX5 | 17 | 1 | 1.68639720956914 | -0.414454206051123 |
| 5992 | TMSB15A | 19 | 1 | -3.91864393670924 | 1.17203463691305 |
| 5993 | ARMCX5 | 1 | 1 | 3.72336007635228 | 2.43321289199423 |
| 5994 | GPRASP1 | 1 | 1 | 3.65120054761998 | 2.56476558822225 |
| 5995 | GPRASP2 | 15 | 1 | 5.08285261671178 | 0.257572308955896 |
| 5996 | LINC00630 | 9 | 1 | 0.269052102967427 | 2.8808039583547 |
| 5997 | BEX1 | 10 | 1 | 3.95241619627111 | -1.56580822092463 |
| 5998 | BEX4 | 10 | 1 | 3.25512362997167 | -1.75215248686243 |
| 5999 | TCEAL8 | 6 | 1 | -0.0187975468195219 | -1.20339216214587 |
| 6000 | TCEAL5 | 6 | 1 | 2.31729890386693 | -2.14980839115549 |
| 6001 | BEX2 | 10 | 1 | 3.55033327619664 | -1.52471129518915 |
| 6002 | TCEAL7 | 10 | 1 | 3.29379750768773 | -1.74395374876429 |
| 6003 | TCEAL9 | 11 | 1 | -3.42404316385157 | -1.40227799040247 |
| 6004 | BEX3 | 6 | 1 | 2.44527246038549 | -2.03406999927927 |
| 6005 | RAB40A | 12 | 1 | 2.31956912557714 | 2.29132141250204 |
| 6006 | TCEAL4 | 10 | 1 | 3.67537380735509 | -1.12161481303145 |
| 6007 | TCEAL3 | 10 | 1 | 3.80464579145543 | -1.40531496626307 |
| 6008 | TCEAL1 | 5 | 1 | 4.21662355939977 | -0.376802190365088 |
| 6009 | MORF4L2 | 17 | 1 | 0.458789244099782 | 0.601145163951623 |
| 6010 | MORF4L2-AS1 | 8 | 1 | -2.29567216356166 | 1.20694126266073 |
| 6011 | TMEM31 | 5 | 1 | 4.0263931898319 | -0.783281161607993 |
| 6012 | PLP1 | 6 | 1 | 2.1577031759464 | -2.15379284244944 |
| 6013 | RAB9B | 5 | 1 | 4.2045633939945 | -0.615788414301169 |
| 6014 | TMSB15B-AS1 | 5 | 1 | 4.0275557188236 | -0.359270318569434 |
| 6015 | TMSB15B | 1 | 1 | 3.31764890234105 | 1.06290532725881 |
| 6016 | TMSB15B.1 | 5 | 1 | 3.94283939878576 | 0.420180217204797 |
| 6017 | SLC25A53 | 9 | 1 | 1.65306057016485 | 2.99796880858968 |
| 6018 | ZCCHC18 | 15 | 1 | 5.28225494901769 | 0.383242384372461 |
| 6019 | FAM199X | 12 | 1 | 2.72806693594091 | 2.31946958678793 |
| 6020 | MUM1L1 | 15 | 1 | 4.69431520979039 | 0.19249714988302 |
| 6021 | CXorf57 | 5 | 1 | 4.11291052381627 | 0.259937659679162 |
| 6022 | TBC1D8B | 8 | 1 | -2.5884179921902 | 1.95899237769674 |
| 6023 | MORC4 | 4 | 1 | -4.60557530839808 | -0.325006350101721 |
| 6024 | RBM41 | 9 | 1 | 0.522649779721425 | 2.56389059203695 |
| 6025 | NUP62CL | 19 | 1 | -4.56522439439662 | 1.11935438293051 |
| 6026 | PIH1D3 | 14 | 1 | -5.4723803849972 | 0.0799040951115965 |
| 6027 | FRMPD3 | 20 | 1 | 3.70211483518712 | -3.45302711349894 |
| 6028 | PRPS1 | 4 | 1 | -4.54222320039637 | -0.117556973518622 |
| 6029 | TSC22D3 | 10 | 1 | 3.4403712896549 | -1.99098049026896 |

| 6030 | PSMD10 | 3 | 1 | -2.85129855592616 | 0.830273286281335 |
| --- | --- | --- | --- | --- | --- |
| 6031 | COL4A6 | 11 | 1 | -4.04565595109828 | -2.17566429001261 |
| 6032 | COL4A5 | 4 | 1 | -4.26325009782679 | -1.06245686036516 |
| 6033 | AL035425.2 | 3 | 1 | -2.85104177911647 | 0.926452294765222 |
| 6034 | IRS4 | 8 | 1 | -2.21827386339076 | 1.21578182357382 |
| 6035 | AL035425.1 | 8 | 1 | -2.09491764028437 | 1.25535096305441 |
| 6036 | GUCY2F | 4 | 1 | -4.3322231622494 | -0.990394032718432 |
| 6037 | NXT2 | 4 | 1 | -3.74855445344813 | -0.50637735349108 |
| 6038 | KCNE5 | 7 | 1 | -3.07602714021571 | -3.7773569665568 |
| 6039 | ACSL4 | 1 | 1 | 2.90849687139623 | 1.8484450735433 |
| 6040 | TMEM164 | 9 | 1 | 1.60830821077459 | 3.01581801551412 |
| 6041 | AMMECR1 | 8 | 1 | -1.9732258172787 | 1.38877691405843 |
| 6042 | RTL9 | 12 | 1 | 1.60259236375921 | 1.95349349158834 |
| 6043 | CHRDL1 | 4 | 1 | -4.23761676271327 | -0.820395029308093 |
| 6044 | PAK3 | 5 | 1 | 4.59114672224157 | 0.457132474361169 |
| 6045 | CAPN6 | 7 | 1 | -3.81828592737086 | -2.75475691181589 |
| 6046 | DCX | 5 | 1 | 3.59868790189855 | -0.126646026195777 |
| 6047 | ALG13 | 2 | 1 | -0.587983653143718 | 0.94094456809591 |
| 6048 | ALG13-AS1 | 1 | 1 | 1.65486504118078 | 1.59216870444845 |
| 6049 | TRPC5 | 15 | 1 | 5.70320631544225 | 0.892546669421899 |
| 6050 | AMOT | 1 | 1 | 2.7905693201267 | 1.47939075606893 |
[truncated: 526,832 more chars]
