# Supplementary material for: Deciphering the Transcriptional Landscape of Human Pluripotent Stem Cell-Derived GnRH Neurons: The Role of Wnt Signaling in Patterning the Neural Fate
Source: Stem Cells. 2022 Sep 25;40(12):1107–21. doi: 10.1093/stmcls/sxac069 (PMC9806769; doi:10.1093/stmcls/sxac069)
Supplement: sxac069_suppl_Supplementary_Table_S3 [file sxac069_suppl_supplementary_table_s3.docx]

| gene | p_val | avg_logFC | pct.1 | pct.2 | p_val_adj | cluster |
| --- | --- | --- | --- | --- | --- | --- |
| ARL4A | 0 | 1.17235483338222 | 0.689583333 | 0.414583333 | 0 | 0 |
| TMSB15A | 0 | 1.11675423903399 | 1 | 0.693055556 | 0 | 0 |
| NR2F1 | 0 | 1.10989492134474 | 0.692361111 | 0.427083333 | 0 | 0 |
| ID4 | 0 | 0.904527836670536 | 0.681944444 | 0.504166667 | 0 | 0 |
| HES5 | 0 | 0.881597778503472 | 0.675694444 | 0.549305556 | 0 | 0 |
| ARMCX2 | 0 | 0.856715476382219 | 0.66875 | 0.545833333 | 0 | 0 |
| RPS27L | 0 | 0.835987461978202 | 1 | 0.690972222 | 0 | 0 |
| GPC3 | 0 | 0.82661048831689 | 0.69375 | 0.691666667 | 0 | 0 |
| PLP1 | 0 | 0.807641727222288 | 0.679166667 | 0.559722222 | 0 | 0 |
| DMRTA1 | 0 | 0.731824036619598 | 0.675694444 | 0.357638889 | 0 | 0 |
| COTL1 | 0 | 0.696346538729563 | 0.69375 | 0.658333333 | 0 | 0 |
| ARL4C | 0 | 0.683296440884173 | 0.69375 | 0.066666667 | 0 | 0 |
| SFRP2 | 0 | 0.671660509910107 | 1 | 0.690972222 | 0 | 0 |
| ALDH2 | 0 | 0.671203141630114 | 0.692361111 | 0.571527778 | 0 | 0 |
| FABP7 | 0 | 0.655654364694046 | 0.661111111 | 0.047916667 | 0 | 0 |
| HES4 | 0 | 0.649689479629544 | 0.674305556 | 0.465972222 | 0 | 0 |
| WNT2B | 0 | 0.644124558448141 | 0.614583333 | 0.160416667 | 0 | 0 |
| FEZF2 | 0 | 0.636017939817004 | 0.636805556 | 0.350694444 | 0 | 0 |
| CALM1 | 0 | 0.633715847739957 | 1 | 0.69375 | 0 | 0 |
| SOX3 | 0 | 0.632862402206611 | 0.645138889 | 0.419444444 | 0 | 0 |
| TMSB4X | 0 | 0.598225864622711 | 1 | 1 | 0 | 0 |
| ARMCX3 | 0 | 0.597527707224161 | 0.681944444 | 0.593055556 | 0 | 0 |
| PHPT1 | 0 | 0.575127060002169 | 1 | 0.688888889 | 0 | 0 |
| PALLD | 0 | 0.572377276345727 | 0.686805556 | 0.611805556 | 0 | 0 |
| TRIM24 | 0 | 0.550096969900359 | 0.691666667 | 0.061111111 | 0 | 0 |
| ZFP36L1 | 0 | 0.545640536329497 | 0.688888889 | 0.6625 | 0 | 0 |
| COL11A1 | 0 | 0.527169120728898 | 0.649305556 | 0.359722222 | 0 | 0 |
| CCN2 | 0 | 0.520891506904976 | 0.621527778 | 0.413194444 | 0 | 0 |
| PURPL | 0 | 0.514952498794153 | 0.553472222 | 0.303472222 | 0 | 0 |
| CROT | 0 | 0.510391351238132 | 0.058333333 | 0.284027778 | 0 | 0 |
| PAX6 | 0 | 0.500194032467144 | 0.689583333 | 0.05625 | 0 | 0 |
| TTYH1 | 0 | 0.484646851438054 | 0.692361111 | 0.649305556 | 0 | 0 |
| ZFP36L2 | 0 | 0.483348245424204 | 0.609027778 | 0.357638889 | 0 | 0 |
| TOX3 | 0 | 0.482191232210147 | 0.690972222 | 0.630555556 | 0 | 0 |
| BAX | 0 | 0.475420455540738 | 0.684722222 | 0.609027778 | 0 | 0 |
| GLI3 | 0 | 0.46595288404725 | 0.683333333 | 0.601388889 | 0 | 0 |
| TIMP3 | 0 | 0.464568551238243 | 0.602083333 | 0.295833333 | 0 | 0 |
| TP53TG1 | 0 | 0.443181397685579 | 0.641666667 | 0.378472222 | 0 | 0 |
| VIM | 0 | 0.439441442734969 | 1 | 0.692361111 | 0 | 0 |
| CEMIP2 | 0 | 0.436356758093821 | 0.682638889 | 0.616666667 | 0 | 0 |
| SMS | 0 | 0.434598665373554 | 1 | 0.690277778 | 0 | 0 |
| ARMCX6 | 0 | 0.429667622054124 | 0.644444444 | 0.478472222 | 0 | 0 |
| PTX3 | 0 | 0.428352967828008 | 0.503472222 | 0.148611111 | 0 | 0 |
| CDH2 | 0 | 0.426014793231055 | 0.691666667 | 0.674305556 | 0 | 0 |
| NEAT1 | 0 | 0.418802290770416 | 0.539583333 | 0.295833333 | 0 | 0 |
| WNT7B | 0 | 0.417711189471115 | 0.502777778 | 0.130555556 | 0 | 0 |
| METRN | 0 | 0.416608453863279 | 0.69375 | 0.067361111 | 0 | 0 |
| CKB | 0 | 0.415184156441086 | 0.692361111 | 0.641666667 | 0 | 0 |
| ISYNA1 | 0 | 0.406194259720775 | 0.684722222 | 0.603472222 | 0 | 0 |
| POU3F2 | 0 | 0.400933813274482 | 0.619444444 | 0.340972222 | 0 | 0 |
| LFNG | 0 | 0.394003606296769 | 0.644444444 | 0.499305556 | 0 | 0 |
| PLAGL1 | 0 | 0.390106243723871 | 0.543055556 | 0.159027778 | 0 | 0 |
| MEIS2 | 0 | 0.387575649919131 | 0.632638889 | 0.370833333 | 0 | 0 |
| ARX | 0 | 0.37260412338923 | 0.445138889 | 0.119444444 | 0 | 0 |
| NRIP1 | 0 | 0.369952442810757 | 0.654166667 | 0.534027778 | 0 | 0 |
| ASCC3 | 0 | 0.362685450206668 | 0.636805556 | 0.522222222 | 0 | 0 |
| CCN1 | 0 | 0.360254078273172 | 0.565972222 | 0.341666667 | 0 | 0 |
| ZNF503 | 0 | 0.352557784986089 | 0.545138889 | 0.264583333 | 0 | 0 |
| ANKRD34B | 0 | 0.34805673779505 | 0.382638889 | 0.068 | 0 | 0 |
| LINC01198 | 0 | 0.346715648108444 | 0.461111111 | 0.077083333 | 0 | 0 |
| COL4A6 | 0 | 0.343747578477577 | 0.625694444 | 0.477083333 | 0 | 0 |
| ZBTB16 | 0 | 0.338711442787601 | 0.642361111 | 0.425694444 | 0 | 0 |
| PHLDA3 | 0 | 0.323127019642663 | 0.452777778 | 0.178472222 | 0 | 0 |
| CDH11 | 0 | 0.323083242148937 | 0.061805556 | 0.390277778 | 0 | 0 |
| SOX1 | 0 | 0.316244639990748 | 0.524305556 | 0.172222222 | 0 | 0 |
| AHCYL1 | 0 | 0.311980973750295 | 0.681944444 | 0.640277778 | 0 | 0 |
| DTX4 | 0 | 0.305128236645308 | 0.54375 | 0.283333333 | 0 | 0 |
| DACH1 | 0 | 0.295755256202663 | 0.553472222 | 0.292361111 | 0 | 0 |
| RHOC | 0 | 0.292988771512761 | 0.611805556 | 0.447222222 | 0 | 0 |
| CST3 | 0 | 0.2895336575952 | 0.66875 | 0.540277778 | 0 | 0 |
| CHCHD2 | 0 | 0.28598867500953 | 0.693055556 | 0.689583333 | 0 | 0 |
| FAM217B | 0 | 0.283241731930787 | 0.563888889 | 0.331944444 | 0 | 0 |
| EPHA7 | 0 | 0.283052974058719 | 0.509722222 | 0.2625 | 0 | 0 |
| ZMAT3 | 0 | 0.279754747075552 | 0.434722222 | 0.170138889 | 0 | 0 |
| NHSL1 | 0 | 0.278198161679533 | 0.510416667 | 0.209722222 | 0 | 0 |
| C1orf61 | 0 | 0.275607758146891 | 0.34375 | 00:01 | 0 | 0 |
| CDH8 | 0 | 0.273135107601121 | 0.504861111 | 0.19375 | 0 | 0 |
| NEK6 | 0 | 0.267479796102214 | 0.582638889 | 0.347916667 | 0 | 0 |
| DRD4 | 0 | 0.260436788353106 | 0.477083333 | 0.158333333 | 0 | 0 |
| AC093866.1 | 0 | 0.257563254431926 | 0.296527778 | 0.073 | 0 | 0 |
| TMEM88 | 2.18750662268049e-307 | 0.266754727112415 | 0.539583333 | 0.297222222 | 4.77532695731152e-303 | 0 |
| FILIP1 | 4.54453612710034e-304 | 0.278395879134864 | 0.5375 | 0.286111111 | 9.92072236546004e-300 | 0 |
| CD63 | 1.16044198038292e-303 | 0.285505442971452 | 0.69375 | 0.69375 | 2.53324484317591e-299 | 0 |
| RGMA | 7.78767194185573e-302 | 0.266928448574443 | 0.622916667 | 0.445138889 | 1.7000487849071e-297 | 0 |
| PLCH1 | 1.23210127961021e-297 | 0.278655304886123 | 0.563888889 | 0.35625 | 2.6896770933891e-293 | 0 |
| WLS | 6.59553736061942e-291 | 0.380315534765279 | 0.684027778 | 0.485416667 | 1.43980580582322e-286 | 0 |
| DDB2 | 3.00752813971207e-286 | 0.29652359163583 | 0.561111111 | 0.377777778 | 6.56543392899146e-282 | 0 |
| FBXO16 | 3.46707934016695e-277 | 0.290268291315213 | 0.521527778 | 0.309027778 | 7.56863419958445e-273 | 0 |
| PRMT2 | 6.21471901775797e-269 | 0.27258834741751 | 0.679861111 | 0.623611111 | 1.35667316157656e-264 | 0 |
| IFITM3 | 1.11263354757885e-267 | 0.317844671596894 | 1 | 0.689583333 | 2.42887903436464e-263 | 0 |

| IGFBP2 | 2.89743183360612e-267 | 0.255751639140042 | 1 | 1 | 6.32509369276217e-263 | 0 |
| --- | --- | --- | --- | --- | --- | --- |
| FDXR | 2.36972345225755e-265 | 0.252054853001268 | 0.453472222 | 0.244444444 | 5.17310629627824e-261 | 0 |
| ACTG1 | 2.13581745272334e-257 | 0.268758581179532 | 1 | 1 | 4.66248949929506e-253 | 0 |
| SLC2A1 | 2.04717400851798e-256 | 0.337200679203079 | 0.657638889 | 0.588888889 | 4.46898086059474e-252 | 0 |
| POLR2L | 1.14938296272166e-252 | 0.251493341185323 | 1 | 0.690277778 | 2.50910300762139e-248 | 0 |
| LIX1 | 4.57441761985588e-251 | 0.324886369346021 | 0.692361111 | 0.619444444 | 9.98595366414539e-247 | 0 |
| BCL2L11 | 5.59407652715247e-248 | 0.287344981245401 | 0.575694444 | 0.397222222 | 1.22118690587738e-243 | 0 |
| IKBIP | 8.99147450561784e-244 | 0.273306264608335 | 0.615972222 | 0.494444444 | 1.96283888457637e-239 | 0 |
| CA4 | 1.97262053081137e-242 | 0.325452131670044 | 0.519444444 | 0.294444444 | 4.30623061876122e-238 | 0 |
| TSPAN18 | 3.2051204109304e-239 | 0.257888688871311 | 0.617361111 | 0.46875 | 6.99677785706107e-235 | 0 |
| NTPCR | 9.90239419226253e-236 | 0.250519075957486 | 0.065972222 | 0.568055556 | 2.16169265217091e-231 | 0 |
| LMO4 | 6.22767359195724e-232 | 0.285473698656771 | 0.685416667 | 0.065972222 | 1.35950114512427e-227 | 0 |
| CLU | 4.72996553259096e-230 | 0.305813409075883 | 0.690277778 | 0.663888889 | 1.03255147576461e-225 | 0 |
| TPM1 | 3.66576379129016e-229 | 0.30465942013588 | 1 | 0.69375 | 8.00236235638641e-225 | 0 |
| JAG1 | 1.58531549029755e-224 | 0.296904726312988 | 0.639583333 | 0.508333333 | 3.46074371531956e-220 | 0 |
| LRATD2 | 6.96338025615883e-219 | 0.259943722550948 | 0.502777778 | 00:45 | 1.52010590991947e-214 | 0 |
| EMX2 | 1.92755494281258e-218 | 0.251354147670864 | 0.474305556 | 0.238194444 | 4.20785244015985e-214 | 0 |
| DLL1 | 9.23148543670639e-216 | 0.29011166273918 | 0.45625 | 0.250694444 | 2.015233270833e-211 | 0 |
| PKM | 8.89236300019883e-213 | 0.250124140096057 | 1 | 0.689583333 | 1.94120284294341e-208 | 0 |
| NR2F2 | 6.32060629846068e-211 | 0.306569667685727 | 0.686805556 | 0.604861111 | 1.37978835495397e-206 | 0 |
| DHFR | 1.81515169591553e-206 | 0.292030253266703 | 0.647916667 | 0.529861111 | 3.96247615218359e-202 | 0 |
| HTATSF1 | 1.48759146858975e-173 | 0.254236494867977 | 0.686111111 | 0.661111111 | 3.24741217593142e-169 | 0 |
| TYMS | 3.77480003736145e-164 | 0.282723664931861 | 0.661111111 | 0.56875 | 8.24038848156004e-160 | 0 |
| SAT1 | 1.17161299492626e-162 | 0.298641997406038 | 0.661111111 | 0.610416667 | 2.55763116792402e-158 | 0 |
| MALAT1 | 1.57274028010808e-159 | 0.311509577942225 | 1 | 1 | 3.43329203147594e-155 | 0 |
| H1FX | 4.82182134072778e-149 | 0.307180067141605 | 0.69375 | 0.688888889 | 1.05260359868088e-144 | 0 |
| TPBG | 1.58499924034945e-141 | 0.294283952513989 | 0.663194444 | 0.614583333 | 3.46005334168286e-137 | 0 |
| CENPE | 3.2209908400943e-43 | 0.289859311577357 | 0.052083333 | 0.446527778 | 7.03142300392586e-39 | 0 |
| DLK1 | 0 | 1.19484675275316 | 1 | 0.69375 | 0 | 1 |
| IGFBP3 | 0 | 0.896969749999697 | 0.584027778 | 0.343055556 | 0 | 1 |
| HES5 | 0 | 0.828800286713892 | 0.6625 | 0.556944444 | 0 | 1 |
| TPM1 | 0 | 0.818364763617743 | 1 | 0.69375 | 0 | 1 |
| FOXG1 | 0 | 0.765749869683973 | 0.629166667 | 0.136111111 | 0 | 1 |
| LINC01551 | 0 | 0.726708861267904 | 0.511805556 | 0.059 | 0 | 1 |
| FZD5 | 0 | 0.669925716872617 | 0.661805556 | 0.511805556 | 0 | 1 |
| PSAT1 | 0 | 0.555767446267489 | 0.683333333 | 0.668055556 | 0 | 1 |
| NAV1 | 0 | 0.538490118631255 | 0.677083333 | 0.642361111 | 0 | 1 |
| HES4 | 0 | 0.528616808050454 | 0.65625 | 0.477777778 | 0 | 1 |
| PTPRZ1 | 0 | 0.516870224469308 | 0.689583333 | 0.674305556 | 0 | 1 |
| PRSS23 | 0 | 0.51592596146529 | 0.69375 | 0.671527778 | 0 | 1 |
| FABP7 | 0 | 0.512547983155301 | 0.653472222 | 0.4875 | 0 | 1 |
| CCND2 | 0 | 0.507399067668554 | 0.688194444 | 0.65 | 0 | 1 |
| TTYH1 | 0 | 0.479301043223513 | 0.690972222 | 0.651388889 | 0 | 1 |
| COL2A1 | 0 | 0.470172928530574 | 0.657638889 | 0.052083333 | 0 | 1 |
| HMGN2 | 0 | 0.467709807042405 | 1 | 1 | 0 | 1 |
| PHGDH | 0 | 0.467183324825338 | 0.681944444 | 0.661805556 | 0 | 1 |
| TTC3 | 0 | 0.452680904149481 | 0.690277778 | 0.675694444 | 0 | 1 |
| VIM | 0 | 0.445107230309225 | 1 | 0.692361111 | 0 | 1 |
| ODC1 | 0 | 0.434051458379366 | 1 | 0.691666667 | 0 | 1 |
| SMS | 0 | 0.430817688444222 | 1 | 0.690972222 | 0 | 1 |
| CDH2 | 0 | 0.429436341773149 | 0.692361111 | 0.674305556 | 0 | 1 |
| NCALD | 0 | 0.428708072508662 | 0.596527778 | 0.315972222 | 0 | 1 |
| PKDCC | 0 | 0.387875378267077 | 0.627083333 | 0.488888889 | 0 | 1 |
| ATF3 | 0 | 0.386195292858807 | 0.45625 | 0.196527778 | 0 | 1 |
| ANP32B | 0 | 0.374958111700809 | 0.691666667 | 0.686111111 | 0 | 1 |
| TMSB10 | 0 | 0.371031017323692 | 1 | 1 | 0 | 1 |
| DIPK1C | 0 | 0.357163326821113 | 0.467361111 | 0.073611111 | 0 | 1 |
| EIF1 | 0 | 0.349461420062015 | 1 | 1 | 0 | 1 |
| CA2 | 0 | 0.349330713083734 | 0.05 | 0.24375 | 0 | 1 |
| AC092958.1 | 0 | 0.328102675090908 | 0.426388889 | 0.129861111 | 0 | 1 |
| H2AFY | 0 | 0.324744006126516 | 0.689583333 | 0.663888889 | 0 | 1 |
| BEX3 | 0 | 0.303642146502956 | 1 | 0.69375 | 0 | 1 |
| SOX1 | 0 | 0.285009766421395 | 0.481944444 | 0.195138889 | 0 | 1 |
| TUBA1A | 1.76460266492244e-306 | 0.378792524922961 | 1 | 1 | 3.85212761752568e-302 | 1 |
| FEZF2 | 3.76455380718829e-304 | 0.438509421299244 | 0.610416667 | 0.366666667 | 8.21802096109203e-300 | 1 |
| CHAC1 | 8.70475354974122e-304 | 0.391584801644463 | 00:06 | 0.178472222 | 1.90024769990851e-299 | 1 |
| SIX3 | 1.32601266860611e-301 | 0.356994686571151 | 0.608333333 | 0.431944444 | 2.89468565556714e-297 | 1 |
| EZH2 | 6.91454866370719e-299 | 0.355323458117164 | 0.642361111 | 0.571527778 | 1.50944597328728e-294 | 1 |
| MTHFD2 | 1.21268029540468e-294 | 0.483208448089771 | 0.596527778 | 0.463194444 | 2.64728108486842e-290 | 1 |
| SIPA1L2 | 1.1415357024232e-293 | 0.279631582860679 | 0.495138889 | 0.283333333 | 2.49197243838984e-289 | 1 |
| CSRP2 | 1.27594464434522e-289 | 0.335796943143093 | 0.69375 | 0.690277778 | 2.78538715860561e-285 | 1 |
| CTXN1 | 3.02614143500812e-288 | 0.305102527907327 | 0.622222222 | 0.502777778 | 6.60606675262273e-284 | 1 |
| CDH11 | 2.86784336323936e-285 | 0.375697684040661 | 0.59375 | 0.404166667 | 6.26050206195152e-281 | 1 |
| MAP1B | 6.16897635937482e-281 | 0.310631799054602 | 0.69375 | 0.688888889 | 1.34668753925152e-276 | 1 |
| MARCKSL1 | 1.07473826584074e-278 | 0.253139988602204 | 1 | 1 | 2.34615363433033e-274 | 1 |
| SPART | 1.47973474957787e-277 | 0.303063270060538 | 0.603472222 | 0.466666667 | 3.23026095832849e-273 | 1 |
| ID4 | 3.03810800302438e-276 | 0.538143961076403 | 0.642361111 | 0.520138889 | 6.63218977060221e-272 | 1 |
| KCNK12 | 1.21451332644199e-273 | 0.286057488151194 | 0.43125 | 0.209722222 | 2.65128259162286e-269 | 1 |
| SOX9 | 1.88385559002962e-272 | 0.3201431765576 | 0.434722222 | 0.215972222 | 4.11245675303465e-268 | 1 |
| ZBTB16 | 2.80971378572841e-271 | 0.360937120185067 | 0.614583333 | 0.440277778 | 6.13360519424511e-267 | 1 |
| SOX2 | 1.45401179512106e-266 | 0.32343159288453 | 1 | 0.692361111 | 3.17410774874927e-262 | 1 |
| BEX1 | 3.28864984713093e-262 | 0.40427198114456 | 0.628472222 | 0.503472222 | 7.17912261628682e-258 | 1 |
| NR2F1 | 3.29255146032372e-261 | 0.347177528068413 | 0.669444444 | 0.441666667 | 7.18763983788668e-257 | 1 |
| AL139246.5 | 3.85605962631389e-261 | 0.357326750725006 | 0.425 | 0.195138889 | 8.41777816424322e-257 | 1 |
| ZNF428 | 2.6033971928134e-252 | 0.288517546433655 | 0.669444444 | 0.638194444 | 5.68321607191165e-248 | 1 |
| TMEM38B | 1.01441317289572e-250 | 0.327526844752588 | 0.060416667 | 0.518055556 | 2.21446395643135e-246 | 1 |
| LUC7L2 | 2.75336096101411e-250 | 0.30002383070878 | 0.664583333 | 0.622222222 | 6.0105869778938e-246 | 1 |
| DPYSL2 | 6.28275041497205e-249 | 0.292675745762228 | 0.690972222 | 0.677777778 | 1.3715244155884e-244 | 1 |
| H2AFZ | 1.32980408861117e-245 | 0.322758389782289 | 1 | 1 | 2.90296232543818e-241 | 1 |
| CALM2 | 6.95946887063045e-241 | 0.280838325502292 | 1 | 1 | 1.51925205445863e-236 | 1 |
| SLC7A11 | 7.62393733351493e-241 | 0.3403468139779 | 0.363888889 | 0.164583333 | 1.66430551990631e-236 | 1 |

| TLE4 | 7.56548190501539e-239 | 0.315957433794441 | 1 | 0.688888889 | 1.65154469986486e-234 | 1 |
| --- | --- | --- | --- | --- | --- | --- |
| TRIM24 | 1.16228911037219e-232 | 0.372031792646039 | 0.067361111 | 0.061805556 | 2.53727712794249e-228 | 1 |
| METRN | 1.72629953386915e-232 | 0.288541002951916 | 0.689583333 | 0.675 | 3.76851188243635e-228 | 1 |
| DDIT4 | 2.24169600525686e-232 | 0.637029811757917 | 0.066666667 | 0.640972222 | 4.89362237947572e-228 | 1 |
| NDUFAF3 | 8.09321331168942e-231 | 0.257960042061891 | 0.597916667 | 0.480555556 | 1.7667484659418e-226 | 1 |
| HERPUD1 | 5.39647407365497e-227 | 0.28522544371878 | 0.054861111 | 0.414583333 | 1.17805029027888e-222 | 1 |
| ATF4 | 3.2214428746864e-224 | 0.289681115895782 | 0.671527778 | 0.647222222 | 7.03240979544041e-220 | 1 |
| PAFAH1B3 | 1.51580893490559e-219 | 0.262155263511332 | 0.678472222 | 0.648611111 | 3.30901090489891e-215 | 1 |
| PHPT1 | 1.65538521849119e-219 | 0.26500444844157 | 0.69375 | 0.689583333 | 3.61370593196627e-215 | 1 |
| IFITM3 | 2.24185074977129e-214 | 0.306132700621965 | 0.69375 | 0.690277778 | 4.89396018675072e-210 | 1 |
| H1FX | 4.01916502148402e-213 | 0.350227106597298 | 1 | 0.688888889 | 8.77383724189961e-209 | 1 |
| VEGFA | 2.42777806238759e-212 | 0.265956795318983 | 0.460416667 | 0.288194444 | 5.29983951019211e-208 | 1 |
| LMNB1 | 8.41415146328187e-212 | 0.321550320192653 | 0.664583333 | 0.617361111 | 1.83680926443443e-207 | 1 |
| NELL2 | 3.66021640068524e-210 | 0.282947246871538 | 0.654166667 | 0.605555556 | 7.99025240269587e-206 | 1 |
| HP1BP3 | 5.6226162343695e-209 | 0.305660942361929 | 0.679861111 | 0.066666667 | 1.22741712396286e-204 | 1 |
| CENPV | 6.87922384081437e-209 | 0.266183260174411 | 0.692361111 | 0.690277778 | 1.50173456444978e-204 | 1 |
| C4orf48 | 1.38790759764089e-208 | 0.254703954889216 | 1 | 0.690277778 | 3.02980228565007e-204 | 1 |
| MCM7 | 1.15769357769873e-206 | 0.314864051176545 | 0.665972222 | 0.602777778 | 2.52724508011632e-202 | 1 |
| NES | 1.11848285859564e-205 | 0.350707813678242 | 0.692361111 | 0.06875 | 2.44164808031429e-201 | 1 |
| KTN1 | 1.76089138441541e-204 | 0.272368456383894 | 0.692361111 | 0.686111111 | 3.84402589217883e-200 | 1 |
| FAM107B | 4.6145024517892e-204 | 0.278599968668506 | 0.615972222 | 0.552777778 | 1.00734588522558e-199 | 1 |
| EIF4EBP1 | 5.73334942822076e-204 | 0.395601117612077 | 0.626388889 | 0.529166667 | 1.25159018018059e-199 | 1 |
| SPRY2 | 4.11505412808596e-201 | 0.348677260312035 | 0.521527778 | 0.404861111 | 8.98316316161164e-197 | 1 |
| SH3RF1 | 3.36916768499272e-198 | 0.26411894668027 | 0.51875 | 0.386805556 | 7.35489305633911e-194 | 1 |
| SPARC | 6.83287459451003e-198 | 0.250892820064866 | 0.661111111 | 0.623611111 | 1.49161652398154e-193 | 1 |
| IDH2 | 4.236002401431e-196 | 0.258585587364486 | 0.605555556 | 0.51875 | 9.24719324232388e-192 | 1 |
| CEP78 | 5.69004801987149e-196 | 0.292454547966187 | 0.630555556 | 0.579861111 | 1.24213748273795e-191 | 1 |
| RAB11FIP1 | 2.28447976275716e-193 | 0.397322117424825 | 0.580555556 | 0.492361111 | 4.98701932209889e-189 | 1 |
| QKI | 3.4050849547201e-191 | 0.289679153764594 | 0.684722222 | 0.670138889 | 7.43330045615398e-187 | 1 |
| MAGED2 | 5.85302953601591e-190 | 0.262315703895136 | 0.06875 | 0.682638889 | 1.27771634771227e-185 | 1 |
| SYNE2 | 2.10288110578818e-189 | 0.371772616004382 | 0.667361111 | 0.640972222 | 4.59058945393559e-185 | 1 |
| LBR | 1.84159933201244e-184 | 0.291550131244211 | 0.643055556 | 0.059027778 | 4.02021134178316e-180 | 1 |
| CKB | 6.68723444720916e-182 | 0.319610607923977 | 0.686111111 | 0.644444444 | 1.45982327982576e-177 | 1 |
| CAPN6 | 1.6716014746505e-180 | 0.30166557071145 | 0.361805556 | 0.186805556 | 3.64910601916203e-176 | 1 |
| TARS | 5.23646508044425e-180 | 0.270195187154246 | 0.639583333 | 0.542361111 | 1.14312032706098e-175 | 1 |
| TUBA1B | 1.05175338293714e-178 | 0.318709140203454 | 1 | 1 | 2.29597763495178e-174 | 1 |
| GARS | 9.6319531113447e-178 | 0.315362621225635 | 0.645138889 | 0.6125 | 2.10265536420655e-173 | 1 |
| LHX2 | 6.89412702736986e-177 | 0.260095626046949 | 0.596527778 | 0.457638889 | 1.50498793007484e-172 | 1 |
| SPRY1 | 2.77238123278294e-176 | 0.328815501997957 | 0.620138889 | 00:08 | 6.05210823116517e-172 | 1 |
| SLC3A2 | 2.65640536112637e-171 | 0.327333512121739 | 0.654861111 | 0.622222222 | 5.79893290333887e-167 | 1 |
| MYO10 | 1.00211931993509e-168 | 0.261943983662841 | 0.66875 | 0.629861111 | 2.18762647541829e-164 | 1 |
| MYL6 | 3.38348266088298e-168 | 0.273851036722517 | 1 | 1 | 7.38614264870755e-164 | 1 |
| ACTB | 2.83055039746039e-166 | 0.275430496366989 | 1 | 1 | 6.17909151765603e-162 | 1 |
| CKAP2 | 3.5444236602047e-164 | 0.319083307463414 | 0.6625 | 0.063888889 | 7.73747685022685e-160 | 1 |
| DNAJC9 | 1.69942500890125e-163 | 0.258723082057526 | 0.636111111 | 0.596527778 | 3.70984479443143e-159 | 1 |
| TSC22D3 | 2.47697460336795e-162 | 0.268214004378873 | 0.499305556 | 0.384722222 | 5.40723555915223e-158 | 1 |
| SHMT2 | 2.02755749009731e-160 | 0.293049500843232 | 0.561111111 | 0.480555556 | 4.42615800088243e-156 | 1 |
| EIF2S2 | 3.4759861031685e-156 | 0.258510480643755 | 0.692361111 | 0.693055556 | 7.58807766321685e-152 | 1 |
| LRP2 | 2.03961203849861e-149 | 0.261433414642852 | 0.621527778 | 0.518055556 | 4.45247308004248e-145 | 1 |
| TYMS | 4.8778009531817e-144 | 0.341711430717229 | 0.636805556 | 0.577777778 | 1.06482394807957e-139 | 1 |
| GINS2 | 4.8441118412958e-141 | 0.281468325082929 | 0.632638889 | 0.552777778 | 1.05746961495487e-136 | 1 |
| NRARP | 1.94091829304819e-122 | 0.252249499218217 | 0.573611111 | 0.520138889 | 4.2370246337242e-118 | 1 |
| IGFBP5 | 9.1388415505108e-121 | 0.3225719805037 | 0.677777778 | 0.620138889 | 1.99500911047651e-116 | 1 |
| PRTG | 1.49260720730556e-120 | 0.273807055199656 | 0.688888889 | 0.656944444 | 3.25836153354803e-116 | 1 |
| SMC1A | 5.41775538180555e-119 | 0.252565933890649 | 0.654861111 | 0.622916667 | 1.18269599984815e-114 | 1 |
| BTG1 | 2.32163329737563e-117 | 0.252000255865383 | 0.661805556 | 0.646527778 | 5.068125488171e-113 | 1 |
| CLSPN | 2.53335031112551e-109 | 0.290454231543925 | 0.597916667 | 0.509027778 | 5.530303729187e-105 | 1 |
| HELLS | 1.09378366208916e-105 | 0.255492564222945 | 0.684722222 | 0.665972222 | 2.38772973434063e-101 | 1 |
| TAGLN | 2.52546942102988e-103 | 1.21086445444918 | 0.402083333 | 0.309722222 | 5.51309974610822e-99 | 1 |
| AUXG01000058. | 11.59995440758518e-101 | 0.281880073671849 | 0.476388889 | 0.3625 | 3.49270047175846e-97 | 1 |
| DUT | 1.11907070794309e-97 | 0.276388835149361 | 0.065277778 | 0.060416667 | 2.44293135543975e-93 | 1 |
| CALD1 | 2.72756462839508e-84 | 0.377588608294571 | 0.688194444 | 0.684722222 | 5.95427358378645e-80 | 1 |
| CENPF | 7.28898777620211e-79 | 0.418746608573762 | 0.654861111 | 0.619444444 | 1.59118603154492e-74 | 1 |
| ACTA2 | 2.58146113726925e-64 | 0.263804829196197 | 0.304861111 | 0.196527778 | 5.63532966265876e-60 | 1 |
| PRC1 | 7.6471551608726e-56 | 0.256654483474698 | 0.470138889 | 0.405555556 | 1.66937397161849e-51 | 1 |
| HIST1H4C | 4.96342359746277e-41 | 0.337483868754793 | 0.606944444 | 0.572222222 | 1.08351537132612e-36 | 1 |
| UBE2C | 0 | 0.890476751734376 | 0.690972222 | 0.48125 | 0 | 2 |
| TOP2A | 0 | 0.880191089735768 | 0.692361111 | 0.547916667 | 0 | 2 |
| HMGB2 | 0 | 0.698692282768007 | 1 | 0.672916667 | 0 | 2 |
| NUSAP1 | 0 | 0.658038605902677 | 0.69375 | 0.49375 | 0 | 2 |
| MKI67 | 0 | 0.638618663916734 | 0.675694444 | 0.3875 | 0 | 2 |
| DLGAP5 | 0 | 0.62395767432736 | 0.06875 | 0.40625 | 0 | 2 |
| CDK1 | 0 | 0.614979657133825 | 0.665277778 | 0.377083333 | 0 | 2 |
| ASPM | 0 | 0.609442986900916 | 0.677083333 | 0.438194444 | 0 | 2 |
| TPX2 | 0 | 0.595008288031304 | 0.683333333 | 0.454166667 | 0 | 2 |
| SGO2 | 0 | 0.555303046659268 | 0.665972222 | 0.397222222 | 0 | 2 |
| AURKA | 0 | 0.545062268700107 | 0.6375 | 0.369444444 | 0 | 2 |
| MIS18BP1 | 0 | 0.540797392111687 | 0.684027778 | 0.513194444 | 0 | 2 |
| CKS2 | 0 | 0.527883749143455 | 1 | 0.682638889 | 0 | 2 |
| CKS1B | 0 | 0.523936891009047 | 1 | 0.627777778 | 0 | 2 |
| BIRC5 | 0 | 0.517059159987994 | 0.693055556 | 0.519444444 | 0 | 2 |
| TTK | 0 | 0.496904361805712 | 0.065972222 | 0.330555556 | 0 | 2 |
| TUBA1B | 0 | 0.468966603261092 | 1 | 1 | 0 | 2 |
| BUB1 | 0 | 0.462352923409168 | 0.654166667 | 0.363194444 | 0 | 2 |
| SGO1 | 0 | 0.460127144994647 | 0.066666667 | 0.399305556 | 0 | 2 |
| DEPDC1 | 0 | 0.458148567691025 | 0.578472222 | 0.270138889 | 0 | 2 |
| CDCA2 | 0 | 0.433116620008053 | 0.640277778 | 0.358333333 | 0 | 2 |
| CDCA8 | 0 | 0.415588306902382 | 0.659027778 | 0.355555556 | 0 | 2 |
| TUBB | 0 | 0.415165196160963 | 1 | 1 | 0 | 2 |
| NUF2 | 0 | 0.414613043319921 | 0.065972222 | 0.354861111 | 0 | 2 |
| GTSE1 | 0 | 0.403923043732083 | 0.063888889 | 0.323611111 | 0 | 2 |

| NCAPG | 0 | 0.400681503768897 | 0.656944444 | 0.372222222 | 0 | 2 |
| --- | --- | --- | --- | --- | --- | --- |
| KIFC1 | 0 | 0.399674310232678 | 0.657638889 | 0.336805556 | 0 | 2 |
| KIF23 | 0 | 0.394834794083414 | 0.609722222 | 0.282638889 | 0 | 2 |
| ID2 | 0 | 0.387336369460444 | 0.652083333 | 0.411805556 | 0 | 2 |
| KIF14 | 0 | 0.359598142357952 | 0.582638889 | 0.286111111 | 0 | 2 |
| NDC80 | 0 | 0.33121385432516 | 0.620833333 | 0.289583333 | 0 | 2 |
| SPC25 | 0 | 0.329550712384534 | 0.645138889 | 0.325694444 | 0 | 2 |
| HJURP | 0 | 0.311961419665434 | 0.601388889 | 0.275 | 0 | 2 |
| KIF11 | 1.3152427350251e-306 | 0.404988570550431 | 0.658333333 | 0.39375 | 2.87117489055979e-302 | 2 |
| MAD2L1 | 1.63455411018332e-304 | 0.402477643659069 | 0.693055556 | 0.525 | 3.56823162253018e-300 | 2 |
| ANLN | 4.76987511249071e-302 | 0.333285699110335 | 0.603472222 | 0.320138889 | 1.04126373705672e-297 | 2 |
| TUBB4B | 3.99486651655593e-300 | 0.518474672264306 | 1 | 0.675694444 | 8.72079360564159e-296 | 2 |
| CENPU | 4.79884193557229e-297 | 0.383556934856064 | 0.692361111 | 0.054166667 | 1.04758719453543e-292 | 2 |
| STMN1 | 1.53454991681284e-295 | 0.372123651796072 | 1 | 1 | 3.34992246840243e-291 | 2 |
| KIF2C | 7.4828246082057e-294 | 0.313188759021832 | 0.6 | 0.2875 | 1.6335006119713e-289 | 2 |
| TACC3 | 5.38288107946137e-292 | 0.388520739329989 | 0.654861111 | 0.38125 | 1.17508293964642e-287 | 2 |
| CKAP2L | 8.97304898344594e-286 | 0.281996372310509 | 0.531944444 | 0.231944444 | 1.95881659308625e-281 | 2 |
| RTKN2 | 1.19034304878854e-282 | 0.314148707168247 | 0.632638889 | 0.373611111 | 2.59851887550539e-278 | 2 |
| ID3 | 5.84606026138389e-282 | 0.611668664033178 | 0.06875 | 0.526388889 | 1.2761949550601e-277 | 2 |
| CCNB1 | 3.38758088839426e-281 | 0.585592284830566 | 0.685416667 | 0.523611111 | 7.39508907936467e-277 | 2 |
| CCNB2 | 8.62196578029954e-275 | 0.614214293188147 | 0.681944444 | 0.554861111 | 1.88217512983939e-270 | 2 |
| RAD51AP1 | 6.06433398578553e-274 | 0.326732553376729 | 0.663888889 | 0.438194444 | 1.32384410909698e-269 | 2 |
| BUB3 | 4.25832494471301e-267 | 0.388399967679009 | 0.692361111 | 0.636111111 | 9.29592335430851e-263 | 2 |
| CENPF | 8.06705096502109e-267 | 0.574258214354631 | 0.692361111 | 0.613888889 | 1.7610372256641e-262 | 2 |
| KPNA2 | 4.50299346276472e-265 | 0.551292293137366 | 0.69375 | 0.678472222 | 9.83003472921538e-261 | 2 |
| CDCA3 | 1.01668976264083e-263 | 0.258192571498506 | 0.535416667 | 0.239583333 | 2.21943375184493e-259 | 2 |
| ECT2 | 4.83152835706832e-262 | 0.421553389009563 | 0.668055556 | 0.504166667 | 1.05472264034801e-257 | 2 |
| AURKB | 3.11979528483592e-261 | 0.319383548734546 | 0.628472222 | 0.34375 | 6.81051310679682e-257 | 2 |
| ARHGAP11A | 6.50942047573189e-257 | 0.324510423008228 | 0.58125 | 00:48 | 1.42100648985227e-252 | 2 |
| KIF4A | 1.31754939346911e-254 | 0.310861435736848 | 0.601388889 | 0.321527778 | 2.87621032594307e-250 | 2 |
| G2E3 | 1.05417763524251e-249 | 0.412818009152605 | 0.664583333 | 0.531944444 | 2.30126977773439e-245 | 2 |
| KIF20B | 3.21293044292598e-247 | 0.361007977306497 | 0.674305556 | 0.495833333 | 7.01382715690741e-243 | 2 |
| H2AFV | 4.72104478235664e-246 | 0.274379302279712 | 1 | 0.693055556 | 1.03060407598845e-241 | 2 |
| PLK1 | 1.85137262127935e-245 | 0.422380039462589 | 0.054166667 | 0.275694444 | 4.04154643225283e-241 | 2 |
| KNL1 | 7.91600117702808e-239 | 0.3152509172581 | 0.6 | 0.341666667 | 1.72806305694523e-234 | 2 |
| PRR11 | 1.43339006690889e-234 | 0.305999573675366 | 0.594444444 | 0.358333333 | 3.12909051606211e-230 | 2 |
| CDC20 | 5.71473104386682e-232 | 0.464930410802452 | 0.636805556 | 0.399305556 | 1.24752578687613e-227 | 2 |
| CCNA2 | 9.42068232316115e-232 | 0.309971621162362 | 0.642361111 | 0.353472222 | 2.05653495114608e-227 | 2 |
| DIAPH3 | 5.67777384899827e-230 | 0.254250011537635 | 0.615277778 | 0.345138889 | 1.23945803123632e-225 | 2 |
| CKAP2 | 7.12210389816467e-223 | 0.393185907766943 | 0.690972222 | 0.634722222 | 1.55475528096935e-218 | 2 |
| SMC4 | 3.23533012995137e-216 | 0.352473251698756 | 0.684722222 | 0.560416667 | 7.06272567368385e-212 | 2 |
| HMGB1 | 7.9907371088534e-211 | 0.314013304829121 | 1 | 1 | 1.7443779108627e-206 | 2 |
| PRC1 | 2.87283312594676e-210 | 0.28866277839899 | 0.63125 | 0.377083333 | 6.27139471394178e-206 | 2 |
| CENPA | 3.45564924568752e-206 | 0.288113334938954 | 0.49375 | 0.245138889 | 7.54368230333586e-202 | 2 |
| PTTG1 | 1.74086113054229e-205 | 0.56415146747417 | 0.688888889 | 0.059722222 | 3.80029984797383e-201 | 2 |
| PIMREG | 1.08851619440426e-204 | 0.263307141968032 | 0.061111111 | 0.35625 | 2.3762308523845e-200 | 2 |
| UBE2S | 4.00128268247397e-200 | 0.475751409101334 | 1 | 0.685416667 | 8.73480009584068e-196 | 2 |
| ARL6IP1 | 3.29767911249961e-196 | 0.617094345213325 | 0.69375 | 0.689583333 | 7.19883350258666e-192 | 2 |
| TUBA1C | 1.27356557030571e-195 | 0.573121988240627 | 0.068055556 | 0.584722222 | 2.78019363997736e-191 | 2 |
| PSRC1 | 3.11161198236411e-194 | 0.251671004492305 | 0.563194444 | 0.322916667 | 6.79264895750086e-190 | 2 |
| DYNLL1 | 5.33090943852495e-193 | 0.271309408451179 | 1 | 1 | 1.16373753043e-188 | 2 |
| EPHA4 | 2.25035096572409e-188 | 0.27873594104227 | 0.650694444 | 0.50625 | 4.91251615817569e-184 | 2 |
| HIST1H4C | 1.3060277722785e-184 | 0.498768237697575 | 0.644444444 | 0.567361111 | 2.85105862688398e-180 | 2 |
| CENPE | 1.37034890832913e-183 | 0.392089852536859 | 0.63125 | 0.43125 | 2.9914716668825e-179 | 2 |
| ID1 | 6.39373586730456e-169 | 0.471674020222926 | 0.565972222 | 0.360416667 | 1.39575253983259e-164 | 2 |
| UBE2T | 4.27203132146889e-164 | 0.258683657988107 | 0.685416667 | 0.54375 | 9.32584437476659e-160 | 2 |
| HMMR | 6.91675203709261e-158 | 0.297881188655577 | 0.579166667 | 0.35 | 1.50992696969732e-153 | 2 |
| CKAP5 | 2.30367700738829e-156 | 0.298648116537853 | 0.658333333 | 0.551388889 | 5.02892690712863e-152 | 2 |
| ORC6 | 3.36764845015906e-156 | 0.258743761083516 | 0.688194444 | 0.056944444 | 7.35157656669722e-152 | 2 |
| TRIM59 | 7.75702025460419e-153 | 0.311748506505089 | 0.625694444 | 0.483333333 | 1.69335752158009e-148 | 2 |
| MALAT1 | 1.06537462649139e-152 | 0.262454464021887 | 1 | 1 | 2.32571280963071e-148 | 2 |
| NUCKS1 | 1.70546409422055e-144 | 0.259452682918744 | 1 | 1 | 3.72302811768346e-140 | 2 |
| UACA | 4.42980635659776e-144 | 0.257765254428584 | 0.644444444 | 0.538194444 | 9.67026727645291e-140 | 2 |
| PTN | 3.83572629538507e-143 | 0.366459386702414 | 0.686805556 | 0.668055556 | 8.37339050282561e-139 | 2 |
| CDKN3 | 4.66080153111052e-118 | 0.26874531914659 | 0.613194444 | 0.400694444 | 1.01745297424143e-113 | 2 |
| MT1X | 3.09632144188277e-68 | 0.274507315816553 | 0.56875 | 0.461805556 | 6.75926970763008e-64 | 2 |
| PIK3R3 | 0 | 0.507157368965213 | 0.681944444 | 0.064583333 | 0 | 3 |
| FJX1 | 0 | 0.465981425008268 | 0.665972222 | 0.545833333 | 0 | 3 |
| RPL37 | 0 | 0.337580591338563 | 1 | 1 | 0 | 3 |
| RPS6 | 0 | 0.314239151233087 | 1 | 1 | 0 | 3 |
| RPS4X | 0 | 0.30866838934231 | 1 | 1 | 0 | 3 |
| RPL34 | 0 | 0.295434296118762 | 1 | 1 | 0 | 3 |
| RPL15 | 0 | 0.289105500763404 | 1 | 1 | 0 | 3 |
| RPL10 | 0 | 0.288533081365821 | 1 | 1 | 0 | 3 |
| RPS15A | 0 | 0.282915756957665 | 1 | 1 | 0 | 3 |
| TPT1 | 0 | 0.281948716631847 | 1 | 1 | 0 | 3 |
| RPS23 | 0 | 0.281765434726899 | 1 | 1 | 0 | 3 |
| RPS13 | 0 | 0.277860101842635 | 1 | 1 | 0 | 3 |
| RPS12 | 0 | 0.270684532334 | 1 | 1 | 0 | 3 |
| RPL17 | 0 | 0.269967918660779 | 1 | 1 | 0 | 3 |
| RPS27 | 0 | 0.268443335787347 | 1 | 1 | 0 | 3 |
| RPS3 | 0 | 0.26578715979072 | 1 | 1 | 0 | 3 |
| RPS3A | 0 | 0.265266539597848 | 1 | 1 | 0 | 3 |
| EEF1A1 | 0 | 0.263905475374718 | 1 | 1 | 0 | 3 |
| RPL18 | 0 | 0.262864070032547 | 1 | 1 | 0 | 3 |
| RPS14 | 0 | 0.253763510255164 | 1 | 1 | 0 | 3 |
| RPL10A | 0 | 0.253500315042997 | 1 | 1 | 0 | 3 |
| RPLP0 | 0 | 0.252102892432377 | 1 | 1 | 0 | 3 |
| RPL13A | 8.48699348587355e-305 | 0.274373451523703 | 1 | 1 | 1.85271067796619e-300 | 3 |
| PAMR1 | 6.62735568788719e-296 | 0.292106389117643 | 0.508333333 | 0.251388889 | 1.44675174666577e-291 | 3 |
| PNRC1 | 1.92548805071886e-282 | 0.436702121352574 | 0.679861111 | 0.634027778 | 4.20334041471927e-278 | 3 |

| PCDH18 | 8.51228714062202e-271 | 0.358853686424866 | 0.585416667 | 0.397916667 | 1.85823228279779e-266 | 3 |
| --- | --- | --- | --- | --- | --- | --- |
| EIF3E | 9.0150140048846e-257 | 0.250991972138163 | 1 | 1 | 1.96797755726631e-252 | 3 |
| FRZB | 1.86003349564516e-251 | 0.343373198835235 | 0.635416667 | 0.505555556 | 4.06045312099339e-247 | 3 |
| SNHG29 | 7.17718810345157e-225 | 0.260966996194118 | 1 | 1 | 1.56678016298348e-220 | 3 |
| CCNG2 | 1.86153520807868e-223 | 0.372133221432213 | 0.624305556 | 0.502083333 | 4.06373135923577e-219 | 3 |
| FBLN1 | 2.48220989805674e-209 | 0.333803333588855 | 0.626388889 | 0.477083333 | 5.41866420745786e-205 | 3 |
| NNAT | 1.46636747299661e-206 | 0.438049685114244 | 0.679861111 | 0.674305556 | 3.20108019355161e-202 | 3 |
| BTG2 | 1.17391541092084e-190 | 0.299373976976034 | 0.543055556 | 0.359722222 | 2.5626573420402e-186 | 3 |
| SFRP1 | 3.80200050887645e-188 | 0.264015077807551 | 1 | 0.693055556 | 8.2997671108773e-184 | 3 |
| PTN | 3.24359989170028e-179 | 0.365540679507066 | 0.686805556 | 0.668055556 | 7.08077856358171e-175 | 3 |
| BST2 | 3.45198244073285e-178 | 0.394354003252706 | 0.636111111 | 0.509027778 | 7.53567766811981e-174 | 3 |
| MIAT | 1.0035776029012e-175 | 0.3300498476821 | 0.633333333 | 0.539583333 | 2.19080990713332e-171 | 3 |
| MAPK10 | 5.73507816323479e-170 | 0.343481909272698 | 0.604861111 | 0.514583333 | 1.25196756303415e-165 | 3 |
| SSBP2 | 1.59502936219954e-164 | 0.28194742965681 | 0.672916667 | 0.646527778 | 3.48194909768159e-160 | 3 |
| C6orf141 | 9.36947132175261e-160 | 0.266903317794971 | 0.059722222 | 0.475694444 | 2.0453555895386e-155 | 3 |
| CXXC4 | 7.81261043401219e-159 | 0.278639775960263 | 0.575694444 | 0.450694444 | 1.70549285774486e-154 | 3 |
| TET1 | 7.58786135831908e-155 | 0.265025589103172 | 0.676388889 | 0.661805556 | 1.65643013452106e-150 | 3 |
| MXD4 | 1.03239699759649e-151 | 0.266358377436563 | 0.625694444 | 0.560416667 | 2.25372264575315e-147 | 3 |
| TLE4 | 1.18115550468666e-148 | 0.277726320167689 | 0.69375 | 0.688888889 | 2.57846246673099e-144 | 3 |
| SMOC1 | 9.16189685835662e-146 | 0.283461953020967 | 0.665277778 | 0.594444444 | 2.00004208417925e-141 | 3 |
| ROBO1 | 3.61705151831199e-128 | 0.254332310714528 | 0.054861111 | 0.472916667 | 7.89602346447507e-124 | 3 |
| HES1 | 2.95196070362129e-99 | 0.263459470021683 | 0.047222222 | 0.347916667 | 6.44413021600527e-95 | 3 |
| ID3 | 0 | 1.20190657853767 | 0.693055556 | 0.529166667 | 0 | 4 |
| ID1 | 0 | 0.871003506735733 | 0.655555556 | 0.35 | 0 | 4 |
| BST2 | 0 | 0.696467936803135 | 0.665277778 | 0.050694444 | 0 | 4 |
| WLS | 0 | 0.67145690726302 | 0.691666667 | 0.504861111 | 0 | 4 |
| TNNT1 | 0 | 0.603130288143051 | 0.682638889 | 0.052777778 | 0 | 4 |
| EGLN3 | 0 | 0.578859991205213 | 0.661805556 | 0.445833333 | 0 | 4 |
| GAS5 | 0 | 0.537161056363572 | 1 | 0.693055556 | 0 | 4 |
| SHISA2 | 0 | 0.429393078842955 | 0.586805556 | 0.334027778 | 0 | 4 |
| CCNI | 0 | 0.420770061856474 | 1 | 1 | 0 | 4 |
| ZFAS1 | 0 | 0.356172005715208 | 1 | 1 | 0 | 4 |
| RPS23 | 0 | 0.338191925713613 | 1 | 1 | 0 | 4 |
| RPL26 | 0 | 0.328991198383591 | 1 | 1 | 0 | 4 |
| RPL13A | 0 | 0.326307640196588 | 1 | 1 | 0 | 4 |
| RPS15A | 0 | 0.319742809551795 | 1 | 1 | 0 | 4 |
| RPL24 | 0 | 0.314654875104323 | 1 | 1 | 0 | 4 |
| RPL11 | 0 | 0.314111054355297 | 1 | 1 | 0 | 4 |
| RPS18 | 0 | 0.312675190274692 | 1 | 1 | 0 | 4 |
| RPL34 | 0 | 0.309535859587466 | 1 | 1 | 0 | 4 |
| RPS25 | 0 | 0.293263413726359 | 1 | 1 | 0 | 4 |
| RPL10 | 0 | 0.292755077553862 | 1 | 1 | 0 | 4 |
| RPL29 | 0 | 0.292436110485184 | 1 | 1 | 0 | 4 |
| RPL17 | 0 | 0.291742110300218 | 1 | 1 | 0 | 4 |
| RPS3A | 0 | 0.285940509727024 | 1 | 1 | 0 | 4 |
| RPL37A | 0 | 0.284238937351401 | 1 | 1 | 0 | 4 |
| RPL18A | 0 | 0.283819446621549 | 1 | 1 | 0 | 4 |
| RPL18 | 0 | 0.280216952470644 | 1 | 1 | 0 | 4 |
| RPS14 | 0 | 0.278731535325716 | 1 | 1 | 0 | 4 |
| RPL37 | 0 | 0.278007601477053 | 1 | 1 | 0 | 4 |
| RPS27 | 0 | 0.277832288811411 | 1 | 1 | 0 | 4 |
| RPL12 | 0 | 0.274661367245284 | 1 | 1 | 0 | 4 |
| RPS3 | 0 | 0.274087880073073 | 1 | 1 | 0 | 4 |
| RPS29 | 0 | 0.271549142137933 | 1 | 1 | 0 | 4 |
| RPS13 | 0 | 0.269529657227251 | 1 | 1 | 0 | 4 |
| RPL15 | 0 | 0.267519535974974 | 1 | 1 | 0 | 4 |
| RPL35A | 0 | 0.262386242675412 | 1 | 1 | 0 | 4 |
| RPL14 | 0 | 0.26215123873588 | 1 | 1 | 0 | 4 |
| RPS2 | 0 | 0.260118280281407 | 1 | 1 | 0 | 4 |
| RPL32 | 0 | 0.25926932104725 | 1 | 1 | 0 | 4 |
| RPL30 | 0 | 0.257965089199131 | 1 | 1 | 0 | 4 |
| RPL7A | 0 | 0.254957405238917 | 1 | 1 | 0 | 4 |
| RPL9 | 0 | 0.254668615236036 | 1 | 1 | 0 | 4 |
| RPL10A | 0 | 0.254164062739701 | 1 | 1 | 0 | 4 |
| NACA | 0 | 0.252799214089723 | 1 | 1 | 0 | 4 |
| EEF1A1 | 0 | 0.251210324014648 | 1 | 1 | 0 | 4 |
| RPL5 | 0 | 0.250517243862916 | 1 | 1 | 0 | 4 |
| SLC6A8 | 3.43177896486864e-307 | 0.439542191813483 | 0.663888889 | 0.568055556 | 7.49157348030825e-303 | 4 |
| SNHG29 | 4.83290327358302e-305 | 0.350409771094268 | 1 | 1 | 1.05502278462317e-300 | 4 |
| RPS4X | 1.11025596145281e-303 | 0.258413666577617 | 1 | 1 | 2.42368876385148e-299 | 4 |
| P4HA1 | 6.00344623440007e-296 | 0.460646563076952 | 0.668055556 | 0.591666667 | 1.31055231296954e-291 | 4 |
| LHX5 | 2.2670356765061e-287 | 0.337900088892613 | 0.51875 | 0.253472222 | 4.94893888181281e-283 | 4 |
| VSNL1 | 3.69748263375943e-287 | 0.34440455020299 | 0.525694444 | 0.259027778 | 8.07160458949684e-283 | 4 |
| ZDHHC9 | 1.45376579763961e-286 | 0.395342681252519 | 0.636111111 | 0.495833333 | 3.17357073624727e-282 | 4 |
| PGK1 | 1.62413675291473e-263 | 0.385677715149053 | 0.69375 | 0.688194444 | 3.54549053161286e-259 | 4 |
| IGFBP2 | 1.2188636921435e-261 | 0.359450974657176 | 1 | 1 | 2.66077943994926e-257 | 4 |
| APOE | 6.45879181615537e-256 | 0.44172349582284 | 0.69375 | 0.66875 | 1.40995425346672e-251 | 4 |
| EIF3E | 1.31821397295378e-253 | 0.254698459739911 | 1 | 1 | 2.87766110295811e-249 | 4 |
| NRIP3 | 2.77424199248585e-237 | 0.385817315231119 | 0.538888889 | 0.324305556 | 6.05617026959661e-233 | 4 |
| FST | 2.39084819616089e-235 | 0.463149390166521 | 0.058333333 | 0.386805556 | 5.21922161221923e-231 | 4 |
| TGIF1 | 8.26587143086501e-234 | 0.397608291915302 | 0.665277778 | 0.586805556 | 1.80443973335783e-229 | 4 |
| LAPTM4A | 8.94882786172617e-231 | 0.290789896473183 | 0.69375 | 0.693055556 | 1.95352912221482e-226 | 4 |
| EPHA4 | 5.91841930664401e-220 | 0.372313516278726 | 0.064583333 | 0.510416667 | 1.29199093464039e-215 | 4 |
| CNTNAP2 | 1.00407580267199e-218 | 0.394093461627627 | 0.690277778 | 0.675694444 | 2.19189747723296e-214 | 4 |
| BASP1 | 7.40290585853765e-218 | 0.272945315257871 | 1 | 0.69375 | 1.61605434891877e-213 | 4 |
| FEZF1 | 1.43463031464606e-212 | 0.3656721014241 | 0.635416667 | 0.05 | 3.13179797687234e-208 | 4 |
| IGFBP5 | 1.58004761048847e-211 | 0.509995302648241 | 0.684722222 | 0.622916667 | 3.44924393369633e-207 | 4 |
| CA4 | 1.0986910992879e-210 | 0.350595062038484 | 0.538888889 | 0.314583333 | 2.39844266974548e-206 | 4 |
| MIAT | 5.82428506344639e-204 | 0.403680077112915 | 0.647222222 | 0.538888889 | 1.27144142935035e-199 | 4 |
| TXNIP | 3.68797217499522e-203 | 0.438539493239434 | 0.65625 | 0.556944444 | 8.05084325801457e-199 | 4 |
| EPB41L4A-AS1 | 5.694386028887e-196 | 0.288908153924504 | 0.663888889 | 0.615277778 | 1.24308447010603e-191 | 4 |

| KRT19 | 7.92439522844698e-196 | 0.444809068353889 | 0.607638889 | 0.474305556 | 1.72989547836998e-191 | 4 |
| --- | --- | --- | --- | --- | --- | --- |
| RNF130 | 1.86177683674912e-194 | 0.278798539095453 | 0.693055556 | 0.684722222 | 4.06425883462332e-190 | 4 |
| OTX2 | 2.25383143715276e-193 | 0.31551297247156 | 0.672916667 | 0.616666667 | 4.92011402730448e-189 | 4 |
| TMEM74 | 5.77576131993899e-193 | 0.283288639134783 | 0.599305556 | 0.429166667 | 1.26084869614268e-188 | 4 |
| SINHCAF | 1.98307819000191e-191 | 0.250636147974498 | 0.69375 | 0.69375 | 4.32905968877417e-187 | 4 |
| LHX5-AS1 | 9.70987512621487e-186 | 0.261020939657076 | 0.556944444 | 0.359027778 | 2.11966574005271e-181 | 4 |
| KDM5B | 3.33797669391064e-176 | 0.284619331224957 | 0.69375 | 0.691666667 | 7.28680312280693e-172 | 4 |
| GJA1 | 3.27134727793992e-163 | 0.34307975935099 | 0.682638889 | 0.657638889 | 7.14135110774285e-159 | 4 |
| CCNB1IP1 | 8.92230925843899e-162 | 0.250795505937699 | 0.688888889 | 0.068055556 | 1.94774011111723e-157 | 4 |
| NNAT | 2.00592507829617e-161 | 0.346434462520781 | 0.679861111 | 0.675 | 4.37893444592054e-157 | 4 |
| GPC3 | 7.08379135857923e-156 | 0.299666989847954 | 0.69375 | 0.691666667 | 1.54639165357785e-151 | 4 |
| APP | 7.16353103138081e-143 | 0.292705930725533 | 0.684722222 | 0.675 | 1.56379882415043e-138 | 4 |
| CITED2 | 3.1683671611714e-135 | 0.308629622062017 | 0.623611111 | 0.543055556 | 6.91654551283717e-131 | 4 |
| WDFY1 | 2.3017831710369e-129 | 0.250923084173269 | 0.609722222 | 0.540972222 | 5.02479266237355e-125 | 4 |
| CACHD1 | 3.55535424328692e-124 | 0.257216876464429 | 0.567361111 | 0.461805556 | 7.76133831309534e-120 | 4 |
| DPYSL3 | 1.63567656919188e-119 | 0.27852597957152 | 0.675 | 0.632638889 | 3.57068195054587e-115 | 4 |
| PRR14L | 8.278727222366e-112 | 0.261644812049561 | 0.6375 | 0.586805556 | 1.8072461526425e-107 | 4 |
| ZFHX4 | 3.07803707190341e-105 | 0.286109845937712 | 0.667361111 | 0.598611111 | 6.71935492796514e-101 | 4 |
| POU5F1 | 0 | 2.41807668128076 | 1 | 0.310416667 | 0 | 5 |
| MT2A | 0 | 2.32642091148434 | 0.69375 | 0.410416667 | 0 | 5 |
| L1TD1 | 0 | 2.23221129283935 | 1 | 0.490277778 | 0 | 5 |
| CD24 | 0 | 1.88679691195866 | 1 | 0.672916667 | 0 | 5 |
| TERF1 | 0 | 1.87335765194297 | 1 | 0.649305556 | 0 | 5 |
| MIR302CHG | 0 | 1.85612160360592 | 1 | 0.424305556 | 0 | 5 |
| S100A11 | 0 | 1.615688204553 | 1 | 0.271527778 | 0 | 5 |
| POLR3G | 0 | 1.59261975934552 | 1 | 00:15 | 0 | 5 |
| MT1G | 0 | 1.55430549384307 | 0.682638889 | 0.073611111 | 0 | 5 |
| MT1E | 0 | 1.52315024496073 | 0.688888889 | 0.234027778 | 0 | 5 |
| RARRES2 | 0 | 1.5135069128124 | 1 | 00:15 | 0 | 5 |
| TDGF1 | 0 | 1.50684064799147 | 1 | 00:01 | 0 | 5 |
| UGP2 | 0 | 1.43849469228069 | 1 | 0.59375 | 0 | 5 |
| FOXD3-AS1 | 0 | 1.40691504357142 | 1 | 0.082 | 0 | 5 |
| SEPHS1 | 0 | 1.39195744164484 | 1 | 0.519444444 | 0 | 5 |
| RPL22L1 | 0 | 1.37782865301366 | 1 | 0.596527778 | 0 | 5 |
| MT1M | 0 | 1.29424958227682 | 0.646527778 | 00:08 | 0 | 5 |
| EPCAM | 0 | 1.20997284343383 | 1 | 0.147222222 | 0 | 5 |
| ESRG | 0 | 1.16204953841529 | 1 | 0.072916667 | 0 | 5 |
| DANCR | 0 | 1.13707676959197 | 1 | 0.65625 | 0 | 5 |
| PHC1 | 0 | 1.12959303761276 | 1 | 0.367361111 | 0 | 5 |
| MFGE8 | 0 | 1.12088070260376 | 1 | 0.540277778 | 0 | 5 |
| PMAIP1 | 0 | 1.11385706634142 | 0.69375 | 0.113888889 | 0 | 5 |
| LINC00678 | 0 | 1.09879733604173 | 1 | 0.053 | 0 | 5 |
| CD9 | 0 | 1.08683101467374 | 1 | 0.081 | 0 | 5 |
| DPPA4 | 0 | 1.08478177090376 | 1 | 0.629166667 | 0 | 5 |
| DNMT3B | 0 | 1.08126520862349 | 1 | 0.428472222 | 0 | 5 |
| KRT18 | 0 | 1.08115255443134 | 1 | 0.577083333 | 0 | 5 |
| THY1 | 0 | 1.07716427782183 | 1 | 0.529861111 | 0 | 5 |
| CTSC | 0 | 1.073847544083 | 1 | 0.43125 | 0 | 5 |
| PPP1R14B | 0 | 1.06603550180358 | 1 | 0.664583333 | 0 | 5 |
| LDHA | 0 | 1.03048426142363 | 1 | 0.571527778 | 0 | 5 |
| PRDX1 | 0 | 1.02354175759972 | 1 | 1 | 0 | 5 |
| CLDN6 | 0 | 1.01745909545677 | 1 | 0.297222222 | 0 | 5 |
| CYB5A | 0 | 0.947403317163856 | 1 | 00:53 | 0 | 5 |
| MT1F | 0 | 0.935722978696177 | 0.688194444 | 0.329861111 | 0 | 5 |
| SNRPN | 0 | 0.932409552752598 | 1 | 0.691666667 | 0 | 5 |
| APOE | 0 | 0.928885321937488 | 1 | 0.670138889 | 0 | 5 |
| NFE2L3 | 0 | 0.920206105456147 | 0.69375 | 0.109722222 | 0 | 5 |
| MGST1 | 0 | 0.919151558732868 | 1 | 0.641666667 | 0 | 5 |
| TKT | 0 | 0.892475562921313 | 1 | 0.692361111 | 0 | 5 |
| FABP5 | 0 | 0.863645130201274 | 1 | 0.656944444 | 0 | 5 |
| PPIB | 0 | 0.842777452539946 | 1 | 0.647916667 | 0 | 5 |
| APRT | 0 | 0.839672461742274 | 1 | 0.677777778 | 0 | 5 |
| EIF5A | 0 | 0.839127744871672 | 1 | 0.69375 | 0 | 5 |
| PFN1 | 0 | 0.832038382513836 | 1 | 1 | 0 | 5 |
| HSPE1 | 0 | 0.828559635929136 | 1 | 1 | 0 | 5 |
| HSPD1 | 0 | 0.809469023404799 | 1 | 1 | 0 | 5 |
| AK4 | 0 | 0.807565457441653 | 0.692361111 | 00:25 | 0 | 5 |
| ACAT2 | 0 | 0.798740027426083 | 1 | 0.598611111 | 0 | 5 |
| AC009446.1 | 0 | 0.797899807830423 | 0.692361111 | 0.024 | 0 | 5 |
| S100A10 | 0 | 0.796473157102782 | 0.693055556 | 0.103472222 | 0 | 5 |
| USP44 | 0 | 0.794582044703129 | 1 | 0.606944444 | 0 | 5 |
| NLGN4X | 0 | 0.792460844838933 | 0.690972222 | 0.365972222 | 0 | 5 |
| C1QBP | 0 | 0.788247071376039 | 1 | 0.689583333 | 0 | 5 |
| DBI | 0 | 0.785299995675251 | 1 | 0.684722222 | 0 | 5 |
| ZFP42 | 0 | 0.773957583966733 | 0.69375 | 0.058 | 0 | 5 |
| GABRB3 | 0 | 0.772318713354604 | 0.692361111 | 0.154166667 | 0 | 5 |
| CLDN7 | 0 | 0.764741384334123 | 0.684722222 | 0.032 | 0 | 5 |
| SLC2A3 | 0 | 0.763262586270802 | 0.69375 | 0.395138889 | 0 | 5 |
| PODXL | 0 | 0.757717258596857 | 0.692361111 | 0.364583333 | 0 | 5 |
| SKIL | 0 | 0.75206751049665 | 0.69375 | 0.359027778 | 0 | 5 |
| TIMM13 | 0 | 0.740659267190933 | 1 | 0.671527778 | 0 | 5 |
| PLPP2 | 0 | 0.725069813558436 | 0.678472222 | 0.059 | 0 | 5 |
| SCG3 | 0 | 0.716327621901122 | 0.690277778 | 0.091 | 0 | 5 |
| AC006329.1 | 0 | 0.714616807521953 | 0.690277778 | 0.081 | 0 | 5 |
| MSH2 | 0 | 0.712426858440946 | 1 | 0.568055556 | 0 | 5 |
| CNMD | 0 | 0.705190285964604 | 0.684722222 | 0.063 | 0 | 5 |
| CCDC85B | 0 | 0.70203107210741 | 0.69375 | 0.483333333 | 0 | 5 |
| RBPMS2 | 0 | 0.699124996474937 | 0.690277778 | 0.183333333 | 0 | 5 |
| FDFT1 | 0 | 0.692904944627304 | 1 | 0.584027778 | 0 | 5 |
| DUSP6 | 0 | 0.692866156373865 | 0.68125 | 0.209027778 | 0 | 5 |
| SLC7A8 | 0 | 0.692049549840998 | 0.691666667 | 0.39375 | 0 | 5 |

| SELENOW | 0 | 0.688326903663117 | 1 | 0.688888889 | 0 | 5 |
| --- | --- | --- | --- | --- | --- | --- |
| SQLE | 0 | 0.685229204860585 | 0.692361111 | 0.577777778 | 0 | 5 |
| MT1H | 0 | 0.685050166675633 | 0.260416667 | 0.025 | 0 | 5 |
| TALDO1 | 0 | 0.681929435363293 | 1 | 0.63125 | 0 | 5 |
| TARS | 0 | 0.677310448069443 | 1 | 0.549305556 | 0 | 5 |
| SRM | 0 | 0.669893305586749 | 1 | 0.617361111 | 0 | 5 |
| MID1IP1 | 0 | 0.66415248317165 | 0.690972222 | 0.171527778 | 0 | 5 |
| DHCR24 | 0 | 0.660645853336673 | 0.06875 | 0.200694444 | 0 | 5 |
| MDN1 | 0 | 0.655947843341779 | 0.684027778 | 0.363888889 | 0 | 5 |
| LDHB | 0 | 0.654734556801593 | 1 | 1 | 0 | 5 |
| GNG4 | 0 | 0.651501235603731 | 0.690972222 | 0.21875 | 0 | 5 |
| UCHL1 | 0 | 0.651264607356685 | 1 | 0.678472222 | 0 | 5 |
| GRID2 | 0 | 0.650905674078624 | 0.682638889 | 0.073 | 0 | 5 |
| FGFBP3 | 0 | 0.649263641169007 | 0.690277778 | 0.35 | 0 | 5 |
| DUT | 0 | 0.64710358720334 | 1 | 0.60625 | 0 | 5 |
| GSTP1 | 0 | 0.646247619730227 | 1 | 1 | 0 | 5 |
| ATP5MC1 | 0 | 0.640917740258894 | 1 | 0.625694444 | 0 | 5 |
| MAL2 | 0 | 0.639878590759887 | 0.06875 | 0.10625 | 0 | 5 |
| NME4 | 0 | 0.633206110851274 | 1 | 0.683333333 | 0 | 5 |
| ACAA2 | 0 | 0.625627346834863 | 0.69375 | 0.439583333 | 0 | 5 |
| SCD | 0 | 0.624755752031643 | 1 | 0.572916667 | 0 | 5 |
| SNHG5 | 0 | 0.622515043672076 | 1 | 0.681944444 | 0 | 5 |
| EIF4EBP1 | 0 | 0.619449355313142 | 0.69375 | 0.535416667 | 0 | 5 |
| JARID2 | 0 | 0.61724109949599 | 0.693055556 | 0.546527778 | 0 | 5 |
| APOC1 | 0 | 0.614815624479945 | 0.692361111 | 00:45 | 0 | 5 |
| IDH1 | 0 | 0.614311394631794 | 1 | 0.538888889 | 0 | 5 |
| SLC25A5 | 0 | 0.613953670427445 | 1 | 0.690277778 | 0 | 5 |
| CYCS | 0 | 0.613761842678521 | 1 | 0.688194444 | 0 | 5 |
| ITM2C | 0 | 0.611645389096434 | 1 | 0.589583333 | 0 | 5 |
| VDAC1 | 0 | 0.610535594385127 | 1 | 0.684027778 | 0 | 5 |
| PEBP1 | 0 | 0.610298270648773 | 1 | 1 | 0 | 5 |
| CYBA | 0 | 0.609232673949851 | 1 | 0.63125 | 0 | 5 |
| FDPS | 0 | 0.605164461058971 | 1 | 0.6375 | 0 | 5 |
| NME1 | 0 | 0.605059563766748 | 1 | 0.659027778 | 0 | 5 |
| SLC16A1 | 0 | 0.604442794635511 | 1 | 0.608333333 | 0 | 5 |
| PGRMC1 | 0 | 0.60282706318978 | 1 | 0.663888889 | 0 | 5 |
| DMKN | 0 | 0.602607130981758 | 1 | 0.622916667 | 0 | 5 |
| CHGA | 0 | 0.601685497604969 | 0.668055556 | 0.043 | 0 | 5 |
| ISOC2 | 0 | 0.599847005401403 | 0.693055556 | 0.380555556 | 0 | 5 |
| GPC4 | 0 | 0.599770604618209 | 0.690277778 | 0.376388889 | 0 | 5 |
| SLIRP | 0 | 0.596102605337812 | 1 | 0.69375 | 0 | 5 |
| TXNDC17 | 0 | 0.594474113846123 | 1 | 0.690277778 | 0 | 5 |
| HMGCS1 | 0 | 0.592075516579712 | 0.684027778 | 0.457638889 | 0 | 5 |
| PRELID1 | 0 | 0.591766679218545 | 1 | 0.690972222 | 0 | 5 |
| EZR | 0 | 0.591558318239011 | 1 | 0.565277778 | 0 | 5 |
| SET | 0 | 0.58988338527615 | 1 | 1 | 0 | 5 |
| NCL | 0 | 0.587938946602846 | 1 | 1 | 0 | 5 |
| TUBA1C | 0 | 0.584185897705675 | 1 | 0.590972222 | 0 | 5 |
| NLN | 0 | 0.583746518697475 | 0.689583333 | 0.321527778 | 0 | 5 |
| VRTN | 0 | 0.583331270010144 | 0.684722222 | 00:12 | 0 | 5 |
| CYP51A1 | 0 | 0.577786175886068 | 1 | 0.586805556 | 0 | 5 |
| ENO1 | 0 | 0.571830905797765 | 1 | 0.69375 | 0 | 5 |
| MICOS10 | 0 | 0.567957110974734 | 1 | 0.676388889 | 0 | 5 |
| GPM6B | 0 | 0.565632230855933 | 1 | 0.053472222 | 0 | 5 |
| NASP | 0 | 0.565228036932848 | 1 | 0.69375 | 0 | 5 |
| MAD2L2 | 0 | 0.562886385096279 | 0.69375 | 0.572916667 | 0 | 5 |
| NANOG | 0 | 0.561567225220357 | 0.677083333 | 0.029 | 0 | 5 |
| IMPA2 | 0 | 0.552335455134302 | 1 | 0.434722222 | 0 | 5 |
| DDT | 0 | 0.55211986598711 | 1 | 0.672916667 | 0 | 5 |
| FLT1 | 0 | 0.552092593045313 | 0.6625 | 0.022 | 0 | 5 |
| RPS26 | 0 | 0.552009194921019 | 1 | 1 | 0 | 5 |
| PDIA4 | 0 | 0.549495943370114 | 1 | 0.584722222 | 0 | 5 |
| HLA-C | 0 | 0.548973348913784 | 1 | 0.523611111 | 0 | 5 |
| CD320 | 0 | 0.548473385753123 | 0.690972222 | 0.395138889 | 0 | 5 |
| REXO2 | 0 | 0.548017816038135 | 1 | 0.511111111 | 0 | 5 |
| HERC2 | 0 | 0.546288953576097 | 0.692361111 | 0.407638889 | 0 | 5 |
| FKBP4 | 0 | 0.544577373751879 | 1 | 0.566666667 | 0 | 5 |
| SERBP1 | 0 | 0.544530571492657 | 1 | 1 | 0 | 5 |
| GAL | 0 | 0.542687292380856 | 0.672916667 | 0.042 | 0 | 5 |
| LARS | 0 | 0.539443502905896 | 1 | 0.654166667 | 0 | 5 |
| MRPL34 | 0 | 0.539273729233546 | 0.693055556 | 0.05 | 0 | 5 |
| LAPTM4B | 0 | 0.538717818886898 | 1 | 0.691666667 | 0 | 5 |
| PARP1 | 0 | 0.538683348211271 | 1 | 0.681944444 | 0 | 5 |
| RCC2 | 0 | 0.537925218004115 | 1 | 0.584027778 | 0 | 5 |
| PAICS | 0 | 0.537253021355254 | 1 | 0.686805556 | 0 | 5 |
| RAN | 0 | 0.532470044463794 | 1 | 1 | 0 | 5 |
| ADSL | 0 | 0.529160140299768 | 1 | 00:08 | 0 | 5 |
| PPM1G | 0 | 0.528824254050633 | 1 | 0.684027778 | 0 | 5 |
| ZIC2 | 0 | 0.526543579356002 | 1 | 0.573611111 | 0 | 5 |
| RNASEH2B | 0 | 0.526383863300554 | 0.690277778 | 0.297222222 | 0 | 5 |
| RAB34 | 0 | 0.526261990074776 | 1 | 0.55 | 0 | 5 |
| RAC3 | 0 | 0.525712210802735 | 0.69375 | 0.399305556 | 0 | 5 |
| PYCARD | 0 | 0.525678126162281 | 0.681944444 | 0.043 | 0 | 5 |
| CDK2AP1 | 0 | 0.525666460401995 | 1 | 0.65 | 0 | 5 |
| CDH1 | 0 | 0.524461886758071 | 0.665277778 | 0.026 | 0 | 5 |
| PLS3 | 0 | 0.523486016588574 | 1 | 0.563194444 | 0 | 5 |
| HMGB3 | 0 | 0.523435096461164 | 1 | 0.679861111 | 0 | 5 |
| PTMA | 0 | 0.52239433695929 | 1 | 1 | 0 | 5 |
| IQGAP2 | 0 | 0.521989918618129 | 0.690277778 | 0.39375 | 0 | 5 |
| SLC25A39 | 0 | 0.521989452002741 | 1 | 0.486805556 | 0 | 5 |
| MRPS34 | 0 | 0.519592326365283 | 1 | 0.682638889 | 0 | 5 |

| TCF7L1 | 0 | 0.516332436990637 | 0.06875 | 0.043055556 | 0 | 5 |
| --- | --- | --- | --- | --- | --- | --- |
| CHCHD10 | 0 | 0.515476049613257 | 0.69375 | 0.422916667 | 0 | 5 |
| CCT5 | 0 | 0.512067595419041 | 1 | 0.691666667 | 0 | 5 |
| ERBB2 | 0 | 0.511521340301096 | 0.686805556 | 0.336805556 | 0 | 5 |
| TIMM10 | 0 | 0.509105315863491 | 0.69375 | 0.567361111 | 0 | 5 |
| DAZAP1 | 0 | 0.507566625521683 | 1 | 0.604861111 | 0 | 5 |
| MCM3 | 0 | 0.506978513063363 | 1 | 0.588888889 | 0 | 5 |
| SSBP1 | 0 | 0.506343772277015 | 1 | 0.69375 | 0 | 5 |
| C20orf27 | 0 | 0.50625574622956 | 0.693055556 | 0.481944444 | 0 | 5 |
| HMGB1 | 0 | 0.505002197458828 | 1 | 1 | 0 | 5 |
| NQO2 | 0 | 0.504407303467632 | 0.685416667 | 0.296527778 | 0 | 5 |
| RAB13 | 0 | 0.504042228664339 | 1 | 0.599305556 | 0 | 5 |
| PGAM1 | 0 | 0.503488820478884 | 1 | 0.690277778 | 0 | 5 |
| MRPS15 | 0 | 0.498720806252326 | 1 | 0.063888889 | 0 | 5 |
| FOXH1 | 0 | 0.495063126363742 | 0.675694444 | 0.032 | 0 | 5 |
| CHCHD2 | 0 | 0.494367433365853 | 1 | 0.690277778 | 0 | 5 |
| RRBP1 | 0 | 0.492327755890232 | 0.69375 | 0.052083333 | 0 | 5 |
| TPM3 | 0 | 0.492242193565784 | 1 | 0.692361111 | 0 | 5 |
| SPINT2 | 0 | 0.49109592323308 | 1 | 0.627777778 | 0 | 5 |
| HSPA9 | 0 | 0.490780111510119 | 1 | 0.066666667 | 0 | 5 |
| TRIML2 | 0 | 0.489998340405695 | 0.660416667 | 0.025 | 0 | 5 |
| GADD45GIP1 | 0 | 0.488921843190157 | 1 | 0.685416667 | 0 | 5 |
| CDCA7L | 0 | 0.488422793153917 | 0.690972222 | 0.346527778 | 0 | 5 |
| CTBP2 | 0 | 0.488320273262989 | 1 | 0.599305556 | 0 | 5 |
| COX5A | 0 | 0.487158480870012 | 1 | 0.692361111 | 0 | 5 |
| TMEM147 | 0 | 0.486765494809062 | 1 | 0.061111111 | 0 | 5 |
| PSIP1 | 0 | 0.483427037000531 | 1 | 1 | 0 | 5 |
| PA2G4 | 0 | 0.481810865848329 | 1 | 0.689583333 | 0 | 5 |
| AC104461.1 | 0 | 0.481658700464964 | 0.06875 | 0.145138889 | 0 | 5 |
| MTHFD1 | 0 | 0.481557848665077 | 0.686805556 | 0.254166667 | 0 | 5 |
| RUVBL1 | 0 | 0.48119963701031 | 1 | 0.59375 | 0 | 5 |
| ATP1B3 | 0 | 0.481150512330796 | 1 | 0.661111111 | 0 | 5 |
| ZSCAN10 | 0 | 0.481023302904129 | 0.679861111 | 0.065 | 0 | 5 |
| TIMM8B | 0 | 0.479893038151666 | 1 | 0.066666667 | 0 | 5 |
| GPATCH4 | 0 | 0.479614401329562 | 0.692361111 | 0.529166667 | 0 | 5 |
| RANBP1 | 0 | 0.478849264027255 | 1 | 1 | 0 | 5 |
| PPIA | 0 | 0.478382001220298 | 1 | 1 | 0 | 5 |
| HSD17B4 | 0 | 0.476486171121948 | 1 | 0.570138889 | 0 | 5 |
| HSP90AA1 | 0 | 0.475868906095585 | 1 | 1 | 0 | 5 |
| IGFBP4 | 0 | 0.473106160634169 | 0.685416667 | 0.271527778 | 0 | 5 |
| NME2 | 0 | 0.472206704621597 | 1 | 1 | 0 | 5 |
| SRSF2 | 0 | 0.471683654478814 | 1 | 0.688194444 | 0 | 5 |
| OCIAD2 | 0 | 0.470337031361122 | 0.69375 | 0.591666667 | 0 | 5 |
| EIF2AK4 | 0 | 0.470274227680954 | 0.693055556 | 0.552083333 | 0 | 5 |
| NOP10 | 0 | 0.47023829015208 | 1 | 0.686805556 | 0 | 5 |
| AMD1 | 0 | 0.469634720287317 | 1 | 0.654166667 | 0 | 5 |
| YWHAB | 0 | 0.4695656837137 | 1 | 0.69375 | 0 | 5 |
| NPM1 | 0 | 0.469107552849886 | 1 | 1 | 0 | 5 |
| H2AFJ | 0 | 0.468047356054474 | 0.688888889 | 0.371527778 | 0 | 5 |
| SNHG8 | 0 | 0.464678300547289 | 1 | 0.598611111 | 0 | 5 |
| MRPL12 | 0 | 0.464158291037929 | 1 | 0.05625 | 0 | 5 |
| MRPL57 | 0 | 0.462238608240062 | 1 | 0.671527778 | 0 | 5 |
| NDUFS6 | 0 | 0.462053044563366 | 1 | 0.691666667 | 0 | 5 |
| TSR1 | 0 | 0.459489580172866 | 0.692361111 | 0.547222222 | 0 | 5 |
| ESRP1 | 0 | 0.459221361865023 | 0.066666667 | 00:05 | 0 | 5 |
| ACLY | 0 | 0.458021148536343 | 0.688194444 | 0.442361111 | 0 | 5 |
| SLC25A6 | 0 | 0.454097282289434 | 1 | 0.69375 | 0 | 5 |
| XRCC5 | 0 | 0.449895393868265 | 1 | 0.69375 | 0 | 5 |
| RBPMS | 0 | 0.447780633658934 | 0.69375 | 0.558333333 | 0 | 5 |
| AP1M2 | 0 | 0.447662209701378 | 0.654861111 | 0.037 | 0 | 5 |
| SLC25A1 | 0 | 0.447153578445262 | 0.69375 | 0.397222222 | 0 | 5 |
| GTF3C6 | 0 | 0.446913645662828 | 1 | 0.6375 | 0 | 5 |
| AP000459.2 | 0 | 0.446286692660017 | 0.061111111 | 0.015 | 0 | 5 |
| PSMA7 | 0 | 0.445934523216244 | 1 | 1 | 0 | 5 |
| RBM47 | 0 | 0.445442047914226 | 0.674305556 | 0.127083333 | 0 | 5 |
| TPI1 | 0 | 0.443713568154424 | 1 | 1 | 0 | 5 |
| HES6 | 0 | 0.441036964477426 | 0.675 | 0.178472222 | 0 | 5 |
| LNCPRESS1 | 0 | 0.440834917437118 | 0.063888889 | 0.015 | 0 | 5 |
| PSMB10 | 0 | 0.440513828501666 | 0.683333333 | 0.183333333 | 0 | 5 |
| NHP2 | 0 | 0.439784792718451 | 1 | 0.068055556 | 0 | 5 |
| CXXC5 | 0 | 0.439726009901938 | 0.686805556 | 0.406944444 | 0 | 5 |
| PSMA3 | 0 | 0.439300939115691 | 1 | 0.684722222 | 0 | 5 |
| MAP7 | 0 | 0.437672305016189 | 0.675 | 0.182638889 | 0 | 5 |
| AURKAIP1 | 0 | 0.436915557903232 | 1 | 0.685416667 | 0 | 5 |
| MRPL3 | 0 | 0.43553124347011 | 1 | 0.657638889 | 0 | 5 |
| TOMM5 | 0 | 0.434972388793117 | 1 | 0.686805556 | 0 | 5 |
| ITGA6 | 0 | 0.434467700193232 | 0.68125 | 0.38125 | 0 | 5 |
| GGCT | 0 | 0.432332783951112 | 1 | 0.657638889 | 0 | 5 |
| TOMM40 | 0 | 0.429313011059592 | 1 | 0.634027778 | 0 | 5 |
| GPR160 | 0 | 0.428534539578532 | 0.066666667 | 00:04 | 0 | 5 |
| POMP | 0 | 0.428251118874009 | 1 | 0.69375 | 0 | 5 |
| ENSA | 0 | 0.427904558804493 | 1 | 0.640277778 | 0 | 5 |
| SLC39A14 | 0 | 0.427793765325553 | 0.663888889 | 0.175 | 0 | 5 |
| SLC39A1 | 0 | 0.427515889047822 | 0.69375 | 0.608333333 | 0 | 5 |
| SNRPG | 0 | 0.427480841652894 | 1 | 1 | 0 | 5 |
| ETV4 | 0 | 0.42653751050803 | 0.679166667 | 0.1375 | 0 | 5 |
| CBR1 | 0 | 0.426007591526157 | 0.664583333 | 0.24375 | 0 | 5 |
| HNRNPD | 0 | 0.424884445378633 | 1 | 0.69375 | 0 | 5 |
| EEF1E1 | 0 | 0.424308290663307 | 1 | 0.654166667 | 0 | 5 |
| DCTPP1 | 0 | 0.424035464139689 | 0.69375 | 0.553472222 | 0 | 5 |
| HK1 | 0 | 0.423332246601564 | 0.681944444 | 00:38 | 0 | 5 |

| PSMD1 | 0 | 0.422060612155472 | 0.69375 | 0.661805556 | 0 | 5 |
| --- | --- | --- | --- | --- | --- | --- |
| CEBPZ | 0 | 0.421358796479426 | 1 | 0.670138889 | 0 | 5 |
| NR2F6 | 0 | 0.420240942806659 | 0.69375 | 0.570833333 | 0 | 5 |
| PSMA2 | 0 | 0.419926128474545 | 1 | 0.69375 | 0 | 5 |
| PRDX5 | 0 | 0.419801726155858 | 1 | 0.688194444 | 0 | 5 |
| ATP5F1D | 0 | 0.419114789500322 | 1 | 0.685416667 | 0 | 5 |
| PAK1 | 0 | 0.418726414949384 | 0.686111111 | 0.395138889 | 0 | 5 |
| HNRNPF | 0 | 0.416817568428505 | 1 | 0.682638889 | 0 | 5 |
| CARHSP1 | 0 | 0.416364883817507 | 1 | 0.685416667 | 0 | 5 |
| MIF | 0 | 0.414423809532087 | 1 | 1 | 0 | 5 |
| PYCR1 | 0 | 0.411136201143848 | 0.691666667 | 0.352083333 | 0 | 5 |
| PDLIM1 | 0 | 0.409610885210378 | 0.682638889 | 0.25625 | 0 | 5 |
| PHB2 | 0 | 0.407898677862092 | 1 | 0.682638889 | 0 | 5 |
| RRM2 | 0 | 0.40725588423584 | 0.689583333 | 0.325 | 0 | 5 |
| PHB | 0 | 0.405371637019999 | 1 | 0.686111111 | 0 | 5 |
| PDIA6 | 0 | 0.404938442970641 | 1 | 0.69375 | 0 | 5 |
| EBP | 0 | 0.404002385334197 | 0.69375 | 0.540972222 | 0 | 5 |
| DSG2 | 0 | 0.403972758646188 | 0.068055556 | 0.397222222 | 0 | 5 |
| GCHFR | 0 | 0.403587607293822 | 0.664583333 | 0.089 | 0 | 5 |
| KLHL4 | 0 | 0.40281559308541 | 0.629861111 | 00:05 | 0 | 5 |
| TRAPPC2L | 0 | 0.402294559828664 | 1 | 0.574305556 | 0 | 5 |
| PSMD11 | 0 | 0.400232628692831 | 1 | 0.661111111 | 0 | 5 |
| IQGAP1 | 0 | 0.399746086889767 | 0.68125 | 00:48 | 0 | 5 |
| PDIA3 | 0 | 0.398639223130361 | 1 | 0.691666667 | 0 | 5 |
| COA4 | 0 | 0.397068454537254 | 1 | 0.6375 | 0 | 5 |
| SDF2L1 | 0 | 0.396649638909154 | 0.686111111 | 0.361805556 | 0 | 5 |
| RRAS2 | 0 | 0.39433032202618 | 0.693055556 | 0.545833333 | 0 | 5 |
| LIMD2 | 0 | 0.394247895746114 | 0.688194444 | 0.313194444 | 0 | 5 |
| PSMA5 | 0 | 0.393756320522696 | 1 | 0.656944444 | 0 | 5 |
| NT5DC2 | 0 | 0.393390287196166 | 1 | 0.602777778 | 0 | 5 |
| APEX1 | 0 | 0.392450294283466 | 1 | 0.689583333 | 0 | 5 |
| ZNF483 | 0 | 0.389450060044215 | 0.675694444 | 0.35 | 0 | 5 |
| EIF6 | 0 | 0.388991134407033 | 0.69375 | 0.571527778 | 0 | 5 |
| NTHL1 | 0 | 0.388766702742359 | 0.691666667 | 0.415277778 | 0 | 5 |
| RABGAP1L | 0 | 0.388060905746696 | 0.066666667 | 0.170833333 | 0 | 5 |
| NUDC | 0 | 0.385897127348416 | 1 | 0.684722222 | 0 | 5 |
| PRICKLE1 | 0 | 0.385670553683393 | 0.623611111 | 0.088888889 | 0 | 5 |
| TCOF1 | 0 | 0.384529937835386 | 0.690972222 | 0.477777778 | 0 | 5 |
| FASN | 0 | 0.382882783054183 | 0.684722222 | 0.419444444 | 0 | 5 |
| EPHA1 | 0 | 0.382538902024611 | 0.658333333 | 0.025 | 0 | 5 |
| UCHL3 | 0 | 0.381547834993853 | 0.69375 | 0.417361111 | 0 | 5 |
| SNRPD1 | 0 | 0.380985829082005 | 1 | 0.69375 | 0 | 5 |
| MCM5 | 0 | 0.380534661753895 | 0.688888889 | 0.363888889 | 0 | 5 |
| AFG3L2 | 0 | 0.38046218598867 | 0.688888889 | 0.441666667 | 0 | 5 |
| PDPN | 0 | 0.378785516604911 | 0.679861111 | 0.21875 | 0 | 5 |
| CYP2S1 | 0 | 0.377390893789001 | 0.663888889 | 0.111805556 | 0 | 5 |
| AIF1L | 0 | 0.377390828817611 | 0.690277778 | 0.454861111 | 0 | 5 |
| TNIK | 0 | 0.375418331080646 | 0.605555556 | 0.054 | 0 | 5 |
| KIF1A | 0 | 0.37365734701186 | 0.676388889 | 0.26875 | 0 | 5 |
| EIF1AX | 0 | 0.372269183860639 | 1 | 0.689583333 | 0 | 5 |
| PSMA4 | 0 | 0.369294745747564 | 1 | 0.691666667 | 0 | 5 |
| EMG1 | 0 | 0.367665292765924 | 0.684027778 | 0.307638889 | 0 | 5 |
| ARPC1B | 0 | 0.367247340372617 | 0.69375 | 0.48125 | 0 | 5 |
| MTAP | 0 | 0.367205702342508 | 0.68125 | 0.372916667 | 0 | 5 |
| CFAP298 | 0 | 0.366989971720063 | 0.69375 | 0.635416667 | 0 | 5 |
| NANS | 0 | 0.366597952351864 | 0.68125 | 0.226388889 | 0 | 5 |
| SORD | 0 | 0.366355905156513 | 0.683333333 | 0.367361111 | 0 | 5 |
| ADD2 | 0 | 0.364868172095494 | 0.672222222 | 0.296527778 | 0 | 5 |
| AL353747.4 | 0 | 0.364590506686424 | 0.645138889 | 0.023 | 0 | 5 |
| VASH2 | 0 | 0.361524286579365 | 0.636805556 | 0.095 | 0 | 5 |
| HSP90AB1 | 0 | 0.361400578250213 | 1 | 1 | 0 | 5 |
| CCT3 | 0 | 0.361034172624082 | 1 | 0.69375 | 0 | 5 |
| NDUFS5 | 0 | 0.360778218202762 | 1 | 1 | 0 | 5 |
| NAXE | 0 | 0.359449974271545 | 0.690972222 | 0.415972222 | 0 | 5 |
| C1GALT1 | 0 | 0.359439273444812 | 0.684027778 | 0.311111111 | 0 | 5 |
| HTATIP2 | 0 | 0.356415049452092 | 0.669444444 | 0.155555556 | 0 | 5 |
| RNASEH2C | 0 | 0.356120825109285 | 1 | 0.047222222 | 0 | 5 |
| HMGA1 | 0 | 0.353837538014864 | 1 | 1 | 0 | 5 |
| MCRIP2 | 0 | 0.352936587763507 | 0.659027778 | 00:13 | 0 | 5 |
| PTGES3 | 0 | 0.352711754235586 | 1 | 1 | 0 | 5 |
| FOXO1 | 0 | 0.352524670185517 | 0.64375 | 0.082638889 | 0 | 5 |
| PPP2R2B | 0 | 0.352056027013648 | 0.683333333 | 0.325694444 | 0 | 5 |
| PARK7 | 0 | 0.351103526672306 | 1 | 0.69375 | 0 | 5 |
| AC068587.4 | 0 | 0.34838636725248 | 0.648611111 | 0.14375 | 0 | 5 |
| TLCD1 | 0 | 0.346120923341153 | 0.656944444 | 00:15 | 0 | 5 |
| BID | 0 | 0.344494415600434 | 0.679861111 | 0.274305556 | 0 | 5 |
| SPINT1 | 0 | 0.344289576651434 | 0.644444444 | 0.072 | 0 | 5 |
| SORBS2 | 0 | 0.340137022909802 | 0.613194444 | 0.163888889 | 0 | 5 |
| MTFP1 | 0 | 0.33975650329566 | 0.686111111 | 0.372916667 | 0 | 5 |
| PEPD | 0 | 0.339624975861116 | 0.691666667 | 0.400694444 | 0 | 5 |
| EIF4A1 | 0 | 0.339051386334051 | 1 | 1 | 0 | 5 |
| SLC29A1 | 0 | 0.338620275919392 | 0.68125 | 0.329861111 | 0 | 5 |
| RASGRP2 | 0 | 0.337954590915528 | 0.60625 | 0.047 | 0 | 5 |
| MFSD3 | 0 | 0.337919263920856 | 0.663888889 | 0.143055556 | 0 | 5 |
| KRTCAP3 | 0 | 0.337899642017862 | 0.658333333 | 0.090972222 | 0 | 5 |
| NQO1 | 0 | 0.335515880342292 | 0.65 | 0.146527778 | 0 | 5 |
| SERPINE2 | 0 | 0.334517113736789 | 0.672916667 | 0.265972222 | 0 | 5 |
| TRNP1 | 0 | 0.33366332469041 | 0.064583333 | 0.034 | 0 | 5 |
| TTF2 | 0 | 0.329191129763536 | 0.667361111 | 0.289583333 | 0 | 5 |
| CLDN10 | 0 | 0.327009142529572 | 0.068055556 | 0.285416667 | 0 | 5 |
| MYBL2 | 0 | 0.326457240355792 | 0.684027778 | 00:46 | 0 | 5 |

| ITPR2 | 0 | 0.326194887774771 | 0.58125 | 0.049 | 0 | 5 |
| --- | --- | --- | --- | --- | --- | --- |
| SMIM3 | 0 | 0.324902033463534 | 0.665972222 | 0.227083333 | 0 | 5 |
| COMTD1 | 0 | 0.323908427723682 | 0.63125 | 0.086 | 0 | 5 |
| PLAAT3 | 0 | 0.323225369552506 | 0.647916667 | 0.236805556 | 0 | 5 |
| BCL11A | 0 | 0.322950067524771 | 0.598611111 | 0.014 | 0 | 5 |
| RBP1 | 0 | 0.321293755062868 | 0.675 | 0.214583333 | 0 | 5 |
| GPR176 | 0 | 0.319562337575153 | 0.615972222 | 0.097 | 0 | 5 |
| UQCRH | 0 | 0.319261887636424 | 1 | 1 | 0 | 5 |
| AC092490.1 | 0 | 0.318868786311623 | 0.644444444 | 0.145138889 | 0 | 5 |
| ZNRD2 | 0 | 0.317959889825609 | 0.683333333 | 0.308333333 | 0 | 5 |
| TUBA4A | 0 | 0.317905869488408 | 0.620833333 | 0.021 | 0 | 5 |
| DUSP23 | 0 | 0.317384785160185 | 0.677777778 | 0.265277778 | 0 | 5 |
| DHCR7 | 0 | 0.316938275694226 | 0.648611111 | 0.204166667 | 0 | 5 |
| MARVELD3 | 0 | 0.314932785742514 | 0.611805556 | 0.021 | 0 | 5 |
| PTGES2 | 0 | 0.31355819895 | 0.676388889 | 0.283333333 | 0 | 5 |
| SNHG4 | 0 | 0.311336737382732 | 0.622222222 | 0.109027778 | 0 | 5 |
| FOXD3 | 0 | 0.310739746492433 | 0.588194444 | 0.014 | 0 | 5 |
| OAT | 0 | 0.310448165537672 | 0.679166667 | 0.335416667 | 0 | 5 |
| BOP1 | 0 | 0.306674150047505 | 0.664583333 | 0.219444444 | 0 | 5 |
| VSIG10 | 0 | 0.305023267839974 | 0.065277778 | 0.219444444 | 0 | 5 |
| C9orf135 | 0 | 0.302579412692851 | 0.596527778 | 0.019 | 0 | 5 |
| IER3 | 0 | 0.301062143972474 | 0.571527778 | 0.096527778 | 0 | 5 |
| PLA2G4C | 0 | 0.296313686389091 | 0.568055556 | 0.019 | 0 | 5 |
| PLA2G12A | 0 | 0.29510288563992 | 0.672222222 | 00:38 | 0 | 5 |
| EXOSC5 | 0 | 0.294593484738726 | 0.672916667 | 0.285416667 | 0 | 5 |
| NTS | 0 | 0.293373455222631 | 0.415972222 | 0.026 | 0 | 5 |
| SIGMAR1 | 0 | 0.293133961195132 | 0.672916667 | 0.280555556 | 0 | 5 |
| FXYD5 | 0 | 0.292653256004976 | 0.066666667 | 0.257638889 | 0 | 5 |
| NR3C1 | 0 | 0.292422199097421 | 0.573611111 | 0.053 | 0 | 5 |
| TSTA3 | 0 | 0.291894584810818 | 0.676388889 | 0.324305556 | 0 | 5 |
| PIM2 | 0 | 0.288986149864804 | 0.622916667 | 0.104861111 | 0 | 5 |
| NMRK2 | 0 | 0.28656524082783 | 0.060416667 | 0.045 | 0 | 5 |
| LINC01405 | 0 | 0.2849203137234 | 0.591666667 | 0.088 | 0 | 5 |
| COL6A1 | 0 | 0.282957809071333 | 0.650694444 | 00:37 | 0 | 5 |
| PHLDA1 | 0 | 0.28286587799149 | 0.052777778 | 0.059 | 0 | 5 |
| PCCA-DT | 0 | 0.281184003933455 | 0.650694444 | 0.191666667 | 0 | 5 |
| LINC01315 | 0 | 0.279015622588379 | 0.646527778 | 0.147916667 | 0 | 5 |
| RND3 | 0 | 0.275490490124224 | 0.531944444 | 0.095833333 | 0 | 5 |
| OLFM1 | 0 | 0.275140712215759 | 0.623611111 | 0.072222222 | 0 | 5 |
| LINC00545 | 0 | 0.274834684579806 | 0.594444444 | 0.022 | 0 | 5 |
| METTL8 | 0 | 0.272805249042624 | 0.659027778 | 0.235416667 | 0 | 5 |
| TENT5B | 0 | 0.270962955500767 | 0.620833333 | 0.072222222 | 0 | 5 |
| LCK | 0 | 0.270664088160799 | 0.581944444 | 0.019 | 0 | 5 |
| CDCA7 | 0 | 0.270619523735897 | 0.652083333 | 0.227777778 | 0 | 5 |
| PMEPA1 | 0 | 0.267964146162134 | 0.575 | 0.008 | 0 | 5 |
| INSIG1 | 0 | 0.266497916090854 | 0.55 | 00:14 | 0 | 5 |
| RAB25 | 0 | 0.265000521134657 | 0.632638889 | 0.10625 | 0 | 5 |
| SHC1 | 0 | 0.264171133610627 | 0.650694444 | 0.219444444 | 0 | 5 |
| SPRY4 | 0 | 0.261064635446826 | 0.565972222 | 00:11 | 0 | 5 |
| SMPDL3B | 0 | 0.260368747640967 | 0.594444444 | 0.028 | 0 | 5 |
| BAG2 | 0 | 0.260192618825827 | 0.6 | 00:13 | 0 | 5 |
| SOCS2 | 0 | 0.259363141124123 | 0.05625 | 0.071 | 0 | 5 |
| GRTP1 | 0 | 0.25854137574648 | 0.638194444 | 00:31 | 0 | 5 |
| SCNN1A | 0 | 0.258247108551413 | 0.579166667 | 0.025 | 0 | 5 |
| ENHO | 0 | 0.257552618072715 | 0.620833333 | 0.136111111 | 0 | 5 |
| TES | 0 | 0.257009275260252 | 0.584027778 | 0.071 | 0 | 5 |
| TDRP | 0 | 0.255214418784222 | 0.626388889 | 0.107638889 | 0 | 5 |
| ZC3HAV1 | 0 | 0.254410726120442 | 0.613194444 | 0.140972222 | 0 | 5 |
| FAM169A | 0 | 0.2542113113546 | 00:09 | 0.183333333 | 0 | 5 |
| FBXO2 | 0 | 0.253414527351051 | 0.613194444 | 0.10625 | 0 | 5 |
| ZIC5 | 0 | 0.252972610168946 | 0.621527778 | 0.195138889 | 0 | 5 |
| FUOM | 0 | 0.252923069675978 | 0.636805556 | 0.165972222 | 0 | 5 |
| GFPT2 | 0 | 0.252433464504266 | 0.616666667 | 0.155555556 | 0 | 5 |
| HAPLN3 | 0 | 0.252105998176469 | 0.6 | 0.072916667 | 0 | 5 |
| PCDH1 | 0 | 0.250932036387585 | 0.621527778 | 0.140277778 | 0 | 5 |
| FRAT2 | 4.28739381550613e-307 | 0.285277916254056 | 0.677777778 | 0.278472222 | 9.35938069924987e-303 | 5 |
| PGP | 1.77123602136104e-306 | 0.388343650177702 | 1 | 0.615972222 | 3.86660823463114e-302 | 5 |
| SPATS2L | 1.97398147084276e-306 | 0.354694956291456 | 0.065277778 | 0.297222222 | 4.30920155084974e-302 | 5 |
| ECI2 | 2.55845036810064e-306 | 0.365495373439865 | 0.690972222 | 0.477083333 | 5.58509715356371e-302 | 5 |
| SNRPD3 | 2.93539429838142e-306 | 0.34805371598075 | 1 | 0.679166667 | 6.40796575336664e-302 | 5 |
| CNTLN | 3.12954797032734e-306 | 0.265669905260902 | 0.652083333 | 0.254861111 | 6.83180321922459e-302 | 5 |
| HMGN5 | 1.30365789305155e-305 | 0.326945805422132 | 0.682638889 | 0.352083333 | 2.84588518053153e-301 | 5 |
| DDX21 | 2.61440619868366e-304 | 0.427348476181032 | 1 | 0.068055556 | 5.70724873172643e-300 | 5 |
| COX8A | 6.21514041499532e-304 | 0.341251928836066 | 1 | 0.692361111 | 1.35676515259348e-299 | 5 |
| FAM98A | 2.85154066681389e-300 | 0.356487867779358 | 0.688888889 | 0.464583333 | 6.22491327565473e-296 | 5 |
| YBX3 | 9.09778158585994e-300 | 0.393235328243549 | 1 | 0.68125 | 1.98604572019322e-295 | 5 |
| ANXA5 | 3.89018831023117e-299 | 0.402783501321015 | 1 | 0.065277778 | 8.49228108123464e-295 | 5 |
| AHCY | 9.05875894014545e-299 | 0.368449870191876 | 1 | 0.619444444 | 1.97752707663375e-294 | 5 |
| PSMD7 | 3.43431327465486e-298 | 0.354546299990822 | 1 | 0.686805556 | 7.49710587857157e-294 | 5 |
| PAGR1 | 7.04511951858366e-298 | 0.348669818408035 | 0.692361111 | 0.511111111 | 1.53794959090681e-293 | 5 |
| SELENOH | 1.22355981911627e-297 | 0.334314365820969 | 1 | 0.69375 | 2.67103108513082e-293 | 5 |
| SSNA1 | 3.43785749542404e-297 | 0.361915708180178 | 0.693055556 | 0.544444444 | 7.50484291251067e-293 | 5 |
| FKBP11 | 1.98402365175869e-296 | 0.260980421730339 | 0.66875 | 0.288194444 | 4.33112363178922e-292 | 5 |
| SNRPB | 2.60966181894412e-295 | 0.359522510957166 | 1 | 0.693055556 | 5.69689175075501e-291 | 5 |
| CMAS | 1.71240771570239e-294 | 0.396057879914547 | 0.69375 | 0.058333333 | 3.73818604337833e-290 | 5 |
| ATP5MC3 | 3.34980652684087e-294 | 0.35054112241928 | 1 | 1 | 7.31262764809361e-290 | 5 |
| FAM136A | 5.31816905607157e-294 | 0.3605350282752 | 1 | 0.665972222 | 1.16095630494042e-289 | 5 |
| BOLA3 | 1.01195271781662e-293 | 0.381853218229307 | 0.693055556 | 0.058333333 | 2.20909278299367e-289 | 5 |
| MTG1 | 1.02705742288264e-293 | 0.288066665544945 | 0.68125 | 0.349305556 | 2.24206635415281e-289 | 5 |
| PPP1CC | 6.10883188622904e-291 | 0.362644469740354 | 1 | 0.684027778 | 1.3335580007638e-286 | 5 |
| TRAPPC5 | 8.51110476409574e-291 | 0.355484970327784 | 1 | 0.642361111 | 1.8579741700021e-286 | 5 |

| HSPA8 | 2.46663657176149e-290 | 0.498221427840468 | 1 | 0.693055556 | 5.38466763615533e-286 | 5 |
| --- | --- | --- | --- | --- | --- | --- |
| FAM207A | 1.16450615818512e-289 | 0.314405702234321 | 0.690277778 | 0.40625 | 2.54211694331811e-285 | 5 |
| NDUFB10 | 2.36979523591587e-289 | 0.340249170036514 | 1 | 0.690277778 | 5.17326300000435e-285 | 5 |
| HSP90B1 | 3.30075536187979e-289 | 0.559194142956766 | 1 | 0.688194444 | 7.20554895498359e-285 | 5 |
| GPI | 5.08517183522359e-289 | 0.369161500608875 | 0.69375 | 0.054861111 | 1.11009301162931e-284 | 5 |
| MDH1 | 5.85096134786117e-289 | 0.379244557362201 | 1 | 0.682638889 | 1.27726486223809e-284 | 5 |
| JADE1 | 1.79225749746363e-288 | 0.453409284296448 | 0.692361111 | 0.523611111 | 3.9124981169631e-284 | 5 |
| SNHG14 | 2.48348978076836e-288 | 0.693509269855142 | 1 | 0.677777778 | 5.42145819141733e-284 | 5 |
| IARS | 4.30191208471745e-288 | 0.381685252100105 | 0.690277778 | 0.486805556 | 9.39107408093819e-284 | 5 |
| SLC1A5 | 1.36280688757811e-287 | 0.39520500739031 | 0.692361111 | 0.485416667 | 2.97500743558302e-283 | 5 |
| FBL | 1.61822150972487e-287 | 0.361979099729642 | 1 | 0.067361111 | 3.5325775557294e-283 | 5 |
| MDH2 | 2.27858768047513e-287 | 0.393107422896191 | 1 | 0.68125 | 4.9741569064772e-283 | 5 |
| CENPW | 5.0102879870426e-286 | 0.389403149511906 | 1 | 0.498611111 | 1.0937458675714e-281 | 5 |
| UTP20 | 4.47288279956896e-285 | 0.277189192856011 | 0.064583333 | 0.279166667 | 9.76430315145903e-281 | 5 |
| WFDC2 | 7.55323006820291e-285 | 0.349284323990704 | 0.068055556 | 0.347916667 | 1.6488701238887e-280 | 5 |
| AP1S2 | 1.13338232372406e-284 | 0.416129305075808 | 1 | 0.681944444 | 2.47417361268962e-280 | 5 |
| PSMD14 | 1.13443738556201e-284 | 0.355988570784416 | 1 | 0.676388889 | 2.47647681268187e-280 | 5 |
| ZMYND8 | 3.01506866801328e-284 | 0.423040020902244 | 0.689583333 | 0.508333333 | 6.58189490227299e-280 | 5 |
| PDCL3 | 1.64256501560474e-283 | 0.286554686673658 | 0.68125 | 0.379861111 | 3.58571942906515e-279 | 5 |
| HESX1 | 1.72332260061124e-283 | 0.253299704229095 | 0.63125 | 0.216666667 | 3.76201323713434e-279 | 5 |
| THOC6 | 6.3258988891476e-283 | 0.268873584923376 | 0.677777778 | 0.327777778 | 1.38094372750092e-278 | 5 |
| HNRNPA3 | 2.18762195508514e-282 | 0.412805238658323 | 1 | 0.69375 | 4.77557872795086e-278 | 5 |
| AP2M1 | 1.18112914138759e-280 | 0.33356992010643 | 1 | 0.683333333 | 2.57840491564912e-276 | 5 |
| PCBP1 | 1.61221001516232e-279 | 0.339623368979498 | 1 | 0.68125 | 3.51945446309935e-275 | 5 |
| SRSF7 | 2.4642474027795e-279 | 0.356365465998976 | 1 | 1 | 5.37945208026764e-275 | 5 |
| MRPL20 | 1.61180473454758e-278 | 0.340625114039333 | 1 | 0.684722222 | 3.51856973551737e-274 | 5 |
| ATIC | 7.09106350403242e-277 | 0.349081742396712 | 0.693055556 | 0.563888889 | 1.54797916293028e-272 | 5 |
| AKAP1 | 7.45641275862193e-277 | 0.257060949510753 | 0.633333333 | 0.252083333 | 1.62773490520717e-272 | 5 |
| GCSH | 1.4930512617301e-276 | 0.315005253587869 | 1 | 0.691666667 | 3.2593309043568e-272 | 5 |
| PGM2L1 | 1.12938246717011e-275 | 0.2531509418432 | 0.654861111 | 0.272916667 | 2.46544192583235e-271 | 5 |
| CALR | 1.98001653631054e-275 | 0.434700499902309 | 1 | 0.690277778 | 4.32237609876591e-271 | 5 |
| EGFL7 | 2.42115945591889e-274 | 0.25068029640402 | 0.663888889 | 0.295833333 | 5.28539109227094e-270 | 5 |
| SRI | 4.05263751793594e-274 | 0.35725063574501 | 1 | 0.668055556 | 8.84690770165415e-270 | 5 |
| SC5D | 4.91614099372076e-273 | 0.330020716475311 | 0.068055556 | 0.392361111 | 1.07319357892924e-268 | 5 |
| POLR2F | 2.271565541353e-272 | 0.342236019767456 | 1 | 0.659027778 | 4.95882757677359e-268 | 5 |
| RDH11 | 6.84308799728669e-272 | 0.348336620952667 | 0.69375 | 0.545833333 | 1.49384610980768e-267 | 5 |
| GYPC | 2.08788581360392e-270 | 0.258293723515382 | 0.661111111 | 0.289583333 | 4.55785473109736e-266 | 5 |
| SLC38A1 | 2.83335654639766e-270 | 0.448137415114891 | 1 | 0.624305556 | 6.18521734078609e-266 | 5 |
| F11R | 4.48585034990317e-270 | 0.315056050112597 | 0.68125 | 0.402083333 | 9.79261131383862e-266 | 5 |
| RMI2 | 4.26616827368644e-269 | 0.279215551689687 | 0.68125 | 0.336111111 | 9.3130453414575e-265 | 5 |
| CCT6A | 9.5375881798458e-269 | 0.295493753801305 | 1 | 1 | 2.08205549966034e-264 | 5 |
| CENPX | 1.12895761310542e-268 | 0.352953541649029 | 1 | 0.627083333 | 2.46451446940914e-264 | 5 |
| PSMB4 | 3.58140149954337e-268 | 0.319795163407214 | 1 | 0.661111111 | 7.81819947350317e-264 | 5 |
| MPP6 | 1.87029782800986e-267 | 0.307684705583109 | 0.682638889 | 0.39375 | 4.08286015854553e-263 | 5 |
| PIH1D1 | 4.63169540329096e-267 | 0.330057229034138 | 0.692361111 | 0.545138889 | 1.01109910653842e-262 | 5 |
| MSMO1 | 3.17797778348252e-266 | 0.470475410593867 | 0.684027778 | 0.470138889 | 6.93752550134234e-262 | 5 |
| EIF2S2 | 6.70805871933891e-266 | 0.366442728130856 | 1 | 0.692361111 | 1.46436921843169e-261 | 5 |
| AGTRAP | 1.01717426723018e-265 | 0.302571181693101 | 0.689583333 | 0.044444444 | 2.22049142536349e-261 | 5 |
| IER2 | 1.41225050702043e-265 | 0.437916211589812 | 0.69375 | 0.570833333 | 3.08294285682559e-261 | 5 |
| SLTM | 3.81613752761545e-265 | 0.411923629845719 | 1 | 0.648611111 | 8.33062822278453e-261 | 5 |
| HNRNPM | 3.85876742200652e-265 | 0.352612927770441 | 1 | 0.69375 | 8.42368928224024e-261 | 5 |
| SERF2 | 6.99157337671343e-264 | 0.257546404242808 | 1 | 1 | 1.52626046813654e-259 | 5 |
| RPL39L | 5.41145336662779e-263 | 0.373982023715393 | 0.692361111 | 0.525 | 1.18132026993485e-258 | 5 |
| ANOS1 | 5.74016513886375e-262 | 0.349563862656243 | 0.686805556 | 0.471527778 | 1.25307804981396e-257 | 5 |
| TXLNG | 3.95006663828492e-261 | 0.33299510056145 | 0.691666667 | 0.525694444 | 8.62299547137598e-257 | 5 |
| PSMB5 | 5.03666666163233e-261 | 0.32103876526413 | 1 | 0.691666667 | 1.09950433223434e-256 | 5 |
| CTSV | 1.05748120600836e-260 | 0.312924020757109 | 0.06875 | 0.413888889 | 2.30848147271625e-256 | 5 |
| RAC1 | 1.71874808363433e-260 | 0.272088549475795 | 1 | 1 | 3.75202706657375e-256 | 5 |
| DCUN1D5 | 4.38661781349961e-260 | 0.335906265082762 | 1 | 0.672222222 | 9.57598668686964e-256 | 5 |
| FAM162A | 1.35724906135591e-259 | 0.406719961489722 | 0.69375 | 0.55625 | 2.96287470093996e-255 | 5 |
| CNDP2 | 2.45298408523801e-259 | 0.310625763497576 | 1 | 0.502083333 | 5.35486425807457e-255 | 5 |
| MFSD10 | 5.95957394024281e-258 | 0.311797220825088 | 0.690972222 | 0.473611111 | 1.30097499115501e-253 | 5 |
| NDUFB9 | 9.50498459195163e-258 | 0.324173651596454 | 1 | 0.684722222 | 2.07493813642304e-253 | 5 |
| PSME2 | 9.73545755729535e-258 | 0.325356904873571 | 1 | 0.538194444 | 2.12525038475757e-253 | 5 |
| MLEC | 3.43928781618368e-257 | 0.358785479771659 | 1 | 0.676388889 | 7.50796530272897e-253 | 5 |
| PPP1CA | 2.41544909715997e-256 | 0.322802030746658 | 1 | 0.633333333 | 5.27292537910022e-252 | 5 |
| SNHG3 | 3.31135095075362e-255 | 0.282096601832583 | 0.669444444 | 0.320138889 | 7.22867912549516e-251 | 5 |
| ATP5F1B | 1.88983762158742e-254 | 0.320898004556096 | 1 | 0.69375 | 4.12551552792533e-250 | 5 |
| PSME3 | 4.12092304227895e-254 | 0.2770401819299 | 0.676388889 | 0.415277778 | 8.99597500129494e-250 | 5 |
| COMMD4 | 4.4420219203526e-254 | 0.311061077955936 | 0.692361111 | 0.522916667 | 9.69693385212973e-250 | 5 |
| ZBTB44 | 3.57620016895476e-253 | 0.318825184188812 | 0.672916667 | 00:57 | 7.80684496882824e-249 | 5 |
| PMF1 | 1.59979696128841e-251 | 0.319292522494328 | 1 | 0.524305556 | 3.4923567664926e-247 | 5 |
| UBE2L3 | 2.08440178626277e-251 | 0.305322219789064 | 1 | 0.066666667 | 4.55024909941163e-247 | 5 |
| ATP5IF1 | 2.7328719190854e-251 | 0.305655398946797 | 1 | 0.69375 | 5.96585939936342e-247 | 5 |
| SAP18 | 1.64650975426164e-250 | 0.320218755933888 | 1 | 0.69375 | 3.59433079355317e-246 | 5 |
| ATP5PD | 1.37027711865264e-249 | 0.3253132793925 | 1 | 0.682638889 | 2.9913149500187e-245 | 5 |
| PPA1 | 1.67703134805011e-249 | 0.338212208314528 | 1 | 0.068055556 | 3.66095943279339e-245 | 5 |
| PSMG4 | 2.73165746136462e-249 | 0.298175601364724 | 0.684027778 | 0.415972222 | 5.96320823815896e-245 | 5 |
| SERP1 | 4.10158268263241e-249 | 0.316043746142546 | 0.69375 | 0.663888889 | 8.95375499618656e-245 | 5 |
| PSMB1 | 6.17547282653712e-249 | 0.301465698566957 | 1 | 1 | 1.34810571803305e-244 | 5 |
| HDDC2 | 1.14106646488526e-248 | 0.330281656168797 | 1 | 0.60625 | 2.49094809284452e-244 | 5 |
| MRPL27 | 1.54081250267671e-248 | 0.312837358197224 | 1 | 0.551388889 | 3.36359369334327e-244 | 5 |
| GSPT1 | 2.06674269823469e-248 | 0.332963202673055 | 1 | 0.663888889 | 4.51169931024632e-244 | 5 |
| NME3 | 4.34875723168412e-248 | 0.317602705373353 | 0.692361111 | 0.495833333 | 9.49333703676643e-244 | 5 |
| TPM2 | 6.76612403148013e-248 | 0.398271623938664 | 1 | 0.058333333 | 1.47704487607211e-243 | 5 |
| NOP56 | 1.5252661426171e-247 | 0.345798916594549 | 1 | 0.657638889 | 3.32965598933314e-243 | 5 |
| SNF8 | 2.00624341451228e-247 | 0.312172302229253 | 1 | 0.563888889 | 4.37962937388031e-243 | 5 |
| GDI2 | 2.18624346534377e-247 | 0.312756485842041 | 1 | 0.06875 | 4.77256948484544e-243 | 5 |
| UQCRC1 | 4.09008990682086e-247 | 0.310620312994229 | 0.69375 | 0.55 | 8.92866626658995e-243 | 5 |
| DNAJC7 | 5.98672613687247e-247 | 0.328857215696121 | 1 | 0.64375 | 1.30690231567926e-242 | 5 |
| POLR2H | 1.45525604823088e-246 | 0.322594742400636 | 0.69375 | 0.609027778 | 3.17682395328801e-242 | 5 |

| GET3 | 1.52142477071661e-245 | 0.286510615594249 | 0.690972222 | 0.475 | 3.32127027447436e-241 | 5 |
| --- | --- | --- | --- | --- | --- | --- |
| SLC44A1 | 1.78108932490534e-245 | 0.290195272117123 | 0.671527778 | 0.370138889 | 3.88811799626835e-241 | 5 |
| SNRNP25 | 9.39340649765952e-245 | 0.313322400514961 | 0.69375 | 0.558333333 | 2.05058063843907e-240 | 5 |
| EIF3B | 1.01522149530842e-243 | 0.32187674382877 | 1 | 0.641666667 | 2.21622852425828e-239 | 5 |
| BAX | 1.34213274022175e-243 | 0.433468499985191 | 1 | 0.620138889 | 2.92987577190407e-239 | 5 |
| MRPL4 | 1.55628758018072e-243 | 0.2978467239259 | 0.690972222 | 0.486805556 | 3.39737578753452e-239 | 5 |
| PRDX4 | 1.31552671567506e-241 | 0.42099686623653 | 1 | 0.681944444 | 2.87179482031865e-237 | 5 |
| ZFP36L2 | 1.89335962864762e-241 | 0.334977653011777 | 0.06875 | 00:56 | 4.13320406933776e-237 | 5 |
| LINC00665 | 7.38456708456915e-241 | 0.316041458200514 | 0.691666667 | 0.574305556 | 1.61205099456145e-236 | 5 |
| SOD1 | 1.33908379251855e-240 | 0.329735799548156 | 1 | 0.688194444 | 2.92321991906799e-236 | 5 |
| NOP16 | 1.6718224238139e-240 | 0.311084012584875 | 0.693055556 | 0.484722222 | 3.64958835118575e-236 | 5 |
| CACYBP | 1.78818711739471e-240 | 0.311592921220611 | 1 | 0.684722222 | 3.90361247727264e-236 | 5 |
| CDC6 | 3.03548016288229e-240 | 0.33871311414674 | 0.689583333 | 0.36875 | 6.62645319557204e-236 | 5 |
| MRPL52 | 9.73815741945967e-240 | 0.311577009554714 | 1 | 0.682638889 | 2.12583976466805e-235 | 5 |
| LPCAT1 | 1.75373228967638e-239 | 0.266547004701471 | 0.670138889 | 0.375694444 | 3.82839758836353e-235 | 5 |
| EBPL | 4.9378747428811e-239 | 0.314935732803389 | 1 | 0.628472222 | 1.07793805637095e-234 | 5 |
| G3BP1 | 5.19648876411303e-239 | 0.334069496175571 | 1 | 0.684722222 | 1.13439349720588e-234 | 5 |
| DCAF13 | 9.61750461385828e-239 | 0.316918535879229 | 1 | 0.655555556 | 2.09950125720526e-234 | 5 |
| CDC123 | 4.65033187679791e-238 | 0.30719027580055 | 1 | 0.065972222 | 1.01516744870498e-233 | 5 |
| SNRPA1 | 4.68496995420278e-238 | 0.308354550361711 | 1 | 0.674305556 | 1.02272894100247e-233 | 5 |
| POLR2I | 3.70155109373206e-237 | 0.312589638019712 | 1 | 0.677083333 | 8.0804860376171e-233 | 5 |
| NDUFAF4 | 5.11124763881906e-237 | 0.296520209463887 | 0.688888889 | 0.470833333 | 1.1157853595542e-232 | 5 |
| MRPL11 | 3.77608737634958e-235 | 0.313180310478492 | 0.69375 | 0.668055556 | 8.24319874257113e-231 | 5 |
| RBM3 | 6.31681297365034e-235 | 0.313991177824854 | 1 | 0.664583333 | 1.37896027214787e-230 | 5 |
| TMEM160 | 7.55035484611672e-235 | 0.317162781099534 | 0.69375 | 0.061111111 | 1.64824246290728e-230 | 5 |
| PDXK | 1.12358393568959e-234 | 0.261942196033201 | 0.06875 | 0.409027778 | 2.45278373161036e-230 | 5 |
| UNG | 2.87825712117725e-234 | 0.36115368103627 | 0.693055556 | 0.482638889 | 6.28323529552994e-230 | 5 |
| SNRPF | 2.93578798607573e-234 | 0.256805948225048 | 1 | 1 | 6.40882517360332e-230 | 5 |
| TOMM7 | 1.79874839277032e-233 | 0.290580509720661 | 1 | 1 | 3.92666774141761e-229 | 5 |
| TCEAL8 | 3.08942779262867e-233 | 0.329566447389144 | 1 | 0.657638889 | 6.74422087130838e-229 | 5 |
| PSME1 | 4.80554168922865e-233 | 0.274110915872824 | 0.685416667 | 0.414583333 | 1.04904975075861e-228 | 5 |
| VCAN | 2.04864146356932e-232 | 0.398963521221009 | 0.068055556 | 0.433333333 | 4.47218431497183e-228 | 5 |
| LRRFIP1 | 7.73363443511296e-232 | 0.289427562995301 | 0.685416667 | 0.431944444 | 1.68825239718516e-227 | 5 |
| POLR3K | 1.01676247794878e-231 | 0.307192638624573 | 0.693055556 | 0.543055556 | 2.21959248936219e-227 | 5 |
| DNAJC15 | 1.19029016199226e-231 | 0.336238554979007 | 0.693055556 | 0.455555556 | 2.5984034236291e-227 | 5 |
| NDUFAF8 | 1.01008916775445e-229 | 0.312193347276906 | 1 | 0.05625 | 2.20502465320797e-225 | 5 |
| CHCHD1 | 1.24218077544353e-229 | 0.291979473362325 | 1 | 0.661111111 | 2.71168063279322e-225 | 5 |
| MRPS24 | 6.65522075679282e-228 | 0.300836633395656 | 1 | 0.645138889 | 1.45283469120787e-223 | 5 |
| IMP3 | 9.74513917590292e-228 | 0.312799498383886 | 0.69375 | 0.648611111 | 2.12736388209961e-223 | 5 |
| HNRNPAB | 2.17677630691075e-226 | 0.298129938493323 | 1 | 1 | 4.75190267798617e-222 | 5 |
| CHCHD3 | 1.36316746437359e-225 | 0.292980687732272 | 0.693055556 | 0.599305556 | 2.97579457472755e-221 | 5 |
| ISCA1 | 2.25340054830538e-225 | 0.26691762316229 | 0.06875 | 0.434722222 | 4.91917339695064e-221 | 5 |
| PSMB6 | 1.88052593419393e-223 | 0.286998942189328 | 1 | 0.692361111 | 4.10518811434534e-219 | 5 |
| LSM3 | 2.96351266245611e-223 | 0.292957181206015 | 1 | 0.683333333 | 6.46934814214169e-219 | 5 |
| TUBB2A | 3.92128684868282e-223 | 0.329597100046766 | 0.690277778 | 0.46875 | 8.56016919067459e-219 | 5 |
| SSB | 1.14548308043676e-222 | 0.251202991170808 | 1 | 1 | 2.50058956459345e-218 | 5 |
| COX7B | 1.84675372607602e-222 | 0.298669630070143 | 1 | 0.692361111 | 4.03146338402396e-218 | 5 |
| CCT2 | 2.51855909833372e-221 | 0.259081636016432 | 1 | 0.69375 | 5.49801451166251e-217 | 5 |
| LAGE3 | 2.6603088674875e-221 | 0.313629932312337 | 0.690972222 | 0.5375 | 5.80745425772522e-217 | 5 |
| EIF4G1 | 4.1028553032538e-220 | 0.354876798460752 | 0.689583333 | 0.557638889 | 8.95653312700304e-216 | 5 |
| CHRAC1 | 1.01978268434264e-219 | 0.252420364143573 | 0.679861111 | 0.390277778 | 2.22618559991998e-215 | 5 |
| COX20 | 3.64916211704148e-219 | 0.317284277298689 | 0.693055556 | 0.5875 | 7.96612090150155e-215 | 5 |
| PSMC4 | 4.24985546621051e-219 | 0.290153644240211 | 0.69375 | 0.609027778 | 9.27743448273755e-215 | 5 |
| HDAC2 | 1.98800516047567e-218 | 0.286750893401991 | 1 | 0.691666667 | 4.33981526531839e-214 | 5 |
| PSMC5 | 5.04692755405151e-217 | 0.288376017064783 | 1 | 0.665972222 | 1.10174428504944e-212 | 5 |
| SEC61B | 1.39263678973898e-216 | 0.25945262922597 | 1 | 0.69375 | 3.0401261120002e-212 | 5 |
| TUFM | 2.03362261741267e-216 | 0.302351735055279 | 1 | 0.672222222 | 4.43939817381186e-212 | 5 |
| VCP | 6.04063031598963e-216 | 0.289463339999 | 1 | 0.672222222 | 1.31866959798054e-211 | 5 |
| ENY2 | 1.12284991441587e-215 | 0.28356645405835 | 1 | 0.692361111 | 2.45118136316985e-211 | 5 |
| FIBP | 1.18463116206158e-215 | 0.28037324832198 | 0.691666667 | 0.533333333 | 2.58604982678043e-211 | 5 |
| CDYL | 1.27553302762894e-215 | 0.280501962548072 | 0.684027778 | 0.4625 | 2.78448859931398e-211 | 5 |
| PSMB3 | 9.36406691728558e-215 | 0.313323812855505 | 1 | 0.682638889 | 2.04417580804344e-210 | 5 |
| DKC1 | 9.58012001724205e-214 | 0.31002922983274 | 1 | 0.646527778 | 2.09134019976394e-209 | 5 |
| GALNT7 | 4.57621815768558e-213 | 0.285776300091943 | 0.682638889 | 0.448611111 | 9.98988423822762e-209 | 5 |
| METTL26 | 1.75840805157815e-212 | 0.298252082581412 | 0.69375 | 0.611805556 | 3.8386047765951e-208 | 5 |
| NPM3 | 2.22246140944076e-212 | 0.2836964373783 | 0.690972222 | 0.536805556 | 4.85163325680919e-208 | 5 |
| GLRX3 | 7.07810394060234e-212 | 0.284203573783845 | 0.69375 | 0.63125 | 1.54515009023349e-207 | 5 |
| GAPDH | 4.35782860403739e-211 | 0.329792964290389 | 1 | 1 | 9.51313984261362e-207 | 5 |
| MCM4 | 1.17127824945951e-210 | 0.383606340117991 | 0.693055556 | 0.489583333 | 2.55690041857012e-206 | 5 |
| SPTBN1 | 3.55316841923955e-210 | 0.392324062754755 | 0.684722222 | 0.495138889 | 7.75656665919993e-206 | 5 |
| ARF3 | 6.46007193544188e-210 | 0.275907165477651 | 0.693055556 | 0.538194444 | 1.41023370350696e-205 | 5 |
| TLE5 | 7.48544364251862e-210 | 0.296993392491958 | 0.69375 | 0.059027778 | 1.63407234716182e-205 | 5 |
| YIF1A | 2.14916583976309e-209 | 0.261325108060015 | 0.688888889 | 0.473611111 | 4.69162902820283e-205 | 5 |
| GTF3A | 1.27841656395783e-208 | 0.279299691065532 | 1 | 0.552083333 | 2.79078335911995e-204 | 5 |
| MGST3 | 2.23746793221114e-208 | 0.307226562567636 | 1 | 0.675 | 4.88439249601692e-204 | 5 |
| DDX46 | 9.88781879680708e-208 | 0.312237392877063 | 1 | 0.675694444 | 2.15851084334299e-203 | 5 |
| CDKN3 | 2.07042666229187e-205 | 0.296987517563476 | 0.691666667 | 00:59 | 4.51974140378316e-201 | 5 |
| DIAPH2 | 4.09307275120039e-205 | 0.317468737342311 | 0.066666667 | 0.407638889 | 8.93517781587045e-201 | 5 |
| CAPZB | 2.59650402868101e-204 | 0.284836188681414 | 1 | 0.679861111 | 5.66816829461064e-200 | 5 |
| REEP5 | 6.59785718057583e-204 | 0.259166075366206 | 0.688194444 | 0.480555556 | 1.4403122225197e-199 | 5 |
| DNMT3A | 3.32773120574858e-202 | 0.294542712934073 | 0.679861111 | 0.4625 | 7.26443722214916e-198 | 5 |
| EBNA1BP2 | 3.78701319112096e-202 | 0.266908207501435 | 0.692361111 | 0.55625 | 8.26704979621705e-198 | 5 |
| ZNF770 | 1.52562630478926e-201 | 0.318148701371366 | 0.690277778 | 0.565277778 | 3.33044222335496e-197 | 5 |
| ALYREF | 1.90135922626963e-201 | 0.286236043980857 | 0.693055556 | 0.6 | 4.1506671909466e-197 | 5 |
| YWHAQ | 2.04092130837964e-201 | 0.251169414520254 | 1 | 0.69375 | 4.45533121619275e-197 | 5 |
| GART | 2.54981311176815e-201 | 0.281734120564227 | 0.69375 | 0.565277778 | 5.56624202298986e-197 | 5 |
| MRPS26 | 6.15020693556257e-200 | 0.281114147125139 | 0.69375 | 0.661111111 | 1.34259017403331e-195 | 5 |
| CNPY2 | 1.14181036343369e-199 | 0.269495301124732 | 1 | 0.681944444 | 2.49257202337574e-195 | 5 |
| PHGDH | 1.49593089436822e-199 | 0.348176669336148 | 1 | 0.663194444 | 3.26561714240582e-195 | 5 |
| PFDN4 | 2.9744246822568e-199 | 0.270912283449867 | 1 | 0.665972222 | 6.49316908136659e-195 | 5 |
| ADI1 | 1.19470190254564e-197 | 0.261984632437555 | 0.688888889 | 0.453472222 | 2.60803425325714e-193 | 5 |

| OAZ1 | 2.45613010485057e-197 | 0.25452949174919 | 1 | 1 | 5.36173201888879e-193 | 5 |
| --- | --- | --- | --- | --- | --- | --- |
| DESI2 | 4.01526376869861e-196 | 0.290602337186573 | 1 | 0.640277778 | 8.76532080706907e-192 | 5 |
| PRPS1 | 5.31053659275452e-195 | 0.270783514795707 | 0.69375 | 0.620833333 | 1.15929013819831e-190 | 5 |
| CYC1 | 1.13891510906754e-194 | 0.288823032199647 | 1 | 0.625694444 | 2.48625168309444e-190 | 5 |
| TRIM28 | 2.44789135328903e-194 | 0.267889972992499 | 1 | 0.667361111 | 5.34374682422995e-190 | 5 |
| MZT2A | 3.75264942023372e-194 | 0.271897243056879 | 1 | 0.065972222 | 8.19203368437021e-190 | 5 |
| PRKCI | 5.22479772401754e-194 | 0.284482911426532 | 0.688888889 | 0.532638889 | 1.14057334315303e-189 | 5 |
| FKBP3 | 6.76690815223341e-194 | 0.25811567793459 | 1 | 0.691666667 | 1.47721604963255e-189 | 5 |
| CISD1 | 2.64755520682189e-193 | 0.264161294199846 | 0.69375 | 0.539583333 | 5.77961301649218e-189 | 5 |
| NAE1 | 5.38654643024495e-193 | 0.269498462143226 | 1 | 0.675 | 1.17588308572247e-188 | 5 |
| MRPL17 | 9.63186385333622e-193 | 0.269620224004536 | 0.693055556 | 0.056944444 | 2.1026358791833e-188 | 5 |
| SIVA1 | 1.87493504616728e-192 | 0.272836753717843 | 1 | 0.681944444 | 4.09298320578318e-188 | 5 |
| TMSB4X | 1.91831445586905e-192 | 0.386119959475808 | 1 | 1 | 4.18768045716215e-188 | 5 |
| UBE2N | 2.67003036324919e-192 | 0.264276571308075 | 1 | 0.676388889 | 5.82867628297299e-188 | 5 |
| PSMB2 | 2.84407076802557e-192 | 0.274754761275932 | 1 | 0.685416667 | 6.20860648659982e-188 | 5 |
| FKBP1A | 3.80001094371273e-192 | 0.269832554283625 | 1 | 0.685416667 | 8.29542389012489e-188 | 5 |
| FADS2 | 7.29945911000364e-192 | 0.267315981574818 | 0.671527778 | 0.042361111 | 1.5934719237138e-187 | 5 |
| BEX1 | 7.87434780387756e-192 | 0.326609291880365 | 1 | 0.5125 | 1.71897012558647e-187 | 5 |
| PAK1IP1 | 1.7597501933562e-191 | 0.281233046945398 | 0.692361111 | 0.584722222 | 3.84153467209658e-187 | 5 |
| TCP1 | 2.41045504575528e-191 | 0.265076093402229 | 1 | 0.677777778 | 5.26202336488377e-187 | 5 |
| JTB | 7.69927648429075e-191 | 0.262662537221598 | 1 | 0.632638889 | 1.68075205652067e-186 | 5 |
| MRPL36 | 2.47071714855421e-190 | 0.262620145744337 | 0.69375 | 0.592361111 | 5.39357553529384e-186 | 5 |
| SEMA6A | 4.86576609349374e-190 | 0.382220276518406 | 0.692361111 | 0.05625 | 1.06219673820968e-185 | 5 |
| LYPLA1 | 5.99447974830477e-190 | 0.261905540383772 | 0.69375 | 0.640972222 | 1.30859492905493e-185 | 5 |
| PSMD2 | 1.41595790996305e-189 | 0.259285786877916 | 1 | 0.675694444 | 3.09103611744933e-185 | 5 |
| CTSD | 2.17842975453241e-189 | 0.250331627675282 | 0.682638889 | 0.459722222 | 4.75551215414424e-185 | 5 |
| RTN4 | 3.13884496708555e-189 | 0.261697608551543 | 0.69375 | 0.691666667 | 6.85209856314776e-185 | 5 |
| MSN | 3.04172134027549e-188 | 0.262237010125015 | 0.690277778 | 0.50625 | 6.64007768582139e-184 | 5 |
| GLRX5 | 5.94551919952421e-188 | 0.257618456298005 | 1 | 0.06875 | 1.29790684125613e-183 | 5 |
| EI24 | 7.23757568032487e-188 | 0.276369626034561 | 0.69375 | 0.653472222 | 1.57996277101492e-183 | 5 |
| THYN1 | 1.37310662101813e-187 | 0.267203169812646 | 0.692361111 | 0.596527778 | 2.99749175368258e-183 | 5 |
| NUDT1 | 7.09315792136146e-187 | 0.273963714051007 | 1 | 0.6125 | 1.54843637423321e-182 | 5 |
| PAWR | 1.19231362921653e-186 | 0.314511952422559 | 0.69375 | 0.063194444 | 2.60282065257969e-182 | 5 |
| UFD1 | 1.76652562926872e-186 | 0.255097378713399 | 0.688194444 | 0.514583333 | 3.85632544869361e-182 | 5 |
| NAA10 | 2.0703553516121e-186 | 0.261939860001178 | 0.69375 | 0.588888889 | 4.51958573256921e-182 | 5 |
| LARP1 | 1.59181678166159e-184 | 0.307689789834543 | 0.689583333 | 0.577777778 | 3.47493603436726e-180 | 5 |
| REX1BD | 6.7774123414065e-184 | 0.267173963915582 | 0.69375 | 0.599305556 | 1.47950911412904e-179 | 5 |
| EIF3K | 1.11596608491266e-183 | 0.253248743542499 | 1 | 0.69375 | 2.43615396336434e-179 | 5 |
| GOT2 | 1.09703627288778e-182 | 0.265564462056322 | 0.690277778 | 0.511111111 | 2.39483018371402e-178 | 5 |
| RPS19BP1 | 5.06787239193793e-182 | 0.257124198907195 | 1 | 0.651388889 | 1.10631654316005e-177 | 5 |
| XPOT | 1.31594718171646e-181 | 0.251721610806997 | 0.670833333 | 0.427083333 | 2.87271269768703e-177 | 5 |
| HSPA5 | 2.37629746870858e-181 | 0.392021083984716 | 1 | 0.685416667 | 5.18745737419082e-177 | 5 |
| HSPA4 | 1.15381273788477e-180 | 0.307588084827974 | 1 | 0.68125 | 2.51877320680245e-176 | 5 |
| RHEB | 1.52523620758238e-179 | 0.264402498113774 | 1 | 0.674305556 | 3.32959064115234e-175 | 5 |
| ADRM1 | 4.25089278941456e-179 | 0.273137774809994 | 1 | 0.646527778 | 9.27969895929198e-175 | 5 |
| PCNA | 1.82162338881289e-178 | 0.300343286611297 | 0.693055556 | 0.542361111 | 3.97660385777854e-174 | 5 |
| ACSL3 | 3.51807776939692e-178 | 0.292678927337281 | 0.692361111 | 0.559722222 | 7.67996377059347e-174 | 5 |
| FOXN3 | 4.16810279110991e-178 | 0.26640958646529 | 0.683333333 | 0.48125 | 9.09896839299294e-174 | 5 |
| SF3A3 | 6.01364482557042e-178 | 0.264849820174269 | 0.693055556 | 0.647222222 | 1.31277866542202e-173 | 5 |
| DYNLL1 | 8.39779372891945e-178 | 0.304357126284652 | 1 | 1 | 1.83323837102312e-173 | 5 |
| CASP3 | 2.06756778993497e-177 | 0.266813790497923 | 0.686805556 | 0.519444444 | 4.51350048542805e-173 | 5 |
| PAM16 | 7.50222456872005e-177 | 0.254108050696996 | 1 | 0.567361111 | 1.63773562335159e-172 | 5 |
| PCLAF | 1.18488755684824e-176 | 0.318719482746621 | 1 | 0.575694444 | 2.58660953659972e-172 | 5 |
| MRPS12 | 1.45912495326685e-176 | 0.251679183765661 | 0.690972222 | 0.593055556 | 3.18526977298154e-172 | 5 |
| PTBP3 | 2.19137287422715e-175 | 0.305938064741248 | 0.688888889 | 0.543055556 | 4.78376698443786e-171 | 5 |
| SS18L2 | 2.95527303474596e-175 | 0.272622259114891 | 1 | 0.61875 | 6.45136103485044e-171 | 5 |
| HLA-A | 4.10418846970253e-175 | 0.291729070816484 | 1 | 0.645138889 | 8.95944342936062e-171 | 5 |
| DNAJC2 | 1.41857020118978e-174 | 0.269316163062653 | 0.692361111 | 0.579861111 | 3.09673874919729e-170 | 5 |
| HMGCR | 6.38116156425978e-174 | 0.375801790040856 | 0.686805556 | 0.544444444 | 1.39300756947791e-169 | 5 |
| USO1 | 1.54981400049079e-173 | 0.274632050989237 | 0.688194444 | 0.547916667 | 3.3832439630714e-169 | 5 |
| POLR2L | 3.35450680048491e-173 | 0.275700560726902 | 1 | 0.690972222 | 7.32288834545856e-169 | 5 |
| ERG28 | 1.15444274448166e-172 | 0.274142713229852 | 0.693055556 | 0.581944444 | 2.52014851120346e-168 | 5 |
| TMED2 | 6.98401570986295e-172 | 0.25420304052449 | 1 | 0.656944444 | 1.52461062946308e-167 | 5 |
| AGO2 | 6.8410790687905e-170 | 0.271710099717723 | 0.674305556 | 0.46875 | 1.49340756071697e-165 | 5 |
| MTHFD2 | 8.62435739039056e-169 | 0.250292622898283 | 0.693055556 | 0.470833333 | 1.88269721832226e-164 | 5 |
| SSRP1 | 1.04063341940379e-167 | 0.276391691522095 | 1 | 0.63125 | 2.27170275455848e-163 | 5 |
| MCM6 | 1.47054495563044e-167 | 0.258563536924197 | 0.675 | 0.401388889 | 3.21019963814125e-163 | 5 |
| BUB3 | 6.42889632505153e-167 | 0.352913559336187 | 1 | 0.640277778 | 1.40342806775875e-162 | 5 |
| STOML2 | 3.06866121864284e-164 | 0.253424571614793 | 1 | 0.663194444 | 6.69888744029732e-160 | 5 |
| ATP11C | 3.37054921247414e-164 | 0.252874009538559 | 0.679861111 | 0.456944444 | 7.35790893083104e-160 | 5 |
| CHAF1A | 1.18203908256998e-163 | 0.269620732771508 | 0.691666667 | 0.475694444 | 2.58039131725026e-159 | 5 |
| ABRACL | 1.64211005393449e-163 | 0.260648781109677 | 1 | 0.684027778 | 3.58472624773898e-159 | 5 |
| UBTF | 9.33057352303176e-162 | 0.273586995126587 | 0.691666667 | 0.531944444 | 2.03686420007783e-157 | 5 |
| ELOVL6 | 6.92222070459209e-161 | 0.262465995647633 | 0.688194444 | 0.510416667 | 1.51112077981245e-156 | 5 |
| STK26 | 2.55986240094052e-160 | 0.269619021842657 | 0.693055556 | 0.61875 | 5.58817962125316e-156 | 5 |
| ADAR | 7.62702098219513e-160 | 0.257501785057547 | 0.681944444 | 0.524305556 | 1.6649786804132e-155 | 5 |
| HNRNPL | 1.16235390445897e-159 | 0.250801855541308 | 1 | 0.651388889 | 2.53741857343394e-155 | 5 |
| KPNB1 | 1.43825380832151e-159 | 0.298342924121688 | 1 | 0.68125 | 3.13970806356585e-155 | 5 |
| PRKDC | 2.17276869920235e-153 | 0.289305263377593 | 1 | 0.06875 | 4.74315407035874e-149 | 5 |
| SUPT16H | 3.14481049083914e-153 | 0.299139499031411 | 1 | 0.686111111 | 6.86512130150185e-149 | 5 |
| HNRNPU | 2.09894623978588e-151 | 0.34414714573907 | 1 | 0.69375 | 4.58199964145257e-147 | 5 |
| RRP1B | 6.6153214920707e-150 | 0.259125677625865 | 0.692361111 | 0.609027778 | 1.44412468171903e-145 | 5 |
| EPRS | 1.02557455592369e-149 | 0.252978998558199 | 1 | 0.640972222 | 2.23882925558141e-145 | 5 |
| HIST1H4C | 6.6536074654778e-149 | 0.573743456737006 | 0.692361111 | 0.56875 | 1.4524825097138e-144 | 5 |
| SMS | 3.55020440216274e-147 | 0.26614598889674 | 1 | 0.690972222 | 7.75009620992126e-143 | 5 |
| HMMR | 3.06165244883874e-146 | 0.276153658257547 | 0.671527778 | 0.359027778 | 6.68358729581497e-142 | 5 |
| GMNN | 1.78596582162313e-144 | 0.271265192076075 | 1 | 0.591666667 | 3.8987633886033e-140 | 5 |
| HIST1H1D | 2.51490376961298e-143 | 0.266239387104688 | 0.384722222 | 0.144444444 | 5.49003492906514e-139 | 5 |
| ANP32E | 6.13998791492736e-142 | 0.296393673760668 | 1 | 0.068055556 | 1.34035936182864e-137 | 5 |
| QSER1 | 1.50378668326699e-133 | 0.264760889288259 | 0.686805556 | 0.573611111 | 3.28276632957183e-129 | 5 |
| LRPPRC | 9.52181196601883e-132 | 0.254100072268219 | 0.693055556 | 0.620138889 | 2.07861155218191e-127 | 5 |

| CLTC | 1.14565226591859e-130 | 0.26122080086445 | 0.690972222 | 0.606944444 | 2.50095889650027e-126 | 5 |
| --- | --- | --- | --- | --- | --- | --- |
| CKB | 4.39546599021297e-123 | 0.280762596610815 | 1 | 0.649305556 | 9.59530225663492e-119 | 5 |
| IDI1 | 3.62009603895207e-121 | 0.26882093797963 | 0.677083333 | 0.529861111 | 7.90266965303236e-117 | 5 |
| MT-CO2 | 5.39149265813895e-119 | 0.30311114158102 | 1 | 1 | 1.17696284727173e-114 | 5 |
| PKIB | 8.23174712290543e-117 | 0.327328399320669 | 0.510416667 | 00:05 | 1.79699039693026e-112 | 5 |
| MT-ND4 | 4.8056081386096e-112 | 0.39856297830115 | 1 | 0.69375 | 1.04906425665848e-107 | 5 |
| MT-CO3 | 1.44563228420483e-97 | 0.363713121749024 | 1 | 1 | 3.15581527641915e-93 | 5 |
| MT-ATP6 | 4.69331162129094e-91 | 0.44535120654822 | 0.69375 | 0.69375 | 1.02454992692781e-86 | 5 |
| BNIP3 | 1.17948328136692e-89 | 0.369437570858093 | 0.690277778 | 0.574305556 | 2.57481200322398e-85 | 5 |
| MT-ND5 | 1.84173566620436e-68 | 0.356485104200574 | 0.692361111 | 0.684722222 | 4.02050895932412e-64 | 5 |
| MT-ND1 | 3.95761665676542e-60 | 0.340258818700036 | 1 | 0.693055556 | 8.63947716171892e-56 | 5 |
| MT-ND2 | 1.07098497209533e-47 | 0.3212022351618 | 1 | 0.693055556 | 2.3379601940841e-43 | 5 |
| ELAVL3 | 0 | 1.77631235939252 | 1 | 0.032 | 0 | 6 |
| CNTN2 | 0 | 1.39339939108429 | 0.584027778 | 0.031 | 0 | 6 |
| ONECUT2 | 0 | 1.38865591818496 | 0.601388889 | 0.031 | 0 | 6 |
| SYP | 0 | 1.29074085897623 | 1 | 0.048 | 0 | 6 |
| NEUROD1 | 0 | 1.25618630751558 | 0.652083333 | 0.014 | 0 | 6 |
| ELAVL4 | 0 | 1.25009428430932 | 0.559027778 | 0.017 | 0 | 6 |
| TFAP2A | 0 | 1.22887700566886 | 0.593055556 | 0.022 | 0 | 6 |
| RSPO3 | 0 | 1.15317884499189 | 0.516666667 | 0.004 | 0 | 6 |
| UNCX | 0 | 0.986805425451253 | 0.584027778 | 0.001 | 0 | 6 |
| TMEM163 | 0 | 0.982342876502812 | 0.652083333 | 0.045 | 0 | 6 |
| NEUROG1 | 0 | 0.96880633287789 | 0.550694444 | 0.001 | 0 | 6 |
| LHX1 | 0 | 0.939770789291529 | 0.397916667 | 0.004 | 0 | 6 |
| DCC | 0 | 0.930338978700807 | 0.635416667 | 0.041 | 0 | 6 |
| EBF3 | 0 | 0.895851079499912 | 0.593055556 | 0.008 | 0 | 6 |
| INSM1 | 0 | 0.858485780031643 | 0.054166667 | 0.012 | 0 | 6 |
| CRH | 0 | 0.828993199806167 | 0.288194444 | 0.001 | 0 | 6 |
| CLSTN2 | 0 | 0.768826937727685 | 0.508333333 | 0.024 | 0 | 6 |
| ST18 | 0 | 0.696179354827309 | 0.054166667 | 0.001 | 0 | 6 |
| LHX9 | 0 | 0.69617231971232 | 0.042361111 | 0.008 | 0 | 6 |
| TTC9B | 0 | 0.631140073768516 | 0.448611111 | 0.004 | 0 | 6 |
| NSG2 | 0 | 0.623365292386518 | 0.054166667 | 0.003 | 0 | 6 |
| LHX1-DT | 0 | 0.590090294477706 | 0.431944444 | 0.004 | 0 | 6 |
| P2RX3 | 0 | 0.586261670435306 | 0.372916667 | 0.011 | 0 | 6 |
| SYT4 | 0 | 0.581743072406556 | 0.05 | 0.012 | 0 | 6 |
| SLIT1 | 0 | 0.524989713378696 | 0.440277778 | 0.008 | 0 | 6 |
| TBR1 | 0 | 0.519630512255089 | 0.288194444 | 0.003 | 0 | 6 |
| EBF1 | 0 | 0.509055034009948 | 0.397916667 | 0.008 | 0 | 6 |
| ADCYAP1 | 0 | 0.48106263816986 | 0.431944444 | 0.002 | 0 | 6 |
| SCRT1 | 0 | 0.468820922774321 | 0.465972222 | 0.003 | 0 | 6 |
| FGF18 | 0 | 0.451398085146757 | 0.363888889 | 0.012 | 0 | 6 |
| EBF2 | 0 | 0.430268768510921 | 0.431944444 | 0.003 | 0 | 6 |
| NEUROD4 | 0 | 0.42379851346776 | 0.304861111 | 0.003 | 0 | 6 |
| AP000894.2 | 0 | 0.42338780489592 | 0.372916667 | 0 | 0 | 6 |
| MAL | 0 | 0.420013991257784 | 0.321527778 | 0.003 | 0 | 6 |
| MIR217HG | 0 | 0.390424704128123 | 00:05 | 0.004 | 0 | 6 |
| CABP7 | 0 | 0.379429364085188 | 0.355555556 | 0.003 | 0 | 6 |
| RELN | 0 | 0.379177609898574 | 00:05 | 0.002 | 0 | 6 |
| GREM2 | 0 | 0.37452948535912 | 0.245833333 | 0 | 0 | 6 |
| EOMES | 0 | 0.333832296375863 | 0.236805556 | 0.001 | 0 | 6 |
| DUSP26 | 0 | 0.296084505976968 | 0.321527778 | 0.008 | 0 | 6 |
| CPLX2 | 0 | 0.289432765234031 | 0.363888889 | 0.007 | 0 | 6 |
| SCN3A | 0 | 0.280872516676414 | 0.304861111 | 0.005 | 0 | 6 |
| SNAP25 | 6.18807347142369e-288 | 0.416365030708589 | 0.440277778 | 00:02 | 1.35085643881179e-283 | 6 |
| ACTL6B | 3.21709473807482e-272 | 0.258664912408302 | 0.330555556 | 0.011 | 7.02291781321734e-268 | 6 |
| CELF3 | 2.10212504342126e-270 | 0.728308120712176 | 0.635416667 | 0.051 | 4.58893896978861e-266 | 6 |
| DNER | 2.46466317966984e-260 | 0.674034580895859 | 0.440277778 | 0.023 | 5.38035972121926e-256 | 6 |
| GABBR2 | 3.0914233632645e-259 | 0.267437761746188 | 0.279166667 | 0.008 | 6.7485772020064e-255 | 6 |
| GPX2 | 7.78683192331524e-254 | 0.341685127996921 | 0.186111111 | 0.003 | 1.69986540885972e-249 | 6 |
| LINC00599 | 2.1646194964859e-243 | 0.753503125315843 | 0.609722222 | 0.053 | 4.72536436082872e-239 | 6 |
| KLHL35 | 2.06157780243197e-239 | 1.52016907819991 | 0.601388889 | 0.052 | 4.50042434270898e-235 | 6 |
| SST | 1.91541844058993e-229 | 2.51758073063371 | 0.313194444 | 0.012 | 4.18135845580782e-225 | 6 |
| PARM1 | 1.05128172079433e-222 | 0.560431160515777 | 0.38125 | 00:02 | 2.29494799649403e-218 | 6 |
| XKR4 | 2.7529724973015e-215 | 0.42381360017713 | 0.338888889 | 0.016 | 6.00973896160918e-211 | 6 |
| CHRNA3 | 1.23812239250719e-213 | 1.17346895917008 | 0.490972222 | 0.037 | 2.7028211828432e-209 | 6 |
| NHLH1 | 2.15210243305403e-212 | 1.95741723792684 | 0.686111111 | 0.083 | 4.69803961135694e-208 | 6 |
| VSTM2B | 7.2052190839716e-210 | 0.266593134624031 | 0.330555556 | 0.015 | 1.572899326031e-205 | 6 |
| CREG2 | 9.87586989835796e-199 | 0.399336277359795 | 0.2625 | 00:01 | 2.15590239881154e-194 | 6 |
| ANKS1B | 2.69850116445463e-198 | 0.975160655225128 | 0.660416667 | 0.081 | 5.89082804200446e-194 | 6 |
| THSD7A | 4.78613504514992e-198 | 0.481361013083784 | 0.313194444 | 0.015 | 1.04481328035623e-193 | 6 |
| MAB21L1 | 2.72875544833424e-186 | 0.419120884883392 | 0.228472222 | 0.008 | 5.95687314371365e-182 | 6 |
| SRRM4 | 9.54096485641584e-185 | 0.747131288806278 | 0.593055556 | 0.068 | 2.08279262815558e-180 | 6 |
| RAB26 | 6.64696618394727e-177 | 0.558220127455606 | 0.338888889 | 00:02 | 1.45103271795569e-172 | 6 |
| PCSK2 | 3.87667833439763e-176 | 0.378474038820015 | 0.245833333 | 00:01 | 8.46278880399002e-172 | 6 |
| CHGB | 1.41304837415196e-164 | 0.587795783704453 | 0.575694444 | 0.071 | 3.08468460077373e-160 | 6 |
| TMEM176A | 1.54533494774736e-152 | 0.328412656812512 | 0.279166667 | 0.016 | 3.37346619093249e-148 | 6 |
| ONECUT1 | 5.52351634204938e-144 | 0.637411894888909 | 0.440277778 | 0.045 | 1.20578361746938e-139 | 6 |
| DUSP8 | 6.3944281045486e-143 | 0.517322541234167 | 0.40625 | 0.038 | 1.39590365522296e-138 | 6 |
| MAPK8IP2 | 4.24121686931479e-138 | 0.37316687204171 | 0.448611111 | 0.049 | 9.25857642571418e-134 | 6 |
| UBE2QL1 | 4.46245757512626e-134 | 0.50404154004059 | 0.40625 | 0.041 | 9.74154488650063e-130 | 6 |
| ROBO3 | 5.13451313347362e-133 | 2.04121339681237 | 0.660416667 | 0.092361111 | 1.12086421703729e-128 | 6 |
| FAM110A | 1.29645181541395e-132 | 0.660074082474932 | 0.533333333 | 0.076 | 2.83015431304865e-128 | 6 |
| AKAP6 | 1.13369688476038e-129 | 0.540137232531433 | 0.440277778 | 00:05 | 2.47486029943191e-125 | 6 |
| NEFM | 5.80846003497046e-129 | 3.2613418697212 | 0.686111111 | 0.105555556 | 1.26798682563405e-124 | 6 |
| ATP1A3 | 8.85491389364574e-124 | 0.489160122179498 | 0.465972222 | 0.061 | 1.93302770298286e-119 | 6 |
| STMN4 | 1.62273289351348e-123 | 1.49844123995993 | 0.490972222 | 0.068 | 3.54242590653993e-119 | 6 |
| STMN2 | 1.77558025764173e-123 | 3.64045872529047 | 1 | 0.116666667 | 3.87609170243189e-119 | 6 |
| NFASC | 5.58411464140076e-120 | 0.45230733447214 | 0.415277778 | 0.048 | 1.21901222621779e-115 | 6 |
| GDAP1L1 | 3.17273837163486e-119 | 0.638895451533305 | 0.559027778 | 0.095 | 6.9260878652789e-115 | 6 |
| MANEAL | 8.89709972159527e-119 | 0.267732148169578 | 0.296527778 | 0.023 | 1.94223686922425e-114 | 6 |

| DCX | 4.35400894471855e-117 | 1.6230985272717 | 1 | 0.122222222 | 9.50480152632059e-113 | 6 |
| --- | --- | --- | --- | --- | --- | --- |
| SCG3 | 2.17919647736407e-115 | 1.13484042160491 | 1 | 0.115277778 | 4.75718591008577e-111 | 6 |
| RORB | 6.78755499322402e-115 | 1.13483991353958 | 0.061805556 | 0.089583333 | 1.4817232550208e-110 | 6 |
| CACNA2D1 | 1.93559090185113e-110 | 0.670606709279601 | 0.533333333 | 0.094 | 4.22539493874101e-106 | 6 |
| BAALC | 1.99397453881998e-110 | 0.800774277438454 | 0.64375 | 00:15 | 4.35284641824401e-106 | 6 |
| TCP10L | 3.45841456065153e-108 | 0.753590394956947 | 0.550694444 | 0.071527778 | 7.54971898590229e-104 | 6 |
| SRRM3 | 1.35417502771512e-107 | 0.364449632317913 | 0.448611111 | 0.064 | 2.95616408550211e-103 | 6 |
| RUNX1T1 | 1.12783777223045e-105 | 0.499074546768512 | 0.465972222 | 0.071 | 2.46206985677906e-101 | 6 |
| GNG3 | 8.50358799777357e-105 | 1.33793571129167 | 0.054166667 | 0.072222222 | 1.85633325991397e-100 | 6 |
| OLFM1 | 1.77858928043496e-103 | 1.04531752045844 | 0.652083333 | 00:17 | 3.88266039918952e-99 | 6 |
| CNRIP1 | 5.17067340703582e-103 | 0.386903201620888 | 0.465972222 | 0.072 | 1.12875800475592e-98 | 6 |
| HS3ST1 | 7.13192835734451e-101 | 0.254544889691014 | 0.2625 | 0.022 | 1.55689996040831e-96 | 6 |
| CADPS | 2.00961039612861e-100 | 0.574394186581976 | 0.559027778 | 0.079166667 | 4.38697949474877e-96 | 6 |
| RIMS1 | 2.7787514470581e-98 | 0.507677830773957 | 0.40625 | 0.057 | 6.06601440892784e-94 | 6 |
| RAB3A | 2.58376360274228e-94 | 0.40341755742451 | 0.397916667 | 0.057 | 5.64035594478639e-90 | 6 |
| NEFL | 4.19526016360603e-94 | 1.71104337190792 | 0.660416667 | 0.140277778 | 9.15825293715195e-90 | 6 |
| CHST8 | 5.5726200959432e-94 | 0.298776088147567 | 0.338888889 | 00:04 | 1.2165029669444e-89 | 6 |
| FNDC5 | 7.1446870220557e-93 | 1.18410580643156 | 0.66875 | 00:21 | 1.55968517691476e-88 | 6 |
| GRM2 | 1.84490438761665e-92 | 0.28411364801534 | 0.330555556 | 0.038 | 4.02742627816714e-88 | 6 |
| INA | 3.125415966502e-92 | 1.38894082317846 | 0.686111111 | 0.163888889 | 6.82278305487387e-88 | 6 |
| SNCG | 8.5641075563119e-89 | 0.967253204635789 | 0.40625 | 0.064 | 1.86954467954289e-84 | 6 |
| STMN3 | 1.14721405849571e-88 | 0.707337805124244 | 0.609722222 | 0.113888889 | 2.50436828969614e-84 | 6 |
| DLL3 | 8.7992911575918e-88 | 1.74392063541147 | 0.686111111 | 0.175 | 1.92088525970229e-83 | 6 |
| TPPP3 | 1.45956421935975e-87 | 0.496147036462719 | 0.465972222 | 0.085 | 3.18622869086233e-83 | 6 |
| LMO2 | 7.27707731892956e-84 | 0.332811380042671 | 0.338888889 | 0.044 | 1.58858597872232e-79 | 6 |
| BRSK2 | 3.03642898499152e-79 | 0.533583427556305 | 0.550694444 | 0.1 | 6.62852447423648e-75 | 6 |
| PCSK1N | 3.17268153065647e-78 | 0.814164373862056 | 0.508333333 | 00:12 | 6.92596378142308e-74 | 6 |
| SCHIP1 | 3.70302558531151e-78 | 0.428592951910601 | 0.508333333 | 0.081944444 | 8.08370485273503e-74 | 6 |
| GNG8 | 1.79074076722759e-74 | 0.826675995998495 | 0.465972222 | 00:01 | 3.90918709485784e-70 | 6 |
| ST8SIA3 | 2.62394179310476e-72 | 0.254062047635638 | 0.288194444 | 0.037 | 5.72806493434769e-68 | 6 |
| LZTS1 | 1.0835778610537e-71 | 0.330015729422229 | 0.288194444 | 0.038 | 2.36545047068023e-67 | 6 |
| SEPTIN4 | 6.75817360386091e-70 | 0.636802673697908 | 0.508333333 | 0.092361111 | 1.47530929772284e-65 | 6 |
| TLCD3B | 1.08046614337302e-69 | 1.33376862942904 | 0.66875 | 0.223611111 | 2.35865759098331e-65 | 6 |
| CHN2 | 1.60168838181405e-68 | 0.262651930917143 | 0.338888889 | 0.054 | 3.49648573750007e-64 | 6 |
| RTN1 | 6.7939214653267e-68 | 1.44081790415139 | 0.66875 | 0.217361111 | 1.48311305588082e-63 | 6 |
| CAMKV | 7.15236800486361e-67 | 0.518899930528675 | 0.457638889 | 0.075694444 | 1.56136193546173e-62 | 6 |
| ROBO2 | 1.93831089867763e-66 | 0.58018939891783 | 0.482638889 | 0.0875 | 4.23133269181326e-62 | 6 |
| CDKN1C | 3.45679652174908e-66 | 2.18104179499738 | 1 | 0.286805556 | 7.54618680697824e-62 | 6 |
| RGS16 | 7.253656318435e-66 | 1.64536299145603 | 0.584027778 | 0.150694444 | 1.58347317431436e-61 | 6 |
| PSTPIP1 | 2.73065061996492e-65 | 0.537465412870021 | 0.313194444 | 0.049 | 5.96101030338341e-61 | 6 |
| PHLDA1 | 2.81053166995918e-64 | 0.683655726035065 | 0.465972222 | 0.081944444 | 6.1353906355209e-60 | 6 |
| IGFBPL1 | 9.32592153535373e-64 | 1.80769881763097 | 0.686111111 | 0.299305556 | 2.03584867116772e-59 | 6 |
| MAP6 | 1.55517915905447e-62 | 1.38119142334763 | 0.686111111 | 0.309722222 | 3.39495610421592e-58 | 6 |
| AC004540.2 | 4.85293527220997e-62 | 0.615020423777488 | 0.550694444 | 0.126388889 | 1.05939576992344e-57 | 6 |
| SYT11 | 5.01322215086722e-62 | 0.536034630693154 | 0.457638889 | 0.079166667 | 1.09438639553431e-57 | 6 |
| RBP1 | 5.84472995833073e-62 | 2.05024369046122 | 0.660416667 | 0.252777778 | 1.2759045499036e-57 | 6 |
| INKA2 | 6.93881203712167e-62 | 0.423741850112428 | 0.474305556 | 0.088194444 | 1.51474266770366e-57 | 6 |
| RBFOX3 | 2.72164251230141e-61 | 0.439046187534132 | 0.457638889 | 00:12 | 5.94134560435397e-57 | 6 |
| CELF4 | 4.11386143570162e-61 | 0.349235550311482 | 0.372916667 | 0.075 | 8.98055951413664e-57 | 6 |
| NDRG4 | 8.55982491543732e-60 | 0.695229211889797 | 0.584027778 | 0.161111111 | 1.86860977903997e-55 | 6 |
| GPM6A | 1.44072129963413e-59 | 0.968954936035768 | 0.626388889 | 0.206944444 | 3.14509459710131e-55 | 6 |
| ELAVL2 | 8.10474277738556e-58 | 1.07468485714253 | 0.652083333 | 0.258333333 | 1.76926534830327e-53 | 6 |
| GNG4 | 2.77936547528323e-57 | 0.872307569770231 | 0.677777778 | 0.257638889 | 6.06735483254328e-53 | 6 |
| SSTR2 | 3.81824732812197e-57 | 0.874831202014101 | 0.550694444 | 0.14375 | 8.33523391729026e-53 | 6 |
| SEZ6L2 | 5.30424561580649e-57 | 1.10528681845658 | 1 | 00:53 | 1.15791681793056e-52 | 6 |
| CORO2B | 4.35351856013742e-56 | 0.28427901383499 | 0.355555556 | 0.074 | 9.50373101677998e-52 | 6 |
| ASIC1 | 5.17984740659321e-56 | 0.512386823318121 | 0.415277778 | 0.073611111 | 1.1307606888593e-51 | 6 |
| LHX5-AS1 | 2.2369998547684e-55 | 1.73979271705613 | 1 | 0.385416667 | 4.88337068295941e-51 | 6 |
| TUBB3 | 2.36995451111014e-55 | 2.95200363161315 | 1 | 0.561111111 | 5.17361069775344e-51 | 6 |
| PCBP4 | 3.95119806139302e-55 | 1.17930145537937 | 1 | 0.425 | 8.62546536802097e-51 | 6 |
| CRABP1 | 5.86784582310335e-55 | 3.33680644924427 | 1 | 0.676388889 | 1.28095074318346e-50 | 6 |
| KIF5C | 1.85360128225721e-54 | 1.7053523173733 | 1 | 0.64375 | 4.0464115991675e-50 | 6 |
| MLLT11 | 4.41765011289467e-54 | 1.86602886595276 | 1 | 0.609722222 | 9.64373019644907e-50 | 6 |
| MAP1B | 4.42887581857965e-54 | 2.00754120996566 | 1 | 0.689583333 | 9.66823591195938e-50 | 6 |
| MAP1A | 5.89104059599251e-54 | 0.833313459660071 | 0.626388889 | 0.227083333 | 1.28601416210517e-49 | 6 |
| PPP1R1A | 1.56289667229046e-53 | 1.57696379271645 | 0.677777778 | 0.395138889 | 3.41180343561008e-49 | 6 |
| ME3 | 4.60015105804531e-53 | 0.965645412702826 | 0.559027778 | 0.164583333 | 1.00421297597129e-48 | 6 |
| GADD45G | 4.69262998007238e-53 | 0.358313983669239 | 0.40625 | 0.099 | 1.0244011246498e-48 | 6 |
| FRRS1L | 1.30336685929387e-52 | 0.619960215508045 | 0.550694444 | 0.15 | 2.84524985383852e-48 | 6 |
| NACAD | 2.5217806026584e-52 | 0.255375886014198 | 00:39 | 0.045 | 5.50504705560329e-48 | 6 |
| RTN4 | 4.44681330341131e-52 | 0.983417779203756 | 1 | 0.691666667 | 9.7073934413469e-48 | 6 |
| TPD52 | 3.17222265994105e-51 | 0.973803646940339 | 0.66875 | 0.294444444 | 6.92496206665131e-47 | 6 |
| WDR47 | 1.23211914357772e-50 | 0.369826866931523 | 0.448611111 | 0.09375 | 2.68971609043017e-46 | 6 |
| CRMP1 | 1.82306879119646e-50 | 1.02554284469262 | 1 | 0.509027778 | 3.97975917118186e-46 | 6 |
| KALRN | 2.14121054399656e-50 | 0.403254664199733 | 0.465972222 | 0.102777778 | 4.6742626175445e-46 | 6 |
| CBFA2T3 | 2.46775712418704e-50 | 0.263803993781716 | 0.372916667 | 0.089 | 5.38711380210032e-46 | 6 |
| REM2 | 3.34808150104671e-50 | 0.255810928681877 | 0.288194444 | 0.052 | 7.30886191678497e-46 | 6 |
| BASP1 | 5.79300941561892e-50 | 1.00417574029159 | 1 | 0.69375 | 1.26461395542961e-45 | 6 |
| EPB41 | 6.26128930201618e-50 | 1.3085234048189 | 1 | 0.6375 | 1.36683945463013e-45 | 6 |
| CAMK2N1 | 1.09169683466358e-49 | 1.29062172318858 | 1 | 0.597916667 | 2.38317419007059e-45 | 6 |
| TERF2IP | 1.19989897012906e-49 | 1.01837036486913 | 1 | 0.529861111 | 2.61937945179174e-45 | 6 |
| NDUFB8 | 1.88904163683522e-49 | 1.14410405368644 | 1 | 0.682638889 | 4.12377789321129e-45 | 6 |
| MTURN | 8.08969824851197e-49 | 0.413578414496222 | 0.508333333 | 0.129861111 | 1.76598112765016e-44 | 6 |
| TAGLN3 | 2.02575162211393e-48 | 1.84015973172224 | 0.660416667 | 0.367361111 | 4.42221579107471e-44 | 6 |
| TUBA1A | 2.67688375026702e-47 | 1.88942340383278 | 1 | 1 | 5.84363722683291e-43 | 6 |
| NKAIN4 | 3.95694979007013e-47 | 1.0824621070229 | 0.686111111 | 0.049305556 | 8.6380213917231e-43 | 6 |
| NCAN | 5.38728261178176e-47 | 0.498413734991189 | 0.042361111 | 0.089583333 | 1.17604379415196e-42 | 6 |
| SYT1 | 1.20988706632561e-46 | 1.19912194214706 | 0.686111111 | 0.596527778 | 2.64118346578881e-42 | 6 |
| BEX1 | 1.99284225497893e-46 | 1.11829364881002 | 0.686111111 | 0.052777778 | 4.35037464261901e-42 | 6 |
| CPE | 2.26927790710021e-46 | 0.978435039343498 | 1 | 0.403472222 | 4.95383367119975e-42 | 6 |
| ATP6V1G1 | 2.41573791471358e-46 | 0.733632782755454 | 1 | 0.693055556 | 5.27355586781974e-42 | 6 |

| MAP2 | 3.4934349704519e-46 | 1.39637856868256 | 0.686111111 | 0.552083333 | 7.6261685404965e-42 | 6 |
| --- | --- | --- | --- | --- | --- | --- |
| EPS8L1 | 6.38918618502571e-46 | 0.648611880830396 | 0.550694444 | 0.172916667 | 1.39475934419111e-41 | 6 |
| TBCB | 8.40584959983306e-46 | 0.849923790410161 | 1 | 0.672916667 | 1.83499696764356e-41 | 6 |
| LHX5 | 1.04606531745112e-45 | 0.857788112585465 | 0.64375 | 0.288888889 | 2.28356058799578e-41 | 6 |
| PPP2R5B | 1.20622486125409e-45 | 0.307113455431383 | 0.330555556 | 0.076 | 2.63318887211767e-41 | 6 |
| SH3BGRL3 | 1.62655654389772e-45 | 1.06666464109116 | 1 | 0.688888889 | 3.55077293532873e-41 | 6 |
| KLC1 | 2.98212624624074e-45 | 1.07431030930249 | 0.686111111 | 0.593055556 | 6.50998159554354e-41 | 6 |
| SNN | 4.01486122796187e-45 | 0.841923637815702 | 1 | 0.475694444 | 8.76444206064075e-41 | 6 |
| NOVA2 | 6.64515738705644e-45 | 0.508994885437233 | 0.584027778 | 0.213194444 | 1.45063785759442e-40 | 6 |
| CDKN2D | 7.19029253386595e-45 | 0.517486801137219 | 0.448611111 | 0.107638889 | 1.56964086014294e-40 | 6 |
| LBH | 1.20187129018347e-44 | 0.752993695605613 | 0.584027778 | 0.206944444 | 2.62368502647053e-40 | 6 |
| DNAJC12 | 1.52120380993181e-44 | 0.326505089664575 | 0.415277778 | 0.086805556 | 3.32078791708113e-40 | 6 |
| CRIP2 | 2.43411289498537e-44 | 0.357709052641016 | 0.363888889 | 0.097 | 5.31366844975306e-40 | 6 |
| LINC02609 | 2.50594283299443e-44 | 0.560541508091776 | 0.40625 | 0.086805556 | 5.47047320442684e-40 | 6 |
| GDI1 | 3.43753304888136e-44 | 0.83276300078307 | 1 | 0.514583333 | 7.50413464570801e-40 | 6 |
| SRGAP3 | 6.2474538837552e-44 | 0.966518026168185 | 0.677777778 | 0.445833333 | 1.36381918282376e-39 | 6 |
| JUND | 1.25696478300258e-43 | 1.12043997549281 | 1 | 0.679861111 | 2.74395412129463e-39 | 6 |
| GABARAP | 1.40653099291793e-43 | 0.769247916665968 | 1 | 0.69375 | 3.07045715753984e-39 | 6 |
| TMEFF1 | 1.42318823206038e-43 | 0.942810444334323 | 1 | 0.647916667 | 3.10681991058782e-39 | 6 |
| NNAT | 1.73799846637174e-43 | 1.86687235640338 | 0.686111111 | 0.675694444 | 3.7940506520895e-39 | 6 |
| DAAM1 | 2.10158888485748e-43 | 1.00998680967271 | 0.677777778 | 0.497222222 | 4.58776853564388e-39 | 6 |
| RNASEK | 2.22628543249379e-43 | 0.610094913507581 | 1 | 0.692361111 | 4.85998109913393e-39 | 6 |
| C4orf48 | 3.009898327182e-43 | 0.936457604375982 | 1 | 0.690972222 | 6.57060804823831e-39 | 6 |
| CAMK2N2 | 3.23900480885794e-43 | 0.326630734934195 | 0.389583333 | 0.079861111 | 7.07074749773689e-39 | 6 |
| EDIL3 | 4.76113137802295e-43 | 0.597884966219837 | 0.05 | 0.149305556 | 1.03935497982241e-38 | 6 |
| TMEM176B | 4.8763510617249e-43 | 0.252939039448682 | 0.186111111 | 0.026 | 1.06450743677455e-38 | 6 |
| NCOA1 | 5.66087788485573e-43 | 0.629344567385007 | 0.64375 | 0.311805556 | 1.23576964226401e-38 | 6 |
| RGMB | 1.47410803541208e-42 | 0.924667166700805 | 0.575694444 | 0.225694444 | 3.21797784130456e-38 | 6 |
| PHF21B | 2.07600408230238e-42 | 0.570611519432032 | 0.054166667 | 0.190277778 | 4.53191691166611e-38 | 6 |
| BEX2 | 2.69587925994146e-42 | 1.02204265273971 | 0.686111111 | 0.552777778 | 5.88510442445221e-38 | 6 |
| NKAIN1 | 3.88729417942224e-42 | 0.716565624666353 | 0.559027778 | 00:31 | 8.48596319367874e-38 | 6 |
| MARCKS | 5.96417420013065e-42 | 0.756365786537624 | 1 | 0.690972222 | 1.30197922788852e-37 | 6 |
| NPTX2 | 8.40347711152295e-42 | 0.772676809302015 | 0.457638889 | 0.122916667 | 1.83447905344546e-37 | 6 |
| KIF5A | 1.11041161977288e-41 | 0.332971206914873 | 0.40625 | 00:13 | 2.4240285659642e-37 | 6 |
| ATCAY | 1.38606361180237e-41 | 0.688719182573508 | 0.601388889 | 0.265277778 | 3.02577686456457e-37 | 6 |
| AFAP1 | 1.393443583689e-41 | 0.424974624946868 | 0.431944444 | 0.10625 | 3.04188734319309e-37 | 6 |
| MAPT | 1.57550180780815e-41 | 0.279603472028626 | 0.355555556 | 0.096 | 3.43932044644519e-37 | 6 |
| RUFY3 | 1.84864554615205e-41 | 0.801938621457902 | 0.686111111 | 0.484027778 | 4.03559322724993e-37 | 6 |
| STMN1 | 3.12770904609548e-41 | 1.11069911813971 | 1 | 1 | 6.82778884762643e-37 | 6 |
| GABARAPL2 | 3.94631709173904e-41 | 0.722321011479799 | 1 | 0.679166667 | 8.61481021126632e-37 | 6 |
| RTL8C | 5.4536206082629e-41 | 0.794971796073366 | 1 | 0.500694444 | 1.19052537878379e-36 | 6 |
| TUBB2B | 6.96133804907059e-41 | 1.51774888247431 | 1 | 0.683333333 | 1.51966009611211e-36 | 6 |
| KCNQ1OT1 | 2.00633586639668e-40 | 1.51767801729829 | 0.677777778 | 0.495833333 | 4.37983119634394e-36 | 6 |
| KIF1A | 3.10316072815266e-40 | 1.1648681027896 | 0.061805556 | 0.302777778 | 6.77419986955727e-36 | 6 |
| LYST | 4.44989408438348e-40 | 0.299485687061329 | 0.38125 | 0.079166667 | 9.71411878620915e-36 | 6 |
| CRABP2 | 4.76038658863484e-40 | 1.30151386247377 | 1 | 0.669444444 | 1.03919239229898e-35 | 6 |
| TACC2 | 6.52206808407615e-40 | 0.837781338523573 | 0.635416667 | 0.355555556 | 1.42376746275382e-35 | 6 |
| NCAM1 | 7.40314659698925e-40 | 0.801318307183625 | 0.061805556 | 00:45 | 1.61610690212275e-35 | 6 |
| IGDCC3 | 8.37743216338205e-40 | 0.805485324428081 | 0.677777778 | 0.044444444 | 1.8287934412663e-35 | 6 |
| NREP | 8.83968039627365e-40 | 0.760134322812601 | 1 | 0.668055556 | 1.92970223050654e-35 | 6 |
| SHC2 | 1.30001357125605e-39 | 0.451331793487077 | 0.054166667 | 00:26 | 2.83792962605195e-35 | 6 |
| WDFY3-AS2 | 1.56045890858815e-39 | 0.309688203160496 | 0.397916667 | 0.089583333 | 3.40648179744793e-35 | 6 |
| FSCN1 | 1.65731382162858e-39 | 0.719762250603555 | 1 | 0.65 | 3.61791607261518e-35 | 6 |
| MALAT1 | 2.07761997143266e-39 | 0.832748659389748 | 1 | 1 | 4.53544439763751e-35 | 6 |
| GABRB3 | 2.16737110939929e-39 | 0.44973439474347 | 0.601388889 | 0.2 | 4.73137113181865e-35 | 6 |
| VAMP2 | 2.54239929871858e-39 | 0.691966739698549 | 0.686111111 | 0.563194444 | 5.55005766910265e-35 | 6 |
| CFAP298 | 6.73167538897511e-39 | 1.56911257405904 | 0.686111111 | 0.640277778 | 1.46952473741327e-34 | 6 |
| KIAA0408 | 7.98866023319085e-39 | 0.381983565791671 | 0.465972222 | 0.129861111 | 1.74392452890556e-34 | 6 |
| KLHL24 | 9.76093320576344e-39 | 0.726465032046271 | 0.635416667 | 0.35625 | 2.13081171881816e-34 | 6 |
| ATL1 | 2.64931618456153e-38 | 0.50458889741873 | 0.559027778 | 0.218055556 | 5.78345723089782e-34 | 6 |
| SOX4 | 3.25548554769772e-38 | 0.92144888982013 | 1 | 0.69375 | 7.10672495062412e-34 | 6 |
| C18orf32 | 3.44948053664498e-38 | 0.689924548044211 | 1 | 0.063888889 | 7.53021601149598e-34 | 6 |
| MAP4K4 | 3.82064513190722e-38 | 1.05318849104572 | 1 | 0.661805556 | 8.34046832295346e-34 | 6 |
| H3F3B | 6.19765331501533e-38 | 0.5110512811472 | 1 | 1 | 1.35294771866785e-33 | 6 |
| SERINC1 | 1.30120882991447e-37 | 0.71780580657961 | 0.677777778 | 0.54375 | 2.84053887570329e-33 | 6 |
| CCDC50 | 2.2620749474164e-37 | 0.96089631315061 | 0.66875 | 0.589583333 | 4.93810961021001e-33 | 6 |
| GPC2 | 2.43211422428564e-37 | 0.697810680425713 | 0.64375 | 0.361805556 | 5.30930535161555e-33 | 6 |
| LOXL1 | 4.41131225299701e-37 | 0.450451387835528 | 0.516666667 | 0.18125 | 9.62989464829248e-33 | 6 |
| PAK3 | 5.6779497971685e-37 | 0.821763345157961 | 0.575694444 | 0.274305556 | 1.23949644072188e-32 | 6 |
| CNIH2 | 7.44562451829907e-37 | 0.508450931493867 | 0.575694444 | 0.231944444 | 1.62537983234469e-32 | 6 |
| LINGO1 | 7.87056671973567e-37 | 0.606267710132963 | 0.626388889 | 0.358333333 | 1.7181447149183e-32 | 6 |
| MAP1LC3A | 1.14555170285341e-36 | 0.584696132268183 | 0.054166667 | 0.202777778 | 2.50073936732899e-32 | 6 |
| TSC22D3 | 1.23803538772758e-36 | 0.671582454386746 | 0.66875 | 0.40625 | 2.70263125140931e-32 | 6 |
| CALM3 | 1.47892700689676e-36 | 0.660317350508273 | 1 | 0.686805556 | 3.22849765605563e-32 | 6 |
| NECAB3 | 1.62654719474399e-36 | 0.442644874351825 | 0.490972222 | 0.158333333 | 3.55075252612612e-32 | 6 |
| FAM89B | 1.79455573185052e-36 | 0.767142410418937 | 0.626388889 | 0.390277778 | 3.91751516262969e-32 | 6 |
| TAFA5 | 1.96419894761063e-36 | 0.461863649847571 | 0.567361111 | 0.2375 | 4.287846302634e-32 | 6 |
| SERPINI1 | 3.88148803258723e-36 | 0.433143206662553 | 0.042361111 | 0.1125 | 8.47328837513793e-32 | 6 |
| ASPHD1 | 8.03321177146084e-36 | 0.369412904216433 | 0.355555556 | 0.079861111 | 1.7536501297099e-31 | 6 |
| PNMA1 | 1.05527244045553e-35 | 0.561508347499776 | 0.660416667 | 0.404861111 | 2.30365973751443e-31 | 6 |
| DPYSL2 | 5.25570427669669e-35 | 0.636383032422023 | 1 | 0.679861111 | 1.14732024360289e-30 | 6 |
| CKB | 6.37139616440475e-35 | 1.03348009946917 | 1 | 0.065277778 | 1.39087578268956e-30 | 6 |
| MFNG | 2.05863214102067e-34 | 0.361304628877314 | 0.389583333 | 00:14 | 4.49399396384812e-30 | 6 |
| DYNC1I2 | 2.13002162073354e-34 | 0.613331596654985 | 1 | 0.679166667 | 4.64983719806131e-30 | 6 |
| CBLB | 2.35708454024408e-34 | 0.727289756302181 | 0.601388889 | 0.289583333 | 5.14551555135283e-30 | 6 |
| CCSAP | 3.12131420435225e-34 | 0.635918448527179 | 0.525 | 00:32 | 6.81382890810096e-30 | 6 |
| DCLK1 | 3.22339605920329e-34 | 0.409660148564435 | 0.415277778 | 0.114583333 | 7.03667359724079e-30 | 6 |
| GAP43 | 4.48290116361924e-34 | 1.26135854907722 | 0.042361111 | 0.127777778 | 9.78617324018081e-30 | 6 |
| RAB31 | 5.14259714813477e-34 | 0.500614577173113 | 0.465972222 | 0.153472222 | 1.12262895743782e-29 | 6 |
| APLP1 | 1.09839062573235e-33 | 0.709554208360725 | 0.66875 | 0.469444444 | 2.39778673597371e-29 | 6 |
| MXRA7 | 1.1256068689022e-33 | 0.777449899465766 | 0.652083333 | 0.547222222 | 2.45719979481351e-29 | 6 |

| ICK | 1.73134894542949e-33 | 0.553903616678973 | 0.609722222 | 0.347916667 | 3.77953474787257e-29 | 6 |
| --- | --- | --- | --- | --- | --- | --- |
| ABCA1 | 1.75137393629586e-33 | 0.60036827403681 | 0.626388889 | 00:05 | 3.82324930293386e-29 | 6 |
| AGAP3 | 1.99981280835745e-33 | 0.50660127454324 | 0.533333333 | 0.223611111 | 4.36559136064432e-29 | 6 |
| MAP1LC3B | 2.77363616368661e-33 | 0.693298547261206 | 1 | 0.629861111 | 6.05484774532786e-29 | 6 |
| GLRX | 3.17310490682661e-33 | 0.576284818845825 | 0.516666667 | 0.198611111 | 6.92688801160248e-29 | 6 |
| SPON2 | 9.46479956437256e-33 | 0.386637962113528 | 0.296527778 | 0.083 | 2.06616574490253e-28 | 6 |
| TMSB4X | 1.62804465223487e-32 | 0.756266331717538 | 1 | 1 | 3.55402147582872e-28 | 6 |
| TUBB2A | 2.11649289126708e-32 | 1.03979609645681 | 0.660416667 | 0.4875 | 4.62030398163604e-28 | 6 |
| ATP6V0E2 | 2.35275730486887e-32 | 0.573782118125572 | 0.635416667 | 00:55 | 5.13606919652874e-28 | 6 |
| CTNND2 | 2.75956674711672e-32 | 0.476049510129596 | 0.559027778 | 0.251388889 | 6.02413420895581e-28 | 6 |
| SRP14 | 3.20643460195026e-32 | 0.366820888427322 | 1 | 1 | 6.99964673605741e-28 | 6 |
| DBN1 | 6.24328802878654e-32 | 0.589915667181497 | 0.686111111 | 0.654861111 | 1.3629097766841e-27 | 6 |
| KLF7 | 1.4716220038971e-31 | 0.382007004716566 | 0.40625 | 00:17 | 3.21255083450736e-27 | 6 |
| UBE2E3 | 2.99050060656396e-31 | 0.567949087221046 | 0.686111111 | 0.681944444 | 6.52826282412913e-27 | 6 |
| TTC3 | 3.10915460610376e-31 | 0.67970492458023 | 1 | 0.679166667 | 6.7872845051245e-27 | 6 |
| SLCO3A1 | 5.95856441833019e-31 | 0.473914596577942 | 0.490972222 | 0.191666667 | 1.30075461252148e-26 | 6 |
| MRPS6 | 1.0556056606028e-30 | 0.696640982291698 | 1 | 0.685416667 | 2.30438715709591e-26 | 6 |
| GNAZ | 1.69802441291153e-30 | 0.374060771386945 | 0.465972222 | 0.165277778 | 3.70678729338587e-26 | 6 |
| KHDRBS3 | 2.07635649201292e-30 | 0.809711554882629 | 0.626388889 | 0.470138889 | 4.5326862220642e-26 | 6 |
| CSNK1E | 2.18988879392865e-30 | 0.579594646533785 | 0.686111111 | 0.656944444 | 4.78052723714624e-26 | 6 |
| STXBP1 | 2.36020006149855e-30 | 0.385956426191225 | 0.559027778 | 0.254861111 | 5.15231673425134e-26 | 6 |
| HPCA | 2.51679733829801e-30 | 0.265918033090756 | 0.2625 | 00:07 | 5.49416858950455e-26 | 6 |
| ACAP3 | 3.37425016455297e-30 | 0.279742511128719 | 0.042361111 | 0.129166667 | 7.36598810921912e-26 | 6 |
| C3orf14 | 6.79194201089176e-30 | 0.4958233577013 | 0.593055556 | 0.314583333 | 1.48268094097767e-25 | 6 |
| ADGRG1 | 7.02305517553567e-30 | 0.362422086808494 | 0.482638889 | 00:25 | 1.53313294481944e-25 | 6 |
| TMEM132A | 7.05999629373839e-30 | 0.59755604334028 | 0.575694444 | 0.331944444 | 1.54119719092309e-25 | 6 |
| FABP3 | 1.43881947323503e-29 | 0.252732196727161 | 0.245833333 | 0.063 | 3.14094291007206e-25 | 6 |
| SORBS2 | 2.55216058032563e-29 | 0.583955077683615 | 0.508333333 | 0.202083333 | 5.57136654685085e-25 | 6 |
| ATP6V0B | 4.17578094821131e-29 | 0.547529515772749 | 0.686111111 | 0.675694444 | 9.11572980994529e-25 | 6 |
| FYN | 4.34608652786156e-29 | 0.590707918040526 | 0.66875 | 0.611805556 | 9.48750689032178e-25 | 6 |
| HAGH | 4.44595244665929e-29 | 0.475605130352814 | 0.626388889 | 0.418055556 | 9.70551419105722e-25 | 6 |
| TTLL7 | 5.50657548174959e-29 | 0.402891328841297 | 0.508333333 | 0.209027778 | 1.20208542766594e-24 | 6 |
| RPAIN | 6.50029316788584e-29 | 0.462639715795893 | 1 | 0.684722222 | 1.41901399854948e-24 | 6 |
| PKIA | 7.54723168169602e-29 | 0.71154027719912 | 0.550694444 | 0.295138889 | 1.64756067611424e-24 | 6 |
| DCTN2 | 9.81214156710188e-29 | 0.538369683647857 | 1 | 0.654861111 | 2.14199050409834e-24 | 6 |
| KIDINS220 | 1.88042141597844e-28 | 0.596011086739817 | 0.64375 | 0.526388889 | 4.10495995108093e-24 | 6 |
| NRXN1 | 2.14734684157817e-28 | 0.728324073454142 | 0.533333333 | 0.264583333 | 4.68765815516514e-24 | 6 |
| GSE1 | 2.14993770399916e-28 | 0.427405268636663 | 0.474305556 | 0.188194444 | 4.69331400783016e-24 | 6 |
| DNAJA1 | 2.17802237857832e-28 | 0.49851818122127 | 1 | 1 | 4.75462285243646e-24 | 6 |
| CASTOR3 | 3.11416839684031e-28 | 0.405094771397745 | 0.567361111 | 0.281944444 | 6.79822961030239e-24 | 6 |
| CALY | 3.68142325386367e-28 | 0.317762587828316 | 0.321527778 | 0.077777778 | 8.0365469631844e-24 | 6 |
| EIF1 | 3.70877280415745e-28 | 0.415559542636463 | 1 | 1 | 8.09625103147572e-24 | 6 |
| JPT1 | 3.93285480574524e-28 | 0.507481284484233 | 1 | 0.693055556 | 8.58542204094186e-24 | 6 |
| PCDH11X | 4.79747459119724e-28 | 0.455944774258775 | 0.431944444 | 0.152083333 | 1.04728870325836e-23 | 6 |
| PBX3 | 7.13792123131465e-28 | 0.526094205479957 | 0.652083333 | 0.477083333 | 1.55820820479599e-23 | 6 |
| NAPB | 9.22437329484625e-28 | 0.385148203391011 | 0.482638889 | 0.190972222 | 2.01368069026494e-23 | 6 |
| GDAP1 | 9.29186596536915e-28 | 0.458699031108964 | 0.054166667 | 0.261805556 | 2.02841434024008e-23 | 6 |
| ATXN7L3B | 1.0642799517773e-27 | 0.534848499188446 | 1 | 0.663888889 | 2.32332313472984e-23 | 6 |
| CD24 | 1.15700799172692e-27 | 0.570430820386708 | 1 | 0.675 | 2.52574844593988e-23 | 6 |
| DPYSL4 | 1.26826960020324e-27 | 0.292022124954878 | 0.482638889 | 0.179166667 | 2.76863253724368e-23 | 6 |
| GFRA1 | 1.43474429611174e-27 | 0.432897953492146 | 0.457638889 | 0.165972222 | 3.13204679841193e-23 | 6 |
| JAKMIP2 | 1.51410441174937e-27 | 0.490411166213957 | 0.559027778 | 0.295138889 | 3.30528993084888e-23 | 6 |
| RTN2 | 1.57301745020357e-27 | 0.296476621215042 | 0.415277778 | 0.136111111 | 3.43389709379438e-23 | 6 |
| CARD19 | 1.58796295262239e-27 | 0.36273511290683 | 0.465972222 | 0.172916667 | 3.46652312557467e-23 | 6 |
| ZIC3 | 1.68757978497047e-27 | 0.369085530827645 | 00:39 | 0.081 | 3.68398667059054e-23 | 6 |
| MAPRE3 | 1.69623244276362e-27 | 0.329441290284 | 0.482638889 | 0.179166667 | 3.70287542255297e-23 | 6 |
| SEC11C | 2.45691468950176e-27 | 0.535270612467423 | 0.677777778 | 0.058333333 | 5.36344476718235e-23 | 6 |
| RIMS3 | 2.46177064555932e-27 | 0.288551685226309 | 0.389583333 | 0.119444444 | 5.37404531925599e-23 | 6 |
| CFL1 | 2.50097975842199e-27 | 0.41940552982514 | 1 | 1 | 5.45963881263521e-23 | 6 |
| SLC16A2 | 3.80514194031062e-27 | 0.668623758375075 | 0.593055556 | 0.386805556 | 8.30662485569809e-23 | 6 |
| DBNDD1 | 4.78380133982932e-27 | 0.297120563736476 | 0.415277778 | 0.1375 | 1.04430383248474e-22 | 6 |
| PLPPR3 | 5.12105757303331e-27 | 0.569963536794548 | 0.64375 | 0.515972222 | 1.11792686819317e-22 | 6 |
| NSG1 | 5.30295273752811e-27 | 0.757163586575377 | 0.490972222 | 0.210416667 | 1.15763458260239e-22 | 6 |
| RALGDS | 5.97445525810318e-27 | 0.389918162077314 | 0.533333333 | 0.261805556 | 1.30422358284392e-22 | 6 |
| SEPTIN6 | 7.81539589182143e-27 | 0.560651763780385 | 0.609722222 | 0.3875 | 1.70610092318462e-22 | 6 |
| DENND1B | 8.17555560345956e-27 | 0.364067454989326 | 0.448611111 | 0.163194444 | 1.78472378823522e-22 | 6 |
| UQCR11 | 8.85732851824226e-27 | 0.413733624534268 | 1 | 0.69375 | 1.93355481553229e-22 | 6 |
| AC008522.1 | 1.04081118678404e-26 | 0.3120195272455 | 00:39 | 0.082 | 2.27209082074956e-22 | 6 |
| PARD3B | 1.11299772261486e-26 | 0.402840833812631 | 0.279166667 | 00:09 | 2.42967402846824e-22 | 6 |
| TMSB10 | 1.28932319527275e-26 | 0.66155887353063 | 1 | 1 | 2.81459253528041e-22 | 6 |
| RAB15 | 1.6206358638945e-26 | 0.25954241813555 | 0.330555556 | 0.0875 | 3.5378480908817e-22 | 6 |
| YWHAZ | 2.04362400508281e-26 | 0.487047551524838 | 1 | 0.69375 | 4.46123120309578e-22 | 6 |
| CCDC167 | 2.32907347856561e-26 | 0.555596260421257 | 0.66875 | 0.059722222 | 5.08436740370872e-22 | 6 |
| MDK | 4.91817291761129e-26 | 0.47188172006262 | 1 | 1 | 1.07363714791454e-21 | 6 |
| PRDX2 | 5.10073145836078e-26 | 0.374898325678279 | 1 | 1 | 1.11348967736016e-21 | 6 |
| BRD3OS | 5.13118792655001e-26 | 0.378071130288262 | 0.516666667 | 0.245138889 | 1.12013832436587e-21 | 6 |
| EID1 | 5.24968825370696e-26 | 0.36745920908033 | 1 | 0.69375 | 1.14600694578423e-21 | 6 |
| TM7SF2 | 5.61360446862765e-26 | 0.542009457915224 | 0.64375 | 0.045833333 | 1.22544985550142e-21 | 6 |
| SEPTIN3 | 6.17471741331708e-26 | 0.33996959911914 | 0.431944444 | 0.153472222 | 1.34794081132712e-21 | 6 |
| DYNLRB1 | 7.25442179392105e-26 | 0.466098460990131 | 0.686111111 | 0.681944444 | 1.58364027761297e-21 | 6 |
| DST | 1.41463508105939e-25 | 0.930660037942259 | 0.660416667 | 0.614583333 | 3.08814838195266e-21 | 6 |
| SCOC | 1.4919117375234e-25 | 0.50578985565139 | 1 | 0.636111111 | 3.25684332301358e-21 | 6 |
| ATP6V0C | 1.8902790814123e-25 | 0.463418035918428 | 0.677777778 | 0.679861111 | 4.12647923472305e-21 | 6 |
| PLD3 | 2.26993247147931e-25 | 0.599273788883844 | 0.64375 | 0.564583333 | 4.95526258523932e-21 | 6 |
| ARL4D | 2.28376240284474e-25 | 0.947152958126857 | 0.431944444 | 0.176388889 | 4.98545332541008e-21 | 6 |
| NDUFB10 | 3.5106854482156e-25 | 0.492318525655868 | 1 | 0.690277778 | 7.66382633345466e-21 | 6 |
| NGRN | 3.56540894529553e-25 | 0.474600524273655 | 0.686111111 | 0.681944444 | 7.78328772758014e-21 | 6 |
| ZC2HC1A | 4.79442908584659e-25 | 0.608989234552793 | 0.626388889 | 0.422916667 | 1.04662386944031e-20 | 6 |
| SARAF | 5.06096046109086e-25 | 0.535181513644934 | 1 | 0.679861111 | 1.10480766865613e-20 | 6 |
| CDC42 | 5.27639214943218e-25 | 0.425196294226013 | 1 | 0.69375 | 1.15183640622105e-20 | 6 |
| THBS3 | 7.52141825513488e-25 | 0.270657856930719 | 0.38125 | 0.121527778 | 1.64192560509594e-20 | 6 |

| CXXC5 | 7.83434355242814e-25 | 0.670417146779144 | 0.609722222 | 0.043055556 | 1.71023719749506e-20 | 6 |
| --- | --- | --- | --- | --- | --- | --- |
| MXI1 | 9.94349886537912e-25 | 0.439003120387741 | 0.525 | 0.259027778 | 2.17066580231226e-20 | 6 |
| KIF3A | 1.29042375149077e-24 | 0.442794425547163 | 0.652083333 | 0.536111111 | 2.81699504950434e-20 | 6 |
| BRK1 | 2.09252771727211e-24 | 0.424391163913145 | 1 | 0.692361111 | 4.56798800680501e-20 | 6 |
| DRAXIN | 2.17675164972235e-24 | 0.781638092144887 | 0.559027778 | 0.352083333 | 4.7518488513439e-20 | 6 |
| SERP2 | 2.71310872049082e-24 | 0.308944268862988 | 0.397916667 | 0.138194444 | 5.92271633683145e-20 | 6 |
| GABARAPL1 | 3.15095755009443e-24 | 0.510208433529829 | 0.584027778 | 0.386805556 | 6.87854033185615e-20 | 6 |
| MARCKSL1 | 3.17160000223464e-24 | 0.383799907198739 | 1 | 1 | 6.92360280487821e-20 | 6 |
| YPEL3 | 4.99070035972572e-24 | 0.485353154199064 | 0.575694444 | 00:48 | 1.08946988852813e-19 | 6 |
| RAB6B | 5.11928151065886e-24 | 0.355461887822653 | 0.533333333 | 0.267361111 | 1.11753915377683e-19 | 6 |
| ZBTB18 | 5.53566621205692e-24 | 0.440334461907445 | 0.474305556 | 0.220833333 | 1.20843593409203e-19 | 6 |
| RDX | 1.35630802858446e-23 | 0.551304964331445 | 0.686111111 | 0.675694444 | 2.96082042639987e-19 | 6 |
| UCHL1 | 1.64411698406361e-23 | 0.758832128683531 | 1 | 0.679861111 | 3.58910737621087e-19 | 6 |
| TRAPPC1 | 1.75855963952956e-23 | 0.452610838998152 | 1 | 0.679166667 | 3.83893569309303e-19 | 6 |
| DCTN3 | 1.89962658209099e-23 | 0.460509635425378 | 1 | 0.635416667 | 4.14688482870463e-19 | 6 |
| ATP6V1G2 | 2.05440161776466e-23 | 0.312183375453497 | 0.397916667 | 0.140277778 | 4.48475873158025e-19 | 6 |
| DDX5 | 2.44709202356962e-23 | 0.366653762390608 | 1 | 1 | 5.34200188745247e-19 | 6 |
| CYB5A | 2.44785796170583e-23 | 0.615724400701704 | 0.609722222 | 00:57 | 5.34367393040382e-19 | 6 |
| SKIL | 5.26345799980431e-23 | 0.460886242420092 | 0.601388889 | 0.3875 | 1.14901288135728e-18 | 6 |
| TMOD2 | 5.61243905743855e-23 | 0.26044818447507 | 0.338888889 | 0.1 | 1.22519544623884e-18 | 6 |
| PCMT1 | 6.87053031973634e-23 | 0.4823409768423 | 0.686111111 | 0.669444444 | 1.49983676879844e-18 | 6 |
| PODXL2 | 7.26571726305698e-23 | 0.388754739570714 | 0.533333333 | 0.279166667 | 1.58610607852534e-18 | 6 |
| MTSS1 | 1.07998112690503e-22 | 0.327183765101505 | 0.38125 | 0.132638889 | 2.35759880003367e-18 | 6 |
| RNF11 | 1.37593719836939e-22 | 0.38565685331892 | 0.061805556 | 0.047916667 | 3.00367090404037e-18 | 6 |
| SCG5 | 1.56093944506189e-22 | 0.683273795455277 | 0.660416667 | 0.057638889 | 3.40753080857011e-18 | 6 |
| SOGA3 | 1.68719123383799e-22 | 0.308020149595564 | 0.397916667 | 0.146527778 | 3.68313846346834e-18 | 6 |
| STMP1 | 1.69006968385489e-22 | 0.420752796639454 | 0.686111111 | 0.684722222 | 3.68942211985523e-18 | 6 |
| CGNL1 | 1.73374365951777e-22 | 0.356491360989294 | 0.38125 | 0.136805556 | 3.78476240872729e-18 | 6 |
| GET1 | 1.86363993075495e-22 | 0.467451899767179 | 0.64375 | 0.497916667 | 4.06832596883805e-18 | 6 |
| VAT1 | 2.01094279036931e-22 | 0.481012976047484 | 0.64375 | 0.495833333 | 4.38988811137619e-18 | 6 |
| RAB2A | 2.31211202970693e-22 | 0.397669414932711 | 1 | 0.661111111 | 5.04734056085022e-18 | 6 |
| NPDC1 | 2.38213194261567e-22 | 0.433486399424292 | 0.533333333 | 0.306944444 | 5.20019403073002e-18 | 6 |
| RIMS2 | 2.81912262224169e-22 | 0.373836689279399 | 0.457638889 | 0.205555556 | 6.1541446843536e-18 | 6 |
| DYNC1H1 | 3.2570343063296e-22 | 0.59345837740747 | 0.686111111 | 0.646527778 | 7.11010589071751e-18 | 6 |
| DACH1 | 3.41836539842538e-22 | 0.523880325073869 | 0.575694444 | 0.351388889 | 7.46229166476261e-18 | 6 |
| CD151 | 3.58711858079633e-22 | 0.465667974671697 | 0.635416667 | 0.503472222 | 7.83067986187839e-18 | 6 |
| DDAH2 | 3.63243592613316e-22 | 0.528639735557862 | 1 | 0.679166667 | 7.9296076267487e-18 | 6 |
| ARL8A | 5.96260072881752e-22 | 0.402309911741251 | 0.609722222 | 00:59 | 1.30163573910086e-17 | 6 |
| RIOK3 | 6.32395019688864e-22 | 0.477593232251698 | 0.652083333 | 0.538888889 | 1.38051832798079e-17 | 6 |
| EVL | 6.65776824215252e-22 | 0.454765412636027 | 0.64375 | 0.566666667 | 1.45339080726189e-17 | 6 |
| CUTA | 7.33669738485712e-22 | 0.422388818427986 | 1 | 0.691666667 | 1.60160103911431e-17 | 6 |
| WSB1 | 7.55922218591095e-22 | 0.486354287634369 | 1 | 0.06875 | 1.65017820318436e-17 | 6 |
| CPEB4 | 7.96670740604677e-22 | 0.361477859765342 | 0.042361111 | 0.176388889 | 1.73913222674001e-17 | 6 |
| JUN | 8.96440127912611e-22 | 0.397368740302965 | 0.363888889 | 0.117361111 | 1.95692879923323e-17 | 6 |
| ARMCX3 | 1.18510627308762e-21 | 0.575812748825356 | 0.686111111 | 0.613194444 | 2.58708699415026e-17 | 6 |
| COX7A2 | 1.30422647026551e-21 | 0.320344765500864 | 1 | 1 | 2.84712638458962e-17 | 6 |
| BEX4 | 1.98285987764925e-21 | 0.441958608003688 | 0.677777778 | 0.654166667 | 4.3285831129083e-17 | 6 |
| GSTA4 | 2.0081433789139e-21 | 0.435269112253348 | 0.652083333 | 0.539583333 | 4.38377699616905e-17 | 6 |
| SLC5A3 | 2.73888256516862e-21 | 0.590316151153622 | 0.061805556 | 0.475694444 | 5.97898063976311e-17 | 6 |
| CBFA2T2 | 3.25524641380083e-21 | 0.335415194630025 | 0.415277778 | 0.16875 | 7.10620292132721e-17 | 6 |
| TCF25 | 3.42241831751094e-21 | 0.370666609793927 | 1 | 0.6375 | 7.47113918712639e-17 | 6 |
| DPP6 | 3.85299798601945e-21 | 0.255566283722282 | 00:39 | 0.070138889 | 8.41109460348045e-17 | 6 |
| TMEM59 | 4.45369325586405e-21 | 0.451561878812577 | 0.677777778 | 0.665972222 | 9.72241237755123e-17 | 6 |
| BCL7A | 4.50525417565086e-21 | 0.616213552443498 | 0.635416667 | 0.539583333 | 9.83496986544583e-17 | 6 |
| DCTN1 | 4.70249062218851e-21 | 0.456288993209049 | 0.626388889 | 0.499305556 | 1.02655370282375e-16 | 6 |
| DYNC1LI1 | 4.99272020694035e-21 | 0.417978930266256 | 0.677777778 | 0.065972222 | 1.08991082117508e-16 | 6 |
| AK1 | 5.39458367560979e-21 | 0.347462014346292 | 0.533333333 | 0.293055556 | 1.17763761638562e-16 | 6 |
| HERPUD1 | 6.257433676526e-21 | 0.54851782915425 | 0.601388889 | 0.440972222 | 1.36599777158563e-16 | 6 |
| TCEAL7 | 8.64680626978839e-21 | 0.85195089603198 | 0.490972222 | 0.2625 | 1.8875978086948e-16 | 6 |
| GNB2 | 1.39440191690914e-20 | 0.34254972940409 | 0.66875 | 0.586111111 | 3.04397938461265e-16 | 6 |
| HSBP1 | 1.40041006606756e-20 | 0.335672988804772 | 1 | 1 | 3.05709517422548e-16 | 6 |
| FTX | 1.4541262047027e-20 | 0.564594514664772 | 0.660416667 | 0.60625 | 3.17435750486599e-16 | 6 |
| NOVA1 | 1.75376914692524e-20 | 0.489020116755557 | 1 | 0.067361111 | 3.82847804773779e-16 | 6 |
| TMEM35A | 1.76389833079732e-20 | 0.389882353764096 | 0.482638889 | 0.231944444 | 3.85059005613054e-16 | 6 |
| SSR4 | 1.84486865124464e-20 | 0.419391503297996 | 1 | 0.685416667 | 4.02734826566705e-16 | 6 |
| CHRNA5 | 2.00153364488385e-20 | 0.508678495329459 | 0.054166667 | 0.325694444 | 4.36934794678145e-16 | 6 |
| RMC1 | 2.5156446085194e-20 | 0.615569399957399 | 0.516666667 | 0.276388889 | 5.49165218039784e-16 | 6 |
| TSPYL4 | 2.80428793974272e-20 | 0.484196914482363 | 0.061805556 | 0.483333333 | 6.12176057245835e-16 | 6 |
| ZNF428 | 2.97274619439711e-20 | 0.490003550022735 | 1 | 0.644444444 | 6.48950494236888e-16 | 6 |
| DIPK2A | 3.48309066190615e-20 | 0.465434446659564 | 0.05 | 0.277083333 | 7.60358691494112e-16 | 6 |
| SERINC3 | 4.34859497783113e-20 | 0.381492993900655 | 0.061805556 | 0.461111111 | 9.49298283660535e-16 | 6 |
| AC010642.2 | 4.47433669362582e-20 | 0.401941649043015 | 0.677777778 | 0.633333333 | 9.76747700218517e-16 | 6 |
| MGST3 | 5.25937087967471e-20 | 0.365513230327442 | 0.686111111 | 0.676388889 | 1.14812066303299e-15 | 6 |
| TCAF1 | 5.64301273899908e-20 | 0.498371326036172 | 0.686111111 | 0.616666667 | 1.2318696809235e-15 | 6 |
| RHOU | 6.53461902995714e-20 | 0.46550732298475 | 0.054166667 | 0.290277778 | 1.42650733423964e-15 | 6 |
| HSPA12A | 6.681658985442e-20 | 0.26663782472521 | 0.313194444 | 00:14 | 1.45860615652199e-15 | 6 |
| NAT14 | 7.12123451552999e-20 | 0.433796333724143 | 0.626388889 | 0.514583333 | 1.5545654947402e-15 | 6 |
| PNPLA8 | 7.94843973230051e-20 | 0.386859165407505 | 0.054166667 | 00:46 | 1.7351443935612e-15 | 6 |
| ARL6IP5 | 8.9889517761486e-20 | 0.431025453980146 | 0.66875 | 0.630555556 | 1.96228817273324e-15 | 6 |
| ENC1 | 1.05700416565596e-19 | 0.738380877523504 | 0.584027778 | 0.425694444 | 2.30744009362697e-15 | 6 |
| TSC22D1 | 1.2907279864904e-19 | 0.553140589871292 | 0.686111111 | 0.688888889 | 2.81765919450855e-15 | 6 |
| PRRT2 | 1.80195914096915e-19 | 0.434031059093087 | 0.593055556 | 0.403472222 | 3.93367680473566e-15 | 6 |
| GNAS | 1.80204387125781e-19 | 0.362829443409081 | 1 | 1 | 3.9338617709558e-15 | 6 |
| OTUD6B-AS1 | 2.04495313727829e-19 | 0.371270314270454 | 0.652083333 | 0.580555556 | 4.46413269867851e-15 | 6 |
| MAPK8IP1 | 2.18722816817069e-19 | 0.316804060307308 | 0.372916667 | 0.144444444 | 4.77471909111661e-15 | 6 |
| OS9 | 4.10704101124979e-19 | 0.386328206839199 | 0.660416667 | 0.547916667 | 8.96567052755828e-15 | 6 |
| CEP170 | 4.87358583554018e-19 | 0.531490089555432 | 0.660416667 | 0.6125 | 1.06390378789842e-14 | 6 |
| WBP2 | 5.56884159943905e-19 | 0.32275312506723 | 0.525 | 0.310416667 | 1.21567812115754e-14 | 6 |
| LSAMP | 5.65439067897788e-19 | 0.473186735546541 | 0.593055556 | 0.45 | 1.23435348522087e-14 | 6 |
| BMPR2 | 6.71929035660789e-19 | 0.315797825926027 | 0.465972222 | 0.228472222 | 1.4668210848475e-14 | 6 |
| STX12 | 7.1935441934831e-19 | 0.40840046748565 | 0.525 | 0.315277778 | 1.57035069743736e-14 | 6 |

| USP11 | 1.20607081595782e-18 | 0.351635663392406 | 0.686111111 | 0.065972222 | 2.63285259123592e-14 | 6 |
| --- | --- | --- | --- | --- | --- | --- |
| MAPKAPK2 | 1.28389402835201e-18 | 0.354231973253746 | 0.054166667 | 0.361805556 | 2.80274066389244e-14 | 6 |
| ENOX2 | 1.36689549188742e-18 | 0.417533011677104 | 0.482638889 | 0.253472222 | 2.98393285879024e-14 | 6 |
| VAV2 | 1.44218803889261e-18 | 0.254235575996128 | 0.40625 | 0.175694444 | 3.14829648890256e-14 | 6 |
| LMBR1L | 1.45124545277546e-18 | 0.276172348660905 | 0.465972222 | 0.239583333 | 3.16806882340883e-14 | 6 |
| ZCCHC17 | 1.49646307113156e-18 | 0.498751653681432 | 0.677777778 | 0.640277778 | 3.2667788842802e-14 | 6 |
| PPP1R15A | 1.65599157793011e-18 | 0.416093047217732 | 0.516666667 | 0.304861111 | 3.61502961462143e-14 | 6 |
| TMEM158 | 1.90825961587035e-18 | 0.441405848561413 | 0.372916667 | 0.152083333 | 4.16573074144498e-14 | 6 |
| PHYHIPL | 2.21660637982537e-18 | 0.288856956433546 | 0.40625 | 0.159027778 | 4.83885172715879e-14 | 6 |
| SPSB3 | 2.37572598331785e-18 | 0.310132206907856 | 0.550694444 | 0.350694444 | 5.18620982158286e-14 | 6 |
| CUL1 | 2.46142077997312e-18 | 0.424203537690552 | 0.609722222 | 0.521527778 | 5.37328156268133e-14 | 6 |
| KLF13 | 2.66143422524143e-18 | 0.373202918847846 | 0.474305556 | 00:38 | 5.80991091370204e-14 | 6 |
| BICD1 | 2.8224763189622e-18 | 0.469086219223218 | 0.550694444 | 0.408333333 | 6.16146580429448e-14 | 6 |
| MTMR6 | 3.38857968931629e-18 | 0.316168064572262 | 0.448611111 | 0.229861111 | 7.39726946177747e-14 | 6 |
| UBC | 3.94837056077425e-18 | 0.402723027595887 | 1 | 1 | 8.61929293417018e-14 | 6 |
| TAX1BP1 | 3.96052963084041e-18 | 0.364328175246789 | 0.686111111 | 0.6625 | 8.64583618412462e-14 | 6 |
| ATP6V1D | 5.15575576695715e-18 | 0.364940919456208 | 0.66875 | 0.61875 | 1.12550148392675e-13 | 6 |
| FMC1 | 6.19746187283036e-18 | 0.370924098642453 | 0.061805556 | 0.5375 | 1.35290592683887e-13 | 6 |
| CHCHD2 | 7.14583191534672e-18 | 0.402236642996597 | 1 | 0.690972222 | 1.55993510712019e-13 | 6 |
| COX6A1 | 7.59817517584859e-18 | 0.285973429180501 | 1 | 1 | 1.65868164088775e-13 | 6 |
| EIF1B | 7.70897736742583e-18 | 0.296005334454156 | 1 | 0.69375 | 1.68286975930906e-13 | 6 |
| CYP51A1 | 8.79912860614302e-18 | 0.400597849004133 | 0.686111111 | 0.595833333 | 1.92084977472102e-13 | 6 |
| ZNHIT1 | 1.04889753303524e-17 | 0.372372493897256 | 0.66875 | 0.669444444 | 2.28974331461593e-13 | 6 |
| MIEN1 | 1.13136032781506e-17 | 0.372785278696814 | 0.061805556 | 0.531944444 | 2.46975959562027e-13 | 6 |
| TP53I11 | 1.14775937023459e-17 | 0.414162808098982 | 0.593055556 | 0.047916667 | 2.50555870522212e-13 | 6 |
| UBE2M | 1.25486342750371e-17 | 0.36053729935126 | 1 | 0.66875 | 2.7393668622406e-13 | 6 |
| KLHDC8B | 1.258857020716e-17 | 0.423429823285885 | 0.601388889 | 0.463194444 | 2.74808487622304e-13 | 6 |
| SSBP3 | 1.33101527208714e-17 | 0.337038036193225 | 0.660416667 | 0.596527778 | 2.90560633896623e-13 | 6 |
| HIST1H2AC | 1.68705146342555e-17 | 0.418136201688391 | 0.372916667 | 0.157638889 | 3.68283334465799e-13 | 6 |
| LITAF | 2.33981841582779e-17 | 0.754560056857871 | 0.584027778 | 0.475 | 5.10782360175206e-13 | 6 |
| LAMTOR5 | 2.49895828388522e-17 | 0.394820962962456 | 0.66875 | 0.665277778 | 5.45522593372144e-13 | 6 |
| MYL6 | 2.60899038122278e-17 | 0.608913181445617 | 1 | 1 | 5.69542600220933e-13 | 6 |
| ITM2C | 3.05910615251321e-17 | 0.485249055634394 | 0.660416667 | 0.598611111 | 6.67802873093635e-13 | 6 |
| ATP6V0E1 | 3.17525432949101e-17 | 0.319188798602858 | 0.686111111 | 0.670138889 | 6.93158020127888e-13 | 6 |
| NEAT1 | 3.26602499282063e-17 | 0.667307095720002 | 0.550694444 | 0.350694444 | 7.12973255932744e-13 | 6 |
| METRN | 3.62461052717507e-17 | 0.539600563704292 | 1 | 0.677777778 | 7.91252478082319e-13 | 6 |
| C12orf76 | 3.68027116747043e-17 | 0.405848119665011 | 0.061805556 | 0.5125 | 8.03403195858795e-13 | 6 |
| PRDX5 | 4.29814081657358e-17 | 0.398134248735832 | 1 | 0.688888889 | 9.38284140258013e-13 | 6 |
| NCS1 | 4.46046511432972e-17 | 0.287805998803388 | 0.533333333 | 0.340972222 | 9.73719534458177e-13 | 6 |
| YWHAG | 4.48743175419251e-17 | 0.362411092972837 | 1 | 0.674305556 | 9.79606351940226e-13 | 6 |
| PLEKHO1 | 5.87689481442936e-17 | 0.415523125716148 | 0.660416667 | 0.627083333 | 1.28292613798993e-12 | 6 |
| CRIM1 | 6.06132419749586e-17 | 0.28233573143173 | 0.338888889 | 0.127777778 | 1.32318707231335e-12 | 6 |
| IDS | 6.1421591306741e-17 | 0.369768178160364 | 0.516666667 | 0.339583333 | 1.34083333822616e-12 | 6 |
| ACSL4 | 6.70284609495577e-17 | 0.412891673332968 | 0.635416667 | 0.554166667 | 1.46323130252885e-12 | 6 |
| PPM1L | 7.27828484598696e-17 | 0.354238299707901 | 0.457638889 | 00:36 | 1.58884958187895e-12 | 6 |
| STARD3NL | 7.33998569725319e-17 | 0.315230952393346 | 0.652083333 | 0.613888889 | 1.60231887771037e-12 | 6 |
| SPAG9 | 8.13547539900026e-17 | 0.440108570346191 | 0.677777778 | 0.643055556 | 1.77597427960176e-12 | 6 |
| NRCAM | 8.74139205298224e-17 | 0.367965800956793 | 0.465972222 | 0.251388889 | 1.90824588516602e-12 | 6 |
| CADM1 | 8.81453479405572e-17 | 0.487250693435794 | 0.635416667 | 0.600694444 | 1.92421294554236e-12 | 6 |
| HOMER3 | 1.03135485721183e-16 | 0.395277636556849 | 0.652083333 | 0.586805556 | 2.25144765329343e-12 | 6 |
| ACTG1 | 1.1329905012311e-16 | 0.41865336275787 | 1 | 1 | 2.47331826418748e-12 | 6 |
| P4HTM | 1.38784424997247e-16 | 0.324562876359419 | 0.516666667 | 0.311111111 | 3.0296639976899e-12 | 6 |
| NDUFA4 | 1.68084854130666e-16 | 0.265490424050794 | 1 | 1 | 3.66929236567245e-12 | 6 |
| CCSER2 | 2.17723137860916e-16 | 0.350648886578115 | 0.567361111 | 0.406944444 | 4.75289609950379e-12 | 6 |
| RMST | 2.22304901496759e-16 | 0.341264507417875 | 0.448611111 | 0.242361111 | 4.85291599967426e-12 | 6 |
| SLC25A36 | 2.28607997664204e-16 | 0.324476929586153 | 0.66875 | 0.627083333 | 4.99051258900957e-12 | 6 |
| CA11 | 2.42384667791697e-16 | 0.353793003928017 | 0.609722222 | 0.490277778 | 5.29125729789275e-12 | 6 |
| POLR2I | 2.76243212084408e-16 | 0.453779382919913 | 0.686111111 | 0.678472222 | 6.03038931980263e-12 | 6 |
| NBDY | 2.8994727323174e-16 | 0.37821096790011 | 0.686111111 | 0.665277778 | 6.32954897464889e-12 | 6 |
| ELOVL4 | 2.90895215711341e-16 | 0.367339349291449 | 0.559027778 | 0.404861111 | 6.35024255897857e-12 | 6 |
| EIF4A2 | 3.1459277744804e-16 | 0.329484789437726 | 1 | 0.681944444 | 6.86756033169071e-12 | 6 |
| CST3 | 3.28240699395211e-16 | 0.472813164871664 | 0.652083333 | 0.056944444 | 7.16549446779746e-12 | 6 |
| ITM2B | 3.90435266123397e-16 | 0.372561981736153 | 1 | 0.684722222 | 8.52320185947375e-12 | 6 |
| FAM32A | 4.49293773405702e-16 | 0.290967694012168 | 0.686111111 | 0.672222222 | 9.80808307344647e-12 | 6 |
| MORF4L2 | 4.66463243671304e-16 | 0.311980494059851 | 1 | 0.692361111 | 1.01828926093446e-11 | 6 |
| POU2F2 | 4.78783864771119e-16 | 0.250337980880253 | 0.279166667 | 0.092361111 | 1.04518517679535e-11 | 6 |
| BTBD17 | 6.99267064523875e-16 | 0.305463658110535 | 0.40625 | 0.197222222 | 1.52650000185562e-11 | 6 |
| CCDC92 | 7.42093713379473e-16 | 0.281669084273091 | 0.474305556 | 0.253472222 | 1.61999057630739e-11 | 6 |
| OPTN | 8.70013872127911e-16 | 0.441484180221516 | 0.508333333 | 0.346527778 | 1.89924028285523e-11 | 6 |
| CALM2 | 9.05869085691347e-16 | 0.321297731159802 | 1 | 1 | 1.97751221406421e-11 | 6 |
| SAT1 | 9.29880661908233e-16 | 0.441989882385111 | 0.66875 | 0.621527778 | 2.02992948494567e-11 | 6 |
| PTTG1IP | 9.56506745192946e-16 | 0.368233309202655 | 0.686111111 | 0.679166667 | 2.0880542247562e-11 | 6 |
| KIFAP3 | 1.01675618272026e-15 | 0.290090939722461 | 0.440277778 | 0.227777778 | 2.21957874687833e-11 | 6 |
| PEG10 | 1.02023573866191e-15 | 0.622035709796731 | 0.677777778 | 0.667361111 | 2.22717461749894e-11 | 6 |
| MAPK8 | 1.04285600451653e-15 | 0.305477153695483 | 0.567361111 | 0.429166667 | 2.27655465785958e-11 | 6 |
| HSD11B1L | 1.21926754266928e-15 | 0.306189517179219 | 0.525 | 0.338888889 | 2.66166104564703e-11 | 6 |
| KDM6B | 1.36252672576857e-15 | 0.323438158917495 | 0.533333333 | 0.336805556 | 2.97439584235279e-11 | 6 |
| HRAS | 1.42843941423638e-15 | 0.333775299567623 | 0.635416667 | 0.547916667 | 3.11828324127802e-11 | 6 |
| RBFOX2 | 1.64550077344845e-15 | 0.334126850007428 | 0.660416667 | 0.639583333 | 3.59212818843797e-11 | 6 |
| TSG101 | 1.65984693280155e-15 | 0.33000454398863 | 0.64375 | 0.597916667 | 3.62344585430579e-11 | 6 |
| CRNDE | 1.77289320520402e-15 | 0.483812106877736 | 0.575694444 | 0.046527778 | 3.87022586696037e-11 | 6 |
| TCEAL4 | 2.15733971798372e-15 | 0.352755809540624 | 0.686111111 | 0.69375 | 4.70947260435846e-11 | 6 |
| JKAMP | 2.36231784340059e-15 | 0.289120498960073 | 0.635416667 | 0.557638889 | 5.15693985214349e-11 | 6 |
| YWHAQ | 2.3692527334202e-15 | 0.304901216195933 | 1 | 0.69375 | 5.17207871705629e-11 | 6 |
| PLXNA1 | 3.03684423347086e-15 | 0.31013514866147 | 0.457638889 | 0.259027778 | 6.62943096166688e-11 | 6 |
| UQCR10 | 3.2857845599973e-15 | 0.269961779138482 | 1 | 1 | 7.1728676944741e-11 | 6 |
| CCNI | 3.45562818955543e-15 | 0.300145696642073 | 1 | 1 | 7.5436363377995e-11 | 6 |
| FSTL1 | 4.48829228819636e-15 | 0.79432412983839 | 0.601388889 | 0.591666667 | 9.79794206513265e-11 | 6 |
| TMEM9B | 4.48853223241634e-15 | 0.290999336378424 | 0.567361111 | 0.438194444 | 9.79846586336488e-11 | 6 |
| DYNLL1 | 4.92414986149234e-15 | 0.378415090725872 | 1 | 1 | 1.07494191476378e-10 | 6 |
| MLF2 | 5.21523399344044e-15 | 0.39826755595526 | 0.677777778 | 0.067361111 | 1.13848558076805e-10 | 6 |

| MEIS3 | 5.2154223241076e-15 | 0.313092844022675 | 0.559027778 | 0.395138889 | 1.13852669335269e-10 | 6 |
| --- | --- | --- | --- | --- | --- | --- |
| PFN2 | 5.38238076609863e-15 | 0.332672626780198 | 0.686111111 | 0.677777778 | 1.17497372123933e-10 | 6 |
| ARHGEF9 | 5.97592328040734e-15 | 0.381809439843362 | 0.054166667 | 0.461805556 | 1.30454405211292e-10 | 6 |
| KAT6B | 6.17625008162856e-15 | 0.357424806960215 | 0.061805556 | 0.051388889 | 1.34827539281951e-10 | 6 |
| C1orf122 | 6.18973515085115e-15 | 0.381796146601985 | 1 | 0.636111111 | 1.35121918343081e-10 | 6 |
| ATP6V0A1 | 6.43551852572284e-15 | 0.326939301119277 | 0.533333333 | 0.370138889 | 1.4048736941653e-10 | 6 |
| BNIP3 | 6.78037602521709e-15 | 0.407183066717763 | 0.652083333 | 0.584027778 | 1.48015608630489e-10 | 6 |
| ATP6V0D1 | 6.80964379483112e-15 | 0.317058935564641 | 0.567361111 | 0.414583333 | 1.48654524041163e-10 | 6 |
| DNAJB9 | 7.30975487922483e-15 | 0.290444976973882 | 0.525 | 0.352777778 | 1.59571949013478e-10 | 6 |
| CFAP36 | 7.63666129314143e-15 | 0.415922086422431 | 0.061805556 | 0.54375 | 1.66708316029277e-10 | 6 |
| TRAF4 | 7.73127700838072e-15 | 0.31711616338755 | 0.677777778 | 0.059027778 | 1.68773777092951e-10 | 6 |
| MSRB2 | 8.48345712082998e-15 | 0.356333193816208 | 0.66875 | 0.620833333 | 1.85193868947718e-10 | 6 |
| PPP1R2 | 8.61479108648475e-15 | 0.48863282028093 | 0.593055556 | 0.504861111 | 1.88060889417962e-10 | 6 |
| YIPF3 | 8.62124383655357e-15 | 0.314684408477838 | 0.652083333 | 0.608333333 | 1.88201752951964e-10 | 6 |
| NDUFA5 | 8.93544894898294e-15 | 0.311571498157986 | 0.686111111 | 0.067361111 | 1.95060850556298e-10 | 6 |
| BMERB1 | 1.10752006925121e-14 | 0.303023455913808 | 0.626388889 | 0.511805556 | 2.41771631117538e-10 | 6 |
| ATP1B2 | 1.37097350983412e-14 | 0.392444693850358 | 0.440277778 | 0.251388889 | 2.99283517196788e-10 | 6 |
| ZBTB16 | 1.39018314035624e-14 | 0.465860511255438 | 0.601388889 | 0.475 | 3.03476979539767e-10 | 6 |
| YPEL5 | 1.79932054936547e-14 | 0.297690738398326 | 0.626388889 | 0.480555556 | 3.92791675926482e-10 | 6 |
| AKIRIN2 | 1.89257666816641e-14 | 0.393022825387919 | 0.626388889 | 0.054166667 | 4.13149486660726e-10 | 6 |
| MIB1 | 2.48106206507912e-14 | 0.428919094800625 | 0.609722222 | 0.558333333 | 5.41615848806772e-10 | 6 |
| BSCL2 | 2.6158880180302e-14 | 0.531876735531649 | 0.516666667 | 0.435416667 | 5.71048354335992e-10 | 6 |
| NAPA | 2.65821137715745e-14 | 0.309757597696671 | 0.559027778 | 0.434027778 | 5.80287543633472e-10 | 6 |
| MT-ND2 | 3.3431114316194e-14 | 0.400994403974071 | 1 | 0.693055556 | 7.29801225522515e-10 | 6 |
| SBDS | 3.34520583340137e-14 | 0.286913367684655 | 0.66875 | 0.596527778 | 7.30258433431519e-10 | 6 |
| SEMA6D | 3.67713652819166e-14 | 0.2669554785501 | 00:39 | 0.092361111 | 8.02718904104238e-10 | 6 |
| NDN | 3.77479876071612e-14 | 0.303618133145024 | 0.635416667 | 0.603472222 | 8.2403856946433e-10 | 6 |
| ARMCX1 | 3.83469060807956e-14 | 0.355456293766459 | 0.550694444 | 0.413194444 | 8.37112959743767e-10 | 6 |
| SNX30 | 3.87099639429651e-14 | 0.250000684019859 | 0.389583333 | 0.190972222 | 8.45038512874929e-10 | 6 |
| ATP5F1B | 4.52347198928728e-14 | 0.29328869898928 | 1 | 0.69375 | 9.87473935261413e-10 | 6 |
| ANK2 | 4.94149768281506e-14 | 0.557411909993795 | 0.054166667 | 0.431944444 | 1.07872894415853e-09 | 6 |
| GRB2 | 5.11858289048992e-14 | 0.332462664770247 | 0.677777778 | 0.635416667 | 1.11738664499395e-09 | 6 |
| ARPC5 | 5.62716044649777e-14 | 0.318494973159794 | 1 | 0.682638889 | 1.22840912547046e-09 | 6 |
| FEZF1 | 5.81736035159646e-14 | 0.650914721160177 | 0.575694444 | 0.51875 | 1.26992976475351e-09 | 6 |
| RHBDD2 | 5.90780654912067e-14 | 0.304619832714221 | 0.575694444 | 0.464583333 | 1.28967416967304e-09 | 6 |
| CAP1 | 6.58946505912643e-14 | 0.290119230496451 | 1 | 0.670833333 | 1.4384802224073e-09 | 6 |
| SPAST | 7.48095551175828e-14 | 0.331885721683023 | 0.593055556 | 0.475694444 | 1.63309258821683e-09 | 6 |
| SOX11 | 7.89005832037624e-14 | 0.433909809940618 | 0.686111111 | 0.06875 | 1.72239973133813e-09 | 6 |
| PTMS | 7.90365381929034e-14 | 0.283183494766312 | 1 | 1 | 1.72536762875108e-09 | 6 |
| AASDHPPT | 8.13396699072137e-14 | 0.297263134320058 | 0.652083333 | 0.644444444 | 1.77564499407448e-09 | 6 |
| TUBB4A | 8.16717743359315e-14 | 0.259625415656776 | 00:39 | 0.097916667 | 1.78289483375338e-09 | 6 |
| RCN2 | 8.44679255843164e-14 | 0.311834793910987 | 1 | 0.69375 | 1.84393481550563e-09 | 6 |
| HMGCS1 | 8.46658093607593e-14 | 0.413332151590351 | 0.609722222 | 0.477083333 | 1.84825461834538e-09 | 6 |
| FAM155A | 8.50802102696157e-14 | 0.267885408544143 | 0.372916667 | 0.177777778 | 1.85730099018571e-09 | 6 |
| DMRTA2 | 8.66100726581346e-14 | 0.27798539854181 | 0.296527778 | 0.105555556 | 1.89069788612708e-09 | 6 |
| DPYSL3 | 9.41792113330421e-14 | 0.364685051421677 | 0.66875 | 0.063888889 | 2.05593218340031e-09 | 6 |
| PRKX | 1.03511879652941e-13 | 0.320555916470853 | 0.550694444 | 0.452777778 | 2.2596643328237e-09 | 6 |
| POU3F1 | 1.08138086375988e-13 | 0.674444877376387 | 0.635416667 | 0.595833333 | 2.36065442558782e-09 | 6 |
| BAD | 1.40085320320728e-13 | 0.359515348679632 | 0.575694444 | 0.471527778 | 3.05806254260149e-09 | 6 |
| YIPF4 | 1.49616325791114e-13 | 0.296971962626495 | 0.584027778 | 0.488194444 | 3.26612439202003e-09 | 6 |
| TMEM14A | 1.73029878990464e-13 | 0.259957000923992 | 0.508333333 | 0.336805556 | 3.77724225836183e-09 | 6 |
| SARS | 1.81559398241231e-13 | 0.398567481239585 | 0.660416667 | 0.663194444 | 3.96344166360607e-09 | 6 |
| WDR83OS | 1.89559058840112e-13 | 0.28047009174279 | 1 | 0.690277778 | 4.13807425447965e-09 | 6 |
| GNG2 | 2.15090296911516e-13 | 0.316813192906628 | 0.448611111 | 0.284027778 | 4.6954211815784e-09 | 6 |
| TULP4 | 2.88697976374019e-13 | 0.369344534378687 | 0.593055556 | 0.534027778 | 6.30227682424483e-09 | 6 |
| UBE2L6 | 3.31423010216029e-13 | 0.481911402148617 | 0.626388889 | 0.636805556 | 7.23496431301591e-09 | 6 |
| ACTR1A | 3.48121737079828e-13 | 0.322932393991297 | 0.559027778 | 0.488888889 | 7.59949752045264e-09 | 6 |
| FSIP2 | 3.64016756712588e-13 | 0.330762610854445 | 0.448611111 | 0.255555556 | 7.94648579903579e-09 | 6 |
| STARD4-AS1 | 4.07736820727172e-13 | 0.330290953354375 | 0.508333333 | 0.35625 | 8.90089479647417e-09 | 6 |
| MCRIP1 | 4.11171574975887e-13 | 0.344490702464909 | 0.660416667 | 0.611805556 | 8.97587548172361e-09 | 6 |
| IER5L | 4.53838907627919e-13 | 0.32207756912379 | 0.465972222 | 0.28125 | 9.90730335351748e-09 | 6 |
| SGCB | 5.42834896600601e-13 | 0.365855997883859 | 0.593055556 | 0.049305556 | 1.18500857927911e-08 | 6 |
| BAG6 | 5.67555874317077e-13 | 0.284310975928749 | 0.635416667 | 0.515277778 | 1.23897447363418e-08 | 6 |
| ATP6V1E1 | 5.99452506822957e-13 | 0.33112265070632 | 0.061805556 | 0.53125 | 1.30860482239451e-08 | 6 |
| C9orf16 | 6.02117534114792e-13 | 0.340736568962818 | 0.686111111 | 0.68125 | 1.31442257697259e-08 | 6 |
| TMTC4 | 6.0263693471894e-13 | 0.272297843864629 | 0.448611111 | 0.281944444 | 1.31555642849145e-08 | 6 |
| COX6C | 6.1652446095818e-13 | 0.25924403939509 | 1 | 1 | 1.34587289827171e-08 | 6 |
| CTXN1 | 6.18448884078387e-13 | 0.309696790827344 | 0.64375 | 0.526388889 | 1.35007391394312e-08 | 6 |
| PDLIM4 | 6.53524481112087e-13 | 0.313345062771845 | 0.465972222 | 0.290972222 | 1.42664394226768e-08 | 6 |
| TMEM169 | 7.22680994584311e-13 | 0.337877774234279 | 0.567361111 | 0.472916667 | 1.57761261117755e-08 | 6 |
| SCRN1 | 7.93588943613759e-13 | 0.29457276925305 | 0.559027778 | 0.429166667 | 1.73240466390884e-08 | 6 |
| RCOR2 | 8.83091269642775e-13 | 0.34271938164021 | 0.626388889 | 0.570138889 | 1.92778824163018e-08 | 6 |
| CASD1 | 9.87336367547218e-13 | 0.342252715675609 | 0.567361111 | 0.475 | 2.15535529035558e-08 | 6 |
| CDIPT | 1.02857034546388e-12 | 0.320577143047742 | 0.05 | 0.363194444 | 2.24536906414766e-08 | 6 |
| LEMD1 | 1.09107688239609e-12 | 0.399370002988145 | 0.05 | 0.341666667 | 2.38182083427066e-08 | 6 |
| MAP3K13 | 1.15113413979214e-12 | 0.398262200620919 | 0.593055556 | 0.517361111 | 2.51292582716623e-08 | 6 |
| IER2 | 1.16220802852352e-12 | 0.504854672887178 | 0.64375 | 0.58125 | 2.53710012626684e-08 | 6 |
| TSPAN3 | 1.22570297679117e-12 | 0.389717775485661 | 0.686111111 | 0.679861111 | 2.67570959833511e-08 | 6 |
| AP2B1 | 1.26964112106532e-12 | 0.315779147796226 | 1 | 0.646527778 | 2.77162656728559e-08 | 6 |
| PSMD8 | 1.56533306447721e-12 | 0.27329454860306 | 0.686111111 | 0.684722222 | 3.41712207975376e-08 | 6 |
| CLTB | 1.6611713864861e-12 | 0.338577499542629 | 0.575694444 | 0.463194444 | 3.62633713669916e-08 | 6 |
| ETFB | 1.74435916923565e-12 | 0.469989977975839 | 0.601388889 | 0.563194444 | 3.80793606644143e-08 | 6 |
| AP2A2 | 2.0208222539268e-12 | 0.270356728348889 | 0.533333333 | 0.403472222 | 4.41145498032221e-08 | 6 |
| TMEM59L | 2.11772860239822e-12 | 0.279017340033864 | 0.288194444 | 0.120833333 | 4.62300153903531e-08 | 6 |
| CCDC28B | 2.21532860432093e-12 | 0.305183179885174 | 0.550694444 | 0.415972222 | 4.83606234323259e-08 | 6 |
| RNF165 | 2.23889736633571e-12 | 0.270468752091699 | 0.508333333 | 0.352083333 | 4.88751295071086e-08 | 6 |
| CHMP1B | 2.33560974122781e-12 | 0.304150160592663 | 0.054166667 | 0.40625 | 5.09863606510032e-08 | 6 |
| GUK1 | 2.33670135212935e-12 | 0.312128605019631 | 1 | 0.672916667 | 5.10101905169838e-08 | 6 |
| CCDC112 | 2.40373046985787e-12 | 0.327532642981 | 0.559027778 | 0.43125 | 5.24734361569972e-08 | 6 |
| GYPC | 2.469976132877e-12 | 0.500680873520576 | 0.474305556 | 0.321527778 | 5.39195789807048e-08 | 6 |
| NDFIP1 | 2.65735176018633e-12 | 0.275358032636038 | 0.64375 | 0.604861111 | 5.80099889248675e-08 | 6 |

| ETNK1 | 2.7607907487117e-12 | 0.305705703636564 | 0.054166667 | 0.407638889 | 6.02680620443763e-08 | 6 |
| --- | --- | --- | --- | --- | --- | --- |
| ENO2 | 2.79542379583389e-12 | 0.507158130283355 | 0.601388889 | 0.495138889 | 6.10241014630538e-08 | 6 |
| CYTH2 | 3.29744979057409e-12 | 0.286579140062761 | 0.508333333 | 0.384722222 | 7.19833289282324e-08 | 6 |
| NARF | 3.5091614173226e-12 | 0.36755778803667 | 0.660416667 | 0.634027778 | 7.66049937401524e-08 | 6 |
| SNAPC3 | 3.60085992216248e-12 | 0.296228730377322 | 0.054166667 | 0.426388889 | 7.86067721008069e-08 | 6 |
| EPHB2 | 3.72927883534217e-12 | 0.316977821656565 | 0.550694444 | 0.395138889 | 8.14101569755197e-08 | 6 |
| C1QL1 | 3.75780613811315e-12 | 0.376440258859816 | 00:28 | 0.085 | 8.20329079950102e-08 | 6 |
| TSPAN13 | 4.03527164371874e-12 | 0.307225178250867 | 0.508333333 | 0.375694444 | 8.80899799823801e-08 | 6 |
| ARHGAP21 | 4.22430155906233e-12 | 0.327515489228772 | 0.559027778 | 0.438194444 | 9.22165030343307e-08 | 6 |
| PTPRG | 4.2702339570926e-12 | 0.389108783359588 | 0.567361111 | 0.453472222 | 9.32192072833316e-08 | 6 |
| GADD45A | 4.30666777816262e-12 | 0.415319864335483 | 0.05 | 0.327083333 | 9.40145575972899e-08 | 6 |
| RTN3 | 4.6974052886895e-12 | 0.275253826386562 | 1 | 0.69375 | 1.02544357452092e-07 | 6 |
| RTL8A | 4.86896286921666e-12 | 0.294637559521329 | 0.593055556 | 0.050694444 | 1.06289459435e-07 | 6 |
| NABP2 | 5.44649903421821e-12 | 0.257031033622337 | 0.054166667 | 0.407638889 | 1.18897073916984e-07 | 6 |
| FNBP1L | 5.56586010669545e-12 | 0.432531690369108 | 0.686111111 | 0.066666667 | 1.21502726129162e-07 | 6 |
| LRCH2 | 5.84487278202949e-12 | 0.302157646527599 | 0.457638889 | 00:45 | 1.27593572831704e-07 | 6 |
| SNX4 | 5.88976141707327e-12 | 0.294841610043502 | 0.601388889 | 0.522222222 | 1.28573491734709e-07 | 6 |
| TMX4 | 6.32483064325284e-12 | 0.255436457044635 | 0.550694444 | 0.433333333 | 1.3807105294221e-07 | 6 |
| CELSR2 | 6.39959985471706e-12 | 0.29972344197791 | 0.448611111 | 0.295138889 | 1.39703264828473e-07 | 6 |
| RABAC1 | 6.42036113854538e-12 | 0.280984605465025 | 0.677777778 | 0.067361111 | 1.40156483654446e-07 | 6 |
| GSK3B | 7.1603125302887e-12 | 0.306206728458112 | 0.609722222 | 0.564583333 | 1.56309622536202e-07 | 6 |
| NT5C3B | 7.45325784750772e-12 | 0.306613064558761 | 0.061805556 | 0.581944444 | 1.62704618811093e-07 | 6 |
| H1FX | 7.48767290341813e-12 | 0.348510000082725 | 1 | 0.689583333 | 1.63455899481618e-07 | 6 |
| RNF187 | 7.69323973164588e-12 | 0.303785406085791 | 0.601388889 | 0.524305556 | 1.6794342334183e-07 | 6 |
| ATP6V1F | 7.74881588164435e-12 | 0.300204264359702 | 0.686111111 | 0.067361111 | 1.69156650696296e-07 | 6 |
| FAM200B | 7.83590883274462e-12 | 0.290036669127723 | 0.609722222 | 0.509027778 | 1.71057889818815e-07 | 6 |
| SELENOK | 7.86132955498143e-12 | 0.325002016623616 | 1 | 0.671527778 | 1.71612824185245e-07 | 6 |
| CCNDBP1 | 8.61967969671159e-12 | 0.266624261208141 | 0.533333333 | 0.427777778 | 1.88167607779214e-07 | 6 |
| PEPD | 8.91498410482049e-12 | 0.315347354377361 | 0.559027778 | 0.425694444 | 1.94614103008231e-07 | 6 |
| FIS1 | 8.91880654452813e-12 | 0.264154788923342 | 0.660416667 | 0.642361111 | 1.94697546867049e-07 | 6 |
| BCAR1 | 9.0540301191869e-12 | 0.263979082472029 | 0.05 | 0.35 | 1.9764947750185e-07 | 6 |
| PGM2L1 | 1.12656684967965e-11 | 0.27152396841276 | 0.474305556 | 00:44 | 2.45929543285067e-07 | 6 |
| CHST12 | 1.15305130217277e-11 | 0.256353375342143 | 0.042361111 | 0.261805556 | 2.51711099264316e-07 | 6 |
| LINC00632 | 1.18288742362818e-11 | 0.380254354338747 | 0.525 | 0.3875 | 2.58224324578033e-07 | 6 |
| RAB4A | 1.2796939901885e-11 | 0.307041609601321 | 0.64375 | 0.629861111 | 2.7935719805815e-07 | 6 |
| NAGK | 1.50484790102447e-11 | 0.251322591525204 | 0.05 | 0.35 | 3.28508296793642e-07 | 6 |
| VPS28 | 1.58010864522702e-11 | 0.308021868138303 | 0.660416667 | 0.615972222 | 3.44937717253059e-07 | 6 |
| ZEB1 | 1.87693780527100e-11 | 0.327262665927396 | 0.584027778 | 0.464583333 | 4.0973552289066e-07 | 6 |
| MT-ND5 | 1.88920108034258e-11 | 0.340267571776024 | 1 | 0.685416667 | 4.12412595838785e-07 | 6 |
| CHIC2 | 1.93726239482815e-11 | 0.278739539065094 | 0.440277778 | 00:39 | 4.22904380790986e-07 | 6 |
| SMARCA5 | 2.19588243600331e-11 | 0.269523232941145 | 1 | 0.691666667 | 4.79361135779522e-07 | 6 |
| MINDY2 | 2.21741957368723e-11 | 0.30787576648901 | 0.054166667 | 0.434722222 | 4.84062692935922e-07 | 6 |
| EIF4G2 | 2.22771783375253e-11 | 0.260646269726995 | 1 | 0.693055556 | 4.86310803108177e-07 | 6 |
| S100A13 | 2.5832051657628e-11 | 0.398292350629751 | 0.677777778 | 0.676388889 | 5.6391368768602e-07 | 6 |
| PPP1R18 | 2.63734019452606e-11 | 0.256388292658043 | 0.448611111 | 0.293055556 | 5.7573136446504e-07 | 6 |
| MYO6 | 2.68971381328914e-11 | 0.336777437096985 | 0.626388889 | 0.595833333 | 5.87164525441019e-07 | 6 |
| HCFC1R1 | 2.86788348423163e-11 | 0.294240405901062 | 0.061805556 | 0.50625 | 6.26058964607765e-07 | 6 |
| KIF5B | 2.94550483754372e-11 | 0.283886573607778 | 0.677777778 | 0.684027778 | 6.43003706035794e-07 | 6 |
| ACLY | 3.04291007115581e-11 | 0.282827660918873 | 0.584027778 | 0.463888889 | 6.64267268533314e-07 | 6 |
| DDX24 | 3.09668990248557e-11 | 0.292939160491051 | 1 | 0.690277778 | 6.760074057126e-07 | 6 |
| ATP6AP1 | 3.74081944265983e-11 | 0.280885452869455 | 0.508333333 | 0.384722222 | 8.1662088433264e-07 | 6 |
| ZNF385A | 4.06912628164579e-11 | 0.304872471538391 | 0.363888889 | 0.200694444 | 8.88290267283275e-07 | 6 |
| PSD3 | 4.15288328924621e-11 | 0.281183524473492 | 0.516666667 | 0.3875 | 9.06574422042448e-07 | 6 |
| SATB1 | 4.19552065438027e-11 | 0.277131548138301 | 0.431944444 | 0.273611111 | 9.15882158851213e-07 | 6 |
| SHISA5 | 4.49618632826367e-11 | 0.319673813065662 | 0.584027778 | 0.528472222 | 9.81517475459959e-07 | 6 |
| SUPT4H1 | 4.57346556620182e-11 | 0.260798394761597 | 0.66875 | 0.6625 | 9.98387533101857e-07 | 6 |
| PJA2 | 4.64224922967903e-11 | 0.339383922034887 | 0.609722222 | 00:09 | 1.01340300683893e-06 | 6 |
| SLC52A2 | 4.77029766997053e-11 | 0.268605380833502 | 0.054166667 | 0.401388889 | 1.04135598135457e-06 | 6 |
| SNRNP27 | 4.82297858069054e-11 | 0.296863850598992 | 0.061805556 | 0.609027778 | 1.05285622416474e-06 | 6 |
| GHITM | 5.92210925439192e-11 | 0.271396234648557 | 0.677777778 | 0.672916667 | 1.29279645023376e-06 | 6 |
| HES6 | 6.16598317874493e-11 | 0.436972239253859 | 0.389583333 | 00:32 | 1.34603412792002e-06 | 6 |
| CELF5 | 6.31838425513285e-11 | 0.254926409694646 | 0.397916667 | 0.239583333 | 1.3793032828955e-06 | 6 |
| CRIPT | 6.68105187880093e-11 | 0.25454385323533 | 0.584027778 | 0.547916667 | 1.45847362514224e-06 | 6 |
| PRKAR1A | 6.79814841572322e-11 | 0.265298336882051 | 0.64375 | 0.582638889 | 1.48403579915238e-06 | 6 |
| HNRNPH2 | 6.90887047604143e-11 | 0.279745707260256 | 0.601388889 | 0.566666667 | 1.50820642491984e-06 | 6 |
| PKIG | 6.93791598625149e-11 | 0.281119049696791 | 0.635416667 | 00:08 | 1.5145470597987e-06 | 6 |
| PIN1 | 7.00686352304103e-11 | 0.307302075510356 | 0.677777778 | 0.6625 | 1.52959830707986e-06 | 6 |
| OAZ1 | 7.09916408581283e-11 | 0.251362848880543 | 1 | 1 | 1.54974751993294e-06 | 6 |
| RAB11B | 7.21193625734837e-11 | 0.255744763425703 | 0.584027778 | 0.502083333 | 1.57436568497915e-06 | 6 |
| FMNL2 | 7.25254892259585e-11 | 0.259766502500997 | 0.516666667 | 0.407638889 | 1.58323142980267e-06 | 6 |
| FYTTD1 | 7.32805906698591e-11 | 0.300354204204777 | 0.626388889 | 0.568055556 | 1.59971529432302e-06 | 6 |
| MID1 | 7.42661126788609e-11 | 0.354652605562711 | 0.054166667 | 0.420833333 | 1.62122923977953e-06 | 6 |
| MPHOSPH8 | 7.51840862618518e-11 | 0.286679726081523 | 0.635416667 | 0.56875 | 1.64126860309622e-06 | 6 |
| KIF21A | 7.67950457682612e-11 | 0.309530111780596 | 0.652083333 | 0.056944444 | 1.67643584912114e-06 | 6 |
| ACTB | 7.89032141228678e-11 | 0.300290681778368 | 1 | 1 | 1.72245716430221e-06 | 6 |
| ATP1B1 | 9.07585526787503e-11 | 0.368141148824468 | 0.584027778 | 0.49375 | 1.98125920497712e-06 | 6 |
| PRAF2 | 1.02200617171471e-10 | 0.253784689732309 | 0.482638889 | 0.339583333 | 2.23103947285321e-06 | 6 |
| CTNNB1 | 1.0481507771079e-10 | 0.303710021595986 | 0.660416667 | 0.645138889 | 2.28811314642655e-06 | 6 |
| ACTR10 | 1.22729743458088e-10 | 0.253776064730329 | 0.584027778 | 0.545138889 | 2.67919029969007e-06 | 6 |
| COX14 | 1.48706618714212e-10 | 0.251381784852686 | 0.635416667 | 0.542361111 | 3.24626548653124e-06 | 6 |
| NDUFA7 | 1.53941526943559e-10 | 0.281044528755608 | 0.686111111 | 0.686111111 | 3.36054353317789e-06 | 6 |
| PCDHGB6 | 1.560435664836e-10 | 0.271307026028112 | 0.431944444 | 0.272222222 | 3.40643105633699e-06 | 6 |
| DYNLT1 | 1.80373478593597e-10 | 0.328899852364605 | 0.677777778 | 0.677083333 | 3.93755303769823e-06 | 6 |
| NCALD | 1.84214276603975e-10 | 0.263676040706366 | 0.550694444 | 0.372222222 | 4.02139765826477e-06 | 6 |
| BAZ2A | 1.89338541646836e-10 | 0.260678224050649 | 0.061805556 | 0.567361111 | 4.13326036415042e-06 | 6 |
| ZNRF1 | 1.98090995755108e-10 | 0.250065839222414 | 0.601388889 | 0.54375 | 4.32432643733401e-06 | 6 |
| ZNF580 | 2.36615460786515e-10 | 0.288462590649211 | 0.567361111 | 0.518055556 | 5.16531550896963e-06 | 6 |
| PDLIM7 | 2.67062609889366e-10 | 0.284585932569744 | 0.677777778 | 0.660416667 | 5.82997677388486e-06 | 6 |
| TCF12 | 2.88322545786673e-10 | 0.380994970213862 | 0.575694444 | 0.511111111 | 6.29408117452308e-06 | 6 |
| SEMA6A | 2.89172670304172e-10 | 0.328726011291468 | 0.652083333 | 0.573611111 | 6.31263939274008e-06 | 6 |
| SCP2 | 3.38778750608829e-10 | 0.25778381668107 | 0.626388889 | 0.577083333 | 7.39554012579075e-06 | 6 |

| BNIP3L | 3.4476779332836e-10 | 0.268583304845952 | 0.677777778 | 0.66875 | 7.5262809283581e-06 | 6 |
| --- | --- | --- | --- | --- | --- | --- |
| IP6K2 | 3.51833615334573e-10 | 0.267518686823663 | 0.66875 | 00:09 | 7.68052782275373e-06 | 6 |
| CHD2 | 3.6564380736069e-10 | 0.296665616127173 | 0.652083333 | 0.621527778 | 7.98200431468387e-06 | 6 |
| MIR124-2HG | 3.9008194782093e-10 | 0.282078444164755 | 0.304861111 | 0.144444444 | 8.5154889209309e-06 | 6 |
| SETBP1 | 3.93134220957168e-10 | 0.275380921346447 | 0.40625 | 0.234027778 | 8.58212004349499e-06 | 6 |
| INAFM1 | 4.59228606822983e-10 | 0.267851008907378 | 0.321527778 | 0.159027778 | 1.00249604869457e-05 | 6 |
| MAPK6 | 5.72884282050345e-10 | 0.258869604727564 | 0.626388889 | 0.565972222 | 1.2506063877159e-05 | 6 |
| ISCA1 | 5.81123919389377e-10 | 0.332676821413441 | 0.533333333 | 0.456944444 | 1.26859351602701e-05 | 6 |
| ATP11A | 5.89836695091698e-10 | 0.254184952155127 | 0.482638889 | 0.376388889 | 1.28761350538518e-05 | 6 |
| CDS2 | 5.93328232779235e-10 | 0.271118754951842 | 0.533333333 | 0.45 | 1.29523553215707e-05 | 6 |
| ARID3A | 6.14581834098112e-10 | 0.350687983027206 | 0.601388889 | 0.536111111 | 1.34163214383618e-05 | 6 |
| PAK2 | 7.957066791896e-10 | 0.268736909187121 | 0.677777778 | 0.685416667 | 1.7370276806709e-05 | 6 |
| ZNF708 | 9.18493671239841e-10 | 0.287946070771613 | 0.559027778 | 0.489583333 | 2.00507168431657e-05 | 6 |
| SLC4A7 | 1.04718788065085e-09 | 0.295648422428242 | 0.601388889 | 0.594444444 | 2.2860111434608e-05 | 6 |
| ARHGDIA | 1.08371622472529e-09 | 0.283722592879118 | 0.601388889 | 0.529166667 | 2.36575251857531e-05 | 6 |
| ZNF493 | 1.08934353135561e-09 | 0.290352661934144 | 0.482638889 | 0.373611111 | 2.3780369289493e-05 | 6 |
| RUFY2 | 1.19464106526027e-09 | 0.309671616622428 | 0.550694444 | 0.45625 | 2.60790144546318e-05 | 6 |
| MIAT | 1.36524429691e-09 | 0.374846099966747 | 0.061805556 | 0.554166667 | 2.98032830015453e-05 | 6 |
| SMDT1 | 1.41990695615277e-09 | 0.288996809415454 | 0.626388889 | 0.567361111 | 3.09965688528151e-05 | 6 |
| FAM171B | 1.57752811368821e-09 | 0.295884281942868 | 0.482638889 | 0.352777778 | 3.44374387218136e-05 | 6 |
| SH3BGRL2 | 1.7530728394098e-09 | 0.286862243989288 | 0.431944444 | 0.315277778 | 3.8269580084316e-05 | 6 |
| PCMTD1 | 1.89525210818378e-09 | 0.307792242370097 | 0.550694444 | 0.4875 | 4.1373353521652e-05 | 6 |
| MAGEF1 | 2.20725806747691e-09 | 0.254771262997231 | 0.593055556 | 0.528472222 | 4.81844436130209e-05 | 6 |
| PALM | 2.44010155164607e-09 | 0.286559307198552 | 0.431944444 | 0.309722222 | 5.32674168724337e-05 | 6 |
| MCTS1 | 2.56452932705881e-09 | 0.266780118416895 | 0.061805556 | 0.057638889 | 5.59836752096938e-05 | 6 |
| PRDX1 | 4.69580098004245e-09 | 0.273472502742409 | 0.686111111 | 1 | 0.000102509335394327 | 6 |
| PHTF1 | 4.80323367664605e-09 | 0.277830574676985 | 0.490972222 | 0.386805556 | 0.000104854591161183 | 6 |
| MACO1 | 4.88552498742798e-09 | 0.272408778731595 | 0.054166667 | 0.438194444 | 0.000106651010475553 | 6 |
| KMT2E | 4.94552553908433e-09 | 0.257638347197894 | 0.66875 | 0.655555556 | 0.000107960822518211 | 6 |
| NDUFA3 | 7.56281290600793e-09 | 0.250802202866661 | 0.677777778 | 0.684027778 | 0.000165096205738153 | 6 |
| TRIM2 | 8.35520889418911e-09 | 0.277151167022583 | 0.516666667 | 0.436805556 | 0.000182394210160148 | 6 |
| DHPS | 8.51110055295841e-09 | 0.258565280005077 | 0.559027778 | 0.509027778 | 0.000185797325071082 | 6 |
| SHF | 9.10291312207568e-09 | 0.282707578710099 | 0.474305556 | 0.397222222 | 0.000198716593454912 | 6 |
| TRH | 1.11249039149197e-08 | 0.403811324436993 | 0.186111111 | 0.094 | 0.000242856652462697 | 6 |
| HECTD4 | 1.36330032150632e-08 | 0.25755594783892 | 0.38125 | 0.249305556 | 0.00029760846018483 | 6 |
| MRPL41 | 1.46983155385208e-08 | 0.279780999790644 | 0.686111111 | 0.068055556 | 0.000320864228205909 | 6 |
| TESC | 2.09781937088677e-08 | 0.554128899759194 | 0.363888889 | 0.236805556 | 0.000457953968664583 | 6 |
| ZBTB10 | 3.30881889036995e-08 | 0.27515629034111 | 0.516666667 | 0.459027778 | 0.00072231516376776 | 6 |
| ACVR2B | 3.95828792020906e-08 | 0.258502819658139 | 0.593055556 | 0.571527778 | 0.000864094252981637 | 6 |
| HMGCR | 4.47713152055118e-08 | 0.278238775629545 | 0.593055556 | 0.556944444 | 0.000977357810936322 | 6 |
| MT-ND4L | 4.99224064992089e-08 | 0.253093369851719 | 0.66875 | 0.622916667 | 0.00108980613387773 | 6 |
| SDCBP | 7.44056202118151e-08 | 0.27517568513657 | 0.64375 | 0.656944444 | 0.00162427468922392 | 6 |
| ARHGAP15 | 7.91073393022536e-08 | 0.395337560734502 | 0.228472222 | 0.102083333 | 0.0017269132169682 | 6 |
| CERS6 | 9.15490572580159e-08 | 0.276206946101862 | 0.05 | 0.443055556 | 0.00199851591994249 | 6 |
| CLASP2 | 9.46997527262791e-08 | 0.285935393908619 | 0.448611111 | 0.338888889 | 0.00206729560201467 | 6 |
| DDIT3 | 9.76387129634441e-08 | 0.258297523128053 | 0.355555556 | 0.200694444 | 0.00213145310399199 | 6 |
| ANK3 | 1.02408803563368e-07 | 0.399069492217769 | 0.508333333 | 0.438888889 | 0.00223558418178833 | 6 |
| TP53BP1 | 1.05244339484077e-07 | 0.259888549532442 | 0.609722222 | 0.539583333 | 0.00229748393093739 | 6 |
| SQLE | 1.31211254242712e-07 | 0.25814245318281 | 0.609722222 | 0.5875 | 0.0028643416801184 | 6 |
| LCOR | 1.38775278267585e-07 | 0.262702267296015 | 0.061805556 | 0.563888889 | 0.00302946432458138 | 6 |
| SCD | 1.80335038582548e-07 | 0.329825123880627 | 0.609722222 | 0.058333333 | 0.00393671389225702 | 6 |
| RASSF8 | 1.81468958054122e-07 | 0.413684810226555 | 0.533333333 | 0.545138889 | 0.00396146735432149 | 6 |
| ADGRL3 | 1.85118185375138e-07 | 0.254123202851759 | 0.533333333 | 0.450694444 | 0.00404112998673925 | 6 |
| CALM1 | 3.017761627658e-07 | 0.347162920940161 | 1 | 0.69375 | 0.0065877736331774 | 6 |
| AUTS2 | 3.11791245423582e-07 | 0.256076995621671 | 0.533333333 | 0.48125 | 0.0068064028875968 | 6 |
| RB1CC1 | 4.45126031142557e-07 | 0.251815101709203 | 0.64375 | 0.061111111 | 0.00971710125984202 | 6 |
| CCDC82 | 4.45316990841877e-07 | 0.270124935267759 | 0.567361111 | 0.534027778 | 0.00972126991007818 | 6 |
| S100A10 | 5.38679251269903e-07 | 0.591660128281294 | 0.288194444 | 0.154861111 | 0.011759368055222 | 6 |
| SELENOM | 6.26908250708953e-07 | 0.262542678699434 | 0.448611111 | 0.363194444 | 0.0136854071129765 | 6 |
| HMOX2 | 7.29746383315713e-07 | 0.275469126462795 | 0.389583333 | 0.307638889 | 0.015930363547782 | 6 |
| PAFAH1B3 | 1.18863433358051e-06 | 0.284805100465617 | 0.660416667 | 0.654166667 | 0.0259478875020626 | 6 |
| CASP3 | 1.42451426312392e-06 | 0.759358122493808 | 0.533333333 | 0.053472222 | 0.0310971463639951 | 6 |
| EIF4A3 | 2.24686501972651e-06 | 0.266868669848506 | 0.66875 | 0.650694444 | 0.0490490633806297 | 6 |
| TUSC3 | 2.40961291656174e-06 | 0.259118855147643 | 0.601388889 | 0.632638889 | 0.0526018499685428 | 6 |
| TBCA | 4.49832291550132e-06 | 0.310780980033245 | 1 | 0.69375 | 0.0981983892453938 | 6 |
| CXADR | 4.63517613722193e-06 | 0.269571559889346 | 1 | 0.686805556 | 0.101185895075555 | 6 |
| SEPTIN11 | 5.16574107721699e-06 | 0.327086960742761 | 0.686111111 | 0.685416667 | 0.112768127715647 | 6 |
| TRIB2 | 8.94704442533433e-06 | 0.273602966004041 | 0.465972222 | 0.415277778 | 0.195313979805049 | 6 |
| CLU | 1.46471715392155e-05 | 0.342856701140479 | 0.64375 | 0.670138889 | 0.319747754701074 | 6 |
| KMT2A | 1.55089611233969e-05 | 0.256189762325642 | 0.686111111 | 0.675 | 0.338560621323754 | 6 |
| PLEKHA1 | 3.6119363229824e-05 | 0.256626931100145 | 0.533333333 | 0.535416667 | 0.788485699307058 | 6 |
| GPM6B | 4.10895993742667e-05 | 0.282460787947018 | 0.567361111 | 0.549305556 | 0.896985954340242 | 6 |
| NAV2 | 4.61073976930943e-05 | 0.269542046737987 | 0.330555556 | 0.252777778 | 1 | 6 |
| TAC3 | 9.49993649138529e-05 | 0.307356441540299 | 00:39 | 0.190972222 | 1 | 6 |
| CITED2 | 0.000100532874364643 | 0.252904374916143 | 0.061805556 | 0.554166667 | 1 | 6 |
| CNTNAP2 | 0.000101237146531772 | 0.499840899104861 | 0.66875 | 0.677777778 | 1 | 6 |
| DUSP4 | 0.000205079592890303 | 0.257142734917802 | 0.38125 | 0.309722222 | 1 | 6 |
| FAM107B | 0.000245509757112353 | 0.253144508540695 | 0.593055556 | 0.565972222 | 1 | 6 |
| PMEL | 0.000366427383721152 | 0.691718884536011 | 0.482638889 | 0.552083333 | 1 | 6 |
