# Supplementary material for: Deciphering the Transcriptional Landscape of Human Pluripotent Stem Cell-Derived GnRH Neurons: The Role of Wnt Signaling in Patterning the Neural Fate
Source: Stem Cells. 2022 Sep 25;40(12):1107–21. doi: 10.1093/stmcls/sxac069 (PMC9806769; doi:10.1093/stmcls/sxac069)
Supplement: sxac069_suppl_Supplementary_Table_S4 [file sxac069_suppl_supplementary_table_s4.docx]

| Target protein | Host | Manufacturer | Cat. | Dilution ratio |
| --- | --- | --- | --- | --- |
| SOX2 | Mouse | Thermo Fisher Scientific | MA1-014 | 1:500 |
| Ki-67 | Rabbit | Sigma-Aldrich | AB9260 | 1:500 |
| PPP1R17 | Rabbit | ATLAS ANTIBODY | HPA047819 | 1:500 |
| DLX5 | Rabbit | Abcam | ab109737 | 1:500 |
| TUJ1 | Mouse | Sigma-Aldrich | T8578 | 1:1000 |
