# Supplementary material for: Deciphering the Transcriptional Landscape of Human Pluripotent Stem Cell-Derived GnRH Neurons: The Role of Wnt Signaling in Patterning the Neural Fate
Source: Stem Cells. 2022 Sep 25;40(12):1107–21. doi: 10.1093/stmcls/sxac069 (PMC9806769; doi:10.1093/stmcls/sxac069)
Supplement: sxac069_suppl_Supplementary_Table_S5 [file sxac069_suppl_supplementary_table_s5.docx]

|  | | Sequencing(5´-3´) |
| --- | --- | --- |
| *PPIG* | Forward | ACTCCCAGCCTGCTTCATAC |
|  | Reverse | TACGTCTGAAACGATCCCTTG |
| *GNRH1* | Forward | TGCCCAGTTTCCTCTTCAAT |
|  | Reverse | GTCAACTGGCAGAAACCCAA |
| *DLX1* | Forward | ACATCAGTTCGGTGCAGTCC |
|  | Reverse | ATATAGGAGCCCGCGTTTCC |
| *DLX5* | Forward | GACTCAGTACCTCGCCTTGC |
|  | Reverse | TCCGAACTTCCCCATATGAA |
| *FOXG1* | Forward | CCGCACCCGTCAATGACTT |
|  | Reverse | CCGTCGTAAAACTTGGCAAAG |
| *OTX2* | Forward | TCTTAAGCAACCGCCTTACG |
|  | Reverse | TCTCGCATGAAGATGTCTGG |
| *PAX6* | Forward | TTTGCCCGAGAAAGACTAGC |
|  | Reverse | CATTTGGCCCTTCGATTAGA |
| EMX2 | Forward | GCTTCTAAGGCTGGAACACG |
|  | Reverse | CCAGCTTCTGCCTTTTGAAC |
| LHX8 | Forward | AAGGACAGGCTTGAGCAGAC |
|  | Reverse | GGGTGGAGGATGAGTGATTAGG |
| CALM1 | Forward | TGGCAGTGCCCTTGAAGTTG |
|  | Reverse | CAAGTCTCAGGCTAAAGCAGGATG |
| HMGB1 | Forward | GCGGACAAGGCCCGTTAT |
|  | Reverse | GAAGAGGAAGAAGGCCGAAGG |
| BASP1 | Forward | CTTCAGACTCAAAACCCGGC |
|  | Reverse | ACGGTTTGGTCGGAATTAGC |
| SST | Forward | CGGGGAAGCAGGAACTGG |
|  | Reverse | GGCATCATTCTCCGTCTGGT |
| PVALB | Forward | ATGTCGATGACAGACTTGCTG |
|  | Reverse | TTAGCTTTCAGCCACCAGAGT |
| PPP1R1B | Forward | GAAGACCCAGCACTAAGTGAG |
|  | Reverse | AAAACAGGGTGAGGATAGAGTG |
| FOXP1 | Forward | GGCAGGCCATTCTCGAATCT |
|  | Reverse | GACGCACTGCATTCTTCCAC |
| BCL11B | Forward | GTCAGTTGTCAGGTAAAGATGAGC |
|  | Reverse | CAGGTAGATGCGGAAGCCG |
| CHRM4 | Forward | CCGTGTACATCATCAAGGGCT |
|  | Reverse | GTGACGCAGAAGTAGCGGTC |
| PLNXD1 | Forward | CCCCAACCCACAGTTCTCTA |
|  | Reverse | TGGATGTCGCAGCTTACTTG |
