# Supplementary material for: Deciphering the Transcriptional Landscape of Human Pluripotent Stem Cell-Derived GnRH Neurons: The Role of Wnt Signaling in Patterning the Neural Fate
Source: Stem Cells. 2022 Sep 25;40(12):1107–21. doi: 10.1093/stmcls/sxac069 (PMC9806769; doi:10.1093/stmcls/sxac069)
Supplement: sxac069_suppl_Supplementary_Table_S6 [file sxac069_suppl_supplementary_table_s6.docx]

| Sample | nUMI | nGene | log10GenePerUMI | mitoRatio |
| --- | --- | --- | --- | --- |
| Day22 | >=1500 | >=1000 | >0.82 | <0.2 |
| Day24 | >=1500 | >=1000 | >0.82 | <0.2 |
| Day26 | >=1500 | >=1000 | >0.82 | <0.2 |
| Day0 | >=30000 | >=5000 | >0.85 | <0.1 |
| Day5_n | >=10000 | >=3000 | >0.78 | <0.1 |
| Day5_x | >=10000 | >=3000 | >0.78 | <0.1 |
| Day10_n | >=10000 | >=3000 | >0.82 & <0.9 | <0.1 |
| Day10_x | >=10000 | >=3000 | >0.82 & <0.9 | <0.1 |
